# A Smartphone-Based Self-management Intervention for Individuals With Bipolar Disorder (LiveWell): Empirical and Theoretical Framework, Intervention Design, and Study Protocol for a Randomized Controlled Trial

**DOI:** 10.2196/30710

**Published:** 2022-02-21

**Authors:** Evan H Goulding, Cynthia A Dopke, Rebecca C Rossom, Tania Michaels, Clair R Martin, Chloe Ryan, Geneva Jonathan, Alyssa McBride, Pamela Babington, Mary Bernstein, Andrew Bank, C Spencer Garborg, Jennifer M Dinh, Mark Begale, Mary J Kwasny, David C Mohr

**Affiliations:** 1 Department of Psychiatry and Behavioral Sciences Feinberg School of Medicine Northwestern University Chicago, IL United States; 2 HealthPartners Institute Minneapolis, MN United States; 3 Department of Psychiatry Case Western Reserve University University Hospitals Cleveland Medical Center Cleveland, OH United States; 4 Carolina Outreach Durham, NC United States; 5 Vibrent Health Fairfax, VA United States; 6 Department of Preventive Medicine Feinberg School of Medicine Northwestern University Chicago, IL United States

**Keywords:** bipolar disorder, self-management, mHealth, eHealth, smartphone, mobile phone, mental health, mobile health

## Abstract

**Background:**

Bipolar disorder is a severe mental illness with high morbidity and mortality rates. Even with pharmacological treatment, frequent recurrence of episodes, long episode durations, and persistent interepisode symptoms are common and disruptive. Combining psychotherapy with pharmacotherapy improves outcomes; however, many individuals with bipolar disorder do not receive psychotherapy. Mental health technologies can increase access to self-management strategies derived from empirically supported bipolar disorder psychotherapies while also enhancing treatment by delivering real-time assessments, personalized feedback, and provider alerts. In addition, mental health technologies provide a platform for self-report, app use, and behavioral data collection to advance understanding of the longitudinal course of bipolar disorder, which can then be used to support ongoing improvement of treatment.

**Objective:**

A description of the theoretical and empirically supported framework, design, and protocol for a randomized controlled trial (RCT) of LiveWell, a smartphone-based self-management intervention for individuals with bipolar disorder, is provided to facilitate the ability to replicate, improve, implement, and disseminate effective interventions for bipolar disorder. The goal of the trial is to determine the effectiveness of *LiveWell* for reducing relapse risk and symptom burden as well as improving quality of life (QOL) while simultaneously clarifying behavioral targets involved in staying well and better characterizing the course of bipolar disorder and treatment response.

**Methods:**

The study is a single-blind RCT (n=205; 2:3 ratio of usual care vs usual care plus LiveWell). The primary outcome is the time to relapse. Secondary outcomes are percentage time symptomatic, symptom severity, and QOL. Longitudinal changes in target behaviors proposed to mediate the primary and secondary outcomes will also be determined, and their relationships with the outcomes will be assessed. A database of clinical status, symptom severity, real-time self-report, behavioral sensor, app use, and personalized content will be created to better predict treatment response and relapse risk.

**Results:**

Recruitment and screening began in March 2017 and ended in April 2019. Follow-up ended in April 2020. The results of this study are expected to be published in 2022.

**Conclusions:**

This study will examine whether LiveWell reduces relapse risk and symptom burden and improves QOL for individuals with bipolar disorder by increasing access to empirically supported self-management strategies. The role of selected target behaviors (medication adherence, sleep duration, routine, and management of signs and symptoms) in these outcomes will also be examined. Simultaneously, a database will be created to initiate the development of algorithms to personalize and improve treatment for bipolar disorder. In addition, we hope that this description of the theoretical and empirically supported framework, intervention design, and study protocol for the RCT of LiveWell will facilitate the ability to replicate, improve, implement, and disseminate effective interventions for bipolar and other mental health disorders.

**Trial Registration:**

ClinicalTrials.gov NCT03088462; https://www.clinicaltrials.gov/ct2/show/NCT03088462

**International Registered Report Identifier (IRRID):**

DERR1-10.2196/30710

## Introduction

Bipolar disorder is a severe mental illness characterized by episodes of mania, hypomania, depression, and mixed states [1,2]. It causes significant impairment in psychosocial functioning and is a leading cause of disability [2,3]. In addition, bipolar disorder doubles all-cause mortality and is associated with a high lifetime risk of suicide [4]. The ongoing suffering produced by this disorder drives a clear need for continuing efforts to develop and increase access to effective treatment.

Pharmacotherapy is the primary treatment for bipolar disorder, but even when pharmacological treatment is initially effective, high rates of episode recurrence, interepisode symptoms, and psychosocial impairment persist [5-9]. Evidence from randomized controlled trials (RCTs) indicates that combining psychotherapy with pharmacotherapy decreases episode recurrence and symptom burden while also improving quality of life (QOL) [10-18]. Treatment guidelines for bipolar disorder recommend providing adjunctive psychotherapy [19-21]. Despite these recommendations and the demonstrated effectiveness of adjunctive psychotherapy, multiple barriers limit access to evidence-based therapy, and only about half of individuals with bipolar disorder receive psychotherapy [22-26].

Smartphones are widely used and accepted for mental health assistance [27-31]. Smartphone-based mental health technologies thus provide a promising means for increasing access to the content of empirically supported psychotherapies for bipolar disorder. In addition, individuals with bipolar disorder in sustained remission report using self-management strategies that overlap significantly with the content of empirically supported psychotherapies, and many people with bipolar disorder are interested in using self-management strategies to stay well [32-35]. These findings suggest that mental health technologies delivering self-management strategies derived from empirically supported psychotherapies may meet user needs and support engagement [36-39]. In addition, mental health technologies provide novel opportunities for improving intervention impact, such as the provision of real-time assessments and adaptive feedback to users as well as status alerts to their mental health providers [40-43]. Furthermore, the use of smartphones allows collection of self-report, app use, and behavioral data that may enhance prediction of longitudinal course and current relapse risk, improve evaluation of treatment response, and provide a better understanding of behavior change processes to facilitate timely and successful intervention delivery [44-46].

Unfortunately, most publicly available smartphone apps for bipolar disorder do not provide information and self-management tools that reflect current practice guidelines [47,48]. However, work is underway to develop and test web- and smartphone-based interventions for bipolar disorder based on empirically supported psychotherapies. These studies consistently demonstrate that individuals use and report high levels of satisfaction with these apps but are less consistent in showing improvement in symptoms and QOL [49-55]. LiveWell, a novel smartphone-based self-management intervention for bipolar disorder, has been developed (NCT02405117) and tested in a single-blind RCT (NCT03088462). As adequate description of interventions is essential to facilitate ongoing efforts to improve and disseminate empirically supported treatments [56-59], the theoretical and empirically supported framework, design, content, mode, and timing of delivery, as well as the evaluation methodology for LiveWell, is described here.

## Methods

### Overview

The development of LiveWell and its evaluation followed an intervention mapping and person-centered approach [60-62]. This approach used an iterative strategy combining multiple stages of framework and design revisions based on feedback from mental health providers and individuals with bipolar disorder. This intervention development process has previously been described in detail [63-66]. In terms of clinical and recovery needs, the goal of LiveWell is to increase access to empirically supported treatment strategies for bipolar disorder, address the self-management interests of individuals with bipolar disorder, and enhance the utility of these strategies by providing real-time assessment feedback and provider alerts. In terms of research needs, the goal of LiveWell is to provide a self-report, app use, and behavioral data collection platform to enhance prediction of the longitudinal course and current relapse risk, improve evaluation of treatment response, and provide a better understanding of behavior change processes to facilitate timely and successful intervention delivery. The details of the intervention framework, its practical design, and evaluation methodology are described below.

### Intervention Framework

**Figure 1:**
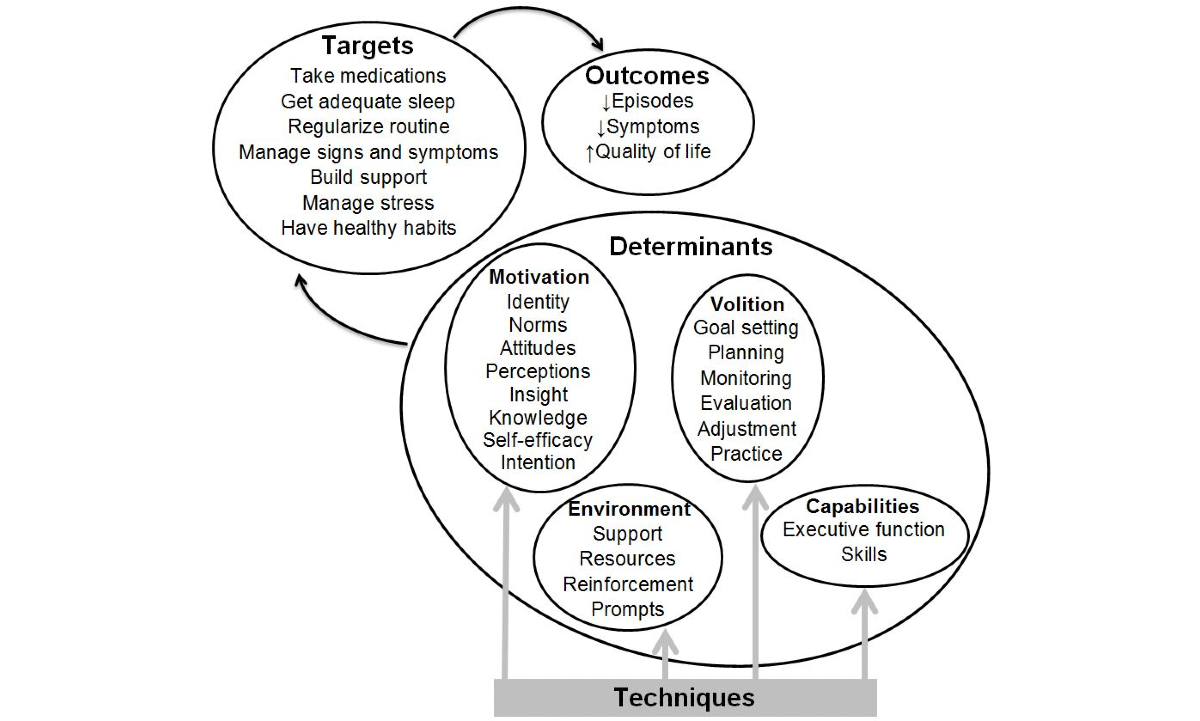
Behavior change framework.

A behavior change framework for use in guiding the creation of content and tools for LiveWell was developed by integrating user feedback with information from empirically supported psychotherapies for bipolar disorder [10-18], health psychology behavior change theories [62,67-85], and chronic disease self-management models [86-92]. The framework proposes that (1) engaging in target behaviors improves clinical and recovery outcomes, (2) behavioral determinants govern the enactment of target behaviors, and (3) exposure to behavior change technique content and tool use alters behavioral determinants (Figure 1 and Table 1). This framework provides a theory-based and empirically supported rationale for including intervention content and tools. It also provides a means to label intervention content in terms of outcomes, targets, and determinants addressed by the behavior change techniques delivered (Tables 2 and 3; Multimedia Appendix 1). This labeling will allow investigation of intervention mechanisms by examining the relationships between changes in outcomes, targets, determinants, and exposure to behavior change technique content and tool use.

**Table 1:** Behavior change framework determinant definitions and theories.

Domains and determinants			Definitions		Theories
**Motivation**					
	Identity	Self-perception of personal characteristics, social roles, and types that form a set of standards guiding behavior		IT^a^, TDF^b^, TPB^c^	
	Norms	Beliefs about whether others would approve or disapprove of a behavior (injunctive) and desire to comply with others (compliance). Beliefs about whether others engage in a behavior (descriptive) and desire to be like others (identification)		FT^d^, IT, TPB	
	Attitudes	Beliefs about the tangible costs and benefits (instrumental) or emotional consequences (affective) of engaging in a behavior		HAPA^e^, MI^f^, SCT^g^, TPB	
	Perceptions	Beliefs about one’s susceptibility to a health condition and the severity of the health condition (risk susceptibility and severity)		HAPA, HBM^h^, CDSM^i^	
	Insight	Awareness of having a health condition, presence of symptoms and consequences, and need for treatment		CDSM	
	Knowledge	Awareness of information necessary to support active participation in management of a health condition		CDSM, COMB^j^, HBM, SCT	
	Self-efficacy	Beliefs about personal ability to perform a target behavior		GST^k^, HAPA, SCT, TPB, TDF	
	Intention	Explicit decision to engage in a target behavior to achieve an outcome		GST, HAPA, SCT, TPB, TDF	
**Volition**					
	Goal setting	Identification of a target behavior to engage in to achieve an outcome		CT^l^, GST, SCT, SDT^m^	
	Planning	Specific plans for engaging in a target behavior (task) or overcoming obstacles to engaging in a target behavior (coping)		CT, CDSM, HAPA	
	Monitoring	Maintaining awareness of engagement in a target behavior		CT, CDSM, HAPA	
	Evaluation	Detecting degree of alignment between actual behavior and target behavior.		CT, CDSM, HAPA	
	Adjustment	On the basis of monitoring and evaluation: acknowledge success and maintain or refocus current goals and plans or understand problems, identify solutions and make changes in current goals and plans		CT, CDSM, HAPA	
	Practice	Repetition of an action or its elements to learn or improve a capability		CDSM, SCT	
**Environment**					
	Support and obstruction	Direct informational, emotional, or tangible physical input from others that facilitates or hinders engagement in a behavior		CDSM, COMB, HAPA, SCT, TDF	
	Resources and constraints	Physical conditions of a situation that facilitate or hinder engagement in a behavior		CDSM, COMB, HAPA, SCT, TDF	
	Reinforcement	Increasing the probability of a behavior by arranging a contingency between the behavior and a consequence that follows the behavior		CT, TDF	
	Prompts	Physical or social stimulus that acts as a reminder to engage in a behavior		CT, TDF	
**Capabilities**					
	Executive function	Cognitive capacities such as working memory, inhibitory control, and mental flexibility		COMB, SCT, TDF	
	Skills	Abilities acquired or developed through practice		COMB, SCT, TDF	

^a^IT: Identity Theory [80,85].

^b^TDF: Theoretical Domain Framework [81,84].

^c^TPB: Theory of Planned Behavior [69].

^d^FT: Focus Theory [93].

^e^HAPA: Health Action Process Approach [76,77].

^f^MI: Motivational Interviewing [74].

^g^SCT: Social Cognitive Theory [68,73].

^h^HBM: Health Belief Model [83].

^i^CDSM: Chronic Disease Self-Management [86-92].

^j^COMB: Capability Opportunity Motivation Behavior [79].

^k^GST: Goal Setting Theory [72,75].

^l^CT: Control Theory [67,71].

^m^SDT: Self-Determination Theory [70,78].

**Table 2:** Smartphone app content.

Domains, determinants, and techniques				PP^a^
**Motivation**				40.1
	**Knowledge**			16.1
		Information on app use	5.1	
		Information about a health condition	4.2	
		Information about treatment of a health condition	4.1	
		Information about effective self-regulation	1.4	
		Information about antecedents	1.3	
	**Attitudes and perceptions**			14.1
		Information about health consequences	9.6	
		Pros and cons	2.0	
		Information about social and environmental consequences	1.3	
		Information about emotional consequences	0.7	
	**Norms**			4.8
		Social comparison	2.5	
		Credible source	1.9	
	**Insight**			2.4
		Guided discovery	2.4	
	**Self-efficacy**			1.5
		Focus on past success	1.0	
		Persuasion about capability	0.5	
	**Identity**			0.8
		Valued self-identity	0.7	
	**Intention**			0.5
		Elicit commitment	0.5	
**Volition**				35.8
	**Evaluation**			11.4
		Feedback on outcome of behavior	7.9	
		Feedback on behavior	2.5	
		Discrepancy between current behavior and goal	1.0	
	**Planning**			8.3
		Coping planning	3.5	
		Task planning	2.6	
		Implementation intentions	2.3	
	**Monitoring**			5.9
		Self-monitoring of outcomes of behavior	4.0	
		Self-monitoring of behavior	1.9	
	**Adjustment**			5.2
		Review behavior goals	5.1	
	**Practice**			4.1
		Behavioral rehearsal	2.9	
		Graded tasks	1.1	
	**Goal setting**			0.9
		Process goal	0.7	
**Environment**				13.0
	**Support and obstruction**			11.0
		Social support—feedback	4.1	
		Social support—treatment	3.7	
		Restructuring social environment	1.5	
		Social support—unspecified	0.7	
		Social support—support group	0.5	
	**Prompts**			1.0
		Introduce cues	1.0	
	**Resources and constraints**			0.8
		Restructuring physical environment	0.6	
**Capabilities**				10.1
	**Skills**			10.0
		Instruction on how to perform a behavior	3.2	
		Relaxation training	1.3	
		Engage in activity	1.2	
		Reduce negative emotions (stress management)	1.1	
		Behavioral experiments	0.8	
		Observing	0.6	
		Framing and reframing	0.5	
		Conserving mental resources	0.4	
		Accepting	0.4	
		Behavioral substitution	0.4	

^a^Percent of smartphone app pages.

**Table 3:** Coaching scripts content.

Domain, determinant, and behavior change technique				PP^a^
**Motivation**				49.5
	**Knowledge**			12.1
		Information about app use	7.7	
		Information about a health condition	4.4	
	**Self-efficacy**			11.0
		Emphasize autonomy	7.4	
		Affirmation	3.6	
	**Intention**			10.4
		Agenda mapping	8.7	
		Summarize the plan	1.4	
		Elicit commitment	0.3	
	**Attitudes and perceptions**			9.4
		Desire-ability-reason-need questions	4.8	
		Elicit-provide-elicit	2.0	
		Information about health consequences	1.9	
		Monitoring of emotional consequences	0.4	
		Information about social and environmental consequences	0.3	
	**Insight**			4.5
		Guided discovery	4.5	
	**Norms**			1.6
		Social comparison	1.1	
		Credible source	0.5	
	**Identity**			0.4
		Valued self-identity	0.4	
**Volition**				27.9
	**Planning**			14.6
		Coping planning	7.2	
		Task planning	3.7	
		Consider change options	2.0	
		Brainstorming	1.5	
		Implementation intentions	0.3	
	**Adjustment**			5.0
		Review behavior goal	4.7	
		Review outcome goal	0.3	
	**Goal setting**			3.6
		Process goal	2.7	
		Outcome goal	0.9	
	**Evaluation**			2.7
		Feedback on behavior	2.4	
		Discrepancy between current behavior and goal	0.3	
	**Monitoring**			2.1
		Self-monitoring of behavior	1.8	
		Self-monitoring of outcome of behavior	0.3	
**Environment**				22.0
	**Support and obstruction**			18.8
		Open-ended questions	5.9	
		Social support—practical	3.5	
		Permission to provide information and advice	3.5	
		Social support—unspecified	3.0	
		Support change and persistence	2.3	
		Reflective statements	0.3	
		Summary statements	0.3	
	**Reinforcement**			1.8
		Social reward	1.8	
	**Prompts**			1.4
		Introduce cues	1.4	
**Capabilities**				0.5
	**Skills**			0.5
		Conserving mental resources	0.5	

^a^Percent of coaching script pages.

Adjunctive psychotherapy interventions for bipolar disorder typically enroll individuals who are between acute episodes and focus on the prevention of relapse [10-15,18]. Time to episode recurrence was thus selected as the primary clinical outcome (Figure 1) to ensure that the efficacy of LiveWell can be assessed in the context of existing face-to-face studies. As cumulative measures of symptom burden are better predictors of psychosocial functioning than episode recurrence rate [94], percentage time symptomatic and symptom severity were selected as secondary outcomes. QOL was also chosen as a secondary outcome because the absence of symptoms is not synonymous with QOL, and improvement in QOL is highly valued by individuals with bipolar disorder [39,95,96].

Although empirically supported adjunctive therapies for bipolar disorder use diverse approaches, changes in shared behavioral targets may result in the improved outcomes produced by these therapies [5,18,26,33,97,98]. Thus, the LiveWell intervention seeks to improve clinical and recovery outcomes by assisting individuals with managing targets proposed to underlie the impact of existing face-to-face therapies (Figure 1). LiveWell emphasizes the importance of identifying signs and symptoms of relapse, developing plans and monitoring for relapse, and enacting and adjusting plans as needed (managing signs and symptoms) [99,100]. In addition, LiveWell uses a similar process to support taking medications as prescribed [101-105], obtaining adequate sleep duration [106-110], and maintaining regular routines [111-115]. LiveWell also addresses strengthening social support [5,35], managing stressors [33,35], and engaging in healthy habits regarding diet, exercise, and substance use [5,116-118].

**Figure 2:**
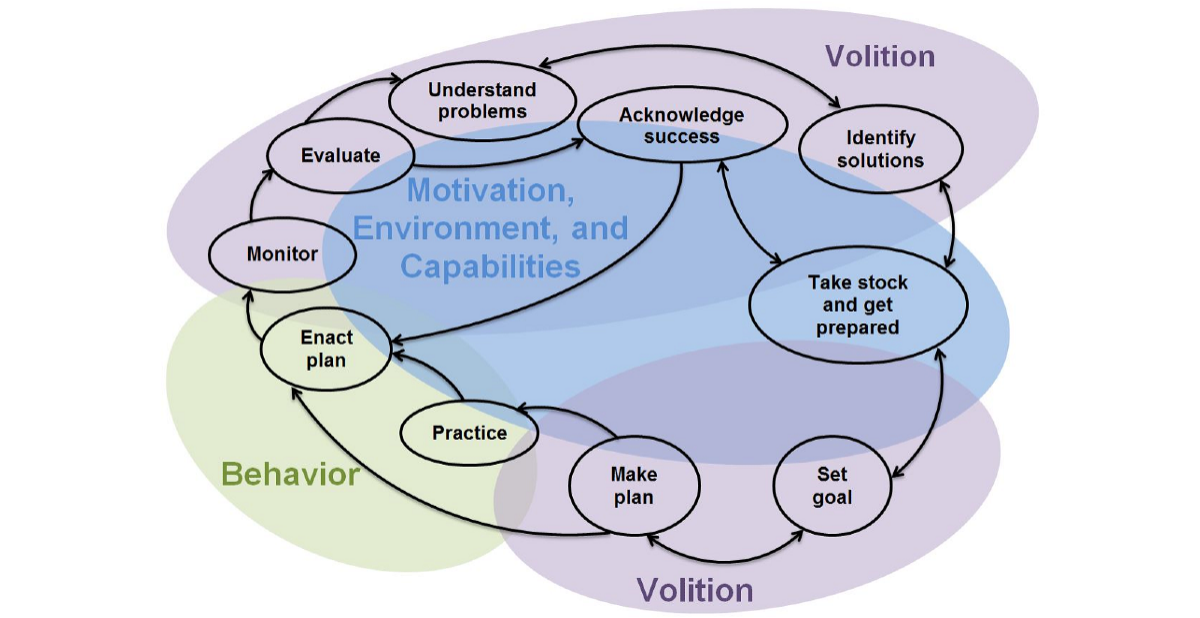
Behavior change process.

Recent studies have proposed that behavioral determinants govern the enactment of target behaviors and that psychosocial interventions produce changes in these determinants via the delivery of behavior change techniques [62,81,82,84,119-124]. Behavior change techniques are replicable and irreducible intervention components that impact behavioral regulation [82]. Taxonomies defining distinct techniques and grouping them into nonoverlapping clusters hypothesized to alter specific behavioral determinants have been developed [82,119-124]. Distinct behavior change techniques can thus be selected and delivered to shift a particular determinant involved in enacting a target behavior (Tables 2 and 3; Multimedia Appendix 1). To align with existing behavior change theories and organize determinants [62,67-85], we grouped determinants and their corresponding techniques into four domains: motivational determinants involved in developing an intention to engage in a behavior, volitional determinants involved in enacting the behavior, environmental determinants and capabilities that impact motivation and volition (Figure 1 and Table 1). Although our behavior change framework is presented as a linear system in which delivery of behavior change techniques alters behavioral determinants to shift target behaviors and improve outcomes (Figure 1), this linear view should be regarded as a simplification. Instead, the behavior change framework should be considered as a continuous and reciprocal system in which multiple wellness outcomes, target behaviors, and behavioral determinants interact continuously and reciprocally to impact the process of health behavior change [87,125]. In terms of providing information about self-management strategies to users, the behavior change framework is therefore recast as an ongoing process that requires assessment of motivation, environment, and capabilities to guide the selection of target behaviors and creation of plans followed by behavioral enactment and practice accompanied by ongoing reassessment and updating based on monitoring (Figure 2).

### Intervention Design

**Figure 3:**
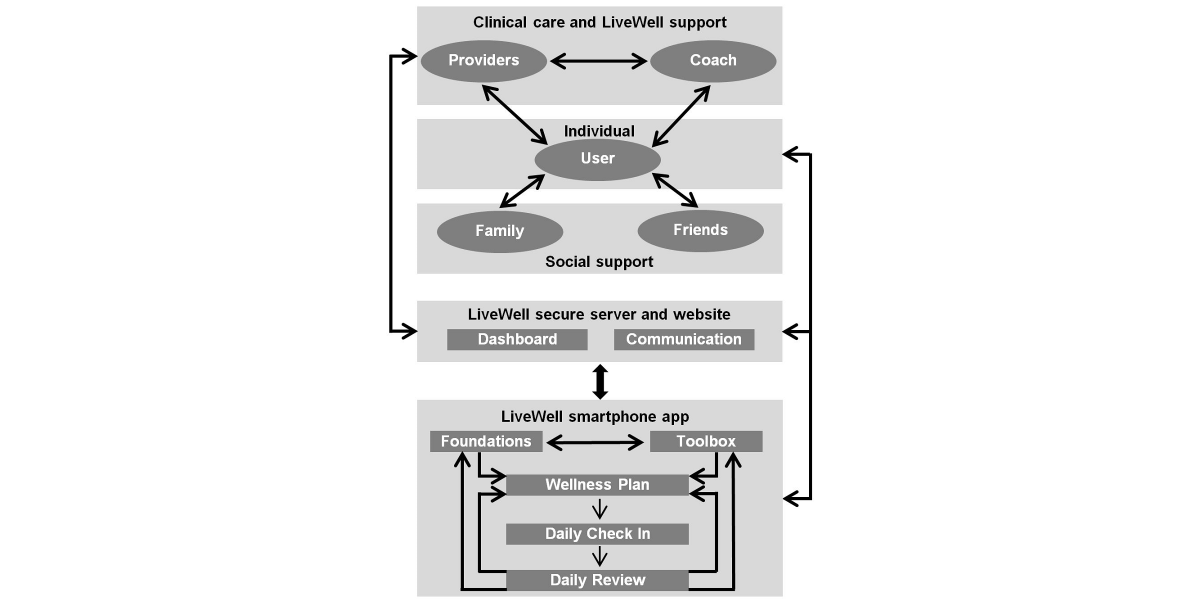
Intervention design: arrows outside gray boxes on left side indicate provider and coach access to dashboard and email communications and on right side user access to the dashboard and smartphone app. Arrows between providers, coach, user, family and friends represent interactions between the user and supports. In the case of the coach, interactions with the user and provider may be prompted by email alerts. Arrows within the app represent the user app workflow. Information in the Foundation lessons and Toolbox is used to develop a personalized Wellness Plan, including daily monitoring using the Daily Check-In. Daily Check-In data are used to provide feedback via the Daily Review. The Daily Review feedback directs the user to relevant app content in their Wellness Plan or the Foundations and Toolbox.

Although the behavior change framework (Figures 1 and 2; Tables 1-3) directs the selection of theory-based and empirically supported strategies for inclusion in LiveWell, it does not address the practical methods for using technology to deliver content and supporting tools [126-129]. Our intervention design thus considers the technical components involved in delivering self-management strategies. In addition, as human support is often a critical feature of effective mental health technology interventions, the design of coaching roles has been addressed [130-136]. As such, the LiveWell intervention has technological and human support components, including a smartphone app, a secure server and a website, and coaching support (Figure 3).

**Figure 4:**
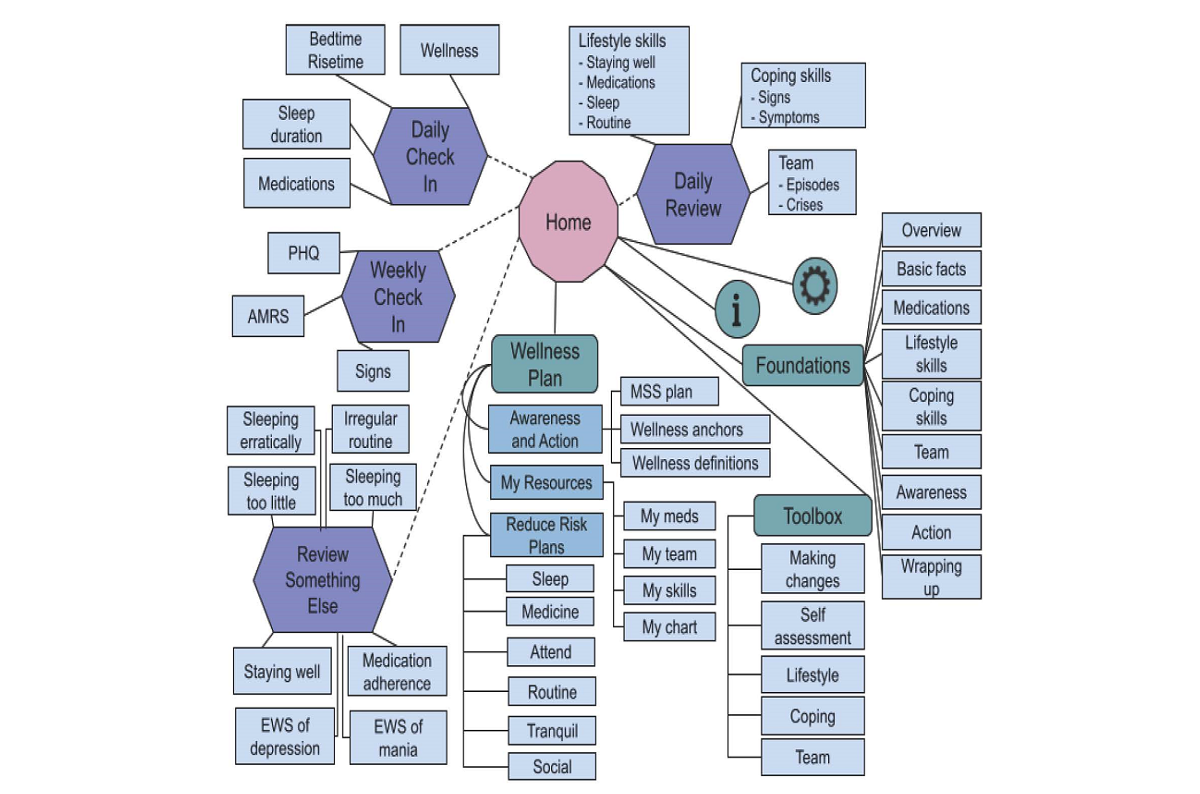
Smartphone App Design: Dashed lines indicate app components available based on timing (Daily and Weekly Check-Ins) or completion of other components (Daily Review, Review Something Else). EWS: early warning signs; MSS: manage signs and symptoms; PHQ: Patient Health Questionnaire 8; AMRS: Altman Mania Rating Scale; i: Instructions; gear symbol: settings.

The smartphone app has five main components: Foundations, Toolbox, Wellness Plan, Daily Check-In, and Daily Review (Figures 3 and 4; Multimedia Appendix 2). The Foundations and Toolbox components discuss the rationale for engaging in target behaviors, using self-management techniques, and the role of beliefs, environmental resources, and social support on behavioral engagement (Multimedia Appendix 2). The Foundations and Toolbox also discuss the importance of setting clear and realistic target goals, making detailed plans for accomplishing goals and overcoming obstacles, monitoring target behaviors, evaluating if goals are being met, and making adjustments as needed. In addition, the roles of self-assessment and learning and practicing skills for achieving target goals are discussed. Over 4 weeks, users work through the Foundations and Toolbox components and develop a personalized Wellness Plan that addresses lifestyle skills for reducing risk, coping skills for managing signs and symptoms, and resources for staying well (Figures 3 and 4). As part of the Wellness Plan, users develop a personalized plan for managing signs and symptoms (Awareness and Action) across a range of wellness levels (0 balanced, −1 or +1 daily hassles or uplifts, −2 or +2 prodromal or residual symptoms, −3 or +3 episode, −4 or +4 crisis). Creating this plan includes reviewing past experiences to identify personalized wellness scale anchors. This anchoring process assists users in monitoring and recognizing their current wellness level [35]. The plans also specify personalized actions for each wellness level [65]. In addition, users develop a personalized Reduce Risk plan. The Reduce Risk plan involves setting achievable goals, anticipating obstacles, and specifying clear actions to take for target behaviors, including taking medications as prescribed, obtaining adequate sleep, maintaining regular routines, strengthening and using social support, managing stressors, and engaging in healthy habits regarding diet, exercise, and substance use [65]. This Reduce Risk plan is described by the acronym SMARTS: Sleep, Medicine, Attend (to diet, exercise, and substance use), Routine, Tranquil, Social.

**Figure 5:**
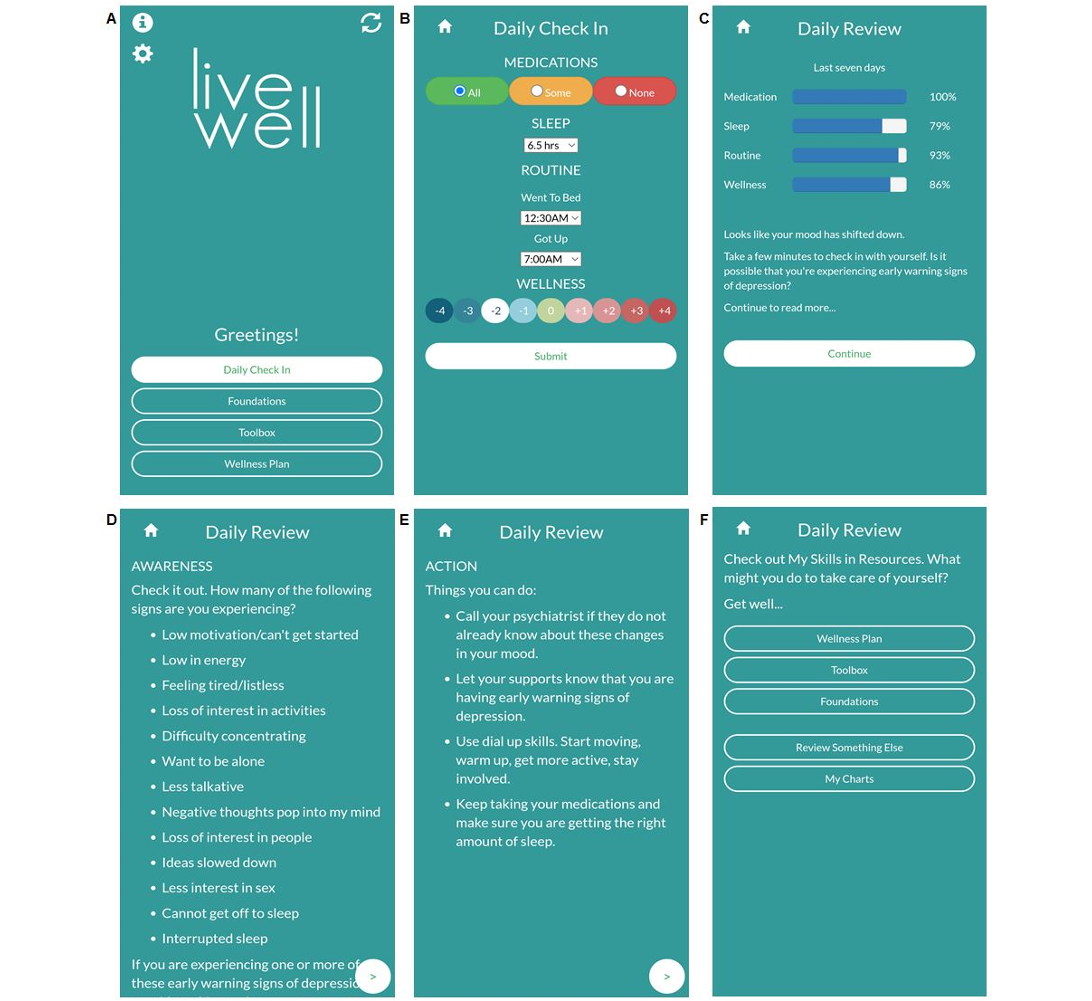
Daily Check-In and Daily Review: (A) Upon opening the LiveWell app, user views the home page with Daily Check-In highlighted indicating task to be completed, (B) User completes Daily Check-In with a wellness rating of -2 indicating possible early warning signs of depression, (C) After user submits Daily Check-In data, Daily Review feedback page displays summary of last 7 check-ins. Expert system identifies a possible shift in mood down as priority, (D-E) User continues through Daily Review and receives information about Awareness and Action, F. Last page of Daily Review suggests user check My Skills in Resources in the Wellness Plan. Daily Review feedback truncated here for display.

Monitoring is a major determinant of behavior change [58], an essential strategy for empirically supported bipolar disorder psychotherapies [11,18,97,99], and individuals with bipolar disorder are interested in using self-monitoring tools [32,33]. Thus, the core of the app is a Daily Check-In (Figure 3). The Daily Check-In monitors medication adherence, sleep duration, routine (bedtime and risetime), and wellness level. These targets were selected for daily monitoring because they are consistently addressed in the core content of adjunctive psychotherapy interventions [5,18,26,33,97,98,137] and are readily amenable to goal setting and self-monitoring. Users were asked to check-in daily for 16 weeks. On the basis of data from the Daily Check-In, the Daily Review uses an expert system to provide adaptive, personalized real-time feedback [64]. As described in detail elsewhere [64], the rules linking delivery of feedback and the Daily Check-In data were developed based on existing literature regarding bipolar disorder and psychiatrist feedback from a web-based survey. This feedback reinforces success and directs users to relevant app sections (within Foundations, Toolbox, and Wellness Plan) to assist users with making adjustments if needed (Figures 3 and 4). If the Daily Check-In data indicate a need for additional clinical support based on the expert system algorithms [64], users receive feedback to contact their psychiatrist, and a message with a link to the psychiatrist’s phone number appears. An example of opening the app and completing a Daily Check-In followed by a truncated example of Daily Review feedback is displayed in Figure 5, and a more detailed use case scenario is provided in Multimedia Appendix 3.

Users also complete a Weekly Check-In, including the 8-question Patient Health Questionnaire (PHQ-8) [138], Altman Self-Rating Mania Scale (ASRM) [139], and checklists for early warning signs of depression and mania [140-142]. Users receive feedback and a pop-up message if their Weekly Check-In responses indicate the new onset of an episode. For example, if the PHQ-8 score transitions from <10 to ≥10, suggesting the onset of a depressive episode based on the published threshold [138], the pop-up message says, “Looks like you may be entering a depressive episode. Call your psychiatrist to check-in.” and contains a link to the psychiatrist’s phone number to prompt a call [64]. Overall, the app content encourages users to work with a psychiatrist to come to a mutual understanding of clinical problems and treatment plans and engage in active and sustained collaborative treatment and progress monitoring [63]. The secure server and website aim to support communication with providers by delivering automated email alerts to enrolled providers when additional clinical support may be needed.

**Figure 6:**
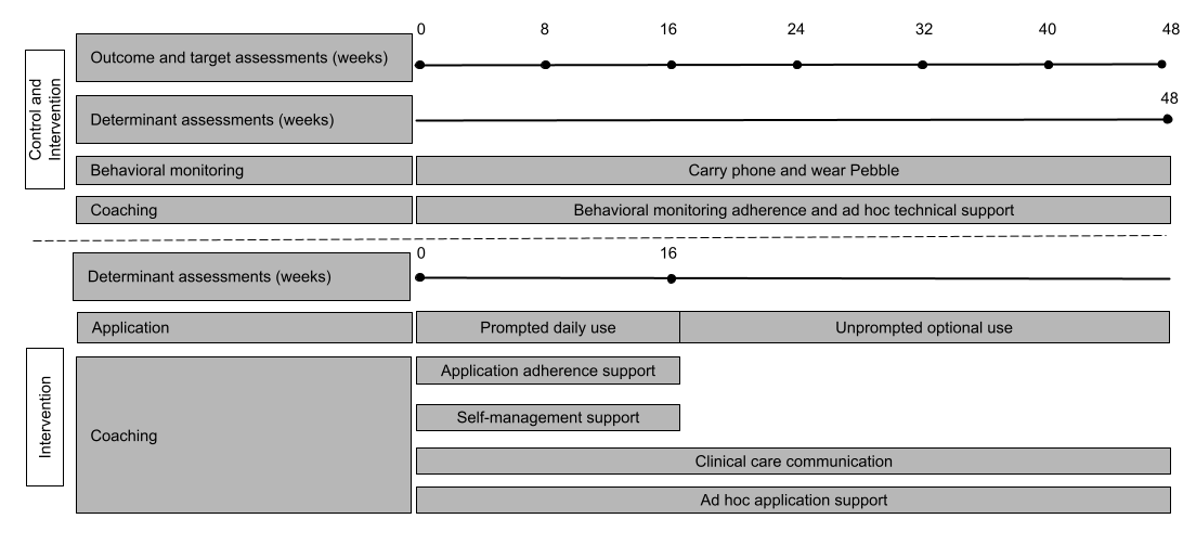
Study timeline.

The LiveWell technology was supported by coaches with bachelor’s degrees who did not have professional mental health training. The coaches were trained and supervised for their roles. Details of the coach training and supervision are presented elsewhere [63]. The coach supports app use adherence, self-management, and clinical care communication (Figure 6). The coach uses a supportive accountability model to facilitate app use by working with the user to establish a bond, legitimacy, and accountability [63,135]. The coach provides self-management support using a simplified adaptation of motivational interviewing, which is effective with brief consultations administered by individuals without professional mental health training [63]. The coach also uses a chronic disease self-management model to provide app content guidance that assists the user by setting appropriate target goals, personalizing a Wellness Plan, monitoring target progress, and enacting and adjusting plans based on success or problems [63,86-92,125]. There is a clear division of labor between the technology and the coach to ensure the coach operates within the scope of nonclinical practice. The technology operates as a psychotherapeutic strategy expert and provides status summaries and alerts to the coach, who uses flow sheets and structured scripts to serve as a technology use concierge [63]. In addition, the coach works to support communication and collaboration with the care provider. The coach is prompted via server email alerts to contact providers via telephone when user self-assessments indicate problems with treatment adherence or the presence of early warning signs, worsening, or severe symptoms.

Coaching starts with a structured face-to-face meeting that addresses how using self-management strategies within the app can assist users in managing their wellness (Multimedia Appendix 4). The coach works with the user to review their experiences with normal ups and downs, early warning signs and symptoms, episodes, and related crises. This review leads to developing a personalized 9-point wellness rating scale to facilitate the self-monitoring of signs and symptoms. Next, the coach walks the user through the app and has the user complete a Daily Check-In and Daily Review. The user then sets specific goals for medication adherence, sleep duration, routine bedtime and risetime, and wellness rating range. The coach encourages the user to set parameters known to facilitate health, including maintaining medication adherence, adequate sleep duration, regular bed and wake windows, and “balanced” wellness ratings (expected ups and downs due to routine events) [5,18,26,33,97,98]. The coach wraps up the face-to-face meeting clarifying the coach’s role and obtaining a commitment to app use and target goal achievement.

Following the face-to-face meeting, 6 scheduled coaching calls occur during weeks 1-4, 6, and 16 (Figure 6). Before each call, the coach reviews a dashboard summarizing app use and the percentage of days that personalized target goals were met [64]. At each call, the coach uses the summary and a structured script to review progress and guide app use (Multimedia Appendix 4). To provide closure and a time-limited treatment, the sixth and final call wraps up working with the coach as well as the request to check-in daily [143]. The coach reviews with the user what they learned and what future plans may assist the user in living well. The user is encouraged to commit to using the strategies they have found helpful and return to app use as needed. The user is asked if they would like to continue to receive daily notifications on their smartphone as a reminder to check-in (Figure 6). The user is also asked to continue carrying their smartphone whenever they leave home and to wear a Pebble watch all day, every day, to allow ongoing behavioral monitoring for study purposes.

All coach face-to-face meetings and telephone calls were audiotaped. To assess coach fidelity, 15% of the completed coach face-to-face meetings and 15% of the weekly scheduled call audiotapes were randomly selected for review each month. Coach fidelity was then assessed using an adapted version (Multimedia Appendix 4) of the behavior change counseling index [144] scored by a trained mental health professional (psychologist). Exit questionnaires assessing smartphone app usability and coaching support were delivered at study week 48 through a web survey sent by the coaches to all participants in the intervention arm (Multimedia Appendix 5). In addition, exit interviews were completed by telephone after week 48 for the first 15% of participants exiting the intervention arm (Multimedia Appendix 5). These exit questionnaires and interviews were completed to obtain user feedback on the intervention to assist with the ongoing development of LiveWell [65].

### Intervention Evaluation

#### Overview

An RCT to evaluate LiveWell has been carried out, and data analysis is underway for the following: aim (1) to establish the capacity of LiveWell to reduce relapse and symptom burden and improve QOL in bipolar disorder, aim (2) to investigate the impact of LiveWell on proximal behavioral targets and the relationship between changes in these targets and changes in relapse rate and symptom burden, and aim (3) to identify novel behavioral signatures in individuals with bipolar disorder that predict treatment response and relapse. Aim 1 will test the following hypotheses: primary hypothesis—participants in the intervention group will experience (H1) a longer time to relapse relative to treatment as usual (TAU); secondary hypotheses—participants in the intervention group will experience (H2) a lower percentage of time being symptomatic, (H3) lower symptom severity, and (H4) a better QOL relative to TAU. Aim 2 will test the following additional hypotheses: primary hypothesis—participants in the intervention group will experience (H1) larger improvements in proximal behavioral targets (eg, medication adherence) relative to TAU; secondary hypotheses—variation in the proximal behavioral targets will account for substantial variance in the (H2) primary clinical outcome (time to relapse) and secondary clinical outcomes of (H3) percentage time symptomatic and (H4) symptom severity; proximal behavioral targets will mediate the intervention effect on the (H5) primary clinical outcome (time to relapse) and the secondary clinical outcomes of (H6) percentage time symptomatic and (H7) symptom severity; tertiary hypotheses—participants in the intervention group will experience (H8) an increase in performance determinant scores for each target, and at the final time point, will have (H9) higher performance determinant scores for each target relative to TAU. Aim 3 is exploratory and will develop a database of behavioral sensing (intervention and TAU arms) as well as app use and self-assessment (intervention arm only) data. The relationships between these data and clinical status assessment data will be examined with the long-term goal of better predicting current relapse risk, treatment response, and longitudinal course for individuals with bipolar disorder.

#### Entry Criteria

To facilitate recruitment of eligible participants and minimize exclusions while maximizing safety and study power, the following inclusion and exclusion criteria were used: The inclusion criteria were as follows: (1) adults aged 18 to 65 years, (2) individuals with bipolar disorder type I, and (3) a minimum of one acute episode in the last 2 years. Exclusion criteria were as follows: (1) not receiving psychiatric care, (2) current mood episode, (3) current severe suicidal ideation or a recent serious suicide attempt (last 3 months), (4) current substance use disorder (last 3 months), (5) visual impairments limiting mobile phone use, and (6) inability to speak and read English.

#### Ethics Approval

The study was reviewed and approved by the Northwestern University Institutional Review Board (STU00202860).

#### Recruitment, Screening, and Enrollment

The RCT for LiveWell recruited participants from the Chicago and Minneapolis-Saint Paul areas. At both sites, recruitment letters were sent to eligible individuals (bipolar disorder diagnosis, 18-65 years, and consent to contact for research) whose information was available in site-specific research registries or electronic health record data warehouses. The recruitment letters were followed up with phone calls from the study staff. Study recruitment information was also available for both sites at ClinicalTrials.gov, ResearchMatch.org, and WeSearchTogether.org websites. In the Chicago area, individuals were also recruited via mental health and university clinic presentations, flyers, brochures, e-mails to mental-health providers affiliated with Northwestern Medicine, and advertisements (Facebook, Reddit, Craigslist, Google AdWords, Chicago Transit Authority, digital, and print newspapers).

The study team contacted individuals via telephone (research registries) or individuals contacted the study team on the web, by email, or telephone. Individuals then completed a web based or telephone screening consent and completed a brief web-based or telephone-based eligibility screener (Multimedia Appendix 6). Eligible individuals were scheduled for telephone screening using a modified Mini International Neuropsychiatric Interview [145-147] and the National Institute on Drug Abuse Quick Screen followed by the Alcohol Use Disorders Identification Test and National Institute on Drug Abuse-modified assist for additional substance use disorders screening. A suicide attempt screener was also delivered, and demographic information was obtained. If still eligible, individuals attended a face-to-face clinic visit at which written consent was completed, followed by a structured interview with a trained mental health clinician (psychiatrist or psychologist) using an abbreviated and modified version of the Affective Disorders Evaluation and Clinical Monitoring Form [148-150]. Individuals with a confirmed diagnosis at the clinic visit were scheduled for a baseline telephone assessment and enrolled if no exclusion criteria were present at this assessment. Individuals who exhibited a current mood episode or substance use disorder, severe suicidality, or a recent serious suicide attempt at any step during screening were offered the opportunity to repeat the step at a later date and continue the screening process if the exclusion criteria were resolved (Multimedia Appendix 6).

At their initial face-to-face coaching meeting, intervention arm participants were asked if they wanted to allow any of their mental health providers to access a secure password-protected website that summarizes their self-report data (Daily and Weekly Check-Ins). If a participant consented to provider participation, a letter offering participation was mailed to the provider, and the coach contacted the provider by telephone. Providers interested in participating completed a web-based, verbal, or written consent form before receiving access to the website. Providers could opt to receive alerts via email or telephone when participant self-report data indicated reduced medication adherence, increased or decreased sleep duration, or deterioration in their daily wellness ratings. Providers were not required to opt for alerts to participate in the study. To allow access to the widest range of participants, participants were not required to allow any providers to access the website and providers were not required to consent to access the website or receive alerts for a participant to enroll in the study. However, as part of the written consent to participate in the study, all participants agreed to allow their psychiatrist to be contacted in the event of self-rated crisis situations, including daily wellness ratings of +4 or −4, new onset of depression (PHQ-8 score≥10), new onset of mania (ASRM score≥6), or other indications of emergent clinical problems or mental health deterioration.

#### Randomization

A biostatistician, blinded to screening and baseline assessment data, conducted computer-generated randomization on a 2:3 ratio (control:intervention) stratified based on clinical status (low risk—asymptomatic recovery; high risk—continued symptomatic, recovering, prodromal, and symptomatic recovery; Tables 4 and 5). Patients were randomized in permuted blocks of 5 at each site. The unbalanced design increases power to investigate effects within the intervention arm with minimal effect on the investigation of outcomes. Participants were stratified because time to relapse is likely to be significantly shorter for the high-risk participants who have not met the time or symptom number criteria for recovery from the last episode or who are recovered but have subsyndromal or prodromal symptoms [9,94,151,152]. Although many prior face-to-face psychotherapy studies restrict participant inclusion to those in asymptomatic recovery [10-15,18], this study includes additional high-risk individuals to increase access to the intervention.

**Table 4:** Clinical status when in an episode.

DSM 4 episode criteria	Episode entry criteria^a^ met?	Number of moderate symptoms of	Impairment	Consecutive days	PSR^b^
Mania	Yes	Mania≥3 if elevated Mania≥4 if only irritable	≥Moderate or hospitalized or psychosis	≥7 or hospitalized	5-6
Depression	Yes	Depression≥5	≥Moderate	≥10 out of 14	5-6
Mixed^c^	Yes, for both mania and depression	Criteria for both mania and depression	≥Moderate	≥7	5-6
Hypomania	Yes	Mania≥3 if elevated Mania≥4 if only irritable	< Moderate and not hospitalized and no psychosis	≥4	3

^a^Entry criteria: mania—moderate severity elevated, expansive, or irritable mood ≥ 7 consecutive days; depression—moderate severity depressed mood or loss of interest/pleasure ≥ 10 out of 14 consecutive days; hypomania—moderate severity elevated, expansive, or irritable mood ≥ 4 consecutive days and < 7 consecutive days. Clinical Monitoring Form symptom severity scale: none=0, mild=0.5, moderate=1, marked=1.5, and severe=2.

^b^PSR: Psychiatric Status Rating score.

^c^Mania with concurrent depression for 1 week. Count depressive symptoms for 5/7 consecutive days instead of 10/14.

**Table 5:** Clinical status when not in an episode.

Clinical Monitoring Form criteria	Recovered from last acute episode?	Symptom count^a^ and impairment	PSR^b^
Continued symptomatic	No	Symptom count>2 or ≥ moderate impairment	3-4
Prodromal	Yes	Symptom count>2 or new^c^ or ≥ moderate impairment	2-4
Recovering	No, recovering ≤8 consecutive weeks	Symptom count≤2 and < moderate impairment	1-2
Symptomatic recovery	Yes	Symptom count>0 and ≤2 and < moderate impairment	2
Asymptomatic recovery	Yes	Symptom count=0 and < moderate impairment	<2

^a^Symptom count: sum of symptom severity, if |severity| ≥1 round up, otherwise 0. Clinical Monitoring Form symptom severity scale: none=0, mild=0.5, moderate=1, marked=1.5, and severe=2.

^b^PSR: Psychiatric Status Rating score.

^c^Two new moderate, marked, or severe symptoms developed while in recovery.

#### Outcome Assessments

The primary outcome, time to relapse, will be measured as the number of weeks to episode onset (depression, mania, hypomania, or mixed) based on the Diagnostic and Statistical Manual of Mental Disorders fourth edition (DSM-4) criteria. The DSM-4 episode criteria were chosen to allow comparison with prior RCTs of face-to-face psychotherapy for bipolar disorder, as these studies primarily used the DSM-4 episode criteria [10-18]. To determine the number of weeks to episode onset, weekly clinical status ratings (Tables 4 and 5) were assessed by timeline follow-back rating of bipolar disorder symptom severity (Multimedia Appendix 7) using a modified Longitudinal Interval Follow-Up Evaluation (LIFE) and Clinical Monitoring Form (CMF) [148,149,153]. The secondary outcomes include the percentage of time symptomatic, symptom severity, and QOL. To determine percentage of time symptomatic, weekly psychiatric status ratings (Table 6) were assessed using the LIFE-CMF to determine the percentage of time symptomatic. A week will be scored as symptomatic if the psychiatric status rating is greater than one and a half (Table 6). Symptom severity was assessed using the Quick Inventory of Depressive Symptomatology (QIDS)–Clinician Rating and the Young Mania Rating Scale [154-157]. QOL was assessed using the World Health Organization Quality of Life BREF [158].

**Table 6:** Psychiatric status rating.

Score	Rating	Definition
6	Severe episode	Psychotic symptoms or severe impairment
5	Episode	No psychotic symptoms and no severe impairment
4	Marked symptoms	Symptom count^a^ >2 and marked or severe impairment
3	Moderate symptoms	Symptom count >2 or moderate impairment
2	Residual or prodromal symptoms	Symptom count >0 and ≤2. No moderate, marked, or severe impairment
1.5	Mild symptoms	≥One mild symptom. No moderate, marked, or severe symptoms (Symptom count=0). No moderate, marked, or severe impairment
1	No symptoms	No mild, moderate, marked, or severe symptoms. No impairment

^a^Symptom count: Sum of symptom severity, if |severity| ≥1 round up, otherwise 0. Clinical Monitoring Form symptom severity scale: none=0, mild=0.5, moderate=1, marked=1.5, and severe=2.

#### Target Assessments

The targets selected for assessment were taking medications as prescribed, obtaining adequate sleep duration, maintaining regular routines, and managing signs and symptoms as these targets are readily amenable to monitoring using the Daily Check-In, which is a core feature of LiveWell. Medication adherence was assessed using the Tablet Routine Questionnaire [159-161] and focused on percentage adherence with prescribed psychiatric medications, not including those prescribed as needed. Sleep duration, quality, and excessive sleepiness were assessed using the CMF, QIDS, Pittsburgh Sleep Quality Index, and an additional question to capture excessive sleepiness [162-164]. Sleep duration was primarily considered in this study. Sleep quality and excessive sleepiness will be explored secondarily because interepisode insomnia and hypersomnia are risk factors for relapse [107,163,165,166]. Routine was assessed using a 5-question trait version of the Social Rhythm Metric [167-170] and will be assessed primarily by a frequency score (Multimedia Appendix 7). Finally, the management of residual and prodromal signs and symptoms was assessed using the Symptom Management Scale. This scale was developed from the prodromes coping interview and inventory, which has been used in face-to-face cognitive behavioral therapy for bipolar disorder [99,137,142,171]. The outcome measure will be the total coping score (Multimedia Appendix 7).

#### Outcome and Target Assessment Delivery

Assessors blinded to the study arm conducted outcome and target assessments by telephone at baseline and then every 8 weeks until study exit at week 48 (Figure 6). Windows for complete assessments were up to 4 weeks after the assessment due date, calculated from the baseline assessment date. To minimize assessment burden and because QOL may vary less rapidly than other outcomes [172], the QOL assessment was delivered only at 0, 24, and 48 weeks. Assessments began by focusing on participants’ experiences over the prior 2 weeks. Mood and sleep were assessed using combined mood (CMF, QIDS, and Young Mania Rating Scale) and combined sleep (CMF, QIDS, and Pittsburgh Sleep Quality Index) instruments (Multimedia Appendix 7). The combined instruments streamlined the interviews and reduced the time required to complete the assessments. This was followed by assessment during the prior 2 weeks of routine (Social Rhythm Metric), medication adherence (Tablet Routine Questionnaire), and depending on the week QOL.

Information from the assessment of the prior 2 weeks was then used to anchor the timeline follow-back of weekly depressive and manic symptom severity (LIFE-CMF). In addition to assessing symptom severity, timeline follow-back using the LIFE assessed weekly variations in anxiety, suicidal ideation and attempts, psychotic symptoms, substance use, life events [173], mental health treatment attendance, and psychiatric medication adherence (Multimedia Appendix 7). The Symptom Management Scale was assessed last because it did not focus on experiences during the previous 2 weeks or since the last assessment. Instead, participants were asked to imagine experiencing ongoing or new low-level depressive or manic symptoms and how they might manage them (Multimedia Appendix 7). This approach was used because participant clinical status during the assessment interval may not have required management of prodromal or residual symptoms.

#### Determinant Assessments

To assess changes in the determinants proposed to govern the enactment of target behaviors, determinant questionnaires were developed for each assessed target (Multimedia Appendix 8). The determinant questionnaires are derived from the question stems of existing medication adherence questionnaires for schizophrenia and bipolar disorder [174,175] and health psychology questionnaires for assessing determinants of physical exercise and other health-related behaviors [76,77,176-183]. The questionnaires measure determinants proposed to underlie the pursuit of LiveWell’s four assessed behavioral targets (taking medications as prescribed, obtaining adequate sleep duration, maintaining regular routines, and managing signs and symptoms). The questionnaires were delivered via a web survey sent by the coaches to the participants. For the intervention arm, they were delivered at weeks 0, 16, and 48 (Figure 6). For participants in the control arm, they were only delivered at week 48 (Figure 6) because the delivery of the determinant questionnaires may serve as an active intervention [76,77,181]. Eventually, the determinant questionnaires may be incorporated into the smartphone app to provide real-time assessment of target change processes to facilitate improved adaptive real-time feedback.

#### Behavioral Sensing

Participants were offered the option of having the LiveWell app (intervention arm only) and an app for sensor data collection and secure transmission of data (Purple Robot, intervention, and control arms) [184] installed on their smartphone. Alternatively, participants had the option of receiving the apps on a study phone with an unlimited national calling and data plan. Individuals who opted to use their own phone were reimbursed for their plan up to the cost of the study phone plan or their own plan, whichever was less. All participants were provided with a wrist-worn actimeter (Pebble). Participants were asked to (1) use the phone with the apps as their only phone, (2) carry the phone with them whenever they left home, and (3) wear the watch 24 hours a day, 7 days a week except while it was charging.

#### Assessor Fidelity Assessments

Clinical outcomes and target assessments were performed by assessors with master’s degrees in mental health or counseling. Structured interviews can be validly and reliably administered by such personnel [185-189]. Assessors were trained by mental health professionals (a psychiatrist and psychologist) with instruction and practice on audiotaped ratings, followed by observing, role-playing, and observed assessments (≥3 each). All assessment telephone calls were audiotaped. Assessor fidelity focused on using the CMF for assessing status in the prior 2 weeks. Assessment audiotapes were randomly selected each month (15%) for review and scoring by a trained mental health professional (psychologist) to assess the fidelity of CMF scoring for symptom severity (percent match within 0.5), asymptomatic status (percent match), clinical status (percent match), and psychiatric status rating (percent match).

#### Analysis Overview

As the randomization was stratified by risk group, analyses will examine if an interaction between risk strata and the study arm is present. If no evidence of an interaction is evident, the models will be stratified or adjusted for risk strata. For time to relapse, missing data for participants who dropped out or were lost to follow-up will be censored at their last study visit. Percentage time symptomatic will be calculated based only on the observed time in the study. For longitudinal data (symptom severity, QOL, targets), multiple imputation using Markov Chain Monte Carlo methods will be used to address missing data by creating five unique data sets (SAS procedure PROC MI), and the results will be combined (SAS procedure PROC MIANALYZE) to obtain valid statistical inferences and least square means of the outcomes for each time point data will be calculated.

#### Aim 1: Primary and Secondary Outcomes Analysis

Time-to-relapse curves will be constructed using the Kaplan-Meier method. Log-rank tests will be used to determine if there is a univariate impact of the study arm while adjusting for risk strata. Cox proportional hazard models will then be fit to adjust for risk strata. Proportional hazards assumptions will be assessed, and the estimated hazard ratio and CIs will be presented. To compare percent time symptomatic, linear regression models will be used to determine if there was an interaction between risk strata and the study arm. If no evidence of interaction is seen, the main effect of arm will be reported and least square means will be estimated while adjusting for risk strata. Generalized linear mixed models for longitudinal data will be used for symptom severity and QOL measures. We will examine the interactive effects of the intervention on time. If no interaction is detected, we will test the independent effects of time as well as the treatment group.

#### Sample Size Considerations

Under an initial assumption of 200 participants enrolled and randomized at a 2:3 ratio with a control group relapse rate of 0.60, there would be 80% power to detect a hazard ratio of 0.61 assuming a loss to follow-up rate of 12%. The control relapse rate estimate was based on the reported percent relapse at 12 months for control (63%) seven psychosocial interventions for bipolar disorder [190-198]. This would equate to a reduction in relapse rate to 43% in the LiveWell intervention group. This additionally assumes 30 months of accrual, and 12 months follow-up using a log-rank test at a type I error rate of 5% (PASS 2008) [199]. For secondary outcomes, assuming 12% loss to follow-up, a 2-tailed *t* test would have 80% power to detect effect sizes of 0.43, for percent time symptomatic, which would equate to differences in mean time of 14% (45% symptomatic vs 69%, assuming an SD of 32%). While power calculations for generalized linear models do exist, they are quite dependent on assumptions that were speculative at the time of study design. Using multiple linear regression, a sample size of 200, adjusting for one covariate that explains 20% of the variability in outcome, we have 80% power to detect an increase in R-squared of 3%.

#### Aim 2: Target Analysis

Generalized linear mixed models will be used to examine the effects of time, risk strata, and study arm on longitudinal target and determinant data. We will use the framework of Muller et al [200] to identify the targets that mediate the effect of the intervention on time to relapse, percentage time symptomatic, and symptom severity. To compare the performance determinant scores for each target at the final time point, *t* tests with correction for multiple testing between the two groups will be used.

#### Aim 3: Behavioral Analysis

For both arms, behavioral sensor data will be collected including activity (Pebble watch and phone accelerometers), location (via the GPS), timing of incoming and outgoing texts, timing and duration of incoming and outgoing telephone calls, and for some phones, ambient light (lux), and sound (power and frequency). Feature variables will be summarized over 1-week windows, to identify behavioral features strongly correlated with depression and mania symptom severity. The relationship between behavioral features and clinical status will then be explored. In addition, for the intervention arm, self-report assessment and app use data as well as personalized wellness rating anchors and plans, reduce risk plans, sleep duration goals, and routine (bedtime and risetime) goals will be available. These data will be used to explore processes involved in staying well with the aim of improving adaptive delivery of content and tools to decrease relapse risk and symptom burden.

## Results

Recruitment and screening began in March 2017 and ended in April 2019. Follow-up ended in April 2020. The results of this study are expected to be published in 2022. Data from this study will be available at the National Institute of Mental Health Data Archive.

## Discussion

The primary goal of developing and delivering LiveWell is to increase access to self-management strategies derived from empirically supported bipolar disorder psychotherapies, thereby assisting individuals with bipolar disorder in staying well. It is hoped that this intervention will decrease relapse rates and symptom burden as well as improve QOL. LiveWell seeks to improve these outcomes by helping individuals manage target behaviors proposed to underlie the impact of existing interventions [5,18,26,33,97,98]. Although therapies for bipolar disorder often address the selected targets, there are limited data demonstrating that delivery of these therapies results in target behavior change or that changes in these behaviors mediate outcome changes [5,10-16,97,98,201]. Existing interventions for bipolar disorder have primarily been outcome studies so they have not focused on understanding relapse prevention and other outcome mechanisms. Relative to outcome studies, studies aimed at understanding the mechanisms of change require substantial modification, including measurement of outcomes and multiple proposed mechanisms before, during, and after intervention delivery [202-204]. As existing bipolar disorder interventions do not typically use this approach [5,10-16,97,98,201], our understanding of the mechanisms underlying the improved outcomes resulting from these interventions is limited. Because it is unclear what intervention components are useful for different individuals at different times, our ability to improve these interventions and adapt them for delivery in diverse settings is limited.

As a secondary goal, the LiveWell intervention aims to investigate the relationships between changes in target behaviors and outcomes over time to provide insights into the mechanisms of change. A behavior change framework provides a theory-based and empirically supported rationale for including intervention content and tools and facilitates the investigation of change mechanisms. It proposes that (1) engaging in target behaviors improves clinical and recovery outcomes, (2) behavioral determinants govern the enactment of target behaviors, and (3) exposure to behavior change technique content and tool use alters behavioral determinants. This framework provides a means to label app use and coaching call content in terms of outcomes, targets, and determinants addressed by the behavior change techniques delivered. This labeling will allow a more detailed exploratory investigation of intervention mechanisms by examining the relationships between changes in outcomes, targets, and determinants and exposure to behavior change technique content and tool use.

App use will measure outcomes via the Weekly Check-In (PHQ-8, ASRM) and target behaviors via the Daily Check-In (medication adherence, sleep duration, routine, and management of signs and symptoms) providing a more detailed examination of changes in outcomes and target behaviors over time than can be achieved via our standard telephone-based assessments delivered every 8 weeks. By labeling all pages of the app using the behavior change framework, it will also be possible to investigate how exposure to behavior change techniques relates to changes in determinants and how changes in determinants relate to changes in target behaviors. In addition, the intervention also uses smartphone and watch sensors to collect behavioral data such as activity levels and sleep duration (actimetry), location (GPS), and social interactions (texts and calls). As these behaviors may vary with clinical status, this passively collected behavioral data provides an opportunity to identify behavioral features correlated with clinical status and may improve our ability to determine what content to deliver to different individuals at different times to improve treatment. Overall the goal of the LiveWell intervention is to assist individuals in staying well while also serving as a platform for data collection that provides insights into treatment mechanisms and trajectories to allow iterative development and improvement of the intervention.

Despite the iterative design process used to develop LiveWell [63-66], the intervention design described here continues to have limitations. For instance, time to relapse was chosen as the primary outcome because most face-to-face interventions from which the LiveWell intervention derives focus on relapse prevention for individuals between mood episodes [10-15,18,205]. However, QOL and recovery outcomes (eg, connectedness, hope, and optimism) are highly valued by individuals with bipolar disorder but have not routinely been incorporated as outcomes in existing studies [39,95,96]. As a result, additional work will be needed to measure recovery outcomes, to identify the targets and determinants to address and the change techniques to deliver to facilitate changes in QOL and recovery outcomes. In addition, while the behavioral targets selected for daily monitoring (medication adherence, sleep duration, routine, managing symptoms and signs) are important and readily amenable to monitoring, other important targets have not been strongly emphasized. In particular, during the person-centered design process, building and using supports were strongly endorsed by users as being important for staying well [65,66]. Because of this feedback, coaching support for users was further developed [63]. Additional elements such as an opportunity for participants to engage in peer-to-peer discussion and exchange ideas could be incorporated, as these types of tools have been helpful in other interventions [206,207]. It will also be necessary to integrate assessments of users’ social support system strength and support use to assess the impact of the intervention on this target and the impact of changes in social support on outcomes.

Although extensive feedback was received during the development of LiveWell [63-66], most of the feedback was obtained from individuals with bipolar disorder and less so from providers. Thus, additional work will be required to obtain provider feedback during future efforts to implement and disseminate the LiveWell intervention. Furthermore, to better understand the impact of delivery and use of behavior change technique content and tools on changes in determinants and targets as well as interactions between multiple targets and determinants, a larger number of participants will be required. Hopefully, a structured behavior change framework will facilitate exploring these issues for bipolar disorder and other mental health disorders, thereby allowing data to be synthesized across studies to enhance the ability to improve mental health technologies and their delivery. Thus, we hope that the description of the theoretical and empirically supported framework, design, and protocol for the RCT of LiveWell will facilitate the replication, improvement, implementation, and dissemination of effective interventions for bipolar and other mental health disorders.

## Appendix

Multimedia Appendix 1 LiveWell app and coaching content coding.

Multimedia Appendix 2 LiveWell smartphone app content.

Multimedia Appendix 3 LiveWell use case scenario.

Multimedia Appendix 4 LiveWell coaching materials.

Multimedia Appendix 5 LiveWell design assessments.

Multimedia Appendix 6 LiveWell screening assessments.

Multimedia Appendix 7 LiveWell outcome and target assessments.

Multimedia Appendix 8 LiveWell determinant assessments.

## Abbreviations

ASRM: Altman Self-Rating Mania Scale
CMF: Clinical Monitoring Form
PHQ-8: 8-question Patient Health Questionnaire
QIDS: Quick Inventory of Depressive Symptomatology
QOL: quality of life
RCT: randomized controlled trial
TAU: treatment as usual
