# Supplementary material for: A Smartphone-Based Self-management Intervention for Individuals With Bipolar Disorder (LiveWell): Empirical and Theoretical Framework, Intervention Design, and Study Protocol for a Randomized Controlled Trial
Source: JMIR Res Protoc. 2022 Feb 21;11(2):e30710. doi: 10.2196/30710 (PMC8902672; doi:10.2196/30710)
Supplement: Multimedia Appendix 2 [file resprot_v11i2e30710_app2.pdf]

FOUNDATIONS

TABLE OF CONTENTS

1. **OVERVIEW**
   1.1. Bipolar Disorder
   1.2. Medications
   1.3. Self-Management
   1.4. *LiveWell* Program
   1.5. Get Ready…
   1.6. Get Set…
   1.7. Go!
   1.8. Key Points

2. **BASIC FACTS**
   2.1. Myth Busters
   2.2. Time Course
   2.3. Symptoms of Depression
   2.4. Early Warning Signs of Depression
   2.5. Symptoms of Mania
   2.6. Early Warning Signs of Mania
   2.7. Crises
   2.8. Triggers
   2.9. Treatment Options
   2.10. Support Organizations
   2.11. Key Points

3. **MEDICATIONS**
   3.1. Why Medications?
   3.2. Weigh Your Options
   3.3. Make a Decision
   3.4. Problem: Sticking With It
   3.5. Solution: Overcoming Barriers
      3.5.1. Misunderstandings About Bipolar
      3.5.2. Misunderstandings About Medication
      3.5.3. Negative Feelings About Bipolar
      3.5.4. Feeling Okay
      3.5.5. Forgetfulness
      3.5.6. Side Effects
      3.5.7. Poor Relationship With Doctor
      3.5.8. Discouragement from Family
      3.5.9. Expense or Inconvenience
   3.6. Key Points

4. LIFESTYLE SKILLS
- 4.1. Healthy Lifestyle
- 4.2. SMARTS
- 4.3. Sleep
- 4.4. Medications
- 4.5. Attend
- 4.6. Routine
- 4.7. Tranquil
- 4.8. Social
- 4.9. Key Points

5. COPING SKILLS
- 5.1. Defining the Problem
- 5.2. Building Coping Skills
- 5.3. Picture This…
- 5.4. Making the Right Personal Choice
- 5.5. Dial Up Skills
- 5.6. Dial Down Skills
- 5.7. Key Points

6. TEAM
- 6.1. It's a Team Effort
- 6.2. Psychiatrist
- 6.3. Finding a Psychiatrist
- 6.4. Working With Your Psychiatrist
  - 6.4.1. Be honest
  - 6.4.2. Be informed
  - 6.4.3. Reach out
- 6.5. Supports
- 6.6. Identifying Your Supports
- 6.7. Working With Your Supports
- 6.8. Hospital
- 6.9. Finding a Hospital
- 6.10. Mental Health Directives
- 6.11. Key Points

7. AWARENESS
- 7.1. Putting It Together
- 7.2. Being Aware
- 7.3. Check Ins
- 7.4. Daily Review
- 7.5. Charts
- 7.6. Key Points

8. ACTION
- 8.1. Putting It Together
- 8.2. Taking Action
- 8.3. Wellness Plan
- 8.4. Wellness Rating

8.5. Resources
8.6. Reduce Risk
8.7. Plan For Well
8.8. Plan For Slight Variations
8.9. Plan For Mild Symptoms
8.10. Plan For Moderate Symptoms
8.11. Plan For Severe Symptoms
8.12. Key Points

9. WRAPPING UP

9.1. Summary of Program
9.2. What Do You Think?
9.3. Future Plans
9.4. Thanks!

Overview

Bipolar Disorder

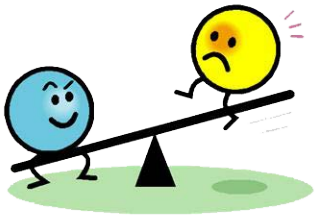

Bipolar disorder is a serious health condition. It is marked by mood swings. You feel sad or cannot enjoy life when depressed. You feel excited or highly irritable when manic. Some people only go through mania. Some people go through depression and mania at the same time. What have you experienced?

The **bad news** is that bipolar disorder causes problems. It impacts self-care, relationships, school, and work.

The **good news** is that bipolar disorder can be treated with a combination of medications and self-management.

Overview

Medications

Medications work.

They reduce symptoms and problems. It is recommended you take medication for the rest of your life. This includes when you have no symptoms.

There are a number of medications and everyone reacts differently. It is important to find the right ones for you. This requires working closely with a psychiatrist.

Overview

Self-Management

Symptoms can occur when you take medications. These are called break-through symptoms. Break-through symptoms can lead to full-blown depression or mania.

Adding self-management to medications can cut symptoms and relapses by about half. This is a big deal!

Self-management includes:

- Living a healthy lifestyle
- Managing early signs of illness
- Using your team

Overview

LiveWell Program

The *LiveWell* Program is built to help you lead a healthy life.

The *LiveWell* Program will help you reduce symptoms. It will help you reduce problems. The program provides:

- Knowledge
- Motivation
- Skills

The tools available here will help you to live well. The rest is up to you. You need to take action to make it work!

Overview

Get Ready…

Are you ready to try something new? Are you ready to practice?

You will need the motivation to follow through. There are two keys to motivation. You can say “It is all about ME.”

You are motivated when your actions are **meaningful.** What are your short and long-term goals? How will using this self-management program help you? How will staying well help you reach your goals?

You are also motivated when your actions are **effective.** This program will help you succeed in managing your illness and life.

Overview

Get Set…

Are you set to learn facts and tools to help you manage your bipolar disorder?

- **Foundations**: These lessons contain information crucial to managing bipolar disorder.
- **Check Ins**: This log helps you track symptoms, sleep, and medications.
- **Daily Review**: This helps you see how you are doing and provides feedback.
- **Wellness plan**: This section contains your individual plan for staying well and also provides you with additional resources to help reduce your risk for future problems.

Overview

Go!

Are you ready to get going?

You will learn the basics. You will customize your program. You will get feedback about how you are doing. You will get suggestions on how to make positive changes in your life.

The *LiveWell* Program is largely a self-management program. It helps you develop knowledge and skills. This will take work every day on your part, but it will get easier over time.

The *LiveWell* Program is NOT a substitute for other treatments. It also is NOT an emergency service. In the case of life threatening situations, such as suicidal intentions, it is crucial that you get immediate care.

7 out of 8

Overview

Key Points

Getting a good handle on bipolar disorder can take some time. It’s not a sprint, but it’s not a marathon either. It’s more like a 5K. You need to prepare and train.

You can live well if you:

- Live a healthy lifestyle
- Manage early signs of illness
- Use your support team

Over the next 16 weeks, you will learn lots of new skills and strategies through *LiveWell.* Others have successfully completed our program. You can, too!

Basic Facts

Myth Busters

Bipolar disorder…

- Is not uncommon
- Is not a sign of personal weakness
- Is not a mystery
- Is not uncontrollable

Bipolar disorder affects 1 in 100 people. It is a medical disease. It is a mood disorder. It also impacts people's bodies, thoughts, and behaviors.

Diagnosis is not destiny. Use medications and self-management. You can survive. In fact, you can thrive.

Basic Facts

Time Course

Bipolar disorder usually starts between the ages of 18 and 30. It involves periods of depression and mania. Low level mania is called hypomania. Having both manic and depressive symptoms at the same time is called a mixed episode.

Most people have many mood episodes. Over 95% of those who have had one episode will have additional episodes in their lifetime.

Early warning signs come before full-blown mood episodes. These signs are often low level symptoms of depression or mania and may be hard to notice.

Residual symptoms often follow full-blown mood episodes. The most common persistent symptom is low level depression.

How well people do over time depends on:

- Taking medications
- Recognizing early warning signs
- Keeping symptoms under control
- Getting the right amount of sleep
- Keeping a regular schedule
- Not using substances
- Managing emotions
- Maintaining self-esteem
- Having a support system

Basic Facts

Symptoms of Depression

The hallmark of depression is low mood or reduced interest and pleasure in life.
Other symptoms include:

- Weight loss or weight gain
- Sleeping too much or too little
- Physical agitation or slowing down
- Fatigue or loss of energy
- Feeling worthless or guilty
- Difficulty concentrating
- Thoughts of death or suicide

A full-blown **depressive episode** requires low mood or reduced interest or pleasure plus 4 or more other symptoms that are present most days for a period of at least 2 weeks.

Basic Facts

Early Warning Signs of Depression

Many people notice low level symptoms or other signs well before entering a depressive episode. Some common early warning signs of depression are:

- Less energy than usual
- Less interest than usual
- Problems concentrating
- Negative thinking
- Sad or anxious mood
- Sleep changes
- Don’t feel like seeing people
- Feeling guilty
- Loss of appetite
- Difficulty making decisions
- Low self-confidence
- Less interest in sex

Basic Facts

Symptoms of Mania

The hallmark of mania is feeling overly happy and excited or feeling irritable. Other symptoms include:

- Increased esteem/feeling superior
- Decreased need for sleep
- More talkative than usual
- Racing thoughts
- Difficulties concentrating
- Increased activity level
- Risky activities

A full-blown **manic episode** requires an overly positive or irritable mood plus 3 or more other symptoms that are present for at least one week.

Basic Facts

Early Warning Signs of Mania

Many people notice low level symptoms or other signs well before entering a manic episode. Some common early warning signs of mania are:

- Sleep disturbance
- More active than usual
- More social than usual
- More talkative than usual
- Euphoric or irritable mood
- More energy than usual
- Increased self-confidence
- Increased sex drive
- Racing thoughts
- Feeling more creative
- Senses seem sharper
- Overspending

Basic Facts

Crises

Severe depression and severe mania can cause crises. The following symptoms are considered a crisis:

- Dangerous behaviors that put you at risk for serious consequences
- Thoughts of suicide that include planning and the intent on following through
- Psychotic symptoms such as delusions or hallucinations

If you have any of these crisis symptoms, call your psychiatrist, call 911, or go to the nearest emergency room right away.

Basic Facts

Triggers

Bipolar disorder runs in families. It is inherited. However, genes do not guarantee illness. Personal and environmental factors can trigger the disorder. A number of things can trigger symptoms once the illness emerges. Some examples are:

- Discontinuing medications
- Sleep changes
- Disruptions in routines
- Too much involvement in activities
- Being stressed
- Negative life events
- Critical family interactions
- Substance use

Basic Facts

Treatment Options

Medications are necessary. Self-management helps. Other treatments can help as well.

- **Psychotherapy** helps people reduce stress. It helps people address problems with self-esteem and relationships.
- **Family education** helps families learn more about bipolar disorder.
- **Family therapy** helps families learn to better solve problems and conflicts.
- **Substance abuse treatment** can help individuals reduce problematic use.

Basic Facts

Support Organizations

Get involved!

- Depression and Bipolar Support Alliance: 312-642-0049 or www.dbsalliance.org
- National Alliance for Mental Illness: 703-524-7600 or www.nami.org
- National Institute of Mental Health: 301-443-4513 or www.nimh.nih.gov

Basic Facts

Key Points

Bipolar disorder:

- Is a common medical condition
- Includes early warning signs
- Can be effectively managed

Medications

Why Medications?

Research shows that medication is critical for people with bipolar disorder to live well.

During an episode: Symptoms can last for months without medication. Symptoms harm relationships and the ability to work or engage in other meaningful activities. With medication, mood episodes may be resolved in a matter of weeks.

When well or better: Not taking medication is associated with more frequent relapses, hospitalizations, and suicidal thoughts. Medication cuts the chances of future mood episodes by half.

Medications

Weigh Your Options

What are your thoughts on medications?

What good things have come from taking medications? What bad things have come from taking medications?

- How did you feel?
- How did you think?
- How was your self-care?
- How were your relationships?
- How was your work?

Think about your goals. Does taking medication help you pursue your goals? What are the pros and cons of taking medications for you?

Medications

Make a Decision

Do you have doubts about taking medications?

Think about your list of pros and cons. What should you do?

Although long-term medications are recommended, you only need to make a decision about what you are going to do right now and for the near future.

Consider your thoughts and feelings. Consider how you are functioning. Consider the advice of those who love you. Consider the recommendations of your doctor.

Be wise!

Medications

Problem: Sticking With It

Do you waver in your commitment to medications?

Following medical advice is hard. Only about 50% of people take medication as prescribed. This is true for all sorts of conditions including diabetes and hypertension. It is true for bipolar disorder as well. Reasons people don’t take medications are:

- Misunderstandings about the illness
- Misunderstandings about medication
- Negative feelings about the illness
- Feeling okay
- Forgetfulness
- Side effects/Fear of side effects
- Poor relationship with doctor
- Discouragement from family
- Expense or inconvenience

Medications

Solution: Overcoming Barriers

Misunderstandings About Bipolar

There is little information available for the public. There is a lot of stigma and fear.

Get informed! **Read!** Make sure you understand the nature of bipolar disorder. **Reflect!** Make sure you understand your bipolar disorder. People often think they are fine when they are manic. Trust your family and doctor to be honest with you.

If you stopped taking medications and others think you are in an episode, take medications for two weeks to clear your mind (even if you think things are fine). Then reconsider your situation.

Medications

Solution: Overcoming Barriers

Misunderstandings About Medications

People often have negative beliefs about medications.

Medication for bipolar disorder does **not** change people's personalities. It only helps take away the mood highs and lows.

Taking medication is a sign of health and taking care of yourself. It is **not** a sign of weakness.

Medications

Solution: Overcoming Barriers

Negative Feelings About Bipolar

Being angry, sad, or scared is a normal reaction to an abnormal situation. Who wants to have a disorder? Nobody!

The fact is that each and every one of us on this earth will face major challenges in our lives. You are no more or less a person for having bipolar disorder.

Work through your feelings about this disorder. Start therapy or join a support group if you want help in this way.

Medications

Solution: Overcoming Barriers

Feeling Okay

So how can feeling okay be a problem you ask?

When people begin to feel better they often think the disorder has gone away, so they stop taking medications. When you feel well you might want to stop your medications. However, stopping your medications makes it more likely you will get symptoms again.

Make a pact with yourself that you will continue taking medications when ill and when well. Stay well! Live well!

Medications

Solution: Overcoming Barriers

Forgetfulness

It is easy to forget to take medications. Some people forget often. And most people forget occasionally. Try the following tips:

- Take your medication at the same time every day
- Keep your medication in the same place
- Put your medication next to something that you do every day such as your toothbrush, coffee pot, or alarm clock
- Set a medication alarm
- Keep a pill box

Medications

Solution: Overcoming Barriers

Side Effects

Negative side effects of medications are bothersome. Talk to your doctor!

- Make sure you are on the right medications for you
- Make sure you are on the lowest dose necessary
- Ask if there is a medication to reduce unwanted side effects
- Make sure the positive effects of the medication outweigh any negative side effects

Medications

Solution: Overcoming Barriers

Poor Relationship With Doctor

It is very important that you are comfortable with your doctor. Make sure that you:

- Find a doctor who is a good match. It is important that you are comfortable with her or his bedside manner.
- Work on having an open dialogue with your doctor. She or he is an expert on medications. You are the expert on your needs and goals.
- Be sure to tell your doctor if she or he is making you uneasy.

Medications

Solution: Overcoming Barriers

Discouragement From Family

Sometimes family members discourage people from taking medications. This often has to do with not wanting to believe that there is a problem. Sometimes it has to do with mistrusting the mental healthcare system.

Invite your loved ones to a family meeting or educational session with your doctor. They need to work through their own misunderstandings and negative feelings about bipolar disorder.

Medications

Solution: Overcoming Barriers

Expense or Inconvenience

Medications are expensive. Going to the doctor is inconvenient. Make sure you are on the health insurance plan that best serves your needs. Make sure you budget money for medications. They are life-saving.

Schedule your doctor appointments in a way that is least disruptive to your life. There is no way around it. Taking care of bipolar disorder requires time, effort, and money.

Medications

Key Points

Remember…

- Medications are an important part of treatment for bipolar disorder
- More people than not run into trouble taking medications regularly
- Common problems with taking medications can be overcome
- Work closely with your psychiatrist to make medications work for you

Lifestyle Skills

Healthy Lifestyle

Remember that triggers can bring on symptoms. Examples of triggers are:

- Sleep changes
- Disruption in routines
- Over involvement in activities
- Being stressed
- Negative life events
- Critical family interactions
- Substance use

Maintaining a healthy lifestyle can significantly reduce your risk of future episodes.

Lifestyle Skills

SMARTS

Take care of yourself! Be wise! Use your SMARTS and keep a healthy lifestyle.

- **S**leep: Get enough sleep every night
- **M**edication: Take medications as prescribed
- **A**ttend: Attend to diet, exercise, and substances
- **R**outine: Keep a regular daily routine
- **T**ranquil: Aim for calm
- **S**ocial Keep healthy relationships

Below you will learn more about using your SMARTS to stay well. For more tips and strategies, visit the application's Lifestyle section of the Toolbox.

Lifestyle Skills

Sleep

Having a regular sleep schedule is important. Changes in the **timing** of sleep can trigger symptoms. Getting enough sleep is also important. Changes in the **amount** of sleep can trigger symptoms.

Anywhere between 6 to 10 hours a night is normal. However, people do vary. To get a good night's sleep, practice these habits daily:

- Go to bed at the same time each night
- Get up at the same time each morning
- Use the bed for sleep and sex only
- Get up if you can't fall asleep after 15 to 20 minutes
- Go back to bed only when sleepy
- Avoid taking naps

Lifestyle Skills

Medications

Medication is an essential part of any treatment plan for bipolar disorder.

Be sure to take your medications as prescribed each and every day. If you are having any problems with your medications, consult your doctor.

Lifestyle Skills

Attend

Attend to diet, exercise, and substance use. Having a good diet, being active and exercising, and minimizing your use of substances will help you keep symptoms of bipolar disorder at bay.

Eat well…

The United States Food and Drug Administration suggests eating a range of “nutrient dense” foods. To maintain the integrity of the foods, prepare them with little or no added solid fats, sugars, refined starches, and sodium.

- A variety of vegetables: dark green, red and orange, beans and peas, starchy, and other vegetables
- Fruits, especially whole fruits
- Grains, at least half of which are whole grains
- Fat-free or low-fat dairy, including milk, yogurt, cheese, and fortified soy beverages
- A variety of protein foods, including seafood, lean meats and poultry, eggs, beans and peas, soy products, and nuts and seeds
- Oils, including those from plants and oils that are naturally present in foods

Stay active…

The USFDA also has physical exercise guidelines. For adults:

- At least 2.5 hours of moderate intensity physical activity every week
- Muscle strengthening exercises on 2 or more days each week

Watch substances…

Psychoactive substances are things individuals ingest that impact their mental state. This includes things such as nicotine, caffeine, alcohol, cannabis and other illegal drugs. Overuse or misuse causes mental, physical and social problems. Even small amounts can interact with medications prescribed for bipolar disorder.

In terms of alcohol, the most common problematic substance for most individuals, the National Institutes of Health recommends:

- No more than 4 drinks/day and no more than 14 drinks/week for men
- No more than 3 drinks/day and no more than 7 drinks/week for women

Higher amounts are considered risky drinking and put people at risk for physical and mental health complications.

Lifestyle Skills

Routine

Routine. Regularity. Rhythm. This is important for health. It is vital to managing bipolar disorder.

What does your schedule look like? Make a plan. Keep a routine for sleep. Keep a routine for activities.

In particular, try to do these 5 activities at about the same time every day:

- Get out of bed
- First have contact with another person
- Start work/school/volunteer/family care
- Dinner
- Get to bed

Lifestyle Skills

Tranquil

Stress is part of life. It is normal. However, intense or prolonged stress is not normal. It causes many physical health problems. It causes many mental health problems. Stress can also trigger symptoms.

Live a grounded life. Eliminate unnecessary stress. Look at your financial, housing, social, and work situations. Are they good enough? Do you need to change anything?

Live an empowered life. Consider your ability to manage intense emotions. Consider your self-esteem. Is it good enough? Do you need to improve? Try psychotherapy if you need help.

Lifestyle Skills

Social

We are social beings. We need to feel connected to others. We need support.

Social problems cause stress. They can trigger symptoms. The reverse is also true. Symptoms can cause social problems. The withdrawal of depression can push others away. The irritability of mania can push others away.

Work to have caring relationships with others. Work to stay connected in healthy ways, even when having symptoms. If you need help consider psychotherapy or family therapy.

Lifestyle Skills

Key Points

Be SMARTS:

- Get enough **sleep** every day
- Take **medications** as prescribed
- **Attend** to diet, exercise, and substance use
- Keep a regular daily **routine**
- Manage your stress to stay **tranquil**
- Maintain a healthy **social** life

Coping Skills

Defining the Problem

Here is the problem…

Depression is a spiral down. People withdraw. People become inactive. These behaviors worsen depression. **Dial Up** to avoid depression.

Mania is a spiral up. People increase engagement. People increase activity and decrease sleep. These behaviors worsen mania. **Dial Down** to avoid mania.

There are skills you can use to reduce your chances of full-blown mood episodes.

Coping Skills

Building Coping Skills

The coping skills here are based on a cognitive-behavioral theory of the human mind. This theory is based on three principles:

- Our thoughts, actions, feelings, and bodies all impact each other
- We can be aware of our thoughts, actions, feelings, and bodies
- We can make changes in life by adjusting our thoughts and actions

In the *LiveWell* Program we will focus primarily on **awareness** and **action** as a path to wellness.

Coping Skills

Picture This…

This is how it works. Everything is connected. Coping skills—taking **action**—can be used to promote healthy thoughts, feelings, and bodies. This is true when dealing with early warning signs of depression and mania.

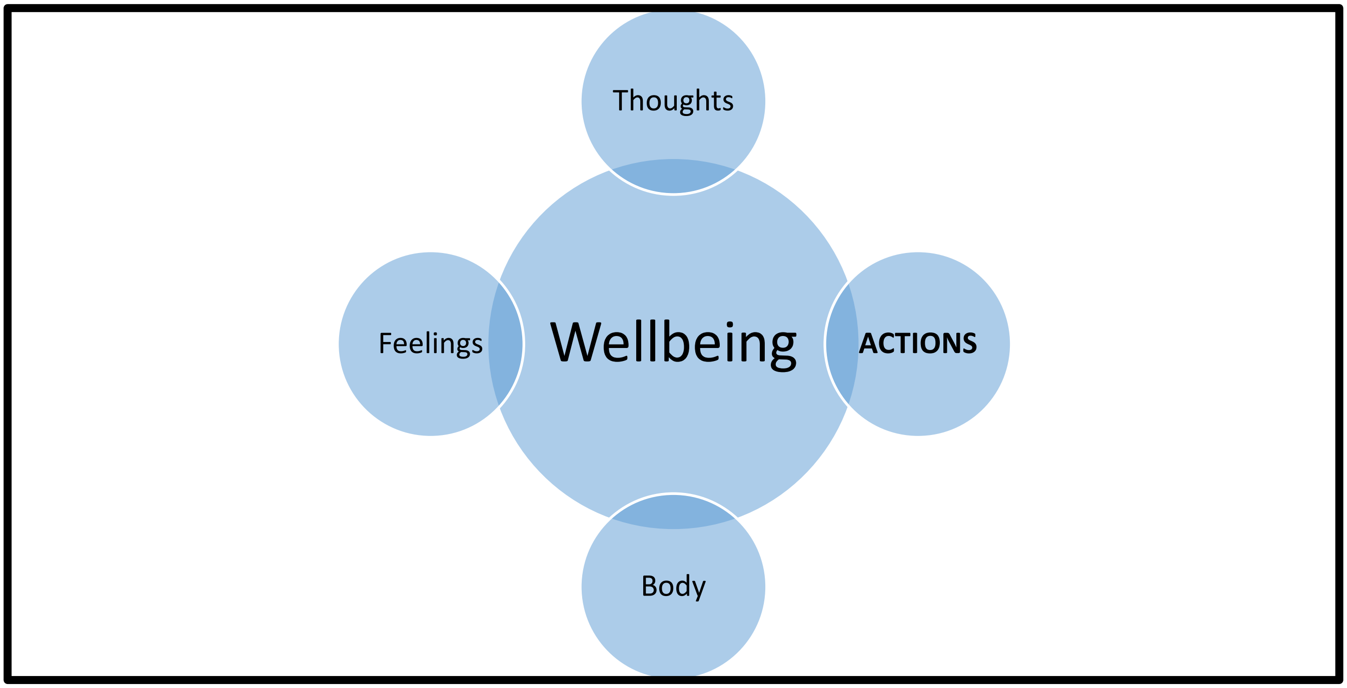

Coping Skills

Make The Right Personal Choice

Be sure that you consider what is best for you in the long-run. Don’t give in to what feels good in the moment.

- Be **aware**
- Consider your options
- Choose wisely
- Take **action**

Remember you can use **Dial Up** and **Dial Down** skills to prevent a spiral into depression or mania.

Coping Skills

Dial Up Skills

What skills can you use for early warning signs or symptoms of depression? Get involved! Get moving!

- Consider skills you already use in life
- Develop new skills on your own
- Ask a therapist to teach you
- Join a skills group in the community
- Try these or other self-help books

- *The Bipolar Workbook by Basco (2006)*
- *Managing Bipolar Disorder by Otto (2009)*

Check out **Dial Up** in the Toolbox section of this application to begin learning new skills.

Coping Skills

Dial Down Skills

What skills can you use for early warning signs and symptoms of mania? Disengage! Slow down!

- Consider skills you already use in life
- Develop new skills on your own
- Ask a therapist to teach you
- Join a skills group in the community
- Try these or other self-help books

- *The Bipolar Workbook by Basco (2006)*
- *Managing Bipolar Disorder by Otto (2009)*

Check out **Dial Down** in the Toolbox section of this application to begin learning new skills.

Coping Skills

Key Points

Watch your activity level:

- Dial up when you're down
- Dial down when you're up

Team

It’s A Team Effort

Managing bipolar disorder requires taking medications. It requires maintaining a healthy lifestyle. It requires using coping skills. It requires having a good team in place.

At a minimum, your team involves a psychiatrist, personal supports, and a hospital.

For some people, a therapist, case manager, and peer support groups are important as well.

1 out of 13

Team

Psychiatrist

Having a good relationship with your psychiatrist is important.

Your health depends upon their expertise. While you know yourself best, your psychiatrist should know the most recent information on medications for bipolar disorder. You need a doctor with whom you can collaborate.

Plus, having a good relationship with your psychiatrist makes it more likely that you will reach out to them when you need their help. It also makes it more likely that you will follow through on their recommendations.

Team

Finding A Psychiatrist

It is common to meet with or work with several different psychiatrists before finding the one that works best for you.

- Be sure your psychiatrist is an expert on bipolar disorder.
- Find someone who is flexible, honest, respectful, trustworthy, confident, warm, interested, and open.
- Check that his or her personality is a good match for you. What style works best for you?

Team

Working With Your Psychiatrist

Once you find a psychiatrist that is a good match for you, do your part to establish and maintain a good working relationship.

Be open

This can be harder than it sounds. It is common for people to not want to share certain things with their psychiatrist. This includes missing medications, using substances, and having early warning signs of mania or hypomanic symptoms. Things to share with your psychiatrist:

- Symptoms
- Sleep habits
- Medication use
- Substance use
- Significant life changes

Team

Working With Your Psychiatrist

Be informed

It is important that you and your psychiatrist are on the same page. Things to ask your psychiatrist:

- What is my diagnosis?
- How will the medications help?
- What are the side effects?
- What should I do if I get side effects?
- What should I do if I miss a dose?
- When should I call you?

Team

Working With Your Psychiatrist

Reach out

Have an agreement with your psychiatrist about when you should call them between sessions. This usually involves things such as:

- Problems with medications
- Sleep disturbances
- Early warning signs
- Symptoms of depression
- Symptoms of mania

Sometimes symptoms keep people from reaching out. The hopelessness of depression and thrill of mania can get in the way. Don’t let your symptoms make decisions for you!

Team

Supports

It is important to have a few people in your life, not practitioners, that you can rely on to help you manage bipolar disorder.

The research on this point is clear. Support is a major factor in getting and staying well. So think about this carefully.

Team

Identifying Your Supports

Think about your family and friends. Who amongst them might serve as effective team members? Pick two or three people. They should:

1. Know you well and care about you
2. Understand bipolar disorder or be willing to learn about it
3. Respect the need for medications to treat bipolar disorder
4. Not be critical or intrusive

If you don’t have any family members or friends that fit this description, consider joining a support group.

Team

Working With Your Supports

Show them your wellness plan. You will complete this with your *LiveWell* coach after reading Awareness and Action Foundation lessons. Make sure you go through your plan in detail with your supports.

Talk explicitly with your supports about your early warning signs of illness. Make sure they understand what red flags to look for.

Share with them exactly how you would like them to give you feedback if they think you are becoming ill. Let them know what you'd like them to do if the situation arises where you are dismissive of their concerns. Should they call your psychiatrist? Should they wait a day and try again?

Team

Hospital

There are times when you may need short-term treatment in the hospital even if you do everything right. That is, even if you take medications regularly, live a healthy lifestyle, and use coping skills, there is a chance you will have break-through symptoms.

This is not the end of the world. Just know in advance where you would like to go for inpatient care.

Get in. Get well. Get out.

Team

Finding A Hospital

Here are some things to consider when picking out a hospital:

- What hospitals are ranked the highest? Check out the latest *US News and World Report*.
- What is the treatment philosophy of the psychiatry departments at nearby hospitals? Read about the inpatient unit online.
- Is your psychiatrist on staff at nearby hospitals? Being on staff helps with the continuity of your care.

Team

Mental Health Directives

Consider completing a declaration for mental health treatment. This is a legal document that allows you to make decisions in advance about mental health treatment when you are well. It includes instructions for inpatient care should the situation arise in which you are hospitalized for severe symptoms.

Search the internet for Advanced Directive for Mental Health for a copy of your state's specific form. These generally include:

- Preferred hospital
- Preferred psychiatrist
- Consent for specific medications
- Limits on specific medications
- Attorney-in-fact (person authorized to make decisions for you if you become unable to give informed consent)

Team

Key Points

Having a good team in place is critical to managing bipolar disorder. Your team should include:

- Psychiatrist
- Personal supports
- Hospital

Awareness

Putting It Together

We have covered a lot of information. All of it is important. Each topic is critical to managing bipolar disorder:

- Basic facts
- Medications
- Lifestyle skills
- Coping skills
- Team support

Putting it together means:

- Developing your own wellness plan
- Following your wellness plan

Your wellness plan involves being **aware** and taking **action**!

The *LiveWell* program is designed to help you put all of this information together in a way that makes sense and is easy to use. All pertinent personal information will be readily accessible to you!

Awareness

Being Aware

…means being mindful.

Identify risk factors for depression and mania. This involves monitoring your lifestyle choices, including your sleep habits and medication compliance.

Identify signs of depression and mania. This involves monitoring your daily wellness ratings and being attentive to any early warning signs of illness.

2 out of 6

Awareness

Check Ins

The *LiveWell* Program includes daily and weekly check ins. Use check ins to become more aware of your day to day state of mind. Use check ins to become more aware of patterns and triggers for symptoms. Each day you will rate:

- Medication use
- Sleep time
- Routine
- Overall wellness

Each week you will complete questionnaires and checklists to identify any:

- Symptoms of depression
- Symptoms of mania
- Early warning signs

Awareness

Daily Review

The *LiveWell* Program provides daily feedback to help you maintain awareness and identify opportunities for action. To access your feedback, visit the Daily Review section of the application.

The Daily Review presents a summary of your information for the previous 7 days:

- Medications
- Sleep
- Routine
- Wellness

It also includes recommendations for wellness.

Awareness

Charts

Charts lets you look at changes over the past week in your:

- Medication use
- Sleep
- Daily routine
- Wellness rating

It also allows you to see how the different areas relate to each other. For example, it shows how sleep and wellness are connected.

Charts will also make it easier to notice triggers or early warning signs. Becoming more aware will help you take action sooner to stay well. This can help you avoid full-blown mania or depression. Try it out!

Visit **My Charts** in your Wellness Plan for more information.

Awareness

Key Points

Rate your mood daily:

- Watch for early warning signs of depression
- Watch for early warning signs of mania
- Notice ongoing symptoms of depression
- Notice ongoing symptoms of mania

Action

Putting It Together

We have covered a lot of information. All of it is important. Each topic is critical to managing bipolar disorder:

- Basic facts
- Medications
- Lifestyle skills
- Coping skills
- Team support

Putting it together means:

- Developing your own wellness plan
- Following your wellness plan

Your wellness plan involves being **aware** and taking **action**!

The *LiveWell* program is designed to help you put all of this information together in a way that makes sense and is easy to use. All pertinent personal information will be readily accessible to you!

Action

Taking Action

…means being effective.

- **Reduce risk** by taking medications and using lifestyle skills.

- **Manage slight variations** by being mindful. Use skills to manage any triggers.

- **Manage mild symptoms and early warning signs** by using skills. Dial up for depression and dial down for mania. Use supports as well. Keep your psychiatrist informed about any changes in your mood.

- **Manage moderate symptoms** by using your team. Reach out to your supports and psychiatrist. If your symptoms persist, consider more intensive treatment, like a partial hospitalization program or an intensive outpatient program. Also consider increasing the frequency in which you attend psychotherapy, at least until your symptoms remit. If you are not in therapy, consider starting.

- **Manage severe symptoms** by using your team. Reach out to your supports. Call your psychiatrist. If you are in crisis, call 911 or go to your nearest emergency room.

Action

Wellness Plan

In the pages ahead, we have included standard recommendations on how to effectively manage bipolar disorder. These recommendations were also incorporated into a standard wellness plan that can be found in the Wellness Plan section of the application.

In the coming weeks, your *LiveWell* coach will help you customize your plan based on your own triggers, early warning signs, and symptoms. Once completed, you will be able to review this personalized plan whenever you want through the Wellness Plan.

Action

Wellness Ratings

Okay. Now you know more. If you need to make any changes, your *LiveWell* coach will help you update your wellness rating scale. Think…

- What are you like when **slightly** up or down in response to good or bad events?
- What are you like when you are **mildly** up or down? What are your personal early warning signs? What are your first symptoms?
- What are you like when you are **moderately** manic or depressed? What are your symptoms?
- What are you like when you are **severely** manic or depressed? What were your symptoms during your worst episode? What were your symptoms if you were hospitalized?

Action

Resources

Have your resources lined up in advance. Preparation is the key!

What are your…

- Medications
- Lifestyle goals
- Coping skills
- Team members
- Crisis plans

Action

Reduce Risk

Have your ideas lined up in advance. Preparation is the key!

What are your plans for…

- **S**leep
- **M**edications
- **A**ttend
- **R**outine
- **T**ranquil
- **S**ocial

Action

Plan For Well

Have your ideas lined up in advance. Preparation is the key!

What are your plans for times when you are well?

Your wellness rating is a **0.**

Our recommendations are that you take your medications and:

- Maintain a healthy lifestyle.

7 out of 12

Action

Plan For Slight Variations

Have your ideas lined up in advance. Preparation is the key!

What are your plans for times when you are **slightly** up or down? This is a response to daily good or bad events. This is most likely a normal variation in mood.

Your wellness rating is a **+1 or -1.**

Our recommendations are that you take your medications and:

- Manage stress with lifestyle skills

Action

Plan For Mild Symptoms

Have your ideas lined up in advance. Preparation is the key!

What are your plans for times when you are **mildly** up or down? This means early warning signs or a few symptoms may be present. A full-blown episode may be coming. However, you are still able to maintain your usual activities and routines.

Your wellness rating is a **+2 or -2.**

Our recommendations are that you take your medications and:

- Use lifestyle skills
- Use coping skills
- Use supports

Action

Plan For Moderate Symptoms

Have your ideas lined up in advance. Preparation is the key!

What are your plans for times when you are **moderately** manic or depressed? This means many symptoms are present day to day. A full-blown episode is probably happening. It is difficult to maintain your usual activities and routines.

Your wellness rating is a **+3 or -3.**

Our recommendations are that you take your medications and:

- Call your supports
- Call your psychiatrist
- Increase treatment frequency
- Consider more intensive treatment

Action

Plan For Severe Symptoms

Have your ideas lined up in advance. Preparation is the key!

What are your plans for times when you have severe mania or depression? You may be engaging in dangerous behaviors, thinking about suicide with some intent to act on thoughts, or have psychotic symptoms such as hallucinations or delusions. You cannot maintain your daily routines and activities. **This is a crisis.**

Your wellness rating is a **+4 or -4.**

Our recommendations are that you take your medications and:

- Call your psychiatrist
- Call 911
- Go to the hospital
- Let your supports know what is happening

Action

Key Points

Take action:

- Use skills to cope with your mild ups and downs
- Reach out to your team when you are moderately up or down
- Consider the hospital when your symptoms are severe

Wrapping Up

Summary of Program

Bipolar disorder is a serious medical condition that requires attention.

The bad news is that the symptoms can cause problems in life. They can cause pain and interfere with self-care, relationships, school, and work. They can lead to hospitalizations.

The good news is that bipolar disorder can be managed. Taking medications consistently, maintaining a healthy lifestyle, and taking quick action around early warning signs and symptoms are the keys to living well.

Remember that diagnosis does not determine destiny! How you manage goes a long way in determining how things unfold for you.

The *LiveWell* application offers a self-management program for bipolar disorder using state-of-the-art technology. We hope that you learned something valuable from your participation that will help you stay well in the years to come.

Remember the keys to success:

- Take your medications
- Live a healthy lifestyle
- Manage early signs of illness
- Use your team

Wrapping Up

What Do You Think?

Take a moment to reflect on the program. Give your coach feedback.

- What were the most important things you learned about bipolar disorder?
- What were the most important things you learned about yourself?
- Did you try making lifestyle changes? If so, how did it go?
- Did you try dial up and dial down skills for managing mild symptoms? If so, how did it go?
- Did you try reaching out when symptoms got serious? If so, how did it go?
- What parts of the *LiveWell* program did you find most useful? How come?
- What parts of the *LiveWell* program did you find least useful? How come?
- Will you do anything different in the future as a result of participating in the *LiveWell* program? If so, what?

Wrapping Up

Future Plans

What will you do in the future…

- When you are well?
- When you have mild symptoms?
- When you have moderate symptoms?
- When you have serious symptoms?

Wrapping Up

Thanks!

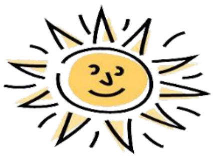

Thank you for completing the *LiveWell* program. Hopefully you found the information and exercises useful.

Keep up with what worked. Make a commitment to yourself to use those strategies regularly. Take care of yourself and be well.

Good luck and best wishes!

TOOLBOX

TABLE OF CONTENTS

MAKING CHANGES

1. Introduction
    1.1. Get Prepared
        1.1.1. About
        1.1.2. Information
        1.1.3. Attitudes
        1.1.4. Confidence
    1.2. Set Goal
        1.2.1. About
        1.2.2. Outcomes
        1.2.3. Targets
        1.2.4. Commitment
    1.3. Develop Plan
        1.3.1. About
        1.3.2. Specify Targets
        1.3.3. Confirm Resources
        1.3.4. Identify Ways to Overcome Obstacles
    1.4. Monitor Behavior
        1.4.1. About
        1.4.2. Monitor Behavior
    1.5. Evaluate Performance
        1.5.1. About
        1.5.2. Review Target Behavior
        1.5.3. Review Outcome Goals

SELF-ASSESSMENT

2. Introduction
    2.1. Symptoms and Triggers
        2.1.1. About
        2.1.2. Symptom History
        2.1.3. Signs Checklist
        2.1.4. Triggers Checklist
        2.1.5. Substance Use Questionnaire
        2.1.6. Beliefs About Medications
    2.2. Skills and Strengths
        2.2.1. About
        2.2.2. Coping Skills Checklist
        2.2.3. Strengths Checklist
        2.2.4. Values Checklist

2.2.5. Happiness Survey
2.2.6. Opinions about Recovery
2.3. Supports and Environment
2.3.1. About
2.3.2. Quality of Life Survey
2.3.3. Social Support Survey

LIFESTYLE

3. Introduction
3.1. Sleep
3.1.1. About
3.1.2. Three Day Sleep Challenge
3.1.3. Digital and Diet "Detox"
3.1.4. Worry Buster 1
3.1.5. Worry Buster 2
3.1.6. Guided Imagery
3.1.7. Sleep Diary
3.2. Medications
3.2.1. About
3.2.2. Educate Yourself about Medications
3.2.3. Solve Problems with Medications
3.2.4. Resolve Conflicts about Medications
3.2.5. Get Support around Medications
3.3. Attend
3.3.1. About
3.3.2. Thirty Day Substance Trial
3.3.3. People, Places, Things
3.3.4. HALT
3.3.5. Avoid Temptation
3.3.6. Deal With Urges
3.3.7. Sobriety Log
3.3.8. Nutrition Challenge
3.3.9. Exercise Challenge
3.3.10. Substance Challenge
3.4. Routine
3.4.1. About
3.4.2. Mastery and Pleasure-Based Routine
3.4.3. Values-Based Routine
3.4.4. Recovery-Based Routine
3.4.5. Activity Log
3.5. Tranquil
3.5.1. About
3.5.2. Problem Solving
3.5.3. Bird's Eye View
3.5.4. Deep Breathing
3.5.5. Muscle Relaxation

3.5.6. Self-Soothe
3.5.7. Improving the Moment
3.5.8. Mindfulness
3.5.9. Radical Acceptance
3.5.10. Stress Log
3.6. Social
3.6.1. About
3.6.2. Promoting Understanding
3.6.3. Promoting Mutuality
3.6.4. Setting Boundaries
3.6.5. Active Listening
3.6.6. Respectful Messaging
3.6.7. Conflict Resolution
3.6.8. Socialization Diary

COPING

4. Introduction
4.1. Depression – Dial Up
4.1.1. About
4.1.2. Graded Activity
4.1.3. Graded Mastery
4.1.4. Graded Pleasure
4.1.5. Activate Your Body
4.1.6. Activate Your Senses
4.1.7. Activate Your Mind
4.1.8. Increase Contacts
4.1.9. Increase Conversations
4.1.10. Increase Offers
4.1.11. DIY up
4.2. Mania – Dial Down
4.2.1. About
4.2.2. PACED Goals
4.2.3. PACED Mastery
4.2.4. PACED Pleasure
4.2.5. Deactivate Your Body
4.2.6. Deactivate Your Senses
4.2.7. Deactivate Your Mind
4.2.8. Decrease Contacts
4.2.9. Decrease Conversations
4.2.10. Decrease Offers
4.2.11. DIY down

TEAM

5. Introduction
5.1. Psychiatrist
5.1.1. About

- 5.1.2. General Guidelines
- 5.1.3. Ongoing Symptoms
- 5.1.4. Early Warning Signs
- 5.2. Supports
  - 5.2.1. About
  - 5.2.2. Why Supports?
  - 5.2.3. Making It Work
- 5.3. Hospital
  - 5.3.1. About
  - 5.3.2. Picking a Hospital
  - 5.3.3. When to Go to the Hospital
  - 5.3.4. What to Expect

Making Changes

Research suggests that there are 5 crucial steps to making lasting behavior changes in life. This is true no matter what the behavior.

It could be trying to take medications on a consistent basis, getting a better daily routine, eating better, or exercising more regularly. Check out the sections below for more information. Try the suggestions and see if you can't be more effective in your life!

Get Prepared

Set Goal

Develop Plan

Monitor Behavior

Evaluate Performance

Get Prepared

About

There are 5 steps that help in developing new habits: getting prepared, setting a goal, developing a plan, monitoring behavior, and evaluating performance. Consideration for each step will help you be more effective in making changes.

STEP 1 involves getting prepared. You are more likely to commit to a change if there is a lot at stake, if the consequences are important, and if you are confident you have what it takes to change. Read more about this…

Information

People are more likely to commit to changing when they consider themselves at high-risk for relatively severe consequences of not changing.

- What do you risk by not making a change?
- How severe do you consider this risk to be?
- How likely do you think it is that this will happen to you should you not make a change?

For example, perhaps you are aware that not taking medications increases people's risk of manic and depressive episodes and ending up in the hospital. At the same time, you might not believe it applies to you because you live a healthy lifestyle.

Attitudes

People are more likely to commit to changing when the consequences are important to them, and when the advantages outweigh the disadvantages of making a change. Consider the following.

- What results do you expect from making a change?
- How important are these results to you?
- What are your feelings about the actions involved in making a change?
- How important are these feelings to you?
- Do an experiment and try the change for three days. What do you notice?
- Take a survey. What do people you trust and/or experts on the topic say?
- Imagine the outcomes a day, week, or month into the future. How would this be?
- Imagine your feelings a day, week, or month into the future. How would this be?

- What are the pros of making a change?
- What are the cons of making a change?

Confidence

People make changes when they are confident they have what it takes to make it happen. Boost your confidence.

- Reflect on past successes. Think about your skills and strengths.
- Get feedback from others. Ask them about your past successes, skills, and strengths.
- Engage in positive self-talk. Watch the messages you give yourself; be encouraging.

Set Goal

About

There are 5 steps that help in developing new habits: getting prepared, setting a goal, developing a plan, monitoring behavior, and evaluating performance. Consideration for each step will help you be more effective in making changes.

STEP 2 involves setting a goal. This means deciding what results you want and what you can reasonably do to bring these results about in your life. It also means making a commitment to action. Read more about this…

Outcomes

Outcomes are the **results** we want. Outcomes are things we cannot directly bring about and include things like losing weight, having lower blood pressure, and reducing the frequency of mood episodes. Make sure you are clear about your outcome. Be as specific as possible. For example, you might hope for no depressive episodes in the next 4 months. Or you might want to lose 5 pounds in 2 months.

Targets

Targets are the **efforts** you are going to make. They are the actions involved that will hopefully bring about the desired outcomes. Exercising should help you lose weight, eating less salt should lower your blood pressure, and taking medications should reduce the frequency of mood episodes. Be specific about the efforts you are going to put in place on a daily or weekly basis.

Commitment

Make a commitment to yourself! Telling another person about your intentions make it more likely you will follow through with your plan. So, consider telling a support or your psychiatrist. Or consider finding a “goal buddy” to check in with on your efforts and progress towards your outcome goals.

Develop Plan

About

There are 5 steps that help in developing new habits: getting prepared, setting a goal, developing a plan, monitoring behavior, and evaluating performance. Consideration for each step will help you be more effective in making changes.

STEP 3 involves developing a plan. Being specific about your effort (behavioral targets), making sure you have the resources to succeed, and identifying ways to overcome obstacles are important parts of this process. Read more about this…

Specify Targets

What are you going to do, **when** are you going to do it, and **where** are you going to do it? Remember the acronym ART to develop your plan.

- Be sure the target is based on actions.
- Be sure the goal is realistic.
- Be sure to include a time and place.

Confirm Resources

Be sure you have the skills and internal resources you need in order to succeed.

- Is the target behavior within your skill set? Do you need instruction on how to perform a behavior? Do you need to practice a behavior in advance?
- Developing a habit can help. It is easier to keep up with an established behavior than it is to start a new one. Think about developing a habit before you initiate your plan. Get a head start.
- Mental fatigue compromises your ability to make changes in life. It tires your "mental muscle". How might you conserve your mental resources?
- Stress and negative emotions will work against you. How might you reduce the interference of distress?
- Mental and physical health symptoms will work against you. They interfere with your ability to make wise decisions and follow through in a consistent way. Do you need medication to help?

Be sure you have the supports and external resources you need in order to succeed.

- Do you have social support for making your change? Telling someone about your intentions makes it more likely you will follow through with your plan.
- Consider checking in with your support person or finding a “change buddy”. Ongoing positive feedback helps with sustaining effort.
- Think about your environment. Will the place you have in mind facilitate your target activity? Will the people there facilitate your target activity? Or will it and they distract you?
- Will setting prompts or cues help you remember or help you get started with your target activity?

Identify Ways to Overcome Obstacles

Some potential obstacles, such as mental fatigue and stress, are mentioned above. Uni-tasking during the day helps reduce mental fatigue. (Uni-tasking is focusing on one task at a time, without distraction or interruptions. It is the opposite of multi-tasking.) Picking a time and place for your target when you have not been engaged in lots of prior mental activity can also make success more likely. Likewise, reducing life stress, coping more effectively, or picking a time and place for your target when you generally feel calmest can make success more likely.

What other obstacles can you anticipate? Generate a number of ways to overcome them. Brainstorm. Then pick the best solution. Build these ideas into your plan.

- Create a series of “if-then” statements with each potential obstacle and best solution.

For example, if I am too stressed out to exercise, then I will take 30 minutes to do a guided meditation and then I will exercise.

Monitor Behavior

About

There are 5 steps that help in developing new habits: getting prepared, setting a goal, developing a plan, monitoring behavior, and evaluating performance. Consideration for each step will help you be more effective in making changes.

STEP 4 involves monitoring your behavior. Keeping a log or asking others for feedback are ways to track your behavior. Read more about this…

Monitor Behavior

Monitoring your behavior makes it more likely that you will effectively make changes. There are two ways to monitor your behavior.

- Self-monitoring of behavior. This involves keeping a log or graph of some sort. Post it in your home where you can see it. Or, alternatively, keep a log in your telephone where you can check it daily.
- Feedback on behavior. Feel free to ask others for feedback. A “change buddy”, supports, or your psychiatrist can let you know how they think you are doing.

As you implement your plan remember the THREE A’s:

- **Activate:** Keep your plan in mind. Use a reminder like posting a note or programming an alert, or schedule the activity into your normal routine. Make it a habit.
- **Attend**: Focus on the task at hand. Gently let go of intrusive thoughts, distractions, competing impulses, temptations, and thoughts of old habits. Zoom in!
- **Action:** Getting started is the hardest part. Keep your eye on the prize. Get yourself to the starting line. Get the materials you need. Just start moving.

Evaluate Performance

About

There are 5 steps that help in developing new habits: getting prepared, setting a goal, developing a plan, monitoring behavior, and evaluating performance. Consideration for each step will help you be more effective in making changes.

STEP 5 involves evaluating your performance. Review your targets (planned efforts). Compare what you actually did against what you planned to do. Did you succeed? Do you need to make adjustments? Read more about this…

Review Target Behavior

Remember that targets are the **efforts** (actions) you planned. How did you do? Did you stick with the plan? If you did, give yourself credit for your efforts. Reward yourself! If you did not, don’t worry. And don’t beat yourself up. Making changes always involves a learning process. Make sure you give the plan a couple of weeks. If things still are not going well, determine where things broke down and make adjustments.

For example, if your plan was to take all medications daily, did you do it? If so what helped? If not, review what got in the way.

Make adjustments:

- Was it your commitment to the plan? Revisit your motivation. Redo Step 1: Get Prepared.
- Was it the selection of the target? Revise your goal. Redo Step 2: Set Goal.
- Was it the plan? Revise your plan. Redo Step 3: Make Plan.

Review Outcome Goals

Remember that outcomes are the **results** you anticipated. Did changes in your behavior result in the desired outcomes? For example, did exercise result in losing weight? Or did getting 9 hours of sleep help you feel more rested? If so, give yourself credit for all of your planning and efforts. If not, don’t worry. Make sure you give the plan a couple of weeks. If you are still not getting the results you want make adjustments.

For example, if you took all medications daily did you have fewer symptoms? If so, give yourself credit for a job well done. If not, what do you think happened?

Make adjustments:

- Was it the selection of the target? Do you need to change your target? Redo Step 2: Set Goal.
- Was it the plan? Do you need to change the frequency of the behavior? Redo Step 3: Make Plan.

Self-Assessment

Awareness means knowing what to look for and then looking for it! You can use tools here to build awareness and help you identify your symptoms, early warning signs, and triggers for depression and mania.

Preparedness means identifying skills and supports in advance. You can use the tools here to build your plans for action.

Take a look!

Symptoms and Triggers
Skills and Strengths
Supports and Environment

Symptoms and Triggers

About

Remember that in order to take action and manage your bipolar disorder, you first need to be aware.

Awareness means **knowing** what to look for and then **looking** for it!

The following exercises will help you identify your symptoms, early warning signs, and triggers for depression and mania.

Take a look!

Symptom History

Life Chart

A Life Chart can help make sense of your bipolar disorder. It will help you understand patterns. You can use this information to avert future episodes.

Click below for an example.

<Insert link>

To chart out your lifetime symptoms, take a piece of paper and turn it on its side. Draw a line across the middle. At the left end write “onset” (which means first symptoms). At the right end, write “now”.

What happened at the onset? Was it depression? (It often is.) How have your symptoms changed or evolved over time since they first started?

Make a note of any past or current mood episodes. Episodes of depression are drawn below the baseline and episodes of mania or hypomania are drawn above the baseline. For each episode record your:

- Age
- Use of alcohol and street drugs
- Major life events
- Treatments

Examine your chart. Is there a connection between:

- Substance use and episodes?
- Life events and episodes?
- Medications and episodes?

Show your psychiatrist and your supports your Life Chart. Discuss your ideas and ask if they have anything to add. Often others notice things that you do not notice yourself.

Signs Checklist

Think about what you are like as a person when you are depressed, well, and manic. Consider symptoms. Also consider how you think and act. That is, how you get along in the world.

Click the link below for an example.

<Insert link>

Take a piece of paper and make three columns. Title the left column Depressed, the middle column Well, and the right column Manic.

Then record what you notice about yourself when you are depressed, well, and manic. Include the following:

- Symptoms
- Early warning signs

Also consider…

- Personality characteristics: Are you…Open? Conscientious? Outgoing? Agreeable? Calm?
- Outlook on life: Are you…Optimistic? Pessimistic?
- Self-care: Are you taking care of hygiene, grooming, laundry, housekeeping, bills?
- Social behaviors: Do you seek out others more or less than usual?
- Work performance: Do you think you're doing well? Do others think you're doing well?
- Interests: What is on your mind? What is your focus when depressed, well, and manic? Is it different across mood states?

Show your psychiatrist and your supports your Signs Checklist. Discuss your ideas and ask if they have anything to add. Often others notice things that you do not notice yourself.

Triggers Checklist

Personal and environmental factors can trigger episodes of depression and mania. Think about this. Reflect on your past episodes. What triggered your depression? What about mania? Make a list of these triggers.

Click the link below for an example.

<Insert link>

Also consider the following list of common triggers:

- Discontinuing medications
- Sleep changes
- Disruptions in routines
- Too much involvement in activities
- Being stressed
- Negative life events
- Critical family interactions
- Substance use

Do any of these apply to you? If so, add them to your list.

Show your psychiatrist and your supports your Triggers Checklist. Discuss your ideas and ask if they have anything to add. Often others notice things that you do not notice yourself.

Substance Use Questionnaire

Do you use alcohol and/or street drugs? Do you use prescription drugs in ways other than as prescribed? Do you use prescription drugs that are not prescribed to you?

Answer the following questions. Try to be as honest with yourself as possible.

1. Have you tried to cut down your use but been unsuccessful in doing so? Or been successful but only for a very brief period of time?
2. Have you gotten annoyed with the comments of others about your alcohol and/or drug use?
3. Have you felt guilty at all about your alcohol and/or drug use?

4. Have you ever had an “eye-opener” or alcohol or drugs first thing in the morning to take the edge off?

If you answered “yes” to even one of these questions, you may be drinking and/or using other substances in ways that are affecting your health and well-being. Consider discussing these issues with your psychiatrist.

Beliefs about Medications

Certain beliefs will promote sticking with your medication plan and certain beliefs will impede sticking with your medication plan.

Reasons **to take** medications:

- You believe the medication helps you feel better
- Your relationship with your prescribing doctor influences you.
- Your relationship with your therapist influences you.
- Someone in your family or a friend believes you should take medication.
- You believe taking medication prevents your illness or symptoms from returning.
- You are pressured or forced to take medication.
- You are afraid of being re-hospitalized.

Reasons **not to take** medications:

- You believe medication does not help you feel better.
- Your bad relationship with your prescribing doctor influences you.
- Your bad relationship with your therapist influences you.
- One of your practitioners does not believe you should be taking the medication.
- Someone whose opinion is important to you is against your taking the medication.
- You have difficulty getting to your appointments and/or difficulty getting medications.
- You feel embarrassed about taking medication.
- You don’t have enough money to pay for treatment or medication.
- You would rather take other drugs or alcohol.
- You don’t believe you have a mental illness.
- You don’t believe that you currently need the medication.
- The side effects of the medication are too upsetting to you.
- You feel more comfortable in the hospital.

Do any of these apply to you? Consider discussing your answers with your psychiatrist and supports. See what they have to say about your views on medications.

Skills and Strengths

About

Remember that you need a good plan in order to take action and manage your bipolar disorder. You need to know what to do to get well when you are not well and you need to know what to do to stay well when you are well.

A successful plan is one that best showcases your skills and strengths. While skills are developed abilities, strengths have more to do with your inborn talents.

The following exercises will help you identify your current skills and strengths. You can use this knowledge to build on your current skills and even to build skills in new areas that align well with your personal strengths.

Take a look!

Coping Skills Checklist

How well are you coping? Take a look at some common coping strategies.

For **mania…**

Good coping skills:

- Modify activities and restrain yourself
- See a doctor
- Take time to rest
- Engage in calming activities

Poor coping skills:

- Find more things to do to fill the extra minutes of the day
- Enjoy the feeling of high
- Do nothing about it
- Continue to take on new tasks and do new things

For depression…

Good coping skills:

- Get organized and keep busy
- Get social support and meet people
- Distract from negative thoughts by doing more
- Recognize realistic thoughts/evaluate if things are worth worrying about

Poor coping skills:

- Stay in bed and hope it goes away
- Do nothing about it
- Take extra medication
- Use alcohol or drugs to numb yourself

How are you doing? Do you need to add some good coping skills to your Wellness Plan? Do you need to eliminate some poor coping skills from your life?

Strengths Checklist

Strengths are inherent talents. You can use your strengths to increase your wellness. Participating in activities that rely on your strengths will increase your sense of well-being.

Everyone is different. What are your strengths?

- Love of learning
- Social intelligence
- Creativity
- Curiosity
- Judgment
- Perspective
- Bravery
- Persistence
- Authenticity
- Zest
- Love
- Kindness
- Fairness
- Leadership

- Citizenship
- Teamwork
- Forgiveness
- Humility
- Prudence
- Self-control
- Appreciation of beauty
- Gratitude
- Hope
- Humor
- Spirituality

How might you use your strengths to promote wellness? Can you include them in your Wellness Plan somehow?

Values Checklist

What is important to you in life? You can use your values to promote wellness. Participating in activities that are meaningful and satisfying will increase your sense of well-being.

Everyone is different. What are your values?

- Family relations (not marriage/parenting)
- Marriage/couples/intimate relations
- Parenting
- Friendships/social relations
- Employment
- Education/training
- Recreation
- Spirituality
- Citizenship/community life
- Physical well-being
- Mental well-being

How might you use your values to promote wellness? Can you include them in your Wellness Plan somehow?

Happiness Survey

Happiness means having a sense of satisfaction and meaning in life. It means more than absence of illness.

Happiness has to do with what is going on inside you and how this impacts your relationships with others. Happiness increases resilience, or the ability to withstand stress without getting sick.

There are six major aspects of happiness. Consider your life.

1. **Mastery.** Do you have a sense of mastery? This means making effective use of opportunities. It also means actively shaping situations to suit you.
2. **Personal Growth.** Do you have a sense of personal growth? This means continuing to develop and learn.
3. **Purpose.** Do you have a purpose in life? This means having goals and a sense of direction.
4. **Independence.** Do you feel independent? This means living in accordance to your personal standards and resisting social pressures to think and act in certain ways.
5. **Acceptance.** Do you accept yourself? This means having an overall positive attitude about your own self-worth. It means accepting both the good and the bad without judgment.
6. **Relationships.** Do you have good relationships? This means having warm, satisfying, and trusting relationships with others. It means having people who are concerned about you and for whom you reciprocate those sentiments.

Reflect on your answers. What do you think? Are you happy? Do you need to make any changes in your life to feel happier?

Opinions about Recovery

Certain attitudes promote recovery and wellness. These things help: personal confidence and hope, willingness to ask for help, having a goal and success orientation, and being able to rely on others.

Below is a list of common views on recovery. Do any of these apply to you?

- Fear doesn't stop me from living the way I want to.
- I can handle what happens in my life.
- I like myself.

- If people really knew me, they would like me.
- I have an idea of who I want to become.
- Something good will eventually happen.
- I am hopeful about my future.
- I continue to have new interests.
- I can handle stress.
- I know when to ask for help.
- I am willing to ask for help.
- I ask for help when I need it.
- I have a desire to succeed.
- I have my own plan for how to stay or become well.
- I have goals in life that I want to reach.
- I believe I can meet my current personal goals.
- I have a purpose in life.
- Even when I don't care about myself, other people do.
- I have people I can count on.
- Even when I don't believe in myself, other people do.
- It is important to have a variety of friends.

The more statements you endorsed, the stronger your recovery orientation.

How are you doing? How does your attitude about recovery affect your wellness? Would having a more positive attitude about recovery increase your overall sense of well-being? Do you need to change some negative attitudes?

Supports and Environment

About

The people, places, and things in your life can facilitate or impede wellness.

A good quality of life is associated with wellness. The following factors impact wellness: physical health, mental health, relationships, and your environment.

The following exercises will help you identify the plusses and minuses when it comes to the quality of your life.

Take a look!

Quality of Life Survey

How would you rate your quality of life? Consider the various aspects of your life. Think about the extent to which they suit you at this time.

Physical health…

- To what extent do you feel that physical pain prevents you from doing what you need to do?
- How often do you need medical treatment to function in your daily life?
- Do you have enough energy for everyday life?
- How well are you able to get around?
- How satisfied are you with your sleep?
- How satisfied are you with your ability to perform your daily living activities?
- How satisfied are you with your capacity for work?

Mental health…

- How much do you enjoy life?
- To what extent do you feel your life to be meaningful?
- How well are you able to concentrate?
- Are you able to accept your bodily appearance?
- How satisfied are you with yourself?
- How often do you experience negative emotions such as feeling blue, hopeless, anxious, or depressed?
- How satisfied are you with your personal relationships?
- How satisfied are you with your sex life?
- How satisfied are you with the support you receive from your family members or friends?

Environment…

- How safe do you feel in your daily life?
- How healthy is your physical environment?
- Do you have enough money to meet your needs?
- How readily available is information that you need for daily living?
- To what extent do you have opportunities to participate in leisure activities?
- How satisfied are you with your living arrangements?
- How satisfied are you with your access to health services?
- How satisfied are you with your transport?

Are there any areas of your life that you want to improve? Consider what changes you would need to make in order to enhance your quality of life.

Social Support Survey

Get out a piece of paper and pencil. For each item below write down two things. First, list all the people in your life who fit in each of the categories. Second, rate how satisfied you are with each person listed.

- Who can you count on to be dependable when you need help?
- Who can you count on to help you feel more relaxed when you are under pressure or tense?
- Who accepts you the way you are, with your strengths and weaknesses?
- Who can you count on to care about you, regardless of the situation?
- Who can you count on to help you feel better when you are feeling generally down-in-the-dumps?
- Who can you count on to console you when you are upset?

Do you have enough supports in your life? Are you satisfied with the supports in your life? Or do you need to work on further developing your support network?

Lifestyle

There are lifestyle choices that can increase your risk of experiencing symptoms. There are also lifestyle choices that can decrease your risk of experiencing symptoms.

You can learn about specific lifestyle skills here. Practice these skills to reduce risk.

Take a look!

Sleep

Medications

Attend

Routine

Tranquil

Social

Sleep

About

Sleep is vital to well-being. Sleep impacts our attention, concentration, memory, motor skills, emotional health, and physical health.

Sleep is especially important in managing bipolar disorder. Changes in the amount, as well as in the timing, of sleep can cause mood episodes.

Getting anywhere from 6 to 10 hours per night is normal. However, people vary. How much sleep do you need in order to feel rested? How much sleep do you need in order to feel well and function well?

Three Day Sleep Challenge

Is your sleep off track? Get on a good schedule. The method is simple, but the first few days can be quite challenging. Try this method:

- Lie down and get up the same time every day
- Go to bed only when sleepy
- Use the bed for sleep and sex only
- If you can't fall asleep after 15 to 20 minutes, get out of bed
- Avoid taking naps

Check your wellness ratings and see if they change after three days of following this method. Then check again after a few weeks. Do you notice any patterns between your sleep and wellness ratings?

Digital and Diet "Detox"

The most common causes of insomnia are substances (including caffeine), medical conditions, mood disorders, stress, poor sleep habits, and poor sleep conditions. Cleanse your body of substances for a good night's rest.

Check your wellness ratings and see if they change after a few days.

- Abstain from substances
- Use skills to reduce symptoms
- Use skills to reduce stress

Worry Buster 1

Stress and anxiety can cause insomnia. If this is true for you, try using a worry pad.

Get a pad of paper and put it next to your bed. Each night before you go to sleep, write down any worries you may have. Now you don't have to think about them! They are on a piece of paper. If you must, you can resume thinking about them the next day when you wake up.

Nighttime is for rest. Daytime is for thinking.

Worry Buster 2

Stress and anxiety can cause insomnia. If this is true for you, try using a worry chair.

If you MUST worry, then set a time and place to do it. Designate a specific chair as your worry chair. Use it during the day as you must. Worrying is not allowed in bed.

If you find yourself worrying in bed, gently set the thought aside. This includes worries about life. It also includes worries about not sleeping!

Guided Imagery

Use imagery to calm your mind and body so that you can rest.

Engage all senses:

- What does it look like?
- What does it sound like?
- What does it feel like?
- How does it smell?
- How does it taste?

If worries or negative thoughts interrupt your imagination, gently set them aside and return to your relaxing image.

Sleep Diary

Good sleep promotes health. It also reduces your vulnerability to depression and mania. Try keeping a sleep diary to better track your sleep.

Click the link below for an example:

<Insert Link>

Week of: February 16, 2014								
SLEEP PLAN:<br>Get up and go to bed the same time daily: My sleep schedule will be 10:00 p.m. to 7:00 a.m.<br>Do not use the bed for activities other than sleep and sex: I will read on the sofa in the evenings instead of in bed.<br>Avoid taking naps during the day: I will get up, go outside, and take a walk if I get drowsy in the afternoon.								
**Success Rating**	**Wellness Rating**	S	M	T	W	R	F	S
Full	0	•	•	•	•	•	•	•
	1	•	•	•	•	•	•	•
Some	2	•	•	•	•	•	•	•
	3	•	•	•	•	•	•	•
Little	4	•	•	•	•	•	•	•
Do you notice any relationship between activity and wellness?<br>My sleep got worse AFTER my wellness rating dropped.<br>Did you stick to your plan? If not, do you need an easier goal? Do you need to plan for obstacles?<br>I stayed up really late on Wednesday night. I got excited about a computer project idea and didn't stop working.								

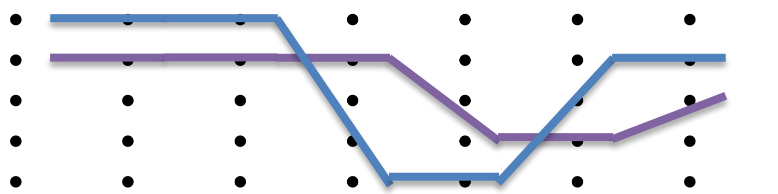

Part of this exercise includes creating a sleep plan. Good sleep means getting the right **amount.** How much do you need? It also means having the right **timing.** Keep regular.

Record how well you follow your plan. Do you stick to it fully, some, only a little…or somewhere in between? Also record your wellness ratings for that week. Are you balanced, severely up or down…or somewhere in between?

See how your sleep relates to your moods.

What did you learn from this exercise? Do you want to refine your relapse prevention plan? Or do you need…

- To continue trying?
- To make more reasonable goals?
- To have a sleep evaluation?

Medication

About

Medication is key to well-being for individuals with bipolar disorder. It reduces the risk of symptoms and relapses better than anything else that has been studied so far.

Making a commitment to medication can be difficult. At times you may think it is unnecessary, you may run into problems with it, you may feel that it seems to conflict with other goals you have, or other people in your life may discourage you about medications.

Remember, it is *your* health!

Following medical advice is hard. Only about 50% of people take medications as prescribed. This is true for all sorts of conditions including diabetes and hypertension. It is also true for bipolar disorder.

Not taking medications as prescribed, however, can result in relapses of mania and depression, hospitalization, and longer hospital stays.

Be wise! Consider these skills.

Educate

Educate yourself! Do you hold any of the following beliefs? I do not need medications because…

- I don't have an illness
- My symptoms are gone
- I can manage on my own
- Medications don't work for me

Read! Make sure you understand the nature of bipolar disorder. **Reflect**! Make sure you understand your bipolar disorder. People often think they are fine when they are manic. Trust your family and doctor to be honest with you.

Solve

Solve problems! Do you have any of the following problems? In terms of medications I struggle with…

- Side effects
- Fear of side effects
- Forgetfulness
- Expense
- Inconvenience
- Embarrassment

These are problems that can be solved. Talk to your psychiatrist. Get a pillbox or set an alarm/reminder, budget for your medications, work on self-acceptance.

Resolve

Resolve conflicts! Do you have any of the following conflicts? I don't want to take medications because…

- I want to drink or use drugs.
- I want to feel like myself.
- I want to feel up.
- I want to be productive.

Talk to your psychiatrist about all of these things. You need to be safe. You need to feel like yourself. A combination of the right medications and skills will get you there.

Support

Get support! Do you experience any of the following challenges? I don't want to take medications because…

- I don't like my psychiatrist.
- I don't like my therapist.
- My family is against medications.
- My friends are against medications.

Try to work things out with your current providers or find new ones. Get your family and friends informed or reach out to others who will support you.

Attend

About

There is a lot of evidence that mental and physical health is inter-related. Mental health impacts physical health, and physical health impacts mental health. You probably already know this. It is intuitive.

Pay attention to your diet, exercise, and use of substances. That is, what you put into your body and what you do with your body. Having a good diet, being active, and minimizing use of substances will help you feel well. It will also help keep symptoms of bipolar disorder at bay.

Thirty Day Substance Trial

Nourish your body and brain! Avoid ingesting substances that are toxic to your body.

Check your wellness ratings. See if they change after 1, 2, 3, and 4 weeks of not using substances that might be causing you problems. Maintain a healthy diet that consists of:

- Vegetables
- Fruits
- Grains
- Dairy
- Protein
- Water

Without toxins in your system, your brain and body both work better. See what you think! If you are using substances to manage your alertness, energy, mood, emotions, or medication side effects see if there aren't other ways to cope.

Note: If you are addicted to substances you will need special treatment to stop. Complete the "Alcohol and Drugs Worksheet" in the Self-Assessment section if you have questions about whether or not you are addicted to substances.

People, Places, and Things

Nourish your body and brain! This will require willpower. It also requires reducing temptation.

Stay away from:

- People that use substances
- Places where substances are used
- Things associated with substance use

Check your wellness ratings and see if they change after a few days and a few weeks of staying away.

Note: If you are addicted to substances you will need special treatment to stop. Complete the "Alcohol and Drugs Worksheet" in the Self-Assessment section if you have questions about whether or not you are addicted to substances.

HALT

Nourish your body and brain! You may not know it, but stress taxes your ability to make good decisions. It compromises your ability to exert willpower. So in order to stick to avoiding substances, try the following:

Avoid being too:

- **H**ungry
- **A**ngry
- **L**onely
- **T**ired

Check your wellness ratings and see if they change after a few days and a few weeks of trying this.
Note: If you are addicted to substances you will need special treatment to stop. Complete the "Alcohol and Drugs Worksheet" in the Self-Assessment section if you have questions about whether or not you are addicted to substances.

Avoid Temptation

Nourish your body and brain! This will require willpower. It also requires avoiding temptation.

Keeping yourself busy can make it easier to resist temptation. Plan your time! Here are some ideas:

- Socialize with family/friends
- Volunteer/community involvement
- Exercise/yoga/meditation
- Recreation/hobbies

- Church/temple

Check your wellness ratings and see if they change after a few days and a few weeks.

Note: If you are addicted to substances you will need special treatment to stop. Complete the "Alcohol and Drugs Worksheet" in the Self-Assessment section if you have questions about whether or not you are addicted to substances.

Deal With Urges

Nourish your body and brain! Avoid ingesting substances that are toxic.

Plan for urges! Here are some ideas:

- Surf the urge: notice it and let it pass
- Fight the urge: imagine wiping it out
- Call someone: family, friend, sponsor

Identify the trigger for your urge:

- Stress
- Symptoms
- Side effects
- Something else?

Find ways to cope with the trigger that don't involve alcohol or drugs.

Check your wellness ratings and see if they change after a few days and a few weeks.

Note: If you are addicted to substances you will need special treatment to stop. Complete the "Alcohol and Drugs Worksheet" in the Self-Assessment section if you have questions about whether or not you are addicted to substances.

Sobriety Log

Keep substance use to a minimum. It promotes health. It reduces your vulnerability to depression and mania. Try keeping a substance diary.

Click the link below for an example:

<Insert link>

<table>
<tr><td colspan="9">Week of: February 16, 2014</td></tr>
<tr><td colspan="9"><u>SOBRIETY PLAN</u>:
Nicotine:
Caffeine: Will stick to two cups of coffee before noon.
Alcohol: Will reduce intake to maximum of 1 drink per night.
Street drugs:
Misuse of prescription drugs:</td></tr>
<tr><td>Success Rating</td><td>Wellness Rating</td><td>S</td><td>M</td><td>T</td><td>W</td><td>R</td><td>F</td><td>S</td></tr>
<tr><td>Full</td><td>0</td><td colspan="7" rowspan="5">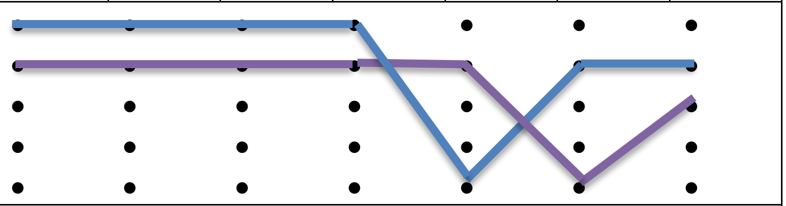</td></tr>
<tr><td></td><td>1</td></tr>
<tr><td>Some</td><td>2</td></tr>
<tr><td></td><td>3</td></tr>
<tr><td>Little</td><td>4</td></tr>
<tr><td colspan="9">Do you notice any relationship between substance use and wellness?
My wellness dropped to severely depressed (-4) after a day of heavy drinking.
Did you stick to your plan? If not, do you need an easier goal? Do you need to plan for obstacles?
I did great for 4 days, okay for 2 and terribly for one. I drank when I saw Roger. He's a big drinker. Probably need to stay away from him.</td></tr>
</table>

Write out your sobriety plan. Include use of nicotine, caffeine, alcohol, street drugs, and misuse of prescription drugs.

Record how well you follow your plan. Do you stick to it fully, some, a little bit…or something in between? Also record your wellness ratings for that week. Are you balanced, severely up or down…or somewhere in between?

See how your substance use relates to your moods.

What did you learn from this exercise? Do you want to refine your relapse prevention plan?

Do you need…

- To continue trying?
- To make more reasonable goals?
- To enroll in substance use treatment?

Nutrition Challenge

Eat well! The United States Food and Drug Administration (USFDA) suggests eating a range of “nutrient dense” foods. To maintain the integrity of the foods, prepare them with little or no added solid fats, sugars, refined starches, and sodium.

- A variety of vegetables: dark green, red and orange, beans and peas, starchy, and other vegetables
- Fruits, especially whole fruits
- Grains, at least half of which are whole grains
- Fat-free or low-fat dairy, including milk, yogurt, cheese, and fortified soy beverages
- A variety of protein foods, including seafood, lean meats and poultry, eggs, beans and peas, soy products, and nuts and seeds
- Oils, including those from plants and oils that are naturally present in foods

How healthy is your diet? Here are some things you can try to improve your nutrition.

- Keep a food diary for a week. See how it measures up to the “nutrient dense” diet recommended by the USFDA.
- Pick out one small thing to do each day to improve your diet. Start easy and see how it goes!
- Make a commitment to healthy eating for 3 straight days. See how you feel at the end of the 3 days.
- Find a “change buddy”. Make a plan together to improve your nutrition. Pick something to change and check in with them daily.

Creating new habits can be challenging. See the Making Changes section in the *LiveWell* Toolbox for tips about how to get motivated, make change plans, and implement plans in a way that maximizes your chances of success.

Exercise Challenge

Get fit! The USFDA also has physical exercise guidelines. For adults:

- At least 2.5 hours of moderate intensity physical activity every week
- Muscle strengthening exercises on 2 or more days each week

How active are you? Here are some things you can try to improve your fitness.

- Keep an exercise diary for a week. See how it measures up to the guidelines recommended by the USFDA.

- Pick out one small thing to do each day to improve your fitness. Start easy and see how it goes!
- Make a commitment to exercise for 3 straight days. See how you feel at the end of the 3 days.
- Find a “change buddy”. Make a plan together to improve your fitness. Pick something to change and check in with them daily.

Creating new habits can be challenging. See the Making Changes section in the *LiveWell* Toolbox for tips about how to get motivated, make change plans, and implement plans in a way that maximizes your chances of success.

Substance Challenge

Stay clean! Psychoactive substances are things individuals ingest that impact their mental state. This includes things such as nicotine, caffeine, alcohol, cannabis and other illegal drugs. These substances impact the way your brain functions. In moderate to large amounts, they are basically toxic. Overuse or misuse causes mental, physical and social problems. Even small amounts can interact with medications prescribed for bipolar disorder.

In terms of alcohol, the most common problematic substance for most individuals, the National Institutes of Health recommends:

- No more than 4 drinks/day and no more than 14 drinks/week for men
- No more than 3 drinks/day and no more than 7 drinks/week for women

Higher amounts are considered risky drinking and put people at risk for physical and mental health complications.

How clean are you? Here are some things you can try to improve your conditioning.

- Keep a substance diary for a week. See how it measures up to the guidelines recommended by the USFDA for alcohol. Make note of other things as well, like nicotine, caffeine, and drugs.
- Pick out one small thing to do each day to improve your conditioning. Start easy and see how it goes!
- Make a commitment to reduce a substance for 3 straight days. See how you feel at the end of the 3 days.
- Find a “change buddy”. Make a plan together to improve your conditioning. Pick something to change and check in with them daily.

Creating new habits can be challenging. See the Making Changes section in the *LiveWell* Toolbox for tips about how to get motivated, make change plans, and implement plans in a way that maximizes your chances of success.

Routine

About

A blueprint is a design for a building. Thinking ahead is necessary. Can you imagine constructing a house with no plan? Can you imagine just randomly putting bricks together? It would be chaos. The house would probably fall apart.

Like a well-built house you also need a well-built life. You need a design for your life. You need a daily schedule. You need structure in order to thrive.

There are key aspects to this structure in life: living, loving, learning, working, and playing.

MAP It

Healthy routines build esteem and joy. Make plans for a sense of **m**astery. Make plans for a sense of **pl**easure.

Activities include sleeping, eating, exercising, grooming, chores, childcare, socializing, working, school, and leisure.

What is your schedule? MAP it out!

- Wake-up time and routine
- Morning activities
- Afternoon activities
- Evening activities
- Bedtime and routine

Values

Healthy routines build on core values.
What is your schedule? Honor your values!

- Family relations
- Marriage/couples/intimate relations
- Parenting
- Friendships/social relations
- Employment
- Education/training

- Recreation
- Spirituality
- Citizenship/community life
- Physical well-being

Recovery

Sometimes episodes of mania or depression derail your plans and goals. Healthy routines support recovery.

There are six keys to recovery from problems due to bipolar disorder. Think about your own life. What do you want?
Focus on recovery in your schedule!

- Environment
- Treatment
- Support
- Hope
- Skills
- Strengths

Think about how to incorporate these keys to recovery into your regular routines.

Activity Log

Keeping a regular routine will promote wellness for you. This means that you should have some regularity to your day. Be sure to balance activity and rest, responsibilities and leisure.

Click the link below for an example:

<Insert link>

Week of: February 16, 2014
<u>ACTIVITY PLAN</u>:<br>Wake-up: Get up at 6:00 a.m. Have a cup of coffee. Do yoga. Eat a healthy breakfast.<br>Morning: Start work at 9:00 a.m.<br>Afternoon: Stay on task at work. Get things done at a moderate, healthy pace.<br>Evening: Cook a nice meal. Watch television or visit with a friend. Not too much, not too little.<br>Wind-down: Have a warm cup of milk. Watch the evening news. Get in bed by 10:00 p.m.

Success Rating	Wellness Rating	S	M	T	W	R	F	S
Full	0	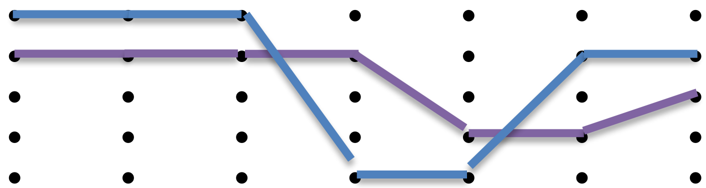						
	1							
Some	2							
	3							
Little	4							

Do you notice any relationship between activity and wellness?
My wellness dropped to depressed (-3) after a day of little activity.
Did you stick to your plan? If not, do you need an easier goal? Do you need to plan for obstacles?
I feel off my plan on Wednesday following an argument with my mother. Next time I'm upset I'll use calming skills so I can stay on track with my activities.

Write out your activity plan. Include things like sleeping, eating, grooming, exercising, chores, socializing, childcare, working, and leisure.

Record how well you follow your plan. Do you stick to it fully, some, a little bit…or something in between? Also record your wellness ratings for that week. Are you balanced, severely up or down…or somewhere in between?

See how your routines relate to your moods.

What did you learn from this exercise? Do you want to refine your relapse prevention plan?

- Do you need a different schedule?
- Do you need different activities?
- Do you need therapy?

Tranquil

About

Stress is part of life. However, intense or prolonged stress can cause many physical and mental health problems. Stress can trigger symptoms.

Problem-focused coping means coping with problems. It means approaching them and solving them if you can. Solvable problems ought not be avoided.

Emotion-focused coping means regulating your feelings. It means allowing them and being gentle. Always tend to your feelings. Console yourself.

Problem Solving

Avoiding problems causes more problems. For things that can be changed…act!

1. Identify the problem. Describe it in detail.
2. Select your goal. Describe what you would like to see happen.
3. Generate alternative solutions. Come up with several plans.
4. Evaluate the alternatives. Which are practical? Which will work best?
5. Implement your plan. Decide on a time and place. Do it!
6. Evaluate the results. Did it work? If not, should you do something else?

Bird's Eye View

When upset, it is easy to get lost in our thoughts and feelings. Take a step back…

- **Summarize** your state of mind. What is the theme of your thoughts and feelings?
- **Understand** your state of mind. What event triggered your thoughts and feelings?

Do not judge. Do not act. Just summarize and understand your thoughts and feelings.

Deep Breathing

Your diaphragm is the muscle that controls breathing. It is located just below your lungs. Using your diaphragm to its fullest is calming.

Breathe by using your belly rather than your chest. This is the best way.

Take slow, deep belly breaths.

Variations:

- Count to three as you breathe in and count to three as you breathe out.
- Think as you breathe in and let go as you breathe out.

Muscle Relaxation

Reducing tension in your body is calming.

Tense 30 seconds, relax 60 seconds.

Do this for each set of muscles:
- Face
- Neck
- Arms
- Hands
- Shoulders
- Torso
- Legs
- Feet

Pay attention to how your muscles feel when relaxed.

Self-Soothe

Use your senses to calm…

- Sight. View or imagine something. Trees and water often work.
- Hearing. Peaceful music or silence often works.
- Taste. Tea and soups often work. Chocolate in moderation can be nice.
- Smell. Candles and flowers often work.
- Touch. Wrapping up in a warm blanket and taking a bath often work.

Lavish yourself with tender loving care.

Improving The Moment

Take charge of your experience! No matter what is going on you can improve your experience in the moment. Answer one question…

What can I do to be more comfortable right now? Be creative!

- What can you think about to improve your experience in this moment?
- What can you do to improve your experience in this moment?

Mindfulness

Mindfulness means being fully in the moment.

You can turn your mind inward and **notice** all you are thinking, feeling, doing, and experiencing physically. This involves observing these things without judgment.

You can turn your mind outward and **participate** by fully immersing yourself in a situation or activity.

Practicing mindfulness builds gray matter in your brain associated with thinking, memory, emotion regulation, introspection, and self-assessment. Yoga and meditation can help.

Radical Acceptance

Fighting reality causes suffering. For things that cannot be changed…accept.

- Acknowledge what is in yourself
- Acknowledge what is in others
- Acknowledge what is in life

Accepting something is not the same as liking something. Accepting something is not the same as judging something.
Remember that nothing and no one is perfect. Acknowledge the good and bad.

Stress Log

Life events and daily hassles cause stress. Good stress is called “eustress”. Bad stress called “distress”. Intense or prolonged stress causes health problems. It also can trigger symptoms. Try keeping a stress log.

Click the link below for an example:

<Insert link>

<table>
<tr><td colspan="9">Week of: February 16, 2014</td></tr>
<tr><td colspan="9"><u>COPING PLAN</u>:
Problem-focused coping: Will use problem solving skills. Will not procrastinate, will approach problems as they occur.
Emotion-focused coping: Will be mindful of my level of tension and use deep breathing and self-soothing to regulate.</td></tr>
<tr><td>Success Rating</td><td>Wellness Rating</td><td>S</td><td>M</td><td>T</td><td>W</td><td>R</td><td>F</td><td>S</td></tr>
<tr><td>Full</td><td>0</td><td colspan="7" rowspan="5">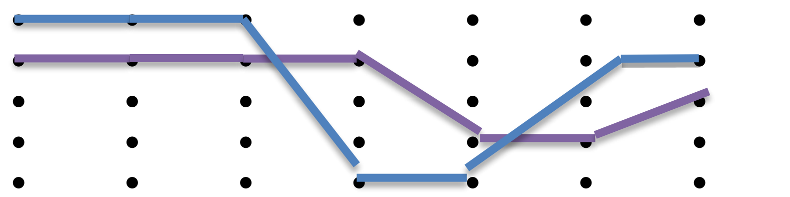</td></tr>
<tr><td></td><td>1</td></tr>
<tr><td>Some</td><td>2</td></tr>
<tr><td></td><td>3</td></tr>
<tr><td>Little</td><td>4</td></tr>
<tr><td colspan="9">Do you notice any relationship between and wellness?
My wellness dropped to depressed (-3) after two days of not actively coping.
Did you stick to your plan? If not, do you need an easier goal? Do you need to plan for obstacles?
I feel off my plan on Wednesday. I had an argument at work and didn’t feeling like dealing with it. I need to recommit myself to this plan.</td></tr>
</table>

Make a coping plan. Again, problem-focused coping means coping with problems. It means approaching them and solving them if you can. Emotion-focused coping means regulating your feelings. It means allowing them and being gentle.

Record how well you follow your plan. Do you stick to it fully, some, a little bit…or something in between? Record your wellness ratings for that week.

See how your ability to cope relates to your moods.

What did you learn from this exercise? Do you want to refine your relapse prevention plan?

Do you need…

- To continue trying?
- To make more reasonable goals?
- A therapist to help with coping skills?

Social

About

Feeling connected to others is vital to health. Social support is vital to health.

Social problems can trigger symptoms. And symptoms can cause social problems. The withdrawal in depression can push others away. The irritability in mania can push others away.

Work to have good relationships with others. Here are some basic skills to help you strengthen and improve your relationships.

Reflection

Reflection is the ability to picture states of mind. Use it to develop a realistic theory about why you think, feel, and act as you do. Use it to develop a realistic theory about why others think, feel, and act as they do.

Reflection allows us to understand ourselves better. It allows us to understand others better. It helps us to manage our feelings. It helps us to respond to the feelings of others.

Work to reflect!

Mutuality

Healthy relationships require give and take. They require having more positive than negative encounters. Be sure to keep a good balance.

- Care
- Listen
- Talk
- Negotiate
- Compromise
- Problem solve

Work for balance!

Boundaries

Boundaries are what separate us from the world around us. We have physical and emotional boundaries. Boundaries develop based on our nature, past experiences, and social norms.

When someone crosses the line, we are uncomfortable. When we cross the line, others are uncomfortable.

Protect your boundaries. And respect the boundaries of others. This involves communication since boundaries vary from person to person.

Active Listening

- Pay attention and show it (nod, make eye contact, sit forward)
- Be encouraging with small gestures (“ok”, “mmm”)
- Ask questions to show interest and to learn more
- Paraphrase their points to show that you’re listening and understand what they’re saying
- Reflect their feelings to show you hear and got it right

Don’t be defensive! No whining, denying responsibility, assuming, or complaining.

Respectful Messaging

Be **courteous** and **direct** when you ask for favors, disagree, express positive feelings, express negative feelings, refuse requests, respond to disagreements, respond to positive feelings, and respond to negative feelings.

- Keep it simple
- Keep it in the here and now
- Use “I” statements
- Voice your preferences

Conflict Resolution

- Use skills to reduce your own physiological agitation. Your feelings are your responsibility. Engage with others only when you are calm enough to do so. Take a time-limited break if need be.
- Use active listening skills. Understanding is harder than judging or arguing. Listen carefully before responding during a conflict
- Use respectful messaging skills. Others listen better when we talk rather than yell. Keep your voice calm and steady.
- Use problem solving skills together. You're on the same team, so whenever possible, come up with a solution together.

Socialization Log

Staying connected to others promotes wellness. This means that you should have some positive contact every day. Be sure to balance having fun, talking, giving support, and receiving support.

Click the link below for an example:

<Insert link>

Week of: February 16, 2014								
SOCIAL PLAN:<br>Activities: Will get out of the house and be around others in some way every day. Will go to the movies with Joyce this weekend.<br>Talking: Will call, text, email, or message at least one person every day.<br>Giving support: Will extend myself to at least one family member, friend, or coworker every day.<br>Receiving support: Will let someone know how I am doing every day—either my husband, therapist, mother, or best friend.								
**Success Rating**	**Wellness Rating**	S	M	T	W	R	F	S
Full	0	•	•	•	•	•	•	•
	1	•	•	•	•	•	•	•
Some	2	•	•	•	•	•	•	•
	3	•	•	•	•	•	•	•
Little	4	•	•	•	•	•	•	•
Do you notice any relationship between social activity and wellness?<br>I became more well (less depressed) as I got more involved with others.<br>Did you stick to your plan? If not, do you need an easier goal? Do you need to plan for obstacles?<br>I did stick to my plan. I kept it simple.								

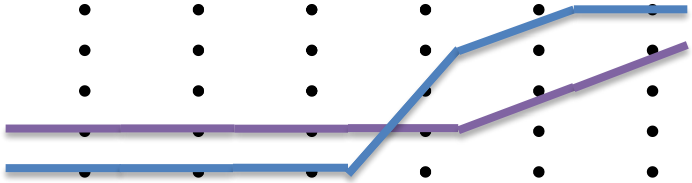

Write out your social plan. Include activities you enjoy, ways you will talk with others, how you will give, and how you will receive.

Record how well you follow your plan. Do you stick to it fully, some, a little bit…or something in between? Also record your wellness ratings for that week. Are you balanced, severely up or down…or somewhere in between?

See how your social contact relates to your moods.

What did you learn from this exercise? Do you want to refine your relapse prevention plan?

Do you need…

- To continue trying?
- To make more reasonable goals?
- A therapist to help with social skills?

Coping Skills

About

You can use coping skills to manage early warning signs and early symptoms. This reduces your chances of having a full-blown episode of mania or depression.

You can learn about specific coping skills here. Practice the skills you like for managing early warning signs and symptoms.

Depression- Dial Up

Mania- Dial Down

Dial up Depression

About

Depression is a spiral down. Many people get withdrawn and less active. These behaviors make depression worse.

Dial Up to avoid depression. Get busy, get moving, and get engaged. Take action-opposite-your-impulse.

Dial Up skills use a method called **behavioral activation.** You can improve your state of mind by becoming more active. You can return yourself to the zone of health.

Graded Activity

Getting active helps counteract depression. It is also very difficult to get going when depressed!

Make a list of things you need to do for the week.

Rank order the items from easy to hard.

Now pick the easiest task and put that on your calendar for today.

Get yourself in position to act and start moving at a gentle pace.

Tomorrow do the same thing.

Pick the next easiest thing on the list.

And so on…

Start low and go slow!

Graded Mastery

Accomplishments repair mild drops in mood. Start **easy** and slow. But go!

- Eat a healthy meal
- Exercise for any length of time

- Take a shower and dress nicely
- Finish a chore around the house
- Finish a task for school
- Finish a task at work

Make a checklist of things that make you feel capable. Start by doing one easy thing. Then do another.

Start easy. Go slow.

Graded Pleasure

Having fun repairs mild drops in mood. Start **easy** and slow. But go!

- Take a bath
- Watch a comedy
- Spend time with your pet
- Have a cup of tea
- Get a massage
- Play a game of chess

Make a checklist of things that you enjoy doing when well. Start by doing one easy thing. Then do another.

Start simple. Go slow.

Activate Your Body

Moving your body repairs mild drops in mood. Start **easy** and slow. But go!

Move…

- Stretch your body
- Lift some weights
- Go to a yoga class
- Go for a short walk

Start with something manageable, maybe just 5 to 15 minutes. Gradually increase the time you move each hour or each day.

No judgment. Ground Yourself.

Activate Your Senses

Minding your senses repairs mild drops in mood. Start **easy** and slow. But go!

Create a sense experience…

- Touch a piece of fabric
- Drink your favorite tea
- Look at an interesting piece of art
- Take in an invigorating scent
- Listen to upbeat music

Touch. Taste. See. Smell. Hear. Really take it in. Allow the experience to unfold, moment by moment.

Be in the moment. Get grounded.

Activate Your Mind

Using your imagination repairs mild drops in mood. Start **easy** and slow. But go! Just think of these things in your mind. Or better yet, draw them out.

Create an upbeat image…

- Doing something important to you
- Doing something you are good at
- Listing your strengths
- Being somewhere energizing
- Being somewhere fun

Create a vivid picture. How does it feel?

You can do it. Ground Yourself.

Increase Contacts

Contact with others repairs mild drops in mood. Start **easy** and slow. But go!

- Call a friend
- Email a family member
- Visit a neighbor
- Sit in the park
- Go to a support group
- Go to a coffee shop

Make a list of simple ways to connect with others. Nothing too challenging.

Start small. Be present.

Increase Conversations

Contact with others repairs mild drops in mood. Start **easy** and slow. But go!

- Initiate a conversation
- Ask questions
- Actively listen
- Share something about yourself
- Talk about the news

Make a list of things to tell others that are simple. Be sure to participate. Be active, not passive. Nothing too challenging.

Start small. Be present.

Increase Offers

Giving to others repairs mild drops in mood. Start **easy** and slow. But go!

- Offer help to someone
- Offer support to someone
- Respond to voicemails and emails

Make a list of ways to give to and receive from others that are simple. Nothing too challenging.

Start small. Be present.

DIY UP

We all have things we do to pick ourselves up. You do as well. Perhaps you have not used them to manage your symptoms, but you can!

Click the link below for an example:

<Insert link>

HEALTHY THINGS I HAVE DONE IN THE PAST TO PICK (DIAL) MYSELF UP:
My strengths: Good sense of humor, hopeful, creative
Things I am good at: Running, piano, cooking, writing poetry
Things I enjoy: Cooking, visiting with friends, movies, playing piano, writing poetry
Things that are stimulating: Playing tennis, going for a run, listening to pop music, watching sit-coms
Things I say to myself: You're going to be okay. You're going to get through this. I am okay.

Think about times when you have been slightly or mildly down in the past. What things did you do that picked you up? What has worked for you?

Consider…

- Your strengths
- Things you are good at
- Things you enjoy
- Things that are stimulating
- Things you say to yourself

What did you learn from this exercise? Do you want to refine your relapse prevention plan?

Do you want to add additional coping skills to your action plan for managing mild symptoms of depression?

Dial down Mania

About

Mania is a spiral up. Most people get more engaged with others and more active. These behaviors worsen mania.

Dial Down to avoid mania. Slow down, do less, and disengage. Take action-oppposite-your-impulse.

Dial Down skills use a method called **behavioral deactivation**. By becoming less active you can improve your state of mind. You can return yourself to the zone of health.

PACED Goals

Slowing your body repairs mild ups in mood. PACE yourself. Slow down!

Not everything is urgent. Set goals so that you don't overdo it:

- **P**riority: Stick to high priority tasks. Everything cannot be important.
- **A**ttitude: Stay mindful and grounded. Not all ideas are great in the long run.
- **C**ommonsensical: Make sure the tasks fit into your life plan.
- **E**ven: Keep a balance of activity and rest. Go slower than you are inclined.

Be thoughtful. Go slow.

PACED Mastery

Mania can cause you to think your ideas are fantastic. That is not to say that you don't have good ideas at times. But, when your mood is too elevated, you need to be careful. Mania tricks you into thinking in exaggerated ways. It is important to be cautious. Slow down!

When up and excited about an idea:

- Wait twenty four hours and then reassess
- Ask two people what they think

Be wise. Go slow.

PACED Pleasure

If you are like others, you may have a tendency to engage in risky activities when your mood is up. Risky actions include things like spending lots of money or being promiscuous.

Are there things you tend to do when manic that you end up regretting later on? If so, build some safeguards for when your mood starts to elevate. For example:

- Give your credit cards to someone
- Stay with a friend and don’t go out
- Remove alcohol from your home
- Close your Facebook account

Be safe. Slow down.

Deactivate Your Body

Slowing your body repairs mild ups in mood. Put on the brakes. Get your body to be quiet on the inside. Be still.

Relax…

- Take deep breaths
- Stretch your body
- Try muscle relaxation
- Do yoga at home

Try to relax for a full day. It will be challenging at first, but you can do it.

Calm your body. Ground yourself.

Deactivate Your Senses

Minding your senses repairs mild ups in mood. Start easy and slow. But go!

Create a sense experience…

- Touch a piece of fabric

- Drink your favorite tea
- Look at a calming piece of art
- Take in an soothing scent
- Listen to slow music

Touch. Taste. See. Smell. Hear. Don't do anything too stimulating. Allow the experience to unfold, moment by moment.

Be present. Get grounded.

Deactivate Your Mind

Using your imagination repairs mild ups in mood. Start easy and slow. But go! Just think of these things in your mind. Or better yet, draw them out.

Create a soothing image…

- Doing something relaxing
- Doing something slowly
- Listing your values
- Being somewhere calming
- Being somewhere laid back

Create a vivid picture. How does it feel?

You can do it. Ground yourself.

Decrease Contacts

Mania makes most people more sociable. It makes some more sexual as well. This can be bad if you don't respect boundaries or push your ideas on others.

Manage mild elevations in mood by removing yourself from others. Start with 24 hours. Dampen your social and sexual intensity.

Retreat a bit. Slow Down.

Decrease Conversations

Positive contact repairs mild ups in mood. Do not overdo it. Take it easy!

DO:

- Let others initiate conversations
- Actively listen

DON'T:

- Cut others off
- Force others to agree

Make a list of things to tell others that are simple. Be sure to participate. Be more passive, less active. Get quiet.

Retreat a bit. Go slow.

Decrease Offers

Giving is an important part of relating. Do not overdo it. Slow down.

- Wait for others to contact you
- Wait for others to ask for help
- Wait for others to ask for support
- Wait for others to call or email you

Make a list of ways to give to others that are simple. Be more passive, less active. Get quiet on the inside.

Retreat a bit. Go slow.

DIY Down

We all have things we do to cool ourselves down. You do as well. Perhaps you have not used them to manage your symptoms, but you can!

Click the link below for an example:

<Insert link>

HEALTHY THINGS I HAVE DONE IN THE PAST TO COOL (DIAL) MYSELF UP:
My strengths: Good sense of humor, hopeful, creative
Things I am good at: Running, piano, cooking, writing poetry
Things I enjoy: Cooking, visiting with friends, movies, playing piano, writing poetry
Things that are calming: Taking a bath, listening to classical music, meditating
Things I say to myself: Easy does it. Slow down. Pace for peace. Walk slowly. Talk slowly.

Think about times when you have been slightly or mildly up in the past. What did you do to slow yourself down? What has worked for you?

Consider…

- Your strengths
- Things you are good at
- Things you enjoy
- Things that are **calming**
- Things you say to yourself

What did you learn from this exercise? Do you want to refine your relapse prevention plan?

Do you want to add additional coping skills to your action plan for managing mild symptoms of mania?

Team

Managing bipolar disorder requires having a good team in place. At a minimum, your team involves a psychiatrist, personal supports, and a hospital.

Building a team and using it effectively requires some effort. You can learn more about building your team here.

Psychiatrist

Supports

Hospital

Psychiatrist

About

Managing bipolar disorder requires taking medications. It requires using lifestyle skills. It requires using coping skills. It requires having a good team in place.

- Psychiatrist
- Personal supports
- Good hospital

For some people a therapist, case manager, and peer support grounds are important as well.

General Guidelines

Having a good relationship with your psychiatrist is important. They are there for more than writing prescriptions. You need to feel comfortable with them and trust them. You need to be able to be open and honest with them. You need to be able to reach out to them when you have symptoms.

When preparing for appointments, it can be helpful to make a list of things you want to tell your psychiatrist and any questions you may have.

Things to tell your psychiatrist:

- Symptoms
- Sleep habits
- Medication use
- Substance use
- Significant life changes

Things to ask your psychiatrist:

- What is my diagnosis?
- Do you think I'm having symptoms now?
- How will the medications help?
- What are the side effects?
- What should I do if I get side effects?
- What should I do if I miss a dose?

Ongoing Symptoms

Be sure that your psychiatrist knows whenever you have ongoing moderate symptoms for more than **2-3 days** or ongoing mild symptoms for more than **7 days**.

- Reach out and call them. Let them know what is going on.
- Agree on a plan of action together. This might involve a medication change. If you have any reservations about the plan, let them know! A plan is no good unless you are willing and able to follow it.
- Implement the plan. Evaluate each day whether or not things are getting better.
- Call your psychiatrist again if your symptoms continue and/or you cannot implement the plan.

Early Warning Signs

Be sure to call your psychiatrist any time you have early warning signs of mania or depression for more than 2 days in a row.

Things to share:

- How you are taking your medications
- How you are sleeping
- How you are spending your time
- Any alcohol or drug use

And:

- How you are taking care of yourself
- How relationships are going
- How work (school) is going
- Feedback you are getting from others

Try not to downplay things. It is common for people to think they can deal with it on their own. Symptoms are serious business. Let others know. Let them help!

Supports

About

Managing bipolar disorder requires taking medications. It requires using lifestyle skills. It requires using coping skills. It requires having a good team in place.

- Psychiatrist
- Personal supports
- Good hospital

For some people a therapist, case manager, and peer support groups are important as well.

Why Supports?

There are many reasons to have wellness supports.

- **Tranquility.** It is important that you not be alone with your bipolar disorder. Having others know what you have gone through and what you are going through makes things less stressful.

- **Commitment.** Letting others know about your intentions to take care of yourself will make it more likely you will stick to your wellness plan.

- **Emotional support.** They can provide emotional support. While they are not therapists, they can listen and offer you kindness.

- **Tangible support**. They can provide tangible support. You may need, for example, a ride to the hospital if you get ill.

- **Feedback.** They can provide feedback. There may be times when you have symptoms that are noticeable to others before they are noticeable to you. They can gently let you know if this happens.

Making It Work

Pick one or two people in your life to support your wellness. Select people who are caring and who you have known for some time. Family members, spouses, and close friends are good choices.

Encourage them to read about bipolar disorder. The Depression and Bipolar Support Alliance website is a good resource.

Meet with them one or two times after they have read the material. Review the information below.

- The lesson Basic Facts about Bipolar Disorder in foundations. Talk with them about what they read. Answer any questions they may have. Consider bringing them to an appointment with your psychiatrist to learn more.

- Review your *LiveWell* Wellness Plan. Discuss your plan to reduce risk as well as your plan for awareness and action.

- Discuss how you want them to tell you when you have early warning signs or other symptoms.

- Talk about what you want them to do if you dismiss, disagree, or argue with them about early warning signs or other symptoms.

Remember:

- Supports are not therapists. They should not be telling you what to do, and you should not be relying on them to solve your problems.

- Supports are not parents. They should not be monitoring whether or not you are following your wellness plan, and you should not ask them to do so (unless you have severe symptoms).

Hospital

About

Managing bipolar disorder requires taking medications. It requires using lifestyle skills. It requires using coping skills. It requires having a good team in place.

- Psychiatrist
- Personal supports
- Good hospital

For some people a therapist, case manager, and peer support groups are important as well.

Picking a Hospital

There are many things to consider when selecting your preferred hospital.

- What hospitals in your area are ranked the highest? Check out the *US News and World Report.*
- Is your psychiatrist on staff at an area hospital? Being on staff can help in the coordination of your care.

Be proactive. Find out the answers to these questions before you ever need to go to the hospital.

When to Go to the Hospital

Inpatient hospitalization is used to treat serious psychiatric problems. Go to the hospital whenever:

- You are thinking about suicide with some intention to act on these thoughts.

- You are engaging in dangerous behaviors such as spending lots of money or having promiscuous sex.

- You are having hallucinations, seeing, or hearing things.

- You are having delusions or ideas that do not line up with reality.
- You are unable to take care of yourself, such as not getting out of bed, not showering, and not going to work.
- If your psychiatrist thinks you should go to the hospital. They may see symptoms of which you are unaware.

What to Expect

Here are some things to expect if you're hospitalized.

- ✓ **Safety**. This is the primary concern. Everything about an inpatient unit, from the physical layout to the policies and procedures, are focused on preventing any patient from harming himself or anyone else.
- ✓ **Milieu.** The treatment environment is called the milieu and it has both structured and unstructured components. Structured components include group therapy, community meetings, and psychoeducation classes. Unstructured components include interactions between patients, staff, and visitors. All aspects of the milieu are designed to contribute to care and recovery.
- ✓ **Length of stay.** The average length of stay is usually less than one week, but this can vary depending on individual needs and treatment plans.
- ✓ **Psychiatrist.** An attending psychiatrist is assigned to each patient to oversee treatment and prescribe necessary medications. He/she will meet with you to review your progress every day during your inpatient stay.
- ✓ **Medication changes.** Most of the time, mild and even moderate symptoms can be treated on an outpatient-basis. In some cases, however, symptoms and impairment can become severe enough to warrant hospitalization. A stay in the hospital can provide a safe and stable environment that promotes recovery. With continuous nursing care available around the clock, an inpatient setting can also be a good choice for patients that require closer monitoring and/or medication changes. Medication changes can be made much more rapidly and efficiently on an inpatient unit because of the higher level of support available to monitor for and respond to any problems related to these more rapid changes.

- ✓ **Staff.** Other staff members, such as nurses, social workers, and therapists are also present on the unit to provide care and support to you within their area of expertise.

WELLNESS PLAN

TABLE OF CONTENTS

1. **MY RESOURCES**
    1.1. My Medications
    1.2. My Team
    1.3. My Skills
    1.4. My Charts

2. **REDUCE RISK**
    2.1. Sleep
    2.2. Medication
    2.3. Attend
    2.4. Routine
    2.5. Tranquil
    2.6. Social

3. **AWARENESS & ACTION**
    3.1. Severe Up
    3.2. Moderate Up
    3.3. Mild Up
    3.4. Slight Up
    3.5. Balanced
    3.6. Slight Down
    3.7. Mild Down
    3.8. Moderate Down
    3.9. Severe Down

1. My Resources

**My Medications**	Your psychiatric medication, Dose in mg, every morning, afternoon, or evening<br>Your psychiatric medication, Dose in mg, every morning, afternoon, or evening
**My Team**	Psychiatrist<br>*Your Psychiatrist*<br>Therapist<br>*Your Therapist*<br>Pharmacy<br>*Your Pharmacy*<br>Family<br>*Your Family*<br>Friend<br>*Your Friend*<br>Coach<br>*Your LiveWell Coach* (312)-503-1886
**My Skills**	*When you have added skills, they will appear here.*
**My Charts**	Medication<br>Sleep<br>Routine<br>Wellness

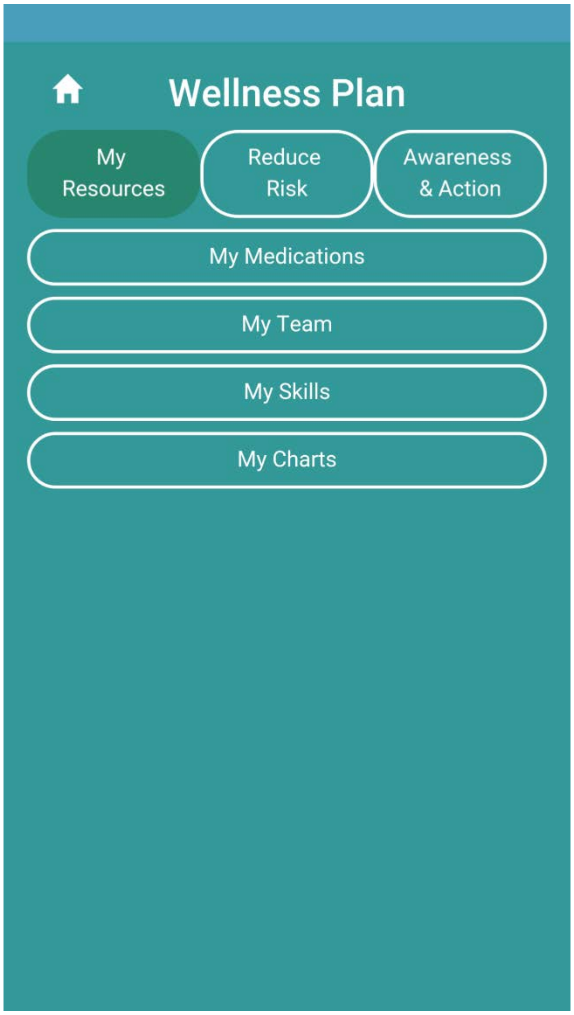

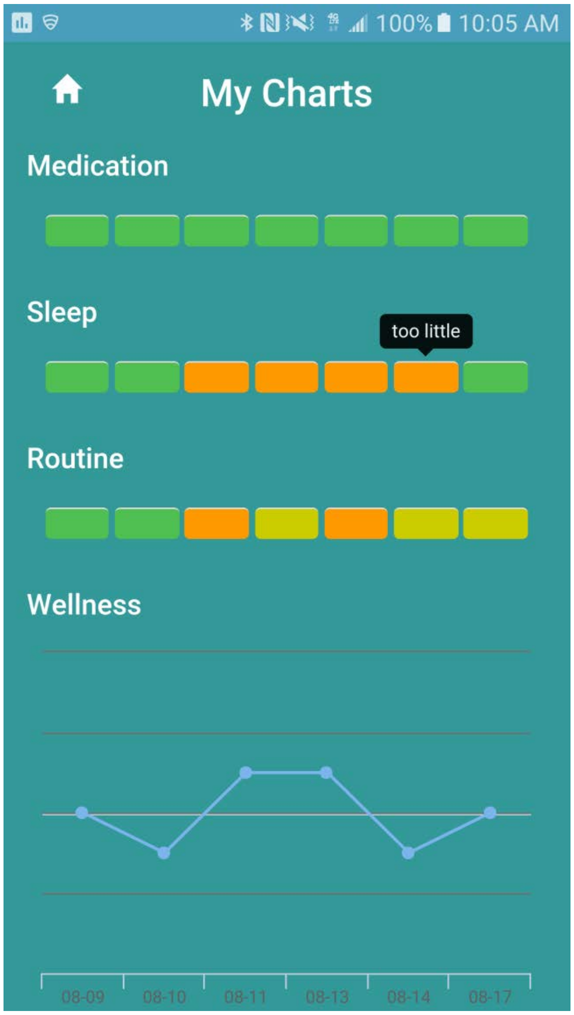

2. Reduce Risk*

**Sleep**	Sleep about 7-9 hours a night.<br>If less than 4 hours sleep per night for 2 nights in a row, then call psychiatrist.
**Medicine**	Take medication daily.<br>If miss 3-4 days in a row, then discuss with psychiatrist.
**Attend**	
**Routine**	Go to bed 10:30pm - 12:00am. Get up 7:00am - 8:30 am.
**Tranquil**	
**Social**	

* Default generic plans

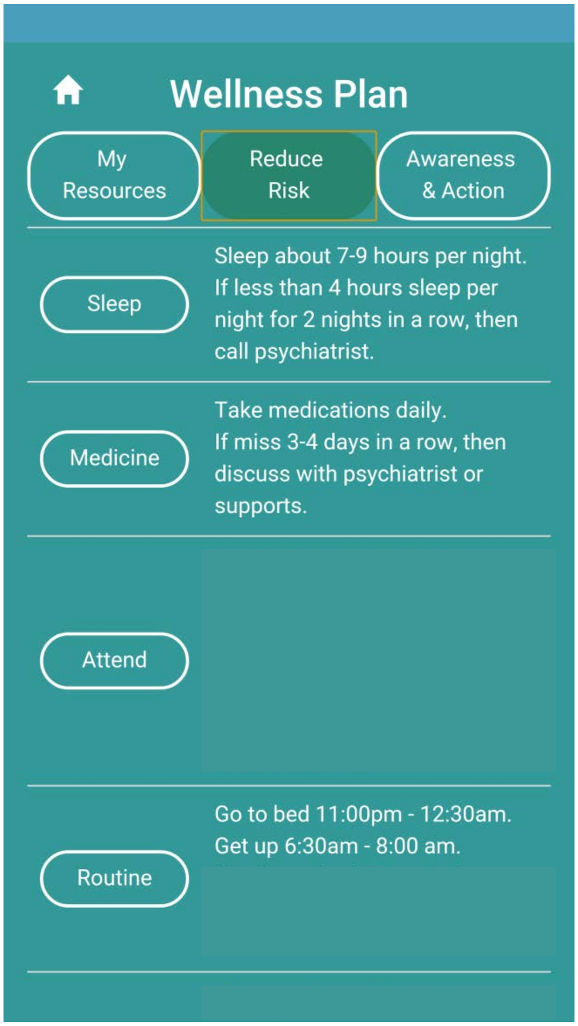

3. Awareness and Action

	(Generic/Personalized) Plans	(Personalized) Anchors	Definition
**+4 Severe Up**	Call psychiatrist or 911.<br>Go to the nearest emergency room.		Poor judgement. Dangerous behaviors.<br>Not sleeping. Hallucinations/delusions.
**+3 Moderate Up**	Work more closely with psychiatrist.<br>If not improving, get more intensive treatment.		Many symptoms day to day. Manic episode probably happening. Difficult to maintain activities/routine.
**+2 Mild Up**	Manage symptoms using coping skills.<br>Contact your supports.<br>If not improving, contact psychiatrist.		Some ongoing symptoms or early warning signs. Manic episode may be coming. Can still maintain activities/routine.
**+1 Slight Up**	Manage triggers using lifestyle skills.		Response recent/upcoming good event.<br>Likely normal variation in wellness.<br>Understandable and manageable.
**0 Balanced**	Maintain a healthy lifestyle.<br>Build skills.		Neither up nor down. Doing well.
**-1 Slight Down**	Manage triggers using lifestyle skills.		Response recent/upcoming bad event.<br>Likely normal variation in wellness.<br>Understandable and manageable.
**-2 Mild Down**	Manage symptoms using coping skills.<br>Contact your supports.<br>If not improving, contact psychiatrist.		Some ongoing symptoms or early warning signs. Depressive episode may be coming. Can still maintain activities/routine.
**-3 Moderate Down**	Work more closely with psychiatrist.<br>If not improving, get more intensive treatment.		Many symptoms day to day.<br>Depressive episode probably happening. Difficult to maintain activities/routine.
**-4 Severe Down**	Call psychiatrist or 911.<br>Go to the nearest emergency room.		Serious ideas about suicide.<br>Immobilized. Dangerous behaviors.<br>Disrupted sleep.<br>Hallucinations/delusions.

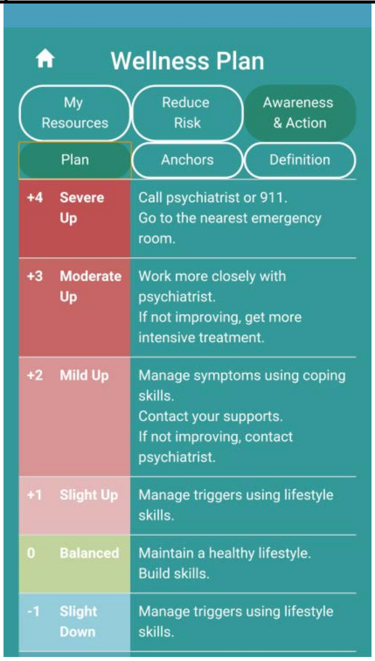

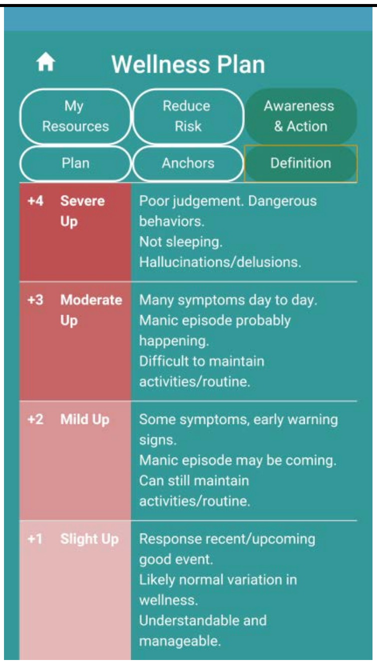

DAILY CHECK-IN

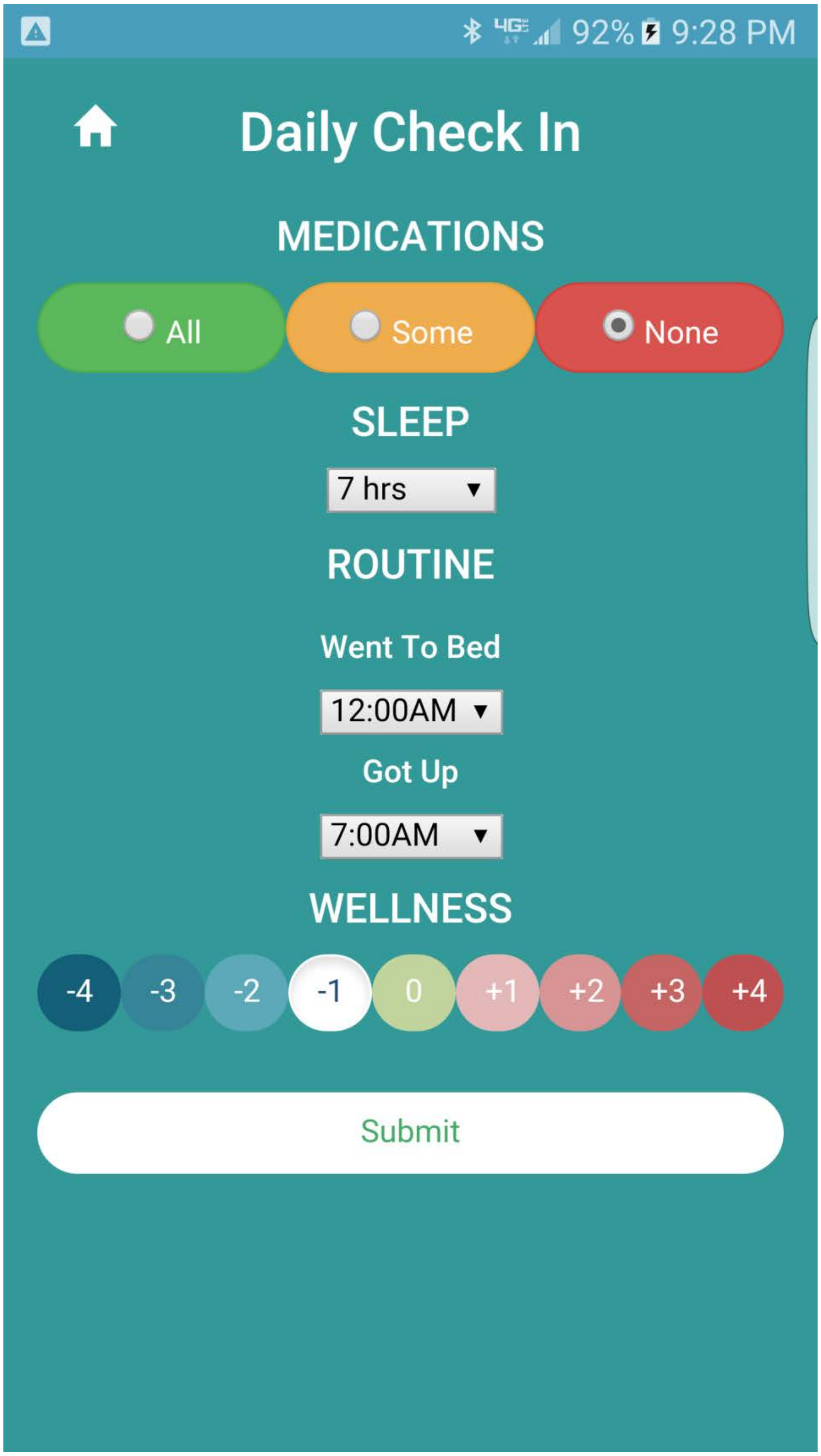

DAILY REVIEW

TABLE OF CONTENTS

1. **Crisis - Up**
2. **Crisis - Down**
3. **Episode - Continuing Up**
4. **Episode - Continuing Down**
5. **Episode - Improving Up**
6. **Episode - Improving Down**
7. **Episode - Improving Balanced**
8. **Worsening Symptoms – Up**
   8.1 Work with psychiatrist (choice 1)
   8.2 Work with supports (choice 2)
9. **Worsening Symptoms – Down**
   9.1 Work with psychiatrist (choice 1)
   9.2 Work with supports (choice 2)
10. **Recovering - Continuing Up**
11. **Recovering - Continuing Down**
12. **Prodromal - Continuing Up**
    12.1 Talk to psychiatrist (choice 1)
    12.2 Talk to supports (choice 2)
    12.3 Dial down (choice 3)
13. **Prodromal - Continuing Down**
    13.1 Talk to psychiatrist (choice 1)
    13.2 Talk to supports (choice 2)
    13.3 Dial up (choice 3)
14. **Recovering - Improving Balanced**
15. **Prodromal - Improving Balanced**
16. **Early Warning Signs - Up**
17. **Early Warning Signs - Down**
18. **High Risk - Medication Adherence**
19. **High Risk - Sleeping Too Little**
20. **High Risk - Sleeping Too Much**
21. **Moderate Risk – Medication Adherence**
    21.1 Really necessary (choice 1)

21.1.1 Do I have bipolar (choice 1)

21.1.2 What if no symptoms (choice 2)

21.1.3 Manage on my own (choice 3)

21.1.4 Does medication help (choice 4)

21.2 Side effects (choice 2)

21.2.1 Current (choice 1)

21.2.2 Fear of (choice 2)

21.3 Others influence (choice 3)

21.3.1 Psychiatrist (choice 1)

21.3.2 Therapist (choice 2)

21.3.3 Family and friends (choice 3)

21.4 Practical problems (choice 4)

21.4.1 Expensive (choice 1)

21.4.2 Inconvenient (choice 2)

21.4.3 Forgetful (choice 3)

22. Moderate Risk - Sleeping Too Little

22.1 Good habits (choice 1)

22.2 Good environments (choice 2)

22.3 Exercise, diet, substances (choice 3)

22.4 Managing anxiety (choice 4)

23. Moderate Risk - Sleeping Too Much

23.1 Sedating medications (choice 1)

23.2 Catch up sleep (choice 2)

23.3 Avoiding stress (choice 3)

24. Moderate Risk - Sleeping Erratically

25. Moderate Risk - Irregular Routine

26. Staying Well

26.1 Awareness (choice 1)

26.1.1 Early warning signs (choice 1)

26.1.2 Symptoms (choice 2)

26.1.3 Triggers (choice 3)

26.2 Lifestyle (choice 2)

26.2.1 Sleep (choice 1)

26.2.2 Medications (choice 2)

26.2.3. Attend (choice 3

26.2.4 Routine (choice 4)

- 26.2.5 Tranquil (choice 5)
- 26.2.6 Social (choice 6)

26.3 Action (choice 3)

- 26.3.1 Dial up (choice 1)
- 26.3.2 Dial down (choice 2)

26.4 Team (choice 4)

- 26.4.1 Providers (choice 1)
- 26.4.2 Supports (choice 2)
- 26.4.3 Hospitals (choice 3)

Daily Review Feedback Category 1: Crisis - Up

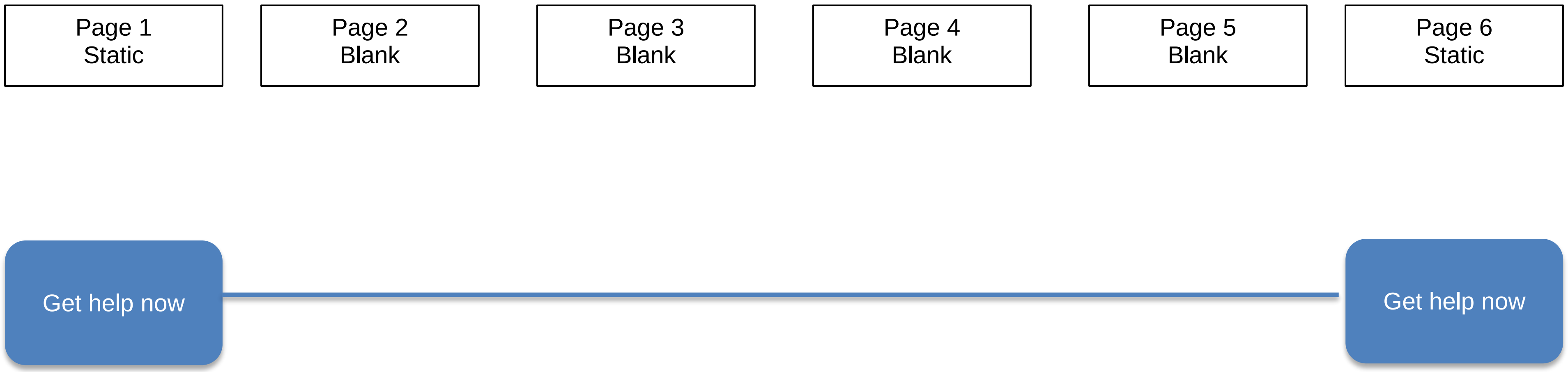

Daily Review Feedback Categories 1: Crisis - Up

Get help now (S1)

Get help now (S1)

1<br>STATIC	2<br>BLANK	3<br>BLANK	4<br>BLANK	5<br>BLANK	6<br>Static
Sorry you are not well.<br><br>You can get help. Reach out. Things will get better. Take action immediately.<br><br>Call your psychiatrist.<br>Call your supports<br><br>If you are thinking about suicide, engaging in dangerous behaviors, or having psychotic symptoms, then call 911 or go to your nearest emergency room.					Sorry you are not well.<br><br>You can get help. Reach out. Things will get better. Take action immediately.<br><br>Call your psychiatrist.<br>Call your supports<br><br>If you are thinking about suicide, engaging in dangerous behaviors, or having psychotic symptoms, then call 911 or go to your nearest emergency room.

Daily Review Feedback Category 2: Crisis - Down

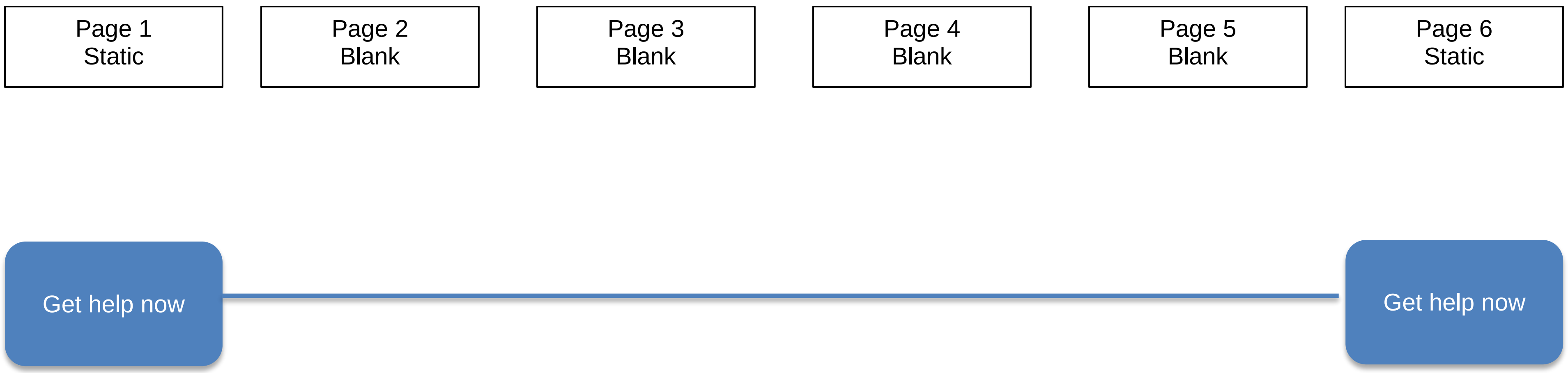

Daily Review Feedback Categories 2: Crisis - Down

Get help now (S1)

Get help now (S1)

1 STATIC	2 BLANK	3 BLANK	4 BLANK	5 BLANK	6 Static
Sorry you are not well.<br><br>You can get help. Reach out. Things will get better. Take action immediately.<br><br>Call your psychiatrist.<br>Call your supports<br><br>If you are thinking about suicide, engaging in dangerous behaviors, or having psychotic symptoms, then call 911 or go to your nearest emergency room.					Sorry you are not well.<br><br>You can get help. Reach out. Things will get better. Take action immediately.<br><br>Call your psychiatrist.<br>Call your supports<br><br>If you are thinking about suicide, engaging in dangerous behaviors, or having psychotic symptoms, then call 911 or go to your nearest emergency room.

Daily Review Feedback Category 3: Episode - Continuing Up

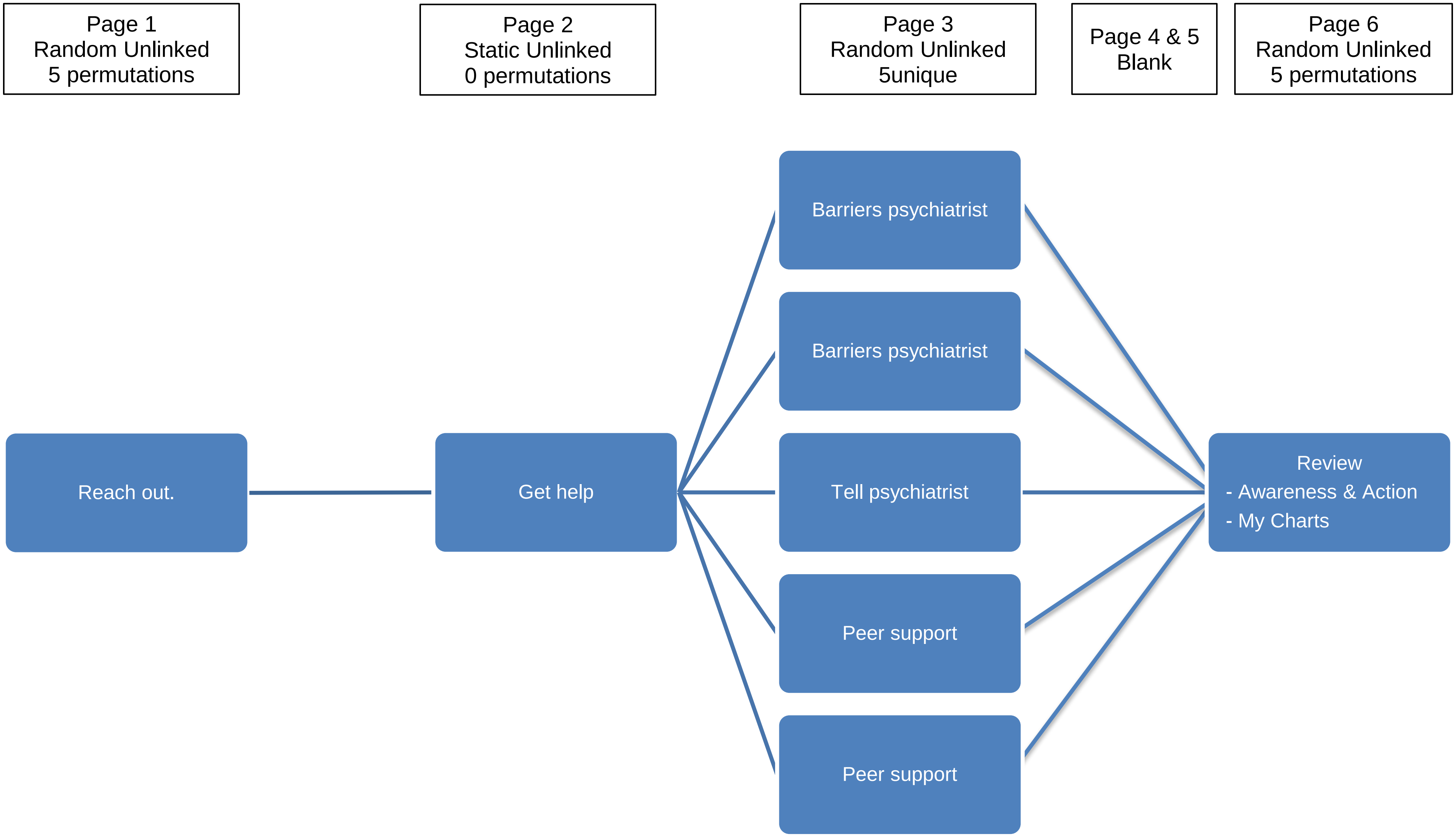

Daily Review Feedback Categories 3: Episode - Continuing Up

Reach out (P1)	Get help (S1)	Barriers (U1)			A & A (P1)
**1<br>RAND0M UNLINKED**	**2<br>STATIC**	**3<br>RANDOM UNLINKED**	**4<br>BLANK**	**5<br>BLANK**	**6<br>RANDOM UNLINKED**
It looks like you've been having symptoms for a while now.<br><br>Work closely with your psychiatrist and other members of your mental health care team to give yourself the best chance of recovery.<br><br>Continue to read more...	The most important thing right now is to get well. Additional help is available.<br><br>• If you haven't already contacted your psychiatrist about your symptoms, now is a good time to do so<br><br>• Consider more intensive treatment, like a partial hospital program or an intensive outpatient program.<br><br>• Consider increasing the frequency in which you attend psychotherapy, at least until your symptoms remit. If you are not in therapy, consider starting.	What might get in the way of reaching out? Do any of these apply to you?<br><br>• I think I can handle it on my own.<br>• I'm embarrassed to reach out.<br>• I don't want to bother my psychiatrist.<br><br>There is no shame here. Even if your symptoms came about after you stopped taking your medications or started smoking marijuana frequently. No one is expected to be perfect!<br><br>Your psychiatrist is there for you! They want to help. So reach out.			Review your ideas for coping with symptoms in Awareness & Action in the Wellness Plan below.<br><br>Get well...

Daily Review Feedback Categories 3: Episode - Continuing Up

Reach out (P2)	Get help (S1)	Barriers (U2)			A & A (P2)

1<br>RAND0M UNLINKED	2<br>STATIC	3<br>RANDOM UNLINKED	4<br>BLANK	5<br>BLANK	6<br>RANDOM UNLINKED
It appears that you've been experiencing symptoms for quite some time now.<br><br>If you haven't already, now is the time to reach out to your team for support.<br><br>Continue to read more...	The most important thing right now is to get well. Additional help is available.<br><br>• If you haven't already contacted your psychiatrist about your symptoms, now is a good time to do so<br><br>• Consider more intensive treatment, like a partial hospital program or an intensive outpatient program.<br><br>• Consider increasing the frequency in which you attend psychotherapy, at least until your symptoms remit. If you are not in therapy, consider starting.	What might get in the way of reaching out? Do any of these apply to you?<br><br>• I think I can handle it on my own.<br>• I'm embarrassed to reach out.<br>• I don't want to bother my psychiatrist.<br><br>There is no shame here. Even if your symptoms came about after you stopped taking your medications or started drinking frequently. No one is expected to be perfect!<br><br>Your psychiatrist is there for you! They want to help. So reach out.			Review your anchors for Moderate Up in Awareness & Action in the Wellness Plan.<br><br>Get well...

Daily Review Feedback Categories 3: Episode - Continuing Up

Reach out (P3)	Get help (S1)	Barriers (U3)			A & A (P3)
**1**<br>**RAND0M UNLINKED**	**2**<br>**STATIC**	**3**<br>**RANDOM UNLINKED**	**4**<br>**BLANK**	**5**<br>**BLANK**	**6**<br>**RANDOM UNLINKED**
You've been describing symptoms for a while now.<br><br>Be sure to reach out to your psychiatrist and let them what know what's going on with you. It may also be a good time to start thinking about other treatments that could be helpful right now. Ask your psychiatrist for his/her opinion.<br><br>Continue to read more...	The most important thing right now is to get well. Additional help is available.<br><br>• If you haven't already contacted your psychiatrist about your symptoms, now is a good time to do so<br><br>• Consider more intensive treatment, like a partial hospital program or an intensive outpatient program.<br><br>• Consider increasing the frequency in which you attend psychotherapy, at least until your symptoms remit. If you are not in therapy, consider starting.	Be sure to tell your psychiatrist the following things:<br><br>• How you are taking your medications<br>• How much and when you are sleeping<br>• How you are spending your time<br>• Any alcohol or drug use			Take a look at your description of Moderate Up in Awareness & Action in the Wellness Plan.<br><br>Get well...

Daily Review Feedback Categories 3: Episode - Continuing Up

Reach out (P4)	Get help (S1)	Barriers (U4)			My Charts (P4)
**1**<br>**RANDOM UNLINKED**	**2**<br>**STATIC**	**3**<br>**RANDOM UNLINKED**	**4**<br>**BLANK**	**5**<br>**BLANK**	**6**<br>**RANDOM UNLINKED**
You've been reporting ongoing symptoms for some time now.<br><br>If you haven't already, be sure to let your psychiatrist know what is going on with you.<br><br>Continue to read more...	The most important thing right now is to get well. Additional help is available.<br><br>• If you haven't already contacted your psychiatrist about your symptoms, now is a good time to do so<br><br>• Consider more intensive treatment, like a partial hospital program or an intensive outpatient program.<br><br>• Consider increasing the frequency in which you attend psychotherapy, at least until your symptoms remit. If you are not in therapy, consider starting.	Also consider getting some support from peers!<br><br>Go to the website for the depression and bipolar alliance:<br><br>www.dbsalliance.org<br><br>Check out Peer Inspiration. There are stories and videos about others with bipolar disorder who have gotten and stayed well.			Check out My Charts in the Wellness Plan. Look for patterns in your wellness over time.<br><br>Get well...

Daily Review Feedback Categories 3: Episode - Continuing Up

Reach out (P5)	Get help (S1)	Barriers (U5)			A & A (P5)
**1**<br>**RAND0M UNLINKED**	**2**<br>**STATIC**	**3**<br>**RANDOM UNLINKED**	**4**<br>**BLANK**	**5**<br>**BLANK**	**6**<br>**RANDOM UNLINKED**
You've been experiencing symptoms for some time now.<br><br>High level, ongoing symptoms require the attention of your team. Unfortunately, you cannot do it on your own.<br><br>Continue to read more...	The most important thing right now is to get well. Additional help is available.<br><br>• If you haven't already contacted your psychiatrist about your symptoms, now is a good time to do so<br><br>• Consider more intensive treatment, like a partial hospital program or an intensive outpatient program.<br><br>• Consider increasing the frequency in which you attend psychotherapy, at least until your symptoms remit. If you are not in therapy, consider starting.	Also consider getting some support from peers!<br><br>Go to the website for the depression and bipolar alliance:<br><br>www.dbsalliance.org<br><br>Attend a support group in your area.			Take a look at your action plan for Moderate Up in Awareness & Action. Go to the Wellness Plan below.<br><br>Get well...

Daily Review Feedback Category 4: Episode - Continuing Down

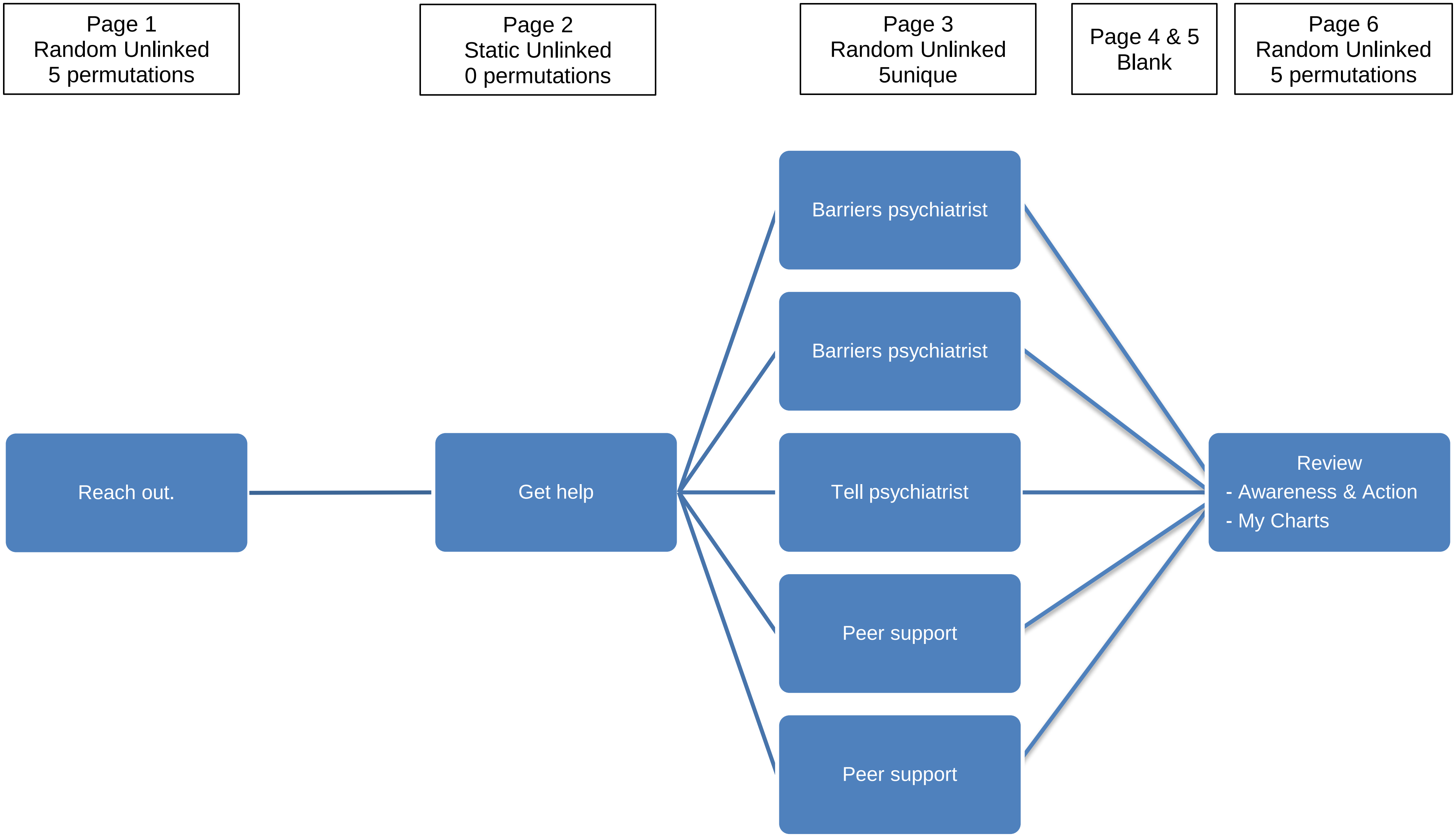

Daily Review Feedback Categories 4: Episode Continuing, Down

Reach out (P1)	Get help (S1)	Barriers (U1)			A & A (P1)
**1**<br>**RANDOM UNLINKED**	**2**<br>**STATIC**	**3**<br>**RANDOM UNLINKED**	**4**<br>**BLANK**	**5**<br>**BLANK**	**6**<br>**RANDOM UNLINKED**
It looks like you've been having symptoms for a while now.<br><br>Work closely with your psychiatrist and other members of your mental health care team to give yourself the best chance of recovery.<br><br>Continue to read more...	The most important thing right now is to get well. Additional help is available.<br><br>• If you haven't already contacted your psychiatrist about your symptoms, now is a good time to do so<br><br>• Consider more intensive treatment, like a partial hospital program or an intensive outpatient program.<br><br>• Consider increasing the frequency in which you attend psychotherapy, at least until your symptoms remit. If you are not in therapy, consider starting.	What might get in the way of reaching out? Do any of these apply to you?<br><br>• I think I can handle it on my own.<br>• I'm embarrassed to reach out.<br>• I don't want to bother my psychiatrist.<br><br>There is no shame here. Even if your symptoms came about after you stopped taking your medications or started smoking marijuana frequently. No one is expected to be perfect!<br><br>Your psychiatrist is there for you! They want to help. So reach out.			Review your ideas for coping with symptoms in Awareness & Action in the Wellness Plan below.<br><br>Get well...

Daily Review Feedback Categories 4: Episode Continuing, Down

Reach out (P2)	Get help (S1)	Barriers (U2)			A & A (P6)

1<br>RAND0M UNLINKED	2<br>STATIC	3<br>RANDOM UNLINKED	4<br>BLANK	5<br>BLANK	6<br>RANDOM UNLINKED
It appears that you've been experiencing symptoms for quite some time now.<br><br>If you haven't already, now is the time to reach out to your team for support.<br><br>Continue to read more...	The most important thing right now is to get well. Additional help is available.<br><br>• If you haven't already contacted your psychiatrist about your symptoms, now is a good time to do so<br><br>• Consider more intensive treatment, like a partial hospital program or an intensive outpatient program.<br><br>• Consider increasing the frequency in which you attend psychotherapy, at least until your symptoms remit. If you are not in therapy, consider starting.	What might get in the way of reaching out? Do any of these apply to you?<br><br>• I think I can handle it on my own.<br>• I'm embarrassed to reach out.<br>• I don't want to bother my psychiatrist.<br><br>There is no shame here. Even if your symptoms came about after you stopped taking your medications or started drinking frequently. No one is expected to be perfect!<br><br>Your psychiatrist is there for you! They want to help. So reach out.			Review your anchors for Moderate Down in Awareness & Action in the Wellness Plan.<br><br>Get well...

Daily Review Feedback Categories 4: Episode Continuing, Down

Reach out (P3)	Get help (S1)	Barriers (U3)			A & A (P7)
**1**<br>**RAND0M UNLINKED**	**2**<br>**STATIC**	**3**<br>**RANDOM UNLINKED**	**4**<br>**BLANK**	**5**<br>**BLANK**	**6**<br>**RANDOM UNLINKED**
You've been describing symptoms for a while now.<br><br>Be sure to reach out to your psychiatrist and let them what know what's going on with you. It may also be a good time to start thinking about other treatments that could be helpful right now. Ask your psychiatrist for his/her opinion.<br><br>Continue to read more...	The most important thing right now is to get well. Additional help is available.<br><br>• If you haven't already contacted your psychiatrist about your symptoms, now is a good time to do so<br><br>• Consider more intensive treatment, like a partial hospital program or an intensive outpatient program.<br><br>• Consider increasing the frequency in which you attend psychotherapy, at least until your symptoms remit. If you are not in therapy, consider starting.	Be sure to tell your psychiatrist the following things:<br><br>• How you are taking your medications<br>• How much and when you are sleeping<br>• How you are spending your time<br>• Any alcohol or drug use			Take a look at your description of Moderate Down in Awareness & Action in the Wellness Plan.<br><br>Get well...

Daily Review Feedback Categories 4: Episode Continuing, Down

Reach out (P4)	Get help (S1)	Barriers (U4)			My Charts (P4)
**1**<br>**RANDOM UNLINKED**	**2**<br>**STATIC**	**3**<br>**RANDOM UNLINKED**	**4**<br>**BLANK**	**5**<br>**BLANK**	**6**<br>**RANDOM UNLINKED**
You've been reporting ongoing symptoms for some time now.<br><br>If you haven't already, be sure to let your psychiatrist know what is going on with you.<br><br>Continue to read more...	The most important thing right now is to get well. Additional help is available.<br><br>• If you haven't already contacted your psychiatrist about your symptoms, now is a good time to do so<br><br>• Consider more intensive treatment, like a partial hospital program or an intensive outpatient program.<br><br>• Consider increasing the frequency in which you attend psychotherapy, at least until your symptoms remit. If you are not in therapy, consider starting.	Also consider getting some support from peers!<br><br>Go to the website for the depression and bipolar alliance:<br><br>www.dbsalliance.org<br><br>Check out Peer Inspiration. There are stories and videos about others with bipolar disorder who have gotten and stayed well.			Check out My Charts in the Wellness Plan. Look for patterns in your wellness over time.<br><br>Get well...

Daily Review Feedback Categories 4: Episode Continuing, Down

Reach out (P5)	Get help (S1)	Barriers (U5)			A & A (P8)
**1**<br>**RANDOM UNLINKED**	**2**<br>**STATIC**	**3**<br>**RANDOM UNLINKED**	**4**<br>**BLANK**	**5**<br>**BLANK**	**6**<br>**RANDOM UNLINKED**
You've been experiencing symptoms for some time now.<br><br>High level, ongoing symptoms require the attention of your team. Unfortunately, you cannot do it on your own.<br><br>Continue to read more...	The most important thing right now is to get well. Additional help is available.<br><br>• If you haven't already contacted your psychiatrist about your symptoms, now is a good time to do so<br><br>• Consider more intensive treatment, like a partial hospital program or an intensive outpatient program.<br><br>• Consider increasing the frequency in which you attend psychotherapy, at least until your symptoms remit. If you are not in therapy, consider starting.	Also consider getting some support from peers!<br><br>Go to the website for the depression and bipolar alliance:<br><br>www.dbsalliance.org<br><br>Attend a support group in your area.			Take a look at your action plan for Moderate Down in Awareness & Action. Go to the Wellness Plan below.<br><br>Get well...

Daily Review Feedback Category 5: Episode Improving, Up

Page 1	Page 2	Page 3	Page 4	Page 5	Page 6
Random Unlinked<br>5 permutations	Static	Random Unlinked<br>5 unique	Blank	Blank	Random Unlinked<br>5 permutations

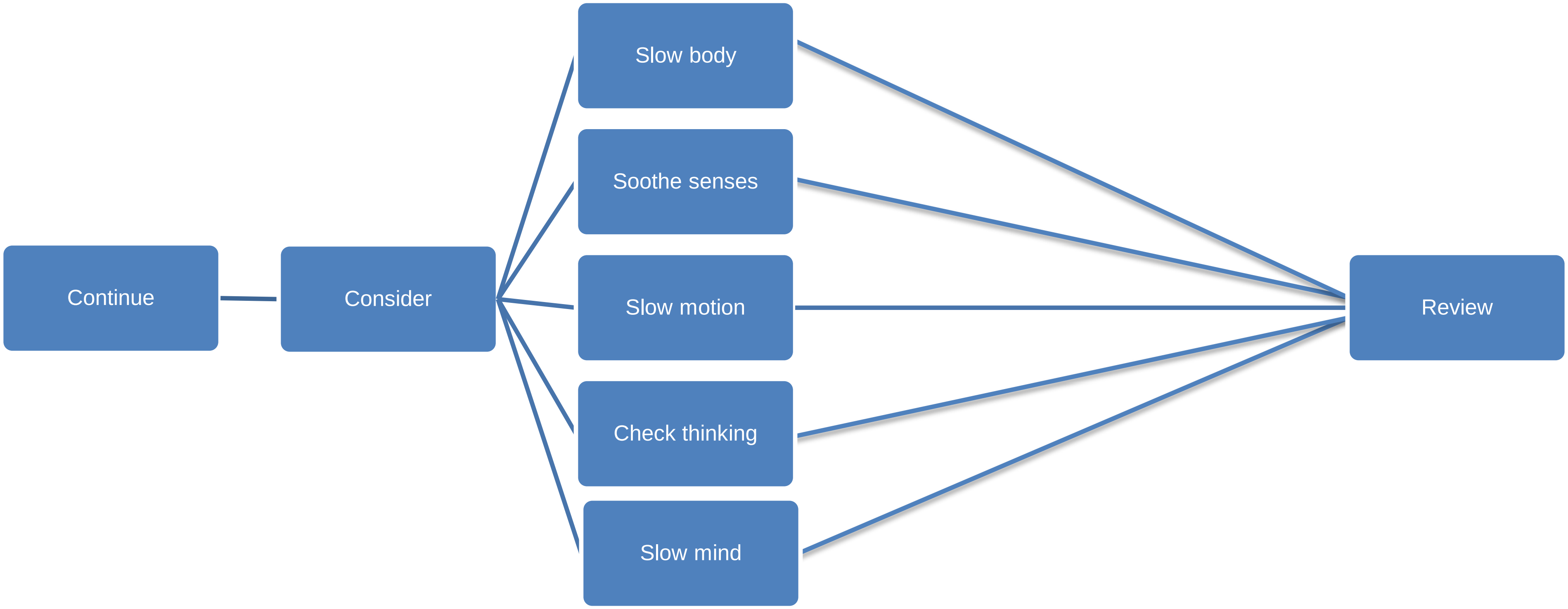

Daily Review Feedback Category 5: Episode Improving, Up

Continue (P1)	Consider (S1)	Slow body (U1)			A & A (P1)
**1**<br>**RANDOM UNLINKED**	**2**<br>**STATIC**	**3**<br>**RANDOM UNLINKED**	**4**<br>**BLANK**	**5**<br>**BLANK**	**6**<br>**RANDOM UNLINKED**
Glad to see you're feeling better. Looks like you're having fewer symptoms today.<br><br>Keep doing whatever you have been doing. It seems to be working!<br><br>Continue to read more...	Things to do:<br><br>Keep up the efforts to get and stay well. In addition to whatever you have been doing that is helpful, consider the following:<br><br>✓✓ Continue working with your psychiatrist.<br><br>✓✓ Continue working with your supports.<br><br>✓✓ Keep using coping skills.<br><br>✓✓ Keep using lifestyle skills.	TIP OF THE DAY<br><br>Slowing your body repairs mild ups in mood. Put on the brakes. Get your body to be quiet on the inside. Be still. Relax.<br><br>• Take deep breaths.<br>• Stretch your body.<br>• Try muscle relaxation.<br>• Do yoga at home. Calm<br><br>your body. Ground yourself.			Review your ideas for Mild Up in Awareness & Action in the Wellness Plan.<br><br>Take the time to recover...

Daily Review Feedback Category 5: Episode Improving, Up

Continue (P2)	Consider (S1)	Soothe senses (U2)			A & A (P2)
**1**<br>**RANDOM UNLINKED**	**2**<br>**STATIC**	**3**<br>**RANDOM UNLINKED**	**4**<br>**BLANK**	**5**<br>**BLANK**	**6**<br>**RANDOM UNLINKED**
It's great that you're doing better today.<br><br>Continue taking care of yourself to keep the momentum going in the right direction.<br><br>Press continue for tips…	Things to do:<br><br>Keep up the efforts to get and stay well. In addition to whatever you have been doing that is helpful, consider the following:<br><br>✓✓ Continue working with your psychiatrist.<br><br>✓✓ Continue working with your supports.<br><br>✓✓ Keep using coping skills.<br><br>✓✓ Keep using lifestyle skills.	TIP OF THE DAY<br><br>Take 10 minutes to sit still. Engage your senses in a soothing way:<br><br>• Draw a peaceful scene such as an ocean, garden, or rural path.<br>• Take a warm bath. Put lavender salts or some other oil in your tub.<br>• Meditate on your breath. Close your eyes and breathe slowly. Pay attention only to your breath. If your mind wanders, gently bring it back.			Take a look at your action plan for Mild Up in Awareness & Action in the Wellness Plan.<br><br>Take the time to recover…

Daily Review Feedback Category 5: Episode Improving, Up

Continue (P3)	Consider (S1)	Slow motion (U3)			A & A (P3)
**1**<br>**RANDOM UNLINKED**	**2**<br>**STATIC**	**3**<br>**RANDOM UNLINKED**	**4**<br>**BLANK**	**5**<br>**BLANK**	**6**<br>**RANDOM UNLINKED**
Good to see things are getting better and that you're having fewer symptoms today.<br><br>Stay on the road to recovery. Continue to read more…	Things to do:<br><br>Keep up the efforts to get and stay well. In addition to whatever you have been doing that is helpful, consider the following:<br><br>✓✓ Continue working with your psychiatrist.<br><br>✓✓ Continue working with your supports.<br><br>✓✓ Keep using coping skills.<br><br>✓✓ Keep using lifestyle skills.	TIP OF THE DAY<br><br>Go---slow---mo…<br><br>Take 10 minutes to act in slow motion.<br><br>• Walk around your home at 50% your normal pace.<br>• Do a task at 50% your normal pace.<br>• Breathe at 50% your normal pace.<br><br>Slow your body down. What does it feel like?			Review your description of Mild Up in Awareness & Action. See what you had said in the Wellness Plan.<br><br>Take the time to recover…

Daily Review Feedback Category 5: Episode Improving, Up

Continue (P4) | Consider (S1) | Check thinking (U4) | My Charts (P4)

1<br>RANDOM UNLINKED	2<br>STATIC	3<br>RANDOM UNLINKED	4<br>BLANK	5<br>BLANK	6<br>RANDOM UNLINKED
Glad to see that you're experiencing less intense symptoms today.<br><br>Continue using coping skills and your support team to stay on track.<br><br>Continue for tips…	Things to do:<br><br>Keep up the efforts to get and stay well. In addition to whatever you have been doing that is helpful, consider the following:<br><br>✓✓ Continue working with your psychiatrist.<br><br>✓✓ Continue working with your supports.<br><br>✓✓ Keep using coping skills.<br><br>✓✓ Keep using lifestyle skills.	TIP OF THE DAY<br><br>Mania can lead to alterations in thinking:<br><br>• Becoming unusually optimistic<br>• Feeling unusually highly about yourself (grandiose)<br>• Feeling overly concerned and skeptical about others (paranoia)<br><br>Check your thinking when you're up. Is there evidence to support your beliefs? Is there evidence that contradicts your beliefs? Be realistic!!!			Take a look at My Charts in the Wellness Plan. Do you see any patterns?<br><br>Take the time to recover…

Daily Review Feedback Category 5: Episode Improving, Up

Continue (P5) | Consider (S1) | Slow mind (U5) | My Skills (P5)

1<br>RANDOM UNLINKED	2<br>STATIC	3<br>RANDOM UNLINKED	4<br>BLANK	5<br>BLANK	6<br>RANDOM UNLINKED
You're reporting less intense symptoms today. That's great news.<br><br>Whatever you are doing to help you get better, stick with it. Remember, one step at a time.<br><br>Continue for additional ideas…	Things to do:<br><br>Keep up the efforts to get and stay well. In addition to whatever you have been doing that is helpful, consider the following:<br><br>✓✓ Continue working with your psychiatrist.<br><br>✓✓ Continue working with your supports.<br><br>✓✓ Keep using coping skills.<br><br>✓✓ Keep using lifestyle skills.	TIP OF THE DAY<br><br>Mania is associated with an increase in the speed or flow of thoughts. Sometimes this feels good, sometimes this feels bad.<br><br>Is this happening to you right now?<br><br>Take action to slow your thinking down. This is easier said than done! But quiet your mind:<br><br>• Write in a journal, slowly.<br>• Talk to someone, slowly.			Take a look at My Skills in the Wellness Plan. Consider trying out a skill you selected as a preferred skill.<br><br>Take the time to recover…

Daily Review Feedback Category 6: Episode Improving, Down

Page 1<br>Random Unlinked<br>5 permutations	Page 2<br>Static	Page 3<br>Random Unlinked<br>5 unique	Page 4<br>Blank	Page 5<br>Blank	Page 6<br>Random Unlinked<br>5 permutations

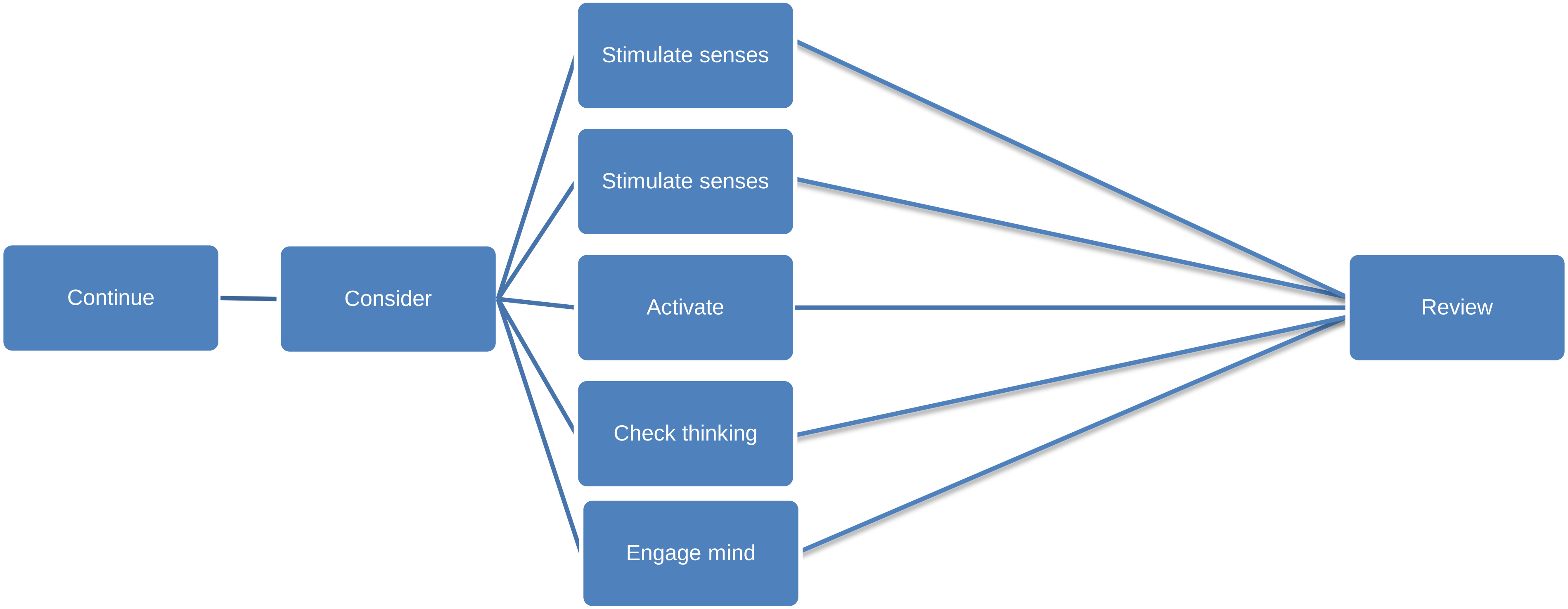

Daily Review Feedback Category 6: Episode Improving, Down

Continue (P1)	Consider (S1)	Stimulate senses (U6)			A & A (P6)
**1 RANDOM UNLINKED**	**2 STATIC**	**3 RANDOM UNLINKED**	**4 BLANK**	**5 BLANK**	**6 RANDOM UNLINKED**
Glad to see you're feeling better. Looks like you're having fewer symptoms today.<br><br>Keep doing whatever you have been doing. It seems to be working!<br><br>Continue to read more…	Things to do:<br><br>Keep up the efforts to get and stay well. In addition to whatever you have been doing that is helpful, consider the following:<br><br>✓✓ Continue working with your psychiatrist.<br><br>✓✓ Continue working with your supports.<br><br>✓✓ Keep using coping skills.<br><br>✓✓ Keep using lifestyle skills.	TIP OF THE DAY<br><br>Take 10 minutes to invigorate yourself. Engage your senses in an stimulating way:<br><br>• Listen to upbeat music, maybe even dance along.<br><br>• Eat something sweet or savory, notice your reaction.<br><br>• Take time and draw an intense picture, take it in.			Review your ideas for Mild Down in Awareness & Action in the Wellness Plan.<br><br>Take the time to recover…

Daily Review Feedback Category 6: Episode Improving, Down

Continue (P2)	Consider (S1)	Stimulate senses (U7)			A & A (P7)
**1**<br>**RANDOM UNLINKED**	**2**<br>**STATIC**	**3**<br>**RANDOM UNLINKED**	**4**<br>**BLANK**	**5**<br>**BLANK**	**6**<br>**RANDOM UNLINKED**
It's great that you're doing better today.<br><br>Continue taking care of yourself to keep the momentum going in the right direction.<br><br>Press continue for tips…	Things to do:<br><br>Keep up the efforts to get and stay well. In addition to whatever you have been doing that is helpful, consider the following:<br><br>✓✓ Continue working with your psychiatrist.<br><br>✓✓ Continue working with your supports.<br><br>✓✓ Keep using coping skills.<br><br>✓✓ Keep using lifestyle skills.	TIP OF THE DAY<br><br>Take 10 minutes to invigorate yourself. Engage your senses in an stimulating way:<br><br>• Close your eyes and touch an object, notice how it feels.<br>• Smell something strong but pleasant, describe your experience.<br>• Eat something sweet or savory, notice your reaction.<br>• Take time and draw an intense picture, take it in.			Take a look at your action plan for Mild Down in Awareness & Action. Go to the Wellness Plan to review.<br><br>Take the time to recover…

Daily Review Feedback Category 6: Episode Improving, Down

Continue (P3)	Consider (S1)	Activate (U8)			A & A (P8)
**1**<br>**RANDOM UNLINKED**	**2**<br>**STATIC**	**3**<br>**RANDOM UNLINKED**	**4**<br>**BLANK**	**5**<br>**BLANK**	**6**<br>**RANDOM UNLINKED**
Good to see things are getting better and that you're having fewer symptoms today.<br><br>Stay on the road to recovery. Continue to read more…	Things to do:<br><br>Keep up the efforts to get and stay well. In addition to whatever you have been doing that is helpful, consider the following:<br><br>✓✓ Continue working with your psychiatrist.<br><br>✓✓ Continue working with your supports.<br><br>✓✓ Keep using coping skills.<br><br>✓✓ Keep using lifestyle skills.	TIP OF THE DAY<br><br>Pick up the pace! Take<br><br>10 minutes to get moving.<br><br>• Walk around your home at double your normal pace.<br>• Do a task at double your normal pace.<br>• Take a walk at double your normal pace.<br>• Dance in your living room at double your normal pace.<br><br>Speed your body up. What does it feel like?			Review your description of Mild Down in Awareness & Action. See what you had said by clicking on the Wellness Plan.<br><br>Take the time to recover…

Daily Review Feedback Category 6: Episode Improving, Down

Continue (P4) | Consider (S1) | Check thinking (U9) | My Charts (P4)

1<br>RANDOM UNLINKED	2<br>STATIC	3<br>RANDOM UNLINKED	4<br>BLANK	5<br>BLANK	6<br>RANDOM UNLINKED
Glad to see that you're experiencing less intense symptoms today.<br><br>Continue using coping skills and your support team to stay on track.<br><br>Continue for tips…	Things to do:<br><br>Keep up the efforts to get and stay well. In addition to whatever you have been doing that is helpful, consider the following:<br><br>✓✓ Continue working with your psychiatrist.<br><br>✓✓ Continue working with your supports.<br><br>✓✓ Keep using coping skills.<br><br>✓✓ Keep using lifestyle skills.	TIP OF THE DAY<br><br>Depression can lead to alterations in thinking:<br><br>• Becoming unusually pessimistic<br>• Thinking you're worthless<br>• Thinking others don't care<br>• Feeling hopeless<br>• Feeling helpless<br><br>Check your thinking when you're down. Is there evidence to support your beliefs? Is there evidence that contradicts your beliefs? Be realistic!			Take a look at My Charts in the Wellness Plan. Do you see any patterns?<br><br>Take the time to recover…

Daily Review Feedback Category 6: Episode Improving, Down

Continue (P5)	Consider (S1)	Engage mind (U10)			My Skills (P5)
**1**<br>**RANDOM UNLINKED**	**2**<br>**STATIC**	**3**<br>**RANDOM UNLINKED**	**4**<br>**BLANK**	**5**<br>**BLANK**	**6**<br>**RANDOM UNLINKED**
You're reporting less intense symptoms today. That's great news.<br><br>Whatever you are doing to help you get better, stick with it. Remember, one step at a time.<br><br>Continue for additional ideas…	Things to do:<br><br>Keep up the efforts to get and stay well. In addition to whatever you have been doing that is helpful, consider the following:<br><br>✓✓ Continue working with your psychiatrist.<br><br>✓✓ Continue working with your supports.<br><br>✓✓ Keep using coping skills.<br><br>✓✓ Keep using lifestyle skills.	TIP OF THE DAY<br><br>Depression is associated with problems concentrating. This can be very frustrating.<br><br>Is this happening to you right now?<br><br>Take action to engage your mind. This is easier said than done! But waken your mind:<br><br>• Write in a journal.<br>• Talk to someone.<br>• Do a crossword puzzle.<br>• Read a short news article.			Take a look at My Skills in the Wellness Plan. Consider trying out a skill you selected as a preferred skill.<br><br>Take the time to recover…

Daily Review Feedback Category 7: Episode Improving, Balanced

Page 1<br>Random Unlinked<br>5 permutations	Page 2<br>Static	Page 3<br>Random Unlinked<br>5 unique	Page 4<br>Blank	Page 5<br>Blank	Page 6<br>Random Unlinked<br>5 permutations

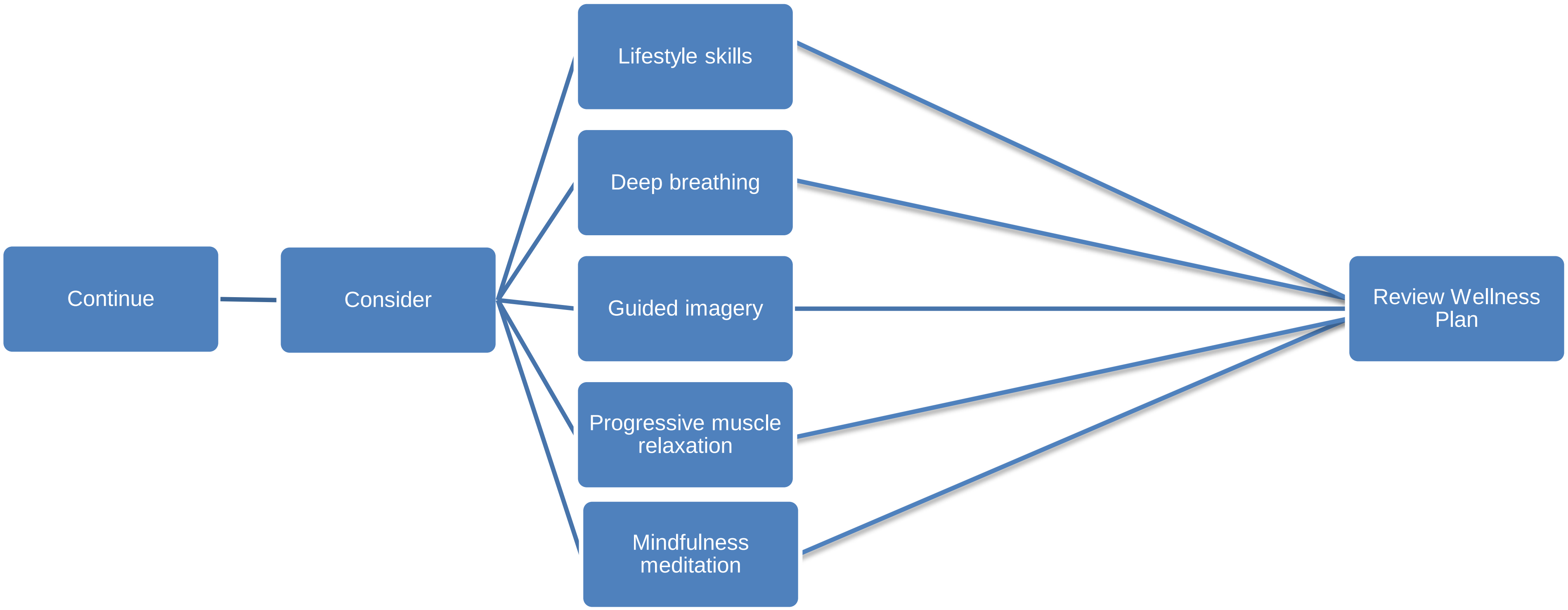

Daily Review Feedback Category 7: Episode Improving, Balanced

Continue (P6)	Consider (S1)	Lifestyle skills (U11)			A & A (P9)
**1**<br>**RANDOM UNLINKED**	**2**<br>**STATIC**	**3**<br>**RANDOM UNLINKED**	**4**<br>**BLANK**	**5**<br>**BLANK**	**6**<br>**RANDOM UNLINKED**
You are reporting feeling balanced today. It's great that you're feeling better.<br><br>Keep doing whatever you have been doing. It seems to be working!<br><br>Continue for additional ideas...	Things to do:<br><br>Keep up the efforts to get and stay well. In addition to whatever you have been doing that is helpful, consider the following:<br><br>✓✓ Continue working with your psychiatrist.<br><br>✓✓ Continue working with your supports.<br><br>✓✓ Keep using coping skills.<br><br>✓✓ Keep using lifestyle skills.	TIP OF THE DAY<br><br>Take 10 minutes today to practice a Lifestyle skill.<br><br>• Problem solving<br>• Bird's eye view<br>• Deep breathing<br>• Muscle relaxation<br>• Self---soothing<br>• Improve the moment<br>• Mindfulness<br>• Radical acceptance<br><br>If you don't remember them, you can look them up in Skills.			Review your ideas for Balanced in Awareness & Action in the Wellness Plan.<br><br>Get well!

Daily Review Feedback Category 7: Episode Improving, Balanced

Continue (P7)	Consider (S1)	Deep breathing (U12)			A & A (P10)
**1**<br>**RANDOM UNLINKED**	**2**<br>**STATIC**	**3**<br>**RANDOM UNLINKED**	**4**<br>**BLANK**	**5**<br>**BLANK**	**6**<br>**RANDOM UNLINKED**
Glad to see you're doing better. Keep up the good work!<br><br>Continue for tips to stay on track…	Things to do:<br><br>Keep up the efforts to get and stay well. In addition to whatever you have been doing that is helpful, consider the following:<br><br>✓✓ Continue working with your psychiatrist.<br><br>✓✓ Continue working with your supports.<br><br>✓✓ Keep using coping skills.<br><br>✓✓ Keep using lifestyle skills.	TIP OF THE DAY<br><br>Take 10 minutes for yourself. Get grounded to stay balanced.<br><br>Try deep breathing.<br><br>Your diaphragm is the muscle that controls breathing. It is located just below your lungs. Using your diaphragm to its fullest is calming.<br><br>Breathe by using your belly rather than your chest. This is the best way.<br><br>Take slow, deep, belly breaths.			Take a look at how you described yourself when Balanced in Awareness & Action. Go to the Wellness Plan now to review your anchors.<br><br>Stay well…

Daily Review Feedback Category 7: Episode Improving, Balanced

Continue (P8)	Consider (S1)	Guided imagery (U13)			A & A (P11)
1<br>**RANDOM UNLINKED**	2<br>**STATIC**	3<br>**RANDOM UNLINKED**	4<br>**BLANK**	5<br>**BLANK**	6<br>**RANDOM UNLINKED**
Good to see that you're experiencing less intense symptoms.<br><br>Is there anything in particular you think has helped? If so, stick with it!<br><br>Continue for additional ideas…	Things to do:<br><br>Keep up the efforts to get and stay well. In addition to whatever you have been doing that is helpful, consider the following:<br><br>✓✓ Continue working with your psychiatrist.<br><br>✓✓ Continue working with your supports.<br><br>✓✓ Keep using coping skills.<br><br>✓✓ Keep using lifestyle skills.	TIP OF THE DAY<br><br>Take 10 minutes for yourself. Get grounded to stay balanced.<br><br>Go on YouTube and search for <u>Guided Imagery</u> videos. Pick one and participate.<br><br>Notice how it makes you feel.			Take a look at your action plan for Balanced in Awareness & Action. Go to the Wellness Plan to review your Plan.<br><br>Stay well…

Daily Review Feedback Category 7: Episode Improving, Balanced

Continue (P9)	Consider (S1)	Relaxation (U14)			Reduce Risk (P12)
**1**<br>**RANDOM UNLINKED**	**2**<br>**STATIC**	**3**<br>**RANDOM UNLINKED**	**4**<br>**BLANK**	**5**<br>**BLANK**	**6**<br>**RANDOM UNLINKED**
It's great that your symptoms are improving and that you're doing better.<br><br>Continue to stay on track…	Things to do:<br><br>Keep up the efforts to get and stay well. In addition to whatever you have been doing that is helpful, consider the following:<br><br>✓✓ Continue working with your psychiatrist.<br><br>✓✓ Continue working with your supports.<br><br>✓✓ Keep using coping skills.<br><br>✓✓ Keep using lifestyle skills.	TIP OF THE DAY<br><br>Take 10 minutes for yourself. Get grounded to stay balanced.<br><br>Go on YouTube and search for Progressive Muscle Relaxation videos. Pick one and participate.<br><br>Notice how it makes you feel.			Take a look at your Lifestyle Skills under Reduce Risk in the Wellness Plan.<br><br>Keep the commitment you made to yourself!

Daily Review Feedback Category 7: Episode Improving, Balanced

Continue (P10) | Consider (S1) | Mindfulness (U15) | My Charts (P4)

1<br>RANDOM UNLINKED	2<br>STATIC	3<br>RANDOM UNLINKED	4<br>BLANK	5<br>BLANK	6<br>RANDOM UNLINKED
Looks like things are headed in the right direction for you. Keep the momentum going, one step at a time.<br><br>Press continue for tips.	Things to do:<br><br>Keep up the efforts to get and stay well. In addition to whatever you have been doing that is helpful, consider the following:<br><br>✓✓ Continue working with your psychiatrist.<br><br>✓✓ Continue working with your supports.<br><br>✓✓ Keep using coping skills.<br><br>✓✓ Keep using lifestyle skills.	TIP OF THE DAY<br><br>Take 10 minutes for yourself. Get grounded to stay balanced.<br><br>Go on YouTube and search for Mindfulness Meditation videos. Pick one and participate.<br><br>Notice how it makes you feel.			Take a look at My Charts in the Wellness Plan. Do you see any patterns?<br><br>Take the time to recover…

Daily Review Feedback Category 8: Worsening Symptoms, Up – Psychiatrist (Choice 1.0)

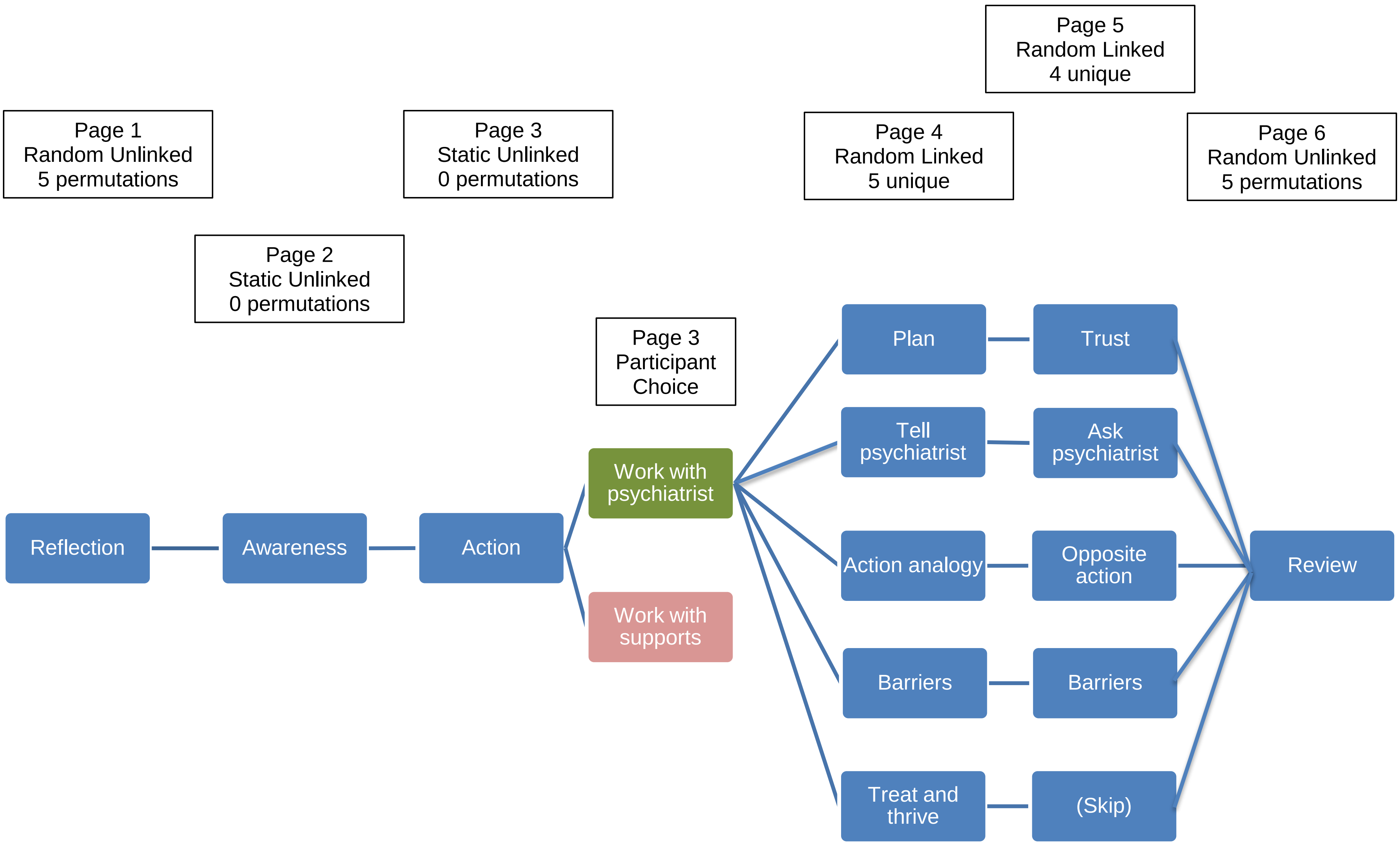

Daily Review Feedback Category 8: Worsening Symptoms, Up – Psychiatrist (Choice 1.0)

Reflection (P1)	Awareness (S1)	Action (S1)	Plan (U1)	Trust (U1)	A & A (P1)
**1**<br>**RANDOM UNLINKED**	**2**<br>**STATIC**	**3**<br>**STATIC**	**4**<br>**RANDOM LINKED**	**5**<br>**RANDOM LINKED**	**6**<br>**RANDOM UNLINKED**
You're saying that you are Moderate Up. Seems like a change from how you've been doing overall.<br><br>Now is the time for awareness and action<br><br>Continue for ideas…	AWARENESS<br><br>Check it out. Are you having any of the following symptoms?<br><br>☐ Overly happy/excited/irritable<br>☐ Increased esteem/feeling superior<br>☐ Decreased need for sleep<br>☐ More talkative than usual<br>☐ Racing thoughts<br>☐ Difficulties concentrating<br>☐ Increased activity level<br>☐ Risky activities<br><br>If you are experiencing several of these symptoms daily, you are likely experiencing mania.	ACTION<br><br>✓✓ Call your psychiatrist if they do not already know about any changes in how you are doing.<br><br>✓✓ Let your supports know that you seem to be experiencing mania.<br><br>✓✓ Use dial down skills. Slow down, cool down, get less active, calm your body.<br><br>✓✓ Keep taking your medications and make sure you are getting enough sleep.	WORKING WITH YOUR PSYCHIATRIST<br><br>1. Reach out. Call them now. Let them know about your symptoms.<br>2. Agree upon a plan of action. This might involve a medication change. If you have any reservations about the plan, let them know. Don't end the call agreeing to something that you might not feel comfortable doing.<br>3. Implement the plan. Evaluate each day whether or not the plan is working.<br>4. Call your psychiatrist again if you symptoms continue and/or you cannot implement the plan.	REMEMBER<br><br>It takes a team to manage bipolar disorder. So trust your psychiatrist in this time of urgency.<br><br>The most important thing right now is for you to get well. You want to keep your life on track! Your home, relationships, and job…<br><br>Take good care of yourself!	Check out your plan for Moderate Up under Awareness & Action in the Wellness Plan.<br><br>Get well!

Daily Review Feedback Category 8: Worsening Symptoms, Up – Psychiatrist (Choice 1.0)

Reflection (P2)	Awareness (S1)	Action (S1)	Tell (U2)	Ask (U2)	A & A (P2)
**1**<br>**RANDOM UNLINKED**	**2**<br>**STATIC**	**3**<br>**STATIC**	**4**<br>**RANDOM LINKED**	**5**<br>**RANDOM LINKED**	**6**<br>**RANDOM UNLINKED**
Looks like there has been a change in your wellness.<br><br>Now is the time to take a closer look at things.<br><br>Continue for ideas…	AWARENESS<br><br>Check it out. Are you having any of the following symptoms?<br><br>☐ Overly happy/excited/irritable<br>☐ Increased esteem/feeling superior<br>☐ Decreased need for sleep<br>☐ More talkative than usual<br>☐ Racing thoughts<br>☐ Difficulties concentrating<br>☐ Increased activity level<br>☐ Risky activities<br><br>If you are experiencing several of these symptoms daily, you are likely experiencing mania.	ACTION<br><br>✓✓ Call your psychiatrist if they do not already know about any changes in how you are doing.<br><br>✓✓ Let your supports know that you seem to be experiencing mania.<br><br>✓✓ Use dial down skills. Slow down, cool down, get less active, calm your body.<br><br>✓✓ Keep taking your medications and make sure you are getting enough sleep.	WORKING WITH YOUR PSYCHIATRIST<br><br>Things to share with your psychiatrist:<br><br>• How you are taking your medications<br>• How much and when you are sleeping<br>• How you are spending your time<br>• Any alcohol or drug use	WORKING WITH YOUR PSYCHIATRIST<br><br>Things to ask your psychiatrist:<br><br>• Why do they think you're having symptoms<br>• What do they think you should do<br>• When you should follow--- up with them	Go to the Wellness Plan to check out your anchors for Moderate Up in Awareness & Action.<br><br>Get well!

Daily Review Feedback Category 8: Worsening Symptoms, Up – Psychiatrist (Choice 1.0)

Reflection (P3)	Awareness (S1)	Action (S1)	Analogy (U3)	Opposite (U3)	A & A (P3)
**1**<br>**RANDOM UNLINKED**	**2**<br>**STATIC**	**3**<br>**STATIC**	**4**<br>**RANDOM LINKED**	**5**<br>**RANDOM LINKED**	**6**<br>**RANDOM UNLINKED**
Seems like your mood is elevated today and that you may be experiencing symptoms of mania.<br><br>Check in with yourself. See what you might need to do.<br><br>Continue for ideas…	AWARENESS<br><br>Check it out. Are you having any of the following symptoms?<br><br>☐ Overly happy/excited/irritable<br>☐ Increased esteem/feeling superior<br>☐ Decreased need for sleep<br>☐ More talkative than usual<br>☐ Racing thoughts<br>☐ Difficulties concentrating<br>☐ Increased activity level<br>☐ Risky activities<br><br>If you are experiencing several of these symptoms daily, you are likely experiencing mania.	ACTION<br><br>✓✓ Call your psychiatrist if they do not already know about any changes in how you are doing.<br><br>✓✓ Let your supports know that you seem to be experiencing mania.<br><br>✓✓ Use dial down skills. Slow down, cool down, get less active, calm your body.<br><br>✓✓ Keep taking your medications and make sure you are getting enough sleep.	Keep your eye on the prize!<br><br>Remember that bipolar disorder is recurrent. That means symptoms are likely to occur from time to time.<br><br>While that is a problem, the most important thing is what you do when you have a recurrence!<br><br>Take swift action. Get rid of the symptoms. Minimize the impact of symptoms on your life.<br><br>It's like getting the flu. Rest and good nutrition can help a speedy recovery.<br><br>Take your medications, work with your team, and dial down.	Easier said than done?<br><br>Right! It is really hard to act in opposition to your mood state.<br><br>There are two factors involved:<br><br>1. <u>Motivation</u><br>When feeling up, unless you're irritable, it feels good. Remember that symptoms are not good for your brain or your life.<br><br>2. <u>Action opposite mood</u><br>Your state of mind influences your behavior. That is, feeling up brings about activity. To slow down, you must deliberately override this process. It's tricky.	Check out what you said you would do when Moderate Up in Awareness & Action in the Wellness Plan.<br><br>Get well!

Daily Review Feedback Category 8: Worsening Symptoms, Up – Psychiatrist (Choice 1.0)

Reflection (P4)	Awareness (S1)	Action (S1)	Barriers (U4)	Barriers (U4)	My Charts (P4)
**1**<br>**RANDOM UNLINKED**	**2**<br>**STATIC**	**3**<br>**STATIC**	**4**<br>**RANDOM LINKED**	**5**<br>**RANDOM LINKED**	**6**<br>**RANDOM UNLINKED**
You're reporting a shift in your symptom status.<br><br>Take a minute to reflect and problem solve<br><br>Continue for ideas…	AWARENESS<br><br>Check it out. Are you having any of the following symptoms?<br><br>☐ Overly happy/excited/irritable<br>☐ Increased esteem/feeling superior<br>☐ Decreased need for sleep<br>☐ More talkative than usual<br>☐ Racing thoughts<br>☐ Difficulties concentrating<br>☐ Increased activity level<br>☐ Risky activities<br><br>If you are experiencing several of these symptoms daily, you are likely experiencing mania.	ACTION<br><br>✓✓ Call your psychiatrist if they do not already know about any changes in how you are doing.<br><br>✓✓ Let your supports know that you seem to be experiencing mania.<br><br>✓✓ Use dial down skills. Slow down, cool down, get less active, calm your body.<br><br>✓✓ Keep taking your medications and make sure you are getting enough sleep.	Overcoming Barriers<br><br>Is something getting in the way of reaching out to your psychiatrist?<br><br>Are you embarrassed because you didn't follow their recommendations and now you're having symptoms?<br><br>Or maybe you used drugs or alcohol and don't want to tell them?<br><br>Do you think you can manage these symptoms on your own?<br><br>Are you not wanting to bother them because it's a weekend or for some other reason?	Overcoming Barriers<br><br>If you are reluctant to contact your psychiatrist, make a list of reasons.<br><br>Write out an alternative rational response for each reason you wrote down. For example, if you're embarrassed you might write "Nobody is perfect. I own and accept that I didn't take my medications for a week. I will proceed ahead and take care of my health."<br><br>Also consider running your concerns by your supports. See what they have to say.	Check out My Charts in the Wellness Plan. Do you see any patterns?<br><br>Get well!

Daily Review Feedback Category 8: Worsening Symptoms, Up – Psychiatrist (Choice 1.0)

Reflection (P5)	Awareness (S1)	Action (S1)	Thrive (U5)	(U5)	My Skills (P5)
**1**<br>**RANDOM UNLINKED**	**2**<br>**STATIC**	**3**<br>**STATIC**	**4**<br>**RANDOM LINKED**	**5**<br>**RANDOM LINKED**	**6**<br>**RANDOM UNLINKED**
You're rating yourself as Moderately Up. It appears that you may be experiencing symptoms typical of mania.<br><br>Take a few minutes and check in with yourself.<br><br>Continue for ideas…	AWARENESS<br><br>Check it out. Are you having any of the following symptoms?<br><br>☐ Overly happy/excited/irritable<br>☐ Increased esteem/feeling superior<br>☐ Decreased need for sleep<br>☐ More talkative than usual<br>☐ Racing thoughts<br>☐ Difficulties concentrating<br>☐ Increased activity level<br>☐ Risky activities<br><br>If you are experiencing several of these symptoms daily, you are likely experiencing mania.	ACTION<br><br>✓✓ Call your psychiatrist if they do not already know about any changes in how you are doing.<br><br>✓✓ Let your supports know that you seem to be experiencing mania.<br><br>✓✓ Use dial down skills. Slow down, cool down, get less active, calm your body.<br><br>✓✓ Keep taking your medications and make sure you are getting enough sleep.	Clinician's Corner…<br><br>I have worked with many individuals living with and living beyond bipolar disorder over the years.<br><br>The challenges faced are huge. I see the problems. I see the havoc. I also see the strength and determination involved in overcoming symptoms and getting well.<br><br>Be honest with yourself, accept your strengths and weaknesses, treat your illness, and live a life with meaning. You can thrive!<br><br>------Dr. Cynthia Dopke	(Skip)	Try out one of My Skills in My Resources in the Wellness Plan.<br><br>Get well!

Daily Review Feedback Category 8: Worsening Symptoms, Up – Supports (Choice 2.0)

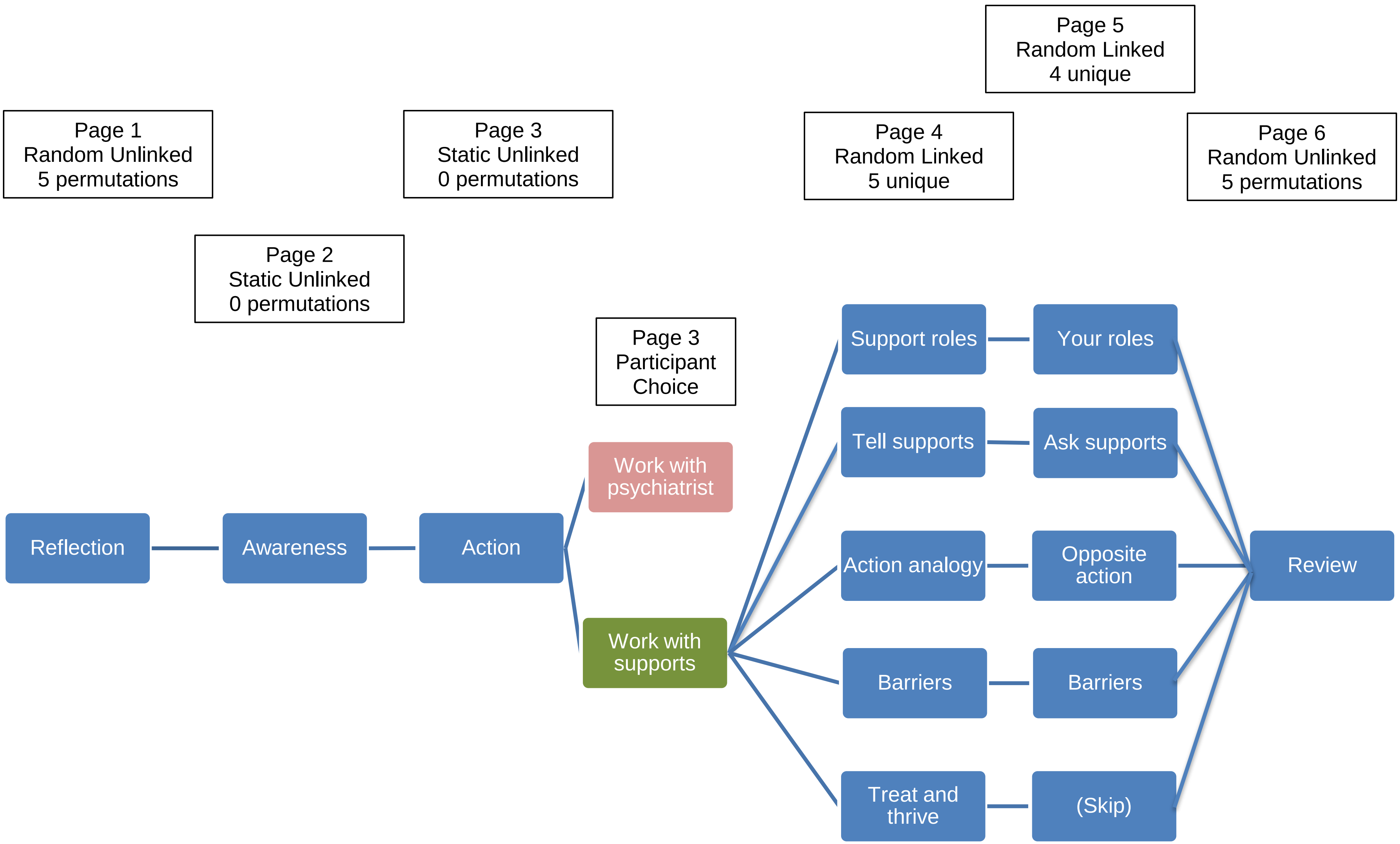

Daily Review Feedback Category 8: Worsening Symptoms, Up – Supports (Choice 2.0)

Reflection (P1)	Awareness (S1)	Action (S1)	Roles (U6)	Roles (U6)	A & A (P6)
**1**<br>**RANDOM UNLINKED**	**2**<br>**STATIC**	**3**<br>**STATIC**	**4**<br>**RANDOM LINKED**	**5**<br>**RANDOM LINKED**	**6**<br>**RANDOM UNLINKED**
You're saying that you are Moderate Up. Seems like a change from how you've been doing overall.<br><br>Now is the time for awareness and action<br><br>Continue for ideas…	AWARENESS<br><br>Check it out. Are you having any of the following symptoms?<br><br>☐ Overly happy/excited/irritable<br>☐ Increased esteem/feeling superior<br>☐ Decreased need for sleep<br>☐ More talkative than usual<br>☐ Racing thoughts<br>☐ Difficulties concentrating<br>☐ Increased activity level<br>☐ Risky activities<br><br>If you are experiencing several of these symptoms daily, you are likely experiencing mania.	ACTION<br><br>✓✓ Call your psychiatrist if they do not already know about any changes in how you are doing.<br><br>✓✓ Let your supports know that you seem to be experiencing mania.<br><br>✓✓ Use dial down skills. Slow down, cool down, get less active, calm your body.<br><br>✓✓ Keep taking your medications and make sure you are getting enough sleep.	WORKING WITH SUPPORTS<br><br>Your support's role…<br><br>Before starting a conversation with your supports, help them to know the role that you would like them to play. You could say:<br>"I'd like you to simply be here for me when I have symptoms. You are not responsible for me. Encourage me to follow my Wellness Plan. Here's some:<br>Dos:<br>• Listen and encourage me<br>• Give honest but gentle feedback<br>Don'ts:<br>• Panic<br>• Get critical<br>• Tell me what to do<br>• Try to solve my problems"	WORKING WITH SUPPORTS<br><br>Your role…<br><br>• Reach out. Let your supports know that you are having symptoms.<br><br>• Let them know what you are going to do to manage your symptoms (e.g., calling your psychiatrist, using skills, taking medications).<br><br>• Agree on a time when you will check in again.<br><br>• Ask them to give you gentle feedback if they think your condition is getting worse.	Check out your anchors for Moderate Up in Awareness & Action.<br><br>Get well!

Daily Review Feedback Category 8: Worsening Symptoms, Up – Supports (Choice 2.0)

Reflection (P2)	Awareness (S1)	Action (S1)	Tell (U7)	Ask (U7)	A & A (P2)
**1**<br>**RANDOM UNLINKED**	**2**<br>**STATIC**	**3**<br>**STATIC**	**4**<br>**RANDOM LINKED**	**5**<br>**RANDOM LINKED**	**6**<br>**RANDOM UNLINKED**
Looks like there has been a change in your wellness.<br><br>Now is the time to take a closer look at things.<br><br>Continue for ideas…	AWARENESS<br><br>Check it out. Are you having any of the following symptoms?<br><br>☐ Overly happy/excited/irritable<br>☐ Increased esteem/feeling superior<br>☐ Decreased need for sleep<br>☐ More talkative than usual<br>☐ Racing thoughts<br>☐ Difficulties concentrating<br>☐ Increased activity level<br>☐ Risky activities<br><br>If you are experiencing several of these symptoms daily, you are likely experiencing mania.	ACTION<br><br>✓✓ Call your psychiatrist if they do not already know about any changes in how you are doing.<br><br>✓✓ Let your supports know that you seem to be experiencing mania.<br><br>✓✓ Use dial down skills. Slow down, cool down, get less active, calm your body.<br><br>✓✓ Keep taking your medications and make sure you are getting enough sleep.	WORKING WITH SUPPORTS<br><br>Things to share with your supports:<br><br>• Your symptoms<br>• Actions you have taken to manage your symptoms<br>• What your psychiatrist advised	WORKING WITH SUPPORTS<br><br>Things to ask of your supports:<br><br>• How severe do your symptoms appear to be<br>• What do they think you should do (what has worked in the past)<br>• When you should follow--- up with them	Go to the Wellness Plan to check out your anchors for Moderate Up in Awareness & Action.<br><br>Get well!

Daily Review Feedback Category 8: Worsening Symptoms, Up – Supports (Choice 2.0)

Reflection (P3)	Awareness (S1)	Action (S1)	Analogy (U3)	Opposite (U3)	A & A (P3)
**1**<br>**RANDOM UNLINKED**	**2**<br>**STATIC**	**3**<br>**STATIC**	**4**<br>**RANDOM LINKED**	**5**<br>**RANDOM LINKED**	**6**<br>**RANDOM UNLINKED**
Seems like your mood is elevated today and that you may be experiencing symptoms of mania.<br><br>Check in with yourself. See what you might need to do.<br><br>Continue for ideas…	AWARENESS<br><br>Check it out. Are you having any of the following symptoms?<br><br>☐ Overly happy/excited/irritable<br>☐ Increased esteem/feeling superior<br>☐ Decreased need for sleep<br>☐ More talkative than usual<br>☐ Racing thoughts<br>☐ Difficulties concentrating<br>☐ Increased activity level<br>☐ Risky activities<br><br>If you are experiencing several of these symptoms daily, you are likely experiencing mania.	ACTION<br><br>✓✓ Call your psychiatrist if they do not already know about any changes in how you are doing.<br><br>✓✓ Let your supports know that you seem to be experiencing mania.<br><br>✓✓ Use dial down skills. Slow down, cool down, get less active, calm your body.<br><br>✓✓ Keep taking your medications and make sure you are getting enough sleep.	Keep your eye on the prize!<br><br>Remember that bipolar disorder is recurrent. That means symptoms are likely to occur from time to time.<br><br>While that is a problem, the most important thing is what you do when you have a recurrence!<br><br>Take swift action. Get rid of the symptoms. Minimize the impact of symptoms on your life.<br><br>It's like getting the flu. Rest and good nutrition can help a speedy recovery.<br><br>Take your medications, work with your team, and dial down.	Easier said than done?<br><br>Right! It is really hard to act in opposition to your mood state.<br><br>There are two factors involved:<br><br>1. Motivation<br>When feeling up, unless you're irritable, it feels good. Remember that symptoms are not good for your brain or your life.<br><br>2. Action opposite mood<br>Your state of mind influences your behavior. That is, feeling up brings about activity. To slow down, you must deliberately override this process. It's tricky.	Check out what you said you would do when Moderate Up in Awareness & Action in the Wellness Plan.<br><br>Get well!

Daily Review Feedback Category 8: Worsening Symptoms, Up – Supports (Choice 2.0)

Reflection (P4)	Awareness (S1)	Action (S1)	Barriers (U8)	Barriers (U8)	My Charts (P4)
**1**<br>**RANDOM UNLINKED**	**2**<br>**STATIC**	**3**<br>**STATIC**	**4**<br>**RANDOM LINKED**	**5**<br>**RANDOM LINKED**	**6**<br>**RANDOM UNLINKED**
You're reporting a shift in your symptom status.<br><br>Take a minute to reflect and problem solve<br><br>Continue for ideas…	AWARENESS<br><br>Check it out. Are you having any of the following symptoms?<br><br>☐ Overly happy/excited/irritable<br>☐ Increased esteem/feeling superior<br>☐ Decreased need for sleep<br>☐ More talkative than usual<br>☐ Racing thoughts<br>☐ Difficulties concentrating<br>☐ Increased activity level<br>☐ Risky activities<br><br>If you are experiencing several of these symptoms daily, you are likely experiencing mania.	ACTION<br><br>✓✓ Call your psychiatrist if they do not already know about any changes in how you are doing.<br><br>✓✓ Let your supports know that you seem to be experiencing mania.<br><br>✓✓ Use dial down skills. Slow down, cool down, get less active, calm your body.<br><br>✓✓ Keep taking your medications and make sure you are getting enough sleep.	Overcoming Barriers<br><br>Is something getting in the way of reaching out to your supports?<br><br>Are you afraid they will tell you something you don't want to hear?<br><br>Are you afraid they will worry or get overbearing somehow?<br><br>Do you think you can manage these symptoms on your own?<br><br>Are you not wanting to bother them for some reason?	Overcoming Barriers<br><br>If you are reluctant to contact your supports, make a list of reasons.<br><br>Write out an alternative rational response for each reason you wrote down. For example, if you're concerned they will get overbearing, you might write "I can ask them to call me no more than once per day."<br><br>Also consider running your concerns by your psychiatrist. See what they have to say.	Check out My Charts in the Wellness Plan. Do you see any patterns?<br><br>Get well!

Daily Review Feedback Category 8: Worsening Symptoms, Up – Supports (Choice 2.0)

Reflection (P5)	Awareness (S1)	Action (S1)	Thrive (U5)	(U5)	My Skills (P5)
**1**<br>**RANDOM UNLINKED**	**2**<br>**STATIC**	**3**<br>**STATIC**	**4**<br>**RANDOM LINKED**	**5**<br>**RANDOM LINKED**	**6**<br>**RANDOM UNLINKED**
You're rating yourself as Moderately Up. It appears that you may be experiencing symptoms typical of mania.<br><br>Take a few minutes and check in with yourself.<br><br>Continue for ideas…	AWARENESS<br><br>Check it out. Are you having any of the following symptoms?<br><br>☐ Overly happy/excited/irritable<br>☐ Increased esteem/feeling superior<br>☐ Decreased need for sleep<br>☐ More talkative than usual<br>☐ Racing thoughts<br>☐ Difficulties concentrating<br>☐ Increased activity level<br>☐ Risky activities<br><br>If you are experiencing several of these symptoms daily, you are likely experiencing mania.	ACTION<br><br>✓✓ Call your psychiatrist if they do not already know about any changes in how you are doing.<br><br>✓✓ Let your supports know that you seem to be experiencing mania.<br><br>✓✓ Use dial down skills. Slow down, cool down, get less active, calm your body.<br><br>✓✓ Keep taking your medications and make sure you are getting enough sleep.	Clinician's Corner…<br><br>I have worked with many individuals living with and living beyond bipolar disorder over the years.<br><br>The challenges faced are huge. I see the problems. I see the havoc. I also see the strength and determination involved in overcoming symptoms and getting well.<br><br>Be honest with yourself, accept your strengths and weaknesses, treat your illness, and live a life with meaning. You can thrive!<br><br>------Dr. Cynthia Dopke	(Skip)	Try out one of My Skills in My Resources in the Wellness Plan.<br><br>Get well!

Daily Review Feedback Category 9: Worsening Symptoms, Down – Psychiatrist (Choice 1.0)

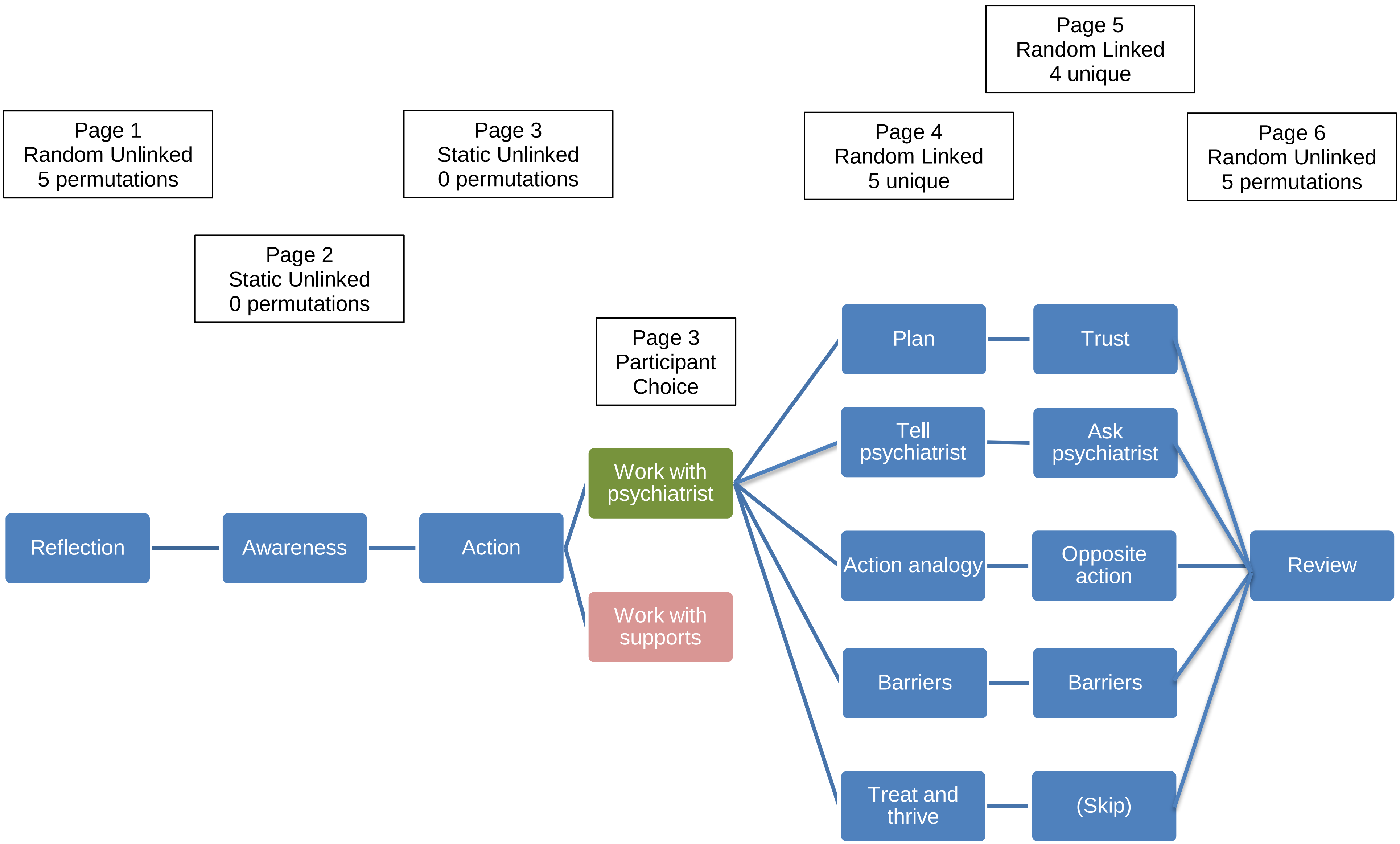

Daily Review Feedback Category 9: Worsening Symptoms, Down – Psychiatrist (Choice 1.0)

Reflection (P6)	Awareness (S2)	Action (S2)	Plan (U1)	Trust (U1)	A & A (P7)
**1**<br>**RANDOM UNLINKED**	**2**<br>**STATIC**	**3**<br>**STATIC**	**4**<br>**RANDOM LINKED**	**5**<br>**RANDOM LINKED**	**6**<br>**RANDOM UNLINKED**
You're saying that you are Moderately Down. Seems like a change from how you've been doing overall.<br><br>Now is the time for awareness and action<br><br>Continue for ideas…	AWARENESS<br><br>Check it out. Are you having any of the following symptoms?<br><br>• Low mood<br>• Reduced interest and pleasure<br>• Weight loss or weight gain<br>• Sleeping too much or too little<br>• Physical agitation or slowing down<br>• Fatigue or loss of energy<br>• Feeling worthless or guilty<br>• Difficulty concentrating<br>• Thoughts of death or suicide<br><br>If you are experiencing several of these symptoms, you are likely experiencing depression	ACTION<br><br>✓✓ Call your psychiatrist if they do not already know about any changes in how you are doing.<br><br>✓✓ Let your supports know that you seem to be experiencing depression.<br><br>✓✓ Use dial up skills. Start moving, warm up, get more active, stay involved.<br><br>✓✓ Keep taking your medications and make sure you are getting proper sleep.	WORKING WITH YOUR PSYCHIATRIST<br><br>1. Reach out. Call them now. Let them know about your symptoms.<br>2. Agree upon a plan of action. This might involve a medication change. If you have any reservations about the plan, let them know. Don't end the call agreeing to something that you might not feel comfortable doing.<br>3. Implement the plan. Evaluate each day whether or not the plan is working.<br>4. Call your psychiatrist again if you symptoms continue and/or you cannot implement the plan.	REMEMBER<br><br>It takes a team to manage bipolar disorder. So trust your psychiatrist in this time of urgency.<br><br>The most important thing right now is for you to get well. You want to keep your life on track! Your home, relationships, and job…<br><br>Take good care of yourself!	Check out your plan for Moderate Down under Awareness & Action in the Wellness Plan.<br><br>Get well!

Daily Review Feedback Category 9: Worsening Symptoms, Down – Psychiatrist (Choice 1.0)

Reflection (P2)	Awareness (S2)	Action (S2)	Tell (U2)	Ask (U2)	A & A (P8)
**1**<br>**RANDOM UNLINKED**	**2**<br>**STATIC**	**3**<br>**STATIC**	**4**<br>**RANDOM LINKED**	**5**<br>**RANDOM LINKED**	**6**<br>**RANDOM UNLINKED**
Looks like there has been a change in your wellness.<br><br>Now is the time to take a closer look at things.<br><br>Continue for ideas…	AWARENESS<br><br>Check it out. Are you having any of the following symptoms?<br><br>• Low mood<br>• Reduced interest and pleasure<br>• Weight loss or weight gain<br>• Sleeping too much or too little<br>• Physical agitation or slowing down<br>• Fatigue or loss of energy<br>• Feeling worthless or guilty<br>• Difficulty concentrating<br>• Thoughts of death or suicide<br><br>If you are experiencing several of these symptoms, you are likely experiencing depression	ACTION<br><br>✓✓ Call your psychiatrist if they do not already know about any changes in how you are doing.<br><br>✓✓ Let your supports know that you seem to be experiencing depression.<br><br>✓✓ Use dial up skills. Start moving, warm up, get more active, stay involved.<br><br>✓✓ Keep taking your medications and make sure you are getting proper sleep.	WORKING WITH YOUR PSYCHIATRIST<br><br>Things to share with your psychiatrist:<br><br>• How you are taking your medications<br>• How much and when you are sleeping<br>• How you are spending your time<br>• Any alcohol or drug use	WORKING WITH YOUR PSYCHIATRIST<br><br>Things to ask your psychiatrist:<br><br>• Why do they think you're having symptoms<br>• What do they think you should do<br>• When you should follow--- up with them	Check out your anchors for Moderate Down under Awareness & Action in the Wellness Plan.<br><br>Get well!

Daily Review Feedback Category 9: Worsening Symptoms, Down – Psychiatrist (Choice 1.0)

Reflection (P7)	Awareness (S2)	Action (S2)	Analogy (U3)	Opposite (U9)	A & A (P9)
**1**<br>**RANDOM UNLINKED**	**2**<br>**STATIC**	**3**<br>**STATIC**	**4**<br>**RANDOM LINKED**	**5**<br>**RANDOM LINKED**	**6**<br>**RANDOM UNLINKED**
Seems like your mood has dropped and that you may be experiencing symptoms of depression.<br><br>Check in with yourself. See what you might need to do.<br><br>Continue for ideas…	AWARENESS<br><br>Check it out. Are you having any of the following symptoms?<br><br>• Low mood<br>• Reduced interest and pleasure<br>• Weight loss or weight gain<br>• Sleeping too much or too little<br>• Physical agitation or slowing down<br>• Fatigue or loss of energy<br>• Feeling worthless or guilty<br>• Difficulty concentrating<br>• Thoughts of death or suicide<br><br>If you are experiencing several of these symptoms, you are likely experiencing depression	ACTION<br><br>✓✓ Call your psychiatrist if they do not already know about any changes in how you are doing.<br><br>✓✓ Let your supports know that you seem to be experiencing depression.<br><br>✓✓ Use dial up skills. Start moving, warm up, get more active, stay involved.<br><br>✓✓ Keep taking your medications and make sure you are getting proper sleep.	Keep your eye on the prize!<br><br>Remember that bipolar disorder is recurrent. That means symptoms are likely to occur from time to time.<br><br>While that is a problem, the most important thing is what you do when you have a recurrence!<br><br>Take swift action. Get rid of the symptoms. Minimize the impact of symptoms on your life.<br><br>It's like getting the flu. Rest and good nutrition can help a speedy recovery.<br><br>Take your medications, work with your team, and dial down.	Easier said than done?<br><br>Right! It is really hard to act in opposition to your mood state.<br><br>There are two factors involved:<br><br>1. <u>Motivation</u> Lack of motivation is part of being depressed. So the very thing you need to take care of yourself is missing! So you have to find a way to act despite not wanting to!<br><br>2. <u>Action opposite mood</u> Your state of mind influences your behavior. That is, feeling down reduces activity. To speed up you must deliberately override this process. It's tricky.	Check out what you said you would do when Moderate Down under Awareness & Action in the Wellness Plan.<br><br>Get well!

Daily Review Feedback Category 9: Worsening Symptoms, Down – Psychiatrist (Choice 1.0)

Reflection (P4)	Awareness (S2)	Action (S2)	Barriers (U4)	Barriers (U4)	My Charts (P4)
**1**<br>**RANDOM UNLINKED**	**2**<br>**STATIC**	**3**<br>**STATIC**	**4**<br>**RANDOM LINKED**	**5**<br>**RANDOM LINKED**	**6**<br>**RANDOM UNLINKED**
You're reporting a shift in your symptom status.<br><br>Take a minute to reflect and problem solve<br><br>Continue for ideas…	AWARENESS<br><br>Check it out. Are you having any of the following symptoms?<br><br>• Low mood<br>• Reduced interest and pleasure<br>• Weight loss or weight gain<br>• Sleeping too much or too little<br>• Physical agitation or slowing down<br>• Fatigue or loss of energy<br>• Feeling worthless or guilty<br>• Difficulty concentrating<br>• Thoughts of death or suicide<br><br>If you are experiencing several of these symptoms, you are likely experiencing depression	ACTION<br><br>✓✓ Call your psychiatrist if they do not already know about any changes in how you are doing.<br><br>✓✓ Let your supports know that you seem to be experiencing depression.<br><br>✓✓ Use dial up skills. Start moving, warm up, get more active, stay involved.<br><br>✓✓ Keep taking your medications and make sure you are getting proper sleep.	Overcoming Barriers<br><br>Is something getting in the way of reaching out to your psychiatrist?<br><br>Are you embarrassed because you didn't follow their recommendations and now you're having symptoms?<br><br>Or maybe you used drugs or alcohol and don't want to tell them?<br><br>Do you think you can manage these symptoms on your own?<br><br>Are you not wanting to bother them because it's a weekend or for some other reason?	Overcoming Barriers<br><br>If you are reluctant to contact your psychiatrist, make a list of reasons.<br><br>Write out an alternative rational response for each reason you wrote down. For example, if you're embarrassed you might write "Nobody is perfect. I own and accept that I didn't take my medications for a week. I will proceed ahead and take care of my health."<br><br>Also consider running your concerns by your supports. See what they have to say.	Check out My Charts in the Wellness Plan. Do you see any patterns?<br><br>Get well!

Daily Review Feedback Category 9: Worsening Symptoms, Down – Psychiatrist (Choice 1.0)

Reflection (P8)	Awareness (S2)	Action (S2)	Thrive (U5)	(U5)	My Skills (P5)
**1**<br>**RANDOM UNLINKED**	**2**<br>**STATIC**	**3**<br>**STATIC**	**4**<br>**RANDOM LINKED**	**5**<br>**RANDOM LINKED**	**6**<br>**RANDOM UNLINKED**
You're rating yourself as Moderately Down. It appears that you may be experiencing symptoms typical of depression.<br><br>Take a few minutes and check in with yourself.<br><br>Continue for ideas…	AWARENESS<br><br>Check it out. Are you having any of the following symptoms?<br><br>• Low mood<br>• Reduced interest and pleasure<br>• Weight loss or weight gain<br>• Sleeping too much or too little<br>• Physical agitation or slowing down<br>• Fatigue or loss of energy<br>• Feeling worthless or guilty<br>• Difficulty concentrating<br>• Thoughts of death or suicide<br><br>If you are experiencing several of these symptoms, you are likely experiencing depression	ACTION<br><br>✓✓ Call your psychiatrist if they do not already know about any changes in how you are doing.<br><br>✓✓ Let your supports know that you seem to be experiencing depression.<br><br>✓✓ Use dial up skills. Start moving, warm up, get more active, stay involved.<br><br>✓✓ Keep taking your medications and make sure you are getting proper sleep.	Clinician's Corner…<br><br>I have worked with many individuals living with and living beyond bipolar disorder over the years.<br><br>The challenges faced are huge. I see the problems. I see the havoc. I also see the strength and determination involved in overcoming symptoms and getting well.<br><br>Be honest with yourself, accept your strengths and weaknesses, treat your illness, and live a life with meaning. You can thrive!<br><br>------Dr. Cynthia Dopke	(Skip)	Try out one of My Skills in My Resources in the Wellness Plan.<br><br>Get well!

Daily Review Feedback Category 9: Worsening Symptoms, Down – Supports (Choice 2.0)

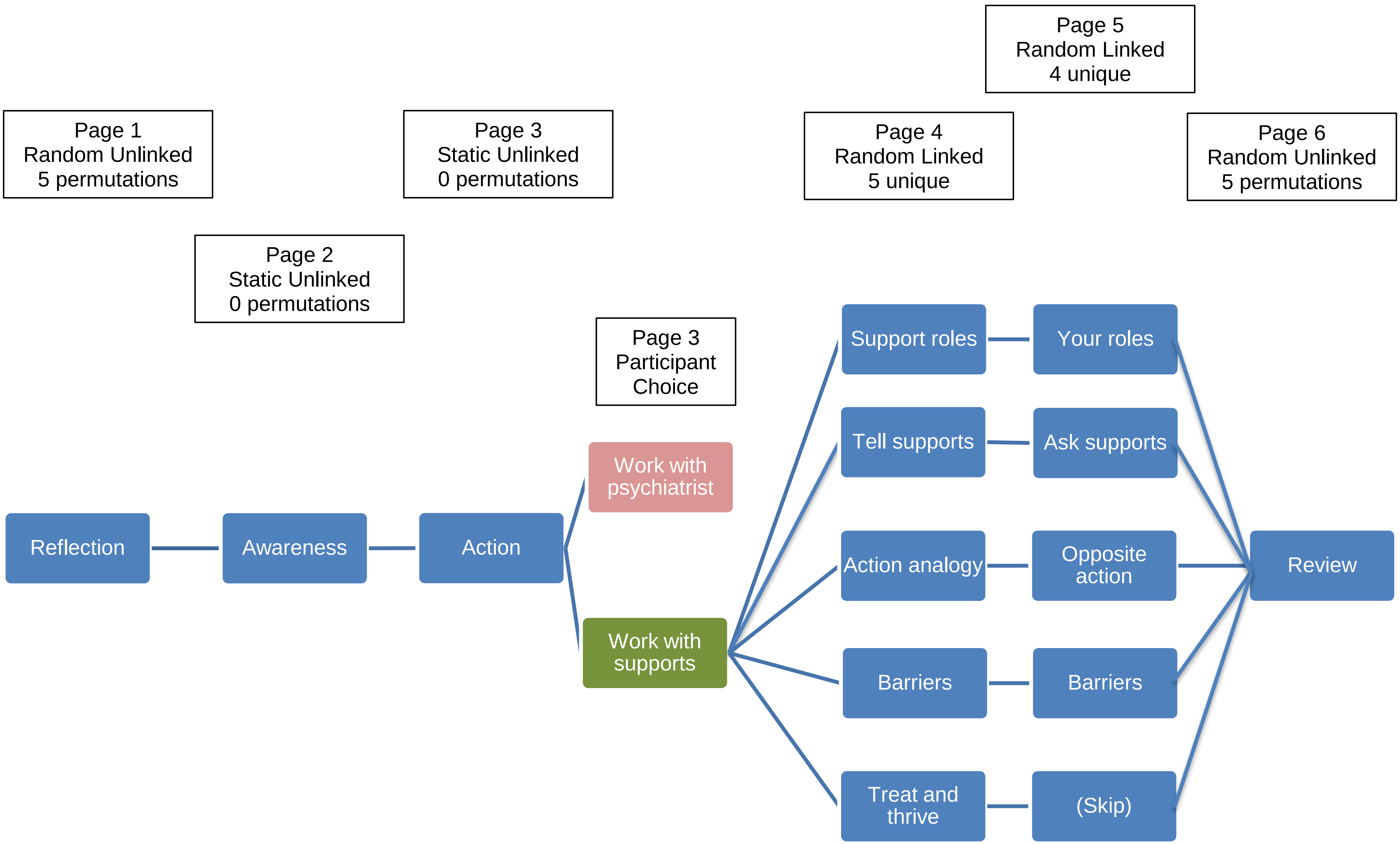

Daily Review Feedback Category 9: Worsening Symptoms, Down – Supports (Choice 2.0)

Reflection (P6)	Awareness (S2)	Action (S2)	Roles (U6)	Roles (U6)	A & A (P7)
**1**<br>**RANDOM UNLINKED**	**2**<br>**STATIC**	**3**<br>**STATIC**	**4**<br>**RANDOM LINKED**	**5**<br>**RANDOM LINKED**	**6**<br>**RANDOM UNLINKED**
You're saying that you are Moderately Down. Seems like a change from how you've been doing overall.<br><br>Now is the time for awareness and action<br><br>Continue for ideas...	AWARENESS<br><br>Check it out. Are you having any of the following symptoms?<br><br>• Low mood<br>• Reduced interest and pleasure<br>• Weight loss or weight gain<br>• Sleeping too much or too little<br>• Physical agitation or slowing down<br>• Fatigue or loss of energy<br>• Feeling worthless or guilty<br>• Difficulty concentrating<br>• Thoughts of death or suicide<br><br>If you are experiencing several of these symptoms, you are likely experiencing depression	ACTION<br><br>✓✓ Call your psychiatrist if they do not already know about any changes in how you are doing.<br><br>✓✓ Let your supports know that you seem to be experiencing depression.<br><br>✓✓ Use dial up skills. Start moving, warm up, get more active, stay involved.<br><br>✓✓ Keep taking your medications and make sure you are getting proper sleep.	WORKING WITH SUPPORTS<br><br>Your support's role...<br><br>Before starting a conversation with your supports, help them to know the role that you would like them to play. You could say:<br>"I'd like you to simply be here for me when I have symptoms. You are not responsible for me. Encourage me to follow my Wellness Plan. Here's some: Dos:<br>• Listen and encourage me<br>• Give honest but gentle feedback<br>Don'ts:<br>• Panic<br>• Get critical<br>• Tell me what to do<br>• Try to solve my problems"	WORKING WITH SUPPORTS<br><br>Your role...<br><br>• Reach out. Let your supports know that you are having symptoms.<br><br>• Let them know what you are going to do to manage your symptoms (e.g., calling your psychiatrist, using skills, taking medications).<br><br>• Agree on a time when you will check in again.<br><br>• Ask them to give you gentle feedback if they think your condition is getting worse.	Check out your plan for Moderate Down under Awareness & Action in the Wellness Plan.<br><br>Get well!

Daily Review Feedback Category 9: Worsening Symptoms, Down – Supports (Choice 2.0)

Reflection (P2)	Awareness (S2)	Action (S2)	Tell (U7)	Ask (U7)	A & A (P8)
**1**<br>**RANDOM UNLINKED**	**2**<br>**STATIC**	**3**<br>**STATIC**	**4**<br>**RANDOM LINKED**	**5**<br>**RANDOM LINKED**	**6**<br>**RANDOM UNLINKED**
Looks like there has been a change in your wellness.<br><br>Now is the time to take a closer look at things.<br><br>Continue for ideas…	AWARENESS<br><br>Check it out. Are you having any of the following symptoms?<br><br>• Low mood<br>• Reduced interest and pleasure<br>• Weight loss or weight gain<br>• Sleeping too much or too little<br>• Physical agitation or slowing down<br>• Fatigue or loss of energy<br>• Feeling worthless or guilty<br>• Difficulty concentrating<br>• Thoughts of death or suicide<br><br>If you are experiencing several [illegible]	ACTION<br><br>✓✓ Call your psychiatrist if they do not already know about any changes in how you are doing.<br><br>✓✓ Let your supports know that you seem to be experiencing depression.<br><br>✓✓ Use dial up skills. Start moving, warm up, get more active, stay involved.<br><br>✓✓ Keep taking your medications and make sure you are getting proper sleep.	WORKING WITH SUPPORTS<br><br>Things to share with your supports:<br><br>• Your symptoms<br>• Actions you have taken to manage your symptoms<br>• What your psychiatrist advised	WORKING WITH SUPPORTS<br><br>Things to ask of your supports:<br><br>• How severe do your symptoms appear to be<br>• What do they think you should do (what has worked in the past)<br>• When you should follow--- up with them	Check out your anchors for Moderate Down under Awareness & Action in the Wellness Plan.<br><br>Get well!

Daily Review Feedback Category 9: Worsening Symptoms, Down – Supports (Choice 2.0)

Reflection (P7)	Awareness (S2)	Action (S2)	Analogy (U3)	Opposite (U6)	A & A (P10)
**1**<br>**RANDOM UNLINKED**	**2**<br>**STATIC**	**3**<br>**STATIC**	**4**<br>**RANDOM LINKED**	**5**<br>**RANDOM LINKED**	**6**<br>**RANDOM UNLINKED**
Seems like your mood has dropped and that you may be experiencing symptoms of depression.<br><br>Check in with yourself. See what you might need to do.<br><br>Continue for ideas…	AWARENESS<br><br>Check it out. Are you having any of the following symptoms?<br><br>• Low mood<br>• Reduced interest and pleasure<br>• Weight loss or weight gain<br>• Sleeping too much or too little<br>• Physical agitation or slowing down<br>• Fatigue or loss of energy<br>• Feeling worthless or guilty<br>• Difficulty concentrating<br>• Thoughts of death or suicide<br><br>If you are experiencing several of these symptoms, you are likely experiencing depression	ACTION<br><br>✓✓ Call your psychiatrist if they do not already know about any changes in how you are doing.<br><br>✓✓ Let your supports know that you seem to be experiencing depression.<br><br>✓✓ Use dial up skills. Start moving, warm up, get more active, stay involved.<br><br>✓✓ Keep taking your medications and make sure you are getting proper sleep.	Keep your eye on the prize!<br><br>Remember that bipolar disorder is recurrent. That means symptoms are likely to occur from time to time.<br><br>While that is a problem, the most important thing is what you do when you have a recurrence!<br><br>Take swift action. Get rid of the symptoms. Minimize the impact of symptoms on your life.<br><br>It's like getting the flu. Rest and good nutrition can help a speedy recovery.<br><br>Take your medications, work with your team, and dial down.	Easier said than done?<br><br>Right! It is really hard to act in opposition to your mood state.<br><br>There are two factors involved:<br><br>1. Motivation<br>Lack of motivation is part of being depressed. So the very thing you need to take care of yourself is missing! So you have to find a way to act despite not wanting to!<br><br>2. Action opposite mood Your state of mind influences your behavior. That is, feeling down reduces activity. To speed up you must deliberately override this process. It's tricky.	Check out what you said you would do when Moderate Down in the Wellness Plan. Check out -3 under Awareness & Action.<br><br>Get well!

Daily Review Feedback Category 9: Worsening Symptoms, Down – Supports (Choice 2.0)

Reflection (P4)	Awareness (S2)	Action (S2)	Barriers (U8)	Barriers (U8)	My Charts (P4)
**1**<br>**RANDOM UNLINKED**	**2**<br>**STATIC**	**3**<br>**STATIC**	**4**<br>**RANDOM LINKED**	**5**<br>**RANDOM LINKED**	**6**<br>**RANDOM UNLINKED**
You're reporting a shift in your symptom status.<br><br>Take a minute to reflect and problem solve<br><br>Continue for ideas…	AWARENESS<br><br>Check it out. Are you having any of the following symptoms?<br><br>• Low mood<br>• Reduced interest and pleasure<br>• Weight loss or weight gain<br>• Sleeping too much or too little<br>• Physical agitation or slowing down<br>• Fatigue or loss of energy<br>• Feeling worthless or guilty<br>• Difficulty concentrating<br>• Thoughts of death or suicide<br><br>If you are experiencing several of these symptoms, you are	ACTION<br><br>✓✓ Call your psychiatrist if they do not already know about any changes in how you are doing.<br><br>✓✓ Let your supports know that you seem to be experiencing depression.<br><br>✓✓ Use dial up skills. Start moving, warm up, get more active, stay involved.<br><br>✓✓ Keep taking your medications and make sure you are getting proper sleep.	Overcoming Barriers<br><br>Is something getting in the way of reaching out to your supports?<br><br>Are you afraid they will tell you something you don't want to hear?<br><br>Are you afraid they will worry or get overbearing somehow?<br><br>Do you think you can manage these symptoms on your own?<br><br>Are you not wanting to bother them for some reason?	Overcoming Barriers<br><br>If you are reluctant to contact your supports, make a list of reasons.<br><br>Write out an alternative rational response for each reason you wrote down. For example, if you're concerned they will get overbearing, you might write "I can ask them to call me no more than once per day."<br><br>Also consider running your concerns by your psychiatrist. See what they have to say.	Check out My Charts in the Wellness Plan. Do you see any patterns?<br><br>Get well!

Daily Review Feedback Category 9: Worsening Symptoms, Down – Supports (Choice 2.0)

Reflection (P8)	Awareness (S2)	Action (S2)	Thrive (U5)	(U5)	My Skills (P5)
**1**<br>**RANDOM UNLINKED**	**2**<br>**STATIC**	**3**<br>**STATIC**	**4**<br>**RANDOM LINKED**	**5**<br>**RANDOM LINKED**	**6**<br>**RANDOM UNLINKED**
You're rating yourself as Moderately Down. It appears that you may be experiencing symptoms typical of depression.<br><br>Take a few minutes and check in with yourself.<br><br>Continue for ideas...	AWARENESS<br><br>Check it out. Are you having any of the following symptoms?<br><br>☐ Overly happy/excited/irritable<br>☐ Increased esteem/feeling superior<br>☐ Decreased need for sleep<br>☐ More talkative than usual<br>☐ Racing thoughts<br>☐ Difficulties concentrating<br>☐ Increased activity level<br>☐ Risky activities<br><br>If you are experiencing several of these symptoms daily, you are likely experiencing mania.	ACTION<br><br>✓✓ Call your psychiatrist if they do not already know about any changes in how you are doing.<br><br>✓✓ Let your supports know that you seem to be experiencing depression.<br><br>✓✓ Use dial up skills. Start moving, warm up, get more active, stay involved.<br><br>✓✓ Keep taking your medications and make sure you are getting proper sleep.	Clinician's Corner...<br><br>I have worked with many individuals living with and living beyond bipolar disorder over the years.<br><br>The challenges faced are huge. I see the problems. I see the havoc. I also see the strength and determination involved in overcoming symptoms and getting well.<br><br>Be honest with yourself, accept your strengths and weaknesses, treat your illness, and live a life with meaning. You can thrive!<br><br>------Dr. Cynthia Dopke	(Skip)	Try out one of My Skills in My Resources in the Wellness Plan.<br><br>Get well!

Daily Review Feedback Category 10: Recovering Continuing, Up

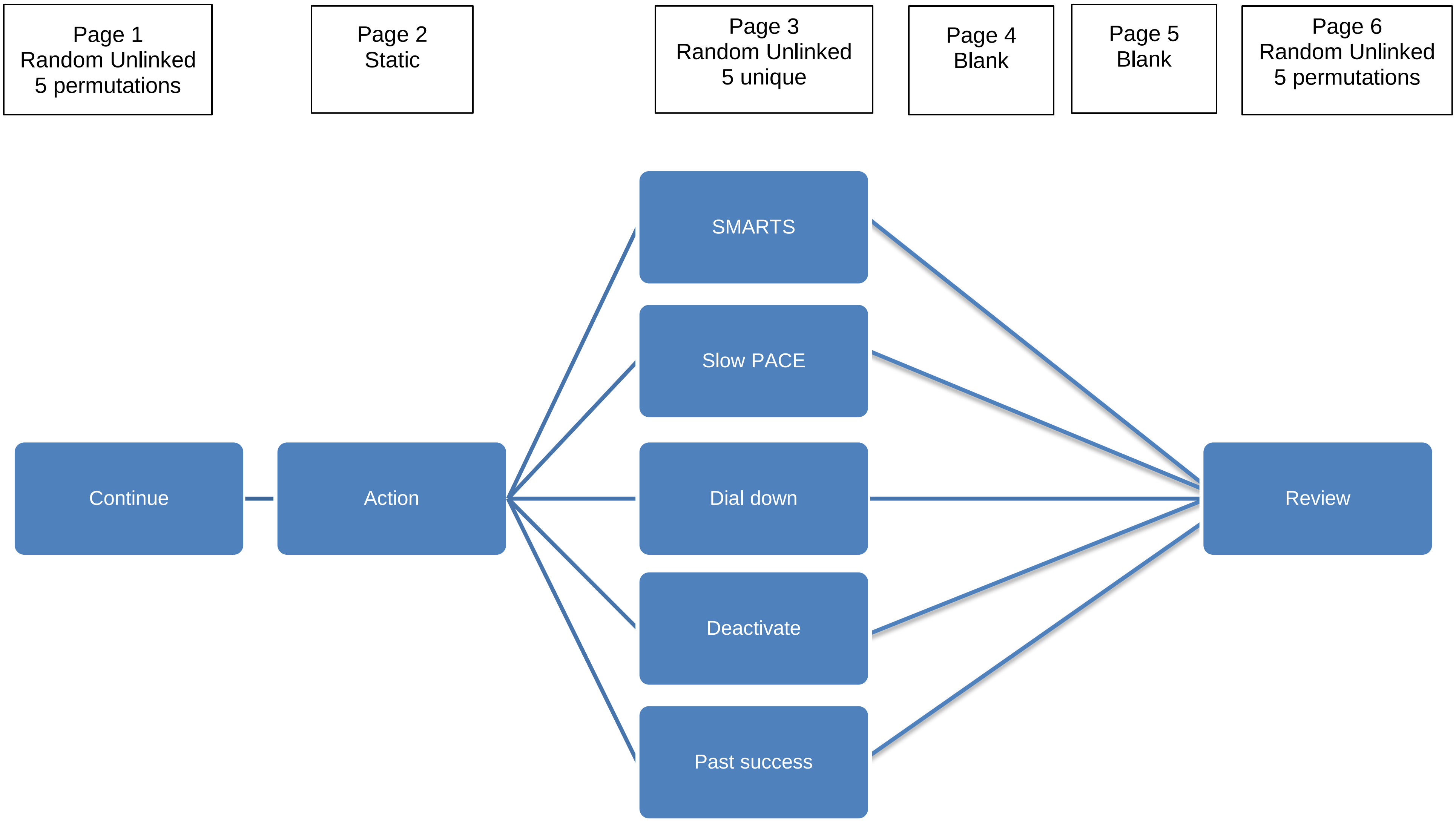

Daily Review Feedback Category 10: Recovering Continuing, Up

Continue (P1)	Action (S1)	SMARTS (U1)			A & A (P1)
**1**<br>**RANDOM UNLINKED**	**2**<br>**RANDOM UNLINKED**	**3**<br>**RANDOM UNLINKED**	**4**<br>**BLANK**	**5**<br>**BLANK**	**6**<br>**RANDOM UNLINKED**
Looks like you're feeling mildly elevated today, but that overall you are headed in the right direction.<br><br>Keep doing whatever you are doing. It seems to be working.<br><br>Continue for additional ideas…	Remember that you are at risk for a mood episode until you have a period of time without symptoms. So keep up your efforts in order to stay well!<br><br>Also consider…<br><br>✓✓ Call your psychiatrist if they do not already know about any changes in how you are doing.<br><br>✓✓ Use dial down skills. Slow down, cool down, get less active, calm your body.<br><br>✓✓ Keep taking your medications and make sure you are getting enough sleep.	Continue in your recovery!<br>Be SMARTS:<br><br>• **S**leep well<br>• Take **m**edications<br>• **A**ttend to diet, exercise, and substance use<br>• Keep a good **R**outine<br>• Stay **T**ranquil<br>• **S**ocialize in moderation<br><br>Just pick a couple of simple things each day to focus on. Every effort counts!			In the Wellness Plan, review your ideas for managing when mildly up. Look at your Plan under Awareness & Action.<br><br>Get well!

Daily Review Feedback Category 10: Recovering Continuing, Up

Continue (P2)	Action (S1)	Slow PACE (U2)			A & A (P2)
**1 RANDOM UNLINKED**	**2 RANDOM UNLINKED**	**3 RANDOM UNLINKED**	**4 BLANK**	**5 BLANK**	**6 RANDOM UNLINKED**
It seems that you're mildly up today, but that overall things are headed in a good direction.<br><br>Has anything in particular helped with your recovery? If so, keep it going!<br><br>Continue for additional ideas…	Remember that you are at risk for a mood episode until you have a period of time without symptoms. So keep up your efforts in order to stay well!<br><br>Also consider…<br><br>✓✓ Call your psychiatrist if they do not already know about any changes in how you are doing.<br><br>✓✓ Use dial down skills. Slow down, cool down, get less active, calm your body.<br><br>✓✓ Keep taking your medications and make sure you are getting enough sleep.	Slowing your body repairs mild ups in mood. PACE yourself. Slow down!<br><br>Not everything is urgent. Set goals so that you don't overdo it:<br><br>• **P**riority: Stick to high priority tasks. Everything cannot be important.<br>• **A**ttitude: Stay mindful and grounded. Not all ideas are great in the long run.<br>• **C**ommonsensical: Make sure the tasks fit into your life plan.<br>• **E**ven: Keep a balance of activity and rest. Go slower than you are inclined.			Check out your anchors for Mild Up in Awareness & Action in the Wellness Plan.<br><br>Get well!

Daily Review Feedback Category 10: Recovering Continuing, Up

Continue (P3)	Action (S1)	Dial down (U3)			My Charts (P3)
**1**<br>**RANDOM UNLINKED**	**2**<br>**RANDOM UNLINKED**	**3**<br>**RANDOM UNLINKED**	**4**<br>**BLANK**	**5**<br>**BLANK**	**6**<br>**RANDOM UNLINKED**
You're checking in as somewhat up today, but overall it appears that you are shifting towards well.<br><br>Continue using strategies and skills that have helped to promote recovery.<br><br>Continue for additional ideas…	Remember that you are at risk for a mood episode until you have a period of time without symptoms. So keep up your efforts in order to stay well!<br><br>Also consider…<br><br>✓✓ Call your psychiatrist if they do not already know about any changes in how you are doing.<br><br>✓✓ Use dial down skills. Slow down, cool down, get less active, calm your body.<br><br>✓✓ Keep taking your medications and make sure you are getting enough sleep.	Continue in your recovery!<br>Use Dial Down skills.<br><br>Pace yourself. Do less than usual. Set aside projects that seem really important but might not be so urgent after all. Quiet your movements.<br><br>Deactivate yourself. Move slower than usual. Talk slower than usual. Think slower than usual. Breathe slower than usual. Quiet your body.<br><br>Decrease involvements with others. See others less than desired, talk less than desired, offer things to others less than desired. Quiet your social appetite.			Check out My Charts in the Wellness Plan. Do you see any patterns?<br><br>Get well!

Daily Review Feedback Category 10: Recovering Continuing, Up

Continue (P4)	Action (S1)	Deactivate (U4)			My Skills (P4)
**1 RANDOM UNLINKED**	**2 RANDOM UNLINKED**	**3 RANDOM UNLINKED**	**4 BLANK**	**5 BLANK**	**6 RANDOM UNLINKED**
It seems as though you are mildly up today, but that you are starting to recover.<br><br>Keep up the good work. Take it one step at a time.<br><br>Continue to read more…	Remember that you are at risk for a mood episode until you have a period of time without symptoms. So keep up your efforts in order to stay well!<br><br>Also consider…<br><br>✓✓ Call your psychiatrist if they do not already know about any changes in how you are doing.<br><br>✓✓ Use dial down skills. Slow down, cool down, get less active, calm your body.<br><br>✓✓ Keep taking your medications and make sure you are getting enough sleep.	Try as best you can to get to a place of peace and stillness.<br><br>Excitement is good in life, from time to time. But mania is destructive. Notice early warning signs of mania and dial down.<br><br>Do some yoga, meditate, breathe deeply. Let your body rest.			Check out My Skills under My Resources in the Wellness Plan. Dial Down for wellness.<br><br>Get well!

Daily Review Feedback Category 10: Recovering Continuing, Up

Continue (P5)	Action (S1)	Past success (U5)			Reduce Risk (P5)
**1**<br>**RANDOM UNLINKED**	**2**<br>**RANDOM UNLINKED**	**3**<br>**RANDOM UNLINKED**	**4**<br>**BLANK**	**5**<br>**BLANK**	**6**<br>**RANDOM UNLINKED**
You're saying that you're mildly up today, but overall it seems that things are improving.<br><br>Take a few moments to acknowledge the hard work you have put into your recovery. Keep doing whatever you've been doing. It seems to be working!<br><br>Continue for additional ideas…	Remember that you are at risk for a mood episode until you have a period of time without symptoms. So keep up your efforts in order to stay well!<br><br>Also consider…<br><br>✓✓ Call your psychiatrist if they do not already know about any changes in how you are doing.<br><br>✓✓ Use dial down skills. Slow down, cool down, get less active, calm your body.<br><br>✓✓ Keep taking your medications and make sure you are getting enough sleep.	What have you done in the past to dial down when you are up? What has worked for you?<br><br>Try it again.<br><br>Persevere… Get out of<br><br>the woods! Get<br><br>balanced!			Take some time to review your Wellness Plan. Focus on Lifestyle Skills!<br><br>Get well!

Daily Review Feedback Category 11: Recovering Continuing, Down

Page 1 Random Unlinked 5 permutations	Page 2 Static	Page 3 Random Unlinked 5 unique	Page 4 Blank	Page 5 Blank	Page 6 Random Unlinked 5 permutations

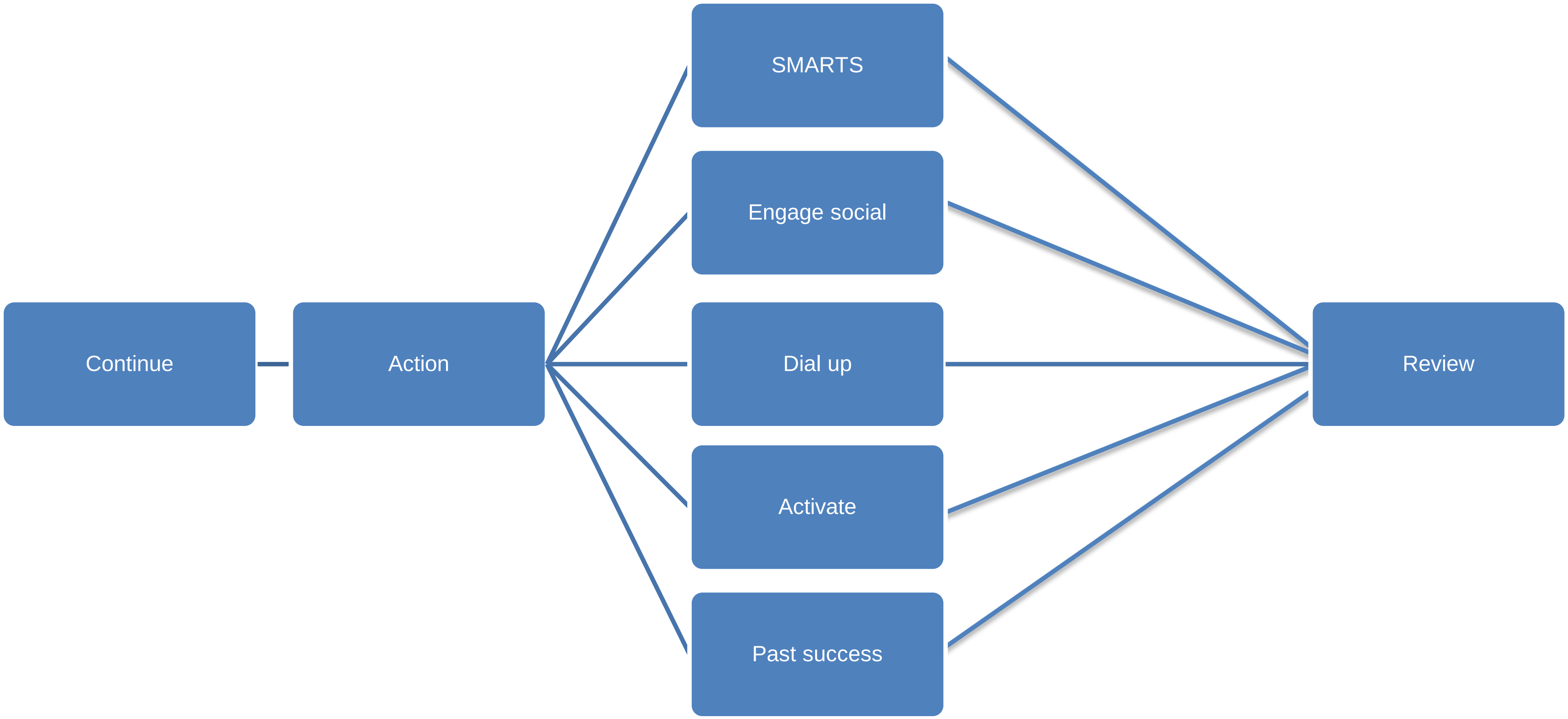

Daily Review Feedback Category 11: Recovering Continuing, Down

Continue (P6)	Action (S2)	SMARTS (U1)			A & A (P6)
**1**<br>**RANDOM UNLINKED**	**2**<br>**RANDOM UNLINKED**	**3**<br>**RANDOM UNLINKED**	**4**<br>**BLANK**	**5**<br>**BLANK**	**6**<br>**RANDOM UNLINKED**
Looks like you're feeling mildly down today, but that overall you are headed in the right direction.<br><br>Keep doing whatever you are doing. It seems to be working.<br><br>Continue for additional ideas…	Remember that you are at risk for a mood episode until you have a period of time without symptoms. So keep up your efforts in order to stay well!<br><br>Also consider…<br><br>✓✓ Call your psychiatrist if they do not already know about any changes in how you are doing.<br><br>✓✓ Use dial up skills. Start moving, warm up, get more active, stay involved.<br><br>✓✓ Keep taking your medications and make sure you are getting enough sleep.	Continue in your recovery!<br>Be SMARTS:<br><br>• **S**leep well<br>• Take **m**edications<br>• **A**ttend to diet, exercise, and substance use<br>• Keep a good **R**outine<br>• Stay **T**ranquil<br>• **S**ocialize in moderation<br><br>Just pick a couple of simple things each day to focus on. Every effort counts!			In the Wellness Plan, review your ideas for managing when mildly down. Look at your Plan under Awareness & Action.<br><br>Get well!

Daily Review Feedback Category 11: Recovering Continuing, Down

Continue (P7) | Action (S2) | Engage social (U6) | A & A (P7)

1<br>RANDOM UNLINKED	2<br>RANDOM UNLINKED	3<br>RANDOM UNLINKED	4<br>BLANK	5<br>BLANK	6<br>RANDOM UNLINKED
It seems that you're mildly down today, but that overall things are headed in a good direction.<br><br>Has anything in particular helped with your recovery? If so, keep it up.<br><br>Continue for additional ideas…	Remember that you are at risk for a mood episode until you have a period of time without symptoms. So keep up your efforts in order to stay well!<br><br>Also consider…<br><br>✓✓ Call your psychiatrist if they do not already know about any changes in how you are doing.<br><br>✓✓ Use dial up skills. Start moving, warm up, get more active, stay involved.<br><br>✓✓ Keep taking your medications and make sure you are getting enough sleep.	Increase Offers<br><br>Giving to others repairs mild drops in mood. Start easy and slow. But go!<br><br>• Offer help to someone<br>• Offer support to someone<br>• Respond to voicemails and emails<br><br>Make a list of ways to give to and receive from others that are simple. Not too challenging.<br><br>START SMALL! BE <u>PRESENT</u>!			Check out your anchors for Mild Down under Awareness & Action in the Wellness Plan.<br><br>Get well!

Daily Review Feedback Category 11: Recovering Continuing, Down

Continue (P8)	Action (S2)	Dial up (U7)			My Charts (P3)
**1<br>RANDOM UNLINKED**	**2<br>RANDOM UNLINKED**	**3<br>RANDOM UNLINKED**	**4<br>BLANK**	**5<br>BLANK**	**6<br>RANDOM UNLINKED**
You're checking in as somewhat down today, but overall it appears that you are shifting towards well.<br><br>Continue using strategies and skills that have helped to promote recovery.<br><br>Continue for additional ideas…	Remember that you are at risk for a mood episode until you have a period of time without symptoms. So keep up your efforts in order to stay well!<br><br>Also consider…<br><br>✓✓ Call your psychiatrist if they do not already know about any changes in how you are doing.<br><br>✓✓ Use dial up skills. Start moving, warm up, get more active, stay involved.<br><br>✓✓ Keep taking your medications and make sure you are getting enough sleep.	Continue in your recovery!<br>Use Dial Up skills.<br><br>Steady participation is the key. Accomplish something simple. Do some small to bring you pleasure. Keep moving, just a bit.<br><br>Activate yourself. Move around. Talk to someone. Look at something stimulating, describe it. Smell, touch, taste, listen…<br><br>Increase involvements with others. Reach out, talk, visit, give. Stimulate your social appetite.			Check out My Charts in the Wellness Plan. Do you see any patterns?<br><br>Get well!

Daily Review Feedback Category 11: Recovering Continuing, Down

Continue (P9)	Action (S2)	Activate (U8)			My Skills (P4)
**1**<br>**RANDOM UNLINKED**	**2**<br>**RANDOM UNLINKED**	**3**<br>**RANDOM UNLINKED**	**4**<br>**BLANK**	**5**<br>**BLANK**	**6**<br>**RANDOM UNLINKED**
It seems as though you are mildly down today, but that you are starting to recover.<br><br>Keep up the good work. Take it one step at a time.<br><br>Continue to read more…	Remember that you are at risk for a mood episode until you have a period of time without symptoms. So keep up your efforts in order to stay well!<br><br>Also consider…<br><br>✓✓ Call your psychiatrist if they do not already know about any changes in how you are doing.<br><br>✓✓ Use dial up skills. Start moving, warm up, get more active, stay involved.<br><br>✓✓ Keep taking your medications and make sure you are getting enough sleep.	Try as best you can to get to a place of peace and contentment.<br><br>Depression is destructive.<br><br>Do some yoga, meditate, breathe deeply. Wake your body.			Check out My Skills in My Resources in the Wellness Plan. Dial Up for wellness.<br><br>Get well!

Daily Review Feedback Category 11: Recovering Continuing, Down

Continue (P10)	Action (S2)	Past success (U9)			Reduce Risk (P5)
**1**<br>**RANDOM UNLINKED**	**2**<br>**RANDOM UNLINKED**	**3**<br>**RANDOM UNLINKED**	**4**<br>**BLANK**	**5**<br>**BLANK**	**6**<br>**RANDOM UNLINKED**
You're saying that your mood is down today, but overall it seems that things are improving.<br><br>Take a few moments to acknowledge the hard work you have put into your recovery. Keep doing whatever you've been doing. It seems to be working!<br><br>Continue for additional ideas…	Remember that you are at risk for a mood episode until you have a period of time without symptoms. So keep up your efforts in order to stay well!<br><br>Also consider…<br><br>✓✓ Call your psychiatrist if they do not already know about any changes in how you are doing.<br><br>✓✓ Use dial up skills. Start moving, warm up, get more active, stay involved.<br><br>✓✓ Keep taking your medications and make sure you are getting enough sleep.	What have you done in the past to dial up when you are down? What has worked for you?<br><br>Try it again.<br><br>Persevere… Get out of<br><br>the woods! Get<br><br>balanced!			Take some time to review your Wellness Plan. Focus on Lifestyle Skills!<br><br>Get well!

Daily Review Feedback Category 12: Prodromal Continuing, Up - Talk to psychiatrist (Choice 1.0)

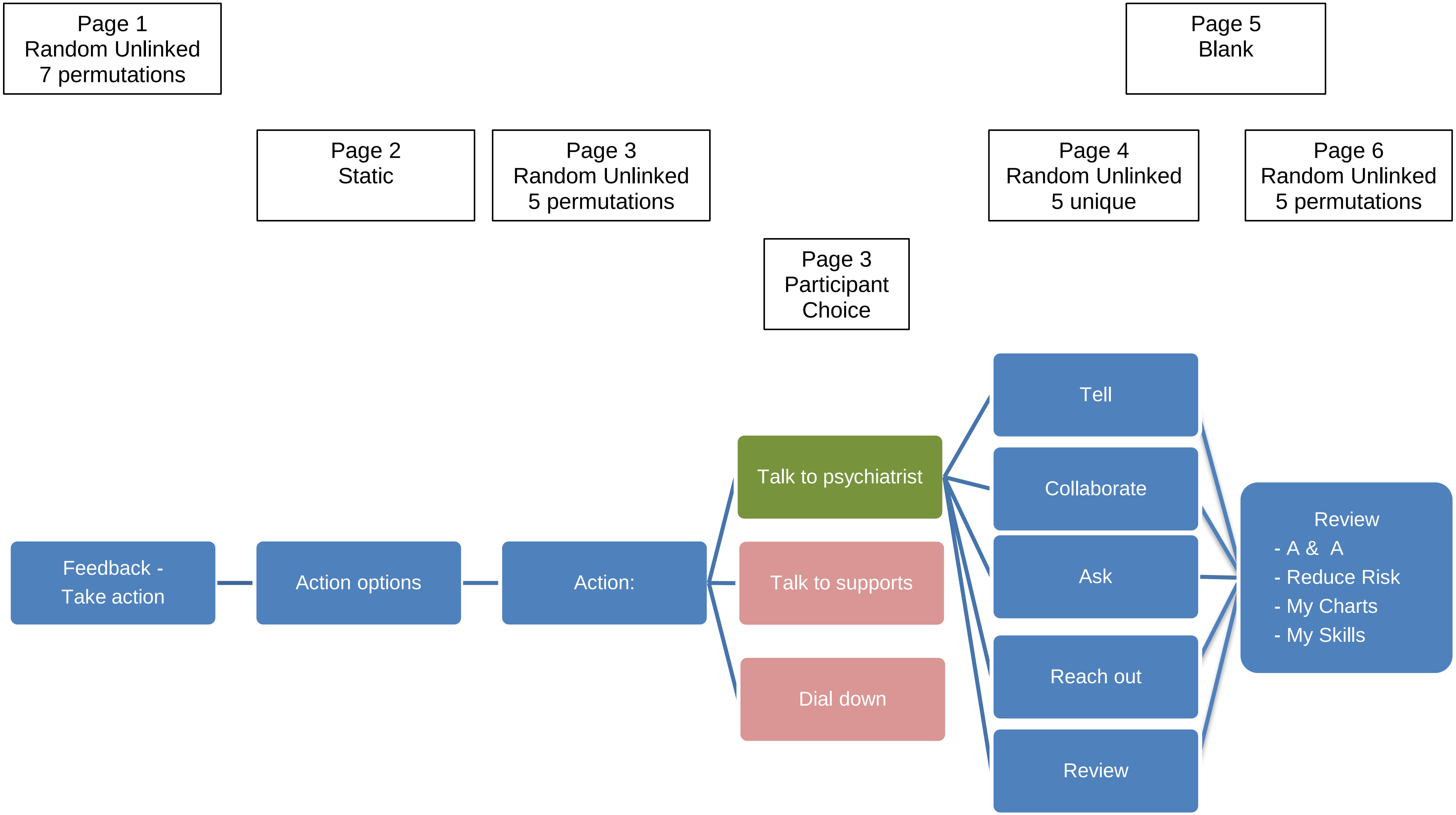

Daily Review Feedback Category 12: Prodromal Continuing, Up – Talk to psychiatrist (Choice 1.0)

Action (P1)	Action (S1)	Action (P1)	Tell (U1)		A & A (P1)
**1**<br>**RANDOM UNLINKED**	**2**<br>**STATIC**	**3**<br>**RANDOM UNLINKED**	**4**<br>**RANDOM UNLINKED**	**5**<br>**BLANK**	**6**<br>**RANDOM UNLINKED**
Looks like you've been having early warning signs of mania.<br><br>Now is the time to take action. Don't let your symptoms get worse.<br><br>Continue for ideas…	Things to do:<br><br>✓✓ Call your psychiatrist if they do not already know about the changes in your mood.<br><br>✓✓ Let your supports know that you are having early warning signs of mania.<br><br>✓✓ Use dial down skills. Slow down, cool down, get less active, calm your body.<br><br>✓✓ Keep taking your medications and make sure you are getting enough sleep.	Knowing what is best to do is easy. Actually doing it is another thing!<br><br>Would you like tips?	Things to share with your psychiatrist:<br><br>✓✓ How you are taking medications<br>✓✓ How you are sleeping<br>✓✓ How you are spending your time<br>✓✓ Any alcohol or drug use<br><br>And:<br><br>✓✓ How you are taking care of yourself<br>✓✓ How relationships are going<br>✓✓ How work (school) is going<br>✓✓ Feedback you are getting from others		Check out your plan for Mild Up under Awareness and Action in the Wellness Plan.<br><br>Get well!

Daily Review Feedback Category 12: Prodromal Continuing, Up – Talk to psychiatrist (Choice 1.0)

Action (P2)	Action (S1)	Action (P2)	Collaborate (U2)		A & A (P2)
**1**<br>**RANDOM UNLINKED**	**2**<br>**STATIC**	**3**<br>**RANDOM UNLINKED**	**4**<br>**RANDOM UNLINKED**	**5**<br>**BLANK**	**6**<br>**RANDOM UNLINKED**
Looks like you've been up for a bit now.<br><br>Take action! Don't let yourself get manic.<br><br>Continue for ideas…	Things to do:<br><br>✓✓ Call your psychiatrist if they do not already know about the changes in your mood.<br><br>✓✓ Let your supports know that you are having early warning signs of mania.<br><br>✓✓ Use dial down skills. Slow down, cool down, get less active, calm your body.<br><br>✓✓ Keep taking your medications and make sure you are getting enough sleep.	Don't fret. Ups and downs are part of bipolar disorder. Everyone has them.<br><br>The key is to be aware and take action.<br><br>Would you like tips?	Remember that your relationship with your psychiatrist is collaborative. That means it is a working relationship.<br><br>You both have something to share. Only you know how you feel and what you want in life. Your psychiatrist knows about what they are seeing when they talk to you. They know about treatments for bipolar disorder.<br><br>Share in your decision making. Be open and honest. Respect your needs. Respect your psychiatrists' expertise. Find a plan that works for you.		Double check your anchors for Mild Up in Awareness and Action. Go to the Wellness Plan to review.<br><br>Get well!

Daily Review Feedback Category 12: Prodromal Continuing, Up – Talk to psychiatrist (Choice 1.0)

Action (P3)	Action (S1)	Action (P3)	Ask (U3)		My Skills (P3)

1<br>**RANDOM UNLINKED**	2<br>**STATIC**	3<br>**RANDOM UNLINKED**	4<br>**RANDOM UNLINKED**	5<br>**BLANK**	6<br>**RANDOM UNLINKED**
You're reporting mild early warning signs of mania.<br><br>Don't let yourself get manic. Take action now!<br><br>Continue for ideas…	Things to do:<br><br>✓✓ Call your psychiatrist if they do not already know about the changes in your mood.<br><br>✓✓ Let your supports know that you are having early warning signs of mania.<br><br>✓✓ Use dial down skills. Slow down, cool down, get less active, calm your body.<br><br>✓✓ Keep taking your medications and make sure you are getting enough sleep.	Symptoms can come because of something you did or did not do. Like missing medications, staying out really late, or using drugs.<br><br>Symptoms can also come from out of the blue.<br><br>Either way, don't judge yourself. Just take care of yourself!<br><br>Would you like tips?	Questions to ask your psychiatrist:<br><br>✓✓ Why do you think I am having symptoms?<br><br>✓✓ What do you think I should do about it?<br><br>✓✓ When should I check back in?		Look at My Skills in the Wellness Plan. Try Dialing Down.<br><br>Get well!

Daily Review Feedback Category 12: Prodromal Continuing, Up – Talk to psychiatrist (Choice 1.0)

Action (P4)	Action (S1)	Action (P4)	Reach out (U4)		My Charts (P4)

1<br>RANDOM UNLINKED	2<br>STATIC	3<br>RANDOM UNLINKED	4<br>RANDOM UNLINKED	5<br>BLANK	6<br>RANDOM UNLINKED
Sounds like you're experiencing early warning signs of mania.<br><br>Take action now! Continue for ideas…	Things to do:<br><br>✓✓ Call your psychiatrist if they do not already know about the changes in your mood.<br><br>✓✓ Let your supports know that you are having early warning signs of mania.<br><br>✓✓ Use dial down skills. Slow down, cool down, get less active, calm your body.<br><br>✓✓ Keep taking your medications and make sure you are getting enough sleep.	Get real practical now. Take care of yourself.<br><br>Would you like tips?	More often than not, individuals do not reach out to their psychiatrists when perhaps they should. That is normal. That is typical. But it is not so helpful!<br><br>What are your thoughts about talking to your psychiatrist?<br><br>Remember that they signed up to help you! They want to talk with you when you are not doing so well. That is what they are there for! So call!		Look at My Charts in the Wellness Plan. How are you doing? What do you need to do for yourself?<br><br>Get well!

Daily Review Feedback Category 12: Prodromal Continuing, Up – Talk to psychiatrist (Choice 1.0)

Action (P5)	Action (S1)	Action (P5)	Review (U5, P1)		Reduce Risk (P5)
**1**<br>**RANDOM UNLINKED**	**2**<br>**STATIC**	**3**<br>**RANDOM UNLINKED**	**4**<br>**RANDOM UNLINKED**	**5**<br>**BLANK**	**6**<br>**RANDOM UNLINKED**
It seems like you've been up for a while now.<br><br>Now is the time to focus on taking care of yourself. Don't let yourself get manic.<br><br>Continue for ideas…	Things to do:<br><br>✓✓ Call your psychiatrist if they do not already know about the changes in your mood.<br><br>✓✓ Let your supports know that you are having early warning signs of mania.<br><br>✓✓ Use dial down skills. Slow down, cool down, get less active, calm your body.<br><br>✓✓ Keep taking your medications and make sure you are getting enough sleep.	Feeling up probably feels good (unless you're irritable). Don't let this state of mind seduce you!<br><br>Get balanced. It is for the best. Would you like tips?	If you're thinking that you don't need or don't want to call your psychiatrist…<br><br>✓✓ Review your action plan for Mild Up<br>✓✓ Ask your supports what they think		Review your commitments under Reduce Risk in the Wellness Plan.<br><br>Get well!

Daily Review Feedback Category 12: Prodromal Continuing, Up – Talk to supports (Choice 2.0)

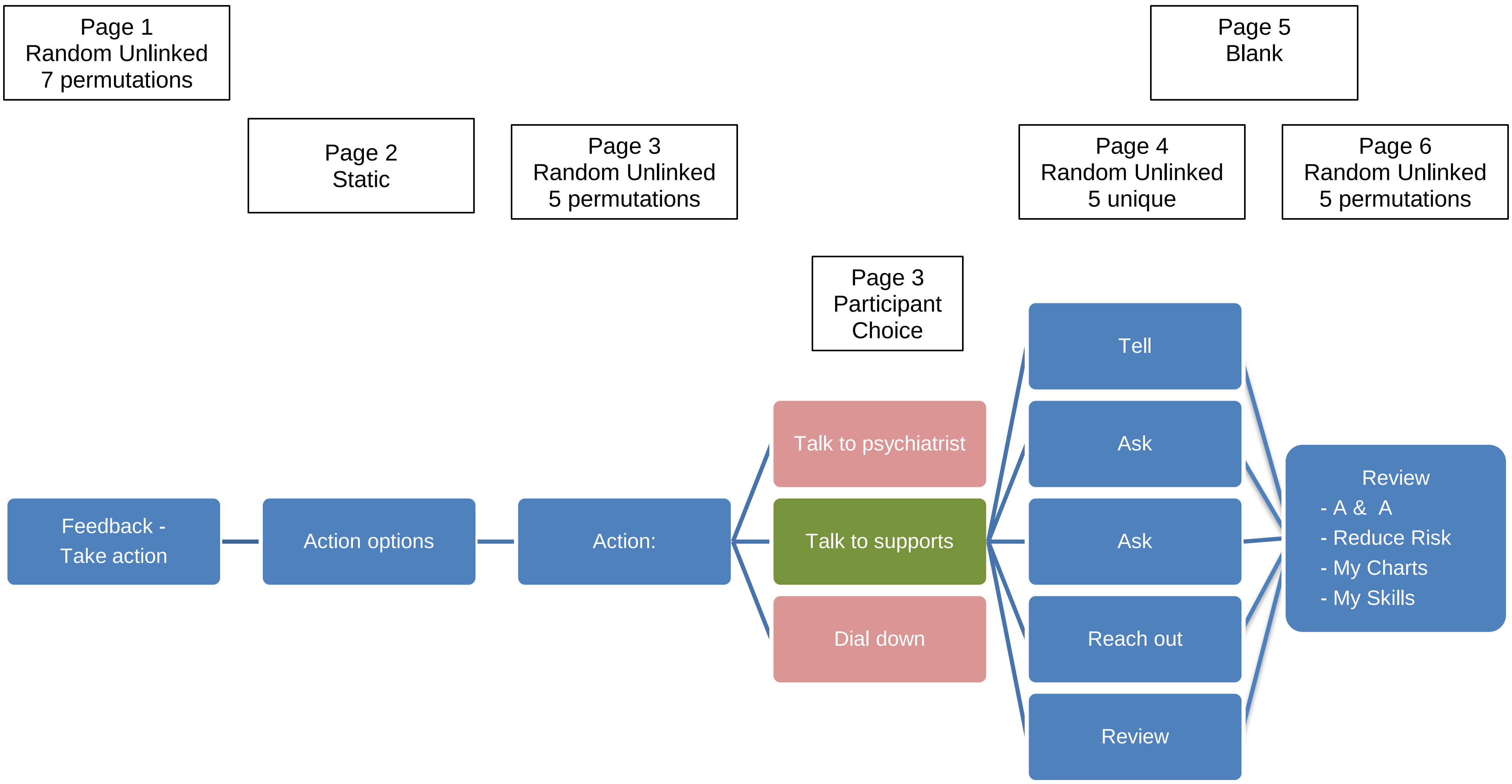

Daily Review Feedback Category 12: Prodromal Continuing, Up – Talk to supports (Choice 2.0)

Action (P1)	Action (S1)	Action (P1)	Tell (U6)		A & A (P1)
**1**<br>**RANDOM UNLINKED**	**2**<br>**STATIC**	**3**<br>**RANDOM UNLINKED**	**4**<br>**RANDOM UNLINKED**	**5**<br>**BLANK**	**6**<br>**RANDOM UNLINKED**
Looks like you've been having early warning signs of mania.<br><br>Now is the time to take action. Don't let your symptoms get worse.<br><br>Continue for ideas...	Things to do:<br><br>✓✓ Call your psychiatrist if they do not already know about the changes in your mood.<br><br>✓✓ Let your supports know that you are having early warning signs of mania.<br><br>✓✓ Use dial down skills. Slow down, cool down, get less active, calm your body.<br><br>✓✓ Keep taking your medications and make sure you are getting enough sleep.	Knowing what is best to do is easy. Actually doing it is another thing!<br><br>Would you like tips?	Things to share with your supports:<br><br>✓ Look at your wellness plan. Review your plan for mild symptoms with your supports.<br><br>✓ Ask that they check in and gently give you feedback if things seem worse in the days ahead.		Check out your plan for Mild Up under Awareness and Action in the Wellness Plan.<br><br>Get well!

Daily Review Feedback Category 12: Prodromal Continuing, Up – Talk to supports (Choice 2.0)

Action (P2)	Action (S1)	Action (P2)	Ask (U7)		A & A (P2)
**1**<br>**RANDOM UNLINKED**	**2**<br>**STATIC**	**3**<br>**RANDOM UNLINKED**	**4**<br>**RANDOM UNLINKED**	**5**<br>**BLANK**	**6**<br>**RANDOM UNLINKED**
Looks like you've been up for a bit now.<br><br>Take action! Don't let yourself get manic.<br><br>Continue for ideas…	Things to do:<br><br>✓✓ Call your psychiatrist if they do not already know about the changes in your mood.<br><br>✓✓ Let your supports know that you are having early warning signs of mania.<br><br>✓✓ Use dial down skills. Slow down, cool down, get less active, calm your body.<br><br>✓✓ Keep taking your medications and make sure you are getting enough sleep.	Don't fret. Ups and downs are part of bipolar disorder. Everyone has them.<br><br>The key is to be aware and take action.<br><br>Would you like tips?	Ask your supports about what has helped you in the past when you've had early warning signs of mania.<br><br>Remember that your supports are there just to be a sounding board. They are not your boss or therapist.<br><br>Listen to what they have to say. Take them seriously.		Double check your anchors for Mild Up in Awareness and Action. Go to the Wellness Plan to review.<br><br>Get well!

Daily Review Feedback Category 12: Prodromal Continuing, Up – Talk to supports (Choice 2.0)

Action (P3)	Action (S1)	Action (P3)	Ask (U8)		My Skills (P3)
**1**<br>**RANDOM UNLINKED**	**2**<br>**STATIC**	**3**<br>**RANDOM UNLINKED**	**4**<br>**RANDOM UNLINKED**	**5**<br>**BLANK**	**6**<br>**RANDOM UNLINKED**
You're reporting mild early warning signs of mania.<br><br>Don't let yourself get manic. Take action now!<br><br>Continue for ideas…	Things to do:<br><br>✓✓ Call your psychiatrist if they do not already know about the changes in your mood.<br><br>✓✓ Let your supports know that you are having early warning signs of mania.<br><br>✓✓ Use dial down skills. Slow down, cool down, get less active, calm your body.<br><br>✓✓ Keep taking your medications and make sure you are getting enough sleep.	Symptoms can come because of something you did or did not do. Like missing medications, staying out really late, or using drugs.<br><br>Symptoms can also come from out of the blue.<br><br>Either way, don't judge yourself. Just take care of yourself!<br><br>Would you like tips?	Questions to ask your supports:<br><br>✓ Why do you think I am having symptoms?<br><br>✓ What do you think I should do about it?<br><br>✓ When should I check back in?		Look at My Skills in the Wellness Plan. Try Dialing Down.<br><br>Get well!

Daily Review Feedback Category 12: Prodromal Continuing, Up – Talk to supports (Choice 2.0)

Action (P4)	Action (S1)	Action (P4)	Reach out (U9)		My Charts (P4)
**1**<br>**RANDOM UNLINKED**	**2**<br>**STATIC**	**3**<br>**RANDOM UNLINKED**	**4**<br>**RANDOM UNLINKED**	**5**<br>**BLANK**	**6**<br>**RANDOM UNLINKED**
Sounds like you're experiencing early warning signs of mania.<br><br>Take action now!<br><br>Continue for ideas…	Things to do:<br><br>✓✓ Call your psychiatrist if they do not already know about the changes in your mood.<br><br>✓✓ Let your supports know that you are having early warning signs of mania.<br><br>✓✓ Use dial down skills. Slow down, cool down, get less active, calm your body.<br><br>✓✓ Keep taking your medications and make sure you are getting enough sleep.	Get real practical now. Take care of yourself.<br><br>Would you like tips?	More often than not, individuals do not reach out to their supports when perhaps they should. That is normal. That is typical. But it is not so helpful!<br><br>What are your thoughts about talking to your supports?<br><br>Remember that they are willing to help you! They want to talk with you when you are not doing so well. That is what they are there for! So call!		Look at My Charts in the Wellness Plan. How are you doing? What do you need to do for yourself?<br><br>Get well!

Daily Review Feedback Category 12: Prodromal Continuing, Up – Talk to supports (Choice 2.0)

Action (P5)	Action (S1)	Action (P5)	Review (U10, P1)		Reduce Risk (P5)
**1**<br>**RANDOM UNLINKED**	**2**<br>**STATIC**	**3**<br>**RANDOM UNLINKED**	**4**<br>**RANDOM UNLINKED**	**5**<br>**BLANK**	**6**<br>**RANDOM UNLINKED**
It seems like you've been up for a while now.<br><br>Now is the time to focus on taking care of yourself. Don't let yourself get manic.<br><br>Continue for ideas…	Things to do:<br><br>✓✓ Call your psychiatrist if they do not already know about the changes in your mood.<br><br>✓✓ Let your supports know that you are having early warning signs of mania.<br><br>✓✓ Use dial down skills. Slow down, cool down, get less active, calm your body.<br><br>✓✓ Keep taking your medications and make sure you are getting enough sleep.	Feeling up probably feels good (unless you're irritable). Don't let this state of mind seduce you!<br><br>Get balanced. It is for the best.<br><br>Would you like tips?	If you're thinking that you don't need or don't want to call your supports…<br><br>✓ Review your action plan for Mild Up<br><br>✓ Ask your psychiatrist what they think		Review your commitments under Reduce Risk in the Wellness Plan.<br><br>Get well!

Daily Review Feedback Category 12: Prodromal Continuing, Up – Dial Down (Choice 3.0)

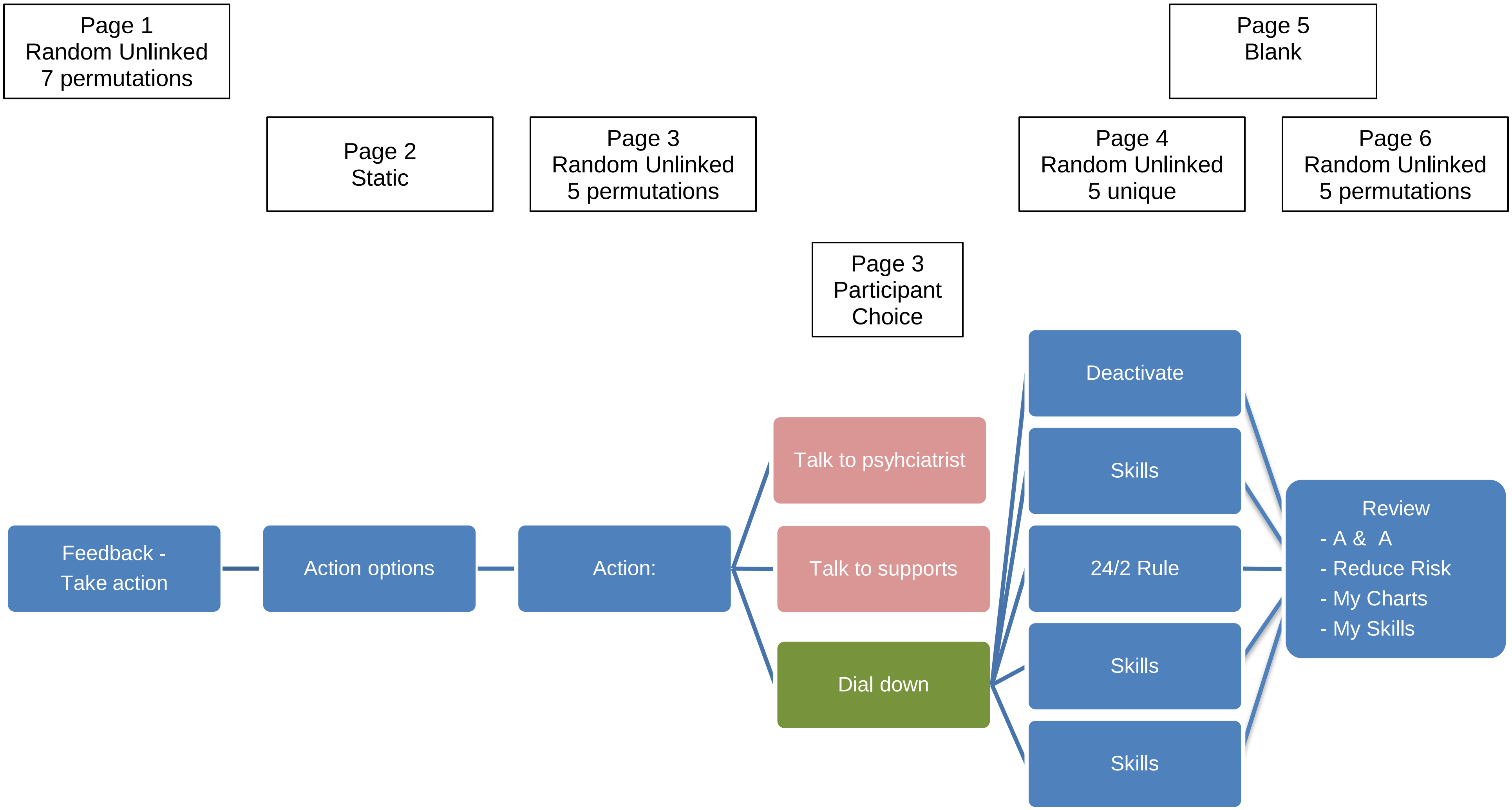

Daily Review Feedback Category 12: Prodromal Continuing, Up – Dial down (Choice 3.0)

Action (P1)	Action (S1)	Action (P1)	Deactivate (U11)		A & A (P1)
**1**<br>**RANDOM UNLINKED**	**2**<br>**STATIC**	**3**<br>**RANDOM UNLINKED**	**4**<br>**RANDOM UNLINKED**	**5**<br>**BLANK**	**6**<br>**RANDOM UNLINKED**
Looks like you've been having early warning signs of mania.<br><br>Now is the time to take action. Don't let your symptoms get worse.<br><br>Continue for ideas…	Things to do:<br><br>✓✓ Call your psychiatrist if they do not already know about the changes in your mood.<br><br>✓✓ Let your supports know that you are having early warning signs of mania.<br><br>✓✓ Use dial down skills. Slow down, cool down, get less active, calm your body.<br><br>✓✓ Keep taking your medications and make sure you are getting enough sleep.	Knowing what is best to do is easy. Actually doing it is another thing!<br><br>Would you like tips?	Things to consider:<br><br>Getting really active can fan the flames of mania. That means when you feel up, getting more active can make things worse.<br><br>Take a few minutes each day to quiet your physiology, your body. Sit still and just be still. Nothing more, nothing less.<br><br>Take some deep breaths. Let go all of you would like to do or think you need to do today. Just breathe.<br><br>Pull up a calming video of yoga or guided imagery or meditation on YouTube. Cool down your system!<br><br>You'll feel and function better in the long run. Try it!		Check out your plan for Mild Up under Awareness and Action in the Wellness Plan.<br><br>Get well!

Daily Review Feedback Category 12: Prodromal Continuing, Up – Dial down (Choice 3.0)

Action (P2)	Action (S1)	Action (P2)	Skills (U12)		A & A (P2)

1<br>RANDOM UNLINKED	2<br>STATIC	3<br>RANDOM UNLINKED	4<br>RANDOM UNLINKED	5<br>BLANK	6<br>RANDOM UNLINKED
Looks like you've been up for a bit now.<br><br>Take action! Don't let yourself get manic.<br><br>Continue for ideas…	Things to do:<br><br>✓✓ Call your psychiatrist if they do not already know about the changes in your mood.<br><br>✓✓ Let your supports know that you are having early warning signs of mania.<br><br>✓✓ Use dial down skills. Slow down, cool down, get less active, calm your body.<br><br>✓✓ Keep taking your medications and make sure you are getting enough sleep.	Don't fret. Ups and downs are part of bipolar disorder. Everyone has them.<br><br>The key is to be aware and take action.<br><br>Would you like tips?	While it may feel good to be so activated, remember that this state of mind puts you at risk for something bad (mania).<br><br>Take some time to cool off, to dial down.<br><br>Look at Skills or My Skills. Take some time each day to adjust your personal tempo.<br><br>Balanced is good!		Double check your anchors for Mild Up in Awareness and Action. Go to the Wellness Plan to review.<br><br>Get well!

Daily Review Feedback Category 12: Prodromal Continuing, Up – Dial down (Choice 3.0)

Action (P3)	Action (S1)	Action (P3)	24/2 Rule (U13)		My Skills (P3)
**1**<br>**RANDOM UNLINKED**	**2**<br>**STATIC**	**3**<br>**RANDOM UNLINKED**	**4**<br>**RANDOM UNLINKED**	**5**<br>**BLANK**	**6**<br>**RANDOM UNLINKED**
You're reporting mild early warning signs of mania.<br><br>Don't let yourself get manic. Take action now!<br><br>Continue for ideas…	Things to do:<br><br>✓✓ Call your psychiatrist if they do not already know about the changes in your mood.<br><br>✓✓ Let your supports know that you are having early warning signs of mania.<br><br>✓✓ Use dial down skills. Slow down, cool down, get less active, calm your body.<br><br>✓✓ Keep taking your medications and make sure you are getting enough sleep.	Symptoms can come because of something you did or did not do. Like missing medications, staying out really late, or using drugs.<br><br>Symptoms can also come from out of the blue.<br><br>Either way, don't judge yourself. Just take care of yourself!<br><br>Would you like tips?	24/2 RULE<br><br>If you're thinking about something you should or ought to do…press the pause button.<br><br>Ideas that come about during manic states could be good. Or they could be bad.<br><br>Take 24 hours to think about whatever it is you want to do. Or ask 2 people what they think.		Look at My Skills in the Wellness Plan. Try Dialing Down.<br><br>Get well!

Daily Review Feedback Category 12: Prodromal Continuing, Up – Dial down (Choice 3.0)

Action (P4)	Action (S1)	Action (P4)	Skills (U14)		My Charts (P4)

1<br>**RANDOM UNLINKED**	2<br>**STATIC**	3<br>**RANDOM UNLINKED**	4<br>**RANDOM UNLINKED**	5<br>**BLANK**	6<br>**RANDOM UNLINKED**
Sounds like you're experiencing early warning signs of mania.<br><br>Take action now!<br><br>Continue for ideas…	Things to do:<br><br>✓✓ Call your psychiatrist if they do not already know about the changes in your mood.<br><br>✓✓ Let your supports know that you are having early warning signs of mania.<br><br>✓✓ Use dial down skills. Slow down, cool down, get less active, calm your body.<br><br>✓✓ Keep taking your medications and make sure you are getting enough sleep.	Get real practical now. Take care of yourself.<br><br>Would you like tips?	DIAL DOWN<br><br>Set aside 10 minutes for yourself today. Pick a Dial Down skill. You can pick from Skills, My Skills, or just from your own experience.<br><br>The key is to get your blood pressure and heart rate to decrease. It is important right now to get your physiology less active.		Look at My Charts in the Wellness Plan. How are you doing? What do you need to do for yourself?<br><br>Get well!

Daily Review Feedback Category 12: Prodromal Continuing, Up – Dial down (Choice 3.0)

Action (P5)	Action (S1)	Action (P5)	Skills (U15)		Reduce Risk (P5)
**1**<br>**RANDOM UNLINKED**	**2**<br>**STATIC**	**3**<br>**RANDOM UNLINKED**	**4**<br>**RANDOM UNLINKED**	**5**<br>**BLANK**	**6**<br>**RANDOM UNLINKED**
It seems like you've been up for a while now.<br><br>Now is the time to focus on taking care of yourself. Don't let yourself get manic.<br><br>Continue for ideas…	Things to do:<br><br>✓✓ Call your psychiatrist if they do not already know about the changes in your mood.<br><br>✓✓ Let your supports know that you are having early warning signs of mania.<br><br>✓✓ Use dial down skills. Slow down, cool down, get less active, calm your body.<br><br>✓✓ Keep taking your medications and make sure you are getting enough sleep.	Feeling up probably feels good (unless you're irritable). Don't let this state of mind seduce you!<br><br>Get balanced. It is for the best. Would you like tips?	DIAL DOWN<br><br>Set aside 10 minutes for yourself today. Take a look at YouTube. Search for:<br><br>• Progressive muscle relaxation<br>• Guided imagery<br>• Mindfulness<br>• Yoga<br><br>Try it out. See if you can't cool down your system. Aim for balance!		Review your commitments under Reduce Risk in the Wellness Plan.<br><br>Get well!

Daily Review Feedback Category 13: Prodromal Continuing, Down - Talk to psychiatrist (Choice 1.0)

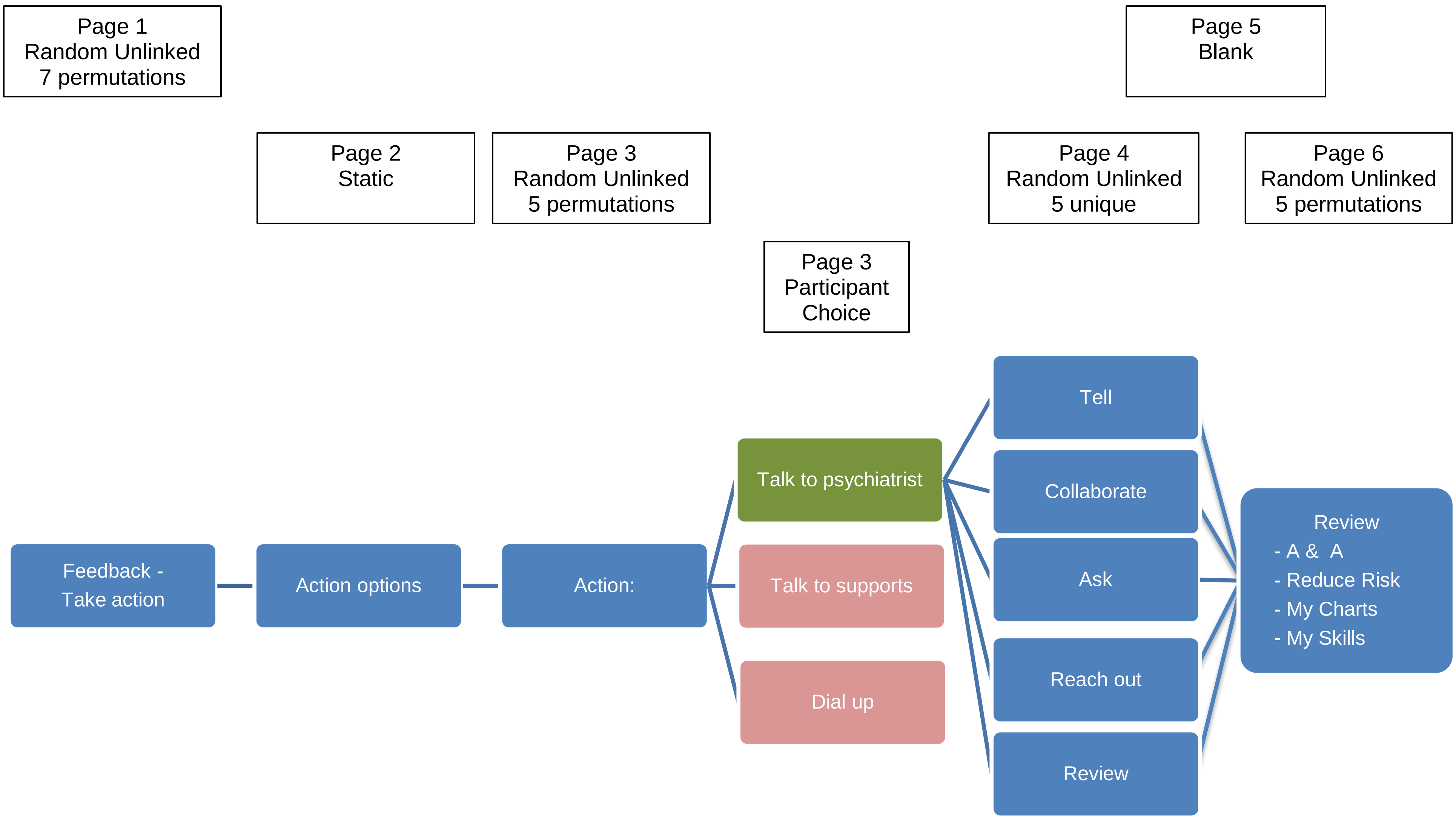

Daily Review Feedback Category 13: Prodromal Continuing, Down – Talk to psychiatrist (Choice 1.0)

Action (P6)	Action (S2)	Action (P1)	Tell (U1)		A & A (P6)
**1**<br>**RANDOM UNLINKED**	**2**<br>**STATIC**	**3**<br>**RANDOM UNLINKED**	**4**<br>**RANDOM UNLINKED**	**5**<br>**BLANK**	**6**<br>**RANDOM UNLINKED**
Looks like you've been having early warning signs of depression.<br><br>Now is the time to take action. Don't let your symptoms get worse.<br><br>Continue for ideas…	Things to do:<br><br>✓✓ Call your psychiatrist if they do not already know about the changes in your mood.<br><br>✓✓ Let your supports know that you are having early warning signs of depression.<br><br>✓✓ Use dial up skills. Start moving, warm up, get more active, stay involved.<br><br>✓✓ Keep taking your medications and make sure you are getting proper sleep.	Knowing what is best to do is easy. Actually doing it is another thing!<br><br>Would you like tips?	Things to share with your psychiatrist:<br><br>✓✓ How you are taking medications<br>✓✓ How you are sleeping<br>✓✓ How you are spending your time<br>✓✓ Any alcohol or drug use<br><br>And:<br><br>✓✓ How you are taking care of yourself<br>✓✓ How relationships are going<br>✓✓ How work (school) is going<br>✓✓ Feedback you are getting from others		Check out your plan for Mild Down in Awareness and Action in the Wellness Plan.<br><br>Get well!

Daily Review Feedback Category 13: Prodromal Continuing, Down – Talk to psychiatrist (Choice 1.0)

Action (P7)	Action (S2)	Action (P2)	Collaborate (U2)		A & A (P7)
**1 RANDOM UNLINKED**	**2 STATIC**	**3 RANDOM UNLINKED**	**4 RANDOM UNLINKED**	**5 BLANK**	**6 RANDOM UNLINKED**
Looks like you've been down for some time now.<br><br>Take action! Don't let yourself get depressed.<br><br>Continue for ideas…	Things to do:<br><br>✓✓ Call your psychiatrist if they do not already know about the changes in your mood.<br><br>✓✓ Let your supports know that you are having early warning signs of depression.<br><br>✓✓ Use dial up skills. Start moving, warm up, get more active, stay involved.<br><br>✓✓ Keep taking your medications and make sure you are getting proper sleep.	Don't fret. Ups and downs are part of bipolar disorder. Everyone has them.<br><br>The key is to be aware and take action.<br><br>Would you like tips?	Remember that your relationship with your psychiatrist is collaborative. That means it is a working relationship.<br><br>You both have something to share. Only you know how you feel and what you want in life. Your psychiatrist knows about what they are seeing when they talk to you. They know about treatments for bipolar disorder.<br><br>Share in your decision making. Be open and honest. Respect your needs. Respect your psychiatrists' expertise. Find a plan that works for you.		Double check your anchors for Mild Down in Awareness and Action in the Wellness Plan.<br><br>Get well!

Daily Review Feedback Category 13: Prodromal Continuing, Down – Talk to psychiatrist (Choice 1.0)

Action (P8)	Action (S2)	Action (P3)	Ask (U3)		My Skills (P8)
**1**<br>**RANDOM UNLINKED**	**2**<br>**STATIC**	**3**<br>**RANDOM UNLINKED**	**4**<br>**RANDOM UNLINKED**	**5**<br>**BLANK**	**6**<br>**RANDOM UNLINKED**
You're reporting early warning signs of depression.<br><br>Don't let yourself get depressed. Take action now!<br><br>Continue for ideas…	Things to do:<br><br>✓✓ Call your psychiatrist if they do not already know about the changes in your mood.<br><br>✓✓ Let your supports know that you are having early warning signs of depression.<br><br>✓✓ Use dial up skills. Start moving, warm up, get more active, stay involved.<br><br>✓✓ Keep taking your medications and make sure you are getting proper sleep.	Symptoms can come because of something you did or did not do. Like missing medications, staying out really late, or using drugs.<br><br>Symptoms can also come from out of the blue.<br><br>Either way, don't judge yourself. Just take care of yourself!<br><br>Would you like tips?	Questions to ask your psychiatrist:<br><br>✓✓ Why do you think I am having symptoms?<br><br>✓✓ What do you think I should do about it?<br><br>✓✓ When should I check back in?		Look at My Skills in the Wellness Plan. Try Dialing Up.<br><br>Get well!

Daily Review Feedback Category 13: Prodromal Continuing, Down – Talk to psychiatrist (Choice 1.0)

Action (P9)	Action (S2)	Action (P4)	Reach out (U4)		My Charts (P4)
**1**<br>**RANDOM UNLINKED**	**2**<br>**STATIC**	**3**<br>**RANDOM UNLINKED**	**4**<br>**RANDOM UNLINKED**	**5**<br>**BLANK**	**6**<br>**RANDOM UNLINKED**
Sounds like you're experiencing early warning signs of depression.<br><br>Take action now!<br><br>Continue for<br><br>ideas…	Things to do:<br><br>✓✓ Call your psychiatrist if they do not already know about the changes in your mood.<br><br>✓✓ Let your supports know that you are having early warning signs of depression.<br><br>✓✓ Use dial up skills. Start moving, warm up, get more active, stay involved.<br><br>✓✓ Keep taking your medications and make sure you are getting proper sleep.	Get real practical now. Take care of yourself.<br><br>Would you like tips?	More often than not, individuals do not reach out to their psychiatrists when perhaps they should. That is normal. That is typical. But it is not so helpful!<br><br>What are your thoughts about talking to your psychiatrist?<br><br>Remember that they signed up to help you! They want to talk with you when you are not doing so well. That is what they are there for! So call!		Look at My Charts in the Wellness Plan. How are you doing? What do you need to do for yourself?<br><br>Get well!

Daily Review Feedback Category 13: Prodromal Continuing, Down – Talk to psychiatrist (Choice 1.0)

Action (P10)	Action (S2)	Action (P6)	Review (U5, P2)		Reduce Risk (P5)
**1**<br>**RANDOM UNLINKED**	**2**<br>**STATIC**	**3**<br>**RANDOM UNLINKED**	**4**<br>**RANDOM UNLINKED**	**5**<br>**BLANK**	**6**<br>**RANDOM UNLINKED**
It seems like you've been down for a while now.<br><br>Now is the time to focus on taking care of yourself. Don't let yourself get depressed.<br><br>Continue for ideas…	Things to do:<br><br>✓✓ Call your psychiatrist if they do not already know about the changes in your mood.<br><br>✓✓ Let your supports know that you are having early warning signs of depression.<br><br>✓✓ Use dial up skills. Start moving, warm up, get more active, stay involved.<br><br>✓✓ Keep taking your medications and make sure you are getting proper sleep.	Feeling down feels bad. Feeling hopeless and helpless can be part of this. Don't let this state of mind control you!<br><br>Get balanced. It is for the best.<br><br>Would you like tips?	If you're thinking that you don't need or don't want to call your psychiatrist…<br><br>✓✓ Review your action plan for Mild Down<br><br>✓✓ Ask your supports what they think		Review your commitments under Reduce Risk in the Wellness Plan.<br><br>Get well!

Daily Review Feedback Category 13: Prodromal Continuing, Down – Talk to supports (Choice 2.0)

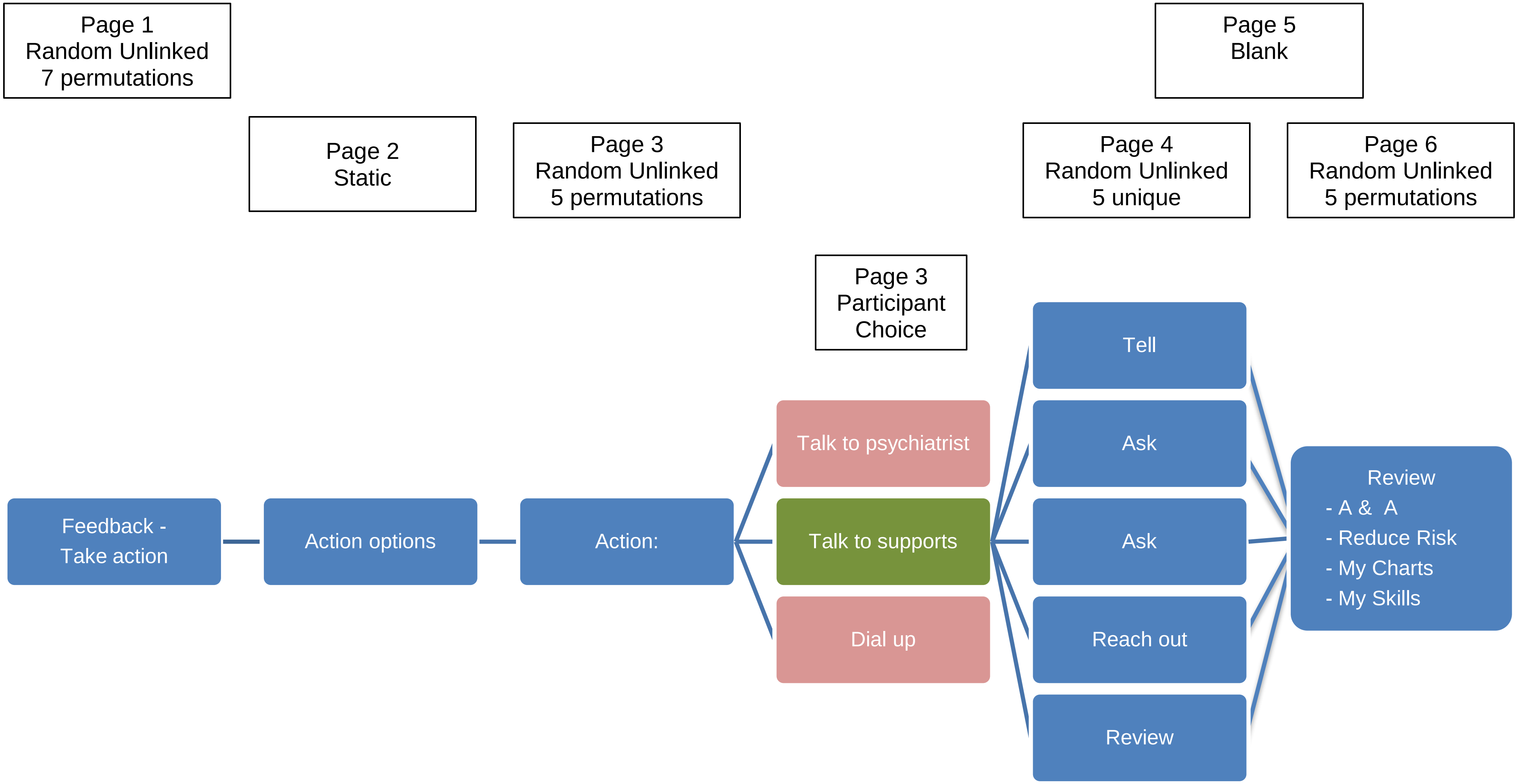

Daily Review Feedback Category 13: Prodromal Continuing, Down – Talk to supports (Choice 2.0)

Action (P6)	Action (S2)	Action (P1)	Tell (U6)		A & A (P6)
**1**<br>**RANDOM UNLINKED**	**2**<br>**STATIC**	**3**<br>**RANDOM UNLINKED**	**4**<br>**RANDOM UNLINKED**	**5**<br>**BLANK**	**6**<br>**RANDOM UNLINKED**
Looks like you've been having early warning signs of depression.<br><br>Now is the time to take action. Don't let your symptoms get worse.<br><br>Continue for ideas...	Things to do:<br><br>✓✓ Call your psychiatrist if they do not already know about the changes in your mood.<br><br>✓✓ Let your supports know that you are having early warning signs of depression .<br><br>✓✓ Use dial up skills. Start moving, warm up, get more active, stay involved.<br><br>✓✓ Keep taking your medications and make sure you are getting proper sleep.	Knowing what is best to do is easy. Actually doing it is another thing!<br><br>Would you like tips?	Things to share with your supports:<br><br>✓✓ Look at your wellness plan. Review your plan for mild symptoms with your supports.<br><br>✓✓ Ask that they check in and gently give you feedback if things seem worse in the days ahead.		Check out your plan for Mild Down in Awareness and Action in the Wellness Plan.<br><br>Get well!

Daily Review Feedback Category 13: Prodromal Continuing, Down – Talk to supports (Choice 2.0)

Action (P7)	Action (S2)	Action (P2)	Ask (U7)		A & A (P7)
**1**<br>**RANDOM UNLINKED**	**2**<br>**STATIC**	**3**<br>**RANDOM UNLINKED**	**4**<br>**RANDOM UNLINKED**	**5**<br>**BLANK**	**6**<br>**RANDOM UNLINKED**
Looks like you've been down for some time now.<br><br>Take action! Don't let yourself get depressed.<br><br>Continue for ideas…	Things to do:<br><br>✓✓ Call your psychiatrist if they do not already know about the changes in your mood.<br><br>✓✓ Let your supports know that you are having early warning signs of depression.<br><br>✓✓ Use dial up skills. Start moving, warm up, get more active, stay involved.<br><br>✓✓ Keep taking your medications and make sure you are getting proper sleep.	Don't fret. Ups and downs are part of bipolar disorder. Everyone has them.<br><br>The key is to be aware and take action.<br><br>Would you like tips?	Ask your supports about what has helped you in the past when you've had early warning signs of depression.<br><br>Remember that your supports are there just to be a sounding board. They are not your boss or therapist.<br><br>Listen to what they have to say. Take them seriously.		Double check your anchors for Mild Down in Awareness and Action in the Wellness Plan.<br><br>Get well!

Daily Review Feedback Category 13: Prodromal Continuing, Down – Talk to supports (Choice 2.0)

Action (P8)	Action (S2)	Action (P3)	Ask (U8)		My Skills (P8)

1<br>RANDOM UNLINKED	2<br>STATIC	3<br>RANDOM UNLINKED	4<br>RANDOM UNLINKED	5<br>BLANK	6<br>RANDOM UNLINKED
You're reporting early warning signs of depression.<br><br>Don't let yourself get depressed. Take action now!<br><br>Continue for ideas…	Things to do:<br><br>✓✓ Call your psychiatrist if they do not already know about the changes in your mood.<br><br>✓✓ Let your supports know that you are having early warning signs of depression .<br><br>✓✓ Use dial up skills. Start moving, warm up, get more active, stay involved.<br><br>✓✓ Keep taking your medications and make sure you are getting proper sleep.	Symptoms can come because of something you did or did not do. Like missing medications, staying out really late, or using drugs.<br><br>Symptoms can also come from out of the blue.<br><br>Either way, don't judge yourself. Just take care of yourself!<br><br>Would you like tips?	Questions to ask your supports:<br><br>✓✓ Why do you think I am having symptoms?<br><br>✓✓ What do you think I should do about it?<br><br>✓✓ When should I check back in?		Look at My Skills in the Wellness Plan. Try Dialing Up.<br><br>Get well!

Daily Review Feedback Category 13: Prodromal Continuing, Down – Talk to supports (Choice 2.0)

Action (P9)	Action (S2)	Action (P4)	Reach out (U9)		My Charts (P4)
**1**<br>**RANDOM UNLINKED**	**2**<br>**STATIC**	**3**<br>**RANDOM UNLINKED**	**4**<br>**RANDOM UNLINKED**	**5**<br>**BLANK**	**6**<br>**RANDOM UNLINKED**
Sounds like you're experiencing early warning signs of depression.<br><br>Take action now!<br><br>Continue for<br><br>ideas…	Things to do:<br><br>✓✓ Call your psychiatrist if they do not already know about the changes in your mood.<br><br>✓✓ Let your supports know that you are having early warning signs of depression .<br><br>✓✓ Use dial up skills. Start moving, warm up, get more active, stay involved.<br><br>✓✓ Keep taking your medications and make sure you are getting proper sleep.	Get real practical now. Take care of yourself.<br><br>Would you like tips?	More often than not, individuals do not reach out to their supports when perhaps they should. That is normal. That is typical. But it is not so helpful!<br><br>What are your thoughts about talking to your supports?<br><br>Remember that they are willing to help you! They want to talk with you when you are not doing so well. That is what they are there for! So call!		Look at My Charts in the Wellness Plan. How are you doing? What do you need to do for yourself?<br><br>Get well!

Daily Review Feedback Category 13: Prodromal Continuing, Down – Talk to supports (Choice 2.0)

Action (P10)	Action (S2)	Action (P6)	Review (U10, P2)		Reduce Risk (P5)
**1**<br>**RANDOM UNLINKED**	**2**<br>**STATIC**	**3**<br>**RANDOM UNLINKED**	**4**<br>**RANDOM UNLINKED**	**5**<br>**BLANK**	**6**<br>**RANDOM UNLINKED**
It seems like you've been down for a while now.<br><br>Now is the time to focus on taking care of yourself. Don't let yourself get depressed.<br><br>Continue for ideas…	Things to do:<br><br>✓✓ Call your psychiatrist if they do not already know about the changes in your mood.<br><br>✓✓ Let your supports know that you are having early warning signs of depression.<br><br>✓✓ Use dial up skills. Start moving, warm up, get more active, stay involved.<br><br>✓✓ Keep taking your medications and make sure you are getting proper sleep.	Feeling down feels bad. Feeling hopeless and helpless can be part of this. Don't let this state of mind control you!<br><br>Get balanced. It is for the<br><br>best. Would you like tips?	If you're thinking that you don't need or don't want to call your supports…<br><br>✓✓ Review your action plan for Mild Down<br><br>✓✓ Ask your psychiatrist what they think		Review your commitments under Reduce Risk in the Wellness Plan.<br><br>Get well!

Daily Review Feedback Category 13: Prodromal - Continuing Down, Dial Up (Choice 3.0)

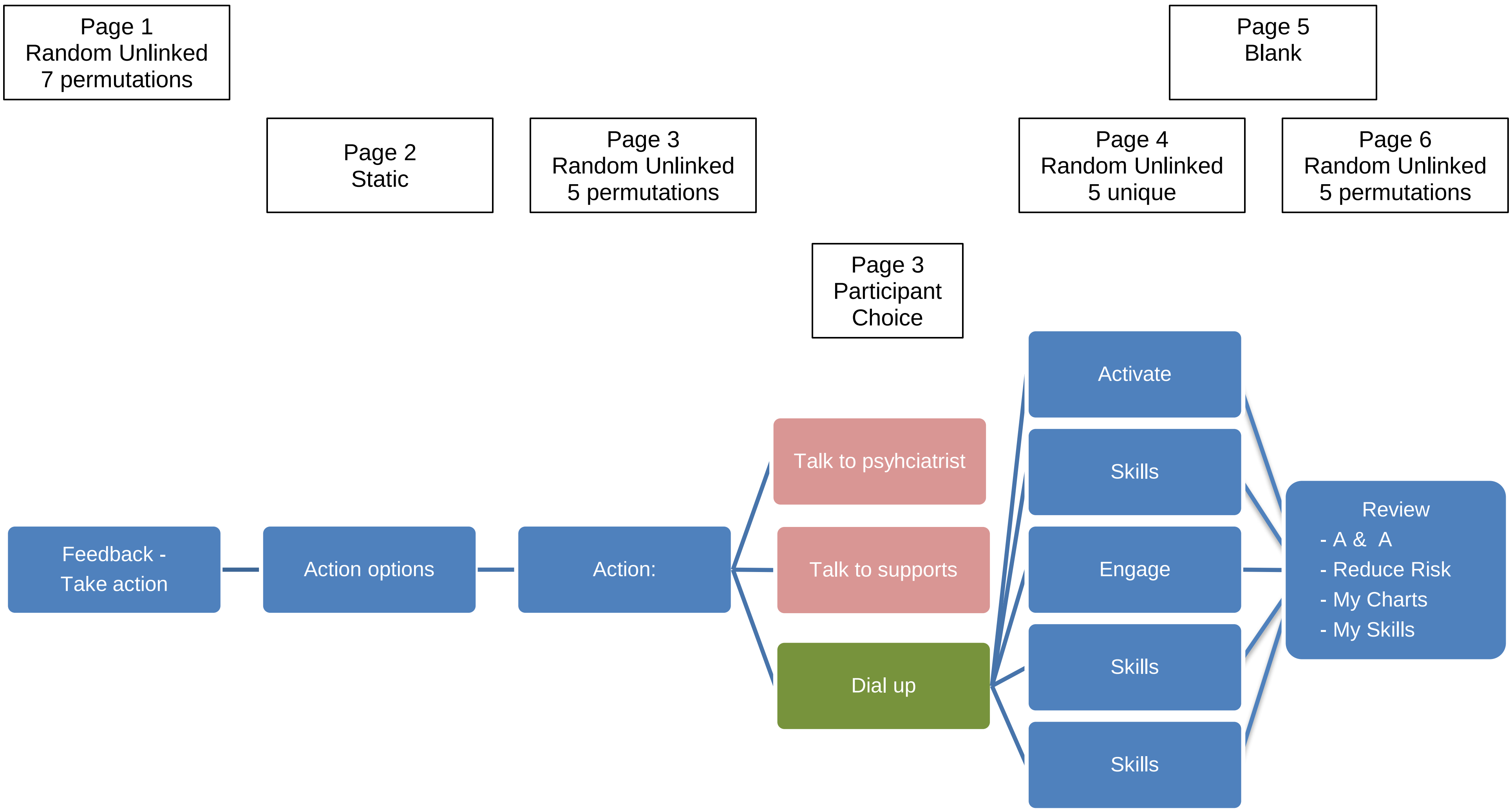

Daily Review Feedback Category 13: Prodromal Continuing, Down – Dial up (Choice 3.0)

Action (P6)	Action (S2)	Action (P1)	Activate (U16)		A & A (P6)
**1**<br>**RANDOM UNLINKED**	**2**<br>**STATIC**	**3**<br>**RANDOM UNLINKED**	**4**<br>**RANDOM UNLINKED**	**5**<br>**BLANK**	**6**<br>**RANDOM UNLINKED**
Looks like you've been having early warning signs of depression.<br><br>Now is the time to take action. Don't let your symptoms get worse.<br><br>Continue for ideas…	Things to do:<br><br>✓✓ Call your psychiatrist if they do not already know about the changes in your mood.<br><br>✓✓ Let your supports know that you are having early warning signs of depression.<br><br>✓✓ Use dial up skills. Start moving, warm up, get more active, stay involved.<br><br>✓✓ Keep taking your medications and make sure you are getting proper sleep.	Knowing what is best to do is easy. Actually doing it is another thing!<br><br>Would you like tips?	Things to consider:<br><br>Inactivity can exacerbate depression. That means when you feel down, getting less active can make things worse.<br><br>Take a few minutes each day to activate your physiology, your body. Move around somehow.<br><br>Move your body. Exercise your mind. Take some deep breaths.<br><br>Pull up a funny video or turn on some music and dance. Heat up your system!<br><br>You'll feel and function better in the long run. Try it!		Check out your plan for Mild Down in Awareness and Action in the Wellness Plan.<br><br>Get well!

Daily Review Feedback Category 13: Prodromal Continuing, Down – Dial up (Choice 3.0)

Action (P7)	Action (S2)	Action (P2)	Skills (U17)		A & A (P7)
**1**<br>**RANDOM UNLINKED**	**2**<br>**STATIC**	**3**<br>**RANDOM UNLINKED**	**4**<br>**RANDOM UNLINKED**	**5**<br>**BLANK**	**6**<br>**RANDOM UNLINKED**
Looks like you've been down for some time now.<br><br>Take action! Don't let yourself get depressed.<br><br>Continue for ideas…	Things to do:<br><br>✓✓ Call your psychiatrist if they do not already know about the changes in your mood.<br><br>✓✓ Let your supports know that you are having early warning signs of depression.<br><br>✓✓ Use dial up skills. Start moving, warm up, get more active, stay involved.<br><br>✓✓ Keep taking your medications and make sure you are getting proper sleep.	Don't fret. Ups and downs are part of bipolar disorder. Everyone has them.<br><br>The key is to be aware and take action.<br><br>Would you like tips?	While it may feel like there is nothing you can do, remember that this state of mind puts you at risk for something worse (clinical depression).<br><br>Take some time to heat up, to dial up.<br><br>Look at Skills or My Skills. Take some time each day to adjust your personal tempo.<br><br>Balanced is good!		Double check your anchors for Mild Down in Awareness and Action in the Wellness Plan.<br><br>Get well!

Daily Review Feedback Category 13: Prodromal Continuing, Down – Dial up (Choice 3.0)

Action (P8)	Action (S2)	Action (P3)	Engage (U18)		My Skills (P8)
**1**<br>**RANDOM UNLINKED**	**2**<br>**STATIC**	**3**<br>**RANDOM UNLINKED**	**4**<br>**RANDOM UNLINKED**	**5**<br>**BLANK**	**6**<br>**RANDOM UNLINKED**
You're reporting early warning signs of depression.<br><br>Don't let yourself get depressed. Take action now!<br><br>Continue for ideas...	Things to do:<br><br>✓✓ Call your psychiatrist if they do not already know about the changes in your mood.<br><br>✓✓ Let your supports know that you are having early warning signs of depression.<br><br>✓✓ Use dial up skills. Start moving, warm up, get more active, stay involved.<br><br>✓✓ Keep taking your medications and make sure you are getting proper sleep.	Symptoms can come because of something you did or did not do. Like missing medications, staying out really late, or using drugs.<br><br>Symptoms can also come from out of the blue.<br><br>Either way, don't judge yourself. Just take care of yourself!<br><br>Would you like tips?	EVERYTHING COUNTS<br><br>Depression is so debilitating. Try to understand that any feelings of hopelessness or helplessness are actually symptoms, not reality.<br><br>Take 10 minutes for yourself. Pick something you can do.<br><br>The act of choosing is healing!<br><br>Do 5 sit---ups. Or walk around the block. Pay a bill. Or just sing a song.<br><br>Put your mind to doing something simple and do it!		Look at My Skills in the Wellness Plan. Try Dialing Up.<br><br>Get well!

Daily Review Feedback Category 13: Prodromal Continuing, Down – Dial up (Choice 3.0)

Action (P9)	Action (S2)	Action (P4)	Skills (U19)		My Charts (P4)
**1**<br>**RANDOM UNLINKED**	**2**<br>**STATIC**	**3**<br>**RANDOM UNLINKED**	**4**<br>**RANDOM UNLINKED**	**5**<br>**BLANK**	**6**<br>**RANDOM UNLINKED**
Sounds like you're experiencing early warning signs of depression.<br><br>Take action now!<br><br>Continue for<br><br>ideas…	Things to do:<br><br>✓✓ Call your psychiatrist if they do not already know about the changes in your mood.<br><br>✓✓ Let your supports know that you are having early warning signs of depression.<br><br>✓✓ Use dial up skills. Start moving, warm up, get more active, stay involved.<br><br>✓✓ Keep taking your medications and make sure you are getting proper sleep.	Get real practical now. Take care of yourself.<br><br>Would you like tips?	DIAL UP<br><br>Set aside 10 minutes for yourself today. Pick a Dial Up skill. You can pick from Skills, My Skills, or just from your own experience.<br><br>The key is to get your blood pressure and heart rate to increase. It is important right now to get your physiology active.		Look at My Charts in the Wellness Plan. How are you doing? What do you need to do for yourself?<br><br>Get well!

Daily Review Feedback Category 13: Prodromal Continuing, Down – Dial up (Choice 3.0)

Action (P10)	Action (S2)	Action (P6)	Skills (U20)		Reduce Risk (P5)
**1**<br>**RANDOM UNLINKED**	**2**<br>**STATIC**	**3**<br>**RANDOM UNLINKED**	**4**<br>**RANDOM UNLINKED**	**5**<br>**BLANK**	**6**<br>**RANDOM UNLINKED**
It seems like you've been down for a while now.<br><br>Now is the time to focus on taking care of yourself. Don't let yourself get depressed.<br><br>Continue for ideas…	Things to do:<br><br>✓✓ Call your psychiatrist if they do not already know about the changes in your mood.<br><br>✓✓ Let your supports know that you are having early warning signs of depression.<br><br>✓✓ Use dial up skills. Start moving, warm up, get more active, stay involved.<br><br>✓✓ Keep taking your medications and make sure you are getting proper sleep.	Feeling down feels bad. Feeling hopeless and helpless can be part of this. Don't let this state of mind control you!<br><br>Get balanced. It is for the<br><br>best. Would you like tips?	DIAL UP<br><br>Set aside 10 minutes for yourself today. Take a look at YouTube. Search for:<br><br>• Dance music<br>• Morning yoga<br>• Exercise programs<br><br>Try it out. See if you can't heat up your system. Aim for balance!		Review your commitments in Reduce Risk in the Wellness Plan.<br><br>Get well!

Daily Review Feedback Category 14: Recovering Improving, Balanced

Page 1	Page 2	Page 3	Page 4	Page 5	Page 6
Random Unlinked<br>5 permutations	Random Unlinked<br>5 unique	Blank	Blank	Blank	Random Unlinked<br>5 permutations

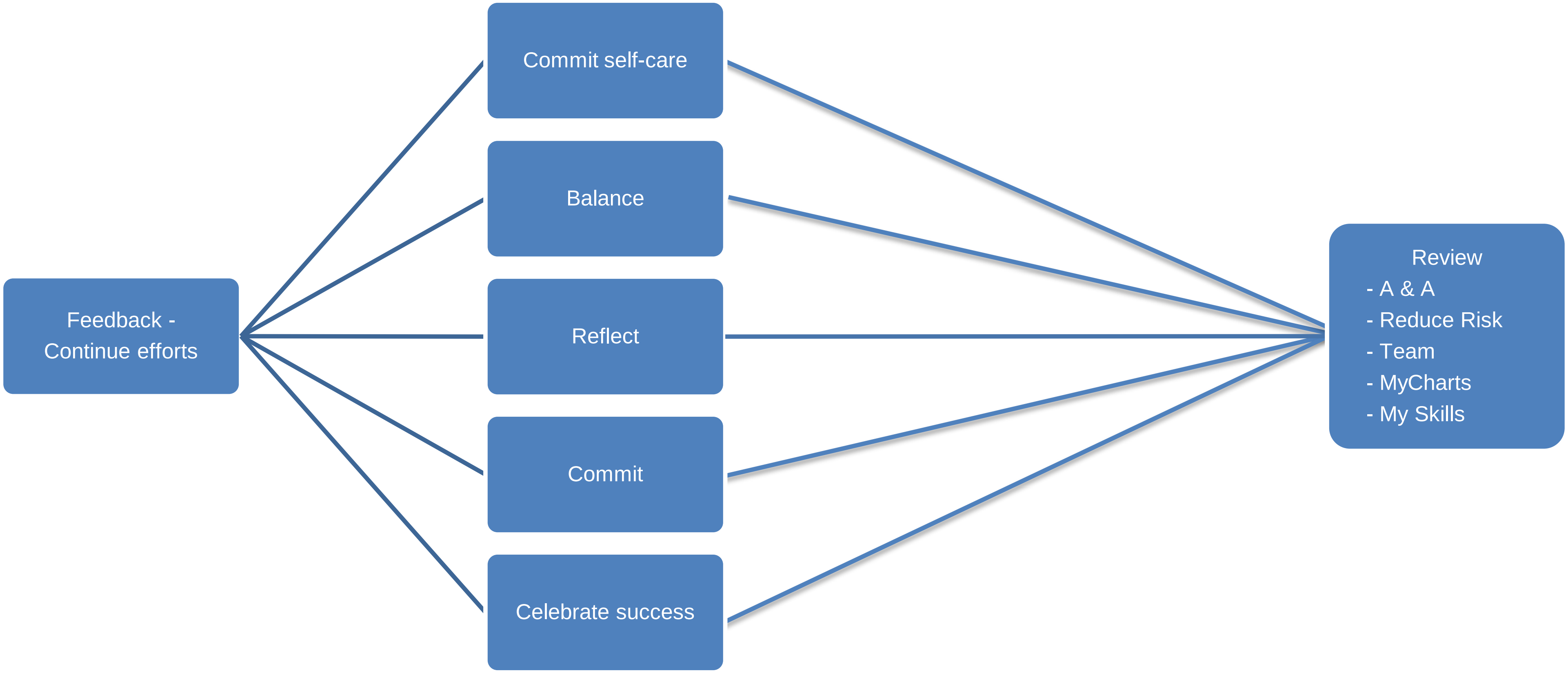

Daily Review Feedback Category 14: Recovering Improving, Balanced

Continue (P1)	Commit (U1)				A & A (P1)
**1**<br>**RANDOM UNLINKED**	**2**<br>**RANDOM UNLINKED**	**3**<br>**BLANK**	**4**<br>**BLANK**	**5**<br>**BLANK**	**6**<br>**RANDOM UNLINKED**
Glad to see that you're feeling better today.<br><br>You seem to be doing much better than you were not so long ago. Keep doing whatever you have been doing. It seems to be working!<br><br>Continue to read more...	Remember that you are at risk for a mood episode until you have a period of time without symptoms. So keep up your efforts in order to stay well!<br><br>Many people find that persistence is the hard part. Just make a goal every morning to continue whatever lifestyle skills, coping skills, and supports you have been using.<br><br>Commit to doing something for yourself at least 15 minutes each day. You can keep it up!<br><br>You can get and stay well!				Review Awareness & Action in the Wellness Plan for ideas.<br><br>Stay well!

Daily Review Feedback Category 14: Recovering Improving, Balanced

Continue (P2) | Balance (U2) | Team (P2)

1<br>RANDOM UNLINKED	2<br>RANDOM UNLINKED	3<br>BLANK	4<br>BLANK	5<br>BLANK	6<br>RANDOM UNLINKED
Recovery is the key. Even though symptoms are likely, what you do when you get them makes all the difference when it comes to your health.<br><br>Something is going right. Keep it up!	Being balanced is best for your brain and your life.<br><br>Take more time to ensure you are recovered.<br><br>It's Goldilocks. Not too much energy and not too little energy. Aim for the middle.				Take a look at My Resources in the Wellness Plan. Don't forget you have people that care.<br><br>Stay well!

Daily Review Feedback Category 14: Recovering Improving, Balanced

Continue (P3)	Reflect (U3)				Reduce Risk (P3)
**1**<br>**RANDOM UNLINKED**	**2**<br>**RANDOM UNLINKED**	**3**<br>**BLANK**	**4**<br>**BLANK**	**5**<br>**BLANK**	**6**<br>**RANDOM UNLINKED**
Looks like things are headed in the right direction.<br><br>Remember that balance is the key.	What are you like when balanced?<br><br>• What is your personality like?<br>• How about your outlook on life?<br>• What do you tend to think about?<br>• How do you tend to generally feel?				Review Reduce Risk in the Wellness Plan. Perhaps there are some lifestyle habits you might want to incorporate into your daily routine.<br><br>Stay well!

Daily Review Feedback Category 14: Recovering Improving, Balanced

Continue (P4) | Commit (U4) | My Charts (P4)

1<br>RANDOM UNLINKED	2<br>RANDOM UNLINKED	3<br>BLANK	4<br>BLANK	5<br>BLANK	6<br>RANDOM UNLINKED
It seems like you're well on your way to regaining your sense of balance. Keep doing whatever you are doing as it seems to be moving you in the right direction.<br><br>Continue to read more…	Ups and downs are part of bipolar disorder. While this can be very uncomfortable, know that you are not at fault. Know that you are not alone.<br><br>Look at your schedule each morning. Make sure that you plan on doing something, but not too much.				Take a look at My Charts in the Wellness Plan. Do you see any patterns?<br><br>Stay well!

Daily Review Feedback Category 14: Recovering Improving, Balanced

Continue (P5)	Success (U5)				My Skills (P5)
**1**<br>**RANDOM UNLINKED**	**2**<br>**RANDOM UNLINKED**	**3**<br>**BLANK**	**4**<br>**BLANK**	**5**<br>**BLANK**	**6**<br>**RANDOM UNLINKED**
Aim for balance in life.<br><br>Seek out people and activities that help you stay centered, positive, and hopeful.<br><br>By the looks of it, you're on your way to wellness. Keep up the good work!	Feel good about whatever actions you have taken to move towards Balanced.<br><br>Bipolar disorder is a difficult illness. While it can definitely be managed, every success should be celebrated.				Look at My Skills in My Resources in the Wellness Plan. Do something for yourself today!<br><br>Stay well!

Daily Review Feedback Category 15: Prodromal Improving, Balanced

Page 1	Page 2	Page 3	Page 4	Page 5	Page 6
Random Unlinked<br>5 permutations	Random Unlinked<br>5 unique	Blank	Blank	Blank	Random Unlinked<br>5 permutations

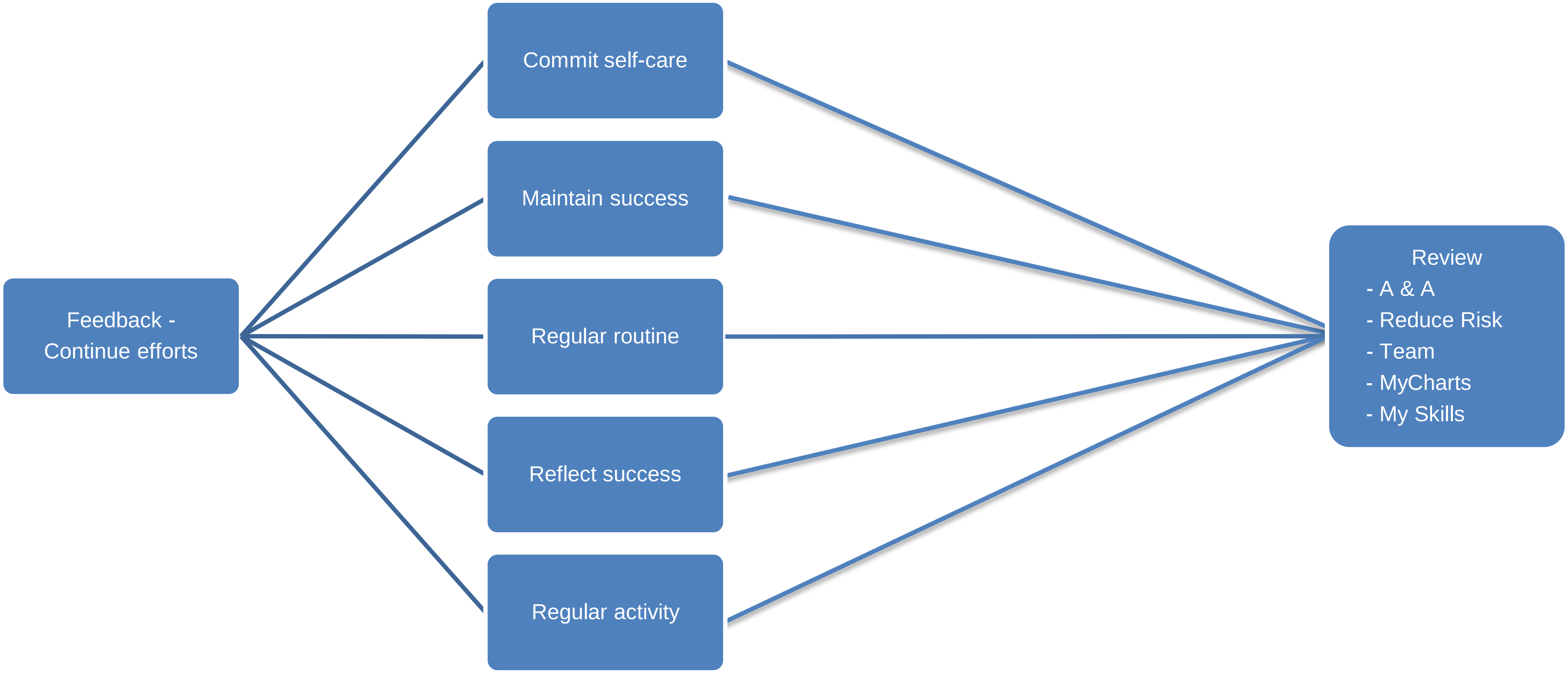

Daily Review Feedback Category 15: Prodromal Improving, Balanced

Continue (P1)	Commit (U1)				A & A (P1)
**1<br>RANDOM UNLINKED**	**2<br>RANDOM UNLINKED**	**3<br>BLANK**	**4<br>BLANK**	**5<br>BLANK**	**6<br>RANDOM UNLINKED**
Glad to see you're feeling better today. You were reporting possible early warning signs not so long ago.<br><br>Keep doing whatever you have been doing. It seems to be working!	Remember that you are at risk for a mood episode until you have a period of time without early warning signs or symptoms. So keep up your efforts in order to stay well!<br><br>Many people find that persistence is the hard part. Just make a goal every morning to continue whatever lifestyle skills, coping skills, and supports you have been using.<br><br>Commit to doing something for yourself at least 15 minutes each day. You can keep it up!<br><br>You can get and stay well!				Review Awareness & Action in the Wellness Plan for ideas.<br><br>Stay well!

Daily Review Feedback Category 15: Prodromal Improving, Balanced

Continue (P2)	Maintain (U2)				A & A (P2)
**1**<br>**RANDOM UNLINKED**	**2**<br>**RANDOM UNLINKED**	**3**<br>**BLANK**	**4**<br>**BLANK**	**5**<br>**BLANK**	**6**<br>**RANDOM UNLINKED**
Balance is the key.<br><br>It's great to see that you are doing better.<br><br>What has helped your recovery?	Don't give up on your efforts. Getting balanced and staying balanced are two different things.<br><br>Take whatever you have done to get well and keep doing it!				Review your anchors for Balanced under Awareness & Action in the Wellness Plan.<br><br>Stay balanced!

Daily Review Feedback Category 15: Prodromal Improving, Balanced

Continue (P3)	Routine (U3)				A & A (P3)
**1**<br>**RANDOM UNLINKED**	**2**<br>**RANDOM UNLINKED**	**3**<br>**BLANK**	**4**<br>**BLANK**	**5**<br>**BLANK**	**6**<br>**RANDOM UNLINKED**
You're reporting being balanced. Glad to hear it!<br><br>Keep up the good work.	Managing bipolar disorder is about regulation.<br><br>It's about getting a good rhythm in life. That is, it's about when you are active and inactive.<br><br>Stick to a schedule and see is you don't feel and function better!				Review your action plan for Balanced under Awareness & Action in the Wellness Plan.

Daily Review Feedback Category 15: Prodromal Improving, Balanced

Continue (P4) | Reflect (U4) | My Skills (P4)

1<br>RANDOM UNLINKED	2<br>RANDOM UNLINKED	3<br>BLANK	4<br>BLANK	5<br>BLANK	6<br>RANDOM UNLINKED
Is seems your symptoms are resolving. That is good to hear.<br><br>What do you need to do to stay on track?	Look back at this past week. What have you done that has promoted balance? What have you done that has contributed to unbalance?<br><br>What do you want to do in the week ahead?				Review My Skills for Tranquility in the Wellness Plan.

Daily Review Feedback Category 15: Prodromal Improving, Balanced

Continue (P5)

Activity (U5)

My Charts (P5)

1<br>RANDOM UNLINKED	2<br>RANDOM UNLINKED	3<br>BLANK	4<br>BLANK	5<br>BLANK	6<br>RANDOM UNLINKED
Good to see you're feeling balanced today.<br><br>To keep things moving in the right direction, try to stay even. Stick to people and activities that help you stay centered, positive, and hopeful.	Do something daily to stay grounded. Yoga, meditation, mindfulness exercises, or something else.<br><br>Is there some ritual you can do each and every day, about the same time each day, to regulate your body? (And your moods…)				Check out My Charts in the Wellness Plan. Do you see any patterns?

Daily Review Feedback Category 16: Early Warning Signs, Up

Page 1<br>Random Unlinked<br>5 5ermutations	Page 2<br>Static	Page 3<br>Static	Page 4<br>Random Unlinked<br>5 permutations	Page 5<br>Random Unlinked<br>5 unique	Page 6<br>Random Unlinked<br>4 permutations

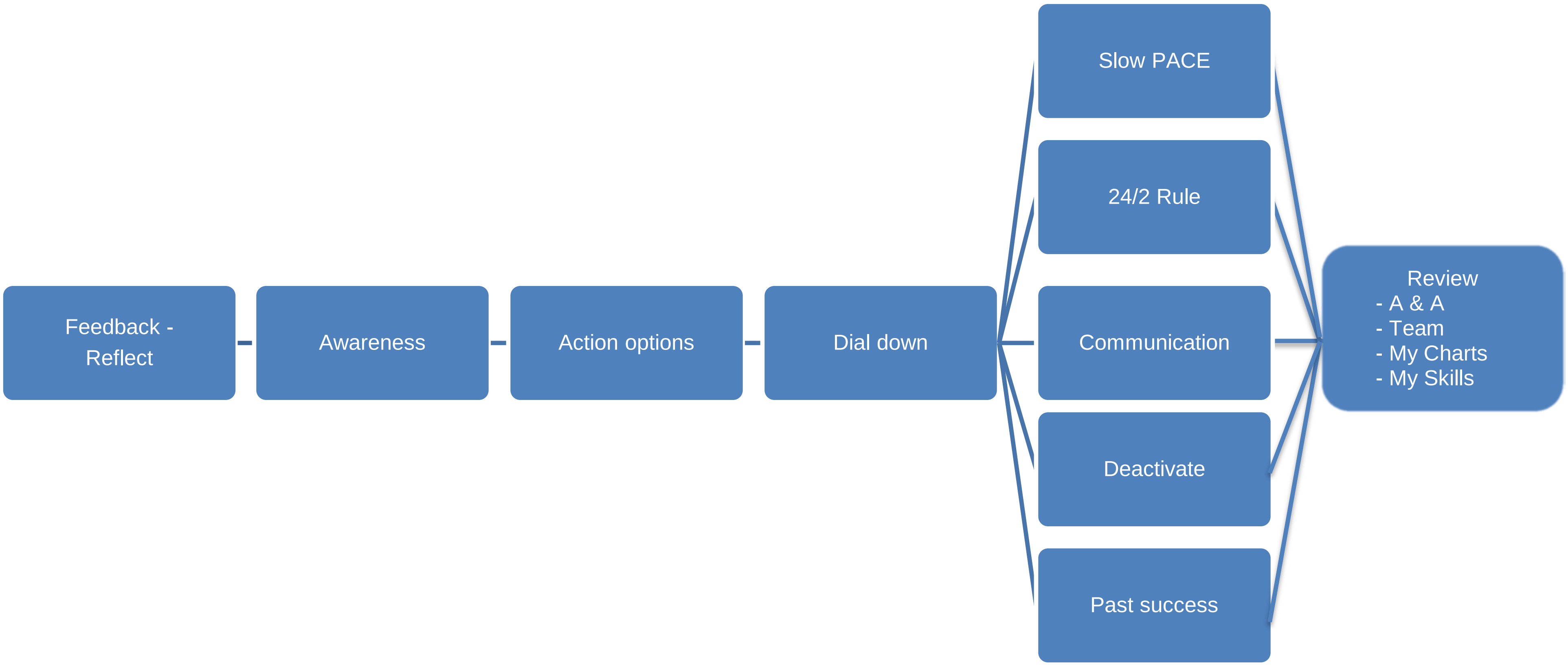

Daily Review Feedback Category 16: Early Warning Signs, Up

Reflection (P1)	Awareness (S1)	Action (S1)	Dial down (P1)	Slow PACE (U1)	A & A (P1)
**1**<br>**RANDOM UNLINKED**	**2**<br>**STATIC**	**3**<br>**STATIC**	**4**<br>**RANDOM UNLINKED**	**5**<br>**RANDOM UNLINKED**	**6**<br>**RANDOM UNLINKED**
You're reporting being mildly up today. Seems like a change from how you've been doing overall.<br><br>Pause, reflect, and follow your action plan.<br><br>Continue for ideas…	AWARENESS<br><br>Check it out. How many of the following signs are you experiencing?<br><br>△ Energetic/very active<br>△ Feeling emotionally high<br>△ More talkative<br>△ Cannot get off to sleep<br>△ Not needing much sleep<br>△ Racing thoughts<br>△ Ideas flowing too fast<br>△ Difficulty concentrating<br>△ Senses seem sharper<br><br>If you are experiencing one or more of these early warning signs of mania, consider taking action.	ACTION<br><br>Things you can do:<br><br>✓✓ Call your psychiatrist if they do not already know about the changes in how you are doing.<br><br>✓✓ Let your supports know that you may be having early warning signs of mania.<br><br>✓✓ Use dial down skills. Slow down, cool down, get less active, calm your body.<br><br>✓✓ Keep taking your medications and make sure you are getting enough sleep.	MORE ON DIALING DOWN<br><br>Mania involves the OVER---ACTIVATION of your system. Your thoughts, behaviors, feelings, and body are all operating at a higher than normal (for you) level.<br><br>Use dial down skills to counteract this process. Slow down, cool down, get less active, calm yourself.<br><br>When dialing down:<br><br>• Pace your actions<br>• Pace your decisions<br>• Pace your socialization<br><br>Aim for a moderate personal tempo!	PACED Activities<br><br>Slowing your body repairs mild ups in mood. PACE yourself. Slow down!<br><br>Not everything is urgent. Set goals so that you don't overdo it:<br><br>**P**riority: Stick to high priority tasks. Everything cannot be important.<br><br>**A**ttitude: Stay mindful and grounded. Not all ideas are great in the long run.<br><br>**C**ommonsensical: Make sure the tasks fit into your life plan.<br><br>**E**ven: Keep a balance of activity and rest. Go slower than you are inclined.<br><br>BE THOUGHTFUL. GO SLOW!	Check out your plan for mild up in Awareness and Action in the Wellness Plan.<br><br>Get well!

Daily Review Feedback Category 16: Early Warning Signs, Up

Reflection (P2)	Awareness (S1)	Action (S1)	Dial down (P2)	24/2 Rule (U2)	A & A (P2)

1<br>**RANDOM UNLINKED**	2<br>**STATIC**	3<br>**STATIC**	4<br>**RANDOM UNLINKED**	5<br>**RANDOM UNLINKED**	6<br>**RANDOM UNLINKED**
Looks like your mood has shifted up. Take a few minutes to consider how you are doing.<br><br>Continue for ideas…	AWARENESS<br><br>Check it out. How many of the following signs are you experiencing?<br><br>∆ Energetic/very active<br>∆ Feeling emotionally high<br>∆ More talkative<br>∆ Cannot get off to sleep<br>∆ Not needing much sleep<br>∆ Racing thoughts<br>∆ Ideas flowing too fast<br>∆ Difficulty concentrating<br>∆ Senses seem sharper<br><br>If you are experiencing one or more of these early warning signs of mania, consider taking action.	ACTION<br><br>Things you can do:<br><br>✓✓ Call your psychiatrist if they do not already know about the changes in how you are doing.<br><br>✓✓ Let your supports know that you may be having early warning signs of mania.<br><br>✓✓ Use dial down skills. Slow down, cool down, get less active, calm your body.<br><br>✓✓ Keep taking your medications and make sure you are getting enough sleep.	MORE ON DIALING DOWN<br><br>Remember that mania involves an OVER---ACTIVATION of your biological systems. That means your thoughts, behaviors, feelings, and body are all operating at a higher intensity than usual for you.<br><br>You can take steps to reverse this process. Slow down, cool down, get less active, calm yourself.<br><br>When dialing down:<br><br>• Pace your actions<br>• Pace your decisions<br>• Pace your socialization<br><br>Aim for a moderate speed!	24/2 Rule<br><br>When you are feeling mildly up, just slow down. You don't want to get so excited that you start saying or doing things that you'll later regret.<br><br>When mildly up and excited about something big, pause before you make a decision:<br><br>• Wait twenty four hours and reassess. Does it still seem like a good idea?<br>• Ask two people what they think. Do they agree that it's a good idea?<br><br>Mania involves a pressure to act. Don't follow the pressure. Follow your wisdom!<br><br>Pace yourself!	Take a look at your anchors for Mild Up in Awareness and Action in the Wellness Plan.<br><br>Get well…

Daily Review Feedback Category 16: Early Warning Signs, Up

Reflection (P3)	Awareness (S1)	Action (S1)	Dial down (P3)	Communication (U3)	My Skills (P3)
**1**<br>**RANDOM UNLINKED**	**2**<br>**STATIC**	**3**<br>**STATIC**	**4**<br>**RANDOM UNLINKED**	**5**<br>**RANDOM UNLINKED**	**6**<br>**RANDOM UNLINKED**
Seems like your mood is elevated. Is it possible that you're experiencing early warning signs of mania?<br><br>Now is the time to take a closer look at things.<br><br>Continue to read more…	AWARENESS<br><br>Check it out. How many of the following signs are you experiencing?<br><br>∆ Energetic/very active<br>∆ Feeling emotionally high<br>∆ More talkative<br>∆ Cannot get off to sleep<br>∆ Not needing much sleep<br>∆ Racing thoughts<br>∆ Ideas flowing too fast<br>∆ Difficulty concentrating<br>∆ Senses seem sharper<br><br>If you are experiencing one or more of these early warning signs of mania, consider taking action.	ACTION<br><br>Things you can do:<br><br>✓✓ Call your psychiatrist if they do not already know about the changes in how you are doing.<br><br>✓✓ Let your supports know that you may be having early warning signs of mania.<br><br>✓✓ Use dial down skills. Slow down, cool down, get less active, calm your body.<br><br>✓✓ Keep taking your medications and make sure you are getting enough sleep.	MORE ON DIALING DOWN<br><br>Mania involves the OVER---ACTIVATION of your system. There is more energy driving your thoughts, behaviors, feelings, and body. There is also more energy driving your socialization.<br><br>Use dial down skills to counteract this process. Slow down, cool down, get less social, get quiet.<br><br>When dialing down:<br><br>• Pace your actions<br>• Pace your decisions<br>• Pace your<br><br>socialization Aim for<br><br>balance!	TAKE THE BACK SEAT<br><br>Positive contact repairs mild ups in mood. Do not overdo it. Take it easy! Let others drive.<br><br>DO:<br>• Let others initiate conversations<br>• Actively listen<br><br>DON'T:<br>• Don't cut others off<br>• Don't force others to agree<br><br>Make a list of things to tell others that are simple. Be sure to participate. Be more passive, less active. Get quiet.	Take a look at My Skills in My Resources in the Wellness Plan. Dial down!<br><br>Get well…

Daily Review Feedback Category 16: Early Warning Signs, Up

Reflection (P4)	Awareness (S1)	Action (S1)	Dial down (P4)	Deactivate (U4)	My Charts (P4)

1<br>RANDOM UNLINKED	2<br>STATIC	3<br>STATIC	4<br>RANDOM UNLINKED	5<br>RANDOM UNLINKED	6<br>RANDOM UNLINKED
Looks like there has been a change in your wellness.<br><br>Now is the time for awareness and action.<br><br>Continue for ideas…	AWARENESS<br><br>Check it out. How many of the following signs are you experiencing?<br><br>△ Energetic/very active<br>△ Feeling emotionally high<br>△ More talkative<br>△ Cannot get off to sleep<br>△ Not needing much sleep<br>△ Racing thoughts<br>△ Ideas flowing too fast<br>△ Difficulty concentrating<br>△ Senses seem sharper<br><br>If you are experiencing one or more of these early warning signs of mania, consider taking action.	ACTION<br><br>Things you can do:<br><br>✓✓ Call your psychiatrist if they do not already know about the changes in how you are doing.<br><br>✓✓ Let your supports know that you may be having early warning signs of mania.<br><br>✓✓ Use dial down skills. Slow down, cool down, get less active, calm your body.<br><br>✓✓ Keep taking your medications and make sure you are getting enough sleep.	MORE ON DIALING DOWN<br><br>Mania involves the OVER---ACTIVATION of your system. Your thoughts, behaviors, feelings, and body are all operating more intensely than usual.<br><br>Use dial down skills to counteract this process. Slow down, cool down, get less active, calm yourself.<br><br>Aim for balance. Aim for peace.	Deactivate Your Body<br><br>Slowing your body repairs mild ups in mood. Put on the brakes. Get your body to be quiet on the inside. Be still!<br><br>RELAX…<br><br>Check out YouTube videos:<br><br>• Mindfulness<br>• Guided imagery<br>• Progressive muscle relaxation<br>• Evening yoga<br><br>Try to relax for a full day. It will be challenging at first. But you can do it.<br><br>CALM YOUR BODY. GROUND YOURSELF!	Take a look at My Charts in the Wellness Plan. Do you notice any patterns? Anything significant about your health?<br><br>Get well…

Daily Review Feedback Category 16: Early Warning Signs, Up

Reflection (P5)	Awareness (S1)	Action (S1)	Dial down (P5)	Past success (U5)	Team (P5)
**1**<br>**RANDOM UNLINKED**	**2**<br>**STATIC**	**3**<br>**STATIC**	**4**<br>**RANDOM UNLINKED**	**5**<br>**RANDOM UNLINKED**	**6**<br>**RANDOM UNLINKED**
You're reporting having mild symptoms or early warning signs of mania.<br><br>Take some time to check in with yourself and make a plan of action.<br><br>Continue for ideas…	AWARENESS<br><br>Check it out. How many of the following signs are you experiencing?<br><br>∆ Energetic/very active<br>∆ Feeling emotionally high<br>∆ More talkative<br>∆ Cannot get off to sleep<br>∆ Not needing much sleep<br>∆ Racing thoughts<br>∆ Ideas flowing too fast<br>∆ Difficulty concentrating<br>∆ Senses seem sharper<br><br>If you are experiencing one or more of these early warning signs of mania, consider taking action.	ACTION<br><br>Things you can do:<br><br>✓✓ Call your psychiatrist if they do not already know about the changes in how you are doing.<br><br>✓✓ Let your supports know that you may be having early warning signs of mania.<br><br>✓✓ Use dial down skills. Slow down, cool down, get less active, calm your body.<br><br>✓✓ Keep taking your medications and make sure you are getting enough sleep.	MORE ON DIALING DOWN<br><br>Mania involves the OVER---ACTIVATION of your system. Your thoughts, behaviors, feelings, and body are all stimulated.<br><br>Use dial down skills to counteract this process. Slow down, cool down, get quiet, calm yourself.<br><br>Aim for a moderation.	Do It Yourself<br><br>What have you done in the past to calm down? When you were stressed, or mad, or manic? What works for you?<br><br>Although there are standard Dial Down skills like 24/2 and progressive muscle relaxation, everyone is different. Some people find gardening relaxing, some people find cooking soothing. Others find sitting by the lake or taking a walk with their dog de---stimulating.<br><br>Think back, what works for you? Why not try it now?	If you are thinking that you don't need to take any action now, be sure to run this by your supports for objective feedback. Review your team in the Wellness Plan.<br><br>Get well…

Daily Review Feedback Category 17: Early Warning Signs, Down

Page 1	Page 2	Page 3	Page 4	Page 5	Page 6
Random Unlinked<br>5 permutations	Static	Static	Random Unlinked<br>5 permutations	Random Unlinked<br>0 permutations	Random Unlinked<br>4 permutations

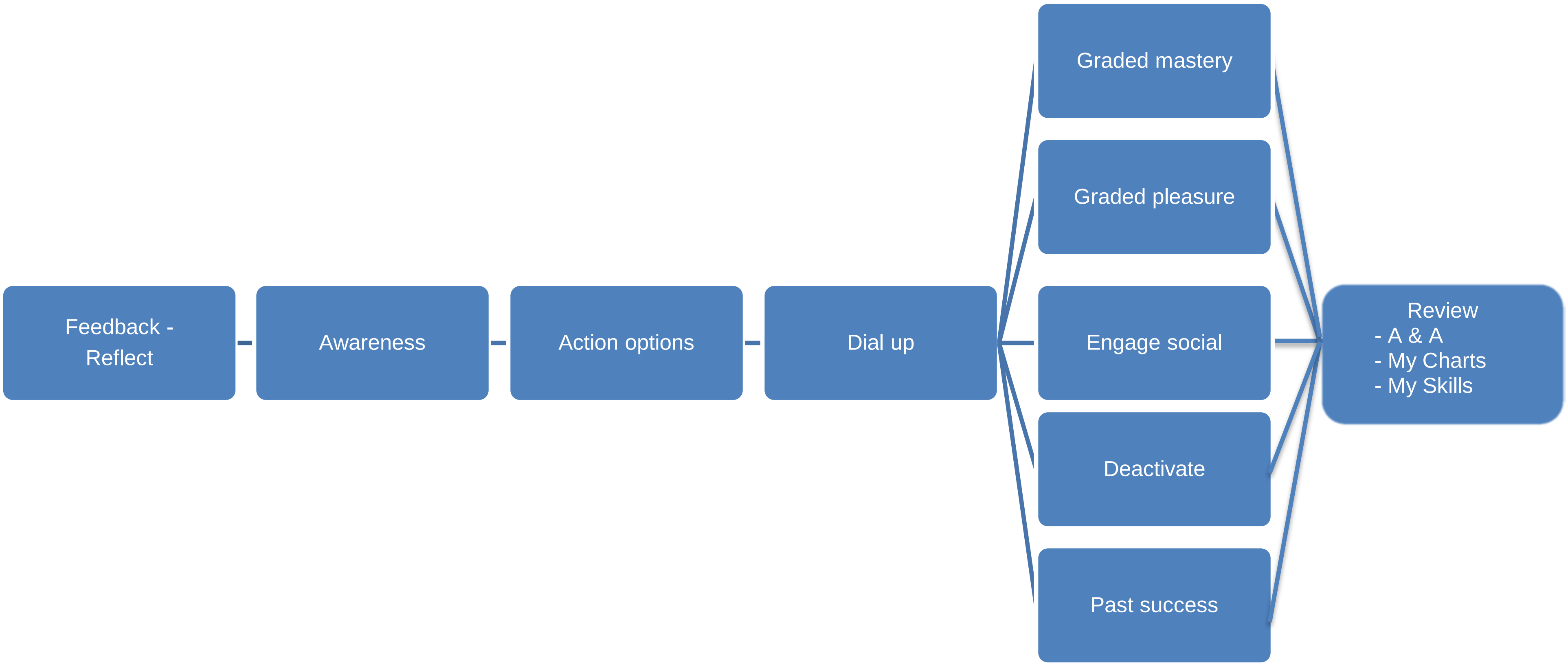

Daily Review Feedback Category 17: Early Warning Signs, Down

Reflection (P6)	Awareness (S2)	Action (S2)	Dial up (P6)	Graded mastery (U6)	A & A (P6)
**1**<br>**RANDOM UNLINKED**	**2**<br>**STATIC**	**3**<br>**STATIC**	**4**<br>**RANDOM UNLINKED**	**5**<br>**RANDOM UNLINKED**	**6**<br>**RANDOM UNLINKED**
You're reporting being mildly down today. Seems like a change from how you've been doing overall.<br><br>Now is the time for awareness and action.<br><br>Continue for ideas...	AWARENESS<br><br>Check it out. How many of the following signs are you experiencing?<br><br>△ Low motivation/can't get started<br>△ Low in energy<br>△ Feeling tired/listless<br>△ Loss of interest in activities<br>△ Difficulty concentrating<br>△ Want to be alone<br>△ Less talkative<br>△ Negative thoughts pop into my mind<br>△ Loss of interest in people<br>△ Ideas slowed down<br>△ Less interest in sex<br>△ Cannot get off to sleep<br>△ Interrupted sleep<br><br>If you are experiencing one or more of these early warning signs of depression, consider taking action.	ACTION<br><br>Things you can do:<br><br>✓ Call your psychiatrist if they do not already know about the changes in how you are doing.<br><br>✓ Let your supports know that you are having early warning signs of depression.<br><br>✓ Use dial up skills. Start moving, warm up, get more active, stay involved.<br><br>✓ Keep taking your medications and make sure you are getting the right amount of sleep.	MORE ON DIALING UP<br><br>Depression involves the UNDER-ACTIVATION of your system. Your thoughts, behaviors, feelings, and body are all operating at a lower than normal (for you) level.<br><br>Use dial up skills to counteract this process. Start moving, warm up, get more active, stay involved.<br><br>When dialing up:<br><br>• Get more active<br>• Get more involved<br>• Get more social<br><br>Aim for a moderate personal tempo!	Graded Mastery<br><br>Accomplishments repair mild drops in mood. Start <u>easy</u> and slow. But go!<br><br>• Eat a healthy meal<br>• Exercise for any length of time<br>• Take a shower and dress nicely<br>• Finish a chore around home<br>• Finish a task for school<br>• Finish a task at work<br><br>Make a checklist of things that make you feel capable. Start by doing one easy thing. Then do another.<br><br>This is hard to do. Get started. Go easy.<br><br>START EASY! GO SLOW!	Check out your plan for Mild Down in Awareness and Action in the Wellness Plan.<br><br>Get well...

Daily Review Feedback Category 17: Early Warning Signs, Down

Reflection (P7)	Awareness (S2)	Action (S2)	Dial up (P7)	Graded pleasure (U7)	A & A (P7)
**1**<br>**RANDOM UNLINKED**	**2**<br>**STATIC**	**3**<br>**STATIC**	**4**<br>**RANDOM UNLINKED**	**5**<br>**RANDOM UNLINKED**	**6**<br>**RANDOM UNLINKED**
Looks like your mood has shifted down.<br><br>Take a few minutes to check in with yourself. Is it possible that you're experiencing early warning signs of depression?<br><br>Continue to read more...	AWARENESS<br><br>Check it out. How many of the following signs are you experiencing?<br><br>△ Low motivation/can't get started<br>△ Low in energy<br>△ Feeling tired/listless<br>△ Loss of interest in activities<br>△ Difficulty concentrating<br>△ Want to be alone<br>△ Less talkative<br>△ Negative thoughts pop into my mind<br>△ Loss of interest in people<br>△ Ideas slowed down<br>△ Less interest in sex<br>△ Cannot get off to sleep<br>△ Interrupted sleep<br><br>If you are experiencing one or more of these early warning signs of depression, consider taking action.	ACTION<br><br>Things you can do:<br><br>✓ Call your psychiatrist if they do not already know about the changes in how you are doing.<br><br>✓ Let your supports know that you are having early warning signs of depression.<br><br>✓ Use dial up skills. Start moving, warm up, get more active, stay involved.<br><br>✓ Keep taking your medications and make sure you are getting the right amount of sleep.	MORE ON DIALING UP<br><br>Depression involves the UNDER-ACTIVATION of your system. Your thoughts, behaviors, feelings, and body are all operating at a lower intensity than usual.<br><br>Use Dial Up skills to counteract this process. Start moving, warm up, get more active, stay involved.<br><br>When dialing up:<br><br>• Get more active<br>• Get more involved<br>• Get more social<br><br>Aim for a moderate<br><br>tempo!	Graded Pleasure<br><br>Fun repairs mild drops in mood. Start <u>easy</u> and slow. But go!<br><br>• Take a bath<br>• Watch a comedy<br>• Spend time with your pet<br>• Have a cup of tea<br>• Get a massage<br>• Play a game of chess<br><br>Make a checklist of things that you enjoy when well. Start by doing one easy thing. Then do another.<br><br>START SIMPLE! <u>GO</u> SLOW!	Check out your anchors for Mild Down in Awareness and Action in the Wellness Plan.<br><br>Get well...

Daily Review Feedback Category 17: Early Warning Signs, Down

Reflection (P8)	Awareness (S2)	Action (S2)	Dial up (P8)	Engage social (U8)	My Skills (P8)
**1**<br>**RANDOM UNLINKED**	**2**<br>**STATIC**	**3**<br>**STATIC**	**4**<br>**RANDOM UNLINKED**	**5**<br>**RANDOM UNLINKED**	**6**<br>**RANDOM UNLINKED**
It looks like you're a bit down today. Seems like a change from how you've been doing overall.<br><br>Pause, reflect, and follow your action plan.<br><br>Continue for ideas…	AWARENESS<br><br>Check it out. How many of the following signs are you experiencing?<br><br>∆ Low motivation/can't get started<br>∆ Low in energy<br>∆ Feeling tired/listless<br>∆ Loss of interest in activities<br>∆ Difficulty concentrating<br>∆ Want to be alone<br>∆ Less talkative<br>∆ Negative thoughts pop into my mind<br>∆ Loss of interest in people<br>∆ Ideas slowed down<br>∆ Less interest in sex<br>∆ Cannot get off to sleep<br>∆ Interrupted sleep<br><br>If you are experiencing one or more of these early warning signs of depression, consider taking action.	ACTION<br><br>Things you can do:<br><br>✓ Call your psychiatrist if they do not already know about the changes in how you are doing.<br><br>✓ Let your supports know that you are having early warning signs of depression.<br><br>✓ Use dial up skills. Start moving, warm up, get more active, stay involved.<br><br>✓ Keep taking your medications and make sure you are getting the right amount of sleep.	MORE ON DIALING UP<br><br>Depression involves the UNDER-ACTIVATION of your system. Your thoughts, behaviors, feelings, and body are all operating at a lower than usual level.<br><br>Use Dial Up skills to counteract this process. Move your body!<br><br>When dialing up:<br><br>• Get more active<br>• Get more involved<br>• Get more social<br><br>Aim for a moderate<br><br>speed!	Increase Contact<br><br>Maybe it's the last thing you want to do! But when you're down, it's exactly what you need!<br><br>Contact with others repairs mild drops in mood. Start easy and slow. But go!<br><br>• Initiate a conversation<br>• Ask questions<br>• Actively listen<br>• Share something about yourself<br>• Talk about the news<br><br>Make a list of things to tell others that are simple. Be sure to participate. Be active, not passive. Not too challenging.<br><br>START SMALL! BE PRESENT!	Check out My Skills in My Resources in the Wellness Plan. What might you do to take care of yourself?<br><br>Get well…

Daily Review Feedback Category 17: Early Warning Signs, Down

Reflection (P4)	Awareness (S2)	Action (S2)	Dial up (P9)	Activate (U9)	My Charts (P4)

**1**<br>**RANDOM UNLINKED**	**2**<br>**STATIC**	**3**<br>**STATIC**	**4**<br>**RANDOM UNLINKED**	**5**<br>**RANDOM UNLINKED**	**6**<br>**RANDOM UNLINKED**
Looks like there has been a change in your wellness.<br><br>Now is the time to take a closer look at things.<br><br>Continue for ideas…	AWARENESS<br><br>Check it out. How many of the following signs are you experiencing?<br><br>△ Low motivation/can't get started<br>△ Low in energy<br>△ Feeling tired/listless<br>△ Loss of interest in activities<br>△ Difficulty concentrating<br>△ Want to be alone<br>△ Less talkative<br>△ Negative thoughts pop into my mind<br>△ Loss of interest in people<br>△ Ideas slowed down<br>△ Less interest in sex<br>△ Cannot get off to sleep<br>△ Interrupted sleep<br><br>If you are experiencing one or more of these early warning signs of depression, consider taking action.	ACTION<br><br>Things you can do:<br><br>✓ Call your psychiatrist if they do not already know about the changes in how you are doing.<br><br>✓ Let your supports know that you are having early warning signs of depression.<br><br>✓ Use dial up skills. Start moving, warm up, get more active, stay involved.<br><br>✓ Keep taking your medications and make sure you are getting the right amount of sleep.	MORE ON DIALING UP<br><br>Depression involves the UNDER-ACTIVATION of your system. Your thoughts, behaviors, feelings, and body are all dampened.<br><br>Use Dial Up skills to counteract this process. Start moving, warm up, get more active, stay involved.<br><br>When dialing up:<br><br>• Get more active<br>• Get more involved<br>• Get more<br><br>social Aim for balance!	Activate Your Body<br><br>Moving your body repairs mild drops in mood. Start easy and slow. But go!<br><br>MOVE…<br><br>• Stretch your body<br>• Lift some weights<br>• Go to a yoga class<br>• Go for a short walk<br><br>Start with just 5 or 15 minutes. Something manageable.<br>Gradually increase the time you move each hour or each day.<br><br>NO JUDGMENT!<br>WAKE YOURSELF!	Check out My Charts in the Wellness Plan. Do you see any patterns? What can you learn?<br><br>Get well…

Daily Review Feedback Category 17: Early Warning Signs, Down

Reflect (P9)	Awareness (S2)	Action (S2)	Dial up (P10)	Past success (U10)	A & A (P9)
**1**<br>**RANDOM UNLINKED**	**2**<br>**STATIC**	**3**<br>**STATIC**	**4**<br>**RANDOM UNLINKED**	**5**<br>**RANDOM UNLINKED**	**6**<br>**RANDOM UNLINKED**
Sorry to hear you're down.<br><br>Take some time to check in with yourself and make a plan of action.<br><br>Continue for ideas…	AWARENESS<br><br>Check it out. How many of the following signs are you experiencing?<br><br>△ Low motivation/can't get started<br>△ Low in energy<br>△ Feeling tired/listless<br>△ Loss of interest in activities<br>△ Difficulty concentrating<br>△ Want to be alone<br>△ Less talkative<br>△ Negative thoughts pop into my mind<br>△ Loss of interest in people<br>△ Ideas slowed down<br>△ Less interest in sex<br>△ Cannot get off to sleep<br>△ Interrupted sleep<br><br>If you are experiencing one or more of these early warning signs of depression, consider taking action.	ACTION<br><br>Things you can do:<br><br>✓ Call your psychiatrist if they do not already know about the changes in how you are doing.<br><br>✓ Let your supports know that you are having early warning signs of depression.<br><br>✓ Use dial up skills. Start moving, warm up, get more active, stay involved.<br><br>✓ Keep taking your medications and make sure you are getting the right amount of sleep.	MORE ON DIALING UP<br><br>Depression involves the UNDER-ACTIVATION of your system. Your thoughts, behaviors, feelings, and body are all stimulated.<br><br>Use Dial Up down skills to counteract this process. Speed up, get moving, generate some energy.<br><br>Aim for a moderation.	Do It Yourself<br><br>What have you done in the past to get yourself moving? When you were stressed, or tired, or depressed? What works for you?<br><br>Although there are standard Dial Up skills like Graded Mastery and Graded Pleasure, everyone is different. Some people find exercise invigorating, some people find movies stimulating. Others find dancing or playing the guitar energizing .<br><br>Think back, what works for you? Why not try it now?	Depression by definition is de- motivating. That means it will make you feel helpless and hopeless.<br><br>Try as best you can to ignore these thoughts. They are symptoms. Review your Plan under Awareness & Action in the Wellness Plan.<br><br>Get well...

Daily Review Feedback Category 18: High Risk – Medication Adherence

Page 1
Random Unlinked
5 permutations

Page 2
Random Unlinked
5 unique

Page 3
Blank

Page 4
Blank

Page 5
Blank

Page 6
Random Unlinked
5 permutations

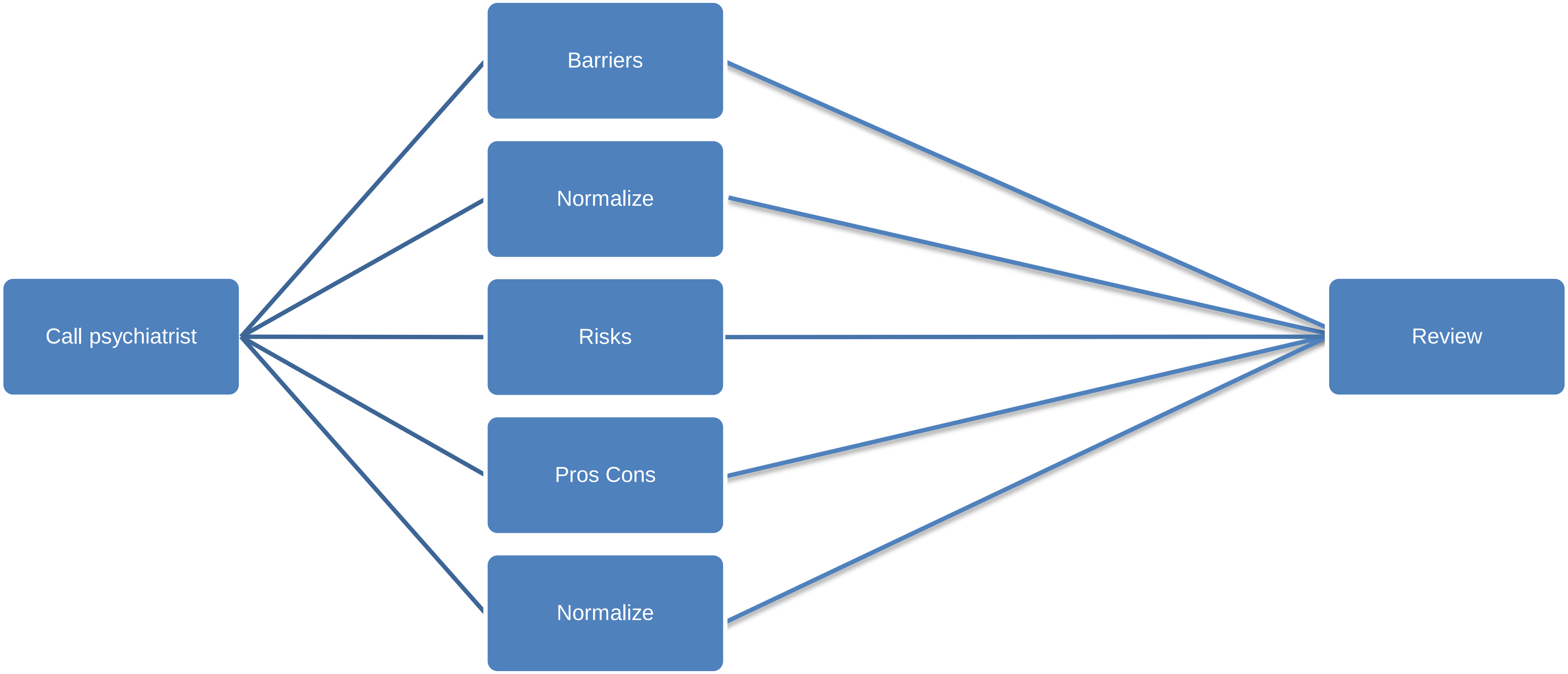

Daily Review Feedback Category 18: High Risk – Medication Adherence

Call (P1)	Barriers (U1)				Reduce Risk (P1)
**1**<br>**RANDOM UNLINKED**	**2**<br>**RANDOM UNLINKED**	**3**<br>**BLANK**	**4**<br>**BLANK**	**5**<br>**BLANK**	**6**<br>**RANDOM UNLINKED**
Glad to see you're doing well. That's great. However, it seems that you are having problems taking your medication consistently.<br><br>Give your psychiatrist a call today. He or she can help you get back on track.	People stop taking their medications for a number of reasons:<br><br>• Thinking they are unnecessary<br>• Side effects<br>• Influence of others<br>• Practical problems<br><br>No matter your reason, talk to your psychiatrist today!				Double check your medication plan in Reduce Risk in the Wellness Plan below.<br><br>Stay well!

Daily Review Feedback Category 18: High Risk – Medication Adherence

Call (P2) | Normalize (U2) | My Charts (P2)

1<br>RANDOM UNLINKED	2<br>RANDOM UNLINKED	3<br>BLANK	4<br>BLANK	5<br>BLANK	6<br>RANDOM UNLINKED
Glad you're doing well.<br><br>Nonetheless, think about calling your psychiatrist. It appears that you are having problems following your medication plan.	Sticking with medications is hard! It may not be pleasant on many levels. In fact, most people don't want to take medications.<br><br>No matter if it is antibiotics, blood pressure medication, or mood stabilizers…people take their medications only about 50% of the time.<br><br>Give your psychiatrist a call today and discuss your concerns.				Check out My Charts in the Wellness Plan. See if you notice anything changing.<br><br>Stay well…

Daily Review Feedback Category 18: High Risk – Medication Adherence

Call (P3)	Risks (U3)				Reduce Risk (P3)
**1**<br>**RANDOM UNLINKED**	**2**<br>**RANDOM UNLINKED**	**3**<br>**BLANK**	**4**<br>**BLANK**	**5**<br>**BLANK**	**6**<br>**RANDOM UNLINKED**
Sounds like you're balanced today, which is great.<br><br>However, you may want to consider what's going on with your medications. It seems that you have missed a number of doses over the past few days.<br><br>Now is a good time to check in with your psychiatrist.	Remember that medications reduce the chances of recurrence by about 50%. Using skills on top of that can further reduce your chances of symptoms.<br><br>Think about the last time you went off medications. What happened? Do you want to risk this again?<br><br>Give your psychiatrist a call today and discuss your concerns.				Take a look at your plan for reducing risk in the Wellness Plan.<br><br>Stay well...

Daily Review Feedback Category 18: High Risk – Medication Adherence

Call (P4)	Pros Cons (U4)				A & A (P4)
**1**<br>**RANDOM UNLINKED**	**2**<br>**RANDOM UNLINKED**	**3**<br>**BLANK**	**4**<br>**BLANK**	**5**<br>**BLANK**	**6**<br>**RANDOM UNLINKED**
Glad to see you're doing well.<br><br>You may, however, want to review your medication plan. It appears that you have missed several doses over the past few days.<br><br>Not taking medications as prescribed puts you at serious risk for relapse. Call your psychiatrist today and let them know what's going on.	Keep your eye on the prize!<br><br>What do you gain by taking medications? What do you lose?<br><br>Is there a way to stay on medications that maximizes the benefits and minimizes the costs?<br><br>Call your psychiatrist today and discuss your medications.				Double check your anchors for Mild Up and Mild Down in the Wellness Plan. Make sure you are balanced.<br><br>Stay well...

Daily Review Feedback Category 18: High Risk – Medication Adherence

Call (P5)	Normalize (U5)				Team (P5)
**1**<br>**RANDOM UNLINKED**	**2**<br>**RANDOM UNLINKED**	**3**<br>**BLANK**	**4**<br>**BLANK**	**5**<br>**BLANK**	**6**<br>**RANDOM UNLINKED**
Looks like you're balanced today, which is great.<br>However, it also seems like things have gotten off track with your medications.<br><br>Give your psychiatrist a call today to discuss any concerns you may have.	Most people find it hard to stick with a medication plan over the long haul.<br><br>The key is to get back on track as quickly as possible.<br><br>Call your psychiatrist today and problem solve together!				If you're thinking you don't need to call or don't want to call your psychiatrist, at least check in with your supports. See what they have to say.<br><br>Review your team in the Wellness Plan.<br><br>Stay well…

Daily Review Feedback Category 19: High Risk – Sleeping Too Little

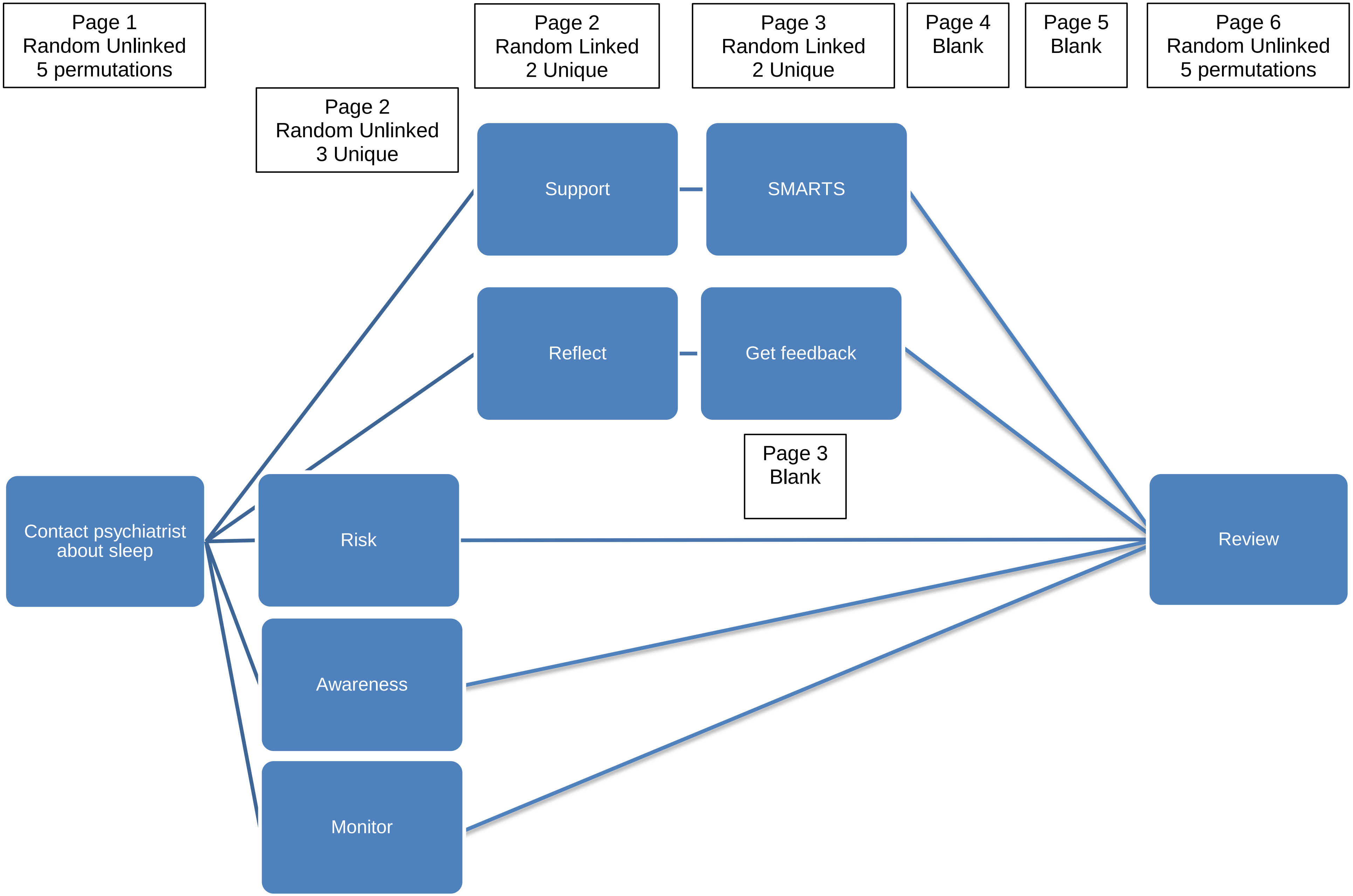

Daily Review Feedback Category 19: High Risk – Sleeping Too Little

Call (P1)	Support (U1)	SMARTS (U1)			Reduce Risk (P1)
**1**<br>**RANDOM UNLINKED**	**2**<br>**RANDOM UNLINKED**	**3**<br>**RANDOM UNLINKED**	**4**<br>**BLANK**	**5**<br>**BLANK**	**6**<br>**RANDOM UNLINKED**
Good to see that you're feeling balanced. It appears, however, that you have been sleeping significantly less than usual lately.<br><br>Now is a good time to check in with your psychiatrist.	Your sleep is really off right now. That can happen for a number of reasons. Don't fret.<br><br>But do call your psychiatrist today for recommendations.	Remember the key to reducing risk of relapse?<br><br>It is to maintain a healthy lifestyle. Be SMARTS:<br><br>• Get adequate **s**leep<br>• Take your **m**edicine<br>• **A**ttend to diet, exercise, and substance use<br>• Keep a good daily **r**outine<br>• Stay **t**ranquil<br>• Maintain a positive **s**ocial life			Remember that by living a healthy lifestyle you can reduce your risk of symptoms.<br><br>Check out Reduce Risk in the Wellness Plan.<br>Stay well...

Daily Review Feedback Category 19: High Risk – Sleeping Too Little

Call (P2) | Reflect (U2) | Feedback (U2) | My Skills (P2)

1<br>RANDOM UNLINKED	2<br>RANDOM UNLINKED	3<br>RANDOM UNLINKED	4<br>BLANK	5<br>BLANK	6<br>RANDOM UNLINKED
Looks like you're wellness ratings are good.<br><br>You're sleep, however, seems to have gotten off track. Sleeping too little puts you at risk for symptoms and it can even trigger mood episodes.<br><br>Check in with your psychiatrist today.	Do you know what has gotten in the way of getting enough sleep? Do you have things going on? Can you just not seem to fall or stay asleep?<br><br>Remember that serious disruptions in sleep often require medical intervention.<br><br>Give your psychiatrist a call. Your medications may need an adjustment, even if it is temporary.	If you are reluctant to call your psychiatrist, talk to your supports.<br><br>See what they have to say.<br><br>Let them know about your sleep and the LiveWell recommendations.			Check out My Skills in in the Wellness Plan for sleep skills.<br><br>If you haven't saved any sleep skills, you can look in the Toolbox.<br><br>Stay well…

Daily Review Feedback Category 19: High Risk – Sleeping Too Little

Call (P3) | Risk (U3) | Reduce Risk (P3)

1<br>RANDOM UNLINKED	2<br>RANDOM UNLINKED	3<br>BLANK	4<br>BLANK	5<br>BLANK	6<br>RANDOM UNLINKED
Glad to see you're doing well. That's great. However, It seems as though you have been getting less sleep than you need for quite some time now.<br><br>Contact your psychiatrist and let them know what's going on.	Don't forget that inadequate sleep is one of the most potent triggers for episodes of mania. It is also an early warning sign of mania.<br><br>Call your psychiatrist today.<br><br>You may need to make some adjustments to your medications in order to prevent a symptom recurrence.				Double check your sleep plan in Reduce Risk in the Wellness Plan below.<br><br>Stay well!

Daily Review Feedback Category 19: High Risk – Sleeping Too Little

Call (P4) | Awareness (U4) | My Charts (P4)

1<br>**RANDOM UNLINKED**	2<br>**RANDOM UNLINKED**	3<br>**BLANK**	4<br>**BLANK**	5<br>**BLANK**	6<br>**RANDOM UNLINKED**
It seems you're doing well, which is great. It also appears, however, that you have not been getting enough rest.<br><br>This is serious enough that you should check in with your psychiatrist today.	Are you feeling tired?<br><br>Double check your anchors for feeling up and feeling down. Sleep disruptions can be a symptom. Make sure you're not entering or having a mood episode.<br><br>Your sleep is off enough that you really should give your psychiatrist a call, even if you are not having other symptoms.				Check out My Charts in the Wellness Plan. See if there are any connections between your sleep and symptoms.<br><br>Stay well...

Daily Review Feedback Category 19: High Risk – Sleeping Too Little

Call (P5)	Monitor (U5)				A & A (P5)
**1<br>RANDOM UNLINKED**	**2<br>RANDOM UNLINKED**	**3<br>BLANK**	**4<br>BLANK**	**5<br>BLANK**	**6<br>RANDOM UNLINKED**
You're reporting doing well, which is good.<br><br>However, the little sleep you have been getting is increasing your risk of developing symptoms.<br><br>Give your psychiatrist a call today and see what you can do about your sleep.	Disruptions in sleep happen all the time, for different reasons. It's good you're keeping track of your sleep.<br><br>Give your psychiatrist a call today.<br><br>Err on the side of caution. See what they think about your sleep and what you might do to get back on track.				Double check your anchors for Mild Up in the Wellness Plan. Make sure you are not having any symptoms.<br><br>Stay well…

Daily Review Feedback Category 20: High Risk – Sleeping Too Much

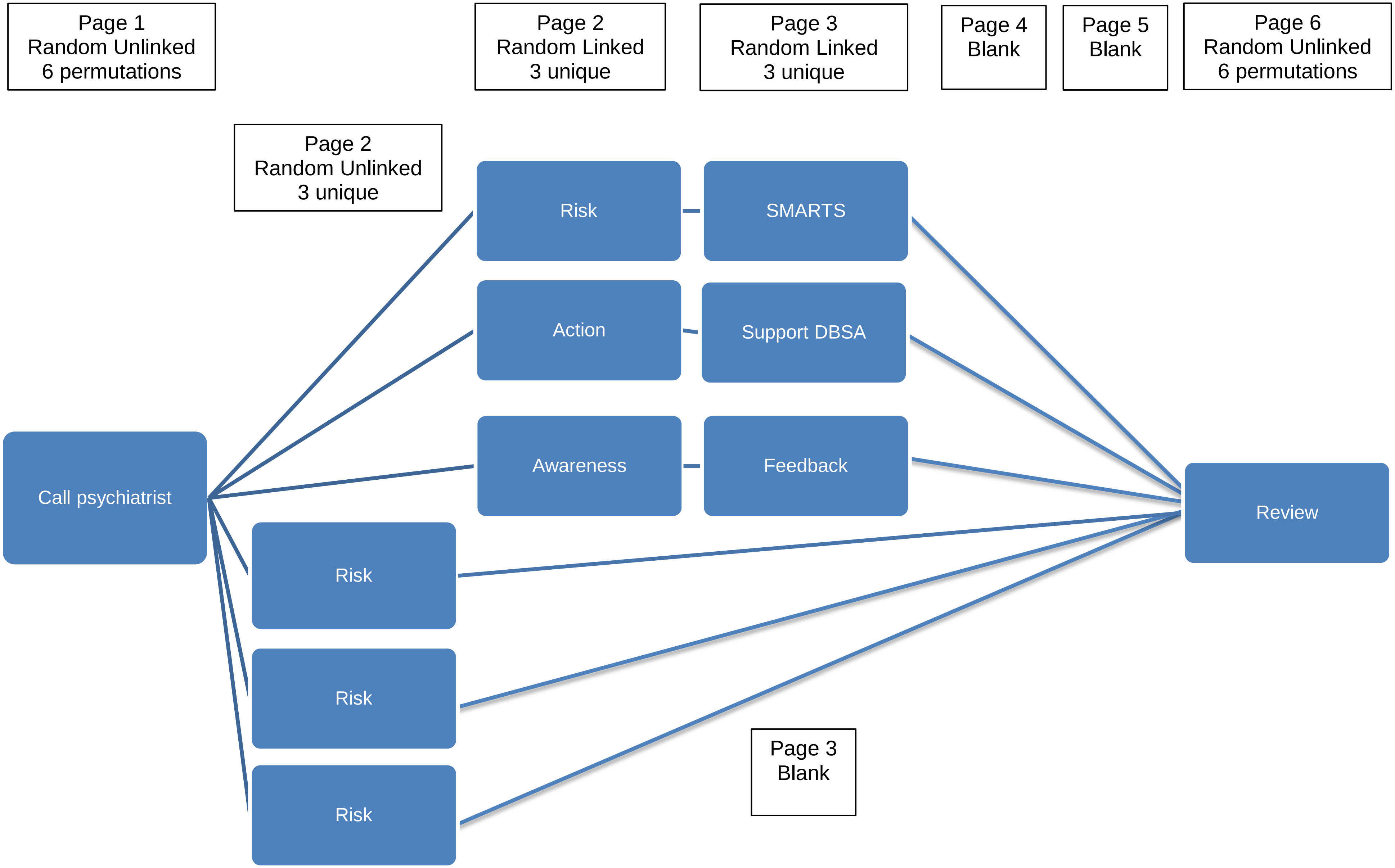

Daily Review Feedback Category 20: High Risk – Sleeping Too Much

Call (P1)	Risk (U1)	SMARTS (U1)			My Skills (P1)
**1<br>RANDOM UNLINKED**	**2<br>RANDOM LINKED**	**3<br>RANDOM LINKED**	**4<br>BLANK**	**5<br>BLANK**	**6<br>RANDOM UNLINKED**
Glad you're feeling well. That's good. However, it seems as though you have been getting a lot of sleep lately. This can be a sign of trouble.<br><br>Check in with your psychiatrist today.	Your sleep is really off right now. While this can happen for a number of reasons, it may be putting you at risk for an episode.<br><br>Don't fret. But do call your psychiatrist today for recommendations.	Remember the key to reducing risk of relapse?<br><br>It is to maintain a healthy lifestyle. Be SMARTS:<br><br>• Get adequate **s**leep<br>• Take your **m**edicine<br>• **A**ttend to diet, exercise, and substance use<br>• Keep a good daily **r**outine<br>• Stay **t**ranquil<br>Maintain a positive **s**ocial life			Check out My Skills in the Wellness Plan for sleep skills.<br><br>If you haven't saved any sleep skills, why not start now?<br><br>Be well!

Daily Review Feedback Category 20: High Risk – Sleeping Too Much

Call (P2) | Action (U2) | Support (U2) | My Charts (P2)

1<br>**RANDOM UNLINKED**	2<br>**RANDOM LINKED**	3<br>**RANDOM LINKED**	4<br>**BLANK**	5<br>**BLANK**	6<br>**RANDOM UNLINKED**
It seems that you're doing well, which is great. However, your sleep seems off. Sleeping too much puts you at risk for symptoms and it can even trigger mood episodes. Give your psychiatrist a call today.	Problems with sleep can put you at risk for symptoms, or you may even be experiencing symptoms and are not aware of it.<br><br>Don't worry, take action. Getting your sleep back on track is the top priority right now.<br><br>Check in with your psychiatrist today.	If you are having concerns about talking to your psychiatrist, try taking a look at the DBSA website for support:<br><br>www.dbsalliance.com			Check out My Charts in the Wellness Plan. Do you see any patterns between your sleep and wellness ratings?<br><br>Stay well!

Daily Review Feedback Category 20: High Risk – Sleeping Too Much

Call (P3)	Awareness (U3)	Feedback (U3)			Reduce Risk (P3)
**1**<br>**RANDOM UNLINKED**	**2**<br>**RANDOM LINKED**	**3**<br>**RANDOM LINKED**	**4**<br>**BLANK**	**5**<br>**BLANK**	**6**<br>**RANDOM UNLINKED**
Good to see that you're balanced. However, it looks like you have been sleeping too much lately.<br><br>Now is a good time to reach out to your psychiatrist.	Are you noticing any early warning signs of depression? Are you noticing any symptoms of depression? Go through your check list.<br><br>Sleeping too much for too long can be a sign of depression. It also can trigger depression and even mania.<br><br>Call your psychiatrist.<br>They should know what is going on with your sleep.	If you are reluctant to call your psychiatrist, reach out to your supports.<br><br>Let them know that your sleep has been off lately and what the LiveWell program recommends.<br><br>They may have ideas or suggestions to help get your sleep back on track!			Double check your sleep plan in Reduce Risk in the Wellness Plan below.<br><br>Stay well!

Daily Review Feedback Category 20: High Risk – Sleeping Too Much

Call (P4)	Risk (U4)				Reduce Risk (P4)
**1**<br>**RANDOM UNLINKED**	**2**<br>**RANDOM UNLINKED**	**3**<br>**BLANK**	**4**<br>**BLANK**	**5**<br>**BLANK**	**6**<br>**RANDOM UNLINKED**
Glad to see you're doing well. That's great. However, It seems as though you have been getting more sleep than you need for quite some time now.<br><br>Call your psychiatrist today and let them know what's going on.	Don't forget that disruptions in sleep schedules—sleeping too much, sleeping too little, and having an erratic sleep schedule—can be a potent trigger for episodes of depression and mania.<br><br>Disturbed sleep is also an early warning sign of mood episodes. So you may want to double---check your wellness rating. Is it possible you are depressed? Maybe a ---2 or<br>---3?<br><br>Call your psychiatrist today. You may need to make some adjustments to your medications in order to prevent a symptom recurrence.				Remember that by living a healthy lifestyle you can reduce your risk of symptoms.<br><br>Check out Reduce Risk in the Wellness Plan to remind yourself of the commitments you made to staying well.

Daily Review Feedback Category 20: High Risk – Sleeping Too Much

Call (P5)	Risk (U5)				My Charts (P5)
**1 RANDOM UNLINKED**	**2 RANDOM UNLINKED**	**3 BLANK**	**4 BLANK**	**5 BLANK**	**6 RANDOM UNLINKED**
Good to see that you're rating yourself as balanced. It appears, however, that you have been sleeping significantly more than usual lately.<br><br>Now is a good time to check in with your psychiatrist.	It happens to everyone at one point or another -- too much sleep, too little sleep, or erratic sleep. It is hard to be consistent.<br><br>Changes to your sleep schedule can occur for many different reasons. However, getting too much sleep for too long is not good for your health.<br><br>Give your psychiatrist a call today.				Check out My Charts in the Wellness Plan. See if your sleep is related to your wellness ratings.<br><br>Stay well…

Daily Review Feedback Category 20: High Risk – Sleeping Too Much

Call (P6)

Risk (U6)

A & A (P6)

1<br>RANDOM UNLINKED	2<br>RANDOM UNLINKED	3<br>BLANK	4<br>BLANK	5<br>BLANK	6<br>RANDOM UNLINKED
It's good that you're doing well. However, it seems as though you have been getting too much sleep lately.<br>Oversleeping can be an early warning sign of problems.<br><br>Take action now. Give your psychiatrist a call today for help getting your sleep back on track.	Sleeping in can feel so good. Maybe you were sleep deprived and are catching up on lost sleep. Or maybe you've had a lot on your plate recently and are just feeling exhausted.<br><br>No matter what the reason is, be careful. Dramatic changes in sleep can trigger symptoms.<br><br>Check in with your psychiatrist. You may need to make some adjustments to your medications.				Double check your anchors for Mild, Moderate, and Severe Down in Awareness & Action.<br><br>Look in the Wellness Plan to make sure you're not feeling down or depressed.<br><br>Stay well…

Daily Review Feedback Category 21: Moderate Risk – Medication Adherence, Necessary-Bipolar (Choice 1.1)

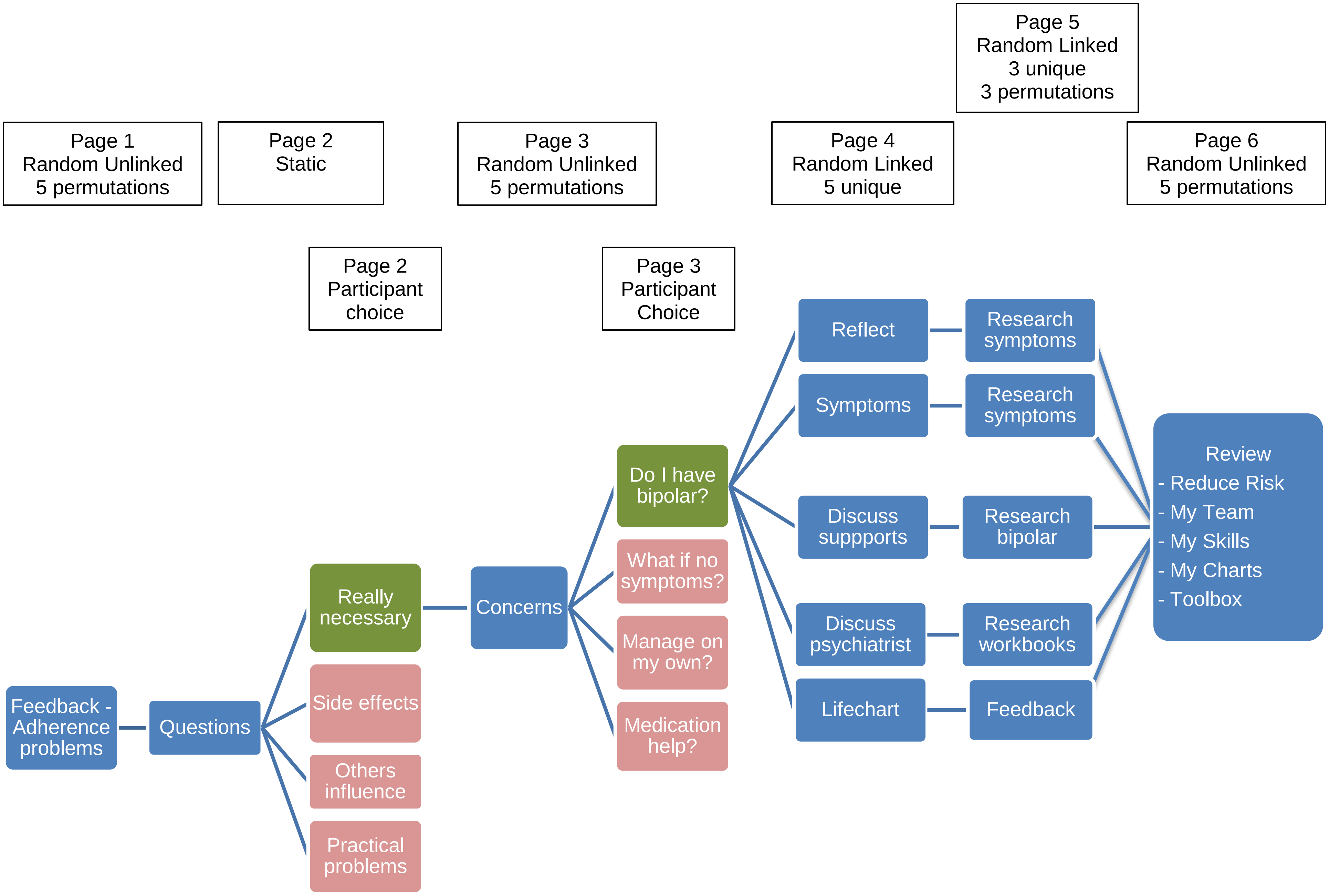

Daily Review Feedback Category 21: Moderate Risk – Medication Adherence, Necessary-Bipolar (Choice 1.1)

Reflect (P1)	Questions (S1)	Concerns (P1)	Reflect (U1)	Symptoms (U1)	My Skills (P1)
**1<br>RANDOM UNLINKED**	**2<br>RANDOM UNLINKED**	**3<br>RANDOM UNLINKED**	**4<br>RANDOM LINKED**	**5<br>RANDOM LINKED**	**6<br>RANDOM UNLINKED**
You're not reporting any symptoms, which is great.<br><br>However, you may want to take some time now to review your medication schedule. It seems that you are having problems taking your medications consistently.<br><br>Continue to learn more…	A lot of things impact people's decision to take medications. It's complicated.<br><br>Below are common questions people have about medications.<br><br>When it comes to medications, do you wonder about…	Taking medications is a big decision. Most people have reservations. You're not alone.<br><br>Read about…	CHECK IT OUT<br><br>Talk with your psychiatrist and supports about your thoughts and feelings about your symptoms.<br><br>Review your history. Have you had experiences consistent with mania? How about depression?<br><br>Have you been in the hospital because of things you said or did? Or how you felt? Are you concerned about ending up in the hospital again?<br><br>There is no need for shame or denial. Bipolar disorder is a medical condition. It can be managed.	DO SOME RESEARCH<br><br>There are many good places to read more about bipolar disorder. The more you know, the more able you will be to make informed decisions.<br><br>You can learn more about the symptoms of bipolar disorder here:<br><br>• Depression & Bipolar Support Alliance<br>• National Alliance for Mental Illness<br>• National Institute of Mental Health	Check out any medication skills you added to My Resources in the Wellness Plan.<br><br>Be well!

Daily Review Feedback Category 21: Moderate Risk – Medication Adherence, Necessary-Bipolar (Choice 1.1)

Reflect (P2)	Questions (S1)	Concerns (P2)	Symptoms (U2)	Symptoms (U2)	My Charts (P2)
**1**<br>**RANDOM UNLINKED**	**2**<br>**RANDOM UNLINKED**	**3**<br>**RANDOM UNLINKED**	**4**<br>**RANDOM LINKED**	**5**<br>**RANDOM LINKED**	**6**<br>**RANDOM UNLINKED**
Seems like you're doing well. That's great!<br><br>To keep it going, you may want to reconsider your medication schedule. It looks like you've missed a couple doses over the past few days.<br><br>Remember that taking medications as planned will help you stay well.<br><br>Continue to read more…	A lot of things impact people's decisions to take medications. It's complicated.<br><br>Below are common questions people have about medications.<br><br>When it comes to medications, do you wonder about…	It is important that any questions or concerns you have about taking medications are adequately addressed. It is your body and your health!<br><br>Read about…	CHECK IT OUT<br><br>Have you ever had…<br><br>△ A low mood or reduced interest and pleasure in life<br>△ Weight loss or weight gain<br>△ Slept too much or too little<br>△ Physical agitation or slowing down<br>△ Fatigue or loss of energy<br>△ Feeling worthless or guilty<br>△ Difficulty concentrating<br>△ Thoughts of death or suicide Have you ever<br><br>had…<br><br>△ A sense of being overly happy and excited, or maybe irritable<br>△ Increased esteem/feeling superior<br>△ Decreased need for sleep<br>△ More talkative than usual<br>△ Racing thoughts<br>△ Difficulties concentrating<br>△ Increased activity level<br>△ Risky activities<br><br>The lists above are the symptoms of depression and mania, respectively.	DO SOME RESEARCH<br><br>Educate yourself and know the symptoms of bipolar disorder (if you don't already). You can check out the lessons here or search online for more information.<br><br>Some good resources are:<br><br>• The Depression and Bipolar Disorder Support Alliance<br>• National Institute of Mental Health<br>• National Alliance on Mental Illness	Check out the relationship between medications and wellness in My Charts in the Wellness Plan.<br><br>Stay well!

Daily Review Feedback Category 21: Moderate Risk – Medication Adherence, Necessary-Bipolar (Choice 1.1)

Reflect (P3)	Questions (S1)	Concerns (P3)	Discuss (U3)	Bipolar (U3)	Toolbox (P3)
**1**<br>**RANDOM UNLINKED**	**2**<br>**RANDOM UNLINKED**	**3**<br>**RANDOM UNLINKED**	**4**<br>**RANDOM LINKED**	**5**<br>**RANDOM LINKED**	**6**<br>**RANDOM UNLINKED**
It appears you're doing well. That's good to hear.<br><br>However, you may want to review your medication schedule. It seems that you've been having problems taking your medications consistently for the past few days.<br><br>There are many reasons why this may be happening.<br><br>Press continue to learn more about common barriers to taking medications.	A lot of things impact people's decisions to take medications. It's complicated.<br><br>Below are common questions people have about medications.<br><br>When it comes to medications, do you wonder about.	There are many factors that can affect your decision to take or not to take medications.<br><br>Read about…	CHECK IT OUT<br><br>Sit down with one of your supports. Look at the list of symptoms of depression and the symptoms of mania in Foundations.<br><br>Have a discussion about your experiences in the past with any or all of these symptoms. Get their feedback as well.	DO SOME RESEARCH<br><br>Read more about bipolar disorder. A good place to start is the website developed by the Depression and Bipolar Disorder Support Alliance.<br><br>Bipolar disorder affects roughly every 1 to 2 people out of 100. So, it is relatively common. It is also manageable!	Learn new skills and strategies for taking medications consistently in the Lifestyle section of Toolbox.<br><br>Stay well!

Daily Review Feedback Category 21: Moderate Risk – Medication Adherence, Necessary-Bipolar (Choice 1.1)

Risk (P4)	Questions (S1)	Concerns (P4)	Discuss (U4)	Workbooks (U4)	My Team (P4)
**1**<br>**RANDOM UNLINKED**	**2**<br>**RANDOM UNLINKED**	**3**<br>**RANDOM UNLINKED**	**4**<br>**RANDOM LINKED**	**5**<br>**RANDOM LINKED**	**6**<br>**RANDOM UNLINKED**
It's great to see that you're doing well. However, it looks like you've been having trouble staying on top of your medication schedule the past couple of days.<br><br>Remember, missed doses and not following a regular schedule can put you at risk for symptoms.<br><br>Continue to learn more….	A lot of things impact people's decisions to take medications. It's complicated.<br><br>Below are common questions people have about medications.<br><br>When it comes to medications, do you wonder about.	The decision to take medications is not easy, especially when it comes to taking them for many years.<br><br>Read about…	CHECK IT OUT<br><br>Talk with your psychiatrist again. Ask them why they think you have bipolar disorder. Express your reservations or concerns.<br><br>An open and honest dialogue is important. Listen to what your psychiatrist has to say. Share your own thoughts and feelings as well.	DO SOME RESEARCH<br><br>Consider purchasing one or more of the bipolar workbooks on the market. Read and get informed.<br><br>*The Bipolar Workbook: Tools for Controlling Your Mood Swings. (Basco, 2006)*<br><br>*Managing Bipolar Disorder: A Cognitive---Behavioral Treatment Program Workbook. (Otto, 2008)*<br><br>*The Bipolar Disorder Survival Guide: What You and Your Family Need to Know. (Miklowitz, 2002).*	Consider sharing your thoughts and feeling about medications with your supports. Having an open dialogue with people who care about you can help you stay on track. You can review your team in the Wellness Plan.<br><br>Stay well!

Daily Review Feedback Category 21: Moderate Risk – Medication Adherence, Necessary-Bipolar (Choice 1.1)

Risk (P5)	Questions (S1)	Concerns (P5)	Lifechart (U5)	Feedback (U5)	Reduce Risk (P5)

**1**<br>**RANDOM UNLINKED**	**2**<br>**RANDOM UNLINKED**	**3**<br>**RANDOM UNLINKED**	**4**<br>**RANDOM LINKED**	**5**<br>**RANDOM LINKED**	**6**<br>**RANDOM UNLINKED**
You're reporting doing well today. It's great to hear that. It seems, however, that you may be having problems taking your medicines as prescribed.<br><br>Remember, taking medications regularly is one of the most important things you can do to stay well.<br><br>Continue to read more…	A lot of things impact people's decisions to take medications. It's complicated.<br><br>Below are common questions people have about medications.<br><br>When it comes to medications, do you wonder about…	Taking medications should make sense to you. Any questions or problems with your medication plan should be addressed.<br><br>Read about…	CHECK IT OUT<br><br>Chart out your lifetime symptoms. Take a piece of paper and turn it on its side. Draw a line across the middle of a piece of paper. At the left end write "onset" (which means first symptoms). At the right end, write "now."<br><br>What happened at the onset? Was it depression? (It often is.) How have your symptoms changed or evolved since they first started?<br><br>The horizontal line across the middle of the chart represents being balanced (neither depressed nor manic). Create a graphical picture of mood fluctuations over time by drawing above the line for mania and below the line for depression. For each episode record your:<br><br>1. Age<br>2. Use of alcohol and street drugs<br>3. Major life events<br>4. Treatments<br><br>Search the internet for Bipolar Life Charts for examples of how to complete this task.<br><br>Examine your chart. Is there a connection between:<br><br>• Substances and episodes?<br>• Life events and episodes?<br>• Medicine and episodes?	DO SOME RESEARCH<br><br>Show your psychiatrist and your supports your Life Chart.<br><br>Discuss your ideas and ask if they have anything to add. Often others notice things that you do not notice yourself.	Review your medication plan in Reduce Risk in the Wellness Plan.<br><br>Stay well!

Daily Review Feedback Category 21: Moderate Risk – Medication Adherence, Necessary- No Symptoms (Choice 1.2)

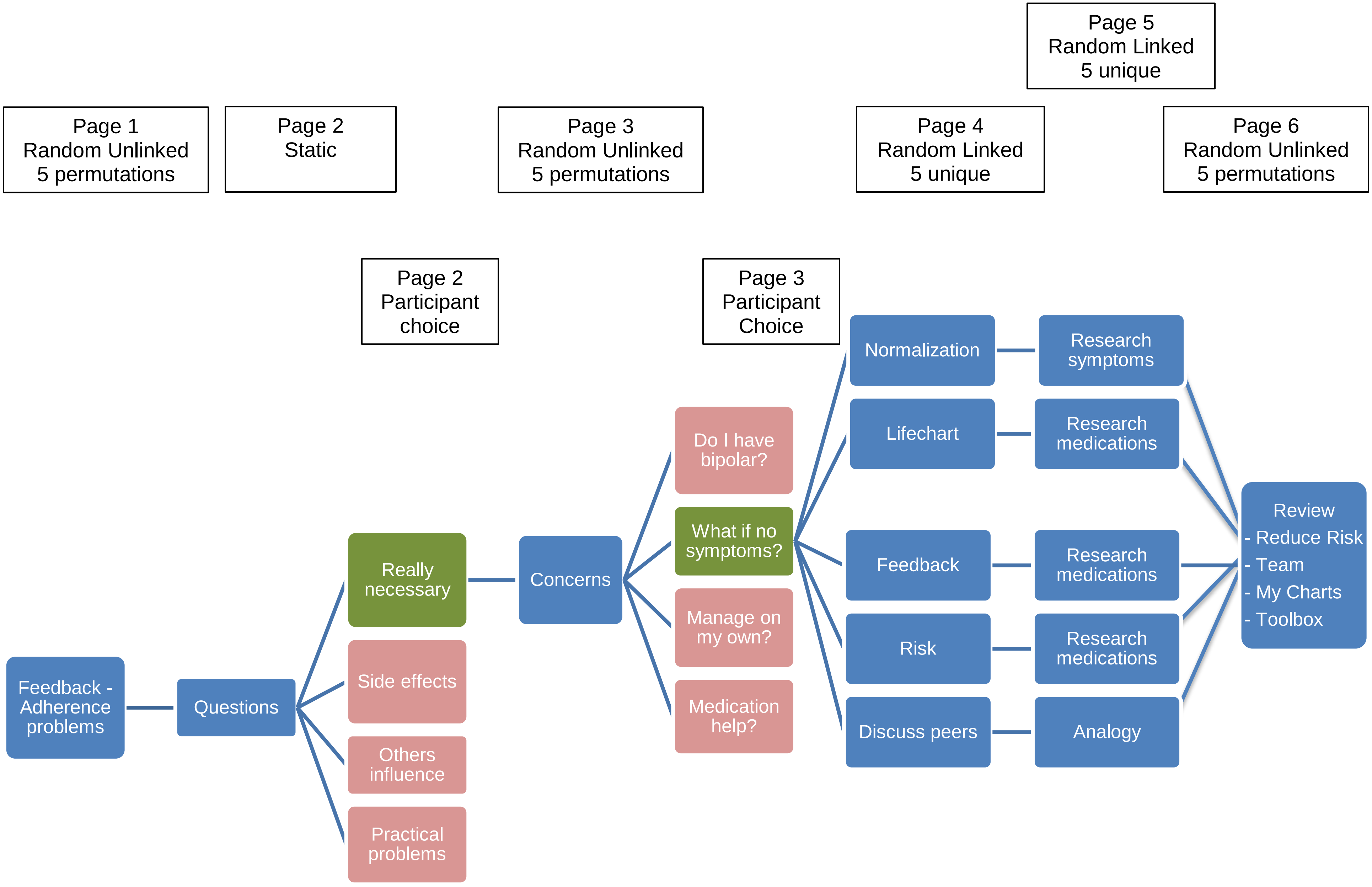

Daily Review Feedback Category 21: Moderate Risk – Medication Adherence, Necessary-No Symptoms (Choice 1.2)

Reflect (P6)	Questions (S1)	Concerns (P1)	Normalize (U6)	Symptoms (U1)	(P6)
**1**<br>**RANDOM UNLINKED**	**2**<br>**RANDOM UNLINKED**	**3**<br>**RANDOM UNLINKED**	**4**<br>**RANDOM LINKED**	**5**<br>**RANDOM LINKED**	**6**<br>**RANDOM UNLINKED**
Glad to see you're well. You may want to take a look at your medication schedule. It seems you may not be keeping up as planned.<br><br>Continue to learn about medications.	A lot of things impact people's decisions to take medications. It's complicated.<br><br>Below are common questions people have about medications.<br><br>When it comes to medications, do you wonder about…	Taking medications is a big decision. Most people have reservations! You're not alone.<br><br>Read about…	CHECK IT OUT<br><br>People often stop taking their medications when they start to feel better. This includes all sorts of medications, such as antibiotics and high blood pressure medicine.<br><br>It is a problem in logic. People assume they are better DESPITE medications, rather than BECAUSE of medications.<br><br>Have you stopped taking your medications in the past? What happened?<br><br>Did you know that going off medications makes it hard to treat bipolar disorder?	DO SOME RESEARCH<br><br>There are many good places to read more about bipolar disorder. The more you know, the more able you will be to make informed decisions.<br><br>Read about the symptoms of bipolar disorder.<br><br>• Depression & Bipolar Support Alliance<br>• National Alliance for Mental Illness<br>• National Institute of Mental Health	Be well!

Daily Review Feedback Category 21: Moderate Risk – Medication Adherence, Necessary-No Symptoms (Choice 1.2)

Reflect (P7)	Questions (S1)	Concerns (P6)	Lifechart (U7)	Medications (U6)	Reduce Risk (P7)
**1<br>RANDOM UNLINKED**	**2<br>RANDOM UNLINKED**	**3<br>RANDOM UNLINKED**	**4<br>RANDOM LINKED**	**5<br>RANDOM LINKED**	**6<br>RANDOM UNLINKED**
Good to see you're doing well.<br><br>You may want to take a look at your medication schedule. Taking medications regularly is one of the most important things you can do to stay well.<br><br>Continue to read more…	A lot of things impact people's decisions to take medications. It's complicated.<br><br>Below are common questions people have about medications.<br><br>When it comes to medications, do you wonder about…	Medications should make sense for you. Any questions or problems need to be addressed!<br><br>Read about…	CHECK IT OUT<br><br>Complete a life chart. (There are instructions on how to do this in the Self---Assessment section of Skills.)<br><br>Essentially, this involves creating a graph of mood episodes during the course of your lifetime.<br><br>Many people have symptoms within weeks or months of discontinuing medications. Is this a pattern for you?	DO SOME RESEARCH<br><br>Taking medications, even when you have no symptoms, decreases the likelihood of getting symptoms.<br><br>Read more for yourself on this topic. Get informed.	Check out your medication plan in Reduce Risk in the Wellness Plan.<br><br>Stay well!

Daily Review Feedback Category 21: Moderate Risk – Medication Adherence, Necessary-No Symptoms (Choice 1.2)

Reflect (P8)	Questions (S1)	Concerns (P7)	Feedback (U8)	Medications (U7)	My Charts (P3)
**1**<br>**RANDOM UNLINKED**	**2**<br>**RANDOM UNLINKED**	**3**<br>**RANDOM UNLINKED**	**4**<br>**RANDOM LINKED**	**5**<br>**RANDOM LINKED**	**6**<br>**RANDOM UNLINKED**
Nice thing to be well.<br><br>Consider your medication schedule. It appears that things are a bit irregular right now.<br><br>Continue on...	A lot of things impact people's decisions to take medications. It's complicated.<br><br>Below are common questions people have about medications.<br><br>When it comes to medications, do you wonder about…	It is important to get all of your questions and concerns about medications answered. It is your body and your health!<br><br>Read about…	CHECK IT OUT<br><br>Talk to your psychiatrist and supports. Ask them what they have noticed in the past when you take your medications and when you don't take your medications.<br><br>Try not to be defensive. No one really wants to have to take medications, but listen to what they have to say.	DO SOME RESEARCH<br><br>What do you know about mood stabilizers? Antidepressants? Antipsychotics?<br><br>Read more about the variety of medications used to treat bipolar disorder. Get informed.	Check out the relationship between taking medications and your wellness in My Charts in the Wellness Plan.<br><br>Stay well!

Daily Review Feedback Category 21: Moderate Risk – Medication Adherence, Necessary-No Symptoms (Choice 1.2)

Reflect (P9)	Questions (S1)	Concerns (P8)	Risk (U9)	Medications (U8)	My Team (P8)
**1**<br>**RANDOM UNLINKED**	**2**<br>**RANDOM UNLINKED**	**3**<br>**RANDOM UNLINKED**	**4**<br>**RANDOM LINKED**	**5**<br>**RANDOM LINKED**	**6**<br>**RANDOM UNLINKED**
It seems you are well. Good to see.<br><br>Consider how you're taking your medications. Seems like you may be a bit off your plan.<br><br>There are many reasons things might have gotten off.<br><br>Continue to read more…	A lot of things impact people's decisions to take medications. It's complicated.<br><br>Below are common questions people have about medications.<br><br>When it comes to medications, do you wonder about…	Taking medications is no easy decision, especially when it comes to taking them for years.<br><br>Read about…	CHECK IT OUT<br><br>People often stop taking medications when their symptoms go away. Sometimes they assume they no longer need them. Sometimes they just want a break and feel it is safe.<br><br>Whatever the reason, studies are clear. Taking medications regularly reduces the risk of mood episodes.<br><br>What do you think?	DO SOME RESEARCH<br><br>Look up the statistics for people with bipolar disorder or ask your psychiatrist.<br><br>How many have a mood episode when taking medications?<br><br>How many have mood episodes after discontinuing medications?	Consider talking to your supports. Have an open dialogue about your thoughts and feelings about medications. You can review your team in the Wellness Plan.<br><br>Stay well!

Daily Review Feedback Category 21: Moderate Risk – Medication Adherence, Necessary-No Symptoms (Choice 1.2)

Risk (P10)	Questions (S1)	Concerns (P9)	Discuss (U10)	Analogy (U9)	Toolbox (P9)
It appears you're doing well. That's great.<br><br>Remember that taking medications as planned will help you stay well.<br><br>Learn more about common barriers to taking medications…	A lot of things impact people's decisions to take medications. It's complicated.<br><br>Below are common questions people have about medications.<br><br>When it comes to medications, do you wonder about…	There are so many factors that impact people's decision to take or not take medications.<br><br>Read about…	CHECK IT OUT<br><br>Talk to your peers. Go to a DBSA meeting or join their online chat. See what others think about taking medications when they don't have symptoms.	DO SOME RESEARCH<br><br>Why take medications when you have no symptoms? That's a very good question.<br><br>It's kind of like taking high blood pressure medications. It is preventative.<br><br>Do some reading about this. Get more informed.	Take a look at Beliefs about Medications in Toolbox.<br><br>Stay well!

Daily Review Feedback Category 21: Moderate Risk – Medication Adherence, Necessary- Manage Own (Choice 1.3)

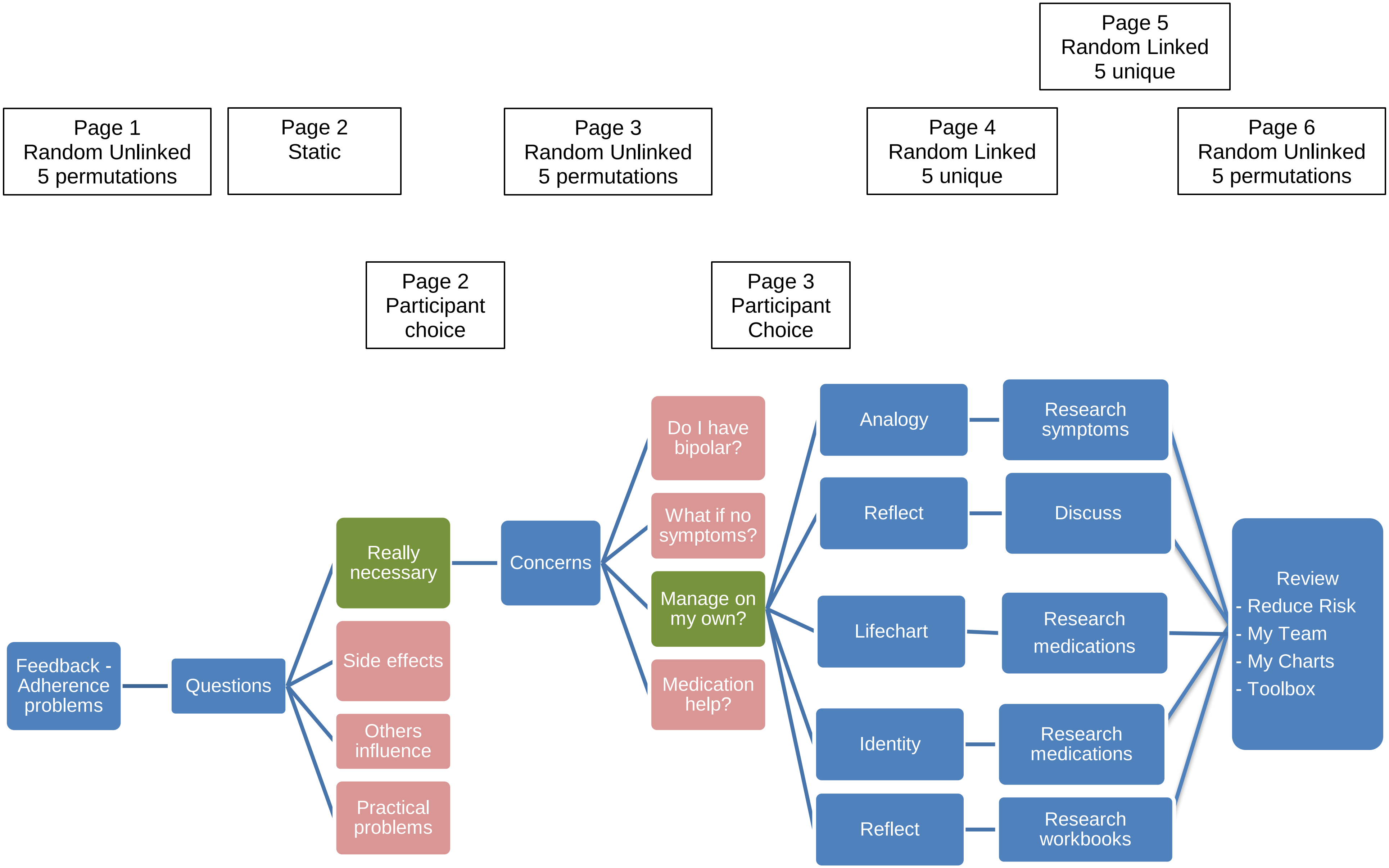

Daily Review Feedback Category 21: Moderate Risk – Medication Adherence, Necessary – Manage Own (Choice 1.3)

Reflect (P6)	Questions (S1)	Concerns (P1)	Analogy (U11)	Research (U1)	(P6)
**1**<br>**RANDOM UNLINKED**	**2**<br>**RANDOM UNLINKED**	**3**<br>**RANDOM UNLINKED**	**4**<br>**RANDOM LINKED**	**5**<br>**RANDOM LINKED**	**6**<br>**RANDOM UNLINKED**
Glad to see you're well. You may want to take a look at your medication schedule. It seems you may not be keeping up as planned.<br><br>Continue to learn about medications.	A lot of things impact people's decisions to take medications. It's complicated.<br><br>Below are common questions people have about medications.<br><br>When it comes to medications, do you wonder about…	Taking medications is a big decision. Most people have reservations! You're not alone.<br><br>Read about…	CONSIDER THIS<br><br>Because the symptoms of bipolar disorder involve your thoughts and behaviors, it is easy to assume you can manage without medications. Everyone likes to think they have control over themselves.<br><br>This is true until the symptoms become moderate or severe. At that point, no one can control the symptoms on their own for a length of time.<br><br>Your biology is just too strong. Managing bipolar disorder on your own is like trying to manage Type I Diabetes on your own without insulin.	DO SOME RESEARCH<br><br>There are many good places to read more about bipolar disorder. The more you know, the more able you will be to make informed decisions.<br><br>Read about the symptoms of bipolar disorder.<br><br>• Depression & Bipolar Support Alliance<br>• National Alliance for Mental Illness<br>• National Institute of Mental Health	Be well!

Daily Review Feedback Category 21: Moderate Risk – Medication Adherence, Necessary – Manage Own (Choice 1.3)

Reflect (P7)	Questions (S1)	Concerns (P6)	Reflect (U12)	Discuss (U10)	Reduce Risk (P7)
**1**<br>**RANDOM UNLINKED**	**2**<br>**RANDOM UNLINKED**	**3**<br>**RANDOM UNLINKED**	**4**<br>**RANDOM LINKED**	**5**<br>**RANDOM LINKED**	**6**<br>**RANDOM UNLINKED**
Good to see you're doing well.<br><br>You may want to take a look at your medication schedule. Taking medications regularly is one of the most important things you can do to stay well.<br><br>Continue to read more…	A lot of things impact people's decisions to take medications. It's complicated.<br><br>Below are common questions people have about medications.<br><br>When it comes to medications, do you wonder about…	Medications should make sense for you. Any questions or problems need to be addressed!<br><br>Read about…	THINK ABOUT IT<br><br>Why is it important to you that you try to manage symptoms on your own?<br><br>Is it your pride? Is it an attempt to think the symptoms are not all that serious? Is it an aversion to putting anything you consider unnatural in your body?<br><br>Think carefully about your opinions here. What do you believe is important?	Talk to your psychiatrist about your concerns. See if there is any way to maintain your pride, accept the idea of having bipolar disorder, work around your aversion to medications, or address any other ideas you have that get in the way of taking your medications.	Check out your medication plan in Reduce Risk in the Wellness Plan.<br><br>Stay well!

Daily Review Feedback Category 21: Moderate Risk – Medication Adherence, Necessary – Manage Own (Choice 1.3)

Reflect (P8)	Questions (S1)	Concerns (P7)	Lifechart (U13)	Medications (U11)	My Charts (P3)
**1<br>RANDOM UNLINKED**	**2<br>RANDOM UNLINKED**	**3<br>RANDOM UNLINKED**	**4<br>RANDOM LINKED**	**5<br>RANDOM LINKED**	**6<br>RANDOM UNLINKED**
Nice thing to be well.<br><br>Consider your medication schedule. It appears that things are a bit irregular right now.<br><br>Continue on...	A lot of things impact people's decisions to take medications. It's complicated.<br><br>Below are common questions people have about medications.<br><br>When it comes to medications, do you wonder about…	It is important to get all of your questions and concerns about medications answered. It is your body and your health!<br><br>Read about…	CHECK IT OUT<br><br>Review your life chart. (There are instructions on how to do this in the Self---Assessment section of Skills.)<br><br>Do you end up symptomatic in the weeks or months after you stop taking medications?<br><br>Trying to manage without medications inevitably fails for people. It's not just you. Unfortunately, there are no coping skills that work alone, that is, without medications.	DO SOME RESEARCH<br><br>Read about treatments for bipolar disorder. Read about people's experiences on and off medications.<br><br>The Depression and Bipolar Support Alliance is a good place to start.	Check out the relationship between taking medications and your wellness in My Charts in the Wellness Plan.<br><br>Stay well!

Daily Review Feedback Category 21: Moderate Risk – Medication Adherence, Necessary – Manage Own (Choice 1.3)

Risk (P10)	Questions (S1)	Concerns (P9)	Identity (U14)	Medications (U12)	Toolbox (P10)

1<br>**RANDOM UNLINKED**	2<br>**RANDOM UNLINKED**	3<br>**RANDOM UNLINKED**	4<br>**RANDOM LINKED**	5<br>**RANDOM LINKED**	6<br>**RANDOM UNLINKED**
It appears you're doing well. That's great.<br><br>Remember that taking medications as planned will help you stay well.<br><br>Learn more about common barriers to taking medications…	A lot of things impact people's decisions to take medications. It's complicated.<br><br>Below are common questions people have about medications.<br><br>When it comes to medications, do you wonder about…	There are so many factors that impact people's decision to take or not take medications.<br><br>Read about…	THINK ABOUT IT<br><br>What does it mean to you to have to take medications?<br><br>Does it make you feel defective in some way? Like less of a person? Does it remind you that you have an illness?<br><br>Write down some rational responses to any negative feelings you have about medications.<br><br>For example, say you write down "They make me feel inferior to others." You can generate rational responses, such as "Having an illness does not make me inferior" or "Lots of people have medical problems."	DO SOME RESEARCH<br><br>Get on the DBSA website. Ask others with bipolar disorder about their experiences trying to manage without medications.<br><br>Consider what they have to say and what they have learned from their experiences.	Take a look at "Opinions about Medications" in Toolbox.<br><br>Stay well!

Daily Review Feedback Category 21: Moderate Risk – Medication Adherence, Necessary – Manage Own (Choice 1.3)

Reflect (P9)	Questions (S1)	Concerns (P8)	Reflect (U15)	Workbooks (U13)	My Team (P8)
**1**<br>**RANDOM UNLINKED**	**2**<br>**RANDOM UNLINKED**	**3**<br>**RANDOM UNLINKED**	**4**<br>**RANDOM LINKED**	**5**<br>**RANDOM LINKED**	**6**<br>**RANDOM UNLINKED**
It seems you are well. Good to see.<br><br>Consider how you're taking your medications. Seems like you may be a bit off your plan.<br><br>There are many reasons things might have gotten off.<br><br>Continue to read more…<br>.	A lot of things impact people's decisions to take medications. It's complicated.<br><br>Below are common questions people have about medications.<br><br>When it comes to medications, do you wonder about…	Taking medications is no easy decision, especially when it comes to taking them for years.<br><br>Read about…	THINK ABOUT IT<br><br>Make a list of things having to do with your body that you can manage or change on your own. Make another list of things having to do with your body that you cannot manage or change on your own.<br><br>Is there any pattern to the things you CAN actively change using skills or behaviors? Is there any pattern to the things you CANNOT actively change using skills or behaviors?<br><br>Where do mood episodes fall in these lists? Can you control them with behaviors alone? Without medications?	DO SOME RESEARCH<br><br>Check out current resources for bipolar disorder.<br><br>*The Bipolar Workbook: Tools for Controlling Your Mood Swings. (Basco, 2006)*<br><br>*Managing Bipolar Disorder: A Cognitive---Behavioral Treatment Program Workbook. (Otto, 2008)*<br><br>*The Bipolar Disorder Survival Guide: What You and Your Family Need to Know. (Miklowitz, 2002).*	Consider talking to your supports. Have an open dialogue about your thoughts and feelings about medications. You can review your team in the Wellness Plan.<br><br>Stay well!

Daily Review Feedback Category 21: Moderate Risk – Medication Adherence, Necessary- Medication Help (Choice 1.4)

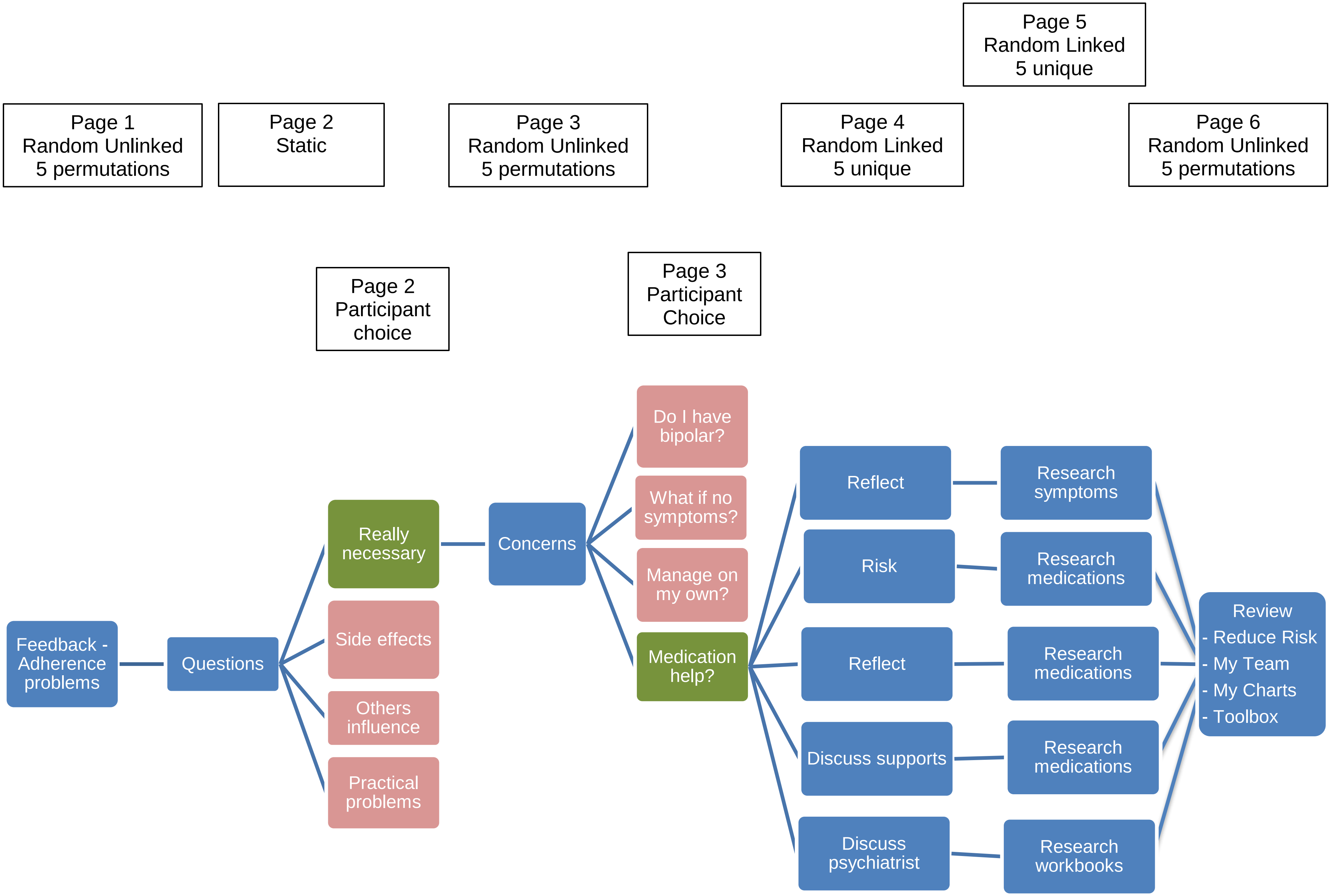

Daily Review Feedback Category 21: Moderate Risk – Medication Adherence, Necessary – Medication Help (Choice 1.4)

Reflect (P6)	Questions (S1)	Concerns (P1)	Reflect (U16)	Symptoms (U1)	(P6)
**1**<br>**RANDOM UNLINKED**	**2**<br>**RANDOM UNLINKED**	**3**<br>**RANDOM UNLINKED**	**4**<br>**RANDOM LINKED**	**5**<br>**RANDOM LINKED**	**6**<br>**RANDOM UNLINKED**
Glad to see you're well. You may want to take a look at your medication schedule. It seems you may not be keeping up as planned.<br><br>Continue to learn about medications.	A lot of things impact people's decisions to take medications. It's complicated.<br><br>Below are common questions people have about medications.<br><br>When it comes to medications, do you wonder about…	Taking medications is a big decision. Most people have reservations! You're not alone.<br><br>Read about…	CHECK IT OUT<br><br>Medicine reduces symptoms and full---blown relapses of depression and mania.<br>Because of this they:<br><br>• Help with self---care<br>• Help maintain friendships<br>• Help with job performance<br>• Reduce hospitalizations<br>• Reduce suicide risk<br><br>What has your experience been with medications?<br>What does your psychiatrist think?<br>How about your therapist?<br>Family and friends?	DO SOME RESEARCH<br><br>There are many good places to read more about bipolar disorder. The more you know, the more able you will be to make informed decisions.<br><br>Read about the symptoms of bipolar disorder.<br><br>• Depression & Bipolar Support Alliance<br>• National Alliance for Mental Illness<br>• National Institute of Mental Health	Be well!

Daily Review Feedback Category 21: Moderate Risk – Medication Adherence, Necessary – Medication Help (Choice 1.4)

Reflect (P7)	Questions (S1)	Concerns (P6)	Risk (U17)	Medications (U14)	Reduce Risk (P7)
**1**<br>**RANDOM UNLINKED**	**2**<br>**RANDOM UNLINKED**	**3**<br>**RANDOM UNLINKED**	**4**<br>**RANDOM LINKED**	**5**<br>**RANDOM LINKED**	**6**<br>**RANDOM UNLINKED**
Good to see you're doing well.<br><br>You may want to take a look at your medication schedule. Taking medications regularly is one of the most important things you can do to stay well.<br><br>Continue to read more…	A lot of things impact people's decisions to take medications. It's complicated.<br><br>Below are common questions people have about medications.<br><br>When it comes to medications, do you wonder about…	Medications should make sense for you. Any questions or problems need to be addressed!<br><br>Read about…	CHECK IT OUT<br><br>On average, medications reduce symptoms and recurrence of mood episodes.<br><br>Remember that it is not just symptoms that are the problem. Mood episodes cause many problems in relationships and work. They can disrupt your life if you end up in the hospital.<br><br>Of course, how well medications work varies from medication to medication and from person to person.	DO SOME RESEARCH<br><br>Ask your psychiatrist for information about your medications. How effective are they on average? What can you expect from them?<br><br>Alternatively, go into a DBSA chat room and ask others about their experiences with your medications.	Check out your medication plan in Reduce Risk in the Wellness Plan.<br><br>Stay well!

Daily Review Feedback Category 21: Moderate Risk – Medication Adherence, Necessary – Medication Help (Choice 1.4)

Reflect (P8)	Questions (S1)	Concerns (P7)	Reflect (U18)	Medications (U15)	My Charts (P3)
**1**<br>**RANDOM UNLINKED**	**2**<br>**RANDOM UNLINKED**	**3**<br>**RANDOM UNLINKED**	**4**<br>**RANDOM LINKED**	**5**<br>**RANDOM LINKED**	**6**<br>**RANDOM UNLINKED**
Nice thing to be well.<br><br>Consider your medication schedule. It appears that things are a bit irregular right now.<br><br>Continue on...	A lot of things impact people's decisions to take medications. It's complicated.<br><br>Below are common questions people have about medications.<br><br>When it comes to medications, do you wonder about…	It is important to get all of your questions and concerns about medications answered. It is your body and your health!<br><br>Read about…	THINK ABOUT IT<br><br>Think about all the different medications you have tried in the past. What has been your experience? Good and bad?<br><br>Did the medications reduce your symptoms? Were you better able to function?	DO SOME RESEARCH<br><br>The Depression and Bipolar Disorder Support Alliance, National Institute of Mental Health, and National Alliance on Mental Illness are all good resources.<br><br>Check out these sites for the latest information on medications for bipolar disorder.	Check out the relationship between taking medications and your wellness in My Charts in the Wellness Plan.<br><br>Stay well!

Daily Review Feedback Category 21: Moderate Risk – Medication Adherence, Necessary – Medication Help (Choice 1.4)

Risk (P10)	Questions (S1)	Concerns (P9)	Discuss (U19)	Medications (U16)	Toolbox (P10)
**1**<br>**RANDOM UNLINKED**	**2**<br>**RANDOM UNLINKED**	**3**<br>**RANDOM UNLINKED**	**4**<br>**RANDOM LINKED**	**5**<br>**RANDOM LINKED**	**6**<br>**RANDOM UNLINKED**
It appears you're doing well. That's great.<br><br>Remember that taking medications as planned will help you stay well.<br><br>Learn more about common barriers to taking medications…	A lot of things impact people's decisions to take medications. It's complicated.<br><br>Below are common questions people have about medications.<br><br>When it comes to medications, do you wonder about…	There are so many factors that impact people's decision to take or not take medications.<br><br>Read about…	CHECK IT OUT<br><br>Sit down with one of your supports. Have an open and honest dialogue about medications.<br><br>What do you think works and what do you think doesn't work? What do they think about medications? What have they noticed in the past with you when it comes to your medications?	DO SOME RESEARCH<br><br>Go online with your support. Read more about bipolar disorder and medications.<br><br>Consider the DBSA, NIMH, or NAMI websites.<br><br>Discuss what you read with your supports or your psychiatrist.	Take a look at "Opinions about Medications" in Toolbox.<br><br>Stay well!

Daily Review Feedback Category 21: Moderate Risk – Medication Adherence, Necessary – Medication Help (Choice 1.4)

Reflect (P9)	Questions (S1)	Concerns (P8)	Discuss (U20)	Workbooks (17)	My Team (P8)
**1**<br>**RANDOM UNLINKED**	**2**<br>**RANDOM UNLINKED**	**3**<br>**RANDOM UNLINKED**	**4**<br>**RANDOM LINKED**	**5**<br>**RANDOM LINKED**	**6**<br>**RANDOM UNLINKED**
It seems you are well. Good to see.<br><br>Consider how you're taking your medications. Seemed like you may be a bit off the plan.<br><br>There are many reasons things might have gotten off.<br><br>Continue to read more…<br>.	A lot of things impact people's decisions to take medications. It's complicated.<br><br>Below are common questions people have about medications.<br><br>When it comes to medications, do you wonder about…	Taking medications is no easy decision, especially when it comes to taking them for years.<br><br>Read about…	CHECK IT OUT<br><br>Talk with your psychiatrist again. Ask them what they notice about you when you are on and off medications.<br><br>An open and honest dialogue is important. Listen to what your psychiatrist has to say. Share your own thoughts and feelings about your experiences.	DO SOME RESEARCH<br><br>Read more…<br><br>*The Bipolar Workbook: Tools for Controlling Your Mood Swings. (Basco, 2006)*<br><br>*Managing Bipolar Disorder: A Cognitive---Behavioral Treatment Program Workbook. (Otto, 2008)*<br><br>*The Bipolar Disorder Survival Guide: What You and Your Family Need to Know. (Miklowitz, 2002).*	Consider talking to your supports. Have an open dialogue about your thoughts and feelings about medications. You can review your team in the Wellness Plan.<br><br>Stay well!

Daily Review Feedback Category 21: Moderate Risk – Medication Adherence, Side Effects - Current (Choice 2.1)

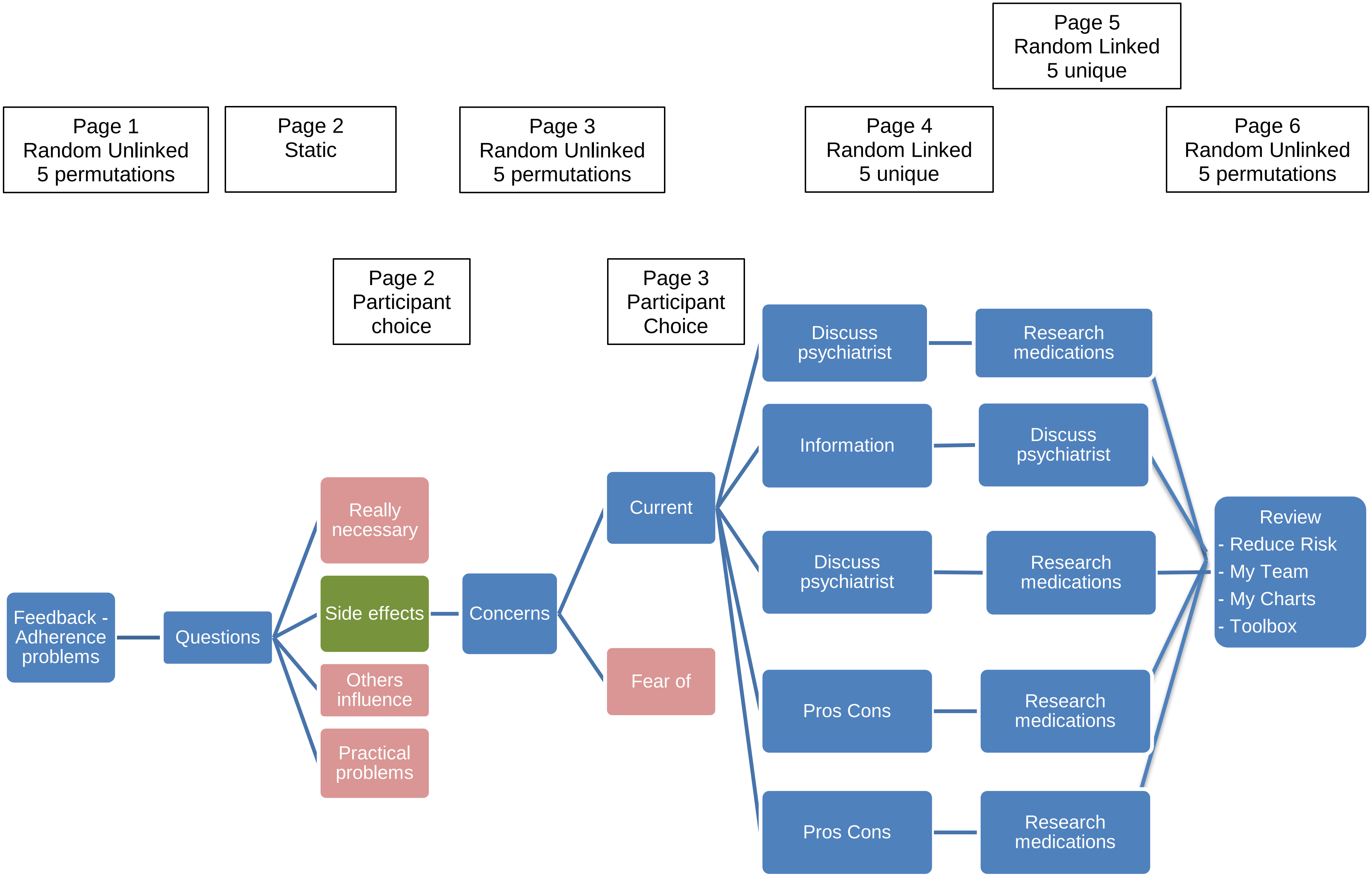

Daily Review Feedback Category 21: Moderate Risk – Medication Adherence, Side Effects – Current (Choice 2.1)

Reflect (P6)	Questions (S1)	Concerns (P1)	Discuss (U21)	Medications (U18)	(P6)
**1**<br>**RANDOM UNLINKED**	**2**<br>**RANDOM UNLINKED**	**3**<br>**RANDOM UNLINKED**	**4**<br>**RANDOM LINKED**	**5**<br>**RANDOM LINKED**	**6**<br>**RANDOM UNLINKED**
Glad to see you're well. You may want to take a look at your medication schedule. It seems you may not be keeping up as planned.<br><br>Continue to learn about medications.	A lot of things impact people's decisions to take medications. It's complicated.<br><br>Below are common questions people have about medications.<br><br>When it comes to medications, do you wonder about…	Taking medications is a big decision. Most people have reservations! You're not alone.<br><br>Read about…	CHECK IT OUT<br><br>There are definitely negative side effects of antidepressants, mood stabilizers, and antipsychotic medications. Sometimes side effects are temporary. Sometimes not. Some side effects are minimal and others too much to tolerate.<br><br>Talk to your psychiatrist:<br><br>• Make sure you are on medications that are right for you<br>• Make sure you are on the lowest dose necessary<br>• Ask if there is a medicine to reduce unwanted side effects	DO SOME RESEARCH<br><br>There are many good places to read more about bipolar disorder. The more you know, the more able you will be to make informed decisions.<br><br>Read about medications for bipolar disorder.<br><br>• Depression & Bipolar Support Alliance<br>• National Alliance for Mental Illness<br>• National Institute of Mental Health	Be well!

Daily Review Feedback Category 21: Moderate Risk – Medication Adherence, Side Effects – Current (Choice 2.1)

Reflect (P7)	Questions (S1)	Concerns (P6)	Information (U22)	Discuss (U19)	Reduce Risk (P7)
**1**<br>**RANDOM UNLINKED**	**2**<br>**RANDOM UNLINKED**	**3**<br>**RANDOM UNLINKED**	**4**<br>**RANDOM LINKED**	**5**<br>**RANDOM LINKED**	**6**<br>**RANDOM UNLINKED**
Good to see you're doing well.<br><br>You may want to take a look at your medication schedule. Taking medications regularly is one of the most important things you can do to stay well.<br><br>Continue to read more…	A lot of things impact people's decisions to take medications. It's complicated.<br><br>Below are common questions people have about medications.<br><br>When it comes to medications, do you wonder about…	Medications should make sense for you. Any questions or problems need to be addressed!<br><br>Read about…	CHECK IT OUT<br><br>Many if not most medications have potential side effects. Of course, not everyone experiences side effects or even the same side effects.<br><br>Most commonly, the side effects of medications start right away and fade over time. In fact, side effects tend to start even before the actual beneficial effects set in! This can make it really hard to stick with the medications.	DO SOME RESEARCH<br><br>Talk to your psychiatrist about your medications.<br><br>• How long until they take effect?<br>• How long until the side effects go away?<br>• How long should you wait to see if the medication is going to help?<br>• How should you weigh the benefits against any long---term side effects?<br><br>Get informed!	Check out your medication plan in Reduce Risk in the Wellness Plan.<br><br>Stay well!

Daily Review Feedback Category 21: Moderate Risk – Medication Adherence, Side Effects – Current (Choice 2.1)

Reflect (P8)	Questions (S1)	Concerns (P7)	Discuss (U23)	Medications (U20)	My Charts (P3)
**1**<br>**RANDOM UNLINKED**	**2**<br>**RANDOM UNLINKED**	**3**<br>**RANDOM UNLINKED**	**4**<br>**RANDOM LINKED**	**5**<br>**RANDOM LINKED**	**6**<br>**RANDOM UNLINKED**
Nice thing to be well.<br><br>Consider your medication schedule. It appears that things are a bit irregular right now.<br><br>Continue on...	A lot of things impact people's decisions to take medications. It's complicated.<br><br>Below are common questions people have about medications.<br><br>When it comes to medications, do you wonder about…	It is important to get all of your questions and concerns about medications answered. It is your body and your health!<br><br>Read about…	CHECK IT OUT<br><br>Don't make a go of this alone.<br><br>If you have stopped your medications because of side effects, it is definitely time to talk with your psychiatrist.<br><br>They are there to help you figure out the best medication plan. This is an individualized plan. No two people are alike in terms of benefits and side effects of medications.<br><br>Reach out! Speak up!	DO SOME RESEARCH<br><br>Read about medications for bipolar disorder. Read about the potential benefits, as well as the potential side effects. Get informed ahead of time.<br><br>The Depression and Bipolar Disorder Support Alliance, National Institute of Mental Health, and National Alliance on Mental Illness are all good resources.	Check out the relationship between taking medications and your wellness in My Charts in the Wellness Plan.<br><br>Stay well!

Daily Review Feedback Category 21: Moderate Risk – Medication Adherence, Side Effects – Current (Choice 2.1)

Risk (P10)	Questions (S1)	Concerns (P9)	Pros Cons (U24)	Medications (U21)	Toolbox (P10)
**1<br>RANDOM UNLINKED**	**2<br>RANDOM UNLINKED**	**3<br>RANDOM UNLINKED**	**4<br>RANDOM LINKED**	**5<br>RANDOM LINKED**	**6<br>RANDOM UNLINKED**
It appears you're doing well. That's great.<br><br>Remember that taking medications as planned will help you stay well.<br><br>Learn more about common barriers to taking medications…	A lot of things impact people's decisions to take medications. It's complicated.<br><br>Below are common questions people have about medications.<br><br>When it comes to medications, do you wonder about…	There are so many factors that impact people's decision to take or not take medications.<br><br>Read about…	CHECK IT OUT<br><br>Write down all the benefits you are experiencing from the medications. On a scale of 1 to 5, rate how important each of these benefits is to you. (One is not at all, 3 is somewhat, and 5 is very much so.)<br><br>Now write down all the side effects that you are experiencing from the medications. On a scale of 1 to 5, rate how troublesome they are for you. (One is not at all, 3 is somewhat, and 5 is very much so.)	DO SOME RESEARCH<br><br>Read online about the benefits and side effects of your medications. See if there is anything to add to your lists.<br><br>Then show your psychiatrist your ratings. Get some feedback from them.	Take a look at "Opinions about Medications" in Toolbox.<br><br>Stay well!

Daily Review Feedback Category 21: Moderate Risk – Medication Adherence, Side Effects – Current (Choice 2.1)

Reflect (P9)	Questions (S1)	Concerns (P8)	Pros Cons (U25)	Medications (U22)	My Team (P8)
**1**<br>**RANDOM UNLINKED**	**2**<br>**RANDOM UNLINKED**	**3**<br>**RANDOM UNLINKED**	**4**<br>**RANDOM LINKED**	**5**<br>**RANDOM LINKED**	**6**<br>**RANDOM UNLINKED**
It seems you are well. Good to see.<br><br>Consider how you're taking your medications. Seemed like you may be a bit off the plan.<br><br>There are many reasons things might have gotten off.<br><br>Continue to read more…<br>.	A lot of things impact people's decisions to take medications. It's complicated.<br><br>Below are common questions people have about medications.<br><br>When it comes to medications, do you wonder about…	Taking medications is no easy decision, especially when it comes to taking them for years.<br><br>Read about…	THINK ABOUT IT<br><br>Side effects range from annoying to intolerable. Where on this spectrum do things fall for you?<br><br>Of course having no side effects would be best. But consider the big picture. Are your medications working? Are there side effects that you can live with? Are there side effects that you cannot live with?	DO SOME RESEARCH<br><br>Sometimes it is hard to decide whether you should change medications or whether you should find ways to cope with the side effects.<br><br>Get on the DBSA or NAMI website. See how others have dealt with this issue.	Consider talking to your supports. Have an open dialogue about your thoughts and feelings about medications. You can review your team in the Wellness Plan.<br><br>Stay well!

Daily Review Feedback Category 21: Moderate Risk – Medication Adherence, Side Effects – Fear of (Choice 2.2)

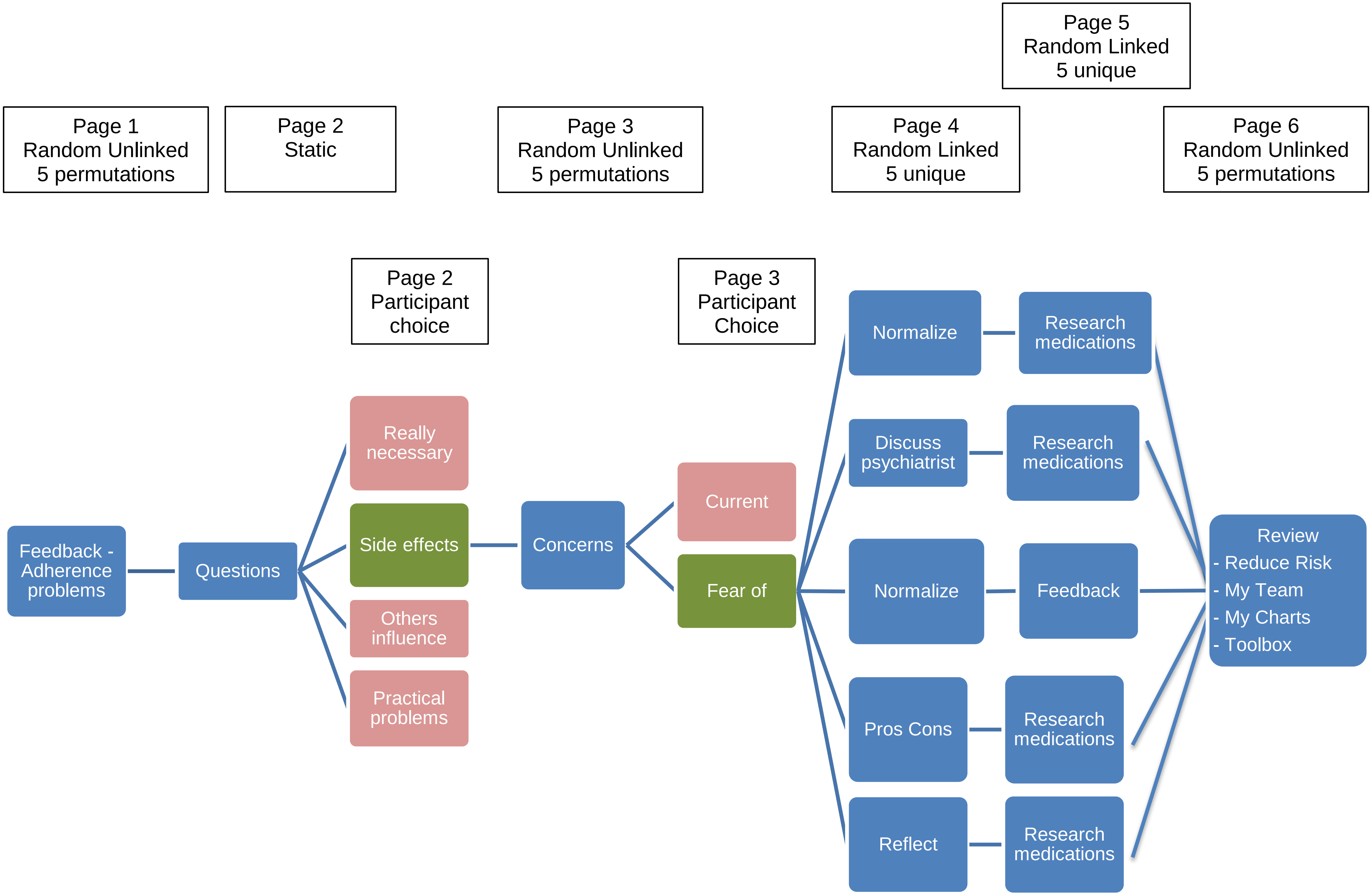

Daily Review Feedback Category 21: Moderate Risk – Medication Adherence, Side Effects – Fears (Choice 2.2)

Reflect (P6)	Questions (S1)	Concerns (P1)	Normalize (U26)	Medications (U18)	(P6)
**1**<br>**RANDOM UNLINKED**	**2**<br>**RANDOM UNLINKED**	**3**<br>**RANDOM UNLINKED**	**4**<br>**RANDOM LINKED**	**5**<br>**RANDOM LINKED**	**6**<br>**RANDOM UNLINKED**
Glad to see you're well. You may want to take a look at your medication schedule. It seems you may not be keeping up as planned.<br><br>Continue to learn about medications.	A lot of things impact people's decisions to take medications. It's complicated.<br><br>Below are common questions people have about medications.<br><br>When it comes to medications, do you wonder about...	Taking medications is a big decision. Most people have reservations! You're not alone.<br><br>Read about...	CHECK IT OUT<br><br>It is not uncommon for people to fear getting addicted or dependent on medications.<br><br>The medications typically prescribed for bipolar disorder are not addictive. However, it is true that you will need to depend on them to stay well.<br><br>There is some evidence that actually being un---medicated is bad for the brains of people with bipolar disorder!	DO SOME RESEARCH<br><br>There are many good places to read more about bipolar disorder. The more you know, the more able you will be to make informed decisions.<br><br>Read about medications for bipolar disorder.<br><br>• Depression & Bipolar Support Alliance<br>• National Alliance for Mental Illness<br>• National Institute of Mental Health	Be well!

Daily Review Feedback Category 21: Moderate Risk – Medication Adherence, Side Effects – Fears (Choice 2.2)

Reflect (P7)	Questions (S1)	Concerns (P6)	Discuss (U27)	Medications (U23)	Reduce Risk (P7)
**1**<br>**RANDOM UNLINKED**	**2**<br>**RANDOM UNLINKED**	**3**<br>**RANDOM UNLINKED**	**4**<br>**RANDOM LINKED**	**5**<br>**RANDOM LINKED**	**6**<br>**RANDOM UNLINKED**
Good to see you're doing well.<br><br>You may want to take a look at your medication schedule. Taking medications regularly is one of the most important things you can do to stay well.<br><br>Continue to read more…	A lot of things impact people's decisions to take medications. It's complicated.<br><br>Below are common questions people have about medications.<br><br>When it comes to medications, do you wonder about…	Medications should make sense for you. Any questions or problems need to be addressed!<br><br>Read about…	CHECK IT OUT<br><br>It is good to be cautious about the medications you take. Check it out, be informed.<br><br>There are sometimes long---term side effects of mood stabilizers and antipsychotics. Read more about it to find out for yourself. Talk with your psychiatrist about any concerns you have.	DO SOME RESEARCH<br><br>There are many resources available online to learn about the effects, side effects, and long---term consequences of taking medications.<br><br>Read up on your medications. Consider the balance of effects and side effects when making decisions with your psychiatrist.	Check out your medication plan in Reduce Risk in the Wellness Plan.<br><br>Stay well!

Daily Review Feedback Category 21: Moderate Risk – Medication Adherence, Side Effects – Fears (Choice 2.2)

Reflect (P8)	Questions (S1)	Concerns (P7)	Normalize (U28)	Feedback (U24)	My Charts (P3)
**1**<br>**RANDOM UNLINKED**	**2**<br>**RANDOM UNLINKED**	**3**<br>**RANDOM UNLINKED**	**4**<br>**RANDOM LINKED**	**5**<br>**RANDOM LINKED**	**6**<br>**RANDOM UNLINKED**
Nice thing to be well.<br><br>Consider your medication schedule. It appears that things are a bit irregular right now.<br><br>Continue on...	A lot of things impact people's decisions to take medications. It's complicated.<br><br>Below are common questions people have about medications.<br><br>When it comes to medications, do you wonder about…	It is important to get all of your questions and concerns about medications answered. It is your body and your health!<br><br>Read about…	CHECK IT OUT<br><br>Most people have concerns about the side effects of medications. It is important to be informed, to be wise about what you put in your body.<br><br>Read up on medications for bipolar disorder. Read about the positive and negative long---term effects of taking medications.	DO SOME RESEARCH<br><br>After you've read up on your medications, discuss what you learned with your supports and psychiatrist.<br><br>See what feedback they have.	Check out the relationship between taking medications and your wellness in My Charts in the Wellness Plan.<br><br>Stay well!

Daily Review Feedback Category 21: Moderate Risk – Medication Adherence, Side Effects – Fears (Choice 2.2)

Risk (P10)	Questions (S1)	Concerns (P9)	Pros Cons (U29)	Medications (U25)	Toolbox (P10)

1<br>RANDOM UNLINKED	2<br>RANDOM UNLINKED	3<br>RANDOM UNLINKED	4<br>RANDOM LINKED	5<br>RANDOM LINKED	6<br>RANDOM UNLINKED
It appears you're doing well. That's great.<br><br>Remember that taking medications as planned will help you stay well.<br><br>Learn more about common barriers to taking medications…	A lot of things impact people's decisions to take medications. It's complicated.<br><br>Below are common questions people have about medications.<br><br>When it comes to medications, do you wonder about…	There are so many factors that impact people's decision to take or not take medications.<br><br>Read about…	CHECK IT OUT<br><br>Write down all the benefits you are experiencing from the medications. Do they help with symptoms? Do they help with how you function?<br><br>Now write down the fears you have about future side effects. What do you think will happen?	DO SOME RESEARCH<br><br>Now read about the effects of medications. Look online. Do you want to add anything to the list of positive effects medications have for you?<br><br>Now read online about the long---term effects of taking medications. What does the research say can happen? Does this information increase or decrease your fears about future side effects?	Take a look at "Opinions about Medications" in Toolbox.<br><br>Stay well!

Daily Review Feedback Category 21: Moderate Risk – Medication Adherence, Side Effects – Fears (Choice 2.2)

Reflect (P9)	Questions (S1)	Concerns (P8)	Reflect (U30)	Medications (U26)	My Team (P8)
**1**<br>**RANDOM UNLINKED**	**2**<br>**RANDOM UNLINKED**	**3**<br>**RANDOM UNLINKED**	**4**<br>**RANDOM LINKED**	**5**<br>**RANDOM LINKED**	**6**<br>**RANDOM UNLINKED**
It seems you are well. Good to see.<br><br>Consider how you're taking your medications. Seemed like you may be a bit off the plan.<br><br>There are many reasons things might have gotten off.<br><br>Continue to read more…<br>.	A lot of things impact people's decisions to take medications. It's complicated.<br><br>Below are common questions people have about medications.<br><br>When it comes to medications, do you wonder about…	Taking medications is no easy decision, especially when it comes to taking them for years.<br><br>Read about…	CHECK IT OUT<br><br>What do you fear about long--- term use of medications?<br><br>What do you already know about using medications for years?<br><br>What do you need to learn about long---term use of medications?	DO SOME RESEARCH<br><br>Get on the DBSA website and talk to others who have taken medications for a long time. See what their experiences have been.<br><br>Discuss what you learn with your psychiatrist.	Consider talking to your supports. Have an open dialogue about your thoughts and feelings about medications. You can review your team in the Wellness Plan.<br><br>Stay well!

Daily Review Feedback Category 21: Moderate Risk – Medication Adherence, Others Influence – Psychiatrist (Choice 3.1)

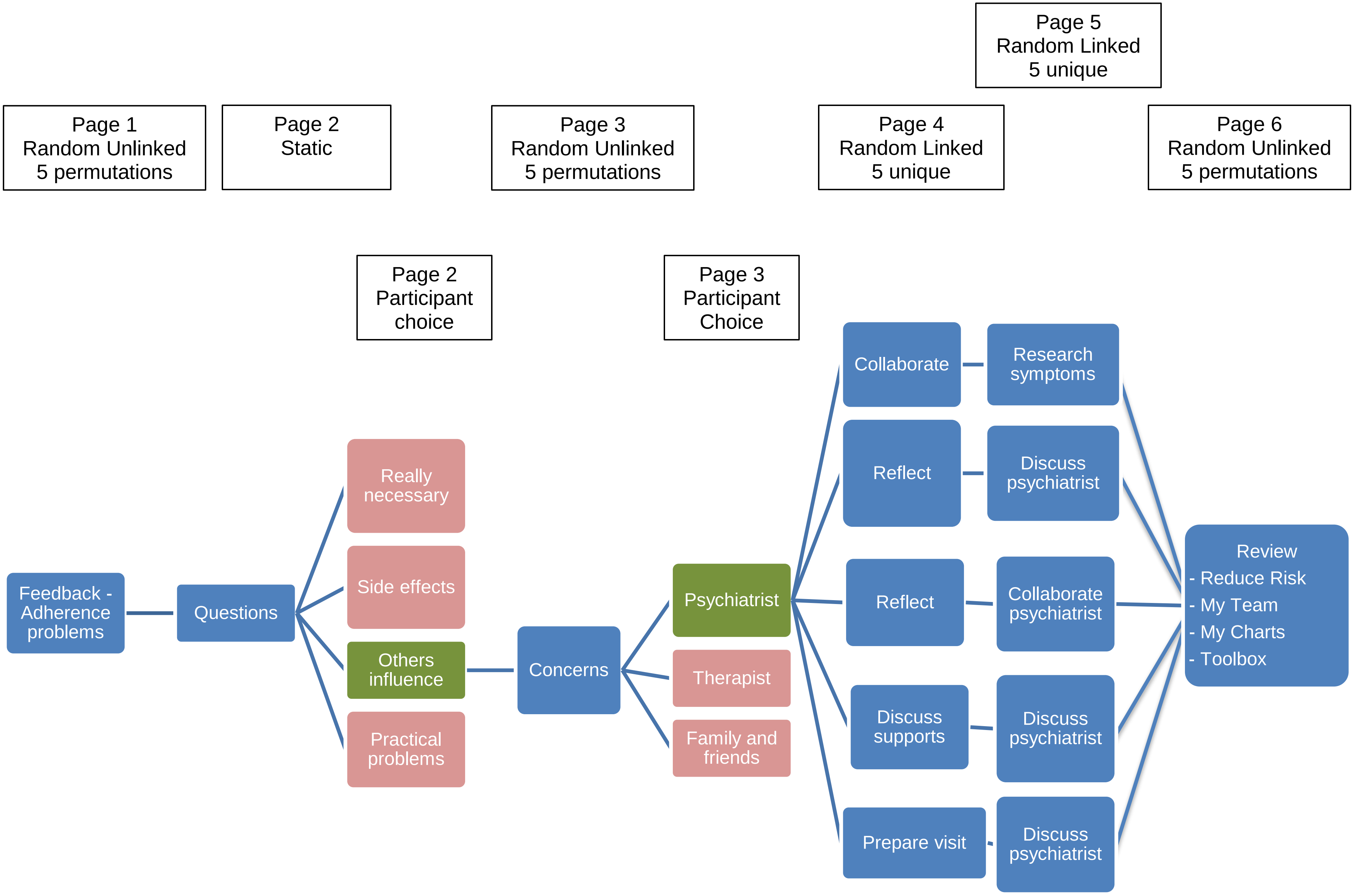

Daily Review Feedback Category 21: Moderate Risk – Medication Adherence, Influence Others – Psychiatrist (Choice 3.1)

Reflect (P6)	Questions (S1)	Concerns (P1)	Collaborate (U31)	Symptoms (U1)	(P6)
**1**<br>**RANDOM UNLINKED**	**2**<br>**RANDOM UNLINKED**	**3**<br>**RANDOM UNLINKED**	**4**<br>**RANDOM LINKED**	**5**<br>**RANDOM LINKED**	**6**<br>**RANDOM UNLINKED**
Glad to see you're well. You may want to take a look at your medication schedule. It seems you may not be keeping up as planned.<br><br>Continue to learn about medications.	A lot of things impact people's decisions to take medications. It's complicated.<br><br>Below are common questions people have about medications.<br><br>When it comes to medications, do you wonder about…	Taking medications is a big decision. Most people have reservations! You're not alone.<br><br>Read about…	CHECK IT OUT<br><br>Having a good relationship with your psychiatrist will influence how and when you take medications.<br><br>• Find a doctor who is a good match. You must be comfortable with their level of expertise and their personality.<br>• Work on an open dialogue with your doctor. They are the expert on medications. You are the expert on your needs and goals.<br>• Be sure to tell your doctor if they are making you uneasy. Try to work it out.	DO SOME RESEARCH<br><br>There are many good places to read more about bipolar disorder. The more you know, the more able you will be to make informed decisions.<br><br>Read about the symptoms of bipolar disorder.<br><br>• Depression & Bipolar Support Alliance<br>• National Alliance for Mental Illness<br>• National Institute of Mental Health	Be well!

Daily Review Feedback Category 21: Moderate Risk – Medication Adherence, Influence Others – Psychiatrist (Choice 3.1)

Reflect (P7)	Questions (S1)	Concerns (P6)	Reflect (U32)	Discuss (U27)	Reduce Risk (P7)
**1**<br>**RANDOM UNLINKED**	**2**<br>**RANDOM UNLINKED**	**3**<br>**RANDOM UNLINKED**	**4**<br>**RANDOM LINKED**	**5**<br>**RANDOM LINKED**	**6**<br>**RANDOM UNLINKED**
Good to see you're doing well.<br><br>You may want to take a look at your medication schedule. Taking medications regularly is one of the most important things you can do to stay well.<br><br>Continue to read more…	A lot of things impact people's decisions to take medications. It's complicated.<br><br>Below are common questions people have about medications.<br><br>When it comes to medications, do you wonder about…	Medications should make sense for you. Any questions or problems need to be addressed!<br><br>Read about…	CHECK IT OUT<br><br>Is there something going on in your working relationship with your psychiatrist that is getting in the way of taking medications?<br><br>The following characteristics in a doctor promote a good working relationship:<br><br>• Flexible<br>• Honest<br>• Respectful<br>• Trustworthy<br>• Confident<br>• Warm<br>• Interested<br>• Open<br><br>How does your psychiatrist measure up?	TALK IT OUT<br><br>If you have concerns about your working relationship with your psychiatrist, talk to them. Let them know how you feel.<br><br>Oftentimes, these types of conversations lead to a better relationship.	Check out your medication plan in Reduce Risk in the Wellness Plan.<br><br>Stay well!

Daily Review Feedback Category 21: Moderate Risk – Medication Adherence, Influence Others – Psychiatrist (Choice 3.1)

Reflect (P8)	Questions (S1)	Concerns (P7)	Reflect (U33)	Collaborate (U28)	My Charts (P3)
**1**<br>**RANDOM UNLINKED**	**2**<br>**RANDOM UNLINKED**	**3**<br>**RANDOM UNLINKED**	**4**<br>**RANDOM LINKED**	**5**<br>**RANDOM LINKED**	**6**<br>**RANDOM UNLINKED**
Nice thing to be well.<br><br>Consider your medication schedule. It appears that things are a bit irregular right now.<br><br>Continue on...	A lot of things impact people's decisions to take medications. It's complicated.<br><br>Below are common questions people have about medications.<br><br>When it comes to medications, do you wonder about…	It is important to get all of your questions and concerns about medications answered. It is your body and your health!<br><br>Read about…	CHECK IT OUT<br><br>Health outcomes are better when there is agreement on treatment goals.<br><br>Do you and your psychiatrist agree on your goals?	TALK IT OUT<br><br>Talk to your psychiatrist. Make sure you are on the same page. Collaboration is the key to a good working relationship!<br><br>Consider having a practice discussion with your support before you go in to talk with your doctor.	Check out the relationship between taking medications and your wellness in My Charts in the Wellness Plan.<br><br>Stay well!

Daily Review Feedback Category 21: Moderate Risk – Medication Adherence, Influence Others – Psychiatrist (Choice 3.1)

Risk (P10)	Questions (S1)	Concerns (P9)	Discuss (U34)	Discuss (U29)	Toolbox (P10)
**1**<br>**RANDOM UNLINKED**	**2**<br>**RANDOM UNLINKED**	**3**<br>**RANDOM UNLINKED**	**4**<br>**RANDOM LINKED**	**5**<br>**RANDOM LINKED**	**6**<br>**RANDOM UNLINKED**
It appears you're doing well. That's great.<br><br>Remember that taking medications as planned will help you stay well.<br><br>Learn more about common barriers to taking medications…	A lot of things impact people's decisions to take medications. It's complicated.<br><br>Below are common questions people have about medications.<br><br>When it comes to medications, do you wonder about…	There are so many factors that impact people's decision to take or not take medications.<br><br>Read about…	CHECK IT OUT<br><br>How is it that your relationship with your psychiatrist is influencing you? What is going on that you don't want to take the medications as prescribed?<br><br>Sit down with one of your supports. Talk about your thoughts and feelings about your psychiatrist. Come up with a plan of action to address your concerns.	TALK IT OUT<br><br>Meet with your psychiatrist and share your concerns. It is important that the two of you are on the same page.<br><br>Remember that your psychiatrist is the expert on medications, you are the expert on yourself, and the two of you cooperate to put all of this together to come up with a good medication plan.	Take a look at "Opinions about Medications" in Toolbox.<br><br>Stay well!

Daily Review Feedback Category 21: Moderate Risk – Medication Adherence, Influence Others – Psychiatrist (Choice 3.1)

Reflect (P9)	Questions (S1)	Concerns (P8)	Prepare (U35)	Discuss (U30)	Team (P8)
**1**<br>**RANDOM UNLINKED**	**2**<br>**RANDOM UNLINKED**	**3**<br>**RANDOM UNLINKED**	**4**<br>**RANDOM LINKED**	**5**<br>**RANDOM LINKED**	**6**<br>**RANDOM UNLINKED**
It seems you are well. Good to see.<br><br>Consider how you're taking your medications. Seemed like you may be a bit off the plan.<br><br>There are many reasons things might have gotten off.<br><br>Continue to read more…<br>.	A lot of things impact people's decisions to take medications. It's complicated.<br><br>Below are common questions people have about medications.<br><br>When it comes to medications, do you wonder about…	Taking medications is no easy decision, especially when it comes to taking them for years.<br><br>Read about…	CHECK IT OUT<br><br>Prepare for your next visit. Write down your life goals and medication goals. Write down side effects that are intolerable. Write down any concerns you have about how your psychiatrist is working with you.<br><br>What would you like to see happen?<br><br>Ask a support to role play. Practice communicating your concerns in preparation for your medication appointment.	TALK IT OUT<br><br>Have an open and honest dialogue with your psychiatrist.<br><br>Make sure the two of you are on the same page.	Consider talking to your supports. Have an open dialogue about your thoughts and feelings about medications. You can review your team in the Wellness Plan.<br><br>Stay well!

Daily Review Feedback Category 21: Moderate Risk – Medication Adherence, Others Influence – Therapist (Choice 3.2)

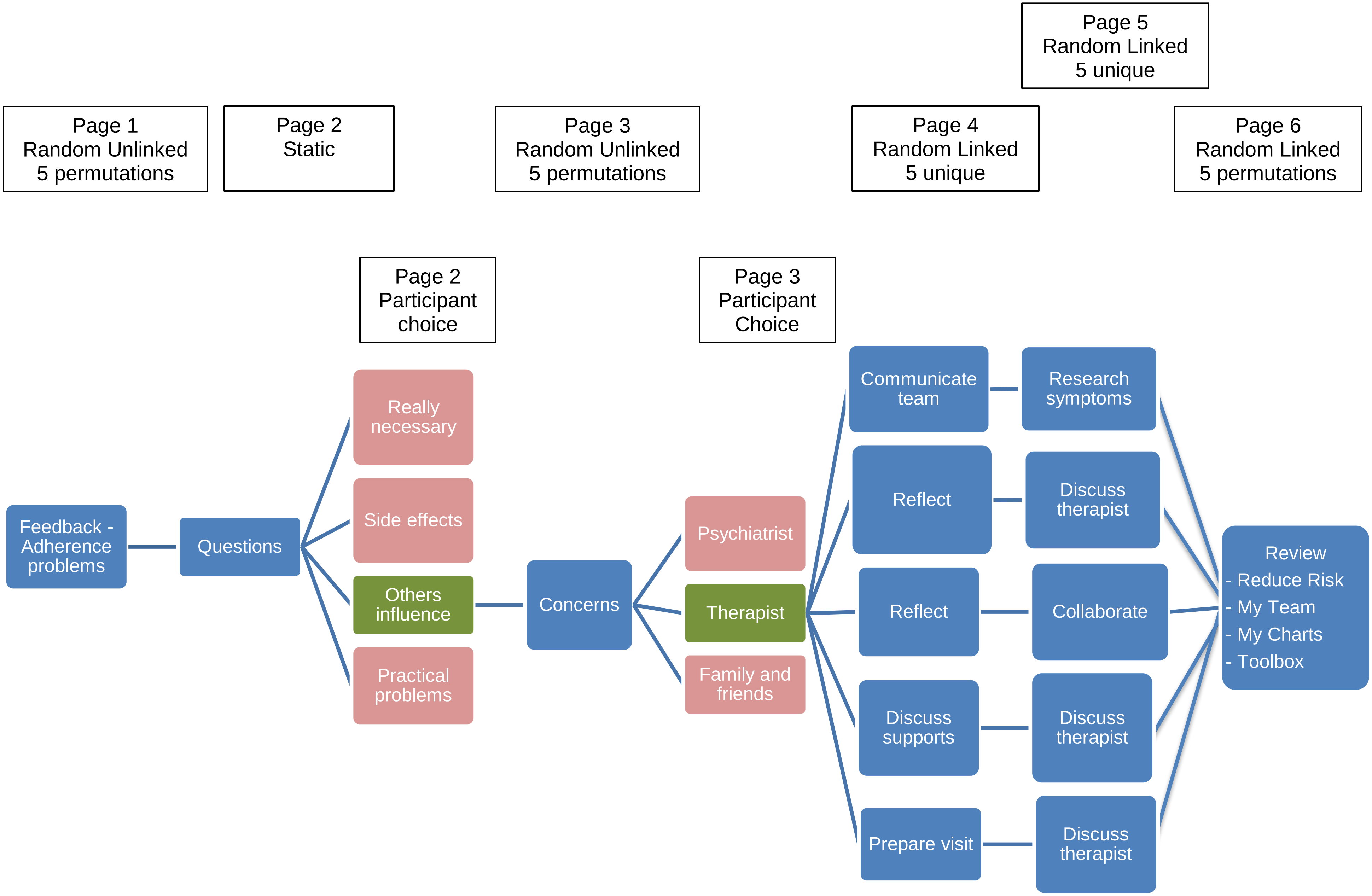

Daily Review Feedback Category 21: Moderate Risk – Medication Adherence, Others Influence – Therapist (Choice 3.2)

Reflect (P6)	Questions (S1)	Concerns (P1)	Communicate (U36)	Symptoms (U1)	(P6)
**1**<br>**RANDOM UNLINKED**	**2**<br>**RANDOM UNLINKED**	**3**<br>**RANDOM UNLINKED**	**4**<br>**RANDOM LINKED**	**5**<br>**RANDOM LINKED**	**6**<br>**RANDOM UNLINKED**
Glad to see you're well. You may want to take a look at your medication schedule. It seems you may not be keeping up as planned.<br><br>Continue to learn about medications.	A lot of things impact people's decisions to take medications. It's complicated.<br><br>Below are common questions people have about medications.<br><br>When it comes to medications, do you wonder about…	Taking medications is a big decision. Most people have reservations! You're not alone.<br><br>Read about…	CHECK IT OUT<br><br>It is important that your therapist and psychiatrist are on the same page. You need a united team.<br><br>• Your therapist should support medications.<br>• Be sure your therapist and psychiatrist are communicating (they should be doing this) with one another about your overall treatment.<br>• If this is happening and you continue to get advice from your psychiatrist and therapist that conflict, you may need to change providers.	DO SOME RESEARCH<br><br>There are many good places to read more about bipolar disorder. The more you know, the more able you will be to make informed decisions.<br><br>Read about the symptoms of bipolar disorder.<br><br>• Depression & Bipolar Support Alliance<br>• National Alliance for Mental Illness<br>• National Institute of Mental Health	Be well!

Daily Review Feedback Category 21: Moderate Risk – Medication Adherence, Others Influence – Therapist (Choice 3.2)

Reflect (P7)	Questions (S1)	Concerns (P6)	Reflect (U37)	Discuss (U31)	Reduce Risk (P7)
**1**<br>**RANDOM UNLINKED**	**2**<br>**RANDOM UNLINKED**	**3**<br>**RANDOM UNLINKED**	**4**<br>**RANDOM LINKED**	**5**<br>**RANDOM LINKED**	**6**<br>**RANDOM UNLINKED**
Good to see you're doing well.<br><br>You may want to take a look at your medication schedule. Taking medications regularly is one of the most important things you can do to stay well.<br><br>Continue to read more…	A lot of things impact people's decisions to take medications. It's complicated.<br><br>Below are common questions people have about medications.<br><br>When it comes to medications, do you wonder about…	Medications should make sense for you. Any questions or problems need to be addressed!<br><br>Read about…	CHECK IT OUT<br><br>Is there something going on in your working relationship with your therapist that is getting in the way of taking medications?<br><br>The following characteristics in a therapist promote a good working relationship:<br><br>• Flexible<br>• Honest<br>• Respectful<br>• Trustworthy<br>• Confident<br>• Warm<br>• Interested<br>• Open<br><br>How does your therapist measure up?	TALK IT OUT<br><br>If you have concerns about your working relationship with your therapist, talk to them. Let them know how you feel.<br><br>Oftentimes, these types of conversations lead to a better relationship.	Check out your medication plan in Reduce Risk in the Wellness Plan.<br><br>Stay well!

Daily Review Feedback Category 21: Moderate Risk – Medication Adherence, Others Influence – Therapist (Choice 3.2)

Reflect (P8)	Questions (S1)	Concerns (P7)	Reflect (U38)	Collaborate (U32)	My Charts (P3)
**1**<br>**RANDOM UNLINKED**	**2**<br>**RANDOM UNLINKED**	**3**<br>**RANDOM UNLINKED**	**4**<br>**RANDOM LINKED**	**5**<br>**RANDOM LINKED**	**6**<br>**RANDOM UNLINKED**
Nice thing to be well.<br><br>Consider your medication schedule. It appears that things are a bit irregular right now.<br><br>Continue on...	A lot of things impact people's decisions to take medications. It's complicated.<br><br>Below are common questions people have about medications.<br><br>When it comes to medications, do you wonder about…	It is important to get all of your questions and concerns about medications answered. It is your body and your health!<br><br>Read about…	CHECK IT OUT<br><br>Health outcomes are better when there is agreement on treatment goals.<br><br>Do you and your therapist agree on your goals?	TALK IT OUT<br><br>Talk to your therapist. Make sure you are on the same page. Collaboration is the key to a good working relationship!<br><br>Consider having a practice discussion with your support before you go in to talk with your therapist.	Check out the relationship between taking medications and your wellness in My Charts in the Wellness Plan.<br><br>Stay well!

Daily Review Feedback Category 21: Moderate Risk – Medication Adherence, Others Influence – Therapist (Choice 3.2)

Risk (P10)	Questions (S1)	Concerns (P9)	Discuss (U39)	Discuss (U33)	Toolbox (P10)
**1**<br>**RANDOM UNLINKED**	**2**<br>**RANDOM UNLINKED**	**3**<br>**RANDOM UNLINKED**	**4**<br>**RANDOM LINKED**	**5**<br>**RANDOM LINKED**	**6**<br>**RANDOM UNLINKED**
It appears you're doing well. That's great.<br><br>Remember that taking medications as planned will help you stay well.<br><br>Learn more about common barriers to taking medications…	A lot of things impact people's decisions to take medications. It's complicated.<br><br>Below are common questions people have about medications.<br><br>When it comes to medications, do you wonder about…	There are so many factors impact people's decision to take or not take medications.<br><br>Read about…	CHECK IT OUT<br><br>How is it that your relationship with your therapist influencing you? What is going on that you don't want to take the medications as prescribed?<br><br>Sit down with one of your supports. Talk about your thoughts and feelings about your therapist. Come up with a plan of action to address your concerns.	TALK IT OUT<br><br>Meet with your therapist and share your concerns. It is important that the two of you are on the same page.<br><br>Remember that your therapist is the expert on mental health, you are the expert on yourself, and the two of you cooperate to put all of this together to come up with a good plan. This plan must be in sync with your medication plan!	Take a look at "Opinions about Medications" in Toolbox.<br><br>Stay well!

Daily Review Feedback Category 21: Moderate Risk – Medication Adherence, Others Influence – Therapist (Choice 3.2)

Reflect (P9)	Questions (S1)	Concerns (P8)	Prepare (U40)	Discuss (U34)	Team (P8)
**1**<br>**RANDOM UNLINKED**	**2**<br>**RANDOM UNLINKED**	**3**<br>**RANDOM UNLINKED**	**4**<br>**RANDOM LINKED**	**5**<br>**RANDOM LINKED**	**6**<br>**RANDOM UNLINKED**
It seems you are well. Good to see.<br><br>Consider how you're taking your medications. Seemed like you may be a bit off the plan.<br><br>There are many reasons things might have gotten off.<br><br>Continue to read more…<br>.	A lot of things impact people's decisions to take medications. It's complicated.<br><br>Below are common questions people have about medications.<br><br>When it comes to medications, do you wonder about…	Taking medications is no easy decision, especially when it comes to taking them for years.<br><br>Read about…	CHECK IT OUT<br><br>Prepare for your next visit. Write down your life goals and therapy goals. Write down any concerns you have about how your therapist is working with you.<br><br>What would you like to see happen?<br><br>Ask a support to role play. Practice communicating your concerns in preparation for your appointment.	TALK IT OUT<br><br>Have an open and honest dialogue with your therapist. Make sure the two of you are on the same page.<br><br>Make sure your psychiatrist and therapist are also communicating. They too need to be on the same page!	Consider talking to your supports. Have an open dialogue about your thoughts and feelings about medications. You can review your team in the Wellness Plan.<br><br>Stay well!

Daily Review Feedback Category 21: Moderate Risk – Medication Adherence, Others Influence – Family and Friends (Choice 3.3)

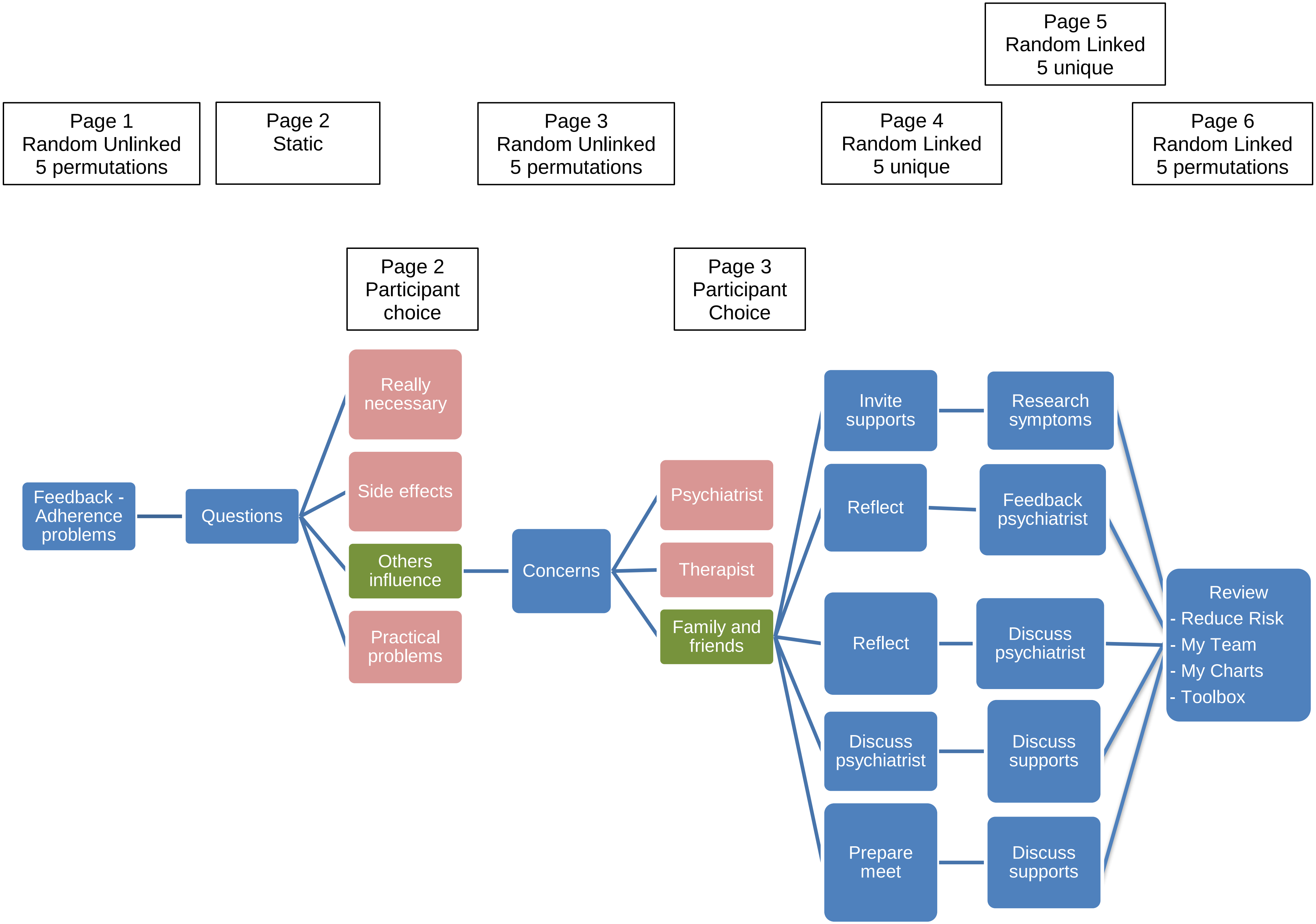

Daily Review Feedback Category 21: Moderate Risk – Medication Adherence, Others Influence – Family and Friends (Choice 3.3)

Reflect (P6)	Questions (S1)	Concerns (P1)	Invite (U41)	Symptoms (U1)	(P6)
**1**<br>**RANDOM UNLINKED**	**2**<br>**RANDOM UNLINKED**	**3**<br>**RANDOM UNLINKED**	**4**<br>**RANDOM LINKED**	**5**<br>**RANDOM LINKED**	**6**<br>**RANDOM UNLINKED**
Glad to see you're well. You may want to take a look at your medication schedule. It seems you may not be keeping up as planned.<br><br>Continue to learn about medications.	A lot of things impact people's decisions to take medications. It's complicated.<br><br>Below are common questions people have about medications.<br><br>When it comes to medications, do you wonder about…	Taking medications is a big decision. Most people have reservations! You're not alone.<br><br>Read about…	CHECK IT OUT<br><br>Sometimes family and friends are against medications.<br>Common reasons are:<br><br>• Denial of your illness<br>• Mistrust of the medical community<br><br>Remember that having the support of your team improves your chances of wellness.<br><br>Invite family and friends to read about bipolar disorder, or even to attend one of your doctor appointments.	DO SOME RESEARCH<br><br>There are many good places to read more about bipolar disorder. The more you know, the more able you will be to make informed decisions.<br><br>Read about the symptoms of bipolar disorder.<br><br>• Depression & Bipolar Support Alliance<br>• National Alliance for Mental Illness<br>• National Institute of Mental Health	Be well!

Daily Review Feedback Category 21: Moderate Risk – Medication Adherence, Others Influence – Family and Friends (Choice 3.3)

Reflect (P7)	Questions (S1)	Concerns (P6)	Reflect (U42)	Feedback (U35)	Reduce Risk (P7)
**1**<br>**RANDOM UNLINKED**	**2**<br>**RANDOM UNLINKED**	**3**<br>**RANDOM UNLINKED**	**4**<br>**RANDOM LINKED**	**5**<br>**RANDOM LINKED**	**6**<br>**RANDOM UNLINKED**
Good to see you're doing well.<br><br>You may want to take a look at your medication schedule. Taking medications regularly is one of the most important things you can do to stay well.<br><br>Continue to read more…	A lot of things impact people's decisions to take medications. It's complicated.<br><br>Below are common questions people have about medications.<br><br>When it comes to medications, do you wonder about…	Medications should make sense for you. Any questions or problems need to be addressed!<br><br>Read about…	CHECK IT OUT<br><br>Are the opinions of your family and friends impacting how or when you take medications?<br><br>What are they saying about your illness, or medications, or doctor? Are they well informed about bipolar disorder and its treatment?	TALK IT OUT<br><br>Talk to your psychiatrist about the opinions of your family and friends. Let your psychiatrist know how their opinions are impacting you.<br><br>Get some feedback about what to do. Get some feedback about how to talk to family and friends. You need their support!	Check out your medication plan in Reduce Risk in the Wellness Plan.<br><br>Stay well!

Daily Review Feedback Category 21: Moderate Risk – Medication Adherence, Others Influence – Family and Friends (Choice 3.3)

Reflect (P8)	Questions (S1)	Concerns (P7)	Reflect (U43)	Discuss (U36)	My Charts (P3)
**1**<br>**RANDOM UNLINKED**	**2**<br>**RANDOM UNLINKED**	**3**<br>**RANDOM UNLINKED**	**4**<br>**RANDOM LINKED**	**5**<br>**RANDOM LINKED**	**6**<br>**RANDOM UNLINKED**
Nice thing to be well.<br><br>Consider your medication schedule. It appears that things are a bit irregular right now.<br><br>Continue on...	A lot of things impact people's decisions to take medications. It's complicated.<br><br>Below are common questions people have about medications.<br><br>When it comes to medications, do you wonder about…	It is important to get all of your questions and concerns about medications answered. It is your body and your health!<br><br>Read about…	CHECK IT OUT<br><br>Health outcomes are better when there is agreement on diagnosis and treatment.<br><br>Is there agreement amongst yourself, your family, your friends, and your psychiatrist?	TALK IT OUT<br><br>Be sure to get everyone on the same page!<br><br>Talk with your psychiatrist about problems you are having managing different people's opinions about your diagnosis and treatment plan.<br><br>You need support, not controversy!	Check out the relationship between taking medications and your wellness in My Charts in the Wellness Plan.<br><br>Stay well!

Daily Review Feedback Category 21: Moderate Risk – Medication Adherence, Others Influence – Family and Friends (Choice 3.3)

Risk (P10)	Questions (S1)	Concerns (P9)	Discuss (U44)	Discuss (U37)	Toolbox (P10)
**1**<br>**RANDOM UNLINKED**	**2**<br>**RANDOM UNLINKED**	**3**<br>**RANDOM UNLINKED**	**4**<br>**RANDOM LINKED**	**5**<br>**RANDOM LINKED**	**6**<br>**RANDOM UNLINKED**
It appears you're doing well. That's great.<br><br>Remember that taking medications as planned will help you stay well.<br><br>Learn more about common barriers to taking medications…	A lot of things impact people's decisions to take medications. It's complicated.<br><br>Below are common questions people have about medications.<br><br>When it comes to medications, do you wonder about…	There are so many factors that impact people's decision to take or not take medications.<br><br>Read about…	CHECK IT OUT<br><br>How is it your relationship with your family and friends influencing you? What is going on that you don't want to take the medications as prescribed?<br><br>Sit down with your psychiatrist. Talk about your thoughts and feelings about your family and friends. Come up with a plan of action to address your concerns.	TALK IT OUT<br><br>Meet with your family and friends share your concerns. It is important that you all are on the same page.<br><br>Remember that your family and friends are there to support you. But your psychiatrist is the expert on medications and you are the expert on yourself.	Take a look at "Opinions about Medications" in Toolbox.<br><br>Stay well!

Daily Review Feedback Category 21: Moderate Risk – Medication Adherence, Others Influence – Family and Friends (Choice 3.3)

Reflect (P9)	Questions (S1)	Concerns (P8)	Prepare (U45)	Discuss (U38)	Team (P8)
**1**<br>**RANDOM UNLINKED**	**2**<br>**RANDOM UNLINKED**	**3**<br>**RANDOM UNLINKED**	**4**<br>**RANDOM LINKED**	**5**<br>**RANDOM LINKED**	**6**<br>**RANDOM UNLINKED**
It seems you are well. Good to see.<br><br>Consider how you're taking your medications. Seemed like you may be a bit off the plan.<br><br>There are many reasons things might have gotten off.<br><br>Continue to read more…<br>.	A lot of things impact people's decisions to take medications. It's complicated.<br><br>Below are common questions people have about medications.<br><br>When it comes to medications, do you wonder about…	Taking medications is no easy decision, especially when it comes to taking them for years.<br><br>Read about…	CHECK IT OUT<br><br>Prepare in advance for your next visit with whoever is negatively impacting your commitment to take medications. Review your Wellness Plan. Review the Foundations in *LiveWell*.<br><br>What would you like to see happen?<br><br>Practice communicating your concerns in preparation for your visit.	TALK IT OUT<br><br>Have an open and honest dialogue with your family and friends. Make sure you all are on the same page.<br><br>Consider bringing your family or friends to your next appointment with your psychiatrist.	Consider talking to your supports. Have an open dialogue about your thoughts and feelings about medications. You can review your team in the Wellness Plan.<br><br>Stay well!

Daily Review Feedback Category 21: Moderate Risk – Medication Adherence, Practical Problems – Expense (Choice 4.1)

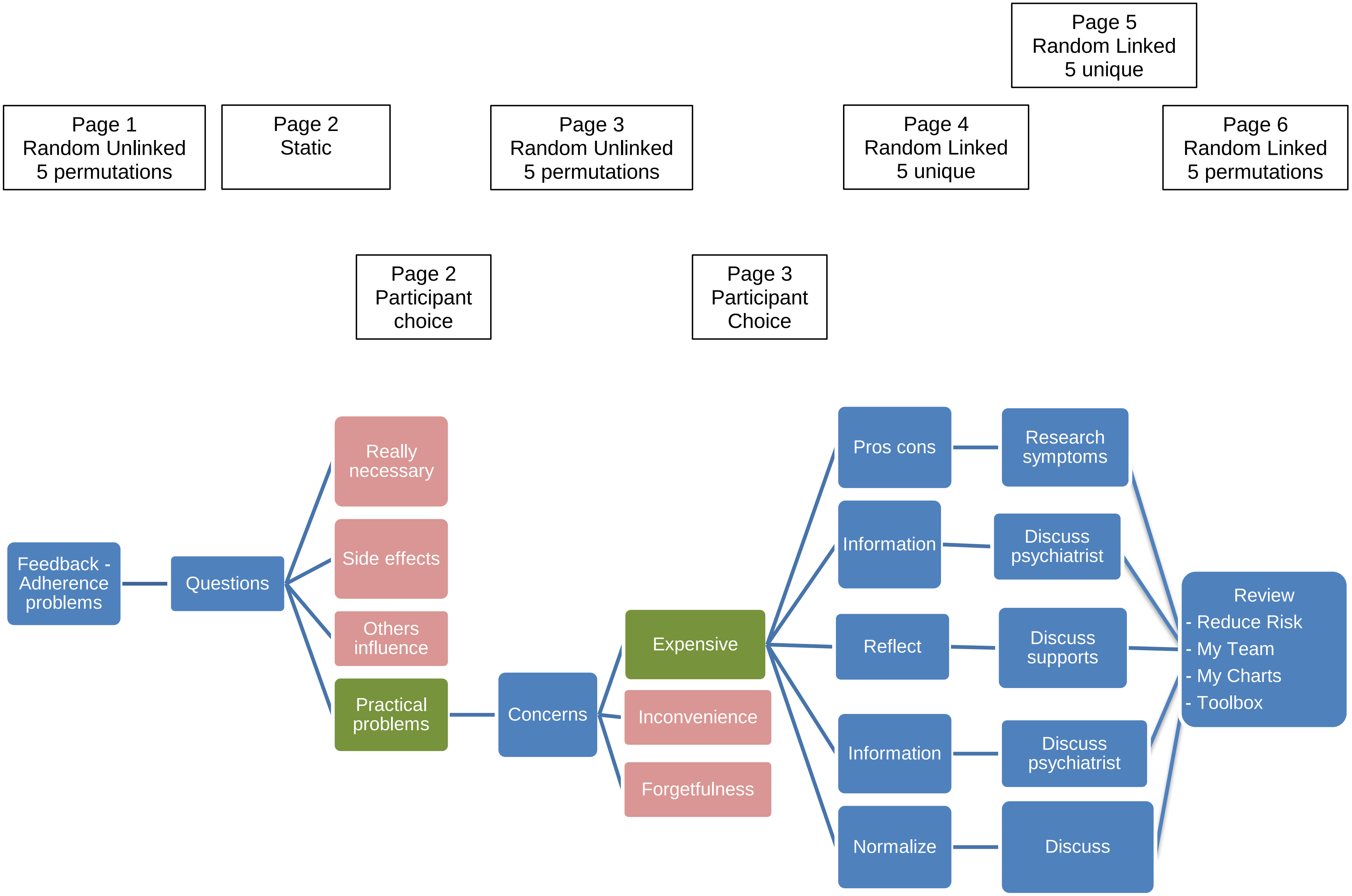

Daily Review Feedback Category 21: Moderate Risk – Medication Adherence, Practical Problems – Expensive (Choice 4.1)

Reflect (P6)	Questions (S1)	Concerns (P1)	Pros Cons (U46)	Symptoms (U1)	(P6)
**1**<br>**RANDOM UNLINKED**	**2**<br>**RANDOM UNLINKED**	**3**<br>**RANDOM UNLINKED**	**4**<br>**RANDOM LINKED**	**5**<br>**RANDOM LINKED**	**6**<br>**RANDOM UNLINKED**
Glad to see you're well. You may want to take a look at your medication schedule. It seems you may not be keeping up as planned.<br><br>Continue to learn about medications.	A lot of things impact people's decisions to take medications. It's complicated.<br><br>Below are common questions people have about medications.<br><br>When it comes to medications, do you wonder about…	Taking medications is a big decision. Most people have reservations! You're not alone.<br><br>Read about…	CHECK IT OUT<br><br>Medications are expensive.<br><br>Make sure you are on the health insurance plan that best serves your needs.<br><br>Many drug companies also provide low---cost medications for people without many financial resources.<br><br>Weigh the pros and cons of taking medications. Try to fit them into your budget. It's unfortunate but important for your wellbeing and future.	DO SOME RESEARCH<br><br>There are many good places to read more about bipolar disorder. The more you know, the more able you will be to make informed decisions.<br><br>Read about the symptoms of bipolar disorder.<br><br>• Depression & Bipolar Support Alliance<br>• National Alliance for Mental Illness<br>• National Institute of Mental Health	Be well!

Daily Review Feedback Category 21: Moderate Risk – Medication Adherence, Practical Problems – Expensive (Choice 4.1)

Reflect (P7)	Questions (S1)	Concerns (P6)	Information (U47)	Discuss (U39)	Reduce Risk (P7)
**1**<br>**RANDOM UNLINKED**	**2**<br>**RANDOM UNLINKED**	**3**<br>**RANDOM UNLINKED**	**4**<br>**RANDOM LINKED**	**5**<br>**RANDOM LINKED**	**6**<br>**RANDOM UNLINKED**
Good to see you're doing well.<br><br>You may want to take a look at your medication schedule. Taking medications regularly is one of the most important things you can do to stay well.<br><br>Continue to read more…	A lot of things impact people's decisions to take medications. It's complicated.<br><br>Below are common questions people have about medications.<br><br>When it comes to medications, do you wonder about…	Medications should make sense for you. Any questions or problems need to be addressed!<br><br>Read about…	CHECK IT OUT<br><br>Medications are expensive.<br><br>Insurance plans these days vary widely in how much they cover and what drugs they cover.<br><br>Consider this when you select your insurance carrier and plan.	DO MORE CHECKING<br><br>Also, talk to your psychiatrist. Perhaps there are generic medications you can take that are as effective but cheaper.<br><br>Your psychiatrist might have other ideas as well.	Check out your medication plan in Reduce Risk in the Wellness Plan.<br><br>Stay well!

Daily Review Feedback Category 21: Moderate Risk – Medication Adherence, Practical Problems – Expensive (Choice 4.1)

Reflect (P8)	Questions (S1)	Concerns (P7)	Reflect (U48)	Discuss (U40)	My Charts (P3)
**1**<br>**RANDOM UNLINKED**	**2**<br>**RANDOM UNLINKED**	**3**<br>**RANDOM UNLINKED**	**4**<br>**RANDOM LINKED**	**5**<br>**RANDOM LINKED**	**6**<br>**RANDOM UNLINKED**
Nice thing to be well.<br><br>Consider your medication schedule. It appears that things are a bit irregular right now.<br><br>Continue on...	A lot of things impact people's decisions to take medications. It's complicated.<br><br>Below are common questions people have about medications.<br><br>When it comes to medications, do you wonder about…	It is important to get all of your questions and concerns about medications answered. It is your body and your health!<br><br>Read about…	CHECK IT OUT<br><br>What are your options? How can you fit the expense of medications into your budget?<br><br>Are there things you might give up in order to make it easier to afford your medications?<br><br>Giving up unhealthy things might be a place to start…like alcohol, cigarettes, and junk food.<br><br>Think about it!	DO MORE CHECKING<br><br>Sit down with your supports. Talk with them about your budget concerns.<br><br>Maybe they will have ideas that you did not think of at first.<br><br>Remember your supports are there for you! This is a good reason to talk with them.	Check out the relationship between taking medications and your wellness in My Charts in the Wellness Plan.<br><br>Stay well!

Daily Review Feedback Category 21: Moderate Risk – Medication Adherence, Practical Problems – Expensive (Choice 4.1)

Risk (P10)	Questions (S1)	Concerns (P9)	Information (U49)	Discuss (U41)	Toolbox (P10)

1<br>RANDOM UNLINKED	2<br>RANDOM UNLINKED	3<br>RANDOM UNLINKED	4<br>RANDOM LINKED	5<br>RANDOM LINKED	6<br>RANDOM UNLINKED
It appears you're doing well. That's great.<br><br>Remember that taking medications as planned will help you stay well.<br><br>Learn more about common barriers to taking medications…	A lot of things impact people's decisions to take medications. It's complicated.<br><br>Below are common questions people have about medications.<br><br>When it comes to medications, do you wonder about…	There are so many factors impact people's decision to take or not take medications.<br><br>Read about…	CHECK IT OUT<br><br>If your financial resources are limited, then look into resources provided by the drug companies. Many have programs designed to help individuals with lower incomes afford their medications.<br><br>You can look on their website for information.	DO MORE CHECKING<br><br>Talk with your psychiatrist about problems you're having affording your medications.<br><br>They will know about different drug company programs for which you might qualify.	Take a look at "Opinions about Medications" in Toolbox.<br><br>Stay well!

Daily Review Feedback Category 21: Moderate Risk – Medication Adherence, Practical Problems – Expensive (Choice 4.1)

Reflect (P9)	Questions (S1)	Concerns (P8)	Normalize (U50)	Discuss (U42)	Team (P8)
**1**<br>**RANDOM UNLINKED**	**2**<br>**RANDOM UNLINKED**	**3**<br>**RANDOM UNLINKED**	**4**<br>**RANDOM LINKED**	**5**<br>**RANDOM LINKED**	**6**<br>**RANDOM UNLINKED**
It seems you are well. Good to see.<br><br>Consider how you're taking your medications. Seemed like you may be a bit off the plan.<br><br>There are many reasons things might have gotten off.<br><br>Continue to read more…<br>.	A lot of things impact people's decisions to take medications. It's complicated.<br><br>Below are common questions people have about medications.<br><br>When it comes to medications, do you wonder about…	Taking medications is no easy decision, especially when it comes to taking them for years.<br><br>Read about…	CHECK IT OUT<br><br>You're not alone. Many people find the expense of medications to be a nuisance at best and prohibitive at worst.<br><br>Make sure you are on the health insurance plan that best serves your needs. Plans vary widely in what they cover and how much they cover.<br><br>Many drug companies also provide low---cost medications for people without many financial resources.	DO MORE CHECKING<br><br>Discuss your concerns with your psychiatrist. They may have some ideas that will help.<br><br>Also consider talking with your supports. Perhaps they have some creative ideas about affording medications.	Consider talking to your supports. Have an open dialogue about your thoughts and feelings about medications. You can review your team in the Wellness Plan.<br><br>Stay well!

Daily Review Feedback Category 21: Moderate Risk – Medication Adherence, Practical Problems – Inconvenient (Choice 4.2)

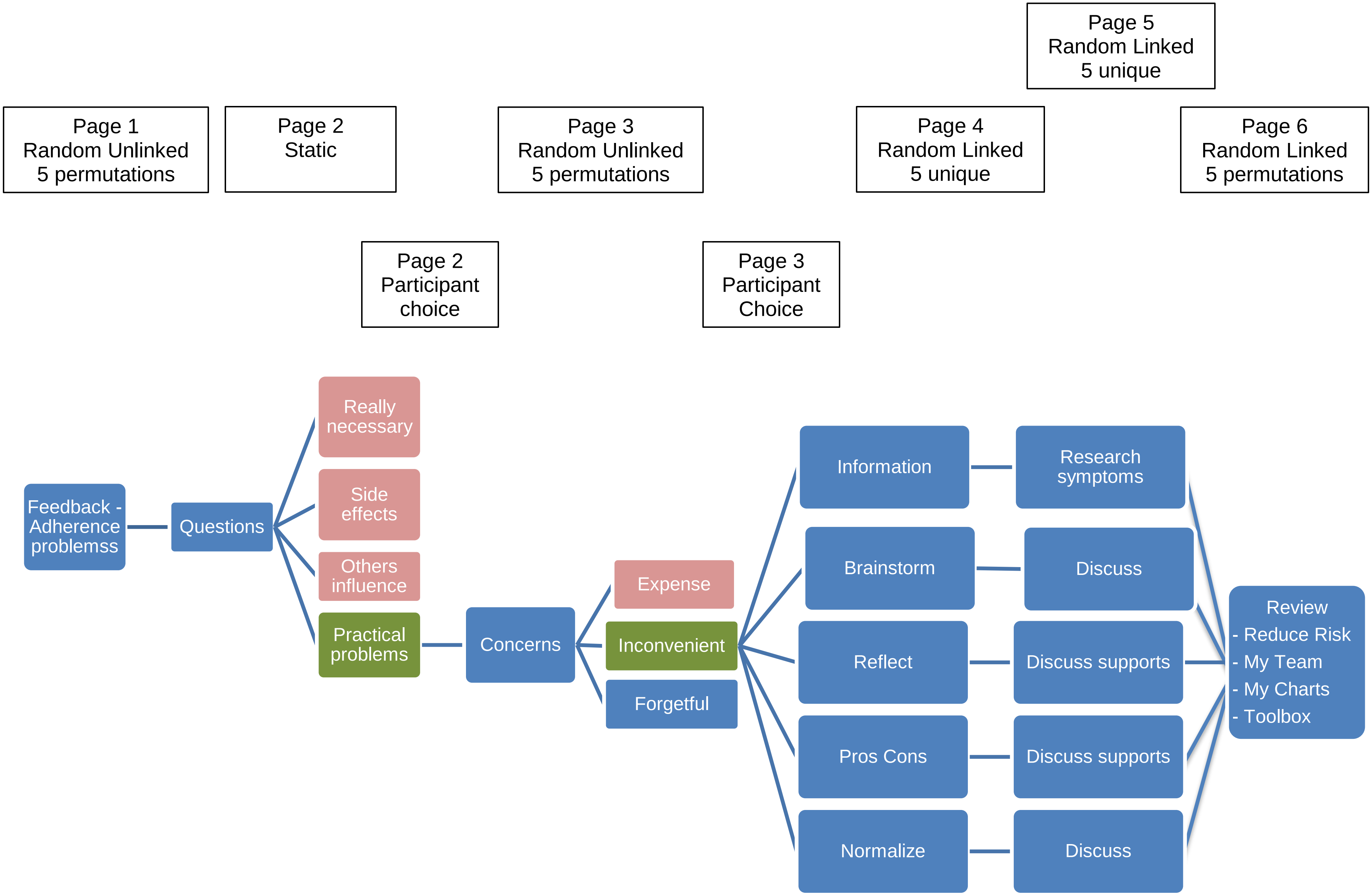

Daily Review Feedback Category 21: Moderate Risk – Medication Adherence, Practical Problems – Inconvenient (Choice 4.2)

Adherence (P6)	Questions (S1)	Concerns (P1)	Information (U51)	Symptoms (U1)	(P6)
**1**<br>**RANDOM UNLINKED**	**2**<br>**RANDOM UNLINKED**	**3**<br>**RANDOM UNLINKED**	**4**<br>**RANDOM LINKED**	**5**<br>**RANDOM LINKED**	**6**<br>**RANDOM UNLINKED**
Glad to see you're well. You may want to take a look at your medication schedule. It seems you may not be keeping up as planned.<br><br>Continue to learn about medications.	A lot of things impact people's decisions to take medications. It's complicated.<br><br>Below are common questions people have about medications.<br><br>When it comes to medications, do you wonder about…	Taking medications is a big decision. Most people have reservations! You're not alone.<br><br>Read about…	CHECK IT OUT<br><br>Taking medications, getting blood tests, and visiting your doctor are inconvenient.<br>There is no arguing that point!<br><br>Try to arrange these things in ways that are least disruptive.<br><br>Remember that self---care always requires time and effort, no matter what it is!	DO SOME RESEARCH<br><br>There are many good places to read more about bipolar disorder. The more you know, the more able you will be to make informed decisions.<br><br>Read about the symptoms of bipolar disorder.<br><br>• Depression & Bipolar Support Alliance<br>• National Alliance for Mental Illness<br>• National Institute of Mental Health	Be well!

Daily Review Feedback Category 21: Moderate Risk – Medication Adherence, Practical Problems – Inconvenient (Choice 4.2)

Adherence (P7)	Questions (S1)	Concerns (P6)	Brainstorm (U52)	Discuss (U43)	Reduce Risk (P7)
**1**<br>**RANDOM UNLINKED**	**2**<br>**RANDOM UNLINKED**	**3**<br>**RANDOM UNLINKED**	**4**<br>**RANDOM LINKED**	**5**<br>**RANDOM LINKED**	**6**<br>**RANDOM UNLINKED**
Good to see you're doing well.<br><br>You may want to take a look at your medication schedule. Taking medications regularly is one of the most important things you can do to stay well.<br><br>Continue to read more…	A lot of things impact people's decisions to take medications. It's complicated.<br><br>Below are common questions people have about medications.<br><br>When it comes to medications, do you wonder about…	Medications should make sense for you. Any questions or problems need to be addressed!<br><br>Read about…	CHECK IT OUT<br><br>Taking medications can be inconvenient.<br><br>You have to keep up getting the prescriptions renewed, remember to take them multiple times a day, and think ahead whenever you're not going to be at home. Maybe you have to take the medications with food.<br><br>If these or other things are getting in the way for you, sit down and brainstorm. What can you do to make it easier?	DO MORE CHECKING<br><br>Talk with your supports or psychiatrist. Maybe they have some creative solutions for you.	Check out your medication plan in Reduce Risk in the Wellness Plan.<br><br>Stay well!

Daily Review Feedback Category 21: Moderate Risk – Medication Adherence, Practical Problems – Inconvenient (Choice 4.2)

Adherence (P8)	Questions (S1)	Concerns (P7)	Reflect (U53)	Discuss (U44)	My Charts (P3)
**1**<br>**RANDOM UNLINKED**	**2**<br>**RANDOM UNLINKED**	**3**<br>**RANDOM UNLINKED**	**4**<br>**RANDOM LINKED**	**5**<br>**RANDOM LINKED**	**6**<br>**RANDOM UNLINKED**
Nice thing to be well.<br><br>Consider your medication schedule. It appears that things are a bit irregular right now.<br><br>Continue on...	A lot of things impact people's decisions to take medications. It's complicated.<br><br>Below are common questions people have about medications.<br><br>When it comes to medications, do you wonder about…	It is important to get all of your questions and concerns about medications answered. It is your body and your health!<br><br>Read about…	CHECK IT OUT<br><br>What are your options? How can you work around the inconvenience of taking medications?<br><br>Are there things you need in order to make it easier to take your medications?<br><br>Think about it!	DO MORE CHECKING<br><br>Sit down with your supports. Talk with them about your concerns.<br><br>Maybe they will have ideas that you did not think of at first.<br><br>Remember your supports are there for you! This is a good reason to talk with them.	Check out the relationship between taking medications and your wellness in My Charts in the Wellness Plan.<br><br>Stay well!

Daily Review Feedback Category 21: Moderate Risk – Medication Adherence, Practical Problems – Inconvenient (Choice 4.2)

Adherence (P9)	Questions (S1)	Concerns (P9)	Pros Cons (U54)	Discuss (U45)	Toolbox (P10)

1<br>RANDOM UNLINKED	2<br>RANDOM UNLINKED	3<br>RANDOM UNLINKED	4<br>RANDOM LINKED	5<br>RANDOM LINKED	6<br>RANDOM UNLINKED
It appears you're doing well. That's great.<br><br>Remember that taking medications as planned will help you stay well.<br><br>Learn more about common barriers to taking medications…	A lot of things impact people's decisions to take medications. It's complicated.<br><br>Below are common questions people have about medications.<br><br>When it comes to medications, do you wonder about…	There are so many factors that impact people's decision to take or not take medications.<br><br>Read about…	CHECK IT OUT<br><br>Think about the advantages of taking medications. Does the inconvenience outweigh the potential advantages?<br><br>Think about ways to make taking medications easier. If there is no way around the inconvenience, consider accepting the burden for the sake of your health.	DO MORE CHECKING<br><br>Sit down and talk with your supports. Share your thoughts and feelings about medications. Talk about how they are inconvenient.<br><br>See what they have to say!	Take a look at "Opinions about Medications" in Toolbox.<br><br>Stay well!

Daily Review Feedback Category 21: Moderate Risk – Medication Adherence, Practical Problems – Inconvenient (Choice 4.2)

Adherence (P10)	Questions (S1)	Concerns (P8)	Normalize (U55)	Discuss (U46)	Team (P8)
**1**<br>**RANDOM UNLINKED**	**2**<br>**RANDOM UNLINKED**	**3**<br>**RANDOM UNLINKED**	**4**<br>**RANDOM LINKED**	**5**<br>**RANDOM LINKED**	**6**<br>**RANDOM UNLINKED**
It seems you are well. Good to see.<br><br>Consider how you're taking your medications. Seemed like you may be a bit off the plan.<br><br>There are many reasons things might have gotten off.<br><br>Continue to read more…<br>.	A lot of things impact people's decisions to take medications. It's complicated.<br><br>Below are common questions people have about medications.<br><br>When it comes to medications, do you wonder about…	Taking medications is no easy decision, especially when it comes to taking them for years.<br><br>Read about…	CHECK IT OUT<br><br>You're not alone. Many people find taking medications to be a nuisance.<br><br>Make sure you are doing all you can to make it as easy as possible.	DO MORE CHECKING<br><br>Discuss your concerns with your psychiatrist. They may have some ideas that will help.<br><br>Also consider talking with your supports. Perhaps they have some creative ideas about taking medications.	Consider talking to your supports. Have an open dialogue about your thoughts and feelings about medications. You can review your team in the Wellness Plan.<br><br>Stay well!

Daily Review Feedback Category 21: Moderate Risk – Medication Adherence, Practical Problems – Forgetful (Choice 4.3)

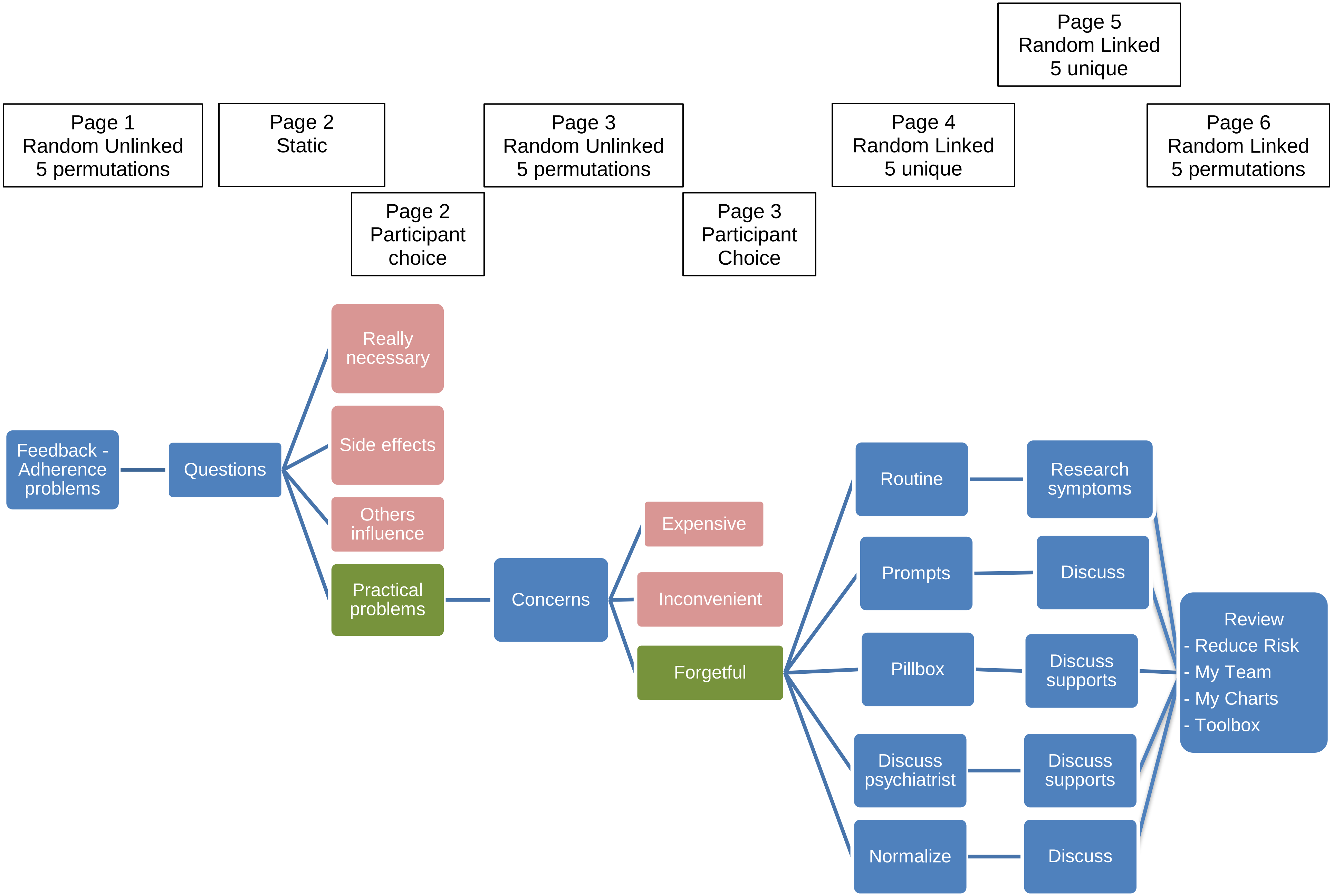

Daily Review Feedback Category 21: Moderate Risk – Medication Adherence, Practical Problems – Forgetful (Choice 4.3)

Adherence (P6)	Questions (S1)	Concerns (P1)	Routine (U56)	Symptoms (U1)	(P6)
**1**<br>**RANDOM UNLINKED**	**2**<br>**RANDOM UNLINKED**	**3**<br>**RANDOM UNLINKED**	**4**<br>**RANDOM LINKED**	**5**<br>**RANDOM LINKED**	**6**<br>**RANDOM UNLINKED**
Glad to see you're well. You may want to take a look at your medication schedule. It seems you may not be keeping up as planned.<br><br>Continue to learn about medications.	A lot of things impact people's decisions to take medications. It's complicated.<br><br>Below are common questions people have about medications.<br><br>When it comes to medications, do you wonder about…	Taking medications is a big decision. Most people have reservations! You're not alone.<br><br>Read about…	CHECK IT OUT<br><br>Try the following tips:<br><br>• Take your medicine at the same time every day<br>• Keep your medicine in the same place<br>• Put our medicine next to something that you do or see every day such as your toothbrush, coffee pot, or alarm clock<br>• Set a medicine alarm<br>• Keep a pill box	DO SOME RESEARCH<br><br>There are many good places to read more about bipolar disorder. The more you know, the more able you will be to make informed decisions.<br><br>Read about the symptoms of bipolar disorder.<br><br>• Depression & Bipolar Support Alliance<br>• National Alliance for Mental Illness<br>• National Institute of Mental Health	Be well!

Daily Review Feedback Category 21: Moderate Risk – Medication Adherence, Practical Problems – Forgetful (Choice 4.3)

Adherence (P7)	Questions (S1)	Concerns (P6)	Prompts (U57)	Discuss (U43)	Reduce Risk (P7)
**1**<br>**RANDOM UNLINKED**	**2**<br>**RANDOM UNLINKED**	**3**<br>**RANDOM UNLINKED**	**4**<br>**RANDOM LINKED**	**5**<br>**RANDOM LINKED**	**6**<br>**RANDOM UNLINKED**
Good to see you're doing well.<br><br>You may want to take a look at your medication schedule. Taking medications regularly is one of the most important things you can do to stay well.<br><br>Continue to read more…	A lot of things impact people's decisions to take medications. It's complicated.<br><br>Below are common questions people have about medications.<br><br>When it comes to medications, do you wonder about…	Medications should make sense for you. Any questions or problems need to be addressed!<br><br>Read about…	CHECK IT OUT<br><br>Set up some sort of cue or prompt for taking medications.<br><br>Setting an alarm, posting a note, taking medications with meals, leaving pill bottles out, or even a pillbox left in plain sight can be helpful.	DO MORE CHECKING<br><br>Talk with your supports or psychiatrist. Maybe they have some creative solutions for you.	Check out your medication plan in Reduce Risk in the Wellness Plan.<br><br>Stay well!

Daily Review Feedback Category 21: Moderate Risk – Medication Adherence, Practical Problems – Forgetful (Choice 4.3)

Adherence (P8)	Questions (S1)	Concerns (P7)	Pillbox (U58)	Discuss (U44)	My Charts (P3)
**1**<br>**RANDOM UNLINKED**	**2**<br>**RANDOM UNLINKED**	**3**<br>**RANDOM UNLINKED**	**4**<br>**RANDOM LINKED**	**5**<br>**RANDOM LINKED**	**6**<br>**RANDOM UNLINKED**
Nice thing to be well.<br><br>Consider your medication schedule. It appears that things are a bit irregular right now.<br><br>Continue on...	A lot of things impact people's decisions to take medications. It's complicated.<br><br>Below are common questions people have about medications.<br><br>When it comes to medications, do you wonder about…	It is important to get all of your questions and concerns about medications answered. It is your body and your health!<br><br>Read about…	CHECK IT OUT<br><br>If you keep forgetting, then forget relying on your memory!<br><br>Make it easy for yourself. Get a pillbox and put it out somewhere you will see it morning and night. Is this in the bathroom? Bedroom? Kitchen?<br><br>Make a plan.	DO MORE CHECKING<br><br>Sit down with your supports. Talk with them about your challenge.<br><br>Maybe they will have ideas that you did not think of at first.<br><br>Remember your supports are there for you! This is a good reason to talk with them.	Check out the relationship between taking medications and your wellness in My Charts in the Wellness Plan.<br><br>Stay well!

Daily Review Feedback Category 21: Moderate Risk – Medication Adherence, Practical Problems – Forgetful (Choice 4.3)

Adherence (P10)	Questions (S1)	Concerns (P9)	Discuss (U59)	Discuss (U47)	Toolbox (P10)
**1**<br>**RANDOM UNLINKED**	**2**<br>**RANDOM UNLINKED**	**3**<br>**RANDOM UNLINKED**	**4**<br>**RANDOM LINKED**	**5**<br>**RANDOM LINKED**	**6**<br>**RANDOM UNLINKED**
It appears you're doing well. That's great.<br><br>Remember that taking medications as planned will help you stay well.<br><br>Learn more about common barriers to taking medications…	A lot of things impact people's decisions to take medications. It's complicated.<br><br>Below are common questions people have about medications.<br><br>When it comes to medications, do you wonder about…	There are so many factors impact people's decision to take or not take medications.<br><br>Read about…	CHECK IT OUT<br><br>Talk with your psychiatrist. See if you can't simplify your medication regimen.<br><br>Taking medications 3 or 4 times a day can be tricky. Sometimes it is possible to get the plan streamlined down to once or twice a day.	DO MORE CHECKING<br><br>Sit down and talk with your supports. Share your thoughts and feelings about medications. Talk about how it is hard to remember to take them.<br><br>See what they have to say!	Take a look at "Opinions about Medications" in Toolbox.<br><br>Stay well!

Daily Review Feedback Category 21: Moderate Risk – Medication Adherence, Practical Problems – Forgetful (Choice 4.3)

Adherence (P9)	Questions (S1)	Concerns (P8)	Normalize (U60)	Discuss (U48)	Team (P8)
**1**<br>**RANDOM UNLINKED**	**2**<br>**RANDOM UNLINKED**	**3**<br>**RANDOM UNLINKED**	**4**<br>**RANDOM LINKED**	**5**<br>**RANDOM LINKED**	**6**<br>**RANDOM UNLINKED**
It seems you are well. Good to see.<br><br>Consider how you're taking your medications. Seemed like you may be a bit off the plan.<br><br>There are many reasons things might have gotten off.<br><br>Continue to read more…<br>.	A lot of things impact people's decisions to take medications. It's complicated.<br><br>Below are common questions people have about medications.<br><br>When it comes to medications, do you wonder about…	Taking medications is no easy decision, especially when it comes to taking them for years.<br><br>Read about…	CHECK IT OUT<br><br>You're not alone. Many people find it hard to remember to take their medications.<br><br>Pair taking medications with something you never forget to do, like eat or brush your teeth, or wash your face.	DO MORE CHECKING<br><br>Discuss your challenges with your psychiatrist. They may have some ideas that will help.<br><br>Also consider talking with your supports. Perhaps they have some creative ideas about remembering to take medications.	Consider talking to your supports. Have an open dialogue about your thoughts and feelings about medications. You can review your team in the Wellness Plan.<br><br>Stay well!

Daily Review Feedback Category 22: Moderate Risk – Sleeping Too Little, Good Sleep Habits (Choice 1.0)

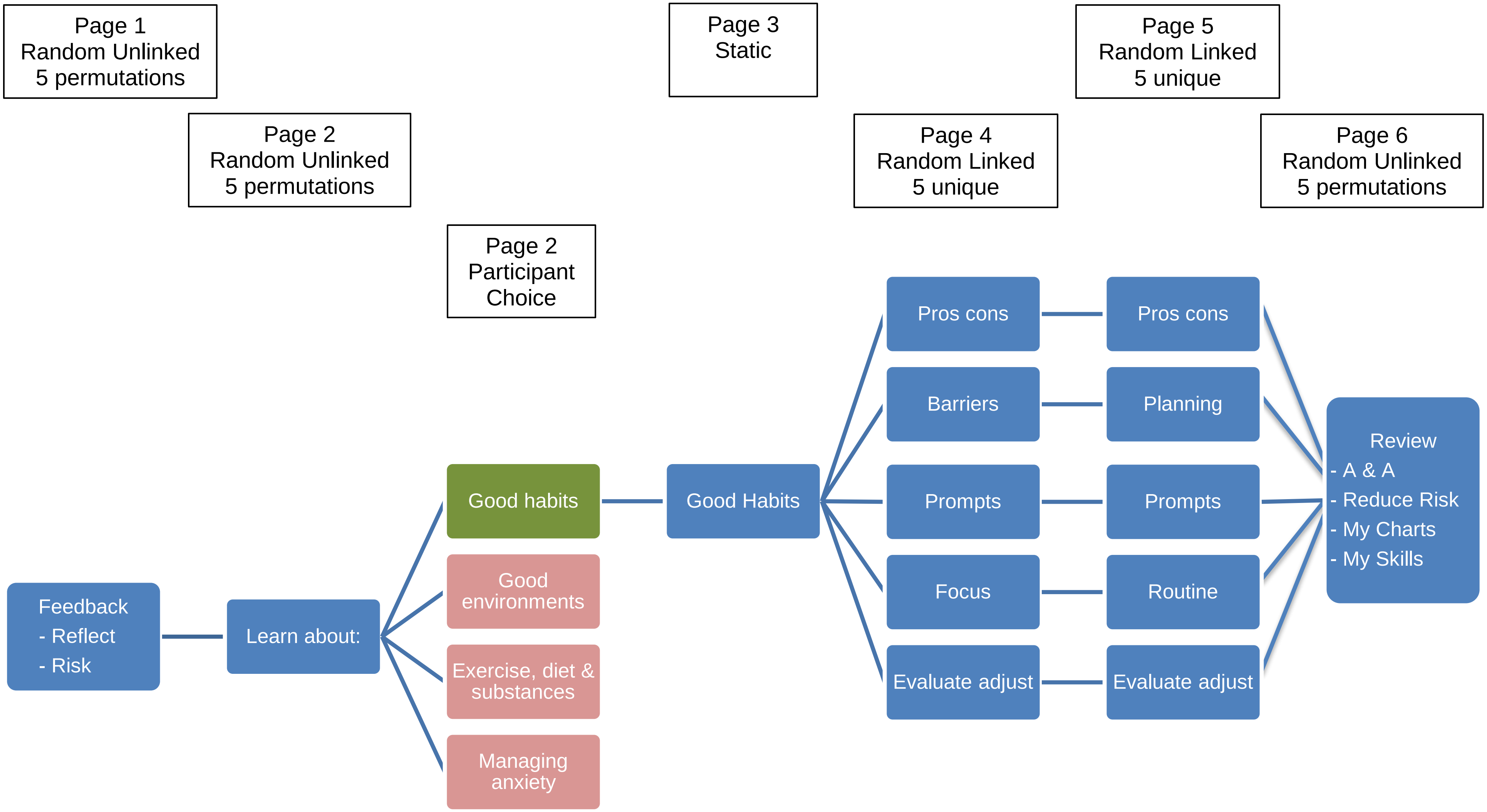

Daily Review Feedback Category 22: Moderate Risk – Sleeping Too Little, Good Sleep Habits (Choice 1.0)

Reflect (P1)	Barriers (P1)	Habits (S1)	Pros Cons (U1)	Pros Cons (U1)	My Charts (P1)
**1<br>RANDOM UNLINKED**	**2<br>RANDOM UNLINKED**	**3<br>STATIC**	**4<br>RANDOM LINKED**	**5<br>RANDOM LINKED**	**6<br>RANDOM UNLINKED**
Glad to see you're well.<br><br>You may, however, want to take a look at your sleep. It seems as though you have been sleeping less than usual the past few nights.<br><br>Continue to learn more about sleeping well.	A lot of things can get in the way of getting adequate sleep. Some details might surprise you!<br><br>Read about…	Good sleep habits include the following:<br><br>• Turn in the same time each night<br>• Arise at the same time each morning<br>• Use your bed for sleep and sex only<br>• Avoid caffeine, alcohol, and drugs<br>• Avoid daytime napping<br><br>Following these guidelines is easier said than done.<br><br>Continue for tips …	KNOW YOUR COSTS AND BENEFITS<br><br>Define the prize! Think about or even write down what you will gain by getting more sleep.<br><br>What will you have to give up if you get more sleep?<br><br>Knowing your own pros and cons for getting more sleep can help you get motivated to make a change.<br><br>Unless the pros outweigh the cons for them, most people find it hard to make and maintain a change, like getting more sleep.	EXAMPLE<br><br>Say you're turning in at 2:00 am every night and have to get up at 7:00 a.m. for work. Five hours is not enough rest.<br><br>Pros of turning in earlier:<br>• Feel better rested<br>• Have more patience, be less irritable<br>• More productive at work<br><br>Cons of turning in earlier:<br>• Would have no alone time at home<br>• Would miss late night television<br><br>Your decision might be to turn in early but arrange some way to get quiet time earlier in the evening.	Check out the relationship between your sleep and wellness in My Charts in the Wellness Plan below.<br><br>Be well!

Daily Review Feedback Category 22: Moderate Risk – Sleeping Too Little, Good Sleep Habits (Choice 1.0)

Reflect (P2)	Routine (P2)	Habits (S1)	Barriers (U2)	Planning (U2)	Reduce Risk (P2)
**1**<br>**RANDOM UNLINKED**	**2**<br>**RANDOM UNLINKED**	**3**<br>**STATIC**	**4**<br>**RANDOM LINKED**	**5**<br>**RANDOM LINKED**	**6**<br>**RANDOM UNLINKED**
Your wellness rating suggests that you are doing well.<br><br>Even so, take a few minutes now to consider your sleep schedule. It appears that you may not be getting enough rest.<br><br>Continue to learn more...	Doesn't it seem like balance is always the key? Activity and rest, fun and work…<br><br>Look to get a good, balanced routine. Look to get more sleep.<br><br>Read about…	Good sleep habits include the following:<br><br>• Turn in the same time each night<br>• Arise the same time each morning<br>• Use your bed for sleep and sex only<br>• Avoid caffeine, alcohol, and drugs<br>• Avoid daytime napping<br><br>Following these guidelines is easier said than done.<br><br>Continue for tips …	ANTICIPATING OBSTACLES<br><br>Whether you like it or not, getting enough sleep is vital to your health. Just like getting enough water and nutrition is vital to health.<br><br>Set a realistic sleep schedule. Then consider things that might get in the way of sticking with your schedule.<br><br>• Do you sometimes want to do other things like watch movies or go out with friends?<br>• Do family or work obligations interfere with your sleep?<br><br>What can you do to overcome these obstacles?	EXAMPLE<br><br>Consider recording your favorite late---night television shows so that you can watch them earlier in the evening.<br><br>Consider planning time with friends on the weekend days or earlier in the evenings.<br><br>Take into account family and work obligations when setting up your sleep schedule. Don't set yourself up for failure!	Check out your sleep plan in Reduce Risk in the Wellness Plan.<br><br>Stay well…

Daily Review Feedback Category 22: Moderate Risk – Sleeping Too Little, Good Sleep Habits (Choice 1.0)

Risk (P3)	Risk (P3)	Habits (S1)	Prompts (U3)	Prompts (U3)	A & A (P3)
**1**<br>**RANDOM UNLINKED**	**2**<br>**RANDOM UNLINKED**	**3**<br>**STATIC**	**4**<br>**RANDOM LINKED**	**5**<br>**RANDOM LINKED**	**6**<br>**RANDOM UNLINKED**
You're rating yourself as doing well, which is great.<br><br>However, it seems as though lately, you may not be getting as much sleep as you need.<br><br>Getting proper rest is essential for maintaining a healthy lifestyle. Not getting enough sleep puts you at risk for developing symptoms.<br><br>Continue to learn more.	You have been getting less sleep than you said you need lately.<br><br>This puts you at risk for problems with attention, concentration, memory, and managing stress. It also puts you at risk for symptoms.<br><br>In order to get better sleep, read about…	Good sleep habits include the following:<br><br>• Turn in the same time each night<br>• Arise the same time each morning<br>• Use your bed for sleep and sex only<br>• Avoid caffeine, alcohol, and drugs<br>• Avoid daytime napping<br><br>Following these guidelines is easier said than done.<br><br>Continue for tips …	USING CUES<br><br>Develop a bedtime routine that starts about 1 hour before you plan on drifting off.<br><br>Use cues to remind yourself when to transition into your bedtime routine. Perhaps set an alarm or post a note. Even if you are good at keeping track of time, cues can help you stay on schedule.<br><br>Use cues to remind your body that it is time to settle down. Turn the lights down lower. Engage in low---stimulation activities like watching television or reading.	TIP OF THE DAY<br><br>Cues for bedtime:<br>• Alarm clocks<br>• Phone reminders<br>• Post---it notes in the kitchen<br><br>Cues for your body:<br>• Lower lights<br>• Put away electronic devices<br>• Engage in restful activities<br><br>If you hope to fall asleep at 11:00 p.m., start to transition around 10:00 p.m. Stop doing chores or socializing or exercising. Instead turn the lights lower and read a book.	Double check for early warning signs of mania and depression in Awareness and Action in the Wellness Plan.<br><br>Sleep disruptions can be a sign of illness.<br><br>Stay well…

Daily Review Feedback Category 22: Moderate Risk – Sleeping Too Little, Good Sleep Habits (Choice 1.0)

Reflect (P4)	Risk (P4)	Habits (S1)	Focus (U4)	Routine (U4)	My Skills (P4)
1<br>**RANDOM UNLINKED**	2<br>**RANDOM UNLINKED**	3<br>**STATIC**	4<br>**RANDOM LINKED**	5<br>**RANDOM LINKED**	6<br>**RANDOM UNLINKED**
Seems like you're doing well. That's good.<br><br>To stay well, consider getting more consistent sleep each night.<br><br>Continue to read more...	Getting enough sleep can feel optional at times. The reality is that even getting 30 minutes less sleep than you need can trigger symptoms!<br><br>Sleep better.<br><br>Read about...	Good sleep habits include the following:<br><br>• Turn in the same time each night<br>• Arise the same time each morning<br>• Use your bed for sleep and sex only<br>• Avoid caffeine, alcohol, and drugs<br>• Avoid daytime napping<br><br>Following these guidelines is easier said than done.<br><br>Continue for tips ...	STAYING FOCUSED<br><br>Once bedtime arrives, stay focused. Gently let go of any:<br><br>• Intrusive thoughts<br>• Distractions<br>• Competing impulses<br>• Temptations<br>• Thoughts of old habits<br><br>Make your bedtime non--- negotiable. Whatever you are working on or wanting to do can undoubtedly wait until the next day!	TIP OF THE DAY<br><br>Stopping all chores or socializing or exercising one hour before bedtime can help you stay focused.<br><br>It gives you some transition time and makes it easier to turn in.<br><br>Try making no exceptions (except true emergencies) in terms of turning in at night.<br><br>Notice any urges to push your bedtime back and gently let them go.	Check out any tips on sleep you saved in My Skills in My Resources within the Wellness Plan.<br><br>Stay well...

Daily Review Feedback Category 22: Moderate Risk – Sleeping Too Little, Good Sleep Habits (Choice 1.0)

Reflect Risk (P5)	Action (P5)	Habits (S1)	Evaluate Adjust (U5)	Evaluate Adjust (U5)	(P5)
**1**<br>**RANDOM UNLINKED**	**2**<br>**RANDOM UNLINKED**	**3**<br>**STATIC**	**4**<br>**RANDOM LINKED**	**5**<br>**RANDOM LINKED**	**6**<br>**RANDOM UNLINKED**
It's great to see that you're doing well.<br><br>However, you may want to take a look at your sleep patterns. It seems as though you have not been getting enough sleep the past couple of nights. Inadequate sleep puts you at an increased risk of illness, so take action now.<br><br>Continue to learn more.	Next to taking medications, getting adequate sleep is one of the best things you can do in order to manage bipolar disorder.<br><br>Read about…	Good sleep habits include the following:<br><br>• Turn in the same time each night<br>• Arise the same time each morning<br>• Use your bed for sleep and sex only<br>• Avoid caffeine, alcohol, and drugs<br>• Avoid daytime napping<br><br>Following these guidelines is easier said than done.<br><br>Continue for tips …	EVALUATE OUTCOMES<br><br>Make a realistic sleep schedule, anticipate and plan around any potential obstacles. Try it out for a few nights.<br><br>Evaluate your effort. Evaluate the outcome. How did things go?<br><br>If you succeeded in turning in at your scheduled time, notice the link between your efforts and the outcome. What helped you succeed?<br><br>If you were unable to stick to the schedule, take the opportunity to learn. Were you not motivated? Was the plan unrealistic? Was there something that got in the way of sticking to the plan?	EXAMPLE<br><br>Say you make a plan to turn in at 11:00 p.m. every night and get up at 8:00 a.m. every morning.<br><br>You try for a few days and see that you're not getting into bed before 2:00 a.m.<br><br>Don't judge yourself! Try to understand what is going on. First consider motivation. Do the advantages of turning in early for you outweigh the disadvantages?<br><br>If so, did your plan make sense? If you haven't been turning in until after 2:00 a.m. for months, it is not realistic to expect yourself to turn in 3 hours earlier. Set a more modest goal.	Stay well…

Daily Review Feedback Category 22: Moderate Risk – Sleeping Too Little, Good Sleep Environments (Choice 2.0)

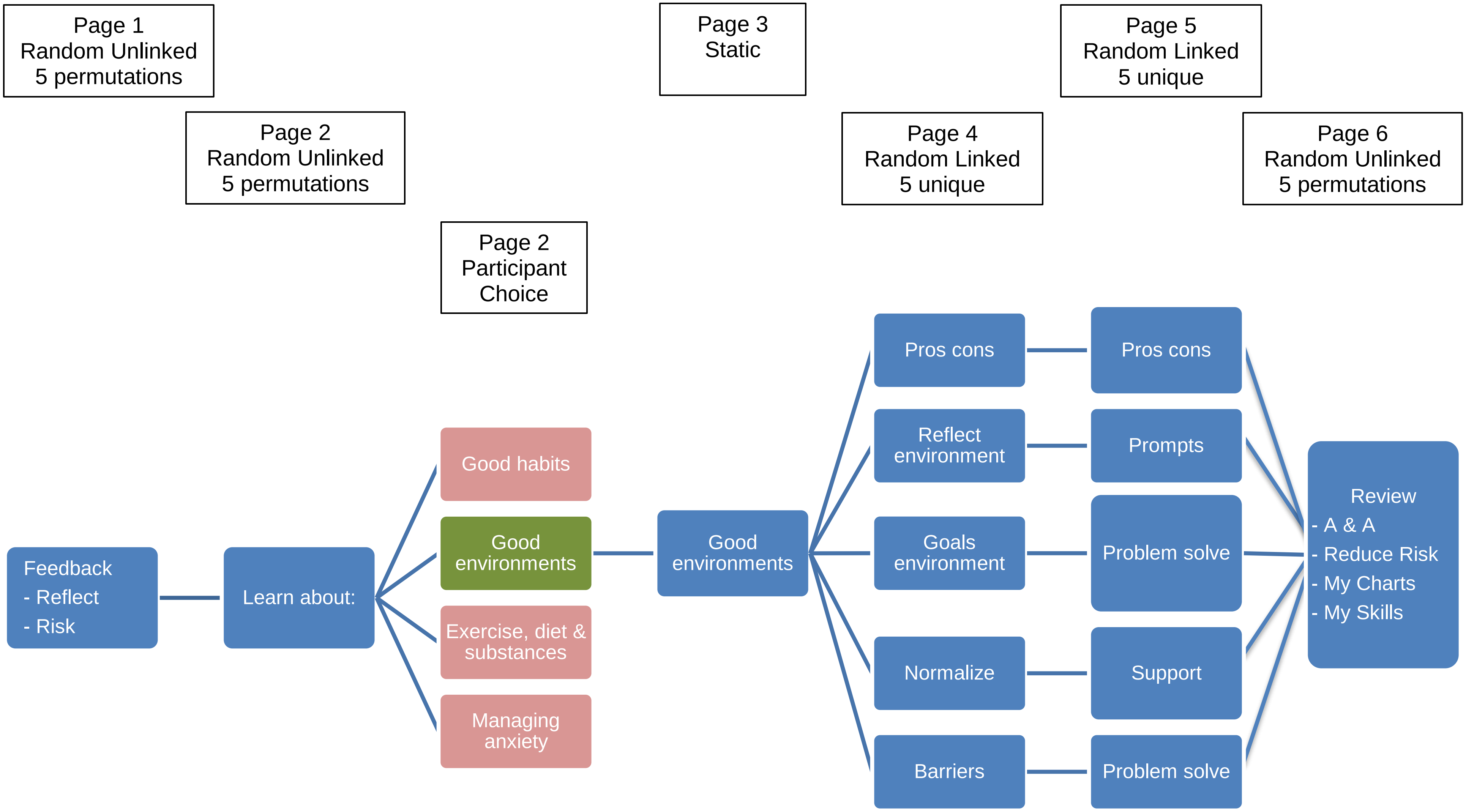

Daily Review Feedback Category 22: Moderate Risk – Sleeping Too Little, Good Sleep Environments (Choice 2.0)

Reflect (P6)	Barriers (P1)	Environment (S2)	Pros cons (U1)	Pros cons (U6)	My Charts (P1)
**1**<br>**RANDOM UNLINKED**	**2**<br>**RANDOM UNLINKED**	**3**<br>**STATIC**	**4**<br>**RANDOM LINKED**	**5**<br>**RANDOM LINKED**	**6**<br>**RANDOM UNLINKED**
Glad to see you're doing well.<br><br>You may, however, want to take a look at your sleep patterns. It seems as though you've been getting too little sleep for the past few nights.<br><br>Continue to learn more about sleeping well.	A lot of things can get in the way of getting adequate sleep. Some details might surprise you!<br><br>Read about…	Good sleep environments include:<br><br>• A comfortable mattress<br>• A comfortable temperature<br>• A quiet partner (if you have one)<br>• A quiet bedroom<br>• No television<br>• No radio<br>• No computer<br>• No smartphone<br>• No desk<br><br>Your bedroom should be a restful space, and it should be reserved for rest. It helps "train" your body to sleep regularly and deeply.<br><br>Continue for tips …	KNOW YOUR COSTS AND BENEFITS<br><br>Define the prize! Think about or even write down what you will gain by getting more sleep.<br><br>What will you have to give up if you get more sleep?<br><br>Knowing your own pros and cons for getting more sleep can help you get motivated to make a change.<br><br>Unless the pros outweigh the cons for them, most people find it hard to make and maintain a change, like getting more sleep.	EXAMPLE<br><br>Say you read the day's news on your smartphone in bed. Maybe you don't like lying still with nothing to do.<br><br>Pros of lying down without gadgets:<br>• Feel better rested<br>• Have more patience, be less irritable<br>• More productive at work<br><br>Cons of lying own without gadgets:<br>• Anxious about falling asleep<br>• Mind wanders to life problems<br><br>In this example, you could do guided imagery (imagine soothing scenes) instead of surfing the internet.	Check out the relationship between your sleep and wellness in My Charts in the Wellness Plan below.<br><br>Be well!

Daily Review Feedback Category 22: Moderate Risk – Sleeping Too Little, Good Sleep Environments (Choice 2.0)

Reflect (P7)	Normalize (P6)	Environment (S2)	Reflect (U6)	Prompts (U7)	Reduce Risk (P6)
**1**<br>**RANDOM UNLINKED**	**2**<br>**RANDOM UNLINKED**	**3**<br>**STATIC**	**4**<br>**RANDOM LINKED**	**5**<br>**RANDOM LINKED**	**6**<br>**RANDOM UNLINKED**
You are not reporting any symptoms, which is great.<br><br>However, you may want to take some now to review your sleep schedule. It seems as though you are getting too little sleep.<br><br>Continue to learn more...	You're not alone! Getting good sleep can be difficult. Maybe it's just that you want to do other things. Maybe it's that you cannot seem to sleep well despite all your efforts.<br><br>Read about…	Good sleep environments include:<br><br>• A comfortable mattress<br>• A comfortable temperature<br>• A quiet partner (if you have one)<br>• A quiet bedroom<br>• No television<br>• No radio<br>• No computer<br>• No smartphone<br>• No desk<br><br>Your bedroom should be a restful space, and it should be reserved for rest. It helps "train" your body to sleep regularly and deeply.<br><br>Continue for tips …	THINK ABOUT IT<br><br>There are two important considerations when it comes to sleep environments.<br><br>The first is comfort. This is about your five senses:<br><br>• Sight<br>• Sound<br>• Taste<br>• Touch<br>• Smell<br><br>If there is anything at all in your environment that stimulates your senses, you will not sleep well.<br><br>Lights in the room, noise from outside or a snoring partner, or even an old mattress can keep you up!	KEEP THINKING<br><br>The second is cuing. Your body automatically responds to aspects of your environment.<br><br>Certainly you have smelled good food and found yourself salivating.<br><br>Same thing with sleep. Restrict your bedroom to one activity (for the most part), and that is sleep.<br><br>The sight, sound, feel, and smell of the room will eventually trigger a readiness to sleep in your body.	Take a look at your sleep plan in Reduce Risk in the Wellness Plan.<br><br>Remember that sleep is an important part of staying well.<br><br>Take care…

Daily Review Feedback Category 22: Moderate Risk – Sleeping Too Little, Good Sleep Environments (Choice 2.0)

Reflect (P8)	Reflect (P7)	Environment (S2)	Goals (U7)	Problem Solve (U8)	A & A (P7)
**1**<br>**RANDOM UNLINKED**	**2**<br>**RANDOM UNLINKED**	**3**<br>**STATIC**	**4**<br>**RANDOM LINKED**	**5**<br>**RANDOM LINKED**	**6**<br>**RANDOM UNLINKED**
You say you're doing well. That's good.<br><br>In order to stay well, you may want to consider making some adjustments to your sleep routine. It seems as though lately, you may be getting less sleep than you need.<br><br>Continue to learn more…	Unfortunately, many things can interfere with sleep. Do you feel like you have control over how much you sleep? Do you want to change how well you're sleeping?<br><br>Read about…	Good sleep environments include:<br><br>• A comfortable mattress<br>• A comfortable temperature<br>• A quiet partner (if you have one)<br>• A quiet bedroom<br>• No television<br>• No radio<br>• No computer<br>• No smartphone<br>• No desk<br><br>Your bedroom should be a restful space, and it should be reserved for rest. It helps "train" your body to sleep regularly and deeply.<br><br>Continue for tips …	SETTING GOALS<br><br>How might you improve your sleep environment? It can always get better?<br><br>Do you need to change your mattress?<br>Get different blankets?<br>Get ear plugs?<br>Move your television, radio, computer, or desk out of your room?<br>Do you need to make a pact with yourself to turn your phone off at night?<br><br>Whatever you decide, keep the goal realistic. Be sure you have control over the outcome.	TEST IT OUT<br><br>Take a look at your bedroom. Try to come up with three things you can do in order to make it a more restful environment.<br><br>Focus on your senses:<br><br>• Sight. What might you do to make the appearance of your bedroom more restful? Get rid of clutter? Paint it a cool, restful color?<br>• Sound. What might you do to make the noise in your room more restful? Ear plugs? A sound machine?<br>• Touch. What might you do to make the feel of your bed cozier? Get a new mattress? How about a fluffy blanket?<br><br>A dark, quiet, and orderly space promotes sleep.	Take a minute to review your Mild Up and Mild Down descriptions in Awareness & Action in the Wellness Plan.<br><br>Sleep changes can be and early warning sign of mania or depression. Make sure you aren't having any other symptoms.<br><br>Stay well…

Daily Review Feedback Category 22: Moderate Risk – Sleeping Too Little, Good Sleep Environments (Choice 2.0)

Reflect (P9)	Reflect (P8)	Environment (S2)	Normalize (U8)	Support (U9)	My Skills (P8)

1<br>RANDOM UNLINKED	2<br>RANDOM UNLINKED	3<br>STATIC	4<br>RANDOM LINKED	5<br>RANDOM LINKED	6<br>RANDOM UNLINKED
Good to see you're doing well.<br><br>To keep it going, you should think about getting more sleep. Getting enough sleep is one of the most important things you can do to stay well.<br><br>Continue to read more.	Most people need between 6 to 10 hours of sleep a night. It varies from person to person. How much do you need in order to feel rested?<br><br>It appears you may not be getting enough sleep. Are you willing to consider making lifestyle changes in order to sleep more?<br><br>Read about...	Good sleep environments include:<br><br>• A comfortable mattress<br>• A comfortable temperature<br>• A quiet partner (if you have one)<br>• A quiet bedroom<br>• No television<br>• No radio<br>• No computer<br>• No smartphone<br>• No desk<br><br>Your bedroom should be a restful space, and it should be reserved for rest. It helps "train" your body to sleep regularly and deeply.<br><br>Continue for tips ...	CLINICIAN'S CORNER<br><br>It is surprising that making small changes in life can be so challenging! What seems so simple in theory can end up being hard in practice.<br><br>Don't beat yourself up if you are having trouble sleeping! Making a change is tough.<br><br>The Building Skills section of this application presents ideas about how to effectively develop and implement change plans.<br><br>------Dr. Cynthia Dopke	GET SUPPORT<br><br>Join DBSA:<br><br>www.dbsalliance.org<br><br>Join NAMI:<br><br>www.nami.org<br><br>Get support in your efforts to live well!	Have you saved any skills in the My Resources section that might help get your sleep back on track?<br><br>Take a look at the Wellness Plan! Stay well...

Daily Review Feedback Category 22: Moderate Risk – Sleeping Too Little, Good Sleep Environments (Choice 2.0)

Risk (P10)	Risk (P9)	Environment (S2)	Barriers (U9)	Problem Solve (U10)	(P5)
**1**<br>**RANDOM UNLINKED**	**2**<br>**RANDOM UNLINKED**	**3**<br>**STATIC**	**4**<br>**RANDOM LINKED**	**5**<br>**RANDOM LINKED**	**6**<br>**RANDOM UNLINKED**
Glad to see you're doing well. However, it looks like you haven't been getting enough sleep the past few nights.<br><br>Remember that sleep loss and poor---quality sleep can trigger symptoms.<br><br>Continue to learn more…	You are sleeping less than you said you need to in order to feel and function well.<br><br>What is getting in the way? Read about…	Good sleep environments include:<br><br>• A comfortable mattress<br>• A comfortable temperature<br>• A quiet partner (if you have one)<br>• A quiet bedroom<br>• No television<br>• No radio<br>• No computer<br>• No smartphone<br>• No desk<br><br>Your bedroom should be a restful space, and it should be reserved for rest. It helps "train" your body to sleep regularly and deeply.<br><br>Continue for tips …	ANTICIPATING OBSTACLES<br><br>What is getting in the way of creating a good sleep environment?<br><br>• Do you not have money to buy a better mattress?<br>• Do you not have control over the heat in your home?<br>• Is it loud outside your home? Or is your partner loud at night?<br>• Do you live in a studio apartment where all your belongings are in one room?	OVERCOMING BARRIERS<br><br>What can you do to work around whatever is getting in the way of a good sleep environment?<br><br>• Could you put a board under your mattress to make it firmer?<br>• Could you open a window, buy a fan, buy a warm blanket, or get new pajamas?<br>• Do you need ear plugs, a sound machine, or to move?<br>• Do you need to get a room divider, dresser, bins, or help organizing?	Stay well…

Daily Review Feedback Category 22: Moderate Risk – Sleeping Too Little, Exercise, Diet, and Substances (Choice 3.0)

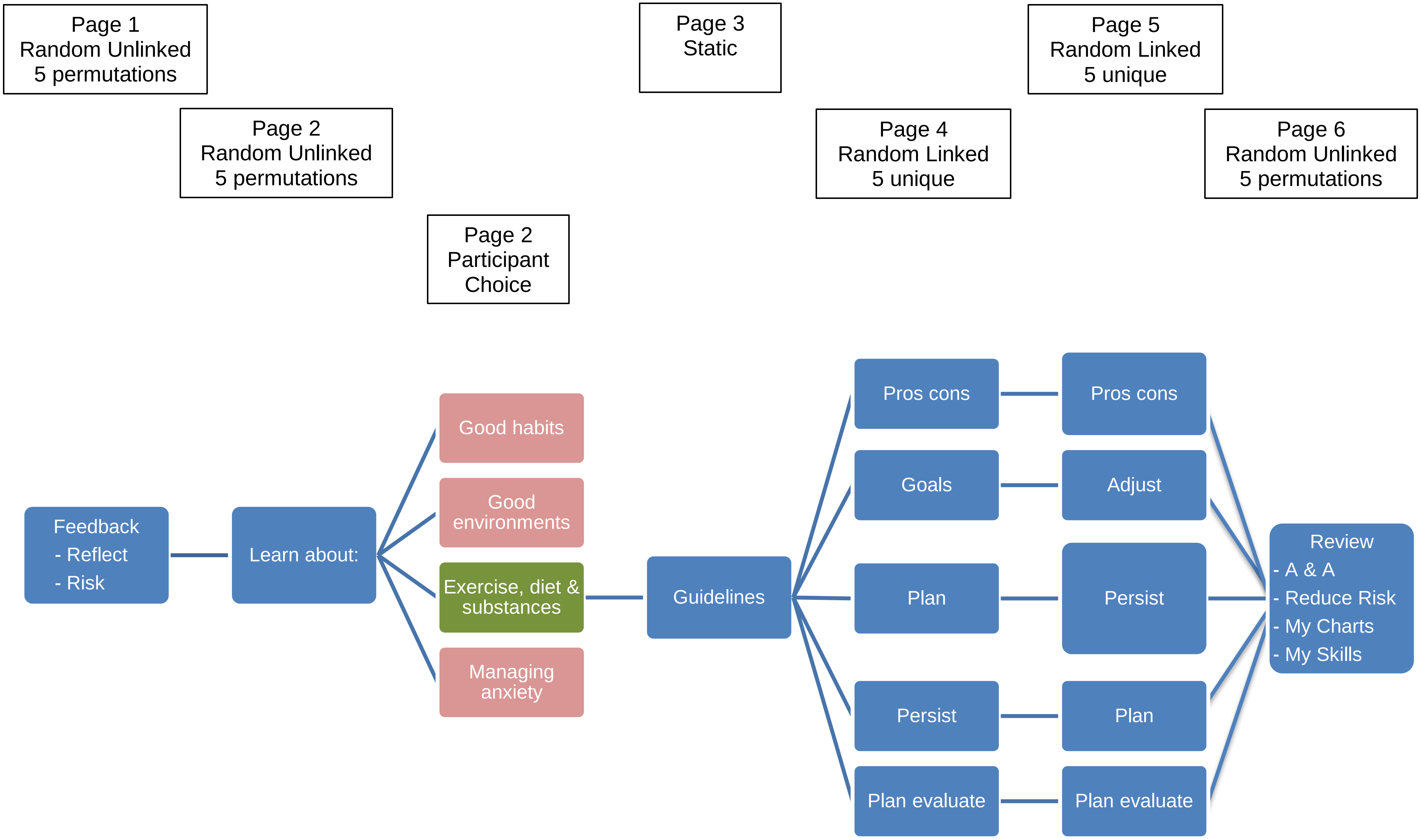

Daily Review Feedback Category 22: Moderate Risk – Sleeping Too Little, Exercise, Diet, and Substances (Choice 3.0)

Reflect (P1)	Barriers (P1)	Guidelines (S3)	Pros Cons (U1)	Pros Cons (U11)	My Charts (P1)
**1**<br>**RANDOM UNLINKED**	**2**<br>**RANDOM UNLINKED**	**3**<br>**STATIC**	**4**<br>**RANDOM LINKED**	**5**<br>**RANDOM LINKED**	**6**<br>**RANDOM UNLINKED**
Glad to see you're well.<br><br>You may, however, want to take a look at your sleep. It seems as though you have been sleeping more than usual the past few nights.<br><br>Continue to learn more about sleeping well.	A lot of things can get in the way of getting adequate sleep. Some details might surprise you!<br><br>Read about…	Taking care of your body is part of a good night's rest. Consider the following:<br><br>• Eat healthy<br>• Avoid alcohol and drugs<br>• Minimize caffeine intake<br>• Avoid exercising within a couple of hours of bedtime<br><br>Following these guidelines is easier said than done.<br><br>Continue for tips …	KNOW YOUR COSTS AND BENEFITS<br><br>Define the prize! Think about or even write down what you will gain by getting more sleep.<br><br>What will you have to give up if you get more sleep?<br><br>Knowing your own pros and cons for getting more sleep can help you get motivated to make a change.<br><br>Unless the pros outweigh the cons for them, most people find it hard to make and maintain a change, like getting more sleep.	EXAMPLE<br><br>Say your medications make you sleepy during the day. So, to counteract that you drink caffeine all day long. As a result, you can't fall asleep before 1:00 a.m.<br><br>Pros of cutting back on caffeine:<br>• Feel better rested<br>• Have more patience, be less irritable<br>• More productive at work<br><br>Cons of cutting back on caffeine:<br>• Tired most of the day<br><br>Perhaps you could cut down on caffeine and talk with your psychiatrist about feeling overly sedated.	Check out the relationship between your sleep and wellness in My Charts in the Wellness Plan below.<br><br>Be well!

Daily Review Feedback Category 22: Moderate Risk – Sleeping Too Little, Exercise, Diet, and Substances (Choice 3.0)

Reflect (P11)	Normalize (P10)	Guidelines (S3)	Goals (U10)	Adjust (U12)	(P5)
**1**<br>**RANDOM UNLINKED**	**2**<br>**RANDOM UNLINKED**	**3**<br>**STATIC**	**4**<br>**RANDOM LINKED**	**5**<br>**RANDOM LINKED**	**6**<br>**RANDOM UNLINKED**
Good to see you're doing well.<br><br>Take a look at your sleep. You probably need more rest in order to stay well.<br><br>Continue to learn more...	You are not alone. A lot of people struggle to keep a regular sleep schedule.<br><br>Read about...	Taking care of your body is part of a good night's rest. Consider the following:<br><br>• Eat healthy<br>• Avoid alcohol and drugs<br>• Minimize caffeine intake<br>• Avoid exercising within a couple of hours of bedtime<br><br>Following these guidelines is easier said than done.<br><br>Continue for tips ...	SETTING GOALS<br><br>Make them action---based. For example, "exercise 20 minutes 3 times a week" rather than "get in better shape".<br><br>Focus on what you want, not what you don't want. For example, "eat three servings of vegetables a day" rather than "avoid chocolate".<br><br>Make your goal realistic. Start small and work your way up. If you're not exercising at all, don't expect yourself to start exercising daily right away.	EXAMPLE<br><br>Say you have two cocktails before bedtime to help you fall asleep. Sometimes alcohol does help people fall asleep, but it is also associated with middle of the night awakening.<br><br>You decide you should try changing this pattern to see if you sleep better. Start by cutting back to just one cocktail per night, and once you have done that for three days cut back to ½ of a drink. After three days, cut out alcohol all together.<br><br>See what happens.	Stay well...

Daily Review Feedback Category 22: Moderate Risk – Sleeping Too Little, Exercise, Diet, and Substances (Choice 3.0)

Reflect (P12)	Reciprocal (P11)	Guidelines (S3)	Plan (U11)	Persist (U13)	Reduce Risk (P9)
**1**<br>**RANDOM UNLINKED**	**2**<br>**RANDOM UNLINKED**	**3**<br>**STATIC**	**4**<br>**RANDOM LINKED**	**5**<br>**RANDOM LINKED**	**6**<br>**RANDOM UNLINKED**
Nice to see you're doing well.<br><br>Do all you can to stay well! You may want to take a look at your sleep patterns. It seems like you're not getting the proper rest.<br><br>Read more…	Good sleep is related to good mental and physical health. Good sleep promotes good health, and good health promotes good sleep. It works both ways.<br><br>Read about…	Taking care of your body is part of a good night's rest. Consider the following:<br><br>• Eat healthy<br>• Avoid alcohol and drugs<br>• Minimize caffeine intake<br>• Avoid exercising within a couple of hours of bedtime<br><br>Following these guidelines is easier said than done.<br><br>Continue for tips …	STAYING FOCUSED<br><br>Identify positive changes you would like to make in order to be healthier. Is it:<br><br>• Nutrition<br>• Alcohol and drugs<br>• Caffeine<br>• Exercise<br><br>Make a realistic plan for progress. Be clear in your plan about <u>when</u> you are going to do the desired activities.<br><br>When the time comes, no matter what, just get started. And then stay focused. Zoom in!<br><br>Notice any intrusive thoughts, distractions, competing impulses, temptations, and thoughts of old habits. And let them go. Return your attention to your goal.	EXAMPLE<br><br>Say you decide to take a brisk walk for 10 minutes every evening upon returning home from work.<br><br>Each night when you arrive home, no matter what, put on your tennis shoes. (In fact, you may want to keep them out by the door to remind yourself.) Once you have your shoes on, no matter what, step outside.<br><br>Keep focused on how good you'll feel once you accomplish your goal. Notice any thoughts of discomfort or desire to just plop down on the couch. Then return your attention to the walk and feelings of accomplishment.	Review your sleep plan in Reduce Risk in the Wellness Plan.<br><br>Stay well…

Daily Review Feedback Category 22: Moderate Risk – Sleeping Too Little, Exercise, Diet, and Substances (Choice 3.0)

Reflect (P13)	Risk (P12)	Guidelines (S3)	Persist (U12)	Plan (U14)	A & A (P10)

1<br>**RANDOM UNLINKED**	2<br>**RANDOM UNLINKED**	3<br>**STATIC**	4<br>**RANDOM LINKED**	5<br>**RANDOM LINKED**	6<br>**RANDOM UNLINKED**
You say you're doing well. That's great.<br><br>Consider your sleep patterns. It appears you are not getting enough rest.<br><br>Continue…	Getting proper rest is important in maintaining your wellness.<br><br>What might help you get a better night's sleep?<br><br>Read about…	Taking care of your body is part of a good night's rest. Consider the following:<br><br>• Eat healthy<br>• Avoid alcohol and drugs<br>• Minimize caffeine intake<br>• Avoid exercising within a couple of hours of bedtime<br><br>Following these guidelines is easier said than done.<br><br>Continue for tips …	GETTING STARTED<br><br>Getting started is oftentimes the hardest part of change. Here are some things you can do in order to increase your chances of success:<br><br>Keep your eye on the prize. Remember how this is going to help you. Review your commitment.<br><br>Think about how good you'll feel once you've accomplished your goal.<br><br>Set your mind on at least starting—for one minute, or five minutes.	EXAMPLE<br><br>Let's say you decide to eat healthier. Late night fast food, you think, is impacting your health.<br><br>You find that at the end of the day you are simply too tired to prepare meals. So instead you order out— cheeseburgers, pizza, etc.<br><br>Purchase plenty of fruits and vegetables for your home. Prepare meals in advance (maybe on Sunday when you're not so tired) or find really basic recipes (salads or stir fry).<br><br>When the time comes for dinner, just get started. Pull out the vegetables and set them on the counter!	Changes in sleep can be an early warning sign of mania and depression.<br><br>Double check for symptoms in Awareness & Action in the Wellness Plan.<br><br>Stay well…

Daily Review Feedback Category 22: Moderate Risk – Sleeping Too Little, Exercise, Diet, and Substances (Choice 3.0)

Reflect (P14)	Risk (P13)	Guidelines (S3)	Plan Evaluate (U13)	Plan Evaluate (U15)	My Skills (P11)
**1**<br>**RANDOM UNLINKED**	**2**<br>**RANDOM UNLINKED**	**3**<br>**STATIC**	**4**<br>**RANDOM LINKED**	**5**<br>**RANDOM LINKED**	**6**<br>**RANDOM UNLINKED**
Seems like you're doing well. That's nice.<br><br>You may want, however, to consider your sleep patterns. Looks like you may need more rest.<br><br>Press continue…	Just because you can function with less than optimal sleep doesn't mean you should.<br><br>Not getting enough sleep – especially on a regular basis---causes mental and physical wear and tear.<br><br>Read about…	Taking care of your body is part of a good night's rest. Consider the following:<br><br>• Eat healthy<br>• Avoid alcohol and drugs<br>• Minimize caffeine intake<br>• Avoid exercising within a couple of hours of bedtime<br><br>Following these guidelines is easier said than done.<br><br>Continue for tips …	KEYS TO SUCCESS<br><br>1. Make a commitment. Clearly identify the advantages of making a change in your life. Also consider any disadvantages. What can you do about the disadvantages?<br><br>2. Make a plan. Be sure it is action---based, realistic, and has a time frame. Be sure it is specific and that there is a 95% chance you can succeed.<br><br>3. Get started and stay focused. Be sure the time and place you choose promotes healthy behavior.<br><br>4. Evaluate the results. How did it go? If well, job well done. If not, what can you learn? What do you need to adjust in order to succeed?	EXAMPLE<br><br>Say you're interested in cutting out all alcohol.<br><br>1. Make a commitment. Why is this important? What will you gain? Better sleep? Better moods? How about the disadvantages? It helps you relax? Are there other ways to relax?<br><br>2. You're only drinking one glass of wine a day. So you decide to cut back to 1/2 glass for a week. That seems realistic.<br><br>3. When time comes to serve yourself, choose a smaller glass. Or maybe post a reminder. (Not that you would forget.)<br><br>4. Try it for a week. How many days were you successful? If 5 of 7, what helped? What got in the way the other 2 days? Were those days more stressful and it was hard to stick to your goal?	Have you saved any sleep skills in My Resources? Check them out in the Wellness Plan.<br><br>Stay well…

Daily Review Feedback Category 22: Moderate Risk - Sleeping Too Little, Managing Anxiety (Choice 4.0)

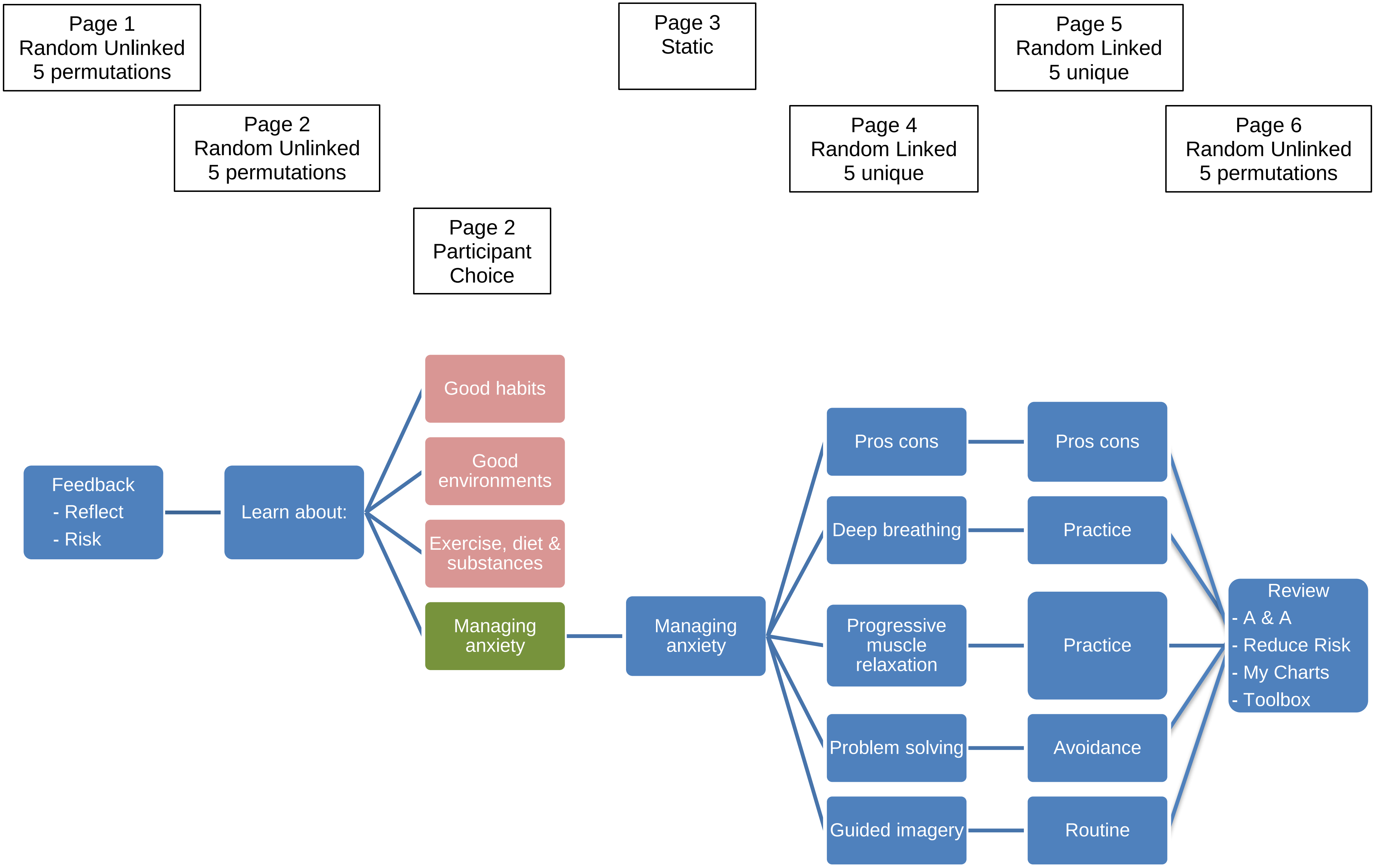

Daily Review Feedback Category 22: Moderate Risk - Sleeping Too Little, Managing Anxiety (Choice 4.0)

Reflect (P1)	Barriers (P11)	Anxiety (S4)	Pros Cons (U1)	Pros Cons (U16)	My Charts (P1)
**1**<br>**RANDOM UNLINKED**	**2**<br>**RANDOM UNLINKED**	**3**<br>**STATIC**	**4**<br>**RANDOM LINKED**	**5**<br>**RANDOM LINKED**	**6**<br>**RANDOM UNLINKED**
Glad to see you're doing well.<br><br>You may want, however, to take a look at your sleep patterns. It seems like you're not getting the proper rest.<br><br>Press continue to learn more about sleeping well.	A lot of things can get in the way of getting adequate sleep. Some details might surprise you!<br><br>Read about...	It is not uncommon for people to begin worrying about problems as soon as they get in bed and the lights are off. There are no distractions.<br>Sometimes people worry about falling asleep.<br><br>Nighttime is not the time to worry. It is time to rest!<br><br>If you worry in bed, try<br>(1) calming skills such as deep breathing, guided imagery, or progressive muscle relaxation and/or<br>(2) writing down your worries on a piece of paper and leaving them until morning for consideration.<br><br>Following these guidelines is easier said than done.<br><br>Continue for tips ...	KNOW YOUR COSTS AND BENEFITS<br><br>Define the prize! Think about or even write down what you will gain by getting more sleep.<br><br>What will you have to give up if you get more sleep?<br><br>Knowing your own pros and cons for getting more sleep can help you get motivated to make a change.<br><br>Unless the pros outweigh the cons for them, most people find it hard to make and maintain a change, like getting more sleep.	EXAMPLE<br><br>Say as soon as you turn out the lights you begin to worry about anything and everything.<br><br>Pros of letting go of worry at night:<br>• Feel better rested<br>• Have more patience, be less irritable<br>• More productive at work<br><br>Cons of letting go of worry at night:<br>• It's a good, quiet time to think	Check out the relationship between your sleep and wellness in My Charts in the Wellness Plan below.<br><br>Be well!

Daily Review Feedback Category 22: Moderate Risk - Sleeping Too Little, Managing Anxiety (Choice 4.0)

Reflect (P15)	Reflect (P14)	Anxiety (S4)	Deep Breathing (U14)	Practice (U17)	(P5)
**1**<br>**RANDOM UNLINKED**	**2**<br>**RANDOM UNLINKED**	**3**<br>**STATIC**	**4**<br>**RANDOM LINKED**	**5**<br>**RANDOM LINKED**	**6**<br>**RANDOM UNLINKED**
Good to see you're doing well.<br><br>Be sure to take a look at your sleep patterns. It seems like you're not getting the proper rest.<br><br>Continue to read more...	Have you noticed that you've been getting less sleep than you need lately? Do you know why this is happening?<br><br>There are many reasons why people don't get enough sleep. Do any of these apply to you?	It is not uncommon for people to begin worrying about problems as soon as they get in bed and the lights are off. There are no distractions.<br>Sometimes people worry about falling asleep.<br><br>Nighttime is not the time to worry. It is time to rest!<br><br>If you worry in bed, try<br>(1) calming skills such as deep breathing, guided imagery, or progressive muscle relaxation<br>and/or<br>(2) writing down your worries on a piece of paper and leaving them until morning for consideration.<br><br>Following these guidelines is easier said than done.<br><br>Continue for tips ...	TIP OF THE DAY<br><br>Your diaphragm is the muscle that controls breathing. It is located just below your lungs. Using your diaphragm to its fullest is calming.<br><br>Breathe by using your belly rather than your chest. This is the best way.<br><br>TAKE SLOW, DEEP, BELLY BREATHS<br><br>Variations:<br><br>• Count to three as you breathe in and count to three as you breathe out.<br>• Think "and" as you breathe in and "let go" as you breathe out.	REMEMBER...<br><br>Coping skills take time to develop. Just like getting physically fit, getting mentally fit takes practice.<br><br>Practice deep breathing several times a day for several days. Build your "mental muscle". Then deep breathing at night should eventually be more effective.<br><br>Don't give up!	Stay well...

Daily Review Feedback Category 22: Moderate Risk - Sleeping Too Little, Managing Anxiety (Choice 4.0)

Reflect (P16)	Normalize (P15)	Anxiety (S4)	Relaxation (U15)	Practice (U18)	Toolbox (P12)
**1**<br>**RANDOM UNLINKED**	**2**<br>**RANDOM UNLINKED**	**3**<br>**STATIC**	**4**<br>**RANDOM LINKED**	**5**<br>**RANDOM LINKED**	**6**<br>**RANDOM UNLINKED**
Nice to see you're doing well.<br><br>You may want, however, to take a look at your sleep. You're getting less than you said you need.<br><br>Continue...	A lot of people have a hard time keeping a regular sleep schedule. Many things can get in the way of sleeping well or sleeping long enough to feel rested.<br><br>What's getting in your way?<br><br>Read about...	It is not uncommon for people to begin worrying about problems as soon as they get in bed and the lights are off. There are no distractions.<br>Sometimes people worry about falling asleep.<br><br>Nighttime is not the time to worry. It is time to rest!<br><br>If you worry in bed, try (1) calming skills such as deep breathing, guided imagery, or progressive muscle relaxation and/or (2) writing down your worries on a piece of paper and leaving them until morning for consideration.<br><br>Following these guidelines is easier said than done.<br><br>Continue for tips ...	TIP OF THE DAY<br><br>Reducing tension in your body is calming. Do this for each set of muscles:<br><br>TENSE 30 SECONDS, RELAX 60 SECONDS<br><br>• Face<br>• Neck<br>• Arms<br>• Hands<br>• Shoulders<br>• Torso<br>• Legs<br>• Feet<br><br>Pay attention to how your muscles feel when relaxed.	REMEMBER...<br><br>Doing this perfectly is not necessary. You do not need perfect focus.<br><br>Undoubtedly your mind will wander. Just notice that and gently bring your attention back to your muscles.<br><br>Over and over...	Read more about managing stress in Toolbox.<br><br>Stay well...

Daily Review Feedback Category 22: Moderate Risk - Sleeping Too Little, Managing Anxiety (Choice 4.0)

Reflect (P17)	Risk (P16)	Anxiety (S4)	Problem Solving (U16)	Avoidance (U19)	Reduce Risk (P9)
**1**<br>**RANDOM UNLINKED**	**2**<br>**RANDOM UNLINKED**	**3**<br>**STATIC**	**4**<br>**RANDOM LINKED**	**5**<br>**RANDOM LINKED**	**6**<br>**RANDOM UNLINKED**
You say you're doing well. That's good.<br><br>Consider adjusting your sleep. Looks like you may not be getting the proper rest.<br><br>Read more…	Sleep is critical for good mental and physical health. Although you can certainly "get away" with sleeping less from time to time, it will take its toll.<br><br>Read about…	It is not uncommon for people to begin worrying about problems as soon as they get in bed and the lights are off. There are no distractions.<br>Sometimes people worry about falling asleep.<br><br>Nighttime is not the time to worry. It is time to rest!<br><br>If you worry in bed, try<br>(1) calming skills such as deep breathing, guided imagery, or progressive muscle relaxation<br>and/or<br>(2) writing down your worries on a piece of paper and leaving them until morning for consideration.<br><br>Following these guidelines is easier said than done.<br><br>Continue for tips …	STEPS TO PROBLEM SOLVING<br><br>Are you not sleeping at night because you are avoiding solving problems during the day? Or procrastinating in terms of getting things done during the day?<br><br>Avoiding problems causes more problems! Always! For things that can be changed…act!<br><br>1. Identify the problem. Describe it in detail.<br>2. Select your goal. Describe what you would like to see happen.<br>3. Generate alternative solutions. Come up with several plans.<br>4. Evaluate the alternatives. Which are practical? Which will work best?<br>5. Implement your plan. Decide on a time and place. Do it!<br>6. Evaluate the results. Did it work? If not should you do something else?<br><br>Get on top of problems during the day so that you can rest at night!	REMEMBER<br><br>Depression is associated with avoiding problems. It works both ways. When depressed, people often feel overwhelmed and avoid problems. When people avoid problems, they often get depressed.<br><br>Avoidance and anxiety and depression all go together and cause a spiral of pain.<br>Approach and solve problems for your mental health!	Review your sleep plan in Reduce Risk in the Wellness Plan.<br><br>Stay well…

Daily Review Feedback Category 22: Moderate Risk - Sleeping Too Little, Managing Anxiety (Choice 4.0)

Reflect (P18)	Risk (P17)	Anxiety (S4)	Imagery (U17)	Routine (U20)	A & A (P13)
**1**<br>**RANDOM UNLINKED**	**2**<br>**RANDOM UNLINKED**	**3**<br>**STATIC**	**4**<br>**RANDOM LINKED**	**5**<br>**RANDOM LINKED**	**6**<br>**RANDOM UNLINKED**
Nice thing that you're well.<br><br>Stay well. Consider getting more sleep!<br><br>Read more about sleeping well…	Don't forget that changes in your sleep schedule - including how much you sleep - can trigger symptoms. So be cautious! Take care of yourself.<br><br>Read about…	It is not uncommon for people to begin worrying about problems as soon as they get in bed and the lights are off. There are no distractions. Sometimes people worry about falling asleep.<br><br>Nighttime is not the time to worry. It is time to rest!<br><br>If you worry in bed, try<br>(1) calming skills such as deep breathing, guided imagery, or progressive muscle relaxation and/or<br>(2) writing down your worries on a piece of paper and leaving them until morning for consideration.<br><br>Following these guidelines is easier said than done.<br><br>Continue for tips …	GUIDED IMAGERY<br><br>Access the web. Google "guided imagery". You will find many resources available.<br><br>Many people relax better when someone talks them through it. Are you one of those people?	REMEMBER<br><br>Take care of your day to day obligations and problems during the day so you can rest at night.<br><br>Resting at night will also help you take care of your day to day obligations and problems.<br><br>Did you know that adequate sleep helps with:<br><br>• Attention<br>• Concentration<br>• Memory<br>• Emotional resilience<br>• Motor skills	Sleep problems can be an early warning sign of mania or depression.<br><br>Review your descriptions of Mild Up and Mild Down in Awareness & Action in the Wellness Plan below.<br><br>Be sure you're not symptomatic.<br><br>Stay well…

Daily Review Feedback Category 23: Moderate Risk – Sleeping Too Much, Sedating Medications (Choice 1.0)

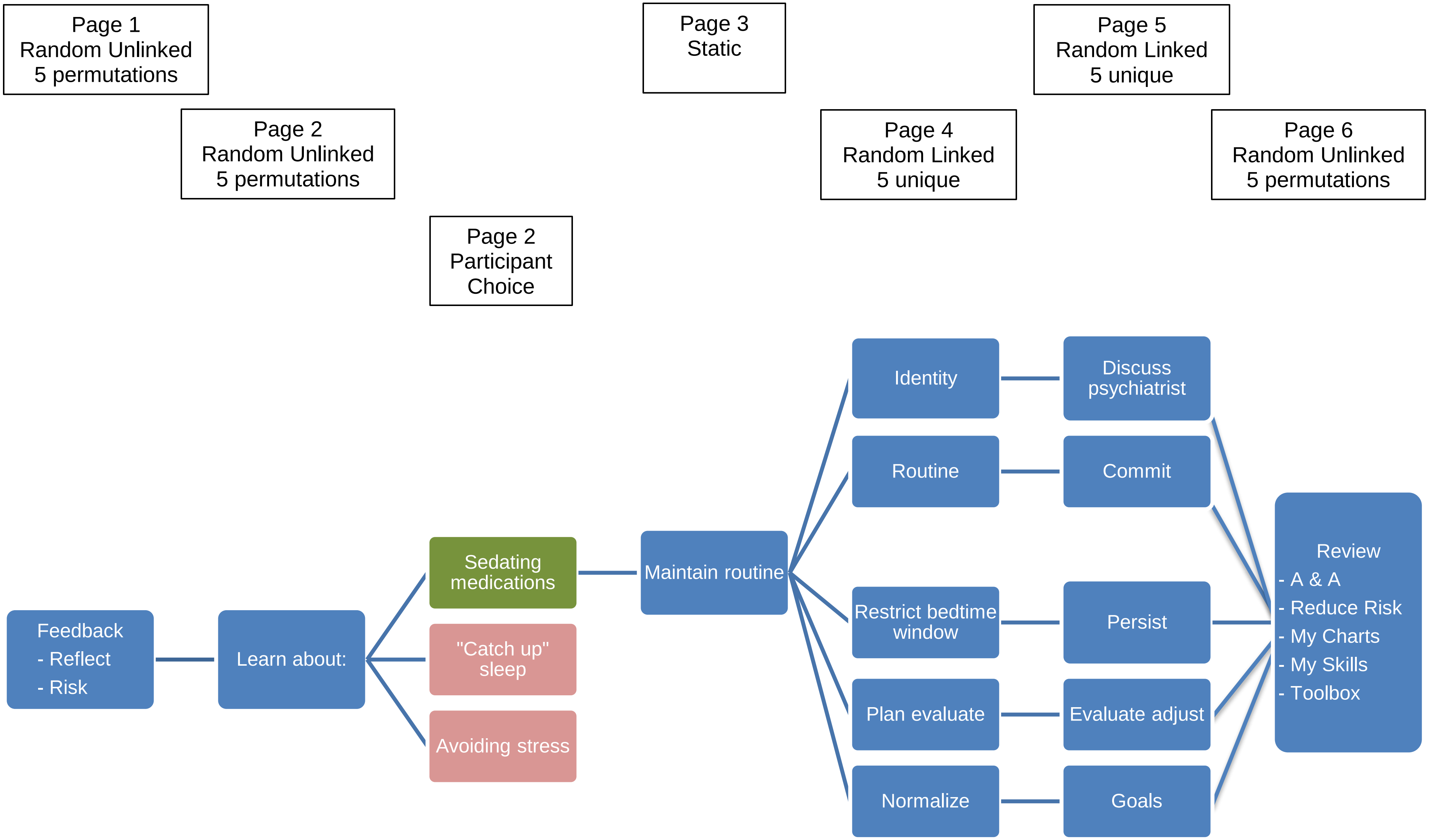

Daily Review Feedback Category 23: Moderate Risk – Sleeping Too Much, Sedating Medications (Choice 1.0)

Reflect (P1)	Risk Reflect (P1)	Routine (S1)	Identity (U1)	Discuss (U1)	My Charts (P1)
**1**<br>**RANDOM UNLINKED**	**2**<br>**RANDOM UNLINKED**	**3**<br>**STATIC**	**4**<br>**RANDOM LINKED**	**5**<br>**RANDOM LINKED**	**6**<br>**RANDOM UNLINKED**
Glad to see you're doing well.<br><br>You may, however, want to take a look at your sleep patterns. It seems as though you have been sleeping more than usual the past few nights.<br><br>Continue to learn more about sleeping well.	Sleeping too much, while it might feel good, can throw things off. It can trigger symptoms.<br><br>The reasons people sleep too much vary. Below are some common causes.<br><br>Before reading further, double---check your wellness rating. Sleeping a lot can be a symptom of depression. Is it possible you are down?<br><br>Read about…	For general health, it is important to get the right amount of sleep on a daily basis. Not too much sleep and not too little sleep.<br><br>Keep a regular sleep schedule:<br><br>• Turn in the same time each night<br>• Arise the same time each morning<br>• Avoid daytime napping<br><br>Following these guidelines is easier said than done.<br><br>Continue for tips …	WHAT DOES IT MEAN?<br><br>What are your aims in life? What do you value?<br>How would you really like to spend your time?<br><br>Will sleeping less help you live more fully?<br>Will it leave you more time for desired activities?<br>How would life be different if you slept less, say 6 to 10 hours a night?<br><br>OVERCOMING BARRIERS<br><br>Don't forget your life aims and values. Act now to overcome any obstacles.<br><br>What can you do about the sedation caused by medications?	EXAMPLE<br><br>Say you've been sleeping 12 hours a day. You know that usually you only need 9 hours.<br><br>Life with less sleep:<br><br>• More time with spouse<br>• More time to exercise<br>• More time for friends<br><br>Solution:<br>Call your psychiatrist and discuss the problems you are having with your medications. You may need a different dose, to take it at a different time, or a different medicine altogether.	Check out the relationship between your sleep and wellness in My Charts in the Wellness Plan below.<br><br>Be well!

Daily Review Feedback Category 23: Moderate Risk – Sleeping Too Much, Sedating Medications (Choice 1.0)

Reflect (P2)	Risk Reflect (P2)	Routine (S1)	Routine (U2)	Commit (U2)	Reduce Risk (P2)
**1**<br>**RANDOM UNLINKED**	**2**<br>**RANDOM UNLINKED**	**3**<br>**STATIC**	**4**<br>**RANDOM LINKED**	**5**<br>**RANDOM LINKED**	**6**<br>**RANDOM UNLINKED**
Your wellness rating suggests that you are doing well.<br><br>Even so, take a few minutes now to consider your sleep schedule. It appears that you've been getting more sleep than you need recently.<br><br>Consider cutting back on the amount of sleep you are getting each night to stay well.<br><br>Continue to read more…	Sleeping too much can throw off your internal clock. This in turn can trigger symptoms.<br><br>Below are some common reasons for sleeping too much.<br><br>Before considering these scenarios, double---check your wellness rating. Sleeping a lot can be a symptom of depression. Is it possible you are down?<br><br>Read about…	For general health, it is important to get the right amount of sleep on a daily basis. Not too much sleep and not too little sleep.<br><br>Keep a regular sleep schedule:<br><br>• Turn in the same time each night<br>• Arise the same time each morning<br>• Avoid daytime napping<br><br>Following these guidelines is easier said than done.<br><br>Continue for tips …	MAKE A PLAN<br><br>Medications can be sedating. However, this does not mean you need more sleep when taking medications.<br><br>Set a sleep schedule. Getting out of bed may be the hardest part! When your alarm goes off…<br><br>• Keep your eye on the prize. Remember how this is going to help you. Remember your commitment.<br>• Think about how good you'll feel if you get up. (Don't focus on how good you'll feel if you stay in bed.)<br>• Commit to getting out of bed for just 5 minutes, at least to start. Everything counts.<br>• Just start moving. Literally, just get up and start moving your body.	EXAMPLE<br><br>Say you've been sleeping 12 hours a day. Before your medication change, you only needed 9 hours of sleep.<br><br>To counter this change, you decide to sleep from 10:00 pm until 7:00 am When your alarm goes off 7:00 am, you commit to getting out of bed, walking around your home, and pouring a cup of coffee. Even if you get back in bed, you are using this as your starting point.<br><br>Remember that when you are trying to make changes in life, everything counts.	Check out your sleep plan in Reduce Risk in the Wellness Plan.<br><br>Stay well…

Daily Review Feedback Category 23: Moderate Risk – Sleeping Too Much, Sedating Medications (Choice 1.0)

Reflect (P3)	Risk Reflect (P3)	Routine (S1)	Restrict (U3)	Persist (U3)	A & A (P3)
**1**<br>**RANDOM UNLINKED**	**2**<br>**RANDOM UNLINKED**	**3**<br>**STATIC**	**4**<br>**RANDOM LINKED**	**5**<br>**RANDOM LINKED**	**6**<br>**RANDOM UNLINKED**
You're rating yourself as doing well, which is great. However, it seems that lately you have been getting more sleep than usual.<br><br>Sleep is a central part of a healthy lifestyle. But the key is moderation. Getting too much sleep can put you at risk for symptoms.<br><br>Continue to learn more.	You got this alert because you may be sleeping more than you need. Sleeping too much can trigger symptoms.<br><br>Below are some common reasons for sleeping too much.<br><br>Before considering these scenarios, double---check your wellness rating. Sleeping a lot can be a symptom of depression. Is it possible you are down?<br><br>Read about…	For general health, it is important to get the right amount of sleep on a daily basis. Not too much sleep and not too little sleep.<br><br>Keep a regular sleep schedule:<br><br>• Turn in the same time each night<br>• Arise the same time each morning<br>• Avoid daytime napping<br><br>Following these guidelines is easier said than done.<br><br>Continue for tips …	YOU CAN DO IT!<br><br>Medications can be sedating, but you can still avoid sleeping too much.<br><br>The surprising thing about behavior change is that it often seems more difficult than it actually is.<br><br>Consider committing to restricting your sleep to 9 hours per night for the next three days.<br><br>You can develop your "mental muscle" (will power) through practice. Just set a very small goal and achieve it. Do it again. And again.	EXAMPLE<br><br>Say you've been sleeping 12 hours a day. You know that usually you only need 9 hours of sleep. You have already consulted your psychiatrist and you both agreed that this is still the best medicine for you right now.<br><br>Reclaim your schedule and your life. Get up when your alarm goes off. Just get out of bed. Walk around. You can do it!	Double check your wellness anchors in Awareness & Action in the Wellness Plan.<br><br>Make sure you're not depressed.<br><br>Be well!

Daily Review Feedback Category 23: Moderate Risk – Sleeping Too Much, Sedating Medications (Choice 1.0)

Reflect (P4)	Risk Reflect (P4)	Routine (S1)	Plan Evaluate (U4)	Evaluate Adjust (U4)	My Skills (P4)
**1**<br>**RANDOM UNLINKED**	**2**<br>**RANDOM UNLINKED**	**3**<br>**STATIC**	**4**<br>**RANDOM LINKED**	**5**<br>**RANDOM LINKED**	**6**<br>**RANDOM UNLINKED**
You're doing well overall, but it seems as though you've been sleeping more than usual lately.<br><br>Consider making some changes to your sleep schedule now to get back on track.<br><br>Continue to read more…	Sleeping too much can be dangerous. It throws off your internal clock and can trigger symptoms.<br><br>Below are some common reasons for sleeping too much.<br><br>Before considering these scenarios, double---check your wellness rating. Sleeping a lot can be a symptom of depression. Is it possible you are down?<br><br>Read about…	For general health, it is important to get the right amount of sleep on a daily basis. Not too much sleep and not too little sleep.<br><br>Keep a regular sleep schedule:<br><br>• Turn in the same time each night<br>• Arise the same time each morning<br>• Avoid daytime napping<br><br>Following these guidelines is easier said than done.<br><br>Continue for tips …	EVALUATE OUTCOMES<br><br>Even if medications make you tired, set a plan to sleep only the number of hours you need to feel rested.<br><br>Assess your effort towards completing your goal. Then evaluate the outcome. How did things go?<br><br>If you succeeded, notice the link between your effort and the outcome. Give yourself credit for a job well done.<br><br>If you did not succeed, then focus on what you can learn. Don't judge, but aim to understand.<br><br>Where did things break down?<br><br>• In preparing: Were you not motivated? What do you need to do?<br>• In planning: Do you need to change your plan?<br>• In performing: What got in the way? What can you do differently next time?<br><br>There is no failure. You either succeed in making changes, or you succeed in learning something.	EXAMPLE<br><br>Say you've been sleeping 12 hours a day. You know that usually you only need 9 hours of sleep.<br><br>You decide to turn in at 10:00 pm and set your alarm for 7:00 am You try this for several days. Despite your best efforts, you notice that you often failed to get up when your alarm went off.<br><br>To assess what went wrong, you evaluate both your efforts to achieve the goal and the outcome. In doing so, you realize that…<br><br>You are motivated to get the appropriate amount of sleep. However, you went from sleeping 12 hours to trying to get only 9 hours. You decide that your plan was unrealistic.<br><br>So you change the plan. You decide to turn in at 10:00 p.m. and get up at 9:00 a.m., reducing your sleep time by 1 hour.	Check out any sleep skills you added to your My Resources section of the Wellness Plan.<br><br>Stay well…

Daily Review Feedback Category 23: Moderate Risk – Sleeping Too Much, Sedating Medications (Choice 1.0)

Reflect (P5)	Risk Reflect (P5)	Routine (S1)	Normalize (U5)	Goals (U5)	Toolbox (P5)
**1**<br>**RANDOM UNLINKED**	**2**<br>**RANDOM UNLINKED**	**3**<br>**STATIC**	**4**<br>**RANDOM LINKED**	**5**<br>**RANDOM LINKED**	**6**<br>**RANDOM UNLINKED**
It's great that you're doing well.<br><br>To keep it going, take some time now to consider your sleep routine. It seems that you've been sleeping more than usual lately, which could put you at risk for symptoms.<br><br>Continue to learn more about sleep…	Sleeping a lot can feel good. However, it can also throw things off in term s of your mood.<br><br>Some common causes for sleeping too much are listed below.<br><br>Before considering these reasons, double---check your wellness rating. Sleeping a lot can be a symptom of depression. Is it possible you are down?<br><br>Read about…	For general health, it is important to get the right amount of sleep on a daily basis. Not too much sleep and not too little sleep.<br><br>Keep a regular sleep schedule:<br><br>• Turn in the same time each night<br>• Arise the same time each morning<br>• Avoid daytime napping<br><br>Following these guidelines is easier said than done.<br><br>Continue for tips …	CLINICIAN'S CORNER<br><br>I have seen so many individuals over the years struggle with their sleep. If it was so easy to correct, then this discussion would not even be happening!<br><br>Medications can be sedating. That doesn't mean you need more sleep! It just means that it will be harder for you to wake up.<br><br>Set your alarm. Get up. Have some coffee. Give yourself 30 minutes. You'll feel better.<br><br>------Dr. Cynthia Dopke	START LOW, GO SLOW<br><br>If you've been sleeping 12 hours a night, cut back to 11 hours. If you've been sleeping 11 hours a night, cut back to 10 hours. If you've been sleeping 10 hours a night, cut back to 9 hours.<br><br>Everything that you do in the right direction counts. So set easy goals and accomplish them.	Check out the Toolbox section of the application for more tips on keeping a regular sleep schedule.<br><br>Stay well.

Daily Review Feedback Category 23: Moderate Risk – Sleeping Too Much, Catch Up Sleep (Choice 2.0)

Page 1
Random Unlinked
5 permutations

Page 2
Random Unlinked
5 permutations

Page 2
Participant
Choice

Page 3
Static

Page 4
Random Linked
5 unique

Page 5
Random Linked
5 unique

Page 6
Random Unlinked
5 permutations

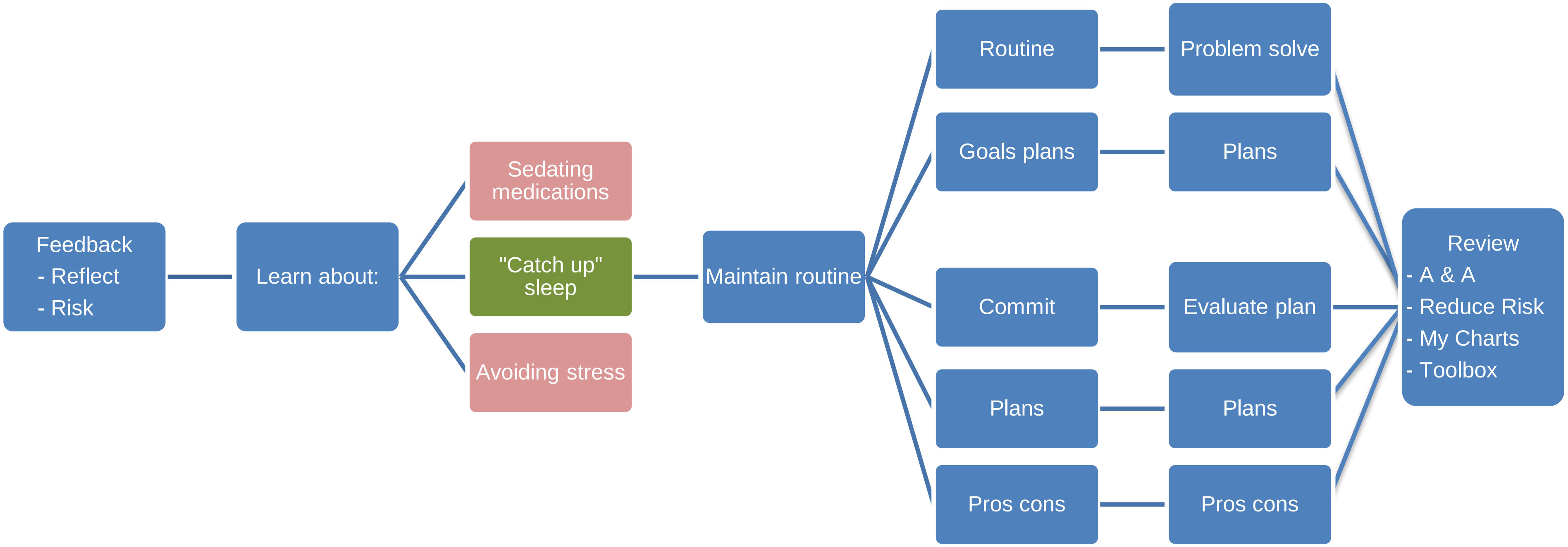

Daily Review Feedback Category 23: Moderate Risk – Sleeping Too Much, Catch Up Sleep (Choice 2.0)

Reflect (P6)	Risk Reflect (P1)	Routine (S1)	Routine (U6)	Problem Solve (U6)	My Charts (P1)
**1**<br>**RANDOM UNLINKED**	**2**<br>**RANDOM UNLINKED**	**3**<br>**STATIC**	**4**<br>**RANDOM LINKED**	**5**<br>**RANDOM LINKED**	**6**<br>**RANDOM UNLINKED**
Glad to see you're doing well.<br><br>You may want, however, to take a look at your sleep patterns. It seems like you might be sleeping too much.<br><br>Press continue to learn more about sleeping well.	Sleeping too much, while it might feel good, can throw things off. It can trigger symptoms.<br><br>The reasons people sleep too much vary. Below are some common causes.<br><br>Before reading further, double---check your wellness rating. Sleeping a lot can be a symptom of depression. Is it possible you are down?<br><br>Read about…	For general health, it is important to get the right amount of sleep on a daily basis. Not too much sleep and not too little sleep.<br><br>Keep a regular sleep schedule:<br><br>• Turn in the same time each night<br>• Arise the same time each morning<br>• Avoid daytime napping<br><br>Following these guidelines is easier said than done.<br><br>Continue for tips …	GET YOUR FACTS STRAIGHT<br><br>There actually is no such thing as "catching up" on lost sleep. This is a myth.<br><br>The best thing you can do is to get the proper rest every night. The next best thing you can do is to return to a regular schedule after any change in your sleep patterns.<br><br>Not only can sleep disruptions trigger symptoms, they impact attention, concentration, memory, mood, ability to manage stress, and motor skills!	FINDING SOLUTIONS THAT WORK<br><br>Say you sleep 6 hours during the week and 12 hours on the weekends. You like staying up late, but during the week you have to be up by 7:00 a.m. for work.<br><br>How might you even out your sleep schedule?<br><br>• Turn in earlier during the week<br>• Start work later during the week<br>• Shorten your commute to work somehow<br><br>Be creative!	Check out the relationship between your sleep and wellness in My Charts in the Wellness Plan below.<br><br>Be well!

Daily Review Feedback Category 23: Moderate Risk – Sleeping Too Much, Catch Up Sleep (Choice 2.0)

Reflect (P7)	Risk Reflect (P6)	Routine (S1)	Goals Plans (U7)	Plans (U7)	Reduce Risk (P2)
**1**<br>**RANDOM UNLINKED**	**2**<br>**RANDOM UNLINKED**	**3**<br>**STATIC**	**4**<br>**RANDOM LINKED**	**5**<br>**RANDOM LINKED**	**6**<br>**RANDOM UNLINKED**
Good to see you're doing well.<br><br>However, take some now to review your sleep schedule. It seems like you might be sleeping too much.<br><br>Continue to read more.	Sleeping too much messes with your internal clock. This can trigger symptoms.<br><br>Now is a good time to double check your wellness rating. Sleeping a lot can be a symptom of depression. Is it possible you are down?<br><br>If not, consider what is going on in your situation. Below are some common reasons people sleep too much. Do any of these apply to you?<br><br>Read about...	For general health, it is important to get the right amount of sleep on a daily basis. Not too much sleep and not too little sleep.<br><br>Keep a regular sleep schedule:<br><br>• Turn in the same time each night<br>• Arise the same time each morning<br>• Avoid daytime napping<br><br>Following these guidelines is easier said than done.<br><br>Continue for tips ...	MAKE A PLAN<br><br>• Set realistic sleep goals. If you need to make dramatic changes, do it step by step.<br><br>• Anticipate any obstacles that might prevent you from implementing your plan. This might be scheduled events, others wanting to spend time with you late at night, or television programs you like to watch.<br><br>• Create opportunities for success. Set a date to start your plan. Get your partner on board if you have one. Set an alarm to turn in at night!	EXAMPLE<br><br>Say you sleep 6 hours during the week and 12 hours on the weekends. You like staying up late, but have to get up at 7:00 am for work during the week. Then on the weekends, you usually end up sleeping until 1:00 pm in the afternoon.<br><br>Fix your weekend schedule first. Start by getting up 30 minutes earlier each day. Set an alarm or make a plan to meet someone somewhere so that you are more motivated to get out of bed. Making a public commitment can really help.	Check out your sleep plan in Reduce Risk in the Wellness Plan below.<br><br>Stay well...

Daily Review Feedback Category 23: Moderate Risk – Sleeping Too Much, Catch Up Sleep (Choice 2.0)

Reflect (P8)	Risk Reflect (P7)	Routine (S1)	Commitment (U8)	Evaluate Plan (U8)	Toolbox (P5)
**1**<br>**RANDOM UNLINKED**	**2**<br>**RANDOM UNLINKED**	**3**<br>**STATIC**	**4**<br>**RANDOM LINKED**	**5**<br>**RANDOM LINKED**	**6**<br>**RANDOM UNLINKED**
You are not reporting any symptoms, which is great.<br><br>In order to stay well, you may want to consider making some adjustments to your sleep routine. It seems as though lately, you may be getting more sleep than you need.<br><br>Continue to learn more…	Sleep generally feels good, often the more the better. However, too much sleep can cause problems.<br><br>Now is a good time to double check your wellness rating. Sleeping a lot can be a symptom of depression. Is it possible you are down?<br><br>Take some time to also consider other reasons you might be sleeping too much. Do any of these apply to you?<br><br>Read about…	For general health, it is important to get the right amount of sleep on a daily basis. Not too much sleep and not too little sleep.<br><br>Keep a regular sleep schedule:<br><br>• Turn in the same time each night<br>• Arise the same time each morning<br>• Avoid daytime napping<br><br>Following these guidelines is easier said than done.<br><br>Continue for tips …	MAKE A COMMITMENT<br><br>Making a commitment to change increases the likelihood you will take action. Here are some ways to make a commitment:<br><br>• Set a start date<br>• Set a time and place to start<br>• Tell someone about your intentions<br>• Do the activity with someone	EXAMPLE<br><br>Say your work schedule varies from day to day, or maybe week to week. You have gotten into the habit of sleeping in during your days off to "catch up".<br><br>Consider your work schedule and personal needs. Set a sleep schedule that makes sense, while also allowing you to get the right amount of sleep every night.<br><br>• Commit to starting on Sunday<br>• Tell your supports about your plan<br>• If you have a partner, ask your partner to do it with you.	Check out the Toolbox section of the application for additional tips on keeping a regular sleep schedule.<br><br>Stay well.

Daily Review Feedback Category 23: Moderate Risk – Sleeping Too Much, Catch Up Sleep (Choice 2.0)

Reflect (P9)	Risk Reflect (P8)	Routine (S1)	Plans (U9)	Plans (U9)	A & A (P6)
**1**<br>**RANDOM UNLINKED**	**2**<br>**RANDOM UNLINKED**	**3**<br>**STATIC**	**4**<br>**RANDOM LINKED**	**5**<br>**RANDOM LINKED**	**6**<br>**RANDOM UNLINKED**
Good to see you're doing well.<br><br>To keep it going, you should think about getting less sleep. Getting too much sleep on a regular basis can trigger symptoms.<br><br>Continue to read more.	Excessive sleep can cause problems. It can trigger symptoms.<br><br>Below are some common reasons people sleep too much.<br><br>Before considering these scenarios, double---check your wellness rating. Sleeping a lot can be a symptom of depression. Is it possible you are down?<br><br>Read about...	For general health, it is important to get the right amount of sleep on a daily basis. Not too much sleep and not too little sleep.<br><br>Keep a regular sleep schedule:<br><br>• Turn in the same time each night<br>• Arise the same time each morning<br>• Avoid daytime napping<br><br>Following these guidelines is easier said than done.<br><br>Continue for tips ...	ANTICIPATING OBSTACLES<br><br>Set a sleep schedule that makes sense across all days of the week. Make sure it gives you the sleep you require, not too much and not too little.<br><br>What might get in the way?<br><br>• Stress<br>• Old habits<br>• Competing desires<br>• Competing obligations<br>• Family and friends<br><br>Make a plan to overcome these obstacles.	EXAMPLE<br><br>Stress can keep you from falling asleep. If that is a problem, listen to some guided imagery or do progressive muscle relaxation upon getting in bed. (Check the web for directions.)<br><br>Enjoying time alone, when everyone else is asleep, can keep you from getting into bed. Try to carve out other times during the day for your alone---time.<br><br>You get the idea...	Double check your description for mild down in Awareness & Action in the Wellness Plan.<br><br>Excessive sleep can be a sign of depression.<br><br>Stay well...

Daily Review Feedback Category 23: Moderate Risk – Sleeping Too Much, Catch Up Sleep (Choice 2.0)

Risk (P10)	Risk Reflect (P9)	Routine (S1)	Pros Cons (U10)	Pros Cons (U10)	(FC22P5)
**1**<br>**RANDOM UNLINKED**	**2**<br>**RANDOM UNLINKED**	**3**<br>**STATIC**	**4**<br>**RANDOM LINKED**	**5**<br>**RANDOM LINKED**	**6**<br>**RANDOM UNLINKED**
Glad to see you're doing well.<br><br>However, it looks like you've been sleeping more than usual lately.<br><br>Remember that getting too much sleep puts you at risk for developing symptoms down the road.<br><br>Continue to learn more…	Your sleep and activity patterns help regulate you body's internal clock. Too much sleep can throw things off.<br><br>Sleeping too much can be a symptom of depression. Is it possible you are down?<br><br>Other common reasons for sleeping too much are listed below. Take some time now to consider why you've been sleeping more than usual lately. Do any of these apply to you?<br><br>Read about…	For general health, it is important to get the right amount of sleep on a daily basis. Not too much sleep and not too little sleep.<br><br>Keep a regular sleep schedule:<br><br>• Turn in the same time each night<br>• Arise the same time each morning<br>• Avoid daytime napping<br><br>Following these guidelines is easier said than done.<br><br>Continue for tips …	KNOW YOUR COSTS AND BENEFITS<br><br>Your sleep schedule is what it is for a reason.<br><br>What are the advantages of changing your sleep schedule? How would things be better?<br><br>What are the disadvantages of changing your sleep schedule? What would you lose?<br><br>Which list is more compelling? Is there a way to minimize the disadvantages and normalize your sleep schedule at the same time?	EXAMPLE<br><br>Say you sleep 6 hours a night on the days you work and 12 hours a night on your days off.<br><br>Advantages of normalizing your sleep might be that you reduce your risk of mood symptoms and have more time to do things on your days off.<br><br>Disadvantages of normalizing your sleep might be that you lose out on social time with friends during the work week.	Stay well…

Daily Review Feedback Category 23: Moderate Risk – Sleeping Too Much, Avoid Stress (Choice 3.0)

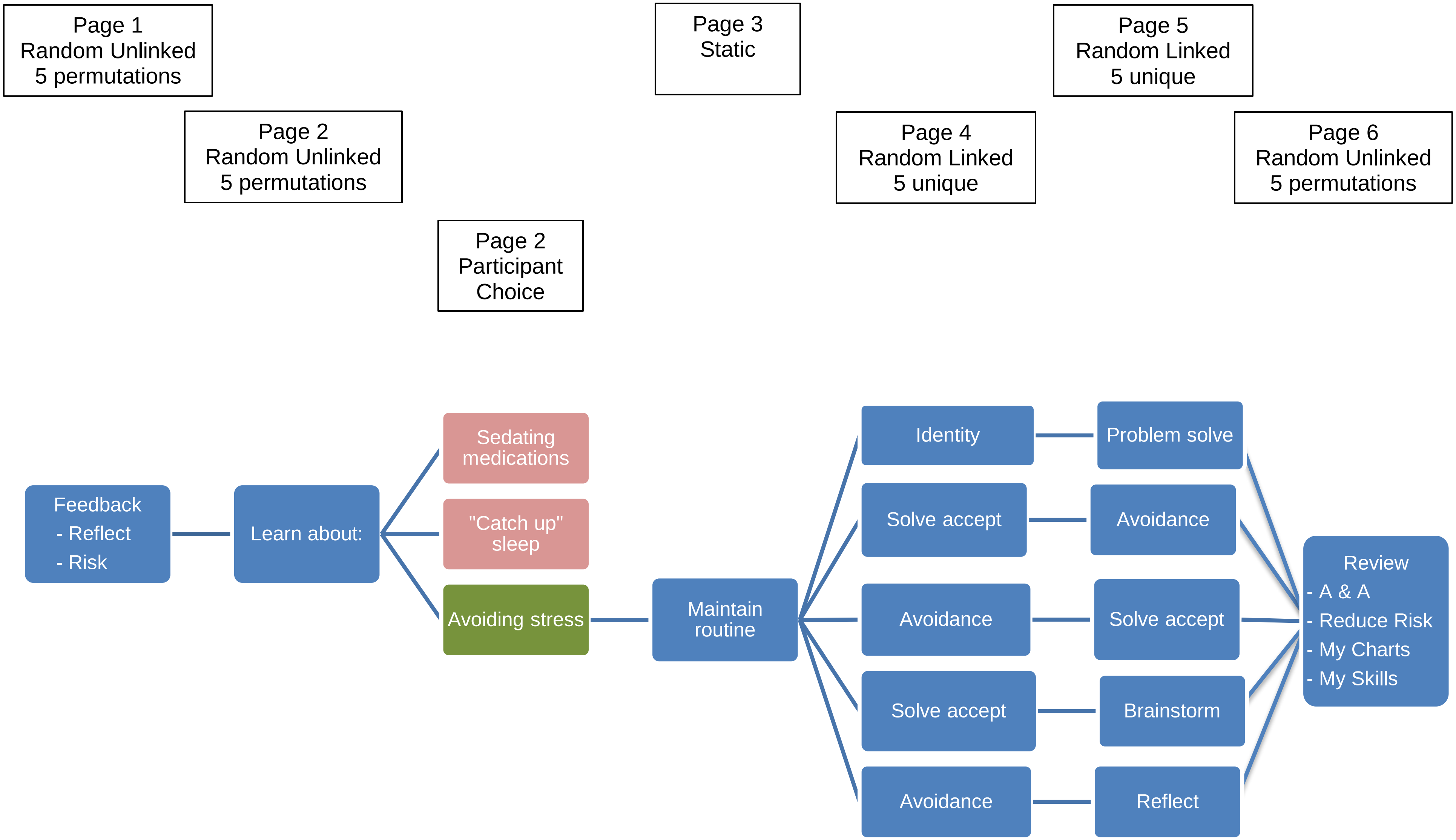

Daily Review Feedback Category 23: Moderate Risk – Sleeping Too Much, Avoiding Stress (Choice 3.0)

Reflect (P6)	Risk Reflect (P1)	Routine (S1)	Identity (U11)	Problem Solve (U11)	My Charts (P1)
**1**<br>**RANDOM UNLINKED**	**2**<br>**RANDOM UNLINKED**	**3**<br>**STATIC**	**4**<br>**RANDOM LINKED**	**5**<br>**RANDOM LINKED**	**6**<br>**RANDOM UNLINKED**
Glad to see you're doing well.<br><br>You may want, however, to take a look at your sleep patterns. It seems like you might be sleeping too much.<br><br>Press continue to learn more about sleeping well.	Sleeping too much, while it might feel good, can throw things off. It can trigger symptoms.<br><br>The reasons people sleep too much vary. Below are some common causes.<br><br>Before reading further, double---check your wellness rating. Sleeping a lot can be a symptom of depression. Is it possible you are down?<br><br>Read about...	For general health, it is important to get the right amount of sleep on a daily basis. Not too much sleep and not too little sleep.<br><br>Keep a regular sleep schedule:<br><br>• Turn in the same time each night<br>• Arise the same time each morning<br>• Avoid daytime napping<br><br>Following these guidelines is easier said than done.<br><br>Continue for tips ...	WHAT DOES IT MEAN?<br><br>What are your aims in life? What do you value?<br>How would you really like to spend your time?<br><br>Will sleeping less help you live more fully?<br>Will it leave you more time for desired activities?<br>How would life be different if you slept less, say 6 to 10 hours a night?<br><br>OVERCOMING BARRIERS<br><br>Don't forget your life aims and values. Act now to overcome any obstacles.<br><br>What can you do about the stress in your life?<br><br>Take action!	EXAMPLE<br><br>Say you've been sleeping 12 hours a day. You know that usually you only need 9 hours.<br><br>Life with less sleep:<br><br>• More time with spouse<br>• More time to exercise<br>• More time for friends<br><br>Solution:<br><br>You barely have enough money to pay the bills next month. Instead of sleeping, brainstorm all the possible ways you can balance your budget.	Check out the relationship between your sleep and wellness in My Charts in the Wellness Plan below.<br><br>Be well!

Daily Review Feedback Category 23: Moderate Risk – Sleeping Too Much, Avoiding Stress (Choice 3.0)

Reflect (P11)	Risk Reflect (P10)	Routine (S1)	Solve Accept (U12)	Avoidance (U12)	Reduce Risk (P7)
**1**<br>**RANDOM UNLINKED**	**2**<br>**RANDOM UNLINKED**	**3**<br>**STATIC**	**4**<br>**RANDOM LINKED**	**5**<br>**RANDOM LINKED**	**6**<br>**RANDOM UNLINKED**
Glad to see you're doing well.<br><br>Take a look at your sleep patterns in order to stay well!	Life can often feel like too much. Sleeping is a nice respite. It is relaxing. It is pleasant. It is a nice escape.<br><br>Unfortunately, sleeping too much can also trigger symptoms.<br><br>Now is a good time to double check your wellness rating. Sleeping a lot can be a symptom of depression. Is it possible you are down?<br><br>Below are other common reasons for sleeping too much. Read about…	For general health, it is important to get the right amount of sleep on a daily basis. Not too much sleep and not too little sleep.<br><br>Keep a regular sleep schedule:<br><br>• Turn in the same time each night<br>• Arise the same time each morning<br>• Avoid daytime napping<br><br>Following these guidelines is easier said than done.<br><br>Continue for tips …	MANAGING STRESS<br><br>For better or worse here is the data. Worry and avoidance worsen problems. Acceptance and problem solving alleviate problems.<br><br>While sleep might be a nice escape, consider the big picture.<br><br>• Do what you can to solve problems<br>• Do what you can to accept things you cannot change<br>• Get support and feedback about what is going on	EXAMPLE<br><br>Say you've been sleeping 12 hours a day. You know that usually you only need 9 hours.<br><br>Take a look at your life. Is there some problem you are avoiding? Consider:<br><br>• Self-care<br>• Home-care<br>• Relationships<br>• School<br>• Work<br><br>Are there any problems you need to address?	Read about your sleep plans in Reduce Risk in the Wellness Plan below.<br><br>Stay well.

Daily Review Feedback Category 23: Moderate Risk – Sleeping Too Much, Avoiding Stress (Choice 3.0)

Reflect (P12)	Risk Reflect (P11)	Routine (S1)	Avoidance (U13)	Solve Accept (U13)	A & A (P8)
**1**<br>**RANDOM UNLINKED**	**2**<br>**RANDOM UNLINKED**	**3**<br>**STATIC**	**4**<br>**RANDOM LINKED**	**5**<br>**RANDOM LINKED**	**6**<br>**RANDOM UNLINKED**
You're saying that you are well. That's good.<br><br>Take a look at your sleep patterns.	Sleeping too much throws things off, and it can trigger symptoms. The reasons people sleep too much vary.<br><br>Below are some common causes.<br><br>Before considering these reasons, double---check your wellness rating. Sleeping a lot can be a symptom of depression. Is it possible you are down?<br><br>Read about…	For general health, it is important to get the right amount of sleep on a daily basis. Not too much sleep and not too little sleep.<br><br>Keep a regular sleep schedule:<br><br>• Turn in the same time each night<br>• Arise the same time each morning<br>• Avoid daytime napping<br><br>Following these guidelines is easier said than done.<br><br>Continue for tips …	Avoidance is a very common coping strategy. People avoid problems by procrastinating, denying, focusing on other things, and sleeping.<br><br>Maybe you don't think you can cope. It is sometimes relieving to just "turn off".<br><br>Unfortunately, avoidance (including just sleeping) worsens problems. You know this intuitively.	CONSIDER THIS<br><br>Take some time and consider the stressors you are facing. Write it out.<br><br>Is there a problem you can solve? How can you get started? Take small steps.<br><br>Is there a problem you cannot solve? How do you accept the reality of what is going on?	Sleeping too much can be an early warning sign.<br><br>Double check for symptoms of depression in Awareness & Action in the Wellness Plan below.<br><br>Stay well.

Daily Review Feedback Category 23: Moderate Risk – Sleeping Too Much, Avoiding Stress (Choice 3.0)

Reflect (P13)	Risk Reflect (P12)	Routine (S1)	Solve Accept (U14)	Brainstorm (U14)	My Skills (P4)

1<br>**RANDOM UNLINKED**	2<br>**RANDOM UNLINKED**	3<br>**STATIC**	4<br>**RANDOM LINKED**	5<br>**RANDOM LINKED**	6<br>**RANDOM UNLINKED**
Good thing to see you're doing well.<br><br>Consider your sleep patterns. It looks like you might be sleeping too much.	You got this alert because you have been sleeping more than usual lately. Remember that sleeping too much can cause problems.<br><br>Before considering the common reasons for sleeping too much below, double--- check your wellness rating. Sleeping a lot can be a symptom of depression. Is it possible you are down?<br><br>Read about…	For general health, it is important to get the right amount of sleep on a daily basis. Not too much sleep and not too little sleep.<br><br>Keep a regular sleep schedule:<br><br>• Turn in the same time each night<br>• Arise the same time each morning<br>• Avoid daytime napping<br><br>Following these guidelines is easier said than done.<br><br>Continue for tips …	BUILD YOUR MENTAL MUSCLE<br><br>Stress can be hard to manage. Checking out by using drugs or other substances, throwing yourself into some task, or sleeping can all be tempting options.<br><br>In the end, as you know, you are faced with the stressor. What should you do?<br><br>• Solve problems you can solve.<br>• Accept problems you cannot solve.<br><br>Easier said than done, but it is possible!	CONSIDER THIS<br><br>Be creative. Take a look at what is going on in your life. What is problematic? What can you do?<br><br>Brainstorm. Come up with as many solutions as possible. Even solutions that seem ridiculous.<br><br>Then be more thoughtful. What are you able and willing to do in order to address your current problems?	Check out any sleep skills you added to your My Resources section of the Wellness Plan.<br><br>Stay well.

Daily Review Feedback Category 23: Moderate Risk – Sleeping Too Much, Avoiding Stress (Choice 3.0)

Reflect (P14)	Risk Reflect (P13)	Routine (S1)	Avoidance (U15)	Reflect (U15)	(FC22P5)
**1**<br>**RANDOM UNLINKED**	**2**<br>**RANDOM UNLINKED**	**3**<br>**STATIC**	**4**<br>**RANDOM LINKED**	**5**<br>**RANDOM LINKED**	**6**<br>**RANDOM UNLINKED**
You're reporting feeling well. That's great.<br><br>Take a closer look at your sleep. Changes in sleep can trigger symptoms.	Sleeping too little, too much, or just at variable times can be problematic. You are more vulnerable right now than you probably realize.<br><br>Sleeping too much throws things off. It can trigger symptoms.<br><br>Before considering the common reasons for sleeping too much below, double--- check your wellness rating. Sleeping a lot can be a symptom of depression. Is it possible you are down?<br><br>Read about…	For general health, it is important to get the right amount of sleep on a daily basis. Not too much sleep and not too little sleep.<br><br>Keep a regular sleep schedule:<br><br>• Turn in the same time each night<br>• Arise the same time each morning<br>• Avoid daytime napping<br><br>Following these guidelines is easier said than done.<br><br>Continue for tips …	DID YOU KNOW…<br><br>That trying not to think about something actually makes you think about it more?<br><br>Avoiding problems only weighs on you in the end.<br><br>The only way around this is to solve the problems you can solve and accept the problems you cannot solve.	TAKE CHARGE<br><br>Say you've been sleeping 12 hours a day. You know that you usually only need 9 hours.<br><br>Be honest with yourself. What is going on?<br><br>If there is a problem at home or school or work, what can you do? If you are uncertain, are you willing to get advice from your supports?	Stay well.

Daily Review Feedback Category 24: Moderate Risk – Sleeping Erratically

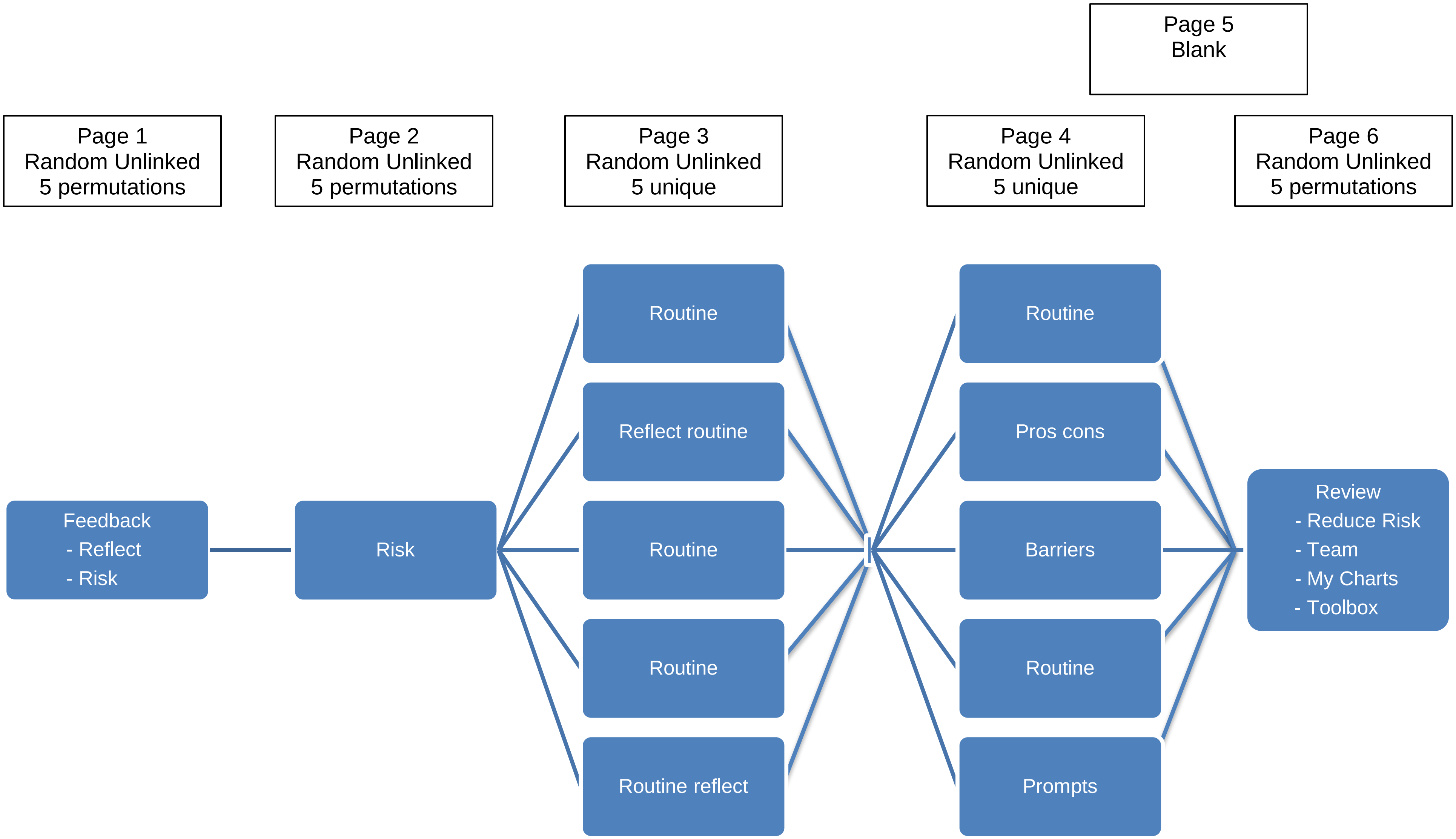

Daily Review Feedback Category 24: Moderate Risk – Sleeping Erratically

Reflect (P1)	Risk (P1)	Routine (U1)	Routine (U1)		My Charts (P1)
**1**<br>**RANDOM UNLINKED**	**2**<br>**RANDOM UNLINKED**	**3**<br>**RANDOM UNLINKED**	**4**<br>**RANDOM LINKED**	**5**<br>**BLANK**	**6**<br>**RANDOM UNLINKED**
Glad to see you're well.<br><br>You may, however, want to take a look at your sleep. It looks like your schedule has not been stable the last few days.<br><br>Press continue for tips on keeping a consistent sleep schedule…	Erratic sleep happens when your bed time and rise time are generally within your target window, but the amount of sleep you have been getting varies from night to night.<br><br>This pattern is not very good for you, as it can disrupt your internal clock. In turn, this can throw off biological systems that operate on a schedule, such as those that regulate hormone levels and body temperature.<br><br>Although not fully understood, disruptions to your internal clock can trigger symptoms of mania and depression.	SETTING GOALS<br><br>Say you find yourself turning in at all different times. Sometimes you're up working late at night. Sometimes you're tired and fall asleep quite early.<br><br>How can you develop a consistent sleep schedule? Pick a time to turn in that is reasonable given your life demands. Then pick a time to get up that is reasonable given your sleep needs.<br><br>• Turn in 11:00 p.m.<br>• Get up 8:00 a.m.<br><br>Check if your goal is realistic. If you've been turning in at 4:00 a.m. and need to be at work by 9:00 a.m., it is not realistic.	TIP OF THE DAY<br><br>Successful behavior change involves breaking old habits, overcoming temptations, and dealing with distractions.<br><br>Be sure you plan your entire evening out in a way that will allow you to turn in at your target time. Be realistic about how much you can get done.<br><br>This should include <u>1 hour</u> of wind down time. Your body needs to know it is about to go to sleep. One hour before bedtime, turn the lights down low. Engage in a low stimulating activity such as reading or watching a television program that is not intense.		Check out the relationship between your sleep and wellness in My Charts in the Wellness Plan below.<br><br>Be well!

Daily Review Feedback Category 24: Moderate Risk – Sleeping Erratically

Reflect Risk (P2)	Barriers Risk (P2)	Reflect Routine (U2)	Pros Cons (U2)		Reduce Risk (P2)
**1**<br>**RANDOM UNLINKED**	**2**<br>**RANDOM UNLINKED**	**3**<br>**RANDOM UNLINKED**	**4**<br>**RANDOM LINKED**	**5**<br>**BLANK**	**6**<br>**RANDOM UNLINKED**
Good to see you're doing well.<br><br>Even so, take a few minutes now to consider your sleep schedule. It has not been very consistent lately, which puts you at risk for future symptoms.<br><br>Press continue to read more…	Significant fluctuations in the amount of sleep you are getting on a daily basis triggered this alert.<br><br>Lots of things can get in the way of getting more regular sleep. Life demands, noise, anxiety, competing desires (like staying up to watch a favorite television show), to name a few. The list goes on…<br><br>An erratic sleep pattern can trigger symptoms of mania and depression. Try your best to get consistent sleep each night.	THINK ABOUT IT<br><br>Do you prefer to live spontaneously? Do you think routines are boring?<br><br>If you answered yes to either of the questions above, is there a way to keep a regular sleep schedule AND live a life that feels natural?<br><br>Having fun is about what you do and not as much about when you do it. Can you make any changes to your schedule to protect your sleep time?	KNOW YOUR COSTS AND BENEFITS<br><br>Think…<br><br>What are the advantages in your mind of keeping a regular sleep schedule?<br><br>Research suggests you'll feel and do better.<br><br>What are the disadvantages of keeping a regular sleep schedule?<br><br>Is there a way for you to regulate your sleep routine in a way that will not be too costly?		Take a look at your sleep plan in Reduce Risk in the Wellness Plan.<br><br>Be well!

Daily Review Feedback Category 24: Moderate Risk – Sleeping Erratically

Reflect (P3)	Risk (P3)	Routine (U3)	Barriers (U3)		Toolbox (P3)
**1**<br>**RANDOM UNLINKED**	**2**<br>**RANDOM UNLINKED**	**3**<br>**RANDOM UNLINKED**	**4**<br>**RANDOM LINKED**	**5**<br>**BLANK**	**6**<br>**RANDOM UNLINKED**
Looks like you're doing well.<br><br>To keep this going, take a look at your sleep schedule. It seems like the amount of sleep you have been getting has varied quite a bit these past couple of days.<br><br>Continue to read more…	Fluctuations in the amount of sleep you are getting each night triggered this alert.<br><br>Did you notice this about your sleep before getting this alert?<br><br>In general, people tend to underestimate the importance of sleep. Irregular sleep impacts attention, concentration, memory, mood, emotional resilience, and even your body. For people with bipolar disorder, irregular sleep can even trigger symptoms.	GETTING STARTED<br><br>The time you get up and turn in are the most important anchor points in the day.<br><br>If your work or school schedule varies from day to day, find a chunk of time to sleep that will be consistent across days no matter your obligations. If your evening activities vary from day to day, again find a chunk of time to sleep that will be consistent across days.<br><br>Aim for as little variability as possible in your sleep. Ideally, one hour or less.	ANTICIPATING OBSTACLES<br><br>Anticipate any obstacles and come up with solutions. Common obstacles include:<br><br>• Staying out or up late on the weekends<br>• Having a work or school schedule that is different every day<br>• Watching certain television programs late at night<br><br>What can you do?<br>Ask for a different work schedule?<br>Record late evening television programs?<br>Turn in earlier on weekends?<br>Or get up earlier on the weekends?		Read more about sleep in Toolbox.<br><br>Be well!

Daily Review Feedback Category 24: Moderate Risk – Sleeping Erratically

Reflect (P4)	Risk (P4)	Routine (U4)	Routine (U4)		My Team (P4)
**1**<br>**RANDOM UNLINKED**	**2**<br>**RANDOM UNLINKED**	**3**<br>**RANDOM UNLINKED**	**4**<br>**RANDOM LINKED**	**5**<br>**BLANK**	**6**<br>**RANDOM UNLINKED**
Seems like you've been doing well. That's great news.<br><br>Consider getting more consistent sleep in order to stay well.<br><br>Continue to learn more about sleep schedules…	Significant fluctuations in the hours of sleep you are getting triggered this sleep alert.<br><br>Having a regular sleep schedule promotes mental and physical health.<br><br>Changes in sleep throw off your internal clock. This, in turn, can trigger symptoms.	THINK ABOUT IT<br><br>Routines always shift some. Life happens.<br><br>Aim to regulate your sleep routine to the greatest extent possible.<br><br>A good routine consists of a day that is anchored. That means doing the following about the same time every day:<br><br>• Getting up<br>• Seeing others for the first time<br>• Starting home care/work/school<br>• Eating dinner<br>• Turning in<br><br>What can you do to anchor your sleep?	YOU CAN DO IT<br><br>Regulating sleep can seem impossible. So many things can get in the way. And falling asleep is not something you can consciously force yourself to do.<br><br>Try not to fret. It is possible to get your sleep schedule into a good rhythm.<br><br>Start by getting up at the same time every day, no matter what. Avoid naps. And then try to turn in about the same time every night. And if you cannot fall right to sleep, don't worry. Get up and do something relaxing.		Reach out to your supports for help getting your sleep back on track.<br><br>Ask for their feedback and honestly consider what they have to say.<br><br>Be well!

Daily Review Feedback Category 24: Moderate Risk – Sleeping Erratically

Reflect (P5)	Risk Reflect (P5)	Routine Reflect (U5)	Prompts (U5)		Toolbox (P5)
**1**<br>**RANDOM UNLINKED**	**2**<br>**RANDOM UNLINKED**	**3**<br>**RANDOM UNLINKED**	**4**<br>**RANDOM LINKED**	**5**<br>**BLANK**	**6**<br>**RANDOM UNLINKED**
It's great to see that you're doing well.<br><br>However, it appears that you may be experiencing day---to---day fluctuations in the amount of sleep you have been getting.<br><br>Press continue to read more…	Significant fluctuations in the hours of sleep you are getting triggered this sleep alert.<br><br>You are putting yourself at risk for symptoms, so take some time now to look at what's going on.<br><br>Consider what needs to happen in order for you to get consistent sleep each night.	REMEMBER…<br><br>There are 5 events that anchor your day:<br><br>• Getting up<br>• First seeing others<br>• Starting work<br>• Eating dinner<br>• Turning in<br><br>Are you having problems with one or more of the areas above? Is there something you are willing and able to do to steady your sleep schedule?	USING CUES<br><br>Your body responds to cues. This includes the natural functions like eating and sleeping that happen within the 24---hour day.<br><br>You can use cues to train your body into a more restive state that is conducive for sleep.<br><br>About 1 hour before the time you hope to fall asleep:<br><br>• Do turn down the lights in your home<br>• Don't engage in intense activities<br><br>When you get in bed:<br><br>• Do turn off the lights<br>• Don't bring your phone, tablet, or computer with you		Learn more about making and sticking with behavior change plans in Building Skills in Toolbox.<br><br>Be well!

Daily Review Feedback Category 25: Moderate Risk – Irregular Routine

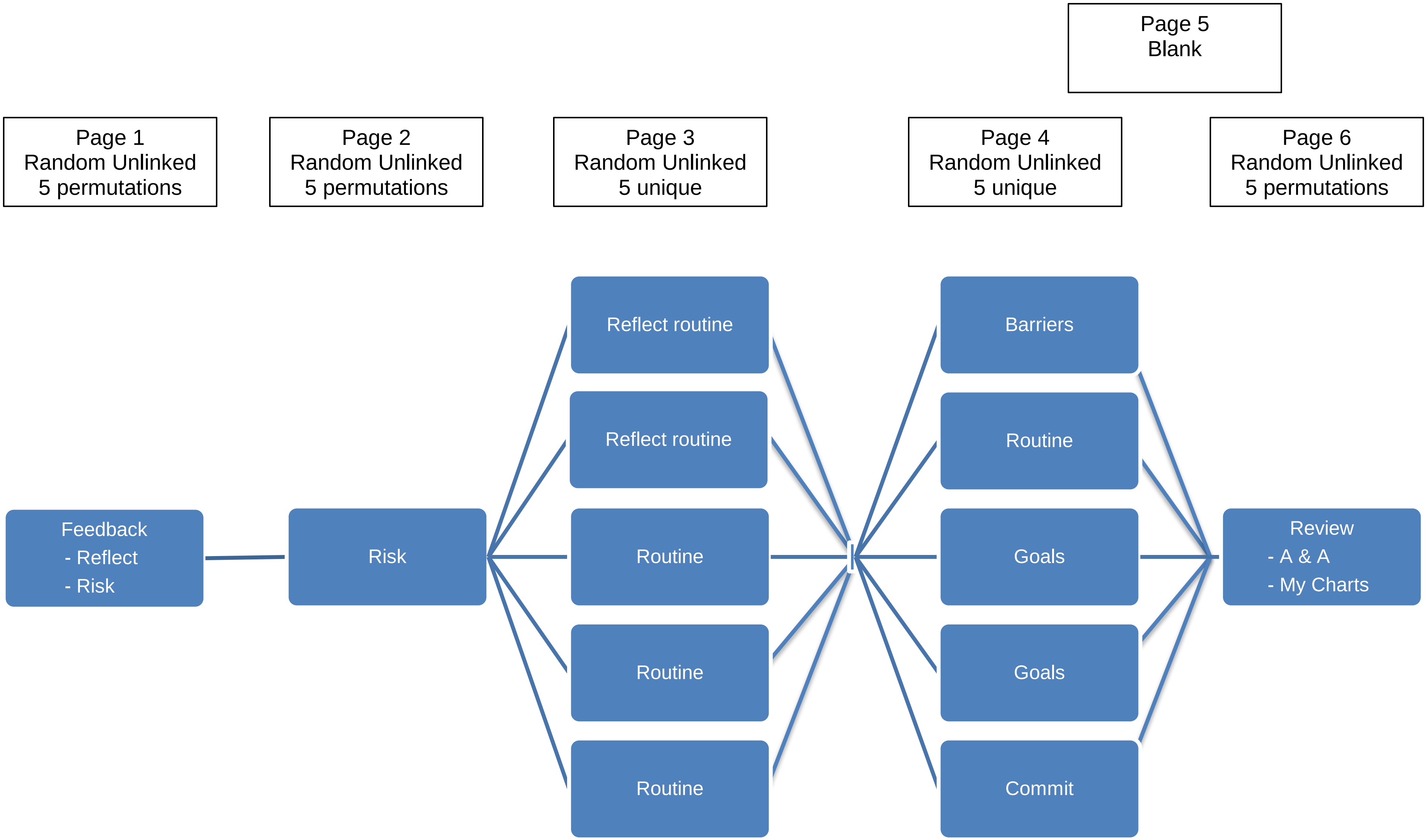

Daily Review Feedback Category 25: Moderate Risk – Irregular Routine

Reflect (P1)	Risk (P1)	Reflect Routine (U1)	Barriers (U1)		My Charts (P1)
**1**<br>**RANDOM UNLINKED**	**2**<br>**RANDOM UNLINKED**	**3**<br>**RANDOM UNLINKED**	**4**<br>**RANDOM LINKED**	**5**<br>**BLANK**	**6**<br>**RANDOM UNLINKED**
Glad to see you're well.<br><br>You may, however, want to take a look at your routine. It looks like your schedule has not been stable the last few days.<br><br>Press continue to learn about routines...	Significant variation across days when you are active and inactive has triggered this Routine Alert.<br><br>Remember that having a regular routine, or rhythm, helps set your internal clock. This keeps systems that operate on a schedule, such as changes in hormone levels and body temperature, in order.<br><br>Although it is not fully understood, disruptions in internal clocks can trigger symptoms of mania and depression.	ADJUST!<br><br>Consider the past 4 days. What time did you get up, first see others, start your day, eat dinner, and turn in? Write down what you remember as best you can.<br><br>Are any of these 5 anchors changing by more than an hour from day to day? This may be what is triggering the Routine Alert for you.<br><br>Set times for these 5 daily transitions:<br><br>• Getting up<br>• Seeing others for first time<br>• Starting home care, work, school<br>• Eating dinner<br>• Turning in<br><br>Aim to get within 30 minutes of your target time for each.	OVERCOMING BARRIERS<br><br>What might get in the way of sticking to your schedule? Anticipate any potential barriers. Plan for how to overcome them before your day even starts.<br><br>Barriers you can plan for include:<br><br>• Old habits<br>• Distractions<br>• Competing impulses<br>• Temptations<br><br>Of course sometimes unanticipated things can throw off your schedule. For example, a traffic jam might leave you late getting home and having dinner.<br><br>Don't worry so much about the unexpected. That happens. But do consider and plan for how to overcome easy to anticipate barriers.		Check out the relationship between your routine and wellness in My Charts in the Wellness Plan below.<br><br>Be well!

Daily Review Feedback Category 25: Moderate Risk – Irregular Routine

Reflect (P2)	Routine (P2)	Reflect Routine (U2)	Routine (U2)		A & A (P2)
**1**<br>**RANDOM UNLINKED**	**2**<br>**RANDOM UNLINKED**	**3**<br>**RANDOM UNLINKED**	**4**<br>**RANDOM LINKED**	**5**<br>**BLANK**	**6**<br>**RANDOM UNLINKED**
Good to see you're doing well.<br><br>You may want to take a look at your schedule. It looks like your routine has been rather variable.<br><br>Press continue to learn more.	Significant variation across days when you are active and inactive has triggered this Routine Alert.<br><br>Aim to anchor your days. This will anchor your biology. That means to be active and inactive, in motion and at rest, at roughly the same time every day.	THINK ABOUT IT<br><br>Do you think routines are boring?<br><br>Consider your anchor points like you would vegetables and protein. A basic part of your diet (day). Spice it up with activities that:<br><br>• Give you a sense of mastery<br>• Give you a sense of pleasure<br>• Rely on your strengths<br>• Are consistent with the things you value in life.<br><br>Do you think routines are impossible?<br><br>Set an alert for 30 minutes before each anchor in your day. When the alert goes off start to transition to the next activity.	EXAMPLE<br><br>Say you like to stay flexible and spontaneous. That makes sense. A lot of people enjoy that way of life. Find a way to go with the flow, while also having some regularity to your schedule.<br><br>Start with the easiest anchor for you. Maybe having dinner at the roughly the same time each night is easiest. Start there.<br><br>Set an alarm thirty minutes before your target dinner hour. When the alarm goes off, find a way to stop what you're doing and start preparing your meal.<br><br>Keep your life interesting by doing different things each day, not the same things at different times!		Review your plan for routine in Awareness & Action in the Wellness Plan.<br><br>Be well…

Daily Review Feedback Category 25: Moderate Risk – Irregular Routine

Reflect (P3)	Risk (P3)	Routine (U3)	Goals (U3)		Toolbox (P3)
**1**<br>**RANDOM UNLINKED**	**2**<br>**RANDOM UNLINKED**	**3**<br>**RANDOM UNLINKED**	**4**<br>**RANDOM LINKED**	**5**<br>**BLANK**	**6**<br>**RANDOM UNLINKED**
Looks like you’re well. That good.<br><br>Take a few minutes to consider your routine. It has been shifting around quite a bit the past few days.<br><br>Press continue to learn more...	Significant variation across days when you are active and inactive has triggered this Routine Alert.<br><br>Do you feel any symptoms coming on? Remember that disruptions in routine can trigger mania and depression.	GETTING STARTED<br><br>The time you get up and turn in are the most important anchor points in the day.<br><br>It is easy for the timing of sleep to get off. Life demands, having fun, stress, too much caffeine, noise, etc. The list could go on and on.<br><br>Start with getting up at the same time every day. The rest will fall in place.<br><br>Even though you may end up being tired one day, this is the quickest way to correct a sleep schedule.	EVERYTHING COUNTS<br><br>Starting is the hardest part!<br><br>Whenever you set out to make changes, be sure your goal is realistic. After that, just get started.<br><br>When you are trying to change something, give yourself credit for every step no matter how small.<br><br>If you are trying to get up at 8:00 a.m., give yourself credit the first few days for getting up even if you get right back in bed.		Learn about making effective behavioral changes in Building Skills in Toolbox.<br><br>Stay well!

Daily Review Feedback Category 25: Moderate Risk – Irregular Routine

Risk (P4)	Risk (P4)	Routine (U4)	Goals (U4)		(P4)
**1**<br>**RANDOM UNLINKED**	**2**<br>**RANDOM UNLINKED**	**3**<br>**RANDOM UNLINKED**	**4**<br>**RANDOM LINKED**	**5**<br>**BLANK**	**6**<br>**RANDOM UNLINKED**
You're doing well overall, but it seems like your schedule has been fluctuating a lot lately.<br><br>This could put you at risk for symptoms down the road.<br><br>Press continue to learn more about keeping a regular routine.	Significant variation across days when you are active and inactive has triggered this Routine Alert.<br><br>Keeping a regular routine— and it doesn't have to be rigid—will promote wellness.	THINK ABOUT IT<br><br>Routines always shift some. Life happens.<br><br>Aim to regulate your routine to the greatest extent possible.<br><br>A good routine consists of a day that is anchored. This means doing the following about the same time every day:<br><br>• Getting up<br>• Seeing others for the first time<br>• Starting home care/work/school<br>• Eating dinner<br>• Turning in<br><br>What points in your routine have been drifting?	SETTING GOALS<br><br>Consider making your routine more regular. Pick one or two anchors.<br><br>• Get up<br>• See others<br>• Start work<br>• Eat dinner<br>• Turn in<br><br>Make your goal ARTfully:<br><br><u>A</u>ction-based. It should involve a behavior not a wish. For example, say you'll lie down at 10:00 each night, not that you'll fall asleep earlier. You cannot control when you fall asleep.<br><br><u>R</u>ealistic. Make sure you have a 95% chance of success. If you're turning in at 2:00 a.m. on average it is not realistic to expect to immediately shift to 10:00 p.m. Try 1:30 a.m. at first. Go step---wise.<br><br><u>T</u>imely. Say when you will engage in the desired behavior. Set a time and date.		Take good care of yourself. Be well!

Daily Review Feedback Category 25: Moderate Risk – Irregular Routine

Reflect Risk (P5)	Reflect (P5)	Routine (U5)	Commit (U5)		A & A (P5)
**1**<br>**RANDOM UNLINKED**	**2**<br>**RANDOM UNLINKED**	**3**<br>**RANDOM UNLINKED**	**4**<br>**RANDOM LINKED**	**5**<br>**BLANK**	**6**<br>**RANDOM UNLINKED**
It's great that you're doing well.<br><br>To keep it going, take some time now to consider your routine. It has been changing a lot day to day, which could put you at risk for symptoms.<br><br>Press continue to read more…	Significant variation across days when you are active and inactive has triggered this Routine Alert.<br><br>How have things been going? How have you been feeling?<br><br>Consider getting more regular with your schedule. It will help you stay well.	ZERO IN<br><br>There are 5 events that "anchor" your day.<br><br>• Getting up<br>• First seeing others<br>• Starting work<br>• Eating dinner<br>• Turning in<br><br>Which of these, if any, have been shifting?<br><br>Is there something you are willing and able to do to get your schedule steadier?	MAKE A PACT<br><br>Making a public commitment—in other words telling someone what you plan on doing—makes it more likely you will follow through.<br><br>What do you plan on doing to make your routine more regular?<br>Why not share this with a support and then call them in a few days and let them know how it is going?<br><br>Don't worry about being perfect. Make a goal and give it your best shot. If you succeed, great. If you do not, learn from your experience.		Review your anchors for Well in Awareness & Action in the Wellness Plan.<br><br>Use them as a motivator to get your routine back on track.<br><br>Be well!

Daily Review Feedback Category 26: Staying Well, Awareness - Early Warning Signs (Choice 1.1)

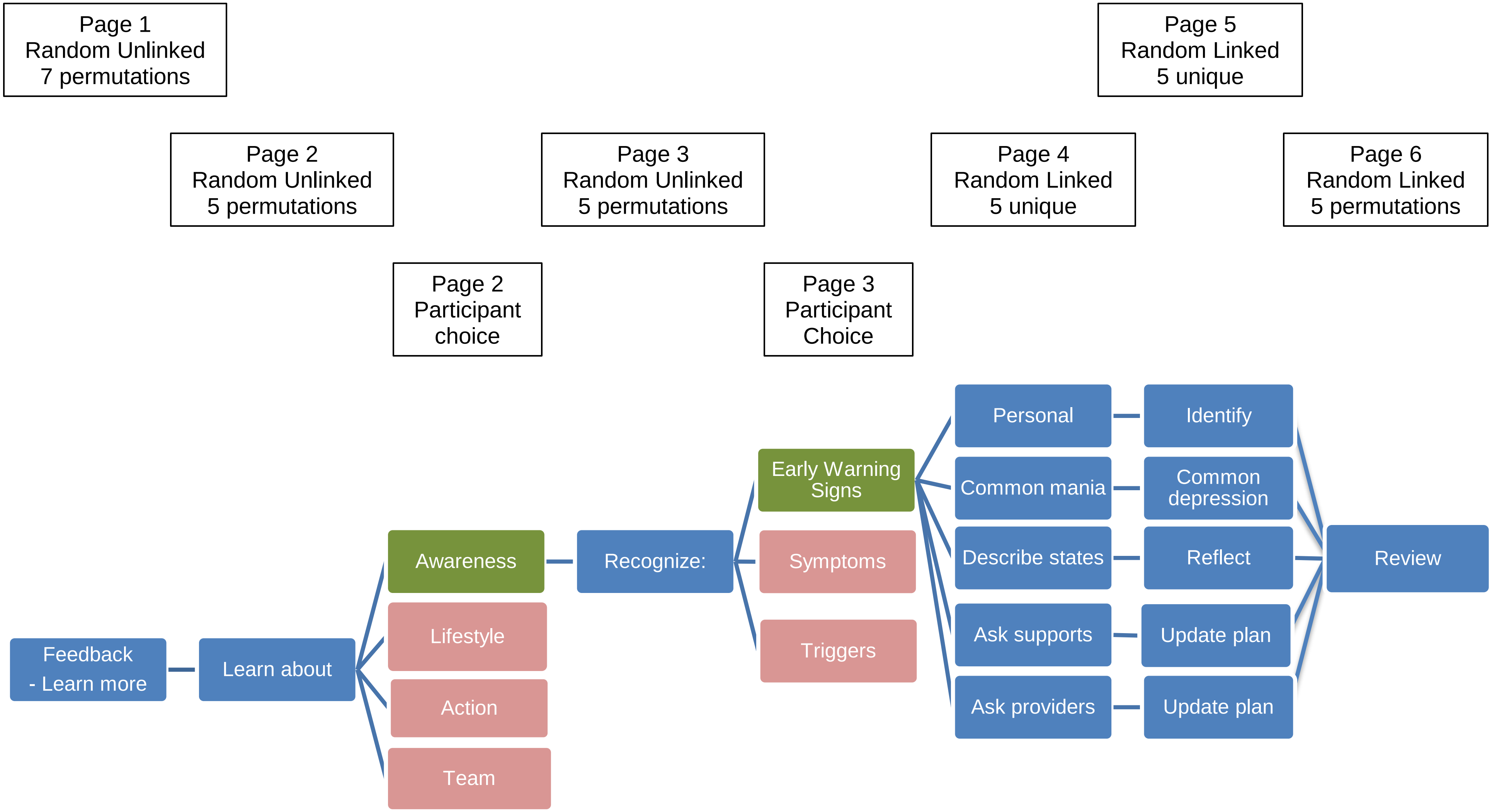

Daily Review Feedback Category 26: Staying Well, Awareness - Early Warning Signs (Choice 1.1)

Learn more (P1)	Learn about (P1)	Recognize (P1)	Personal (U1)	Identify (U1)	A & A (P1)
**1**<br>**RANDOM UNLINKED**	**2**<br>**RANDOM UNLINKED**	**3**<br>**RANDOM UNLINKED**	**4**<br>**RANDOM LINKED**	**5**<br>**RANDOM LINKED**	**6**<br>**RANDOM UNLINKED**
Glad to see you're doing well. That's great!<br><br>Press continue to learn more about staying well.	Learn more now when you're well. You will be ready to take action whenever things get off for you.<br><br>Read about...	Great choice! Awareness is crucial to good self-care. Without it, managing your life and illness becomes tricky.<br><br>Recognizing early warning signs, symptoms, and triggers allows you to change the course of things quickly.<br><br>Learn about...	Think about the last time you got a runny nose. What happened next? Did it just go away by itself? Was it an allergy? A reaction to slicing an onion? Did it turn into a full---blown cold?<br><br>A runny nose can be an early warning sign of a cold. That means it might, but also might not, be a sign that you are getting sick.<br><br>Similarly, you can have early signs of mania and depression. Although there are more and less common early warning signs, everyone is unique.<br><br>What are your early warning signs?	Even when you do everything "right"—like take your medications, sleep well, and keep up a good routine—you can get symptoms.<br><br>If you can identify the earliest warning signs, you have a better chance of heading off a full---blown mood episode.	Double check your early warning signs (mild up and down) listed in Awareness and Action in the Wellness Plan. Do you need to make any changes?<br><br>Stay well...

Daily Review Feedback Category 26: Staying Well, Awareness - Early Warning Signs (Choice 1.1)

Learn more (P2)	Learn about (P2)	Recognize (P2)	Mania (U2)	Depression (U2)	Toolbox (P2)
**1**<br>**RANDOM UNLINKED**	**2**<br>**RANDOM UNLINKED**	**3**<br>**RANDOM UNLINKED**	**4**<br>**RANDOM LINKED**	**5**<br>**RANDOM LINKED**	**6**<br>**RANDOM UNLINKED**
Good to see you're well.<br><br>Continue to learn more about staying well.	Read more about the keys to staying well.<br><br>Check out…	Awareness is one of the foundations of staying well.<br><br>Become more aware of…	Maybe you have already seen this list of common early warning signs of mania. What do you think? Are any of the following the <u>first</u> things that change when you're getting manic?<br><br>• Energetic/very active<br>• Feeling emotionally high<br>• More talkative<br>• Cannot get off to sleep<br>• Not needing much sleep<br>• Racing thoughts<br>• Ideas flowing too fast<br>• Difficulty concentrating<br>• Senses seem sharper	How about this list of common early warning signs of depression. What do you think? Are any of the following the <u>first</u> things that change when you're getting depressed?<br><br>• Low motivation/can't get started<br>• Low in energy<br>• Feeling tired/listless<br>• Loss of interest in activities<br>• Difficulty concentrating<br>• Want to be alone<br>• Less talkative<br>• Negative thoughts pop into my mind<br>• Loss of interest in people<br>• Ideas slowed down<br>• Less interest in sex<br>• Cannot get off to sleep<br>• Interrupted sleep	Check out the self-assessment section of Toolbox to learn even more about early warning signs.<br><br>Stay well…

Daily Review Feedback Category 26: Staying Well, Awareness - Early Warning Signs (Choice 1.1)

Learn more (P3)	Learn about (P3)	Recognize (P3)	Describe States (U3)	Reflect (U3)	My Skills (P3)
**1**<br>**RANDOM UNLINKED**	**2**<br>**RANDOM UNLINKED**	**3**<br>**RANDOM UNLINKED**	**4**<br>**RANDOM LINKED**	**5**<br>**RANDOM LINKED**	**6**<br>**RANDOM UNLINKED**
You say you're doing well. That's good.<br><br>Continue on to learn more about staying well.	Being aware, living a healthy lifestyle, coping with symptoms, and having a good team in place will help you stay well.<br><br>Read more about…	Good choice. Most people find it tricky to recognize early warning signs, symptoms, and triggers. However, with work it can be done.<br><br>Learn to recognize…	Think about what you are like as a person when you are depressed, well, and manic.<br><br>Take a piece of paper and make three columns. Title the left column "depressed", the middle column "well", and the right column "manic." Then record what you notice about yourself when you are depressed, well, and manic. Include the following:<br><br>• Symptoms<br>• Personality characteristics<br>• Outlook on life<br>• Self-care<br>• Social behaviors<br>• Work performance<br>• Interests<br><br>Look at your descriptions of depressed and manic. What changes occur first? These are early warning signs.	Did you learn anything about yourself from doing the exercise?<br><br>Are your early warning signs low-level symptoms?<br><br>Are your early warning signs subtle changes in how you think, feel, or act?	Have you saved any tips for identifying early warning signs to your Resources? Check them out in the Wellness Plan.<br><br>Stay well…

Daily Review Feedback Category 26: Staying Well, Awareness - Early Warning Signs (Choice 1.1)

Learn more (P4)	Learn about (P4)	Recognize (P4)	Ask Supports (U4)	Update (U4)	Foundations (P4)
**1**<br>**RANDOM UNLINKED**	**2**<br>**RANDOM UNLINKED**	**3**<br>**RANDOM UNLINKED**	**4**<br>**RANDOM LINKED**	**5**<br>**RANDOM LINKED**	**6**<br>**RANDOM UNLINKED**
Looks like you're doing well, which is great.<br><br>Continue to learn more about staying well.	Now is a good time to learn more about staying well. There are four areas on which you can focus.<br><br>Read about…	Awareness and action are important aspects of staying well.<br><br>Recognizing early warning signs, symptoms, and triggers allows you to take action quickly, before things get bad.<br><br>Learn to be more aware of…	Review the concept of early warning signs with your supports. Read over "Basic Facts about Bipolar Disorder" in the Foundations section of *LiveWell.*<br><br>Ask your supports what they first noticed about you before you got depressed the last time.<br><br>Ask your supports what they first noticed about you before you got manic the last time.<br><br>Sometimes others are quite good at detecting early warning signs. Consider the input of your supports.	What did your supports have to say? Any surprises?<br><br>If they identified any early warning signs you didn't know about, consider adding them to your wellness plan (mild up and mild down anchors).<br><br>Also consider sharing this information with your psychiatrist.	Review the lesson "Basic Facts about Bipolar Disorder" in Foundations.<br><br>Stay well…

Daily Review Feedback Category 26: Staying Well, Awareness - Early Warning Signs (Choice 1.1)

Learn more (P5)	Learn about (P5)	Recognize (P5)	Ask Providers (U5)	Update (U5)	(P5)
**1**<br>**RANDOM UNLINKED**	**2**<br>**RANDOM UNLINKED**	**3**<br>**RANDOM UNLINKED**	**4**<br>**RANDOM LINKED**	**5**<br>**RANDOM LINKED**	**6**<br>**RANDOM UNLINKED**
Great to see you're well.<br><br>Continue for strategies to help you stay on track.	There are four key areas to focus on in order to stay well.<br><br>Read about…	Excellent choice. As I'm sure you know, bipolar disorder can be a challenging illness to manage.<br><br>One of the best ways to stay healthy is to learn more about when your symptoms tend to develop and what this looks like for you.<br><br>Learn to recognize…	Ask your psychiatrist or therapist what they first noticed about you before you got depressed the last time.<br><br>Ask your psychiatrist or therapist what they first noticed about you before you got manic the last time.<br><br>Sometimes others are quite good at detecting early warning signs. Consider the input of your psychiatrist.	What did your psychiatrist or therapist have to say? Any surprises?<br><br>If they identified any early warning signs you didn't know about, consider adding them to your wellness plan (mild up and mild down anchors).<br><br>Also consider sharing this information with your supports.	Stay well…

Daily Review Feedback Category 26: Staying Well, Awareness - Symptoms (Choice 1.2)

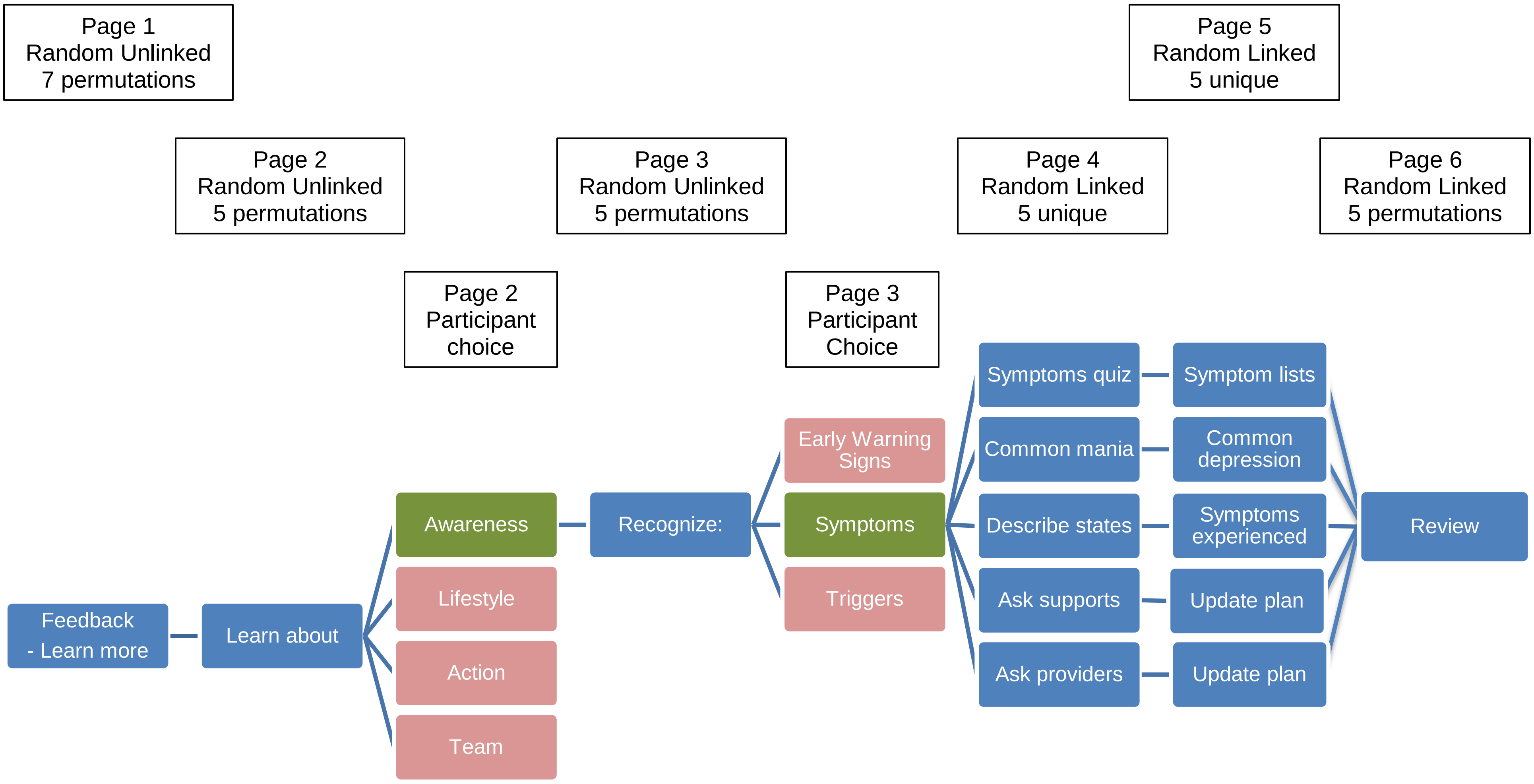

Daily Review Feedback Category 26: Staying Well, Awareness - Symptoms (Choice 1.2)

Learn more (P6)	Learn about (P1)	Recognize (P1)	Quiz (U6)	Lists (U6)	A & A (P6)
**1**<br>**RANDOM UNLINKED**	**2**<br>**RANDOM UNLINKED**	**3**<br>**RANDOM UNLINKED**	**4**<br>**RANDOM LINKED**	**5**<br>**RANDOM LINKED**	**6**<br>**RANDOM UNLINKED**
Looks like you're doing well. Good job.<br><br>Learn more about staying well.	Learn more now when you're well. You will be ready to take action whenever things get off for you.<br><br>Read about…	Great choice! Awareness is crucial to good self-care. Without it, managing your life and illness becomes tricky.<br><br>Recognizing early warning signs, symptoms, and triggers allows you to change the course of things quickly.<br><br>Learn about…	Quiz yourself:<br><br>There are nine key symptoms of depression. How many can you name?<br><br>There are eight key symptoms of mania. How many can you name?<br><br>HINT:<br><br>Even though bipolar disorder is considered a mood disorder, the symptoms involve more than just changes in feelings. They include changes in thinking and body processes as well.<br><br>Press continue for answers to the questions above.	Symptoms of depression:<br><br>✓✓ Depressed mood<br>✓✓ Reduced interest/pleasure in life<br>✓✓ Weight loss or weight gain<br>✓✓ Sleeping too much or too little<br>✓✓ Physical agitation or slowing down<br>✓✓ Fatigue or loss of energy<br>✓✓ Feeling worthless or guilty<br>✓✓ Difficulty concentrating<br>✓✓ Thoughts of death or suicide<br><br>Symptoms of mania:<br><br>✓✓ Feeling overly happy, exited, or irritable<br>✓✓ Increased esteem/feeling superior<br>✓✓ Decreased need for sleep<br>✓✓ More talkative than usual<br>✓✓ Racing thoughts<br>✓✓ Difficulties concentrating<br>✓✓ Increased activity level<br>✓✓ Risky activities	Double check your symptoms (moderate up and down) listed in Awareness and Action in the Wellness Plan. Do you need to make any changes?<br><br>Stay well…

Daily Review Feedback Category 26: Staying Well, Awareness - Symptoms (Choice 1.2)

Learn more (P7)	Learn about (P2)	Recognize (P2)	Mania (U7)	Depression (U7)	Toolbox (P7)
**1**<br>**RANDOM UNLINKED**	**2**<br>**RANDOM UNLINKED**	**3**<br>**RANDOM UNLINKED**	**4**<br>**RANDOM LINKED**	**5**<br>**RANDOM LINKED**	**6**<br>**RANDOM UNLINKED**
Nice to see you're well.<br><br>Learn more about staying well. Continue…	Read more about the keys to staying well.<br><br>Check out…	Awareness is one of the foundations of staying well.<br><br>Become more aware of…	Remember the common symptoms of mania? Which ones do you get when you're up?<br><br>• Feeling overly happy, exited, or irritable<br>• Increased esteem/feeling superior<br>• Decreased need for sleep<br>• More talkative than usual<br>• Racing thoughts<br>• Difficulties concentrating<br>• Increased activity level<br>• Risky activities	Remember the common symptoms of depression? Which ones do you get when you're down?<br><br>• Depressed mood<br>• Reduced interest/pleasure in life<br>• Weight loss or weight gain<br>• Sleeping too much or too little<br>• Physical agitation or slowing down<br>• Fatigue or loss of energy<br>• Feeling worthless or guilty<br>• Difficulty concentrating<br>• Thoughts of death or suicide	Check out the self--- assessment section of Toolbox to learn even more about symptoms.<br><br>Stay well…

Daily Review Feedback Category 26: Staying Well, Awareness - Symptoms (Choice 1.2)

Learn more (P3)	Learn about (P3)	Recognize (P3)	Describe States (U8)	Experienced (U8)	My Team (P8)
**1**<br>**RANDOM UNLINKED**	**2**<br>**RANDOM UNLINKED**	**3**<br>**RANDOM UNLINKED**	**4**<br>**RANDOM LINKED**	**5**<br>**RANDOM LINKED**	**6**<br>**RANDOM UNLINKED**
You say you're doing well. That's good.<br><br>Continue on to learn more about staying well.	Being aware, living a healthy lifestyle, coping with symptoms, and having a good team in place will help you stay well.<br><br>Read more about…	Good choice. Most people find it tricky to recognize early warning signs, symptoms, and triggers. However, with work it can be done.<br><br>Learn to recognize…	Think about what you are like as a person when you are depressed, well, and manic.<br><br>Take a piece of paper and make three columns. Title the left column "depressed", the middle column "well", and the right column "manic." Then record what you notice about yourself when you are depressed, well, and manic. Include the following:<br><br>• Personality characteristics<br>• Outlook on life<br>• Personal hygiene /Self---care<br>• Social interactions<br>• Work performance<br>• Interests<br><br>Press continue to review the symptoms of depression and mania.	How do changes in how you think, feel, and act line up with the symptoms of depression and mania?<br><br>Which symptoms do you generally experience?<br><br>Depression:<br><br>✓✓ Depressed mood<br>✓✓ Reduced interest/pleasure in life<br>✓✓ Weight loss or weight gain<br>✓✓ Sleeping too much or too little<br>✓✓ Physical agitation or slowing down<br>✓✓ Fatigue or loss of energy<br>✓✓ Feeling worthless or guilty<br>✓✓ Difficulty concentrating<br>✓✓ Thoughts of death or suicide<br><br>Mania:<br><br>✓✓ Feeling overly happy, exited, or irritable<br>✓✓ Increased esteem/feeling superior<br>✓✓ Decreased need for sleep<br>✓✓ More talkative than usual<br>✓✓ Racing thoughts<br>✓✓ Difficulties concentrating<br>✓✓ Increased activity level<br>✓✓ Risky activities	Remember that not everyone with bipolar disorder experiences the same symptoms. Have a good understanding of your symptoms and sharing this information with your team can help prevent future episodes.<br><br>Stay well!

Daily Review Feedback Category 26: Staying Well, Awareness - Symptoms (Choice 1.2)

Learn more (P4)	Learn about (P4)	Recognize (P4)	Ask Supports (U9)	Update (U9)	My Charts (P9)
**1**<br>**RANDOM UNLINKED**	**2**<br>**RANDOM UNLINKED**	**3**<br>**RANDOM UNLINKED**	**4**<br>**RANDOM LINKED**	**5**<br>**RANDOM LINKED**	**6**<br>**RANDOM UNLINKED**
Looks like you're doing well, which is great.<br><br>Continue to learn more about staying well.	Now is a good time to learn more about staying well. There are four areas on which you can focus.<br><br>Read about…	Awareness and action are important aspects of staying well.<br><br>Recognizing early warning signs, symptoms, and triggers allows you to take action quickly, before things get bad.<br><br>Learn to be more aware of…	Review the symptoms of mania and depression with your supports. Read over "Basic Facts about Bipolar Disorder" in the Foundations section of *LiveWell.*<br><br>Ask your supports what they notice about you when you get depressed.<br><br>Ask your supports what they notice about you when you get manic.<br><br>Sometimes others are quite good at identifying symptoms. Consider the input of your supports.	What did your supports have to say? Any surprises?<br><br>If they identified any symptoms of which you were unaware, consider adding them to your wellness plan (moderate up and down).<br><br>Also consider sharing this information with your psychiatrist.	Take a look at My Charts in the Wellness Plan. Do you notice any patterns?<br><br>Be well.

Daily Review Feedback Category 26: Staying Well, Awareness - Symptoms (Choice 1.2)

Learn more (P5)	Learn about (P5)	Recognize (P5)	Ask Providers (U10)	Update (U10)	(P5)
**1**<br>**RANDOM UNLINKED**	**2**<br>**RANDOM UNLINKED**	**3**<br>**RANDOM UNLINKED**	**4**<br>**RANDOM LINKED**	**5**<br>**RANDOM LINKED**	**6**<br>**RANDOM UNLINKED**
Great to see you're well.<br><br>Continue for strategies to help you stay on track.	There are four key areas to focus on in order to stay well.<br><br>Read about…	Excellent choice. As I'm sure you know, bipolar disorder can be a challenging illness to manage.<br><br>One of the best ways to stay healthy is to learn more about when your symptoms tend to develop and what this looks like for you.<br><br>Learn to recognize…	Ask your psychiatrist or therapist what they notice about you when you get depressed. What symptoms do they see?<br><br>Ask your psychiatrist or therapist what they notice about you when you get manic? What symptoms do they see?<br><br>Consider the input of your psychiatrist or therapist.	What did your psychiatrist or therapist have to say? Any surprises?<br><br>If they identified any symptoms of which you were unaware, consider adding them to your wellness plan (moderate up and down).<br><br>Also consider sharing this information with your supports.	Stay well…

Daily Review Feedback Category 26: Staying Well, Awareness - Triggers (Choice 1.3)

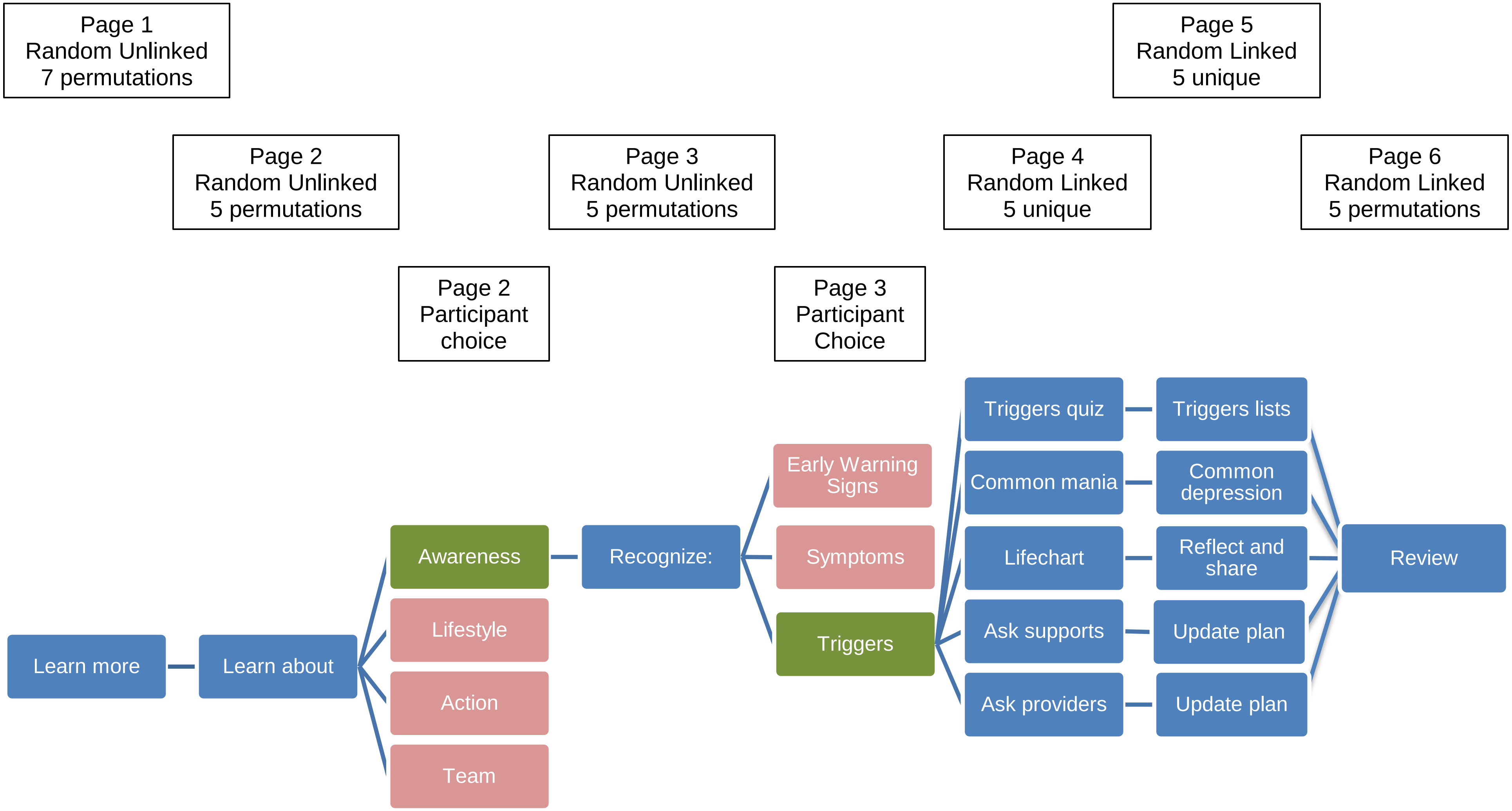

Daily Review Feedback Category 26: Staying Well, Awareness - Triggers (Choice 1.3)

Learn more (P1)	Learn about (P1)	Recognize (P1)	Quiz (U11)	Lists (U11)	Reduce Risk (P10)
**1**<br>**RANDOM UNLINKED**	**2**<br>**RANDOM UNLINKED**	**3**<br>**RANDOM UNLINKED**	**4**<br>**RANDOM LINKED**	**5**<br>**RANDOM LINKED**	**6**<br>**RANDOM UNLINKED**
Glad to see you're doing well. That's great!<br><br>Press continue to learn more about staying well.	Learn more now when you're well. You will be ready to take action whenever things get off for you.<br><br>Read about…	Great choice! Awareness is crucial to good self-care. Without it, managing your life and illness becomes tricky.<br><br>Recognizing early warning signs, symptoms, and triggers allows you to change the course of things quickly.<br><br>Learn about…	Quiz yourself:<br><br>What do you think might be common triggers for depression and mania?<br><br>Take a few minutes to first carefully consider this question.<br><br>When you're ready, press continue for the answer.	Common triggers for depression:<br><br>• Low social support<br>• Negative life events<br>• Critical family interactions<br><br>Common triggers for mania:<br><br>• Stress<br>• Over---involvement in activities<br>• Sleep disruptions<br>• Schedule disruptions	Double check your plans for Reduce Risk in the Wellness Plan. Make sure you are actively addressing any trigger areas. Do you need to make any changes?<br><br>Stay well…

Daily Review Feedback Category 26: Staying Well, Awareness - Triggers (Choice 1.3)

Learn more (P2)	Learn about (P2)	Recognize (P2)	Mania (U12)	Depression (U12)	Toolbox (P11)
**1**<br>**RANDOM UNLINKED**	**2**<br>**RANDOM UNLINKED**	**3**<br>**RANDOM UNLINKED**	**4**<br>**RANDOM LINKED**	**5**<br>**RANDOM LINKED**	**6**<br>**RANDOM UNLINKED**
Good to see you're well.<br><br>Continue to learn more about staying well.	Read more about the keys to staying well.<br><br>Check out…	Awareness is one of the foundations of staying well.<br><br>Become more aware of…	There are common triggers for mania. Which ones trigger you?<br><br>• Stress<br>• Over---involvement in activities<br>• Sleep disruptions<br>• Schedule disruptions	There are common triggers for depression. Which ones trigger you?<br><br>• Low social support<br>• Negative life events<br>• Critical family interactions	Check out the self---assessment section of Toolbox to learn even more about triggers.<br><br>Stay well…

Daily Review Feedback Category 26: Staying Well, Awareness - Triggers (Choice 1.3)

Learn more (P3)	Learn about (P3)	Recognize (P3)	Lifechart (U13)	Reflect (U13)	My Charts (P9)
**1**<br>**RANDOM UNLINKED**	**2**<br>**RANDOM UNLINKED**	**3**<br>**RANDOM UNLINKED**	**4**<br>**RANDOM LINKED**	**5**<br>**RANDOM LINKED**	**6**<br>**RANDOM UNLINKED**
You say you're doing well. That's good.<br><br>Continue on to learn more about staying well.	Being aware, living a healthy lifestyle, coping with symptoms, and having a good team in place will help you stay well.<br><br>Read more about…	Good choice. Most people find it tricky to recognize early warning signs, symptoms, and triggers. However, with work it can be done.<br><br>Learn to recognize…	Chart out your lifetime symptoms. Take a piece of paper and turn it on its side. Draw a line across the middle. At the left end write "onset" (which means first symptoms). At the right end, write "now".<br><br>Make a note of any past or current mood episodes. Episodes of depression are drawn below the baseline and episodes of mania or hypomania are drawn above the baseline. For each episode record your:<br><br>1. Age<br>2. Use of alcohol and street drugs<br>3. Major life events<br>4. Treatments	Examine your chart. Is there a connection between:<br><br>• Substance use and episodes?<br>• Life events and episodes?<br>• Medications and episodes?<br><br>Did you learn anything about your triggers from this exercise?<br><br>If so, share this information with your psychiatrist and supports.	Take a look at My Charts in the Wellness Plan. Do you notice any patterns?<br><br>Be well.

Daily Review Feedback Category 26: Staying Well, Awareness - Triggers (Choice 1.3)

Learn more (P4)	Learn about (P4)	Recognize (P4)	Ask supports (U14)	Update (U14)	Foundations (P4)
**1**<br>**RANDOM UNLINKED**	**2**<br>**RANDOM UNLINKED**	**3**<br>**RANDOM UNLINKED**	**4**<br>**RANDOM LINKED**	**5**<br>**RANDOM LINKED**	**6**<br>**RANDOM UNLINKED**
Looks like you're doing well, which is great.<br><br>Continue to learn more about staying well.	Now is a good time to learn more about staying well. There are four areas on which you can focus.<br><br>Read about…	Awareness and action are important aspects of staying well.<br><br>Recognizing early warning signs, symptoms, and triggers allows you to take action quickly, before things get bad.<br><br>Learn to be more aware of…	Review the lists of triggers with your supports. Read over "Basic Facts about Bipolar Disorder" in the Foundations section of *LiveWell.*<br><br>Ask your supports if they notice things that trigger your depression.<br><br>Ask your supports if they notice things that trigger your mania.<br><br>Sometimes others are quite good at identifying triggers. Consider the input of your supports.	What did your supports have to say? Any surprises?<br><br>If they identified any triggers of which you were unaware, consider addressing them in your wellness plan (see reduce risk).<br><br>Also consider sharing this information with your psychiatrist.	Review the lesson "Basic Facts about Bipolar Disorder" in Foundations.<br><br>Stay well…

Daily Review Feedback Category 26: Staying Well, Awareness - Triggers (Choice 1.3)

Learn more (P5)	Learn about (P5)	Recognize (P5)	Ask providers (U15)	Update (U15)	(P5)
**1**<br>**RANDOM UNLINKED**	**2**<br>**RANDOM UNLINKED**	**3**<br>**RANDOM UNLINKED**	**4**<br>**RANDOM LINKED**	**5**<br>**RANDOM LINKED**	**6**<br>**RANDOM UNLINKED**
Great to see you're well.<br><br>Continue for strategies to help you stay on track.	There are four key areas to focus on in order to stay well.<br><br>Read about…	Excellent choice. As I'm sure you know, bipolar disorder can be a challenging illness to manage.<br><br>One of the best ways to stay healthy is to learn more about when your symptoms tend to develop and what this looks like for you.<br><br>Learn to recognize…	Ask your psychiatrist or therapist what they think triggers your depression. What have they noticed?<br><br>Ask your psychiatrist or therapist what they think triggers your manias? What have they noticed?<br><br>Consider the input of your psychiatrist or therapist.	What did your psychiatrist or therapist have to say? Any surprises?<br><br>If they identified any triggers of which you were unaware, consider addressing them in your wellness plan (see reduce risk).<br><br>Also consider sharing this information with your supports.	Stay well…

Daily Review Feedback Category 26: Staying Well, Lifestyle – Sleep (Choice 2.1)

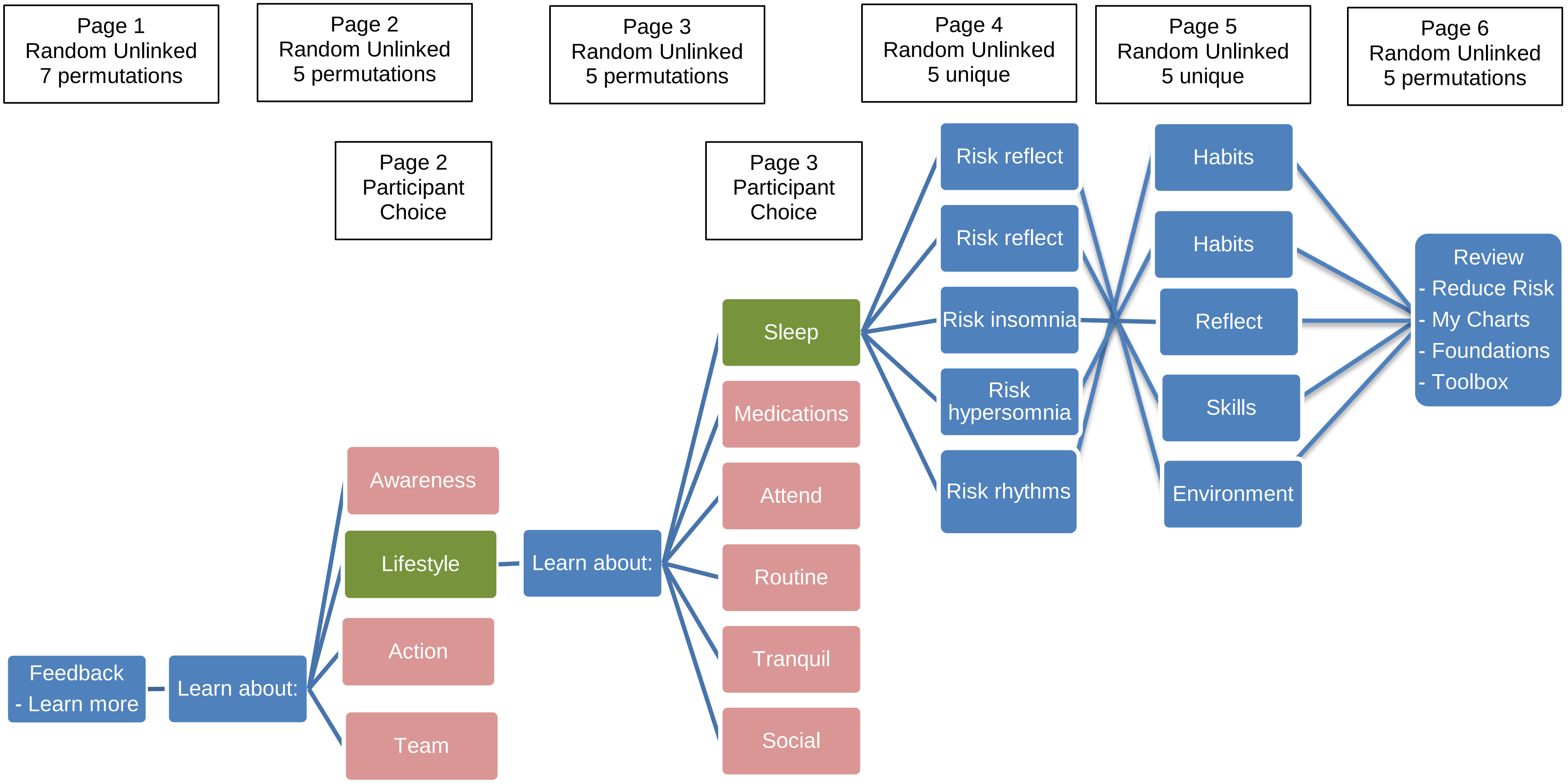

Daily Review Feedback Category 26: Staying Well, Lifestyle – Sleep (Choice 2.1)

Learn more (P1)	Learn about (P1)	Learn about (P6)	Risk Reflect (U16)	Habits (U16)	Reduce Risk (P12)
**1**<br>**RANDOM UNLINKED**	**2**<br>**RANDOM UNLINKED**	**3**<br>**RANDOM UNLINKED**	**4**<br>**RANDOM UNLINKED**	**5**<br>**RANDOM UNLINKED**	**6**<br>**RANDOM UNLINKED**
Glad to see you're doing well. That's great!<br><br>Press continue to learn more about staying well.	Learn more now when you're well. You will be ready to take action whenever things get off for you.<br><br>Read about…	Great choice! Maintaining a healthy lifestyle is crucial to staying well. Without it, managing your life and illness is difficult if not impossible.<br><br>Remember the acronym SMARTS: sleep, medications, attend, routine, tranquil, and social. These are the keys to a healthy lifestyle.<br><br>Learn about…	Have you noticed a relationship between your sleep and symptoms?<br><br>Depression often involves excessive sleep, and mania often involves insufficient sleep.<br><br>Did you know that getting too much or too little sleep can trigger symptoms? Even an hour more or an hour less than needed can cause problems.<br><br>Sleeping 7-9 hours each night is recommended, but anywhere from 6 to 10 hours of sleep per night is considered okay.<br><br>Everyone is different. How much sleep do you need in order to feel rested?	TIP OF THE DAY<br><br>Practice good sleep habits!<br><br>• Turn in the same time every night<br>• Arise the same time every morning<br>• Use your bed for sleep and sex only<br>• Avoid drugs, alcohol, and caffeine<br>• Avoid daytime napping<br><br>If you find it difficult to fall asleep, try some calming imagery or progressive muscle relaxation.<br><br>If you awaken during the night with worries, write them down and then clear your mind.<br><br>Nighttime is for rest, not work!	Double check your sleep plan in Reduce Risk within the Wellness Plan. Do you need to make any changes?<br><br>Stay well…

Daily Review Feedback Category 26: Staying Well, Lifestyle – Sleep (Choice 2.1)

Learn more (P2)	Learn about (P2)	Learn about (P7)	Risk Reflect (U17)	Habits (U17)	Toolbox (P13)

**1**<br>**RANDOM UNLINKED**	**2**<br>**RANDOM UNLINKED**	**3**<br>**RANDOM UNLINKED**	**4**<br>**RANDOM UNLINKED**	**5**<br>**RANDOM UNLINKED**	**6**<br>**RANDOM UNLINKED**
Good to see you're well.<br><br>Continue to learn more about staying well.	Read more about the keys to staying well.<br><br>Check out…	Maintaining a healthy lifestyle is one of the foundations of staying well.<br><br>Learn more…	Sleep is vital to wellbeing. Sleep impacts our attention, concentration, memory, motor skills, emotional health, and physical health.<br><br>Sleep is especially important in managing bipolar disorder. Changes in the amount, as well as in the timing, of sleep can cause mood episodes.<br><br>Sleeping 7-9 hours each night is recommended, but anywhere from 6 to 10 hours of sleep per night is considered ok.<br><br>However, people vary. How much sleep do you need in order to feel rested? How much sleep do you need in order to feel well and function well?	TIP OF THE DAY<br><br>Your bodily processes are very reactive to your surroundings. They respond to time of day, light, and other environmental cues.<br><br>Develop a bedtime routine. This actually should start ONE HOUR before you hope to drift off. Consider:<br><br>• Switch to a low---stimulation activity such as reading or watching television.<br>• Turn down the lights in your home.<br>• Turn off or set aside your computer, laptop, and/or smart phone.<br><br>Allow yourself a smooth transition from activity to sleep.	Check out the Sleep section of Toolbox for more tips on getting better sleep.<br><br>Stay well…

Daily Review Feedback Category 26: Staying Well, Lifestyle – Sleep (Choice 2.1)

Learn more (P3)	Learn about (P3)	Learn about (P8)	Risk (U18)	Reflect (U18)	Foundations (P14)
**1**<br>**RANDOM UNLINKED**	**2**<br>**RANDOM UNLINKED**	**3**<br>**RANDOM UNLINKED**	**4**<br>**RANDOM UNLINKED**	**5**<br>**RANDOM UNLINKED**	**6**<br>**RANDOM UNLINKED**
You say you're doing well. That's good.<br><br>Continue on to learn more about staying well.	Being aware, living a healthy lifestyle, coping with symptoms, and having a good team in place will help you stay well.<br><br>Read more about…	Good choice. Most people find it tough to consistently maintain a healthy lifestyle.<br><br>Don't aim for perfection! Do the best you can, and when you get off track try to get back on track as quickly as possible.<br><br>Learn about…	INSOMNIA<br><br>Insomnia means not getting enough sleep. It can be a symptom of mania.<br><br>Surprisingly perhaps, sleeping too little can also trigger mania!<br><br>The most common causes of insomnia are substances (including caffeine), medical conditions, mood disorders, stress, poor sleep habits, and poor sleep conditions.	TIP OF THE DAY<br><br>Think about the last time you had problems sleeping. What might have been the cause?<br><br>• Mania?<br>• Depression?<br>• Caffeine use?<br>• Alcohol or drug use?<br>• Stress?<br>• Poor sleep habits?<br>• Poor sleep conditions?<br><br>Be mindful of the things that can interfere with your sleep. Watch that you don't put yourself at risk in the future!	Review the module "Lifestyle Skills" in Foundations.<br><br>Stay well…

Daily Review Feedback Category 26: Staying Well, Lifestyle – Sleep (Choice 2.1)

Learn more (P4)	Learn about (P4)	Learn about (P9)	Risk (U19)	Skills (U19)	(P5)

1<br>RANDOM UNLINKED	2<br>RANDOM UNLINKED	3<br>RANDOM UNLINKED	4<br>RANDOM UNLINKED	5<br>RANDOM UNLINKED	6<br>RANDOM UNLINKED
Looks like you're doing well, which is great.<br><br>Continue to learn more about staying well.	Now is a good time to learn more about staying well. There are four areas on which you can focus.<br><br>Read about…	Getting good sleep, taking medications, staying abstinent from drugs and alcohol, having a regular routine, aiming for tranquility, and having a good social life are important aspects of staying well.<br><br>Learn more about…	HYPERSOMNIA<br><br>Have you ever heard of this? It is the opposite of insomnia. Hypersomnia means sleeping TOO MUCH.<br><br>Sleeping too much can be a symptom of depression.<br><br>Surprisingly perhaps, sleeping too much can also worsen depression!<br><br>The most common causes of hypersomnia are depression and stress. (People sleep to avoid stress.)	TIP OF THE DAY<br><br>Sharpen your stress management skills. Coping strategies that ultimately are not helpful include worrying and avoiding.<br><br>There are three effective coping strategies:<br><br>• **Reframe**. This means to think about the situation in a way that brings out the positives for you.<br>• **Accept**. Let go of trying to control things that you cannot control. Accept that the situation "is what it is."<br>• **Problem solve**. If there is a stressful situation that you can make better, do it!<br><br>Most importantly, manage stress during the day! Once nighttime comes, turn it off. It is the time to rest. Set aside your problems!	Stay well…

Daily Review Feedback Category 26: Staying Well, Lifestyle – Sleep (Choice 2.1)

Learn more (P5)	Learn about (P5)	Learn about (P10)	Risk (U20)	Environment (U20)	My Charts (P15)
**1**<br>**RANDOM UNLINKED**	**2**<br>**RANDOM UNLINKED**	**3**<br>**RANDOM UNLINKED**	**4**<br>**RANDOM UNLINKED**	**5**<br>**RANDOM UNLINKED**	**6**<br>**RANDOM UNLINKED**
Great to see you're well.<br><br>Continue for strategies to help you stay on track.	There are four key areas to focus on in order to stay well.<br><br>Read about…	The ability to maintain a healthy lifestyle is related to fewer illness relapses. Think about ways to take care of your body and mind!<br><br>Read about…	Have you heard of circadian rhythms? They are internal processes that cycle roughly every 24 hours. These cycles are observed in plants and animals.<br><br>In humans, circadian rhythms are physical, mental, and behavioral changes that follow a 24-hour cycle. These include changes in consciousness (sleep or wake), hormones, and body temperature<br><br>Disruptions in rhythms are associated with depression, mania, obesity, and diabetes.<br><br>Circadian rhythms respond to internal processes and to the environment.<br><br>Try to maintain routines in your activity and sleep in order to regulate your circadian rhythms.	TIP OF THE DAY<br><br>Do all that you can to make your sleep environment comfortable. Is your mattress comfortable? Is your room quiet? Are you neither too hot nor too cold?<br><br>Consider decorating your bed and bedroom in soothing colors. That includes blues, greens, and purples. Yellows can also be soothing. Avoid reds and oranges. Be sure your room doesn't end up too bright in the mornings when the sun rises.<br><br>Purchase a good mattress. Make sure you have good bed clothes and blankets.<br><br>If it is loud outside move, run a fan, or purchase ear plugs.<br><br>Keep non-sleep objects out of your room, including televisions, computers, and phones.	Check out the relationship between your sleep and wellness in My Charts by clicking on the Wellness Plan below.<br><br>Be well!

Daily Review Feedback Category 26: Staying Well, Lifestyle – Medications (Choice 2.2)

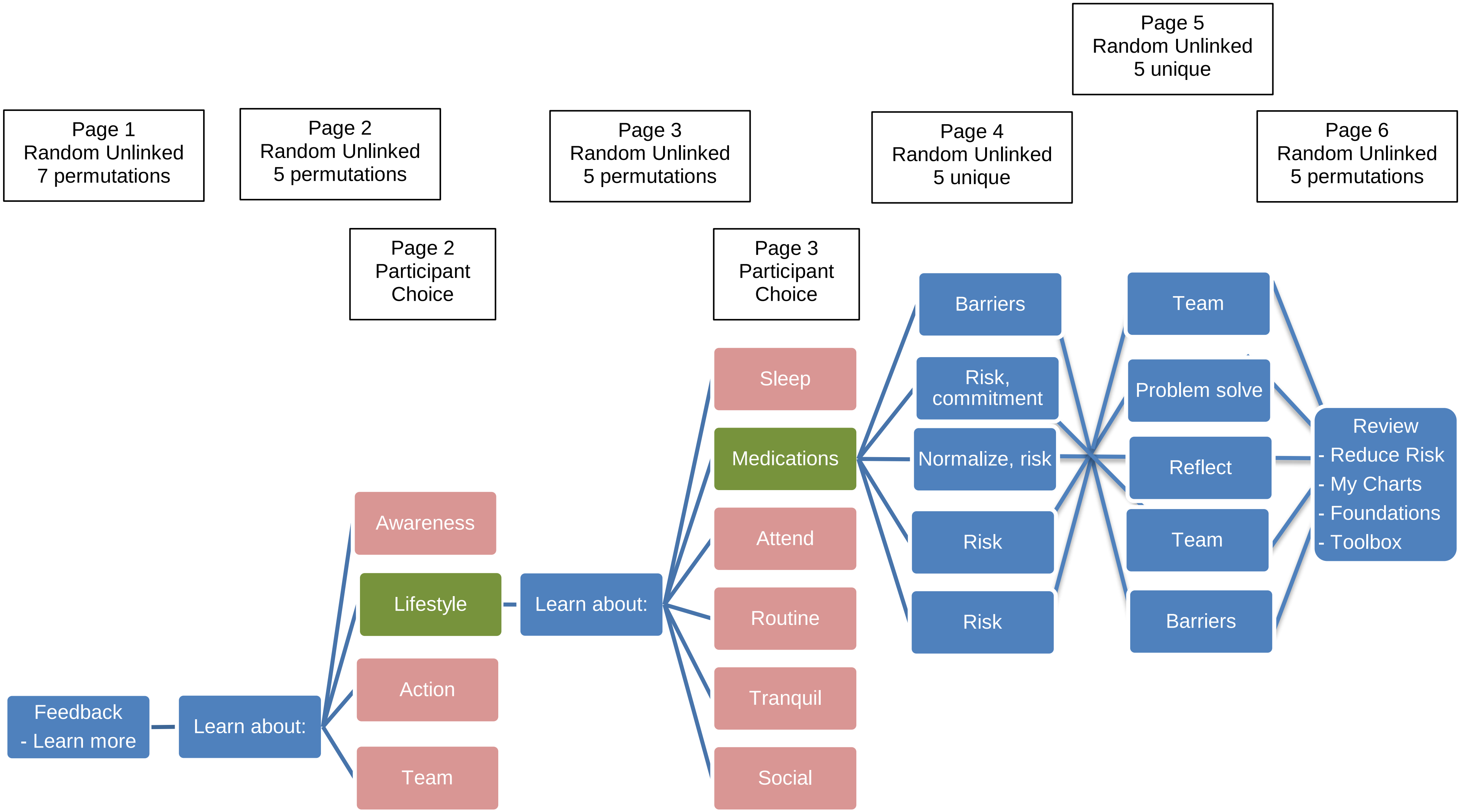

Daily Review Feedback Category 26: Staying Well, Lifestyle – Medications (Choice 2.2)

Learn more (P1)	Learn about (P1)	Learn about (P6)	Barriers (U21)	Team (U21)	Reduce Risk (P16)
**1**<br>**RANDOM UNLINKED**	**2**<br>**RANDOM UNLINKED**	**3**<br>**RANDOM UNLINKED**	**4**<br>**RANDOM UNLINKED**	**5**<br>**RANDOM UNLINKED**	**6**<br>**RANDOM UNLINKED**
Glad to see you're doing well. That's great!<br><br>Press continue to learn more about staying well.	Learn more now when you're well. You will be ready to take action whenever things get off for you.<br><br>Read about…	Great choice! Maintaining a healthy lifestyle is crucial to staying well. Without it, managing your life and illness is difficult if not impossible.<br><br>Remember the acronym SMARTS: sleep, medications, attend, routine, tranquil, and social. These are the keys to a healthy lifestyle.<br><br>Learn about…	Factors related to stopping medicines:<br><br>• I don't think I have an illness<br>• My symptoms are gone<br>• I can manage without medicine<br>• Medicines don't work for me<br>• I don't like the side effects<br>• I'm afraid of side effects<br>• I forget to take my medicine<br>• It's too expensive or inconvenient<br>• Taking medicine is embarrassing<br>• I want to drink or use drugs<br>• I want to feel like myself<br>• I want to feel up<br>• I want to be productive<br>• I don't like my psychiatrist or therapist<br>• My family is against medications<br>• My friends are against medications	TIP OF THE DAY<br><br>Talk to your team about any medication concerns you have. Talk to them about any of the statements on the previous page that you endorsed.<br><br>Open dialogue and careful consideration will help you stay on track.	Double check your medication plan under Reduce Risk in the Wellness Plan. Do you need to make any changes?<br><br>Stay well…

Daily Review Feedback Category 26: Staying Well, Lifestyle – Medications (Choice 2.2)

Learn more (P2)	Learn about (P2)	Learn about (P7)	Risk Commit (U22)	Problem Solve (U22)	Toolbox (P17)
**1**<br>**RANDOM UNLINKED**	**2**<br>**RANDOM UNLINKED**	**3**<br>**RANDOM UNLINKED**	**4**<br>**RANDOM UNLINKED**	**5**<br>**RANDOM UNLINKED**	**6**<br>**RANDOM UNLINKED**
Good to see you're well.<br><br>Continue to learn more about staying well.	Read more about the keys to staying well.<br><br>Check out…	Maintaining a healthy lifestyle is one of the foundations of staying well.<br><br>Learn more…	About Medications<br><br>Medicine is key to wellbeing for individuals with bipolar disorder. It reduces the risk of symptoms and relapses better than anything else that has been studied so far.<br><br>Making a commitment to medicine can be difficult. At times you may think it is unnecessary, you may run into problems with it, you may feel that it seems to conflict with other goals you have, or other people in your life may discourage you about medications.	TIP OF THE DAY<br><br>Solve problems! Do you have any of the following problems? In terms of medications I struggle with…<br><br>• Side effects<br>• Fear of side effects<br>• Forgetfulness<br>• Expense<br>• Inconvenience<br>• Embarrassment<br><br>These are problems that can be solved. Talk to your psychiatrist. Get a pillbox or set an alarm/reminder, budget for your medicine, work on self---acceptance.	Check out the Medication section of Toolbox to learn even more.<br><br>Stay well…

Daily Review Feedback Category 26: Staying Well, Lifestyle – Medications (Choice 2.2)

Learn more (P3)	Learn about (P3)	Learn about (P8)	Normalize Risk (U23)	Reflect (U23)	Foundations (P14)
**1**<br>**RANDOM UNLINKED**	**2**<br>**RANDOM UNLINKED**	**3**<br>**RANDOM UNLINKED**	**4**<br>**RANDOM UNLINKED**	**5**<br>**RANDOM UNLINKED**	**6**<br>**RANDOM UNLINKED**
You say you're doing well. That's good.<br><br>Continue on to learn more about staying well.	Being aware, living a healthy lifestyle, coping with symptoms, and having a good team in place will help you stay well.<br><br>Read more about…	Good choice. Most people find it tough to consistently maintain a healthy lifestyle.<br><br>Don't aim for perfection! Do the best you can, and when you get off track try to get back on track as quickly as possible.<br><br>Learn about…	About Medications<br><br>Following medical advice is hard. Only about 50% of people take medicine as prescribed. This is true for all sorts of conditions including diabetes and hypertension. It is also true for bipolar disorder.<br><br>Not taking medications as prescribed, however, can result in relapses of mania and depression, hospitalization, and longer hospital stays.<br><br>Remember, it's your health!	TIP OF THE DAY<br><br>Educate yourself! Do you hold any of the following beliefs? I do not need medications because…<br><br>• I don't have an illness<br>• My symptoms are gone<br>• I can manage on my own<br>• Medications don't work for me<br><br>Read! Make sure you understand the nature of bipolar disorder.<br><br>Reflect! Make sure you understand your bipolar disorder. People often think they are fine when they are manic. Trust your family and doctor to be honest with you.	Review the module "Lifestyle Skills" in Foundations.<br><br>Stay well…

Daily Review Feedback Category 26: Staying Well, Lifestyle – Medications (Choice 2.2)

Learn more (P4)	Learn about (P4)	Learn about (P9)	Risk (U24)	Team (U24)	(P5)

1<br>RANDOM UNLINKED	2<br>RANDOM UNLINKED	3<br>RANDOM UNLINKED	4<br>RANDOM UNLINKED	5<br>RANDOM UNLINKED	6<br>RANDOM UNLINKED
Looks like you're doing well, which is great.<br><br>Continue to learn more about staying well.	Now is a good time to learn more about staying well. There are four areas on which you can focus.<br><br>Read about…	Getting good sleep, taking medications, staying abstinent from drugs and alcohol, having a regular routine, aiming for tranquility, and having a good social life are important aspects of staying well.<br><br>Learn more about…	Why Medications?<br><br>Research shows that medication is critical for people with bipolar disorder to live well.<br><br>ACUTE PHASE<br>Symptoms can last for months without medicine. Symptoms harm relationships and the ability to work. With medicine, mood episodes may be resolved in weeks.<br><br>REMISSION PHASE<br>Not taking medicine is associated with more frequent relapses, hospitalizations, and suicidal thoughts. Medicine cuts the chances of future mood episodes by half.	TIP OF THE DAY<br><br>Get support! Do you experience any of the following challenges? I don't want to take medications because…<br><br>• Problems with my psychiatrist<br>• Problems with my therapist<br>• My family is against medications<br>• My friends are against medications<br><br>Try to work things out with your current providers or find new ones. Get your family and friends informed or reach out to others who will support you.	Stay well…

Daily Review Feedback Category 26: Staying Well, Lifestyle – Medications (Choice 2.2)

Learn more (P5)	Learn about (P5)	Learn about (P10)	Risk (U25)	Barriers (U25)	My Charts (P18)
**1**<br>**RANDOM UNLINKED**	**2**<br>**RANDOM UNLINKED**	**3**<br>**RANDOM UNLINKED**	**4**<br>**RANDOM UNLINKED**	**5**<br>**RANDOM UNLINKED**	**6**<br>**RANDOM UNLINKED**
Great to see you're well.<br><br>Continue for strategies to help you stay on track.	There are four key areas to focus on in order to stay well.<br><br>Read about…	The ability to maintain a healthy lifestyle is related to fewer illness relapses. Think about ways to take care of your body and mind!<br><br>Read about…	Why Medications?<br><br>Medication is an essential part of the treatment for bipolar disorder. It is the foundation for the treatment of bipolar disorder.<br><br>Be sure to take your medicine as prescribed each and every day. If you are having any problems with your medicine, then consult your doctor.	TIP OF THE DAY<br><br>Resolve conflicts! Do you have any of the following conflicts? I don't want to take medications because…<br><br>• I want to drink or use drugs<br>• I want to feel like myself<br>• I want to feel up<br>• I want to be productive<br><br>Talk to your psychiatrist about all of these things. You need to be safe. You need to feel like yourself. A combination of the right medications and skills will get you there.	Check out the relationship between your medication and wellness in My Charts by clicking on the Wellness Plan below.<br><br>Be well!

Daily Review Feedback Category 26: Staying Well, Lifestyle – Attend (Choice 2.3)

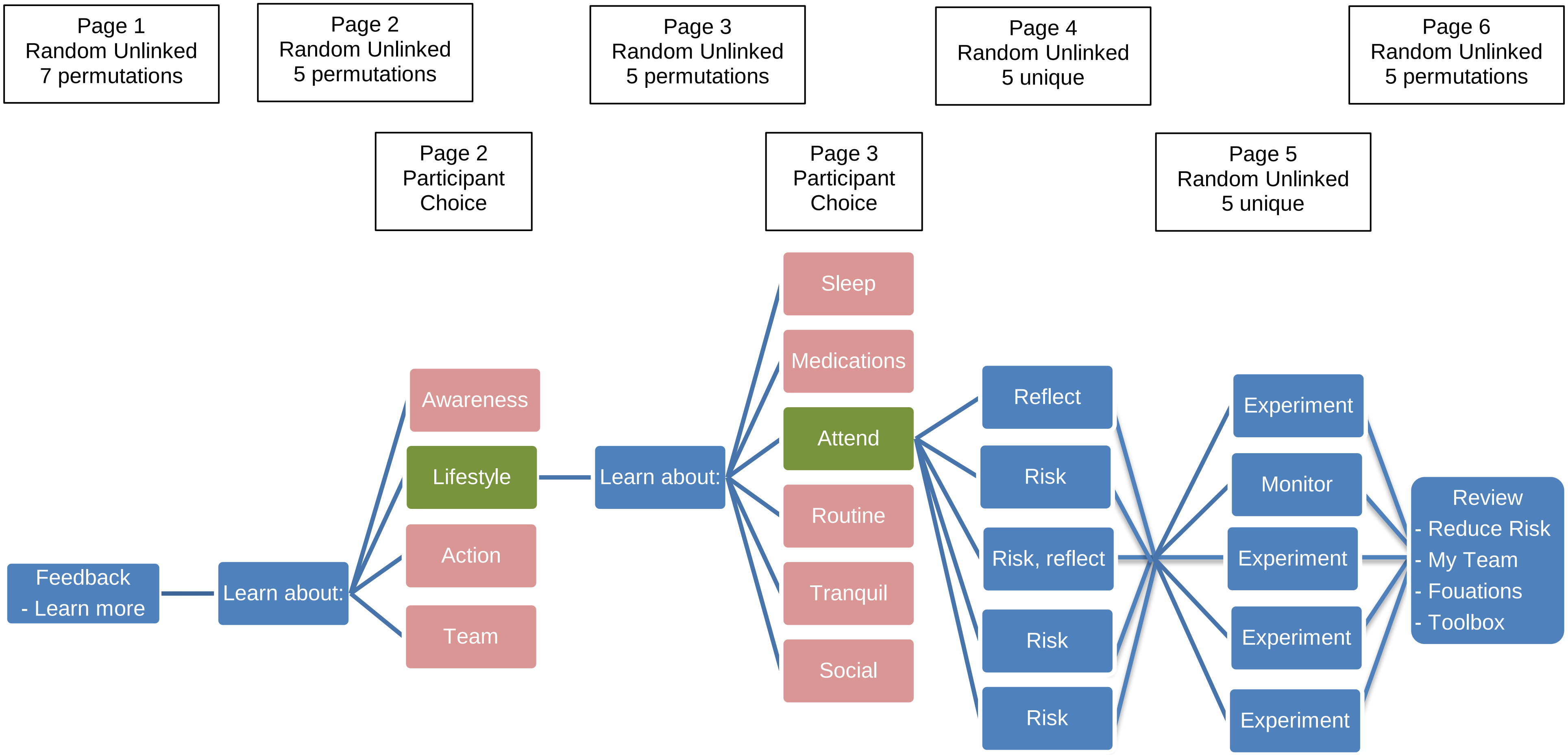

Daily Review Feedback Category 26: Staying Well, Lifestyle – Attend (Choice 2.3)

Learn more (P1)	Learn about (P1)	Learn about (P6)	Reflect (U26)	Experiment (U26)	Reduce Risk (P19)
**1**<br>**RANDOM UNLINKED**	**2**<br>**RANDOM UNLINKED**	**3**<br>**RANDOM UNLINKED**	**4**<br>**RANDOM UNLINKED**	**5**<br>**RANDOM UNLINKED**	**6**<br>**RANDOM UNLINKED**
Glad to see you're doing well. That's great!<br><br>Press continue to learn more about staying well.	Learn more now when you're well. You will be ready to take action whenever things get off for you.<br><br>Read about...	Great choice! Maintaining a healthy lifestyle is crucial to staying well. Without it, managing your life and illness is difficult if not impossible.<br><br>Remember the acronym SMARTS: sleep, medications, attend, routine, tranquil, and social. These are the keys to a healthy lifestyle.<br><br>Learn about...	Quiz Yourself<br><br>Consider the following:<br><br>• Have you tried to cut down your substance use?<br>• Do you get annoyed when others talk to you about your use?<br>• Do you feel guilty from time to time about your use?<br>• Have you been drinking as an "eye opener" of late?<br><br>If you answered "yes" to any of these questions you may want to take a closer look at your substance use.	TIP OF THE DAY<br><br>Try cutting back on the "psychoactive" substances you use. Psychoactive means any substance that impacts your state of mind. This includes:<br><br>• Nicotine<br>• Caffeine<br>• Alcohol<br>• Illegal drugs<br>• Un-prescribed legal drugs<br><br>Try it for 3 days. See if you feel better or worse. (If you are using large amounts of substances, consult your psychiatrist before cutting back. It can be dangerous.)	Go to the Wellness Plan's Reduce Risk section to review your plan for attending to a healthy lifestyle.<br><br>Do you need to make changes?<br><br>Stay well...

Daily Review Feedback Category 26: Staying Well, Lifestyle – Attend (Choice 2.3)

Learn more (P2)	Learn about (P2)	Learn about (P7)	Risk (U27)	Monitor (U27)	Toolbox (P20)

1<br>**RANDOM UNLINKED**	2<br>**RANDOM UNLINKED**	3<br>**RANDOM UNLINKED**	4<br>**RANDOM UNLINKED**	5<br>**RANDOM UNLINKED**	6<br>**RANDOM UNLINKED**
Good to see you're well.<br><br>Continue to learn more about staying well.	Read more about the keys to staying well.<br><br>Check out…	Maintaining a healthy lifestyle is one of the foundations of staying well.<br><br>Learn more…	About Attend<br><br>Substance misuse causes mental, physical, and social problems.<br><br>Problem chemicals include nicotine, caffeine, alcohol, illegal drugs, and use of prescription medications in ways not recommended by your physician.<br><br>Substance misuse worsens bipolar disorder. It triggers symptoms. It reduces the effectiveness of medicines. It interferes with taking medicines as prescribed. For your health, consider removing these things from your "diet".<br><br>Nourish your body and mind!	TIP OF THE DAY<br><br>Write down everything you consumed over the past 3 days. Include types of food, water, caffeine, alcohol, and drugs.<br><br>Now for the next three days try eating only vegetables, fruits, and protein. Drink 8 cups of water a day. Cut out caffeine, alcohol, and drugs.<br><br>See if you feel any different…	Go to Toolbox below to check out the Attend section of Lifestyle to learn even more.<br><br>Stay well…

Daily Review Feedback Category 26: Staying Well, Lifestyle – Attend (Choice 2.3)

Learn more (P3)	Learn about (P3)	Learn about (P8)	Risk Reflect (U28)	Experiment (U28)	Foundations (P14)
**1**<br>**RANDOM UNLINKED**	**2**<br>**RANDOM UNLINKED**	**3**<br>**RANDOM UNLINKED**	**4**<br>**RANDOM UNLINKED**	**5**<br>**RANDOM UNLINKED**	**6**<br>**RANDOM UNLINKED**
You say you're doing well. That's good.<br><br>Continue on to learn more about staying well.	Being aware, living a healthy lifestyle, coping with symptoms, and having a good team in place will help you stay well.<br><br>Read more about…	Good choice. Most people find it tough to consistently maintain a healthy lifestyle.<br><br>Don't aim for perfection! Do the best you can, and when you get off track try to get back on track as quickly as possible.<br><br>Learn about…	Did you know?<br><br>Roughly 25% to 50% of individuals with bipolar disorder abuse alcohol and/or drugs.<br><br>Undoubtedly this is often an attempt to self-medicate as the symptoms are so difficult to tolerate.<br><br>Of course, in the end alcohol and drugs only makes things worse. What has been your experience with alcohol and drugs? How much do you use now?	TIP OF THE DAY<br><br>Use of caffeine, alcohol, and drugs is often driven by attempts to wake up or relax.<br><br>Try going "natural" for three days. Avoid caffeine, alcohol, and drugs. Instead, eat healthy, drink lots of water, sleep well, and exercise 30 minutes a day (even just walking).<br><br>See what your energy level is like…	Review the module "Lifestyle Skills" in Foundations.<br><br>Stay well…

Daily Review Feedback Category 26: Staying Well, Lifestyle – Attend (Choice 2.3)

Learn more (P4)	Learn about (P4)	Learn about (P9)	Risk (U29)	Experiment (U29)	(P5)

**1**<br>**RANDOM UNLINKED**	**2**<br>**RANDOM UNLINKED**	**3**<br>**RANDOM UNLINKED**	**4**<br>**RANDOM UNLINKED**	**5**<br>**RANDOM UNLINKED**	**6**<br>**RANDOM UNLINKED**
Looks like you're doing well, which is great.<br><br>Continue to learn more about staying well.	Now is a good time to learn more about staying well. There are four areas on which you can focus.<br><br>Read about…	Getting good sleep, taking medications, staying abstinent from drugs and alcohol, having a regular routine, aiming for tranquility, and having a good social life are important aspects of staying well.<br><br>Learn more about…	About Attend<br><br>Psychoactive substances are things individuals ingest that impact their mental state. This includes things such as nicotine, caffeine, alcohol, and illegal drugs. Misuse causes mental, physical, and social problems.<br><br>Misuse also worsens bipolar disorder. It triggers symptoms. It interferes with medicines. It gets in the way of taking medications regularly.<br><br>For your health, consider eliminating these things from your "diet". Nourish your body and mind! Consider treatment if you cannot stop yourself.	TIP OF THE DAY<br><br>Do something for your physical health today. Pick from the following menu:<br><br>• Consume only fruits, vegetables, protein, and water (i.e., no junk food, caffeine, alcohol, and drugs)<br>• Prepare meals made only from fresh ingredients (no boxed or packaged foods)<br>• Take a brisk walk for 20 minutes outside (exercising outside has more mental health benefits than exercising indoors)<br>• Do yoga for 30 minutes (go to a class or follow a video online)<br><br>See how you feel at the end of the day…	Stay well…

Daily Review Feedback Category 26: Staying Well, Lifestyle – Attend (Choice 2.3)

Learn more (P5)	Learn about (P5)	Learn about (P10)	Risk (U30)	Experiment (U30)	My Team (P21)
**1**<br>**RANDOM UNLINKED**	**2**<br>**RANDOM UNLINKED**	**3**<br>**RANDOM UNLINKED**	**4**<br>**RANDOM UNLINKED**	**5**<br>**RANDOM UNLINKED**	**6**<br>**RANDOM UNLINKED**
Great to see you're well.<br><br>Continue for strategies to help you stay on track.	There are four key areas to focus on in order to stay well.<br><br>Read about…	The ability to maintain a healthy lifestyle is related to fewer illness relapses. Think about ways to take care of your body and mind!<br><br>Read about…	About Attend<br><br>Smoking, drinking coffee, and drinking alcohol are part of our culture. They are often woven into the fabric of our daily lives.<br><br>The widespread use of mind altering substances hides their danger. Even small amounts of these substances alter your chemistry. They change how you think, feel, and act. They alter the smooth functioning of your circadian rhythms. They can trigger symptoms.	TIP OF THE DAY<br><br>Do you use caffeine, alcohol, or drugs? If so, think about what you get out of it. Now think about other ways you can get the same thing! Consider altering your behavior.<br><br>For example, if coffee helps you wake up in the morning, consider a brisk walk instead, or maybe even just 10 minutes of stretching. If alcohol helps you wind down at night, consider a walk instead, or maybe even just 10 minutes of mindful meditation.	Did you learn something new? Would it be good to discuss with your psychiatrist and maybe your supports?<br><br>Stay well…

Daily Review Feedback Category 26: Staying Well, Lifestyle – Routine (Choice 2.4)

Page 5
Random Unlinked
5 unique

Page 1
Random Unlinked
7 permutations

Page 2
Random Unlinked
5 permutations

Page 3
Random Unlinked
5 permutations

Page 4
Random Unlinked
5 unique

Page 6
Random Unlinked
5 permutations

Page 2
Participant
Choice

Page 3
Participant
Choice

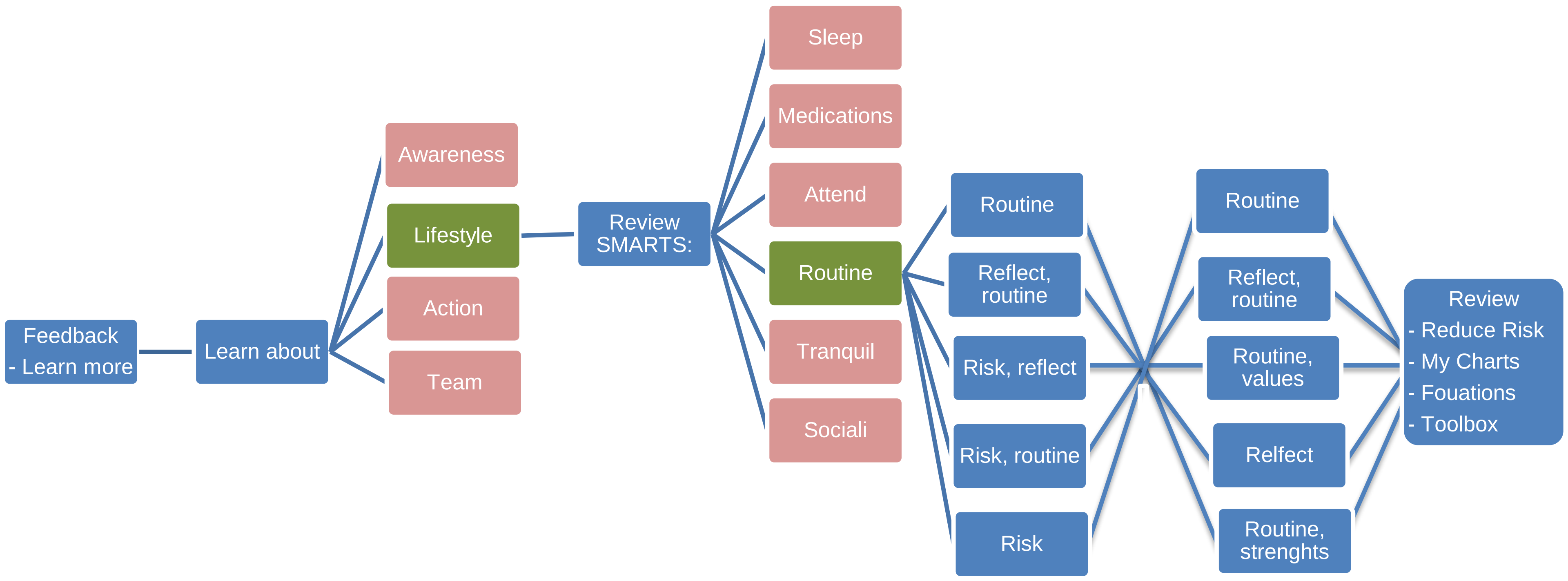

Daily Review Feedback Category 26: Staying Well, Lifestyle – Routine (Choice 2.4)

Learn more (P1)	Learn about (P1)	Learn about (P6)	Routine (U31)	Routine (U31)	Reduce Risk (P22)
**1**<br>**RANDOM UNLINKED**	**2**<br>**RANDOM UNLINKED**	**3**<br>**RANDOM UNLINKED**	**4**<br>**RANDOM UNLINKED**	**5**<br>**RANDOM UNLINKED**	**6**<br>**RANDOM UNLINKED**
Glad to see you're doing well. That's great!<br><br>Press continue to learn more about staying well.	Learn more now when you're well. You will be ready to take action whenever things get off for you.<br><br>Read about…	Great choice! Maintaining a healthy lifestyle is crucial to staying well. Without it, managing your life and illness is difficult if not impossible.<br><br>Remember the acronym SMARTS: sleep, medications, attend, routine, tranquil, and social. These are the keys to a healthy lifestyle.<br><br>Learn about…	About Routine<br><br>Doesn't moderation always seem to be the answer to everything?<br><br>Try striking a balance between living with structure and living with spontaneity.<br><br>Why?<br><br>Because having regularity in your routine is good for your body. It helps set your internal "clock". This helps your body function properly in many ways.<br><br>Because having some regularity in your routine is good for your mind. It cuts down on the number of decisions you have to make every day. This reduces mental strain and keeps you sharper.	TIP OF THE DAY<br><br>Do the following about the same time every day (give or take 30 minutes).<br><br>• Getting up in the morning<br>• Seeing others for the first time<br>• Starting your work day<br>• Eating dinner<br>• Turning in for the night<br><br>Even if you don't think this will make a difference, try it for a week or two. See what happens to your sense of wellness.	Double check your routine plan in Reduce Risk in the Wellness Plan below. Do you need to make any changes?<br><br>Stay well…

Daily Review Feedback Category 26: Staying Well, Lifestyle – Routine (Choice 2.4)

Learn more (P2)	Learn about (P2)	Learn about (P7)	Reflect Routine (U32)	Reflect Routine (U32)	Toolbox (P23)

1<br>RANDOM UNLINKED	2<br>RANDOM UNLINKED	3<br>RANDOM UNLINKED	4<br>RANDOM UNLINKED	5<br>RANDOM UNLINKED	6<br>RANDOM UNLINKED
Good to see you're well.<br><br>Continue to learn more about staying well.	Read more about the keys to staying well.<br><br>Check out…	Maintaining a healthy lifestyle is one of the foundations of staying well.<br><br>Learn more…	About Routine<br><br>Routine. Regularity. Rhythm. This is important for health. It is vital to managing bipolar disorder.<br><br>What does your schedule look like? Make a plan. Keep a routine for sleep. Keep a routine for activities.<br><br>In particular try to keep these things about the same every day. Time you:<br><br>• Get out of bed<br>• First have contact with another person<br>• Start work/school/volunteer/ family care<br>• Dinner<br>• Get to bed Try it.<br><br>See how you feel.	TIP OF THE DAY<br><br>Healthy routines build esteem and joy.<br><br>Make plans for a sense of MASTERY. Make plans for a sense of PLEASURE.<br><br>Activities include sleeping, eating, exercising, grooming, chores, childcare, socializing, working, school, and leisure.<br><br>What is your schedule? MAP it out!<br><br>• Wake-up time and routine<br>• Morning activities<br>• Afternoon activities<br>• Evening activities<br>• Bedtime time and routine<br><br>See how you feel after trying this for a couple of days.	Check out the Routine section of Toolbox to learn even more.<br><br>Stay well…

Daily Review Feedback Category 26: Staying Well, Lifestyle – Routine (Choice 2.4)

Learn more (P3)	Learn about (P3)	Learn about (P8)	Risk Reflect (U33)	Routine Values (U33)	Foundations (P14)
**1**<br>**RANDOM UNLINKED**	**2**<br>**RANDOM UNLINKED**	**3**<br>**RANDOM UNLINKED**	**4**<br>**RANDOM UNLINKED**	**5**<br>**RANDOM UNLINKED**	**6**<br>**RANDOM UNLINKED**
You say you're doing well. That's good.<br><br>Continue on to learn more about staying well.	Being aware, living a healthy lifestyle, coping with symptoms, and having a good team in place will help you stay well.<br><br>Read more about…	Good choice. Most people find it tough to consistently maintain a healthy lifestyle.<br><br>Don't aim for perfection! Do the best you can, and when you get off track try to get back on track as quickly as possible.<br><br>Learn about…	About Routine<br><br>Having a regular routine is important for physical and mental health. When you're active and when you're asleep are the main points of a routine.<br><br>It is especially important for individuals with bipolar disorder. It seems that getting out of sync can trigger symptoms.<br><br>What term do you like to describe routine?<br><br>• Routine<br>• Rhythm<br>• Regularity<br>• Structure<br>• Anchors	TIP OF THE DAY<br><br>Healthy routines build on core values. What is your schedule?<br><br>Honor your values!<br>• Family relations<br>• Marriage, couples, intimate relations<br>• Parenting<br>• Friendships/social relations<br>• Employment<br>• Education/training<br>• Recreation<br>• Spirituality<br>• Citizenship/community life<br>• Physical well---being<br><br>Make sure your day to day activities include those things you value most! Try it for a few days. See how it feels.	Review the module "Lifestyle Skills" in Foundations.<br><br>Stay well…

Daily Review Feedback Category 26: Staying Well, Lifestyle – Routine (Choice 2.4)

Learn more (P4)	Learn about (P4)	Learn about (P9)	Risk Routine (U34)	Reflect (U34)	Reduce Risk (P24)
**1**<br>**RANDOM UNLINKED**	**2**<br>**RANDOM UNLINKED**	**3**<br>**RANDOM UNLINKED**	**4**<br>**RANDOM UNLINKED**	**5**<br>**RANDOM UNLINKED**	**6**<br>**RANDOM UNLINKED**
Looks like you're doing well, which is great.<br><br>Continue to learn more about staying well.	Now is a good time to learn more about staying well. There are four areas on which you can focus.<br><br>Read about…	Getting good sleep, taking medications, staying abstinent from drugs and alcohol, having a regular routine, aiming for tranquility, and having a good social life are important aspects of staying well.<br><br>Learn more about…	About Routine<br><br>Depressive and manic symptoms tend to disrupt daily routines. It is also true that disruptions in daily routines can trigger symptoms.<br><br>No matter what, try to keep a regular schedule in life. Get up around the same time each day, and turn in around the same time each day. Have a morning and evening routine.	TIP OF THE DAY<br><br>Sometimes episodes of mania or depression derail your plans and goals. Healthy routines support recovery.<br><br>There are six keys to recovery from problems due to bipolar disorder.<br><br>• Environment<br>• Treatment<br>• Support<br>• Hope<br>• Skills<br>• Strengths<br><br>Think about your own life. What do you want?<br><br>Devote time to these aspects of your life for a few days. See how it feels.	Read more healthy lifestyles:<br><br>www.cdc.gov<br><br>Stay well…

Daily Review Feedback Category 26: Staying Well, Lifestyle – Routine (Choice 2.4)

Learn more (P5)	Learn about (P5)	Learn about (P10)	Risk (U35)	Routine Strengths (U35)	My Charts (P25)
**1**<br>**RANDOM UNLINKED**	**2**<br>**RANDOM UNLINKED**	**3**<br>**RANDOM UNLINKED**	**4**<br>**RANDOM UNLINKED**	**5**<br>**RANDOM UNLINKED**	**6**<br>**RANDOM UNLINKED**
Great to see you're well.<br><br>Continue for strategies to help you stay on track.	There are four key areas to focus on in order to stay well.<br><br>Read about…	The ability to maintain a healthy lifestyle is related to fewer illness relapses. Think about ways to take care of your body and mind!<br><br>Read about…	About Routine<br><br>Did you know that there are more car accidents than average when the time changes ahead in the spring? People are sleep deprived and have trouble concentrating!<br><br>In fact, as much as a 30 minute shift in your schedule will impact your mind, emotions, and body. Stick to a routine for good health!	TIP OF THE DAY<br>Strengths are inherent talents. You can use your strengths to increase your wellness. Participating in activities that rely on your strengths will increase your sense of wellbeing. Be sure your routine includes activities that rely on your strengths.<br><br>□ Love of learning<br>□ Social intelligence<br>□ Creativity<br>□ Curiosity<br>□ Judgment<br>□ Perspective<br>□ Bravery<br>□ Persistence<br>□ Authenticity<br>□ Zest<br>□ Love<br>□ Kindness<br>□ Fairness<br>□ Leadership<br>□ Citizenship<br>□ Teamwork<br>□ Forgiveness<br>□ Humility<br>□ Prudence<br>□ Self---control<br>□ Appreciation of beauty<br>□ Gratitude<br>□ Hope<br>□ Humor<br>□ Spirituality<br><br>Engage in activities that emphasize your strengths. See how it feels at the end of the day.	Check out the relationship between your routine and wellness in My Charts by clicking on the Wellness Plan below.<br><br>Stay well.

Daily Review Feedback Category 26: Staying Well, Lifestyle – Tranquil (Choice 2.5)

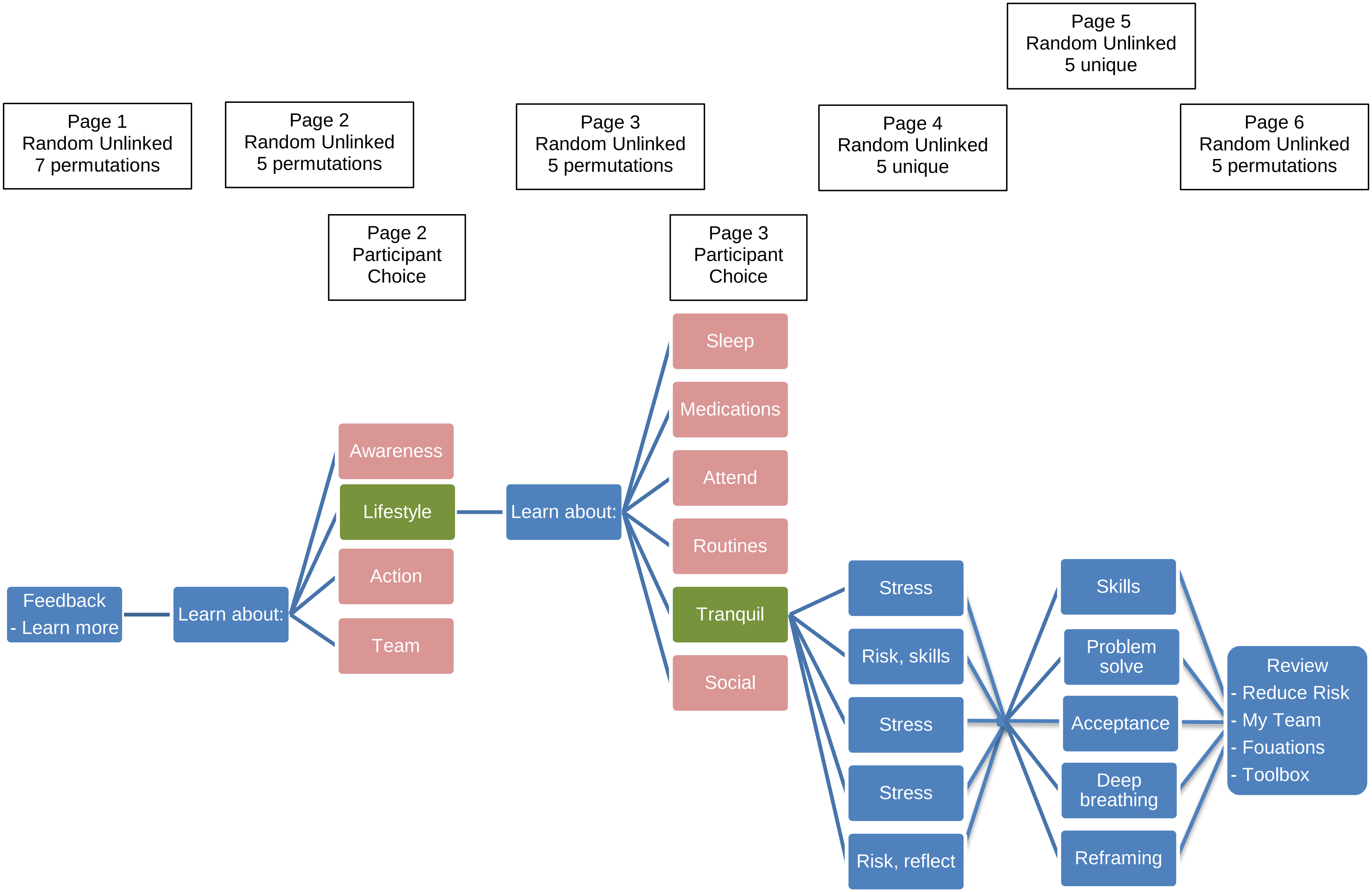

Daily Review Feedback Category 26: Staying Well, Lifestyle – Tranquil (Choice 2.5)

Learn more (P1)	Learn about (P1)	Lifestyle (P6)	Stress (U36)	Skills (U36)	Reduce Risk (P26)
**1**<br>**RANDOM UNLINKED**	**2**<br>**RANDOM UNLINKED**	**3**<br>**RANDOM UNLINKED**	**4**<br>**RANDOM UNLINKED**	**5**<br>**RANDOM UNLINKED**	**6**<br>**RANDOM UNLINKED**
Glad to see you're doing well. That's great!<br><br>Press continue to learn more about staying well.	Learn more now when you're well. You will be ready to take action whenever things get off for you.<br><br>Read about…	Great choice! Maintaining a healthy lifestyle is crucial to staying well. Without it, managing your life and illness is difficult if not impossible.<br><br>Remember the acronym SMARTS: sleep, medications, attend, routine, tranquil, and social. These are the keys to a healthy lifestyle.<br><br>Learn about…	About Stress<br><br>Stress is a part of life.<br><br>Daily hassles are the day-to- day events that occur and cause tension. Daily hassles include things like getting stuck in traffic or misplacing your keys.<br><br>Live events are the more occasional, major disruptions in life. Live events include losing a job, getting a divorce, and moving.<br><br>The key to wellness is to cope as best as possible with daily hassles and life events.	TIP OF THE DAY<br><br>DO:<br><br>• Maintain a positive attitude<br>• Solve the problems you can<br>• Accept problems you cannot change<br>• Use deep breathing to calm down<br><br>DON'T:<br><br>• Avoid problems that can be solved (it makes things worse)<br>• Worry (because it does not help)<br><br>Try out these strategies. See if you can cope better with practice.	Go to the Wellness Plan to double check your stress plan under tranquility in Reduce Risk. Do you need to make any changes?<br><br>Stay well…

Daily Review Feedback Category 26: Staying Well, Lifestyle – Tranquil (Choice 2.5)

Learn more (P2)	Learn about (P2)	Lifestyle (P7)	Risk Skills (U37)	Problem Solve (U37)	Toolbox (P27)
**1**<br>**RANDOM UNLINKED**	**2**<br>**RANDOM UNLINKED**	**3**<br>**RANDOM UNLINKED**	**4**<br>**RANDOM UNLINKED**	**5**<br>**RANDOM UNLINKED**	**6**<br>**RANDOM UNLINKED**
Good to see you're well.<br><br>Continue to learn more about staying well.	Read more about the keys to staying well.<br><br>Check out…	Maintaining a healthy lifestyle is one of the foundations of staying well.<br><br>Learn more…	About Stress<br><br>Stress is part of life. However, intense or prolonged stress can cause many physical and mental health problems. Stress can trigger symptoms.<br><br>Problem---focused coping means coping with problems. It means approaching them and solving them if you can. Solvable problems should not be avoided.<br><br>Emotion---focused coping means regulating your feelings. It means allowing them and being gentle. Always tend to your feelings. Console yourself.	TIP OF THE DAY<br><br>Problem solving…<br><br>Avoiding problems causes more problems. For things that can be changed…act!<br><br>1. Identify the problem. Describe it in detail.<br>2. Select your goal. Describe what you would like to see happen.<br>3. Generate alternative solutions. Come up with several plans.<br>4. Evaluate the alternatives. Which are practical? Which will work best?<br>5. Implement your plan. Decide on a time and place. Do it!<br>6. Evaluate the results. Did it work? If not, should you do something else?<br><br>Practice problem solving today! See what you think.	Check out the Tranquility section of Toolbox to learn even more.<br><br>Stay well…

Daily Review Feedback Category 26: Staying Well, Lifestyle – Tranquil (Choice 2.5)

Learn more (P3)	Learn about (P3)	Lifestyle (P8)	Stress (U38)	Acceptance (U38)	Foundations (P14)
**1**<br>**RANDOM UNLINKED**	**2**<br>**RANDOM UNLINKED**	**3**<br>**RANDOM UNLINKED**	**4**<br>**RANDOM UNLINKED**	**5**<br>**RANDOM UNLINKED**	**6**<br>**RANDOM UNLINKED**
You say you're doing well. That's good.<br><br>Continue on to learn more about staying well.	Being aware, living a healthy lifestyle, coping with symptoms, and having a good team in place will help you stay well.<br><br>Read more about…	Good choice. Most people find it tough to consistently maintain a healthy lifestyle.<br><br>Don't aim for perfection! Do the best you can, and when you get off track try to get back on track as quickly as possible.<br><br>Learn about…	About Stress<br><br>As you probably already know, stress is related to physical and mental health problems. Intense, frequent, and prolonged stress is especially problematic.<br><br>Take charge of how you cope with stress. While stress is unavoidable, you certainly can control how you respond to stress. Practice positive coping strategies!	TIP OF THE DAY<br><br>Radical acceptance…<br><br>Fighting reality causes suffering. For things that cannot be changed…accept.<br><br>• Acknowledge what is in yourself<br>• Acknowledge what is in others<br>• Acknowledge what is in life<br><br>Accepting something is not the same as liking something. Accepting something is not the same as judging something.<br><br>Remember that nothing and no one is perfect. Acknowledge the good and bad.<br><br>Practice acceptance today. See what you think.	Review the module "Lifestyle Skills" in Foundations.<br><br>Stay well…

Daily Review Feedback Category 26: Staying Well, Lifestyle – Tranquil (Choice 2.5)

Learn more (P4)	Learn about (P4)	Lifestyle (P9)	Stress (U39)	Deep Breathing (U39)	Reduce Risk (P24)
**1**<br>**RANDOM UNLINKED**	**2**<br>**RANDOM UNLINKED**	**3**<br>**RANDOM UNLINKED**	**4**<br>**RANDOM UNLINKED**	**5**<br>**RANDOM UNLINKED**	**6**<br>**RANDOM UNLINKED**
Looks like you're doing well, which is great.<br><br>Continue to learn more about staying well.	Now is a good time to learn more about staying well. There are four areas on which you can focus.<br><br>Read about…	Getting good sleep, taking medications, staying abstinent from drugs and alcohol, having a regular routine, aiming for tranquility, and having a good social life are important aspects of staying well.<br><br>Learn more about…	About Stress<br><br>Stress is a part of life. Stress can also trigger symptoms of depression and mania. So it is important that you actively cope with stress each and every day.<br><br>Aim for tranquility in your life. No matter what is going on, aim to cope. Solve the problems you can and accept the problems you cannot solve. Don't fight life. Be at peace!	TIP OF THE DAY<br><br>Deep breathing…<br><br>Your diaphragm is the muscle that controls breathing. It is located just below your lungs. Using your diaphragm to its fullest is calming.<br><br>Breathe by using your belly rather than your chest. This is the best way.<br><br>TAKE SLOW, DEEP, BELLY BREATHS<br><br>Variations:<br><br>• Count to three as you breathe in and count to three as you breathe out.<br><br>• Think as you breathe in and let go as you breathe out.<br><br>Practice deep breathing today. See what you think.	Read more healthy lifestyles:<br><br>www.cdc.gov<br><br>Stay well…

Daily Review Feedback Category 26: Staying Well, Lifestyle – Tranquil (Choice 2.5)

Learn more (P5)	Learn about (P5)	Lifestyle (P10)	Risk Reflect (U40)	Reframe (U40)	My Team (P21)
**1**<br>**RANDOM UNLINKED**	**2**<br>**RANDOM UNLINKED**	**3**<br>**RANDOM UNLINKED**	**4**<br>**RANDOM UNLINKED**	**5**<br>**RANDOM UNLINKED**	**6**<br>**RANDOM UNLINKED**
Great to see you're well.<br><br>Continue for strategies to help you stay on track.	There are four key areas to focus on in order to stay well.<br><br>Read about…	The ability to maintain a healthy lifestyle is related to fewer illness relapses. Think about ways to take care of your body and mind!<br><br>Read about…	About Stress<br><br>Stress is part of life. It is normal. However, intense or prolonged stress is not normal. It causes many physical health problems. It causes many mental health problems. Stress can trigger symptoms.<br><br>Live a grounded life. Eliminate unnecessary stress. Look at your financial, housing, social, and work situations. Are they good enough? Do you need to change anything?<br><br>Live an empowered life. Consider your ability to manage intense emotions. Consider your self-esteem. Are they good enough? Do you need to improve? Try psychotherapy if you need help.	TIP OF THE DAY<br><br>Reframing…<br><br>It is not situations that cause us stress, but rather what we make of the situations that cause us stress.<br><br>Ways to reframe a challenging situation:<br><br>• Look for lessons to be learned<br>• Look for opportunities to practice skills<br><br>Can you think of other ways to positively reframe stressful situations?<br><br>Practice reframing today. See what you think.	Did you learn something new? Would it be good to discuss with your psychiatrist and maybe your supports?<br><br>Stay well…

Daily Review Feedback Category 26: Staying Well, Lifestyle – Social (Choice 2.6)

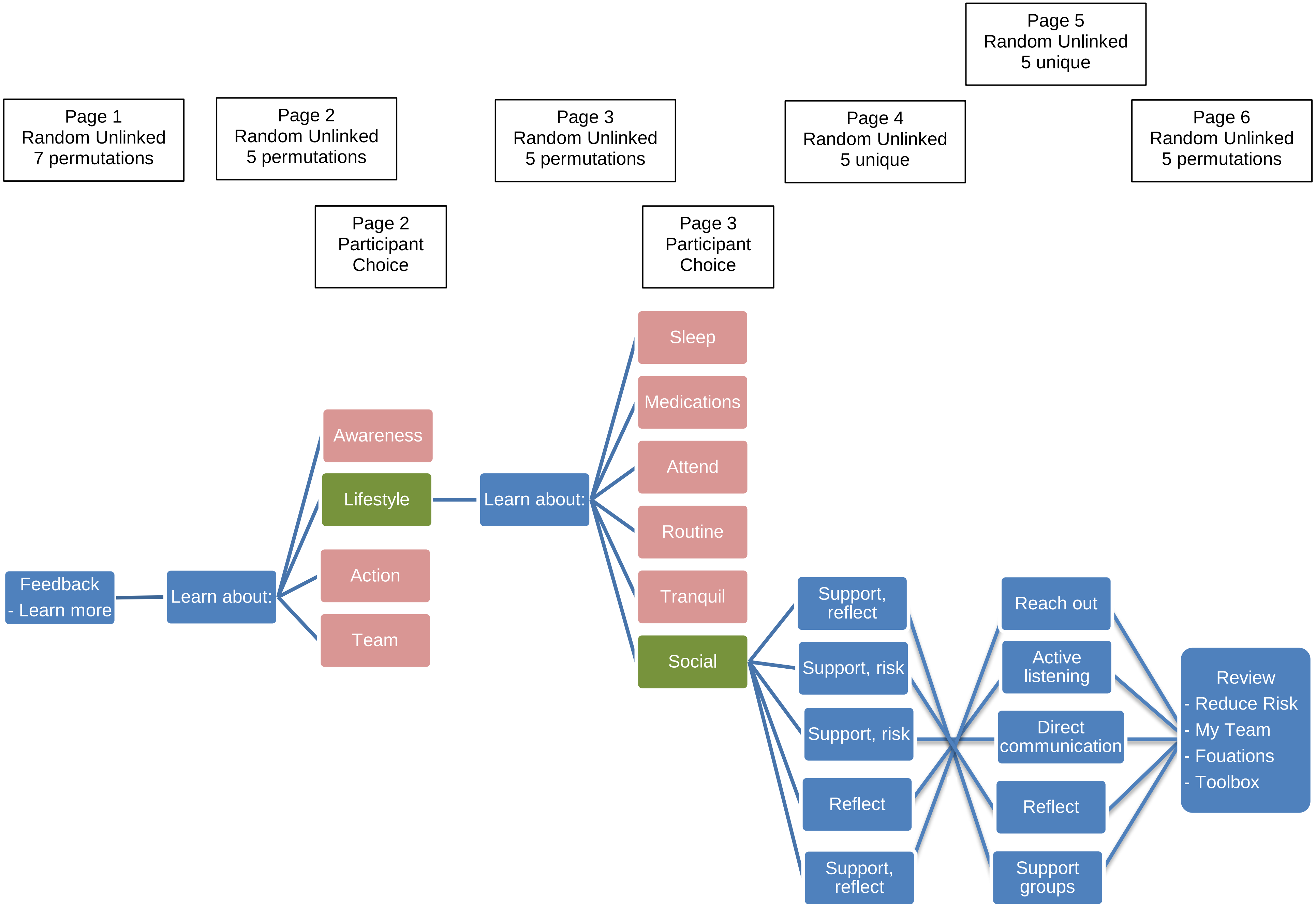

Daily Review Feedback Category 26: Staying Well, Lifestyle – Social (Choice 2.6)

Learn more (P)	Learn about (P1)	Lifestyle (P6)	Support Reflect (U41)	Reach out (U41)	Reduce Risk (P28)
**1**<br>**RANDOM UNLINKED**	**2**<br>**RANDOM UNLINKED**	**3**<br>**RANDOM UNLINKED**	**4**<br>**RANDOM UNLINKED**	**5**<br>**RANDOM UNLINKED**	**6**<br>**RANDOM UNLINKED**
Glad to see you're doing well. That's great!<br><br>Press continue to learn more about staying well.	Learn more now when you're well. You will be ready to take action whenever things get off for you.<br><br>Read about…	Great choice! Maintaining a healthy lifestyle is crucial to staying well. Without it, managing your life and illness is difficult if not impossible.<br><br>Remember the acronym SMARTS: sleep, medications, attend, routine, tranquil, and social. These are the keys to a healthy lifestyle.<br><br>Learn about…	About Socialization<br><br>Are you satisfied with your relationships?<br><br>Social support will keep you healthy! It buffers the impact of life stress. It helps you stay mentally and physically healthy.<br><br>Social support involves:<br><br>• Emotional support such as having someone listen and show interest.<br>• Informational support such as giving guidance and advice.<br>• Tangible support such as offering money or transportation.<br><br>Do you need to expand your network of supports?	TIP OF THE DAY<br><br>Strengthen your social support network. Choose from amongst the ideas below.<br><br>Reach out to a family member or friend and…<br><br>• Offer help<br>• Offer emotional support<br>• Offer information support<br>• Pay a compliment to them<br>• Tell them you care<br>• Ask for emotional support<br>• Ask for information support	Double check your socialization plan in Reduce Risk in the Wellness Plan below. Do you need to make any changes?<br><br>Stay well…

Daily Review Feedback Category 26: Staying Well, Lifestyle – Social (Choice 2.6)

Learn more (P2)	Learn about (P2)	Lifestyle (P7)	Support Risk (U42)	Listening (U42)	Toolbox (P29)
**1**<br>**RANDOM UNLINKED**	**2**<br>**RANDOM UNLINKED**	**3**<br>**RANDOM UNLINKED**	**4**<br>**RANDOM UNLINKED**	**5**<br>**RANDOM UNLINKED**	**6**<br>**RANDOM UNLINKED**
Good to see you're well.<br><br>Continue to learn more about staying well.	Read more about the keys to staying well.<br><br>Check out…	Maintaining a healthy lifestyle is one of the foundations of staying well.<br><br>Learn more…	About Socialization<br><br>Feeling connected to others is vital to health. Social support is vital to health.<br><br>Social problems can trigger symptoms. And symptoms can cause social problems. The withdrawal in depression can push others away. The irritability in mania can push others away.<br><br>Work to have good relationships with others!	TIP OF THE DAY<br><br>Active listening…<br><br>Being a good listener is easier said than done! Here are the keys to being a good listener:<br><br>• Pay attention and show it (nod, make eye contact, sit forward)<br>• Be encouraging with small gestures ("ok", "mmm")<br>• Ask questions to show interest and to learn more<br>• Paraphrase their points to show that you're listening and understand what they're saying<br>• Reflect their feelings to show you hear and got it right<br><br>Don't be defensive! No whining, denying responsibility, assuming, or complaining.<br><br>Practice your listening skills today. See what you think.	Check out the Socialization section of Toolbox to learn even more.<br><br>Stay well…

Daily Review Feedback Category 26: Staying Well, Lifestyle – Social (Choice 2.6)

Learn more (P3)	Learn about (P3)	Lifestyle (P8)	Support Risk (U43)	Communication (U43)	Foundations (P14)
**1**<br>**RANDOM UNLINKED**	**2**<br>**RANDOM UNLINKED**	**3**<br>**RANDOM UNLINKED**	**4**<br>**RANDOM UNLINKED**	**5**<br>**RANDOM UNLINKED**	**6**<br>**RANDOM UNLINKED**
You say you're doing well. That's good.<br><br>Continue on to learn more about staying well.	Being aware, living a healthy lifestyle, coping with symptoms, and having a good team in place will help you stay well.<br><br>Read more about…	Good choice. Most people find it tough to consistently maintain a healthy lifestyle.<br><br>Don't aim for perfection! Do the best you can, and when you get off track try to get back on track as quickly as possible.<br><br>Learn about…	About Socialization<br><br>We are social beings. We need to feel connected to others. We need support.<br><br>Social problems cause stress. They can trigger symptoms. Also, symptoms can cause social problems. The withdrawal of depression can push others away. The irritability of mania can push others away.<br><br>Work to have caring relationships with others. Work to stay connected in healthy ways, even when having symptoms. If you need help consider psychotherapy or family therapy.	TIP OF THE DAY<br><br>Respectful messaging…<br><br>Communicating effectively is easier said than done!<br><br>Be COURTEOUS and DIRECT when you ask for favors, disagree, expressive positive feelings, express negative feelings, refuse requests, respond to disagreements, respond to positive feelings, and respond to negative feelings.<br><br>• Keep it simple<br>• Keep it in the here and now<br>• Use "I" statements<br>• Voice your preferences<br><br>Practice respectful messaging today. See what you think.	Review the module "Lifestyle Skills" in Foundations.<br><br>Stay well…

Daily Review Feedback Category 26: Staying Well, Lifestyle – Social (Choice 2.6)

Learn more (P4)	Learn about (P4)	Lifestyle (P9)	Reflect (U44)	Reflect (U44)	(P5)
**1**<br>**RANDOM UNLINKED**	**2**<br>**RANDOM UNLINKED**	**3**<br>**RANDOM UNLINKED**	**4**<br>**RANDOM UNLINKED**	**5**<br>**RANDOM UNLINKED**	**6**<br>**RANDOM UNLINKED**
Looks like you're doing well, which is great.<br><br>Continue to learn more about staying well.	Now is a good time to learn more about staying well. There are four areas on which you can focus.<br><br>Read about…	Getting good sleep, taking medications, staying abstinent from drugs and alcohol, having a regular routine, aiming for tranquility, and having a good social life are important aspects of staying well.<br><br>Learn more about…	About Socialization<br><br>Get out a piece of paper and pencil. For each item below write down two things. First, list all the people in your life who fit in each of the categories. Second, rate how satisfied you are with each person listed.<br><br>□ Who can you count on to be dependable when you need help?<br>□ Who can you count on to help you feel more relaxed when you are under pressure or tense?<br>□ Who accepts you the way you are, with your strengths and weaknesses?<br>□ Who can you count on to care about you, regardless of the situation?<br>□ Who can you count on to help you feel better when you are feeling generally down---in---the---dumps?<br>□ Who can you count on to console you when you are upset?<br><br>Do you have enough supports in your life? Are you satisfied with the supports in your life?<br><br>Do you need to work on further developing your support network?	TIP OF THE DAY<br><br>Consider your network of family and friends. Are you satisfied?<br><br>Do you need more people in your life? Do you need different people in your life? Do you need to develop deeper relationships with the people you do have in your life?<br><br>Lack of social support is a problem to be solved. It is of high priority as your health depends on it.<br><br>Be creative, find ways to get more support if this area of your life is lacking.	Stay well…

Daily Review Feedback Category 26: Staying Well, Lifestyle – Social (Choice 2.6)

Learn more (P5)	Learn about (P5)	Lifestyle (P10)	Support Reflect (U45)	Groups (U45)	My Team (P21)
**1**<br>**RANDOM UNLINKED**	**2**<br>**RANDOM UNLINKED**	**3**<br>**RANDOM UNLINKED**	**4**<br>**RANDOM UNLINKED**	**5**<br>**RANDOM UNLINKED**	**6**<br>**RANDOM UNLINKED**
Great to see you're well.<br><br>Continue for strategies to help you stay on track.	There are four key areas to focus on in order to stay well.<br><br>Read about…	The ability to maintain a healthy lifestyle is related to fewer illness relapses. Think about ways to take care of your body and mind!<br><br>Read about…	About Socialization<br><br>One of the key factors for living a happy life is having positive relationships.<br><br>This means you have warm, satisfying, and trusting relationships with others. It means you are concerned about the welfare of others. It means you are capable of empathy, affection, intimacy, and mutuality.<br><br>Having positive relationships means that you feel connected intimately with those close to you as well as with the larger world around you.<br><br>Are you satisfied with your relationships? Do you need to make changes in this area of your life?	TIP OF THE DAY<br><br>Consider joining a DBSA support group. There are online and local (in person) groups available to you.<br><br>Check it out. See what you think.	Did you learn something new? Would it be good to discuss with your psychiatrist and maybe your supports?<br><br>Stay well…

Daily Review Feedback Category 26: Staying Well, Action – Dial Up (Choice 3.1)

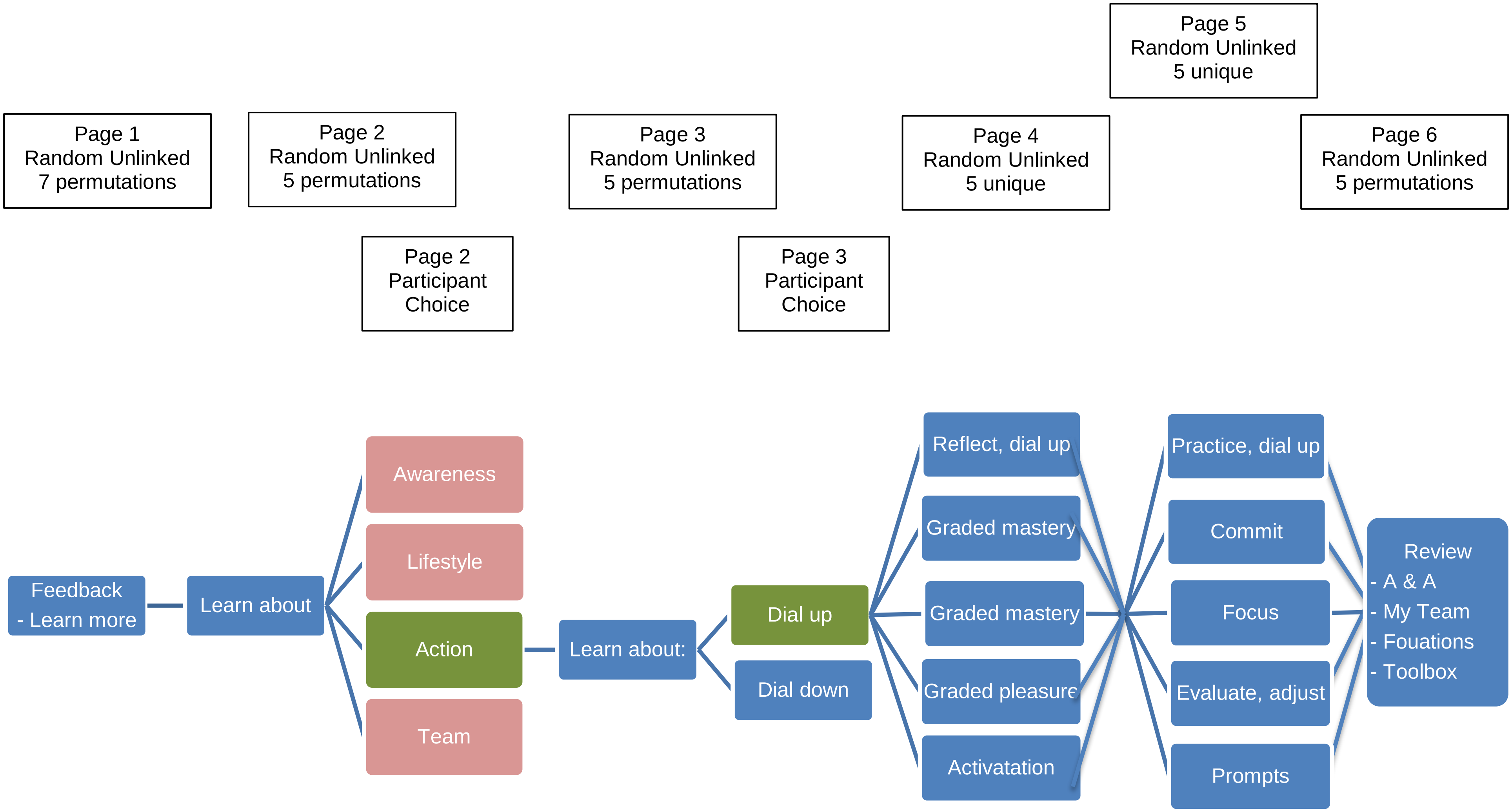

Daily Review Feedback Category 26: Staying Well, Action – Dial Up (Choice 3.1)

Learn more (P1)	Learn about (P1)	Learn about (P11)	Reflect Dial Up (U46)	Practice (U46)	A & A (P30)
**1**<br>**RANDOM UNLINKED**	**2**<br>**RANDOM UNLINKED**	**3**<br>**RANDOM UNLINKED**	**4**<br>**RANDOM UNLINKED**	**5**<br>**RANDOM UNLINKED**	**6**<br>**RANDOM UNLINKED**
Glad to see you're doing well. That's great!<br><br>Press continue to learn more about staying well.	Learn more now when you're well. You will be ready to take action whenever things get off for you.<br><br>Read about…	Great choice! Having an action plan is crucial to good self---care. Without it, managing your life and illness becomes tricky.<br><br>Coping with low level mood symptoms allows you to live a healthier life.<br><br>Learn about…	When you're depressed do you feel unmotivated? Is your energy low?<br><br>These are very common experiences.<br><br>Getting moving can actually counteract these symptoms. The term "dial up" means to increase your energy. In the most basic way, this means MOVE your body!<br><br>Dial up skills are important to use whenever you have early warning signs or symptoms of depression. Moving can reduce depression.<br><br>It works!	**TIP OF THE DAY**<br><br>Practice dialing up and dialing down.<br><br>1. Write down your energy level on a scale of 1 to 10.<br>2. Go for a brisk 5- minute walk around the block.<br>3. Record your energy level again. Did it go up?<br>4. Now sit and close your eyes. Take slow, deep breaths for 2 minutes.<br>5. Record your energy level again. Did it go down?<br><br>If these exercises didn't get your energy level up and down, try something else.	Double check your action plan for symptoms (mild up and down) listed in Awareness and Action. Do you need to make any additions or changes?<br><br>Stay well…

Daily Review Feedback Category 26: Staying Well, Action – Dial Up (Choice 3.1)

Learn more (P2) | Learn about (P2) | Learn about (P12) | Mastery (U47) | Commit (U47) | Toolbox (P31)

1<br>RANDOM UNLINKED	2<br>RANDOM UNLINKED	3<br>RANDOM UNLINKED	4<br>RANDOM UNLINKED	5<br>RANDOM UNLINKED	6<br>RANDOM UNLINKED
Good to see you're well.<br><br>Continue to learn more about staying well.	Read more about the keys to staying well.<br><br>Check out…	Action is one of the foundations of staying well.<br><br>Become more aware of…	**GRADED MASTERY**<br><br>Success repairs mild drops in mood. When starting to feel down, try to succeed at a small task but be SMART. Start easy and slow. But go!<br><br>• **S**pecific: Make goals very specific and clear. Make them concrete.<br>• **M**eaningful: Make goals that are important to you.<br>• **A**ction-based: Base goals on effort not outcome. Effort is in your control.<br>• **R**ealistic: Make sure you can do it. Aim for a 95% chance of success.<br>• **T**imely: Include a time frame. Make a schedule.<br><br>EVERY SUCCESS COUNTS! GO SLOW!	**GETTING STARTED**<br><br>Getting started is oftentimes the hardest part. Here are some different things you can do to make things easier:<br><br>• Keep your eye on the prize. Remember how this is going to help you. Remember your commitment.<br>• Think about how good you'll feel if you take action. (Don't focus on how good you'll feel if you do something else instead.)<br>• Commit to doing just 5 minutes, at least to start. Everything counts.<br>• Start by getting yourself in place. Gather your materials. Set up.<br>• Just start moving. Literally, just start moving your body.	Check out the self---assessment section of Skills to learn even more about coping with symptoms.<br><br>Stay well…

Daily Review Feedback Category 26: Staying Well, Action – Dial Up (Choice 3.1)

Learn more (P3)	Learn about (P3)	Learn about (P13)	Mastery (U48)	Focus (U48)	Foundations (P32)
**1**<br>**RANDOM UNLINKED**	**2**<br>**RANDOM UNLINKED**	**3**<br>**RANDOM UNLINKED**	**4**<br>**RANDOM UNLINKED**	**5**<br>**RANDOM UNLINKED**	**6**<br>**RANDOM UNLINKED**
You say you're doing well. That's good.<br><br>Continue on to learn more about staying well.	Being aware, living a healthy lifestyle, coping with symptoms, and having a good team in place will help you stay well.<br><br>Read more about…	Good choice. Most people find it tricky to cope with symptoms. It involves action in opposition to how you feel.<br><br>Learn to recognize…	**GRADED MASTERY**<br><br>Accomplishments repair mild drops in mood. Start easy and slow. But go!<br><br>• Eat a healthy meal<br>• Exercise for any length of time<br>• Take a shower and dress nicely<br>• Finish a chore around home<br>• Finish a task for school<br>• Finish a task at work<br><br>Make a checklist of things that make you feel capable. Start by doing one easy thing. Then do another.<br><br>START EASY! GO SLOW!	**STAYING FOCUSED**<br><br>Focus on the task at hand. Gently let go of any:<br><br>• Intrusive thoughts<br>• Distractions<br>• Competing impulses<br>• Temptations<br>• Thoughts of old habits<br><br>Trying NOT to think about something will make you want to think about it more. So don't try to not think of these things. Instead…<br><br>• Zoom in on the task at hand.<br>• Gently bring your mind back to the task at hand anytime it wanders (as it will).	Review the module "Coping Skills" in Foundations.<br><br>Stay well…

Daily Review Feedback Category 26: Staying Well, Action – Dial Up (Choice 3.1)

Learn more (P6)	Learn about (P4)	Learn about (P14)	Pleasure (U49)	Evaluate Adjust (U49)	(P5)
**1**<br>**RANDOM UNLINKED**	**2**<br>**RANDOM UNLINKED**	**3**<br>**RANDOM UNLINKED**	**4**<br>**RANDOM UNLINKED**	**5**<br>**RANDOM UNLINKED**	**6**<br>**RANDOM UNLINKED**
Looks like you're doing well. Good job.<br><br>Learn more about staying well.	Now is a good time to learn more about staying well. There are four areas on which you can focus.<br><br>Read about...	Awareness and action are important aspects of staying well.<br><br>Coping with mild ups and downs helps tings from getting really bad.<br><br>Learn to ...	**GRADED PLEASURE**<br><br>Having fun repairs mild drops in mood. Start <u>easy</u> and slow. But go!<br><br>• Take a bath<br>• Watch a comedy<br>• Spend time with your pet<br>• Have a cup of tea<br>• Get a massage<br>• Play a game of chess<br><br>Make a checklist of things that you enjoy doing when well. Start by doing one easy thing. Then do another.<br><br>START SIMPLE! <u>GO</u> SLOW!	**EVALUATING OUTCOMES**<br><br>Evaluate your effort. Evaluate the outcome. How did things go?<br><br>If you succeeded, take a look at the link between your effort and the outcome. Give yourself credit for a job well done.<br><br>If you did not succeed, then focus on what you can learn. Don't judge, but aim to understand.<br>Where did things break down?<br><br>• In preparing: Were you not motivated? What do you need to do?<br>• In planning: Do you need to make changes to your plan?<br>• In performing: What got in the way? What can you do differently next time?<br><br>There is no failure. You either succeed in making changes or you succeed in learning something from the experience.	Stay well...

Daily Review Feedback Category 26: Staying Well, Action – Dial Up (Choice 3.1)

Learn more (P7)	Learn about (P5)	Learn about (P15)	Activation (U50)	Prompts (U50)	My Team (P21)
**1<br>RANDOM UNLINKED**	**2<br>RANDOM UNLINKED**	**3<br>RANDOM UNLINKED**	**4<br>RANDOM UNLINKED**	**5<br>RANDOM UNLINKED**	**6<br>RANDOM UNLINKED**
Nice to see you're well.<br><br>Learn more about staying well. Continue…	There are four key areas to focus on in order to stay well.<br><br>Read about…	The ability to cope with low level mood symptoms is related to a better quality of life.<br><br>Do you have a good handle on coping with symptoms? If not, read more about it.	**ACTIVATING YOUR BODY**<br><br>Moving your body repairs mild drops in mood. Start easy and slow. But go!<br><br>MOVE…<br><br>• Stretch your body<br>• Lift some weights<br>• Go to a yoga class<br>• Go for a short walk<br><br>Start with something manageable, maybe just 5 to 15 minutes. Gradually increase the time you move each hour or each day.<br><br>NO JUDGEMENT! GROUND YOURSELF!	**USING CUES**<br><br>Cues facilitate change! Cues can be a wide range of things. Be creative. Set it up so you see them or hear them without much effort on your part.<br><br>• Notes. Post a note somewhere clearly visible that you will see around the time you are scheduled to act.<br>• Alarms. Set a reminder on your phone at or before the time you are to act.<br>• Routines. Build your activity into your routine so you cannot forget.<br>• Materials. Place materials you need to use in clear view (e.g., running clothes).<br><br>We are creatures of habit. Use your mind and your environment to try something new.	Did you learn something new? Anything you want to share with your psychiatrist or supports?<br><br>Stay well…

Daily Review Feedback Category 26: Staying Well, Action – Dial Down (Choice 3.2)

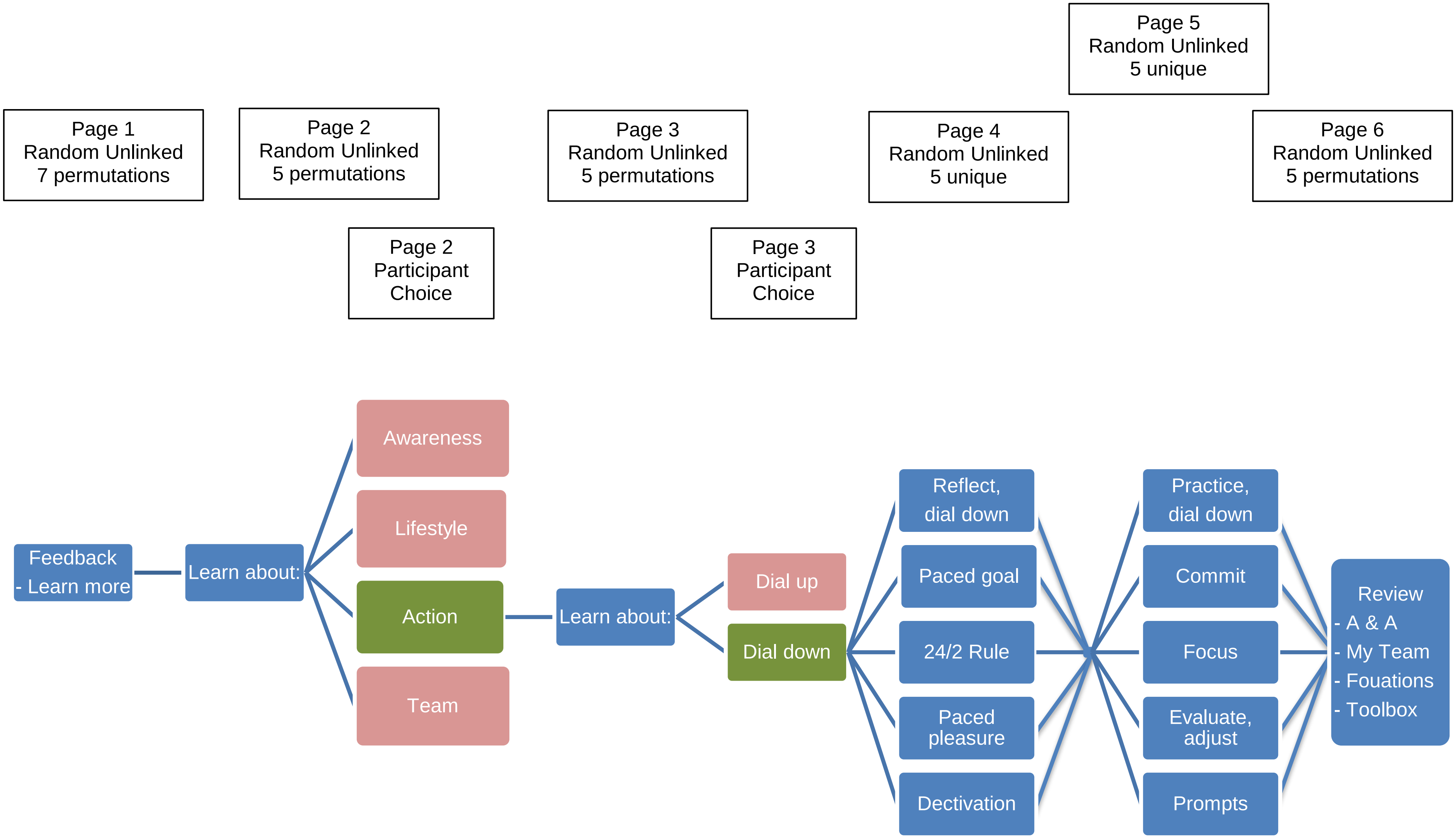

Daily Review Feedback Category 26: Staying Well, Action – Dial Down (Choice 3.2)

Learn more (P1)	Learn about (P1)	Learn about (P11)	Reflect Dial Down (U51)	Practice (U51)	A & A (P30)
**1**<br>**RANDOM UNLINKED**	**2**<br>**RANDOM UNLINKED**	**3**<br>**RANDOM UNLINKED**	**4**<br>**RANDOM UNLINKED**	**5**<br>**RANDOM UNLINKED**	**6**<br>**RANDOM UNLINKED**
Glad to see you're doing well. That's great!<br><br>Press continue to learn more about staying well.	Learn more now when you're well. You will be ready to take action whenever things get off for you.<br><br>Read about…	Great choice! Having an action plan is crucial to good self--- care. Without it, managing your life and illness becomes tricky.<br><br>Coping with low level mood symptoms allows you to live a healthier life.<br><br>Learn about…	When you're manic do you feel really motivated? Is your energy high?<br><br>These are very common experiences.<br><br>Slowing down can actually counteract these symptoms. The term "dial down" means to decrease your energy. In the most basic way, this means SLOW your body!<br><br>Dial down skills are important to use whenever you have early warning signs or symptoms of mania. Slowing can reduce mania.<br><br>It works!	TIP OF THE DAY<br><br>Practice dialing up and dialing down.<br><br>1. Write down your energy level on a scale of 1 to 10.<br>2. Go for a brisk 5--- minute walk around the block.<br>3. Record your energy level again. Did it go up?<br>4. Now sit and close your eyes. Take slow, deep breaths for 2 minutes.<br>5. Record your energy level again. Did it go down?<br><br>If these exercises didn't get your energy level up and down, try something else.	Double check your action plan for symptoms (mild up and down) listed in Awareness and Action in the Wellness Plan below.<br><br>Do you need to make any additions or changes?<br><br>Stay well…

Daily Review Feedback Category 26: Staying Well, Action – Dial Down (Choice 3.2)

Learn more (P2)	Learn about (P2)	Learn about (P12)	Paced Goal (U52)	Commit (U47)	Toolbox (P31)

1<br>RANDOM UNLINKED	2<br>RANDOM UNLINKED	3<br>RANDOM UNLINKED	4<br>RANDOM UNLINKED	5<br>RANDOM UNLINKED	6<br>RANDOM UNLINKED
Good to see you're well.<br><br>Continue to learn more about staying well.	Read more about the keys to staying well.<br><br>Check out...	Action is one of the foundations of staying well.<br><br>Become more aware of...	**PACED GOALS**<br><br>Slowing your body repairs mild ups in mood. PACE yourself. Slow down!<br><br>Not everything is urgent. Set goals so that you don't overdo it:<br><br>• **<u>P</u>**riority: Stick to high priority tasks. Everything cannot be important.<br>• **<u>A</u>**ttitude: Stay mindful and grounded. Not all ideas are great in the long run.<br>• **<u>C</u>**ommonsensical: Make sure the tasks fit into your life plan.<br>• **<u>E</u>**ven: Keep a balance of activity and rest. Go slower than you are inclined.<br><br>BE THOUGHTFUL. GO <u>SLOW</u>!	**GETTING STARTED**<br><br>Getting started is oftentimes the hardest part. Here are some different things you can do to make things easier:<br><br>• Keep your eye on the prize. Remember how this is going to help you. Remember your commitment.<br>• Think about how good you'll feel if you take action. (Don't focus on how good you'll feel if you do something else instead.)<br>• Commit to doing just 5 minutes, at least to start. Everything counts.<br>• Start by getting yourself in place. Gather your materials. Set up.<br>• Just start moving. Literally, just start moving your body.	Check out the self---assessment section of Toolbox to learn even more about coping with symptoms.<br><br>Stay well...

Daily Review Feedback Category 26: Staying Well, Action – Dial Down (Choice 3.2)

Learn more (P3)	Learn about (P3)	Learn about (P13)	24/2 Rule (U53)	Focus (U48)	Foundations (P32)
**1**<br>**RANDOM UNLINKED**	**2**<br>**RANDOM UNLINKED**	**3**<br>**RANDOM UNLINKED**	**4**<br>**RANDOM UNLINKED**	**5**<br>**RANDOM UNLINKED**	**6**<br>**RANDOM UNLINKED**
You say you're doing well. That's good.<br><br>Continue on to learn more about staying well.	Being aware, living a healthy lifestyle, coping with symptoms, and having a good team in place will help you stay well.<br><br>Read more about…	Good choice. Most people find it tricky to cope with symptoms. It involves action in opposition to how you feel.<br><br>Learn to …	**PACED MASTERY**<br><br>Mania can cause you to think your ideas are fantastic. That is not to say that you don't have good ideas at times. But, when your mood is too elevated, you need to be careful. Mania "tricks" you into thinking in exaggerated ways. It is important to be cautious. Slow down!<br><br>When up and excited about an idea, try the 24/2 check:<br><br>• Wait twenty four hours and then reassess<br>• Ask two people what they think<br><br>BE WISE. GO SLOW!	**STAYING FOCUSED**<br><br>Focus on the task at hand. Gently let go of any:<br><br>• Intrusive thoughts<br>• Distractions<br>• Competing impulses<br>• Temptations<br>• Thoughts of old habits<br><br>Trying NOT to think about something will make you want to think about it more. So don't try to not think of these things. Instead…<br><br>• <u>Zoom in</u> on the task at hand.<br>• Gently bring your mind back to the task at hand anytime it wanders (as it will).	Review the module "Coping Skills" in Foundations.<br><br>Stay well…

Daily Review Feedback Category 26: Staying Well, Action – Dial Down (Choice 3.2)

Learn more (P6)	Learn about (P4)	Learn about (P14)	Pleasure (U54)	Evaluate Adjust (U49)	(P5)
**1**<br>**RANDOM UNLINKED**	**2**<br>**RANDOM UNLINKED**	**3**<br>**RANDOM UNLINKED**	**4**<br>**RANDOM U N LINKED**	**5**<br>**RANDOM U N LINKED**	**6**<br>**RANDOM UNLINKED**
Looks like you're doing well. Good job.<br><br>Learn more about staying well.	Now is a good time to learn more about staying well. There are four areas on which you can focus.<br><br>Read about…	Awareness and action are important aspects of staying well.<br><br>Coping with mild ups and downs helps tings from getting really bad.<br><br>Learn to be more aware of…	**PACED PLEASURE**<br><br>If you are like others, you may have a tendency to engage in risky activities when your mood is up. Risky actions include things like spending lots of money or being promiscuous.<br><br>Are there things you tend to do when manic that you end up regretting later on? If so, build some safeguards for when your mood starts to elevate. For example:<br><br>• Give your credit cards to someone<br>• Stay with a friend and don't go out<br>• Remove alcohol from your home<br>• Close your Facebook account<br><br>BE SAFE. GO SLOW!	**EVALUATING OUTCOMES**<br><br>Evaluate your effort. Evaluate the outcome. How did things go?<br><br>If you succeeded, take a look at the link between your effort and the outcome. Give yourself credit for a job well done.<br><br>If you did not succeed, then focus on what you can learn. Don't judge, but aim to understand.<br>Where did things break down?<br><br>• In preparing: Were you not motivated? What do you need to do?<br>• In planning: Do you need to make changes to your plan?<br>• In performing: What got in the way? What can you do differently next time?<br><br>There is no failure. You either succeed in making changes or you succeed in learning something from the experience.	Stay well…

Daily Review Feedback Category 26: Staying Well, Action – Dial Down (Choice 3.2)

Learn more (P7)	Learn about (P5)	Learn about (P15)	Deactivation (U55)	Prompts (U50)	My Team (P21)
**1**<br>**RANDOM UNLINKED**	**2**<br>**RANDOM UNLINKED**	**3**<br>**RANDOM UNLINKED**	**4**<br>**RANDOM UNLINKED**	**5**<br>**RANDOM UNLINKED**	**6**<br>**RANDOM UNLINKED**
Nice to see you're well.<br><br>Learn more about staying well. Continue…	There are four key areas to focus on in order to stay well.<br><br>Read about…	The ability to cope with low level mood symptoms is related to a better quality of life.<br><br>Do you have a good handle on coping with symptoms? If not, read more about it.	**DEACTIVATING YOUR BODY**<br><br>Slowing your body repairs mild ups in mood. Put on the brakes. Get your body to be quiet on the inside. Be still!<br><br>RELAX…<br><br>• Take deep breaths<br>• Stretch your body<br>• Try muscle relaxation<br>• Do yoga at home<br><br>Try to relax for a full day. It will be challenging at first, but you can do it.<br><br>CALM YOUR BODY. GROUND YOURSELF!	**USING CUES**<br><br>Cues facilitate change! Cues can be a wide range of things. Be creative. Set it up so you see them or hear them without much effort on your part.<br><br>• Notes. Post a note somewhere clearly visible that you will see around the time you are scheduled to act.<br>• Alarms. Set a reminder on your phone at or before the time you are to act.<br>• Routines. Build your activity into your routine so you cannot forget.<br>• Materials. Place materials you need to use in clear view (e.g., running clothes).<br><br>You are a creature of habit. Use your mind and your environment to try something new.	Did you learn something new? Anything you want to share with your psychiatrist or supports?<br><br>Stay well…

Daily Review Feedback Category 26: Staying Well, Team – Providers (Choice 4.1)

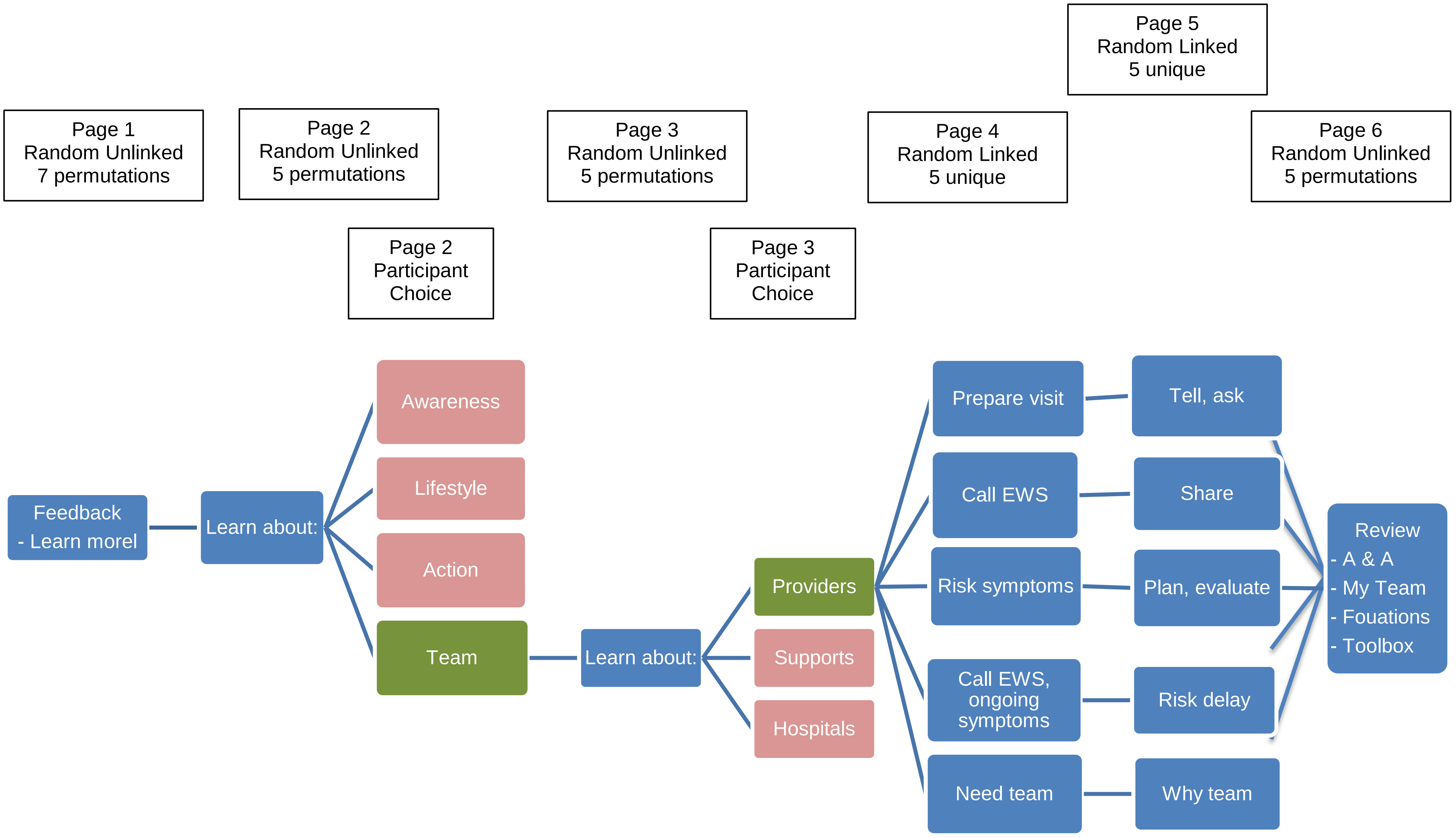

Daily Review Feedback Category 26: Staying Well, Team – Providers (Choice 4.1)

Learn more (P1)	Learn about (P1)	Learn about (P16)	Prepare (U56)	Share Ask (U52)	My Team (P33)
**1**<br>**RANDOM UNLINKED**	**2**<br>**RANDOM UNLINKED**	**3**<br>**RANDOM UNLINKED**	**4**<br>**RANDOM LINKED**	**5**<br>**RANDOM LINKED**	**6**<br>**RANDOM UNLINKED**
Glad to see you're doing well. That's great!<br><br>Press continue to learn more about staying well.	Learn more now when you're well. You will be ready to take action whenever things get off for you.<br><br>Read about…	Great choice! Forming an effective team is crucial to staying well. Without it, managing your life and illness is difficult if not impossible.<br><br>Having a good relationship with your psychiatrist, having good supports in place, and having a hospital identified in advance (just in case) allow you to stay on top of your health.<br><br>Learn about…	**GENERAL GUIDELINES**<br><br>Having a good relationship with your psychiatrist is important. They are there for more than writing prescriptions. You need to feel comfortable with them and trust them. You need to be able to be open and honest with them. You need to be able to reach out to them when you have symptoms.<br><br>When preparing for appointments, it can be helpful to make a list of things you want to tell your psychiatrist and any questions you may have.	Things to tell your psychiatrist:<br><br>• Symptoms<br>• Sleep habits<br>• Medication use<br>• Substance use<br>• Significant life changes<br><br>Things to ask your psychiatrist:<br><br>• What is my diagnosis?<br>• Do you think I'm having symptoms now?<br>• How will the medications help?<br>• What are the side effects?<br>• What should I do if I get side effects?<br>• What should I do if I miss a dose?	Double check the information you have about your team in My Resources.<br><br>Do you need to make any changes?<br><br>Stay well…

Daily Review Feedback Category 26: Staying Well, Team – Providers (Choice 4.1)

Learn more (P2)	Learn about (P2)	Learn about (P17)	Call (U57)	Share (U53)	Toolbox (P34)

1<br>RANDOM UNLINKED	2<br>RANDOM UNLINKED	3<br>RANDOM UNLINKED	4<br>RANDOM LINKED	5<br>RANDOM LINKED	6<br>RANDOM UNLINKED
Good to see you're well.<br><br>Continue to learn more about staying well.	Read more about the keys to staying well.<br><br>Check out…	Having a good team is one of the foundations of staying well.<br><br>Learn more…	**EARLY WARNING SIGNS**<br><br>Be sure to call your psychiatrist any time you have early warning signs of mania or depression for more than 2 or 3 days in a row.<br><br>Try not to downplay things. It is common for people to think they can deal with it on their own.<br><br>Symptoms are serious business. The sooner you act the better. The longer symptoms go on the greater the chance of a major mood episode.<br><br>Act swiftly, get help, and get well!	Things to share:<br><br>• How you are taking your medications<br>• How you are sleeping<br>• How you are spending your time<br>• Any alcohol or drug use<br><br>And:<br><br>• How you are taking care of yourself<br>• How relationships are going<br>• How work (school) is going<br>• Feedback you are getting from others	Check out the Team section of the Toolbox to learn even more about using a team effectively.<br><br>Stay well…

Daily Review Feedback Category 26: Staying Well, Team – Providers (Choice 4.1)

Learn more (P3)	Learn about (P3)	Learn about (P18)	Risk (U58)	Plan Evaluate (U54)	Foundations (P35)
**1**<br>**RANDOM UNLINKED**	**2**<br>**RANDOM UNLINKED**	**3**<br>**RANDOM UNLINKED**	**4**<br>**RANDOM LINKED**	**5**<br>**RANDOM LINKED**	**6**<br>**RANDOM UNLINKED**
You say you're doing well. That's good.<br><br>Continue on to learn more about staying well.	Being aware, living a healthy lifestyle, coping with symptoms, and having a good team in place will help you stay well.<br><br>Read more about…	Good choice. Most people find it tough to use their team regularly and effectively.<br><br>Learn about working with…	**ONGOING SYMPTOMS**<br><br>Be sure that your psychiatrist knows whenever you have new symptoms for more than a couple of days especially if you are having several symptoms of depression or mania each day.<br><br>Ongoing symptoms are not good for your spirits, life, or brain!	**Things to do:**<br><br>• Reach out and call them. Let them know what is going on.<br><br>• Agree on a plan of action together. This might involve a medication change. If you have any reservations about the plan, let them know! A plan is no good unless you are willing and able to follow it.<br><br>• Implement the plan. Evaluate each day whether or not things are getting better.<br><br>• Call your psychiatrist again if your symptoms continue and/or you cannot implement the plan.	Review the module "Using Your Team Effectively" in Foundations.<br><br>Stay well…

Daily Review Feedback Category 26: Staying Well, Team – Providers (Choice 4.1)

Learn more (P4)	Learn about (P4)	Learn about (P19)	Call (U59)	Risk Delay (U55)	(P5)
**1**<br>**RANDOM UNLINKED**	**2**<br>**RANDOM UNLINKED**	**3**<br>**RANDOM UNLINKED**	**4**<br>**RANDOM LINKED**	**5**<br>**RANDOM LINKED**	**6**<br>**RANDOM UNLINKED**
Looks like you're doing well, which is great.<br><br>Continue to learn more about staying well.	Now is a good time to learn more about staying well. There are four areas on which you can focus.<br><br>Read about…	Having good working relationships with your psychiatrist and supports are important aspects of staying well.<br><br>Identifying a hospital in advance is good in the event that you need to go in.<br><br>Learn more about…	**WHEN TO REACH OUT**<br><br>Early warning signs:<br><br>Be sure to contact your psychiatrist if you have early warning signs. A rough guideline is that you call if you have early warning signs 2 or more days in a row.<br><br>Ongoing symptoms:<br><br>Be sure to contact your psychiatrist if you have ongoing symptoms. As a general guideline, call if you have new moderate symptoms 2 or more days in a row, and new mild symptoms 3---4 days in a row.	**DON'T DELAY…**<br><br>Have you heard the phrase "delays are dangerous"? That holds true when it comes to managing symptoms.<br><br>Don't delay. When you are having early warning signs or ongoing symptoms, reach out to your psychiatrist.<br><br>Make sure the two of you have a good plan about what to do and when to touch base next.	Stay well…

Daily Review Feedback Category 26: Staying Well, Team – Providers (Choice 4.1)

Learn more (P5)	Learn about (P5)	Learn about (P20)	Need (U60)	Why (U56)	My Team (P21)
**1**<br>**RANDOM UNLINKED**	**2**<br>**RANDOM UNLINKED**	**3**<br>**RANDOM UNLINKED**	**4**<br>**RANDOM LINKED**	**5**<br>**RANDOM LINKED**	**6**<br>**RANDOM UNLINKED**
Great to see you're well.<br><br>Continue for strategies to help you stay on track.	There are four key areas to focus on in order to stay well.<br><br>Read about…	The ability to use your team effectively is related to fewer illness relapses. Don't be too proud or shy or otherwise reluctant. Reach out!<br><br>Read about…	**WHAT IT TAKES…**<br><br>Managing bipolar disorder most effectively requires taking medications, living a healthy lifestyle, and using coping skills. It also requires having a good "team" in place.<br><br>• Psychiatrist<br>• Personal supports<br>• Good hospital<br><br>For some people a therapist, case manager, and peer support groups are important as well.	**WHY A TEAM…**<br><br>Just like other chronic medical conditions such as diabetes and hypothyroidism, bipolar disorder is not something you can manage on your own.<br><br>Don't make the mistake of thinking you can do it on your own. Don't let pride get in the way of good medical care! (There is no shame anyway in having bipolar disorder.)	Did you learn something new? Anything you want to share with your psychiatrist or supports?<br><br>Stay well…

Daily Review Feedback Category 26: Staying Well, Team – Supports (Choice 4.2)

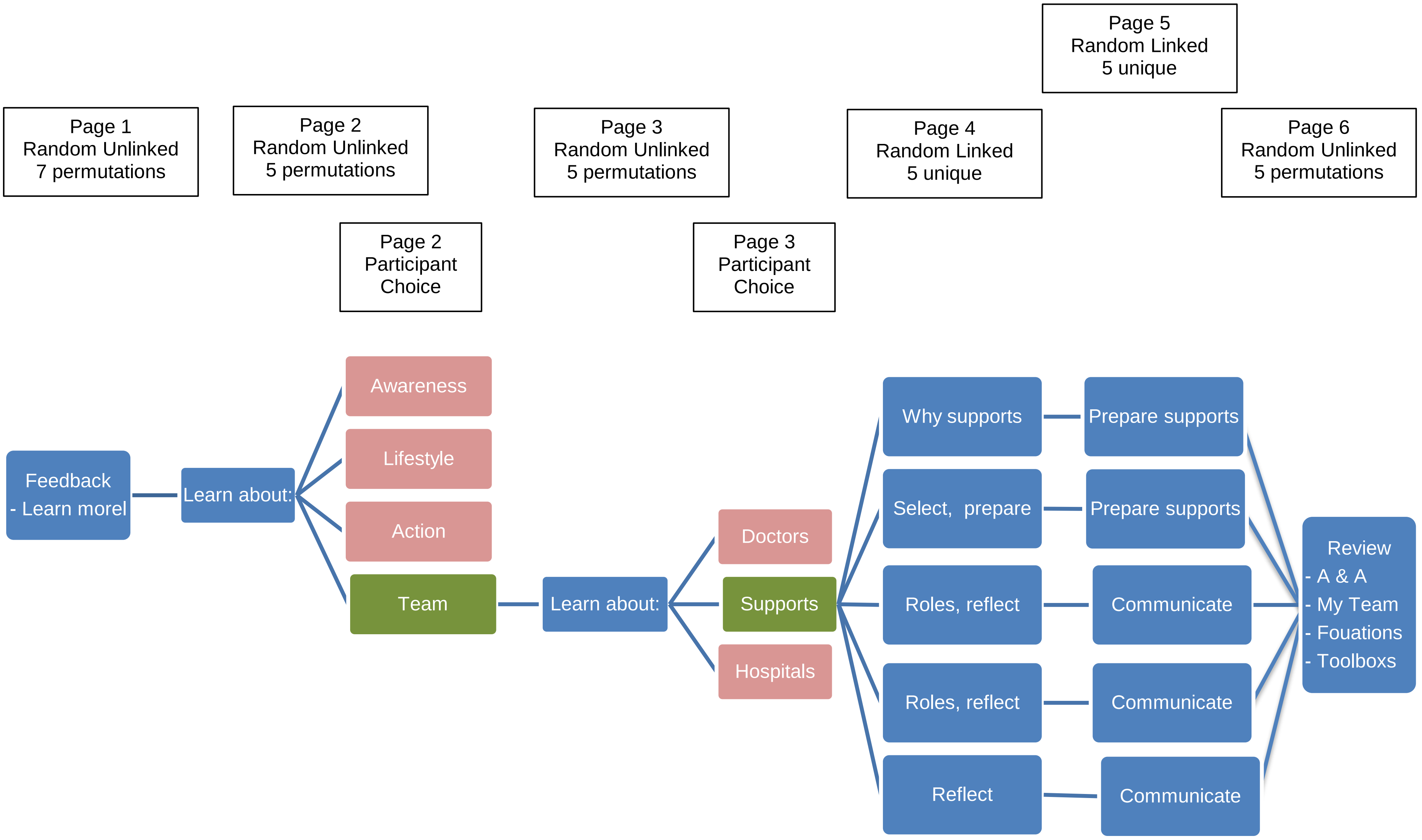

Daily Review Feedback Category 26: Staying Well, Team – Supports (Choice 4.2)

Learn more (P1)	Learn about (P1)	Learn about (P16)	Why (U61)	Prepare (U57)	My Team (P33)
**1**<br>**RANDOM UNLINKED**	**2**<br>**RANDOM UNLINKED**	**3**<br>**RANDOM UNLINKED**	**4**<br>**RANDOM LINKED**	**5**<br>**RANDOM LINKED**	**6**<br>**RANDOM UNLINKED**
Glad to see you're doing well. That's great!<br><br>Press continue to learn more about staying well.	Learn more now when you're well. You will be ready to take action whenever things get off for you.<br><br>Read about…	Great choice! Forming an effective team is crucial to staying well. Without it, managing your life and illness is difficult if not impossible.<br><br>Having a good relationship with your psychiatrist, having good supports in place, and having a hospital identified in advance (just in case) allow you to stay on top of your health.<br><br>Learn about…	**WHY SUPPORTS?**<br><br>There are many reasons to have wellness supports.<br><br>• Tranquility: It is important that you not be alone with your bipolar disorder. Having others know what you have gone through and what you are going through makes things less stressful.<br><br>• Commitment: Letting others know about your intentions to take care of yourself will make it more likely you will stick to your wellness plan.<br><br>• Emotional support: They can provide emotional support. While they are not therapists, they can listen and offer you kindness.<br><br>• Tangible support: They can provide tangible support. You may need, for example, a ride to the hospital if you get ill.<br><br>• Feedback: They can provide feedback. There may be times when you have symptoms that are noticeable to others before they are noticeable to you. They can gently let you know if this happens.	Things to do:<br><br>Agree in advance about when and how you want feedback from your supports.<br><br>Agree in advance about boundaries in the relationship. What are your expectations and limits? What are your supports expectations and limits?<br><br>Remember that supports are not therapists. They should not be telling you what to do, and you should not be relying on them to solve your problems.<br><br>Remember that supports are not parents. They should not be monitoring whether or not you are following your wellness plan, and you should not ask them to do so (unless you have severe symptoms).	Double check the information you have about your team in My Resources.<br><br>Do you need to make any changes?<br><br>Stay well…

Daily Review Feedback Category 26: Staying Well, Team – Supports (Choice 4.2)

Learn more (P2)	Learn about (P2)	Learn about (P17)	Select Prepare (U62)	Prepare (U58)	Toolbox (P34)
**1**<br>**RANDOM UNLINKED**	**2**<br>**RANDOM UNLINKED**	**3**<br>**RANDOM UNLINKED**	**4**<br>**RANDOM LINKED**	**5**<br>**RANDOM LINKED**	**6**<br>**RANDOM UNLINKED**
Good to see you're well.<br><br>Continue to learn more about staying well.	Read more about the keys to staying well.<br><br>Check out…	Having a good team is one of the foundations of staying well.<br><br>Learn more…	**MAKING IT WORK**<br><br>Pick one or two people in your life to support your wellness. Select people who are caring and who you have known for some time. Family members, spouses, and close friends are good.<br><br>Encourage them to read about bipolar disorder. The Depression and Bipolar Support Alliance website is a good resource.<br><br>Meet with them one or two times after they have read the material. Continue for ideas about things to talk about with your supports.	Things to do:<br><br>• Have them read "Basic Facts about Bipolar Disorder" in the Foundations section.<br><br>• Talk with them about what they read. Answer any questions they may have. Consider bringing them to an appointment with your psychiatrist to learn more.<br><br>• Review your *LiveWell* Wellness Plan. Discuss your plan to reduce risk as well as your plan for awareness and action.<br><br>• Discuss how you want them to tell you when you have early warning signs or other symptoms.<br><br>• Talk about what you want them to do if you dismiss, disagree, or argue with them about early warning signs or other symptoms.	Check out the Team section of the Toolbox to learn even more about using a team effectively.<br><br>Stay well…

Daily Review Feedback Category 26: Staying Well, Team – Supports (Choice 4.2)

Learn more (P3)	Learn about (P3)	Learn about (P18)	Roles Reflect (U63)	Communication (U59)	Foundations (P35)
1<br>**RANDOM UNLINKED**	2<br>**RANDOM UNLINKED**	3<br>**RANDOM UNLINKED**	4<br>**RANDOM LINKED**	5<br>**RANDOM LINKED**	6<br>**RANDOM UNLINKED**
You say you're doing well. That's good.<br><br>Continue on to learn more about staying well.	Being aware, living a healthy lifestyle, coping with symptoms, and having a good team in place will help you stay well.<br><br>Read more about…	Good choice. Most people find it tough to use their team regularly and effectively.<br><br>Learn about working with…	**USING SUPPORTS**<br><br>Supports can help you feel more tranquil and committed to your wellness plan. They can also listen, help, and provide feedback.<br><br>All of these things are important in life. They are not restricted to individuals with bipolar disorder!<br><br>Do you have people in your life that you can ask to be a wellness support?	Communicate:<br><br>Here are some ways to start talking about bipolar disorder.<br><br>• Sit down together and look at the Depression and Bipolar Support Alliance website. Discuss what you read. Share your personal experiences. Answer your support's questions.<br><br>• Sit down together and look at the "Basic Facts about Bipolar Disorder" section of Foundations. Discuss what you read. Share your personal experiences. Answer your support's questions.	Review the module "Using Your Team Effectively" in Foundations.<br><br>Stay well…

Daily Review Feedback Category 26: Staying Well, Team – Supports (Choice 4.2)

Learn more (P4)	Learn about (P4)	Learn about (P19)	Roles Reflect (U64)	Communication (U60)	(P5)
**1**<br>**RANDOM UNLINKED**	**2**<br>**RANDOM UNLINKED**	**3**<br>**RANDOM UNLINKED**	**4**<br>**RANDOM LINKED**	**5**<br>**RANDOM LINKED**	**6**<br>**RANDOM UNLINKED**
Looks like you're doing well, which is great.<br><br>Continue to learn more about staying well.	Now is a good time to learn more about staying well. There are four areas on which you can focus.<br><br>Read about…	Having good working relationships with your psychiatrist and supports are important aspects of staying well.<br><br>Identifying a hospital in advance is good in the event that you need to go in.<br><br>Learn more about…	**USING SUPPORTS**<br><br>Supports can help you:<br><br>• Reduce stress by sharing.<br>• Stay on track with your wellness plan<br>• Feel valued.<br>• Become more aware of symptoms through feedback.<br><br>Have you ever used wellness supports before? Can you imagine the benefits? Do you have any concerns?	Communicate:<br><br>Wellness plans are individual. Sit down with your supports and discuss your wellness plan. Review your resources, lifestyle goals, and awareness and action plans.<br><br>Answer any questions they have. Get feedback from them. Is there anything they think you are missing in your plan? Should you make additions or changes to your plan?	Stay well…

Daily Review Feedback Category 26: Staying Well, Team – Supports (Choice 4.2)

Learn more (P5)	Learn about (P5)	Learn about (P20)	Reflect (U65)	Communication (U61)	My Team (P21)
**1**<br>**RANDOM UNLINKED**	**2**<br>**RANDOM UNLINKED**	**3**<br>**RANDOM UNLINKED**	**4**<br>**RANDOM LINKED**	**5**<br>**RANDOM LINKED**	**6**<br>**RANDOM UNLINKED**
Great to see you're well.<br><br>Continue for strategies to help you stay on track.	There are four key areas to focus on in order to stay well.<br><br>Read about…	The ability to use your team effectively is related to fewer illness relapses. Don't be too proud or shy or otherwise reluctant. Reach out!<br><br>Read about…	**USING SUPPORTS**<br><br>Treatment, positive home environments, supports, hope, skills, and strengths are all key to living well.<br><br>Do you have good wellness supports? Do you have one or more people in your personal life that can serve as a good support? If not, join a bipolar support group or get a therapist.<br><br>Everyone needs someone!	Communicate:<br><br>Talk about the expectations and goals of your support relationship. Here are some guidelines:<br><br>It is recommended you reach out and let your supports know when you have moderate or severe symptoms. Let them know how you are going to take care of yourself (including you calling your psychiatrist). Agree on a follow---up contact.<br><br>It is recommended your support reach out to you if they think you are having early warning signs or more serious symptoms. Try to be non---defensive. Agree on a plan of action (including you calling your psychiatrist). Agree on a follow---up contact.	Did you learn something new? Anything you want to share with your psychiatrist or supports?<br><br>Stay well…

Daily Review Feedback Category 26: Staying Well, Team – Hospital (Choice 4.3)

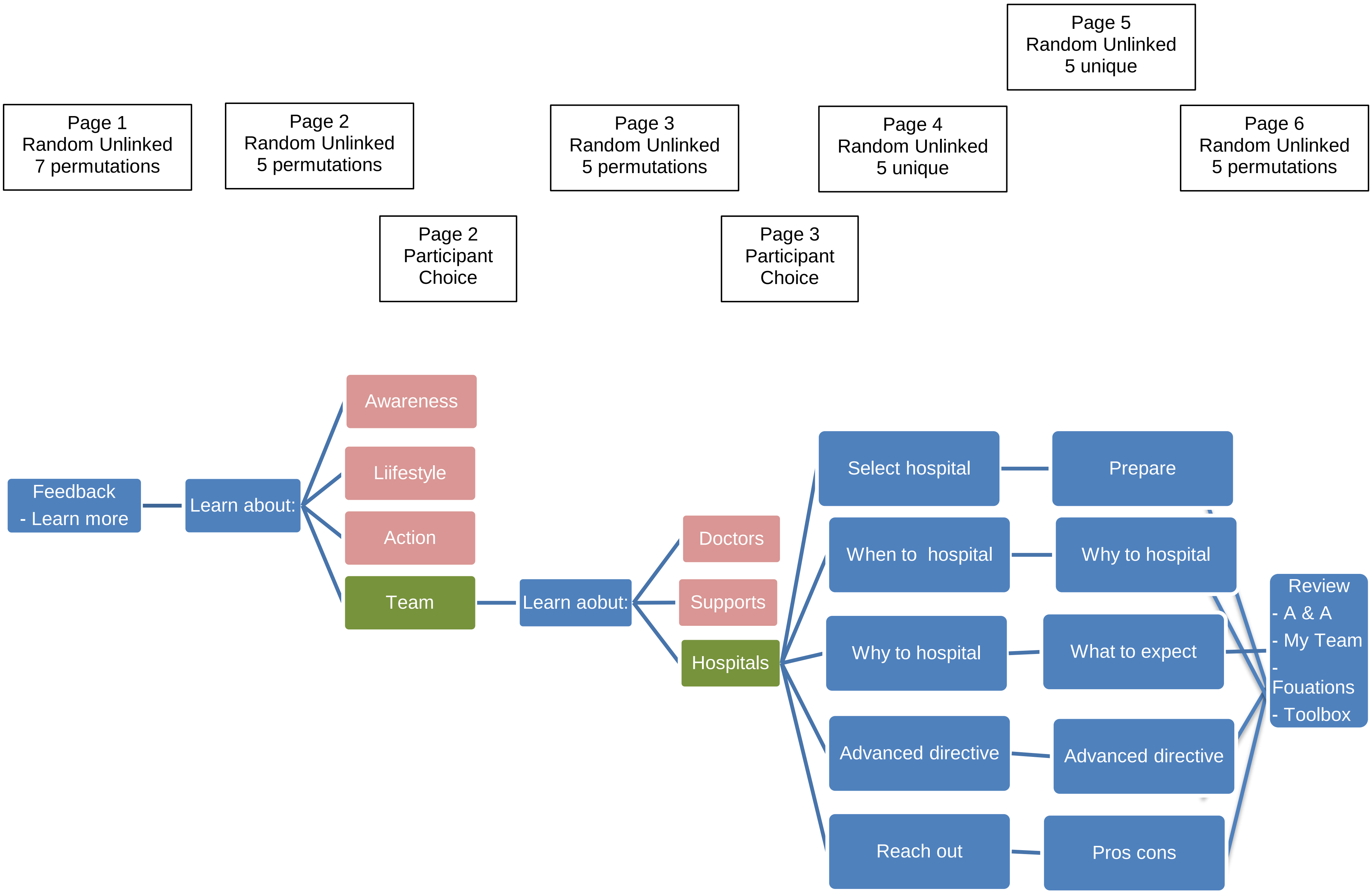

Daily Review Feedback Category 26: Staying Well, Team – Hospital (Choice 4.3)

Learn more (P1)	Learn about (P1)	Learn about (P16)	Select (U66)	Prepare (U62)	My Team (P33)
**1**<br>**RANDOM UNLINKED**	**2**<br>**RANDOM UNLINKED**	**3**<br>**RANDOM UNLINKED**	**4**<br>**RANDOM LINKED**	**5**<br>**RANDOM LINKED**	**6**<br>**RANDOM UNLINKED**
Glad to see you're doing well. That's great!<br><br>Press continue to learn more about staying well.	Learn more now when you're well. You will be ready to take action whenever things get off for you.<br><br>Read about…	Great choice! Forming an effective team is crucial to staying well. Without it, managing your life and illness is difficult if not impossible.<br><br>Having a good relationship with your psychiatrist, having good supports in place, and having a hospital identified in advance (just in case) allow you to stay on top of your health.<br><br>Learn about…	**PICKING A HOSPITAL**<br><br>There are many things to consider when selecting your preferred hospital.<br><br>• What hospitals in your area are ranked the highest? Check out the *US News and World Report.*<br><br>• Is your psychiatrist on staff at an area hospital? Being on staff can help in the coordination of your care.	**BE PROACTIVE**<br><br>Find out the answers to these questions before you ever need to go to the hospital.<br><br>You do not want to be thinking about where to go and what to do in the middle of an emergency. That should all be laid out in advance.	Double check the information you have about your team in My Resources.<br><br>Do you need to make any changes?<br><br>Stay well…

Daily Review Feedback Category 26: Staying Well, Team – Hospital (Choice 4.3)

Learn more (P2)	Learn about (P2)	Learn about (P17)	When (U67)	Why (U63)	Toolbox (P34)
**1**<br>**RANDOM UNLINKED**	**2**<br>**RANDOM UNLINKED**	**3**<br>**RANDOM UNLINKED**	**4**<br>**RANDOM LINKED**	**5**<br>**RANDOM LINKED**	**6**<br>**RANDOM UNLINKED**
Good to see you're well.<br><br>Continue to learn more about staying well.	Read more about the keys to staying well.<br><br>Check out...	Having a good team is one of the foundations of staying well.<br><br>Learn more...	**WHEN TO GO TO THE HOSPITAL**<br><br>Inpatient hospitalization is used to treat serious psychiatric problems.<br><br>Go to the hospital whenever:<br><br>• You are thinking about suicide with some intention to act on these thoughts.<br>• You are engaging in dangerous behaviors such as spending lots of money or having promiscuous sex.<br>• You are having hallucinations, seeing, or hearing things.<br>• You are having delusions or ideas that do not line up with reality.<br>• You are unable to take care of yourself, such as not getting out of bed, not showering, and not going to work.<br>• If your psychiatrist thinks you should go to the hospital. They may see symptoms of which you are unaware.	**WHY GO TO THE HOSPITAL**<br><br>There are two main reasons to go to the hospital when you have severe symptoms.<br><br>To stay safe.<br><br>Severe symptoms are life threatening. A large percent of individuals with bipolar disorder attempt suicide at some time in their life. Sadly, a significant number actually kill themselves. Bipolar disorder is a treatable condition. There is a way out of the pain. The hospital will keep you safe until you get the right medicine and the right amount of medicine to feel well.<br><br>To get intensive treatment.<br><br>Rapid medication changes can be made only on an inpatient unit where you are under constant medical supervision. Other individual and group treatments also help in a more rapid recovery.	Check out the Team section of the Toolbox to learn even more about using a team effectively.<br><br>Stay well...

Daily Review Feedback Category 26: Staying Well, Team – Hospital (Choice 4.3)

Learn more (P3)	Learn about (P3)	Learn about (P18)	Why (U68)	What (U64)	Foundations (P35)
**1**<br>**RANDOM UNLINKED**	**2**<br>**RANDOM UNLINKED**	**3**<br>**RANDOM UNLINKED**	**4**<br>**RANDOM LINKED**	**5**<br>**RANDOM LINKED**	**6**<br>**RANDOM UNLINKED**
You say you're doing well. That's good.<br><br>Continue on to learn more about staying well.	Being aware, living a healthy lifestyle, coping with symptoms, and having a good team in place will help you stay well.<br><br>Read more about…	Good choice. Most people find it tough to use their team regularly and effectively.<br><br>Learn about working with…	**WHY THE HOSPITAL**<br><br>Most of the time, mild and even moderate symptoms can be treated on an outpatient---basis. In some cases, however, symptoms and impairment can become severe enough to warrant hospitalization. A stay in the hospital can provide a safe and stable environment that promotes recovery. With continuous nursing care available around the clock, an inpatient setting can also be a good choice for patients that require closer monitoring and/or medication changes.<br><br><u>Medication changes</u> can be made much more rapidly and efficiently on an inpatient unit because of the higher level of support available to monitor for and respond to any problems related to these more rapid changes.	**WHAT TO EXPECT**<br><br>• <u>Safety</u> is the primary concern. Everything about an inpatient unit, from the physical layout to the policies and procedures, are focused on preventing any patient from harming himself or anyone else.<br>• The treatment environment is called the <u>milieu</u> and it has both structured and unstructured components. Structured components include group therapy, community meetings, and psychoeducation classes. Unstructured components include interactions between patients, staff, and visitors.<br>• The average <u>length of stay</u> is usually less than one week, but this can vary depending on individual needs.<br>• An attending <u>psychiatrist</u> is assigned to each patient to oversee treatment and prescribe necessary medications. He/she will meet with you to review your progress every day.<br>• Other <u>staff members</u>, such as nurses, social workers, and therapists are also present on the unit to provide care and support to you within their area of expertise.	Review the module "Using Your Team Effectively" in Foundations.<br><br>Stay well…

Daily Review Feedback Category 26: Staying Well, Team – Hospital (Choice 4.3)

Learn more (P4)	Learn about (P4)	Learn about (P19)	Directives (U69)	Directives (U65)	My Team (P36)
**1**<br>**RANDOM UNLINKED**	**2**<br>**RANDOM UNLINKED**	**3**<br>**RANDOM UNLINKED**	**4**<br>**RANDOM LINKED**	**5**<br>**RANDOM LINKED**	**6**<br>**RANDOM UNLINKED**
Looks like you're doing well, which is great.<br><br>Continue to learn more about staying well.	Now is a good time to learn more about staying well. There are four areas on which you can focus.<br><br>Read about...	Having good working relationships with your psychiatrist and supports are important aspects of staying well.<br><br>Identifying a hospital in advance is good in the event that you need to go in.<br><br>Learn more about...	**ADVANCED DIRECTIVES**<br><br>If you want, you can fill out an advanced directive for mental health treatment. Sometimes these are called "Declaration for Mental Health Treatment." You can find these forms on line. Some states have standard forms.<br><br>In a nutshell, you write out instructions about when, where, and how you would (and would not) like to receive treatment when ill.	Directives include:<br><br>• Preferred medications as well as and medications to which you do not consent<br><br>• Physician you wish to determine your capacity for consent if the hospital suggests you are incapacitated<br><br>• Attorney in fact, or who will make decisions for you if you are unable to do so yourself	Read more about doctors , supports, and hospitals:<br><br>www.dbsalliance.org<br><br>Stay well...

Daily Review Feedback Category 26: Staying Well, Team – Hospital (Choice 4.3)

Learn more (P5)	Learn about (P5)	Learn about (P20)	Reach out (U70)	Pros Cons (U66)	My Team (P21)
**1**<br>**RANDOM UNLINKED**	**2**<br>**RANDOM UNLINKED**	**3**<br>**RANDOM UNLINKED**	**4**<br>**RANDOM LINKED**	**5**<br>**RANDOM LINKED**	**6**<br>**RANDOM UNLINKED**
Great to see you're well.<br><br>Continue for strategies to help you stay on track.	There are four key areas to focus on in order to stay well.<br><br>Read about…	The ability to use your team effectively is related to fewer illness relapses. Don't be too proud or shy or otherwise reluctant. Reach out!<br><br>Read about…	**REACH OUT**<br><br>Generally speaking, no one wants to be in the hospital. However, a short stay can<br><br>(1) Keep you safe when you're having severe symptoms.<br><br>(2) Result in more rapid recovery than if you try to get well as an outpatient.	When you are well consider…<br><br>What are the advantages of a short hospital stay if you get severely ill?<br><br>What are the disadvantages of a short hospital stay if you get severely ill?<br><br>Write out your thoughts and keep it somewhere for future reference.	Did you learn something new? Anything you want to share with your psychiatrist or supports?<br><br>Stay well…

WEEKLY CHECK-IN

TABLE OF CONTENTS

1. Patient Health Questionnaire 8
2. Altman Self-Rating Mania Scale
3. Early Warning Signs CheckList - Mania
4. Early Warning Signs CheckList - Depression

PATIENT HEALTH QUESTIONNAIRE 8

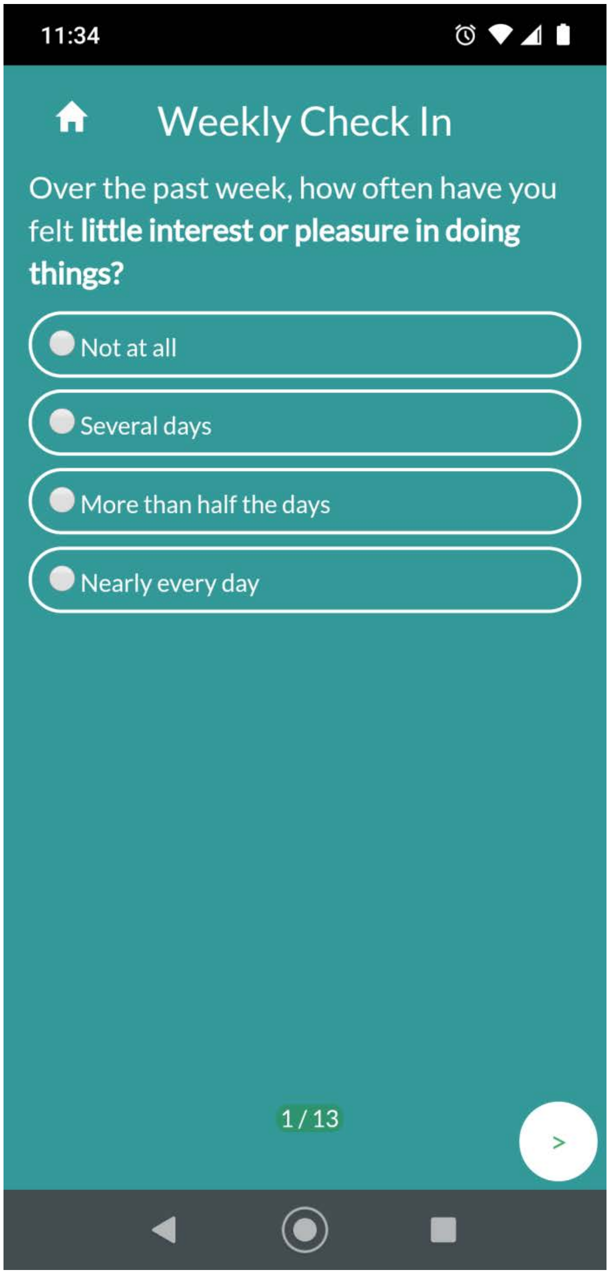

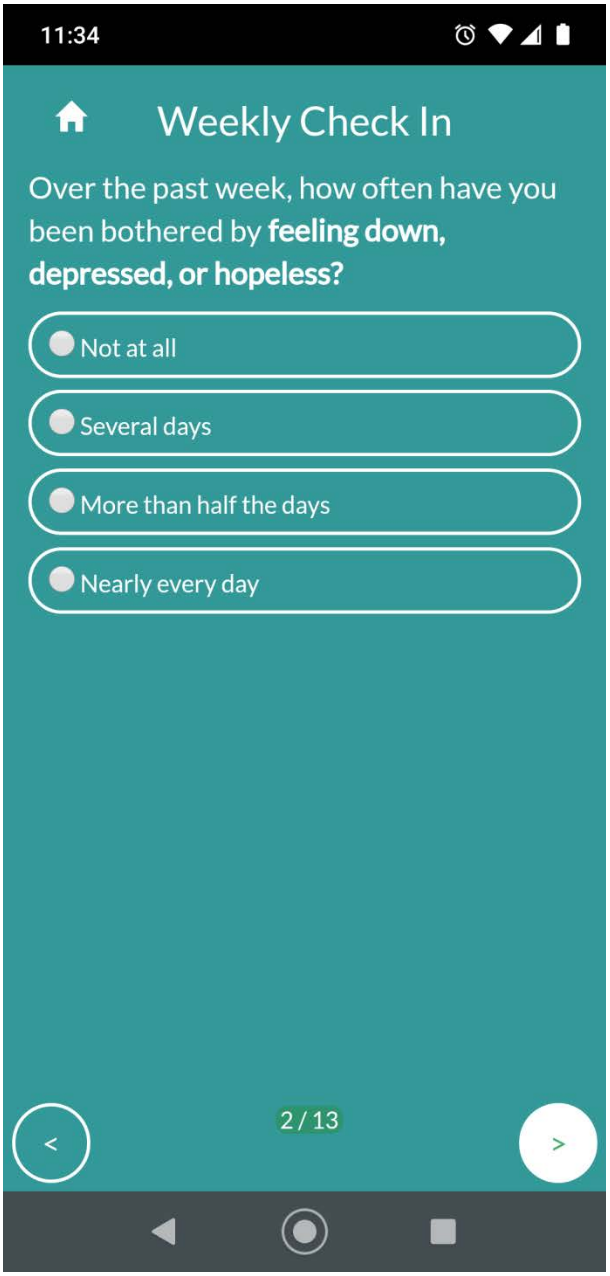

PATIENT HEALTH QUESTIONNAIRE 8

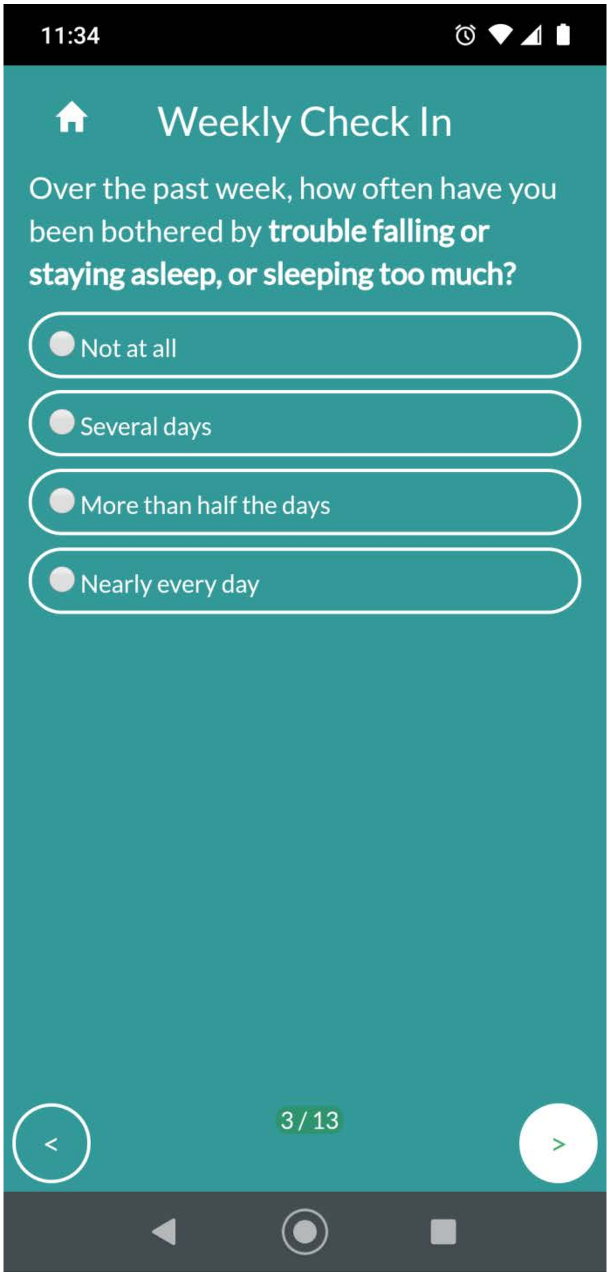

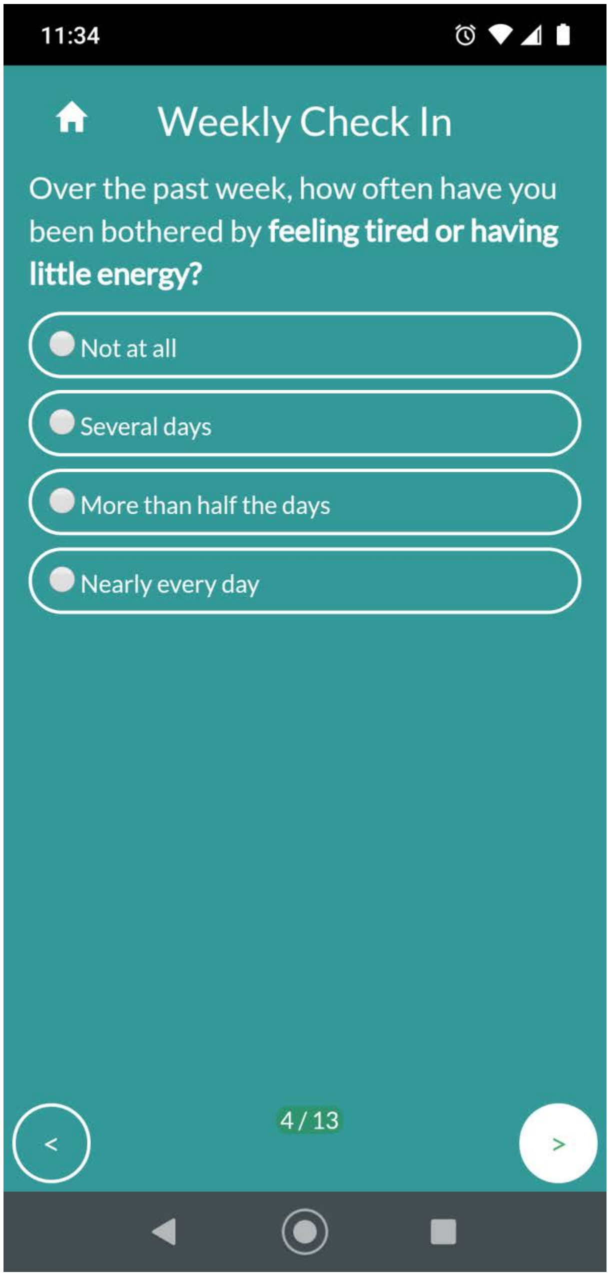

PATIENT HEALTH QUESTIONNAIRE 8

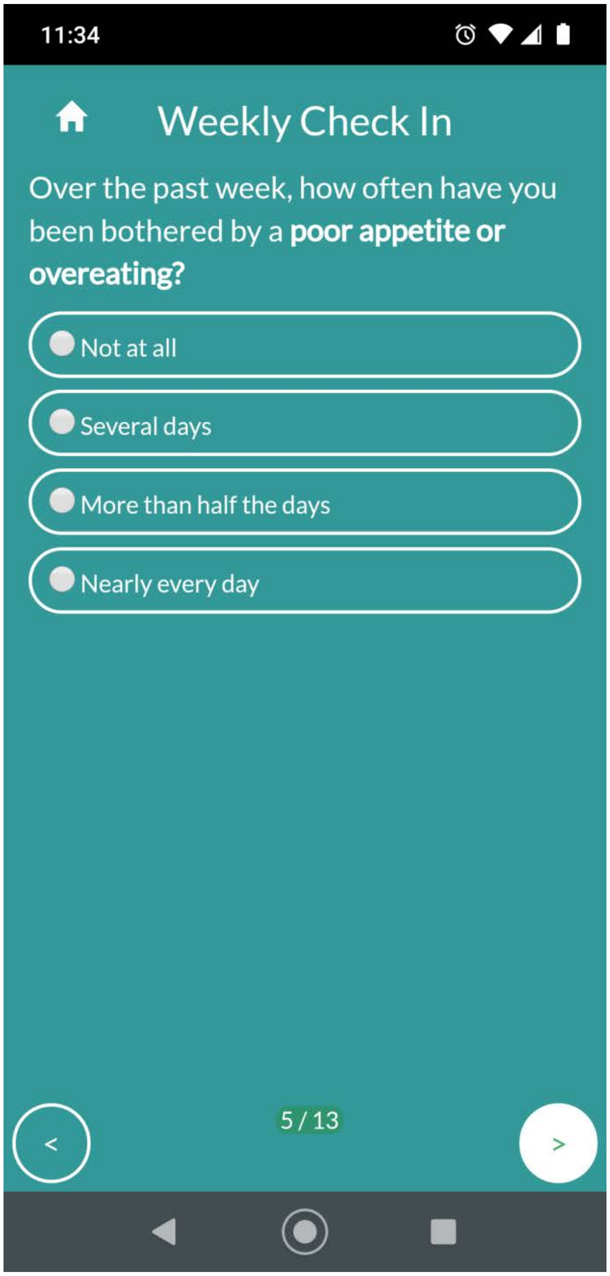

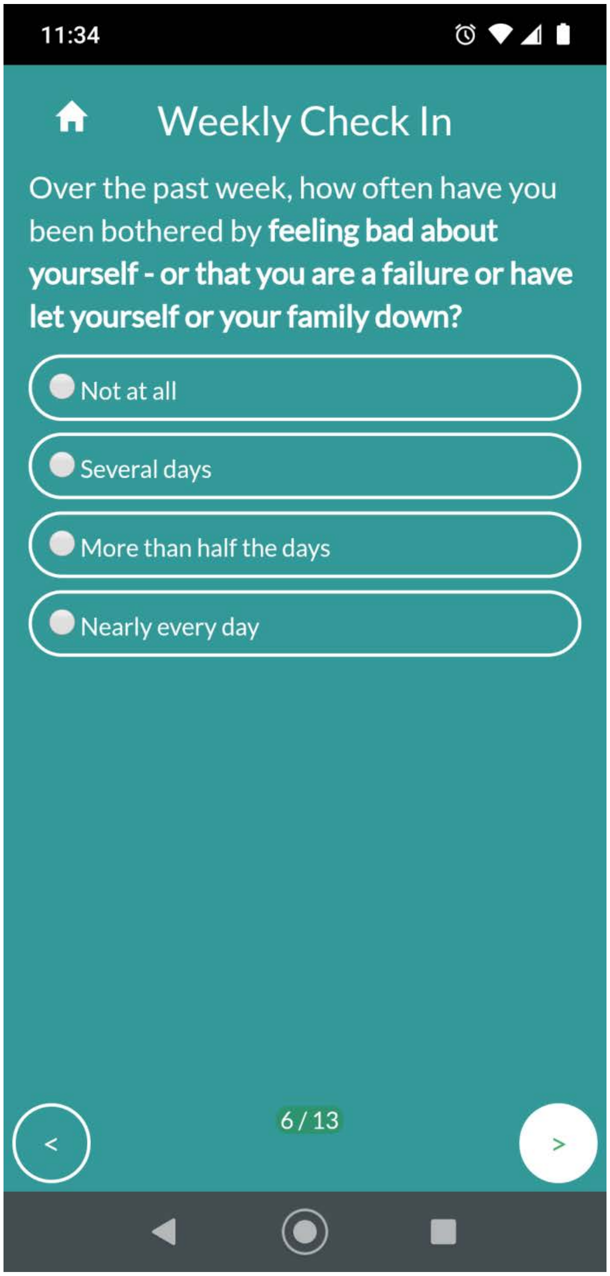

Patient Health Questionnaire 8

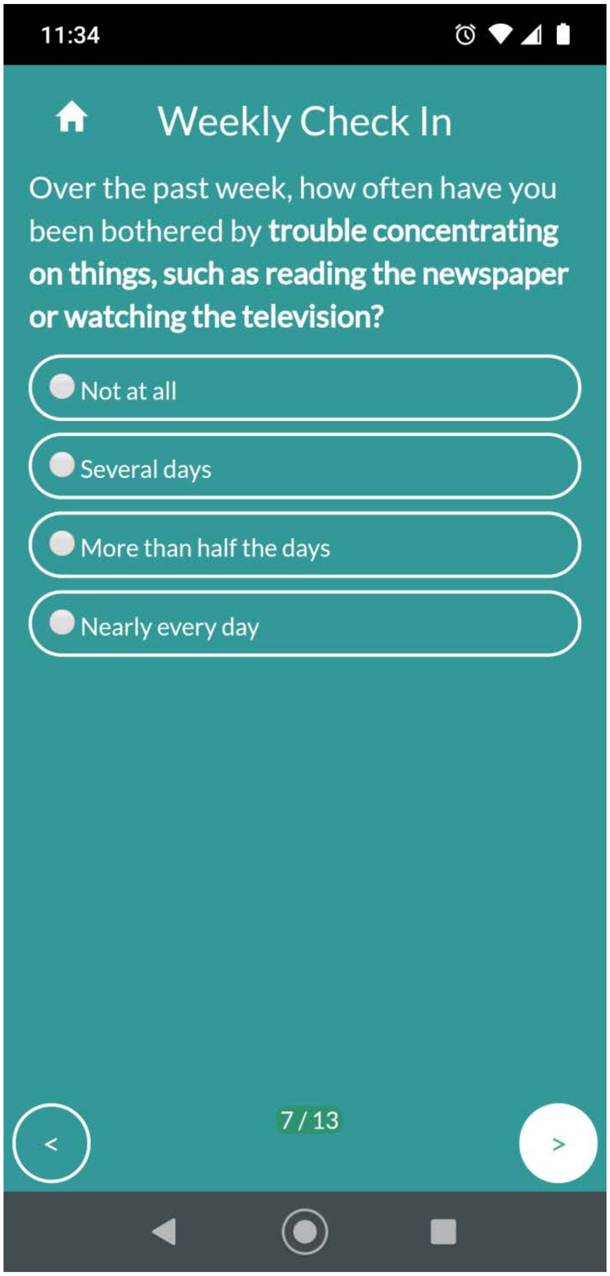

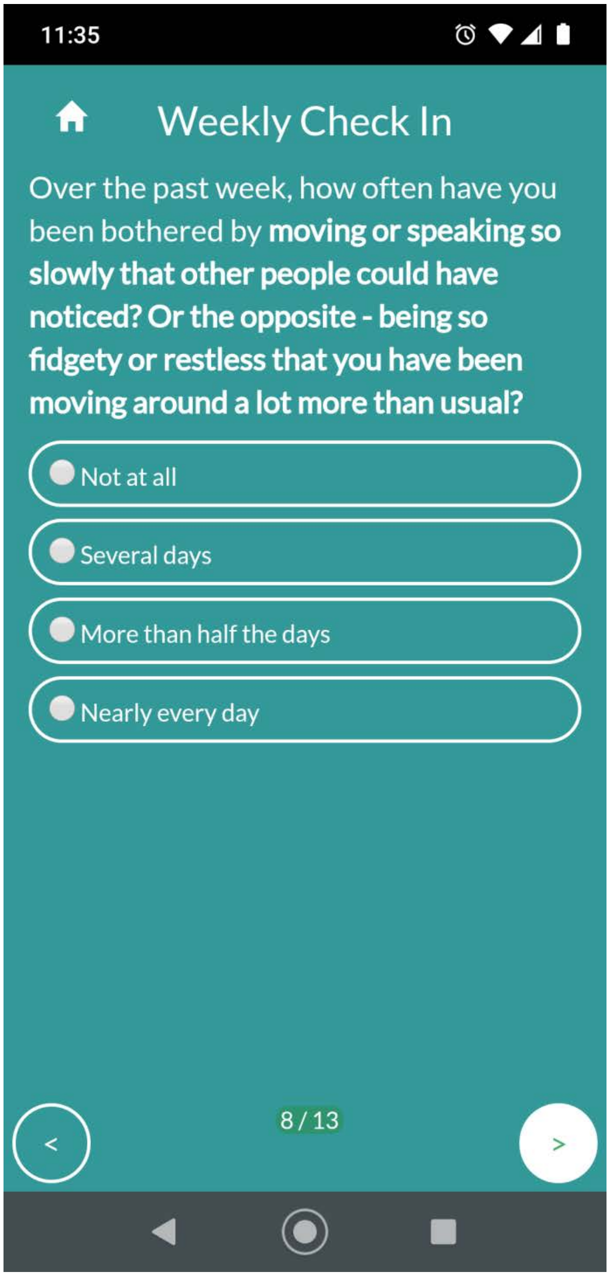

ALTMAN SELF-RATING MANIA

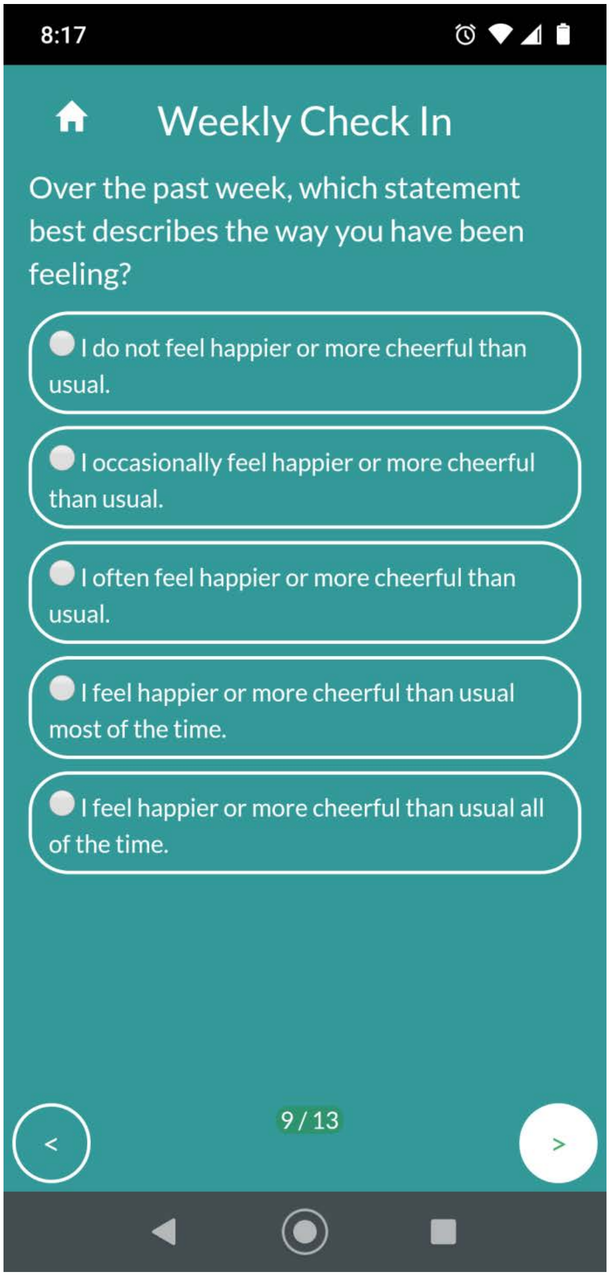

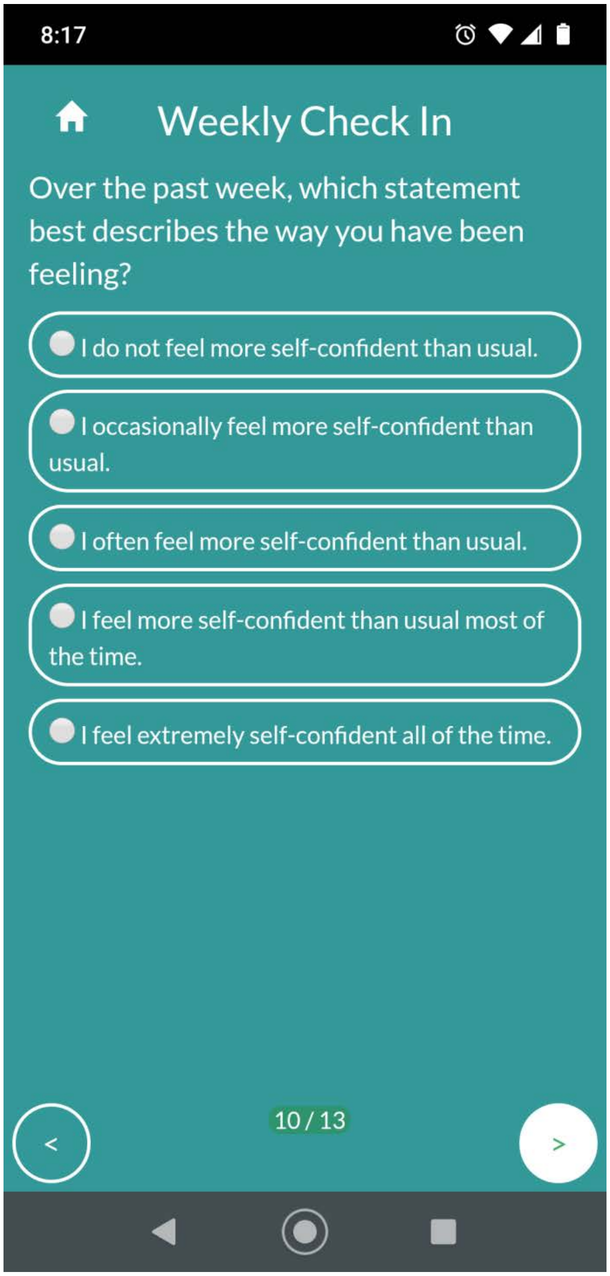

ALTMAN SELF-RATING MANIA

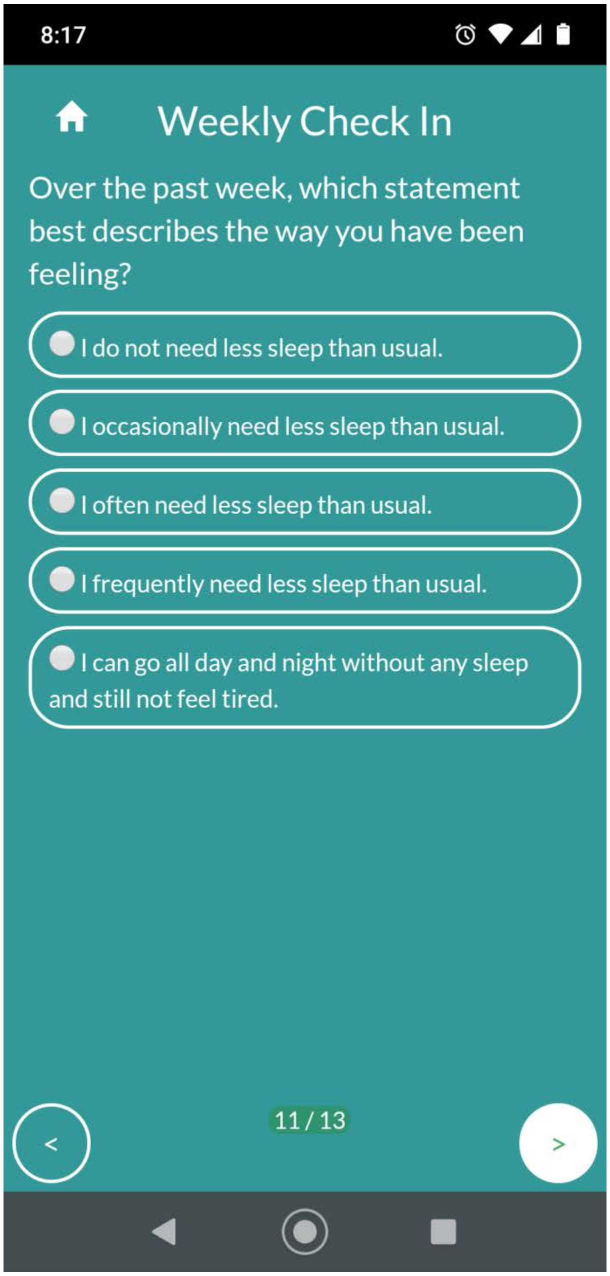

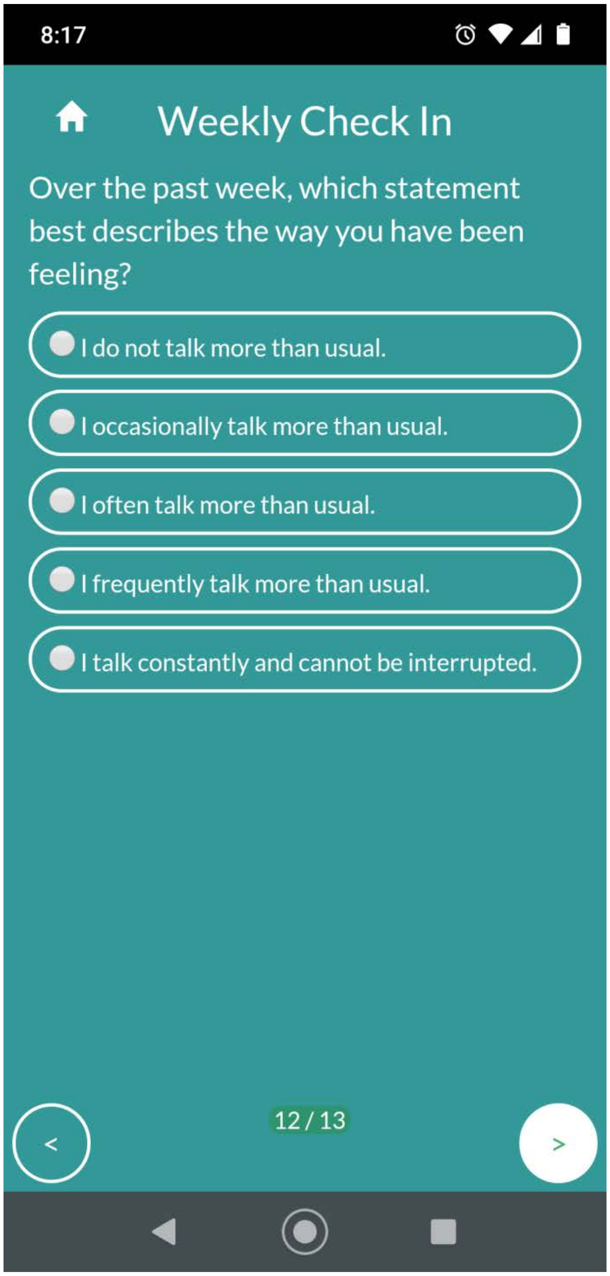

ALTMAN SELF-RATING MANIA

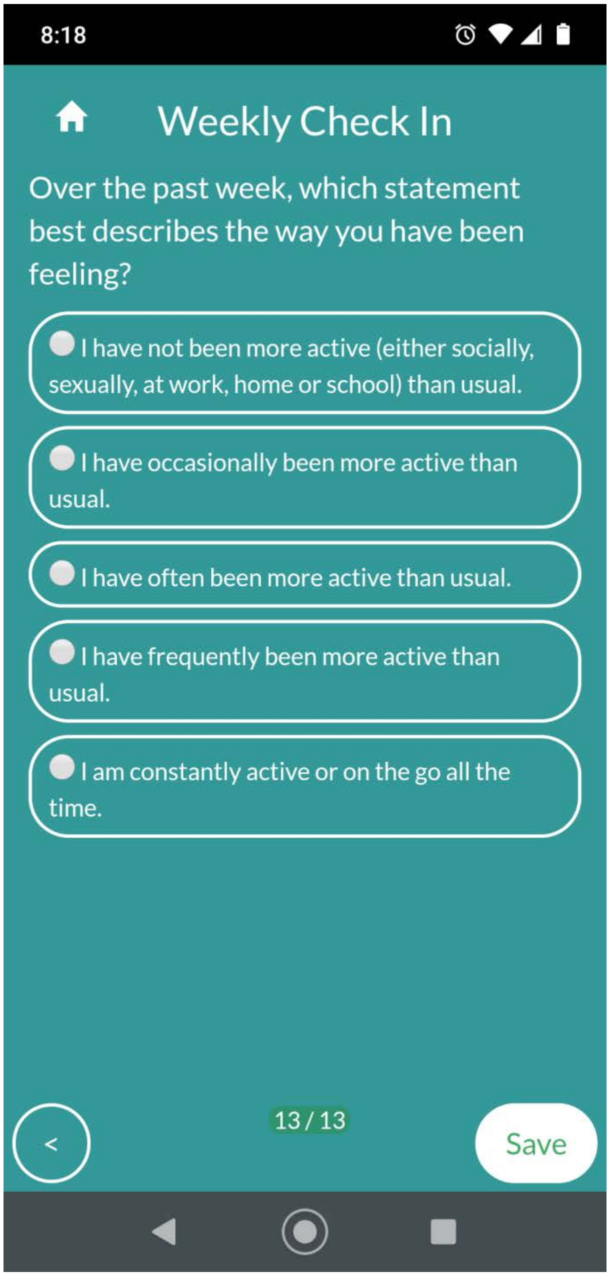

EARLY WARNING SIGNS CHECKLIST

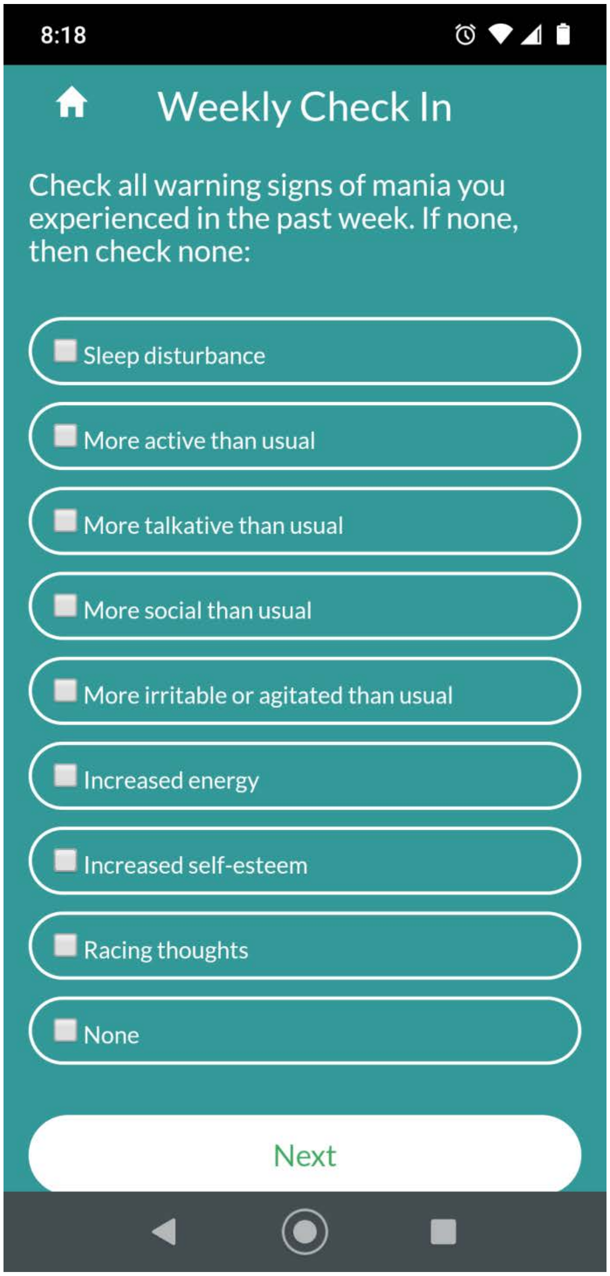

EARLY WARNING SIGNS CHECKLIST

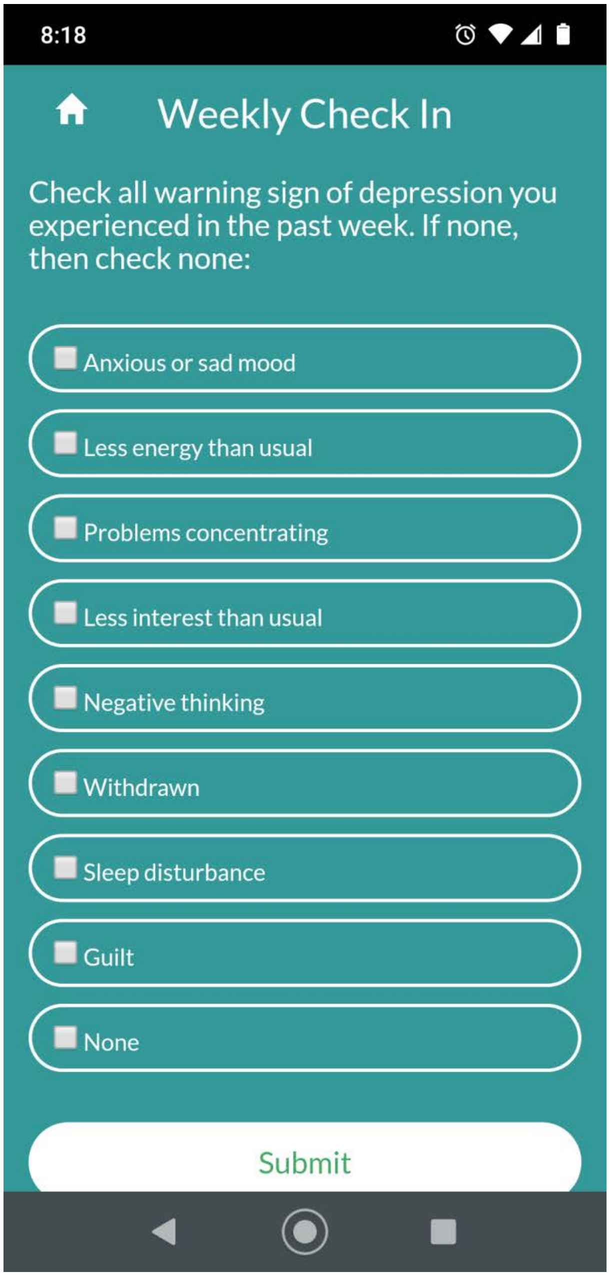

Settings

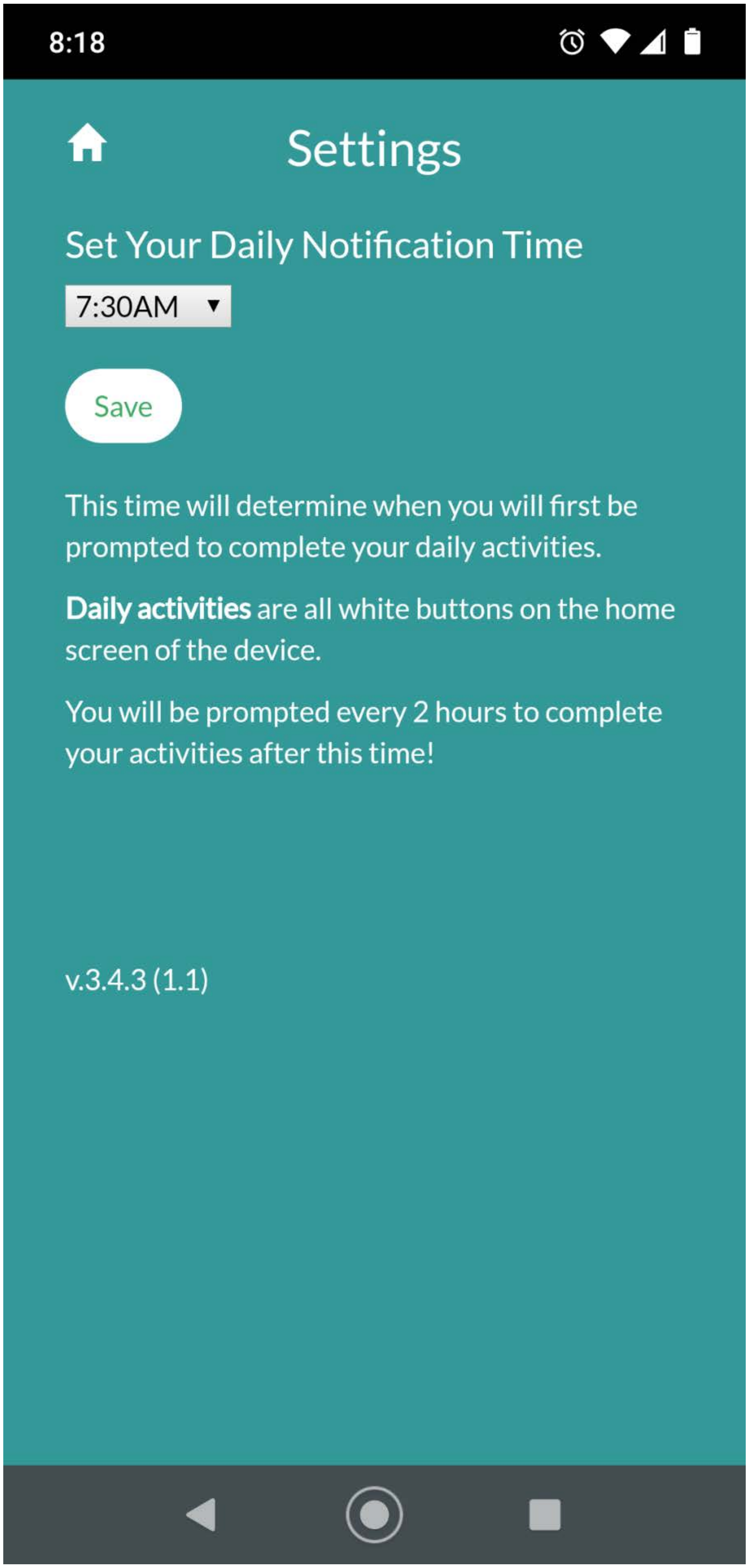
8:18
Settings
Set Your Daily Notification Time
7:30AM
Save
This time will determine when you will first be prompted to complete your daily activities.
Daily activities are all white buttons on the home screen of the device.
You will be prompted every 2 hours to complete your activities after this time!
v.3.4.3 (1.1)

INSTRUCTIONS

TABLE OF CONTENTS

1. **INTRODUCTION**
   - 1.1. Overview
   - 1.2. Timetable
2. **SETTINGS**
3. **TOOLBOX**
4. **COACH**
5. **PSYCHIATRIST**
6. **FOUNDATIONS**
7. **DAILY CHECK IN**
   - 7.1. Daily Check In
   - 7.2. Medications
   - 7.3. Sleep
   - 7.4. Routine
   - 7.5. Wellness Scale
8. **WEEKLY CHECK IN**
   - 8.1. Overview
   - 8.2. Symptoms of depression
   - 8.3. Symptoms of mania
   - 8.4. Early warning signs of depression
   - 8.5. Early warning signs of mania
9. **DAILY REVIEW**
10. **WELLNESS PLAN**
    - 10.1. Overview
    - 10.2. My Resources
    - 10.3. Reduce Risk
    - 10.4. Awareness & Action
11. **CHARTS**

Introduction

Overview

The goal of *LiveWell* is to help people with bipolar disorder better manage their symptoms and function at their best. The program will help you develop knowledge and skills. It will teach you self-management strategies and help you take your medications consistently.

The *LiveWell* application has 5 main components:

- Foundations
- Toolbox
- Check ins
- Daily Review
- Wellness Plan

Each of these components will be explained in more detail in the sections that follow. A coach will also be available to support you in learning to use the application.

It's important to remember that *LiveWell* is a self-management tool designed to supplement work with your psychiatrist. *LiveWell* will provide you with many resources, but it will take work every day on your part. The good news is that it gets easier with time.

Introduction

Timetable

LiveWell is a 4-month program. During Month 1, you will be asked to read the Foundation lessons and practice using all aspects of this application. Your *LiveWell* coach will schedule short weekly phone calls to check in on how things are going and to review the Foundation lessons with you.

At the end of the 1st month, your coach will schedule a longer phone call to help you personalize different sections of the application.

For the remainder of the program, your coach will continue to check in, but not as frequently. However, if you have any problems, your coach is available to help and can be reached by phone or email. You and your coach will have a scheduled call during your last week of the program as well.

A study staff member will also complete a phone survey with you each month. At the end of the study, you may come in for an interview about how the *LiveWell* program worked for you. Any study equipment you have will be returned then.

Settings

Each day a notification asking if you can complete your *LiveWell* activities will appear at the top of your phone screen. If it's a good time for you, press the notification. This will take you to the application and you can complete some or all of the activities. If it isn't a good time for you, ignore the notification. The application will then prompt you again later in the day.

The *LiveWell* activities include the Daily Check In and Daily Review as well as the Weekly Check In. The activities to be completed will show up as white buttons on the home page of the application. Once you complete the activities their buttons will no longer be shaded white or will disappear from the home screen.

To set the time you want to receive your first notification, push the Settings icon on the home page of the application. Set a time that works best for you to receive your first reminder of the day.

Toolbox

The Toolbox button is on the homepage.

The Toolbox has five parts:

- Making Changes
- Assessments
- Lifestyle
- Coping
- Team

Making Changes provides basic information and exercises for getting motivated, setting goals, and making plans to achieve your goals. This section can help you with achieving important goals of the *LiveWell* program like taking medications daily and getting the right amount of sleep.

Assessments provides a series of surveys to help you identify what areas you might want to work on to stay well.

Lifestyles, Coping, and **Team** all have information about skills and exercises to help you practice building these skills. As you work your way through the various skills, you can save those you find most helpful to My Skills in your Wellness Plan. To save a skill, press the Save to My Skills button, which can be found at the end of each skill.

Throughout your use of the *LIveWell* application, we encourage you to use the Toolbox regularly to help you get motivated, make plans, and to learn and practice skills to help you stay well.

Coach

The role of your *LiveWell* coach is to support you in using the application. Remember that your coach will not provide therapy but will help you with the self-management strategies. You and your coach will have six scheduled calls to help you get the most out of using the *LiveWell* application.

In addition to these scheduled calls, if you have problems or questions about using the application, you can contact your coach by phone or email at:

(312) 503-1886

LiveWellCoach@Northwestern.edu

Your coach will get back to you within 1 to 2 days on Monday to Friday between 9:00 a.m. and 5:00 p.m.

If you are having an urgent psychiatric problem, contact your psychiatrist immediately. If you are feeling suicidal, call 911 or go to the nearest emergency room.

Psychiatrist

The *LiveWell* program is set up to share important information with your psychiatrist so he or she can provide you with the best treatment. If you want, your psychiatrist will be able to review a report that provides a weekly overview of how you are doing. Your psychiatrist will get information about your:

- Medication use
- Sleep duration
- Routine- bedtime and risetime
- Wellness ratings
- Early warning signs
- Depression and mania scores

All information shared with your psychiatrist is **<u>confidential</u>.** No one else will have access to your information.

Foundations

The first 8 Foundations lessons cover the nuts and bolts. They provide key information about bipolar disorder and describe important strategies for managing symptoms.

To access these lessons, click on the Foundations button on the main page of the application. This will open the Foundations page. You will see 9 buttons, one for each lesson.

Each lesson takes about 5 to 10 minutes to read. We ask that you try to read the first 8 lessons during the 1st month. Your coach will review 2 per week with you. In the last week of the program, we'll ask that you read a final wrapping up lesson and discuss with your coach.

Daily Check In

Daily Check In

The Daily Check In allows you to track:

- Your medication use
- Sleep duration
- Routine – bedtime and risetime
- Wellness

Getting in the habit of completing Daily Check Ins will help you recognize warning signs and symptoms of illness and allow you to act early to prevent full-blown episodes from occurring. This can also help you tune in to changes in your mood, thoughts, energy levels, and behaviors.

Having a record of how you have been doing can also help your mental health providers have a better understanding of what is going on with you. They can use this information to make treatment decisions.

Daily Check In

Medications

Record your daily medication use using the buttons under Medications in your Daily Check In. If you took all your psychiatric medications as prescribed the day before, push the All button. If you only took some, then push Some. If you did not take any of your medications the day before, push None.

Only report whether you have taken the medications prescribed by your psychiatrist for bipolar disorder. You should only track medications you are prescribed to take every day.

Some medications are prescribed for only occasional use. For instance, some medications for sleep are only used when you cannot fall asleep. If you have any questions about which medications to track, please talk with your coach.

To review an up-to-date list of your medications, visit My Medications in your Wellness Plan. If your medications change at any point during the study, inform your study coach. Call or email as soon as you can. Your coach's contact information can be found under My Team in your Wellness Plan. It is also listed under Coach in the Instructions.

Daily Check In

Sleep

Getting the right amount of sleep is important for reducing symptoms and episodes of mania and depression. Changes in how many hours you are sleeping a day can also be an early sign that you are starting to develop mania or depression. Because of these things, keeping track of your sleep can be very useful.

The Daily Check In allows you to track the number of hours you sleep each night. To record the number of hours you slept, tap the dropdown box under SLEEP. The number of hours you slept should be your best estimate of the total duration during which you were asleep. It may not correspond exactly to when you first went to bed for the night or got up for the day.

Daily Check In

Routine

Having a regular routine for when you go to bed and when you get up for the day is important for reducing symptoms and episodes of mania and depression. Changes in the pattern of sleep can also be an early sign that you are starting to develop mania or depression. Because of these things, keeping track of your routine can be very useful.

The Daily Check In allows you to track your sleep and wake routine. To record the time you went to bed the night before with the intention of going to sleep, tap the text box under the heading Went To Bed. A dropdown list will appear on your screen. Select the time that best corresponds with the time at which you tried to go to sleep. Keep in mind that this might be different from the time when you actually fell asleep.

To record the time you got up with the intention of starting your day, press the text box under the heading Got Up. A dropdown list will appear on your screen. Select the time that best corresponds with the time at which you got up to start your day.

Daily Check In

Wellness Scale

As part of the Daily Check In, you will also rate how well you are doing each day. To do this, select the button on the wellness rating scale that best matches how you are doing. Once you push the button, it will turn white to show it has been selected.

The wellness scale goes from -4 for seriously depressed to +4 for seriously manic. Changes in mood, thinking, behaviors, sleep, and energy levels are a part of life. These changes can be:

- Normal reactions to life events (-1 to +1)
- Warning signs or symptoms before an episode (-2 or +2)
- Some ongoing symptoms (-2 or +2)
- Multiple continuing symptoms during an episode (-3 or +3)
- Dangerous symptoms (-4 or +4)

Look at each number on the wellness scale. For each level think about how you would describe:

- Your mood
- The kind of thoughts you have
- How fast or slow your thinking is
- Your behaviors
- Your sleep

These descriptions are called anchors. They fix the points on your scale.

As part of the enrollment process, your *LiveWell* coach reviewed the wellness ratings with you and helped you create personal anchors for each point on the scale. The anchors you came up with during this training session are available for you to review in the Awareness & Action section of your Wellness Plan. There you will also find definitions for each of the wellness rating categories.

During week 4 of the program, you will have an opportunity to review your anchors again with your coach and make any necessary changes. If you need to make changes to your anchors at other times during your participation, let your coach know. He or she will be happy to assist you in making any additional changes.

Weekly Check In

Overview

Once a week, you will be asked to complete 4 brief questionnaires that measure symptoms and early warning signs of depression and mania. These questionnaires will come up once a week on Sundays. Together they will take about 5-10 minutes to complete.

Weekly Check Ins will help you track how you are doing over time. Looking at this data will also be very helpful for your mental health providers because the questionnaires are standard measures of depression and mania. Your responses can be used to help guide decisions about your treatment.

Weekly Check In

Symptoms of Depression

The first survey asks about symptoms of depression. Please read each question carefully. Mark the answer that best describes how often you have been bothered by that problem in the past week.

Weekly Check In

Symptoms of Mania

The second survey asks about symptoms of mania. There are 5 groups of statements in this survey. For each group, you should choose the statement in each that best describes the way you have been feeling for the past week.

Please note when used in this survey:

- "Occasionally" means once or twice
- "Often" means several times or more
- "Frequently" means most of the time

Weekly Check In

Early Warning Signs of Depression

The third survey asks about early warning signs of depression. Check off any early warning signs of depression you experienced in the past week. If you did not experience any early warning signs of depression, check none.

Weekly Check In

Early Warning Signs of Mania

The fourth survey asks about early warning signs of mania. Check off any early warning signs of mania you experienced in the past week. If you did not experience any early warning signs of mania, check none.

Daily Review

The Daily Review provides you with timely feedback on how you are doing. It will help you maintain awareness and identify opportunities for action when needed. On the initial feedback page, you will receive summary information describing how successful you were in achieving your wellness targets.

The Daily Review has 4 scales:

- Medications
- Sleep
- Routine
- Wellness

The scales provide a summary over 7 days of:

- How consistently you have been taking your medications
- How often your sleep duration is in your selected window
- How regular your bedtime and risetime routine is
- How often your wellness rating is in the -1 to +1 range

You can click on the shaded bars to see what your targets are for these 4 areas. Knowing your specific targets will help you better understand how the application is providing this feedback.

By pressing the Continue button on the first page, you will receive additional feedback specific to how you are doing. If you are doing well, the Daily Review will provide you with tips for staying well. If you appear to be having problems, it will provide you with tips for getting back on track.

Wellness Plan

Overview

The Awareness and Action modules in Foundations will help you create your Wellness Plan. To review your plan, push the Wellness Plan button on the main page of the *LiveWell* application.

The wellness plan has 3 sections:

- My Resources
- Reduce Risk
- Awareness & Action

Press the tabs at the top of the Wellness Plan to navigate between the different sections of your plan.

Wellness Plan

My Resources

The My Resources page has four buttons.

- Press **My Medications** to see a list of your current medications. If your medications change during the study, contact your coach to let him or her know. He or she can update your medication list for you.

- Press **My Team** to view a list of your supports. This list includes your psychiatrist. It can also include other people who support you in staying well, such as therapists, family members, and friends.

- Press **My Skills** to review any skills you saved from the general catalog of *LiveWell* skills. You can use these to manage stressors and symptoms. Practicing skills is the key to making them really useful for you.

- Press **My Charts** to review your data. This can help you see patterns that may give you new insights into how to stay well.

Wellness Plan

Reduce Risk

Live a healthy lifestyle to reduce your risk of symptoms. Learn more about reducing risk by looking at the Lifestyle Skills section of Foundations.

The Reduce Risk section of the Wellness Plan has a list of your ideas for a healthy lifestyle. These are broken down into the following sections:

- **S**leep
- **M**edications
- **A**ttend
- **R**outine
- **T**ranquil
- **S**ocial

For the 1st month of the program, your plan for reducing risk will include standard recommendations. During week 4, you will have the opportunity to work with your coach to personalize your plan. If you want to make additional changes later on, you can always contact your coach by phone or email. He or she can update your lifestyle plan.

Wellness Plan

Awareness & Action

This section of the wellness plan has 2 parts.

<u>Awareness</u>. Being aware involves knowing how you are doing. It means looking out for stressors, early warning signs, and symptoms. You will use this information to rate your wellness each day when completing your Daily Check In.

You can press the Definitions button to see an explanation for each of the wellness rating categories. You can press the Anchors button to review the personal descriptors you selected to fix each point on the wellness rating scale.

<u>Action.</u> Taking action means following a plan. You can press the Action button to see your personalized plan for what you will do depending on your wellness rating.

Your action plan has a list of ideas about what to do when:

- Balanced - normal reactions to life events (-1 to +1)
- Slightly down or up - warning signs or symptoms before an episode (-2 or +2)
- Slightly down or up - ongoing symptoms after an episode (-2 or +2)
- Moderately down or up - multiple continuing symptoms during an episode (-3 or +3),
- Severely down or up - dangerous symptoms (-4 or +4).

As part of the enrollment process, your *LiveWell* coach helped you create personalized anchors. During week 4, you will have the opportunity to work with your coach to develop a personalized plan. If you need to make additional changes to your action plan at other times, let your coach know. He or she will be happy to assist you.

Charts

You can see a summary over the last week of your:

- Medication use
- Sleep duration
- Routine- bedtime and risetime
- Wellness Ratings

Charts may help you see patterns that identify your own specific signs of doing well or having problems. By being aware sooner, you can take action early. This can help you avoid full-blown mania or depression.

You can access My Charts by going to the My Resources section of your Wellness Plan.
