# Supplementary material for: A Smartphone-Based Self-management Intervention for Individuals With Bipolar Disorder (LiveWell): Empirical and Theoretical Framework, Intervention Design, and Study Protocol for a Randomized Controlled Trial
Source: JMIR Res Protoc. 2022 Feb 21;11(2):e30710. doi: 10.2196/30710 (PMC8902672; doi:10.2196/30710)
Supplement: Multimedia Appendix 3 [file resprot_v11i2e30710_app3.pdf]

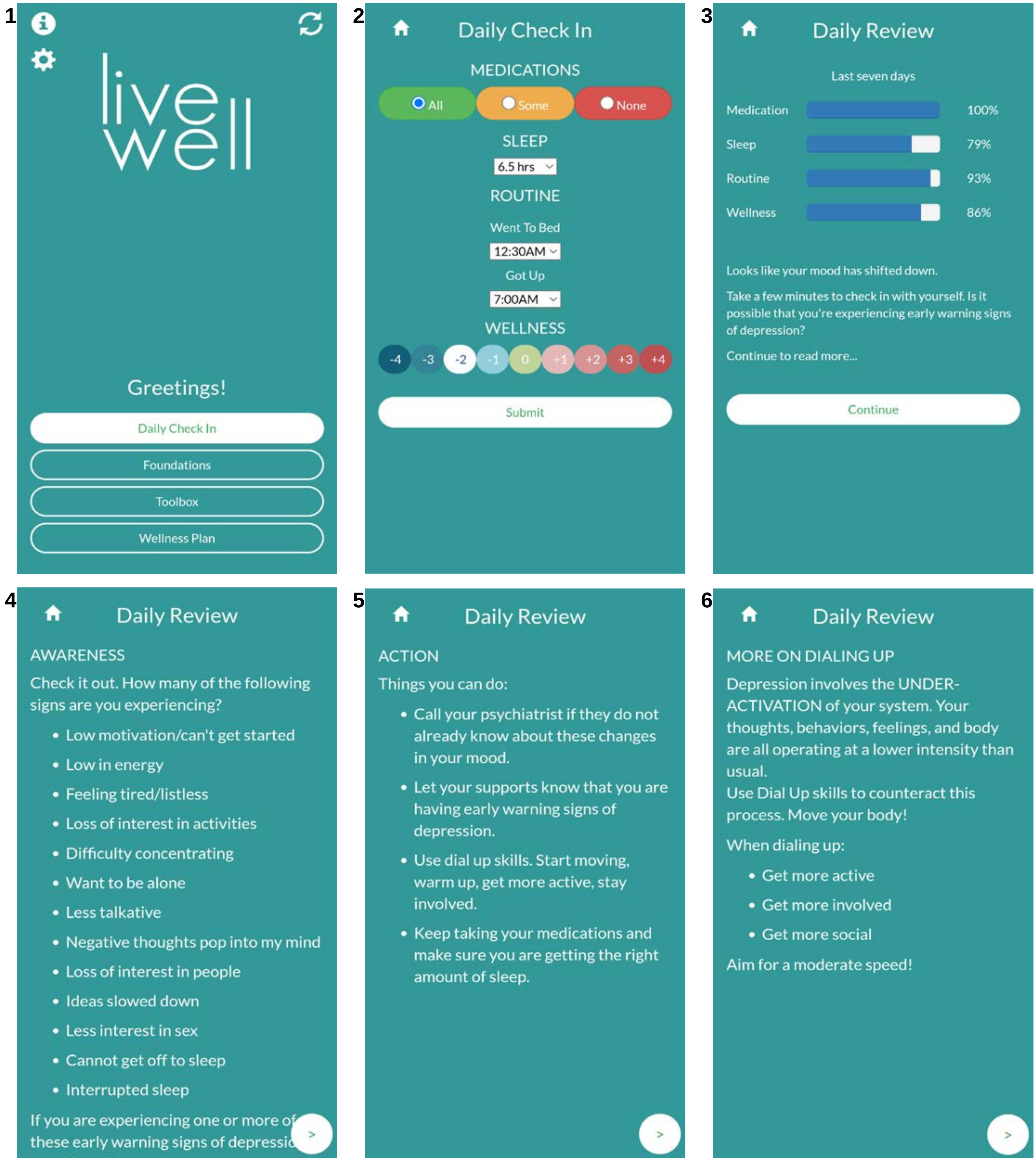

LiveWell Use Case Scenario: User opens app and the home page is displayed with Daily Check In highlighted indicating task to be completed (**1**). User completes Daily Check In with a wellness rating of -2 indicating possible early warning signs of depression (**2**). After user submits Daily Check In data, Daily Review feedback page displays summary of last 7 check ins via bar graphs and expert system identifies possible shift in mood down as priority (**3**). User continues through Daily Review and receives information about awareness (**4**), action (**5**), dialing up (**6**), and increasing social contact (**7**).

7

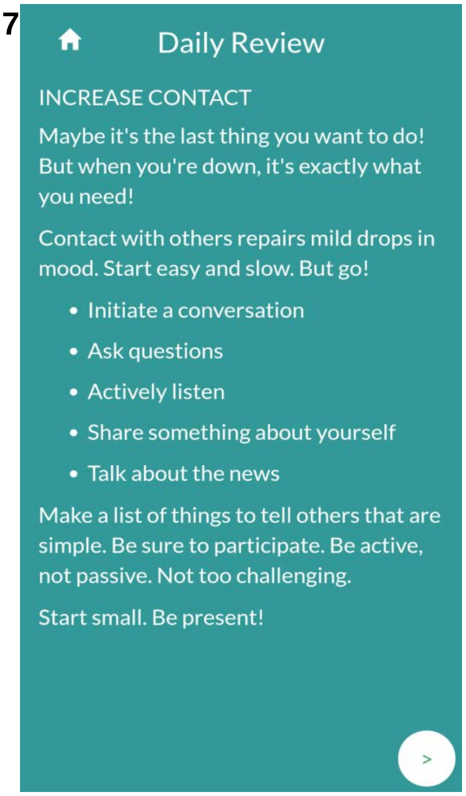

8

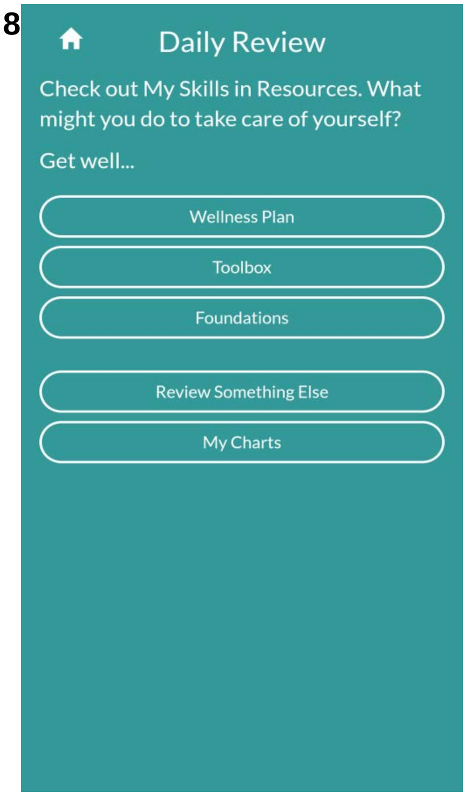

9

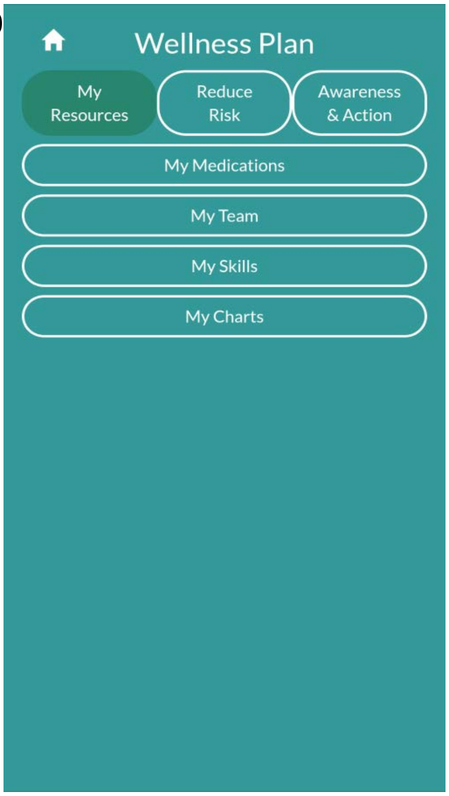

10

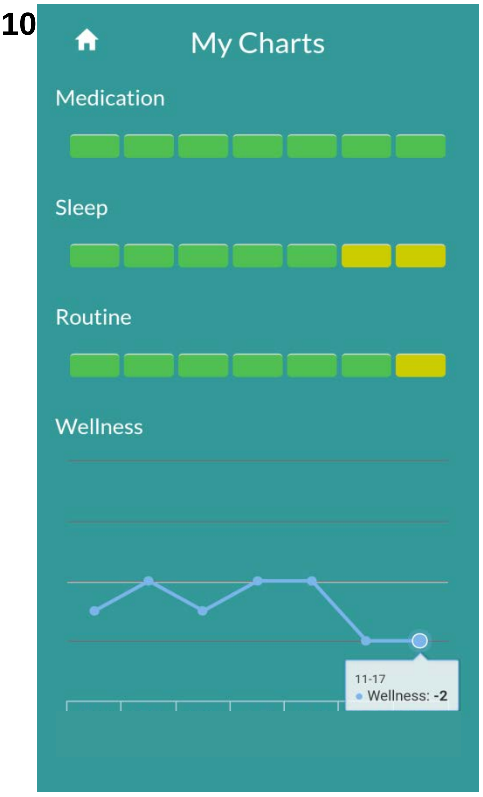

11

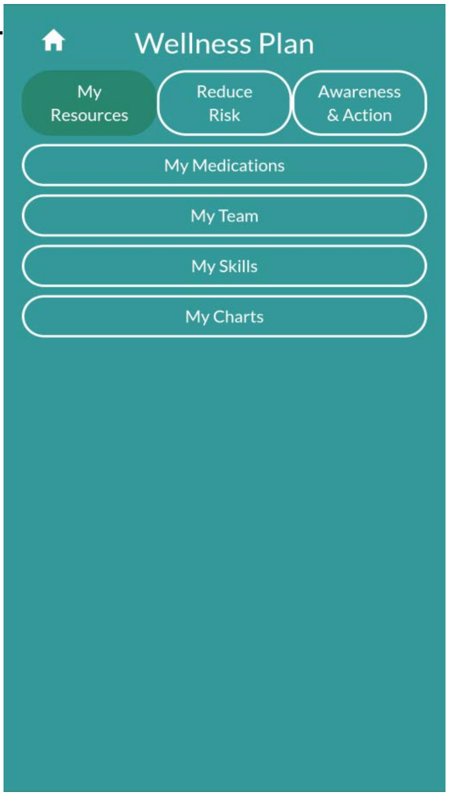

12

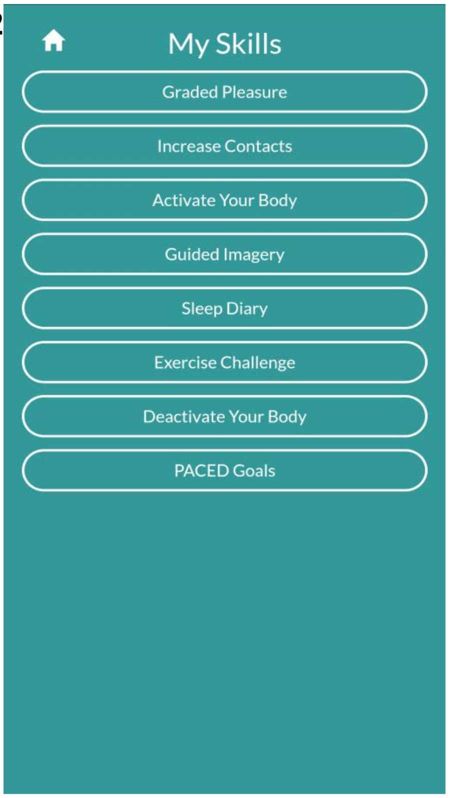

LiveWell Use Case Scenario: User continues to the last page of the Daily Review which suggests user check My Skills in Resources in the Wellness Plan (**8**). User goes to Wellness Plan which defaults to My Resources (**9**). User selects My Charts. My Chart summary is displayed. User clicks on last wellness rating and wellness rating value and date are displayed (**10**). User returns to the Wellness Plan (**11**) and selects My Skills. My Skills page is displayed showing techniques which user has added to Wellness Plan from Toolbox (**12**).

13
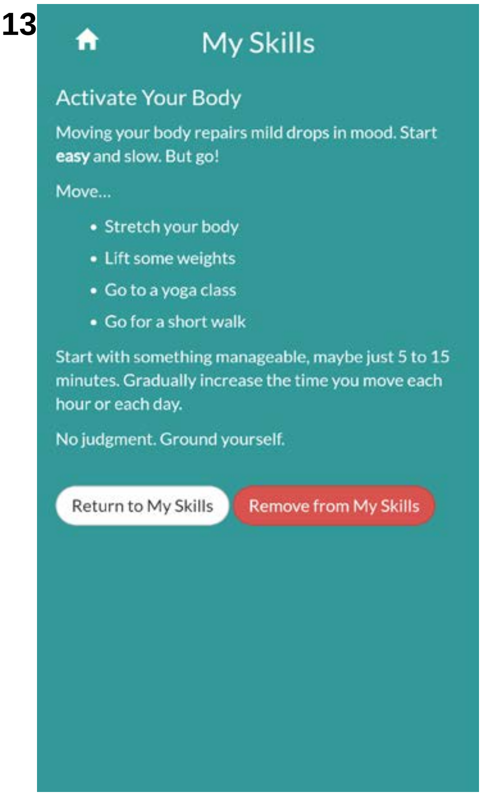

14
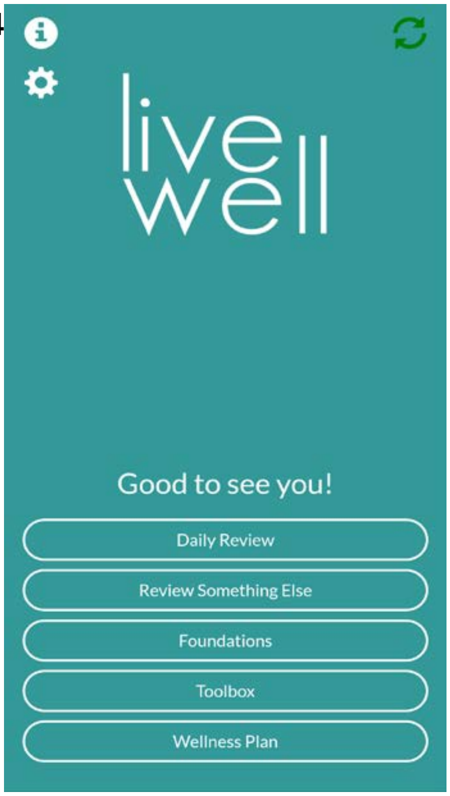

15
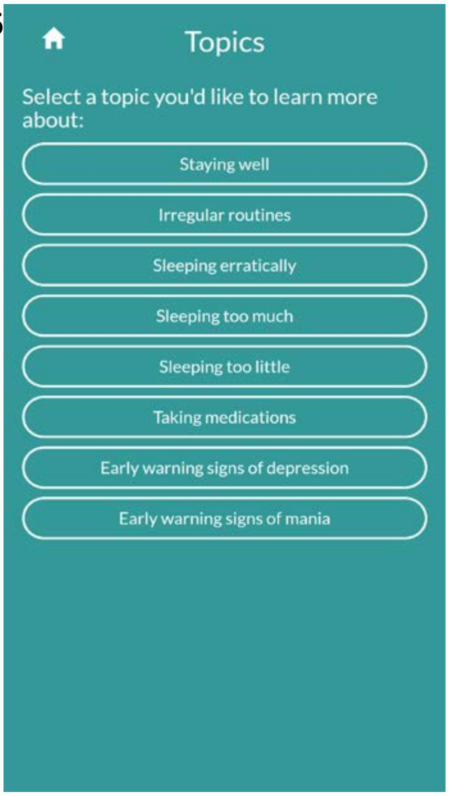

16
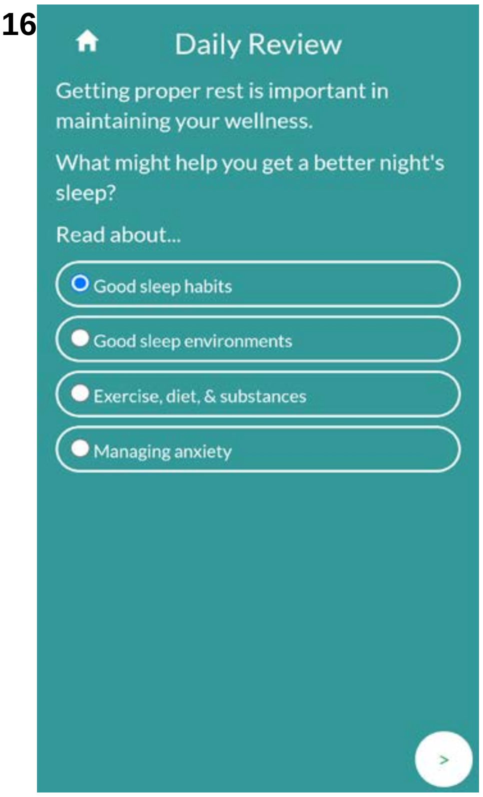

17
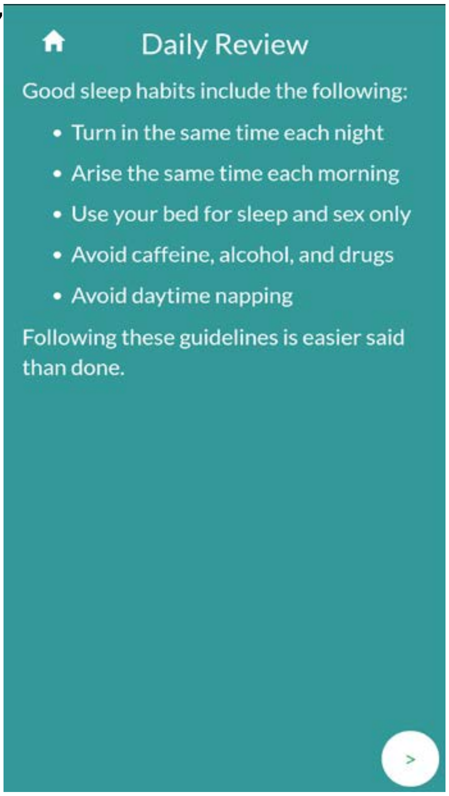

18
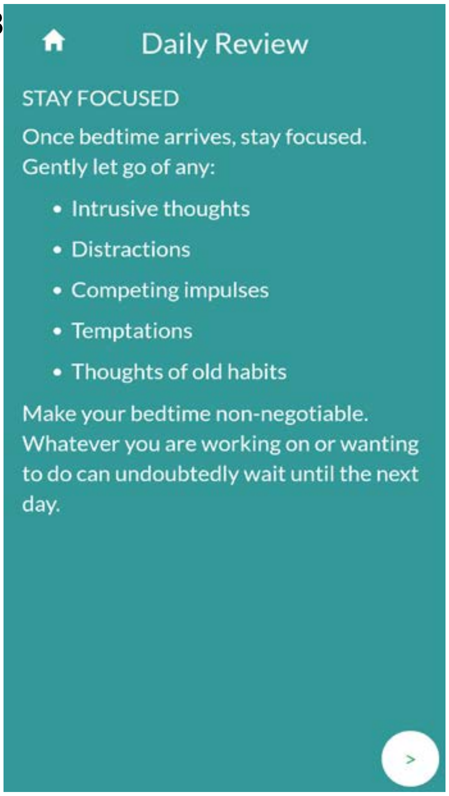

LiveWell Use Case Scenario: User selects Activate Your Body from My Skills (**13**). User returns to app home page which no longer displays Daily Check In as this has been completed for this day. However, home page now displays Daily Review which only becomes available on the home page after the Daily Check In has been completed. Home page also displays Review Something Else which only becomes available on the home page after the Daily Review has been completed (**14**). User selects Review Something Else and a subset of Daily Review topics are displayed (**15**). User selects Sleeping too little and Daily Review content on this topic is displayed with user selecting Good sleep habits (**16**). User continues in Daily Review (**17-19**).

19

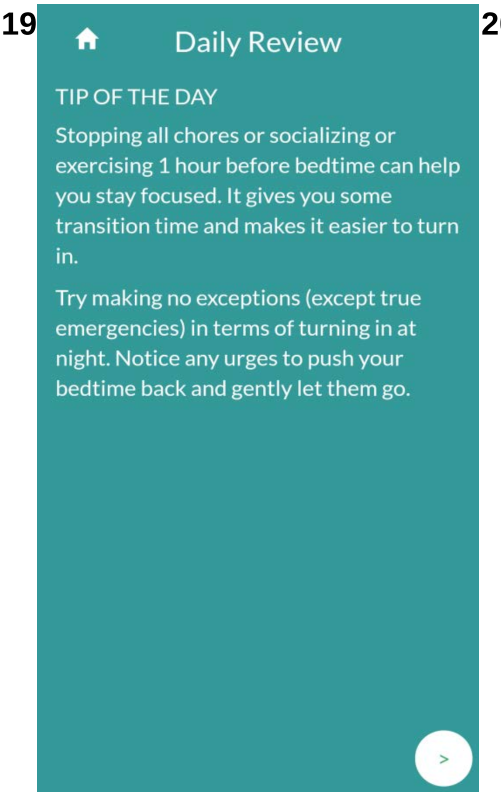

20

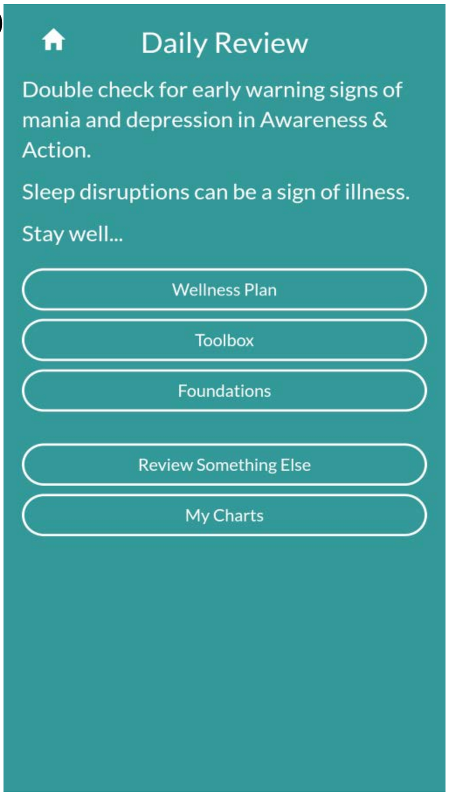

21

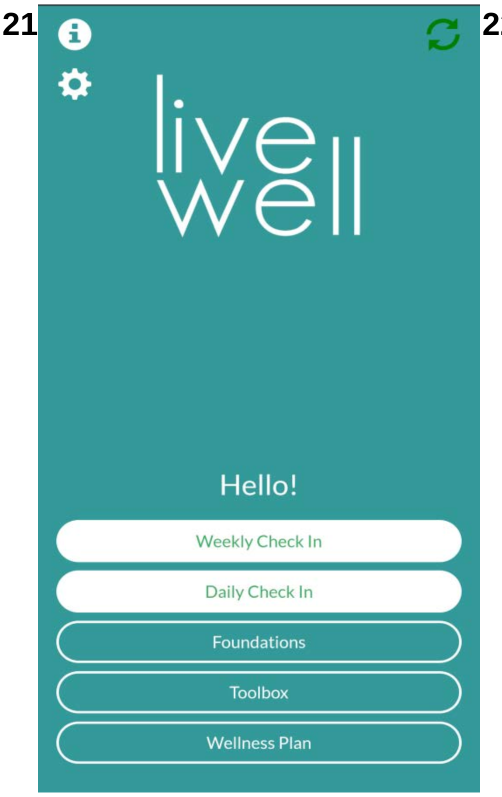

22

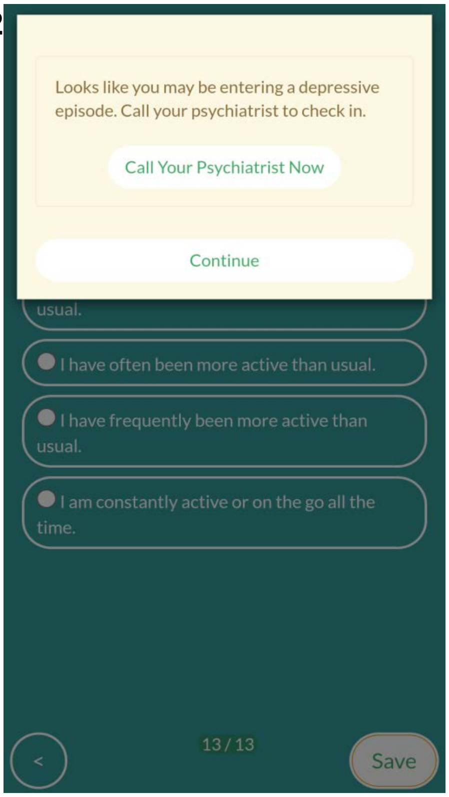

LiveWell Use Case Scenario: User continues to last page of Daily Review which suggests reviewing personalized early warning signs in the Awareness and Action section of the Wellness Plan (**20**). User exits app.

On a subsequent day, user opens app. Because it is a Sunday, both the Daily and Weekly Check In are highlighted indicating tasks to be completed (**21**). User completes PHQ8 questions in the Weekly Check In (not shown) and receives a pop-up notification to contact psychiatrist due to a possible depressive episode (**22**). User exits via link to phonebook and psychiatrist's number.
