# Supplementary material for: A Smartphone-Based Self-management Intervention for Individuals With Bipolar Disorder (LiveWell): Empirical and Theoretical Framework, Intervention Design, and Study Protocol for a Randomized Controlled Trial
Source: JMIR Res Protoc. 2022 Feb 21;11(2):e30710. doi: 10.2196/30710 (PMC8902672; doi:10.2196/30710)
Supplement: Multimedia Appendix 5 [file resprot_v11i2e30710_app5.pdf]

Exit Questionnaire

Please complete the survey below.

Thank you!

Part I: Overall

		Strongly Disagree	Disagree	Somewhat Disagree	Neither Agree Nor Disagree	Somewhat Agree	Agree	Strongly Agree
1)	The application is visually appealing.	○	○	○	○	○	○	○
2)	It was easy to move from one page to another.	○	○	○	○	○	○	○
3)	The overall organization of the application is easy to understand.	○	○	○	○	○	○	○
4)	Individual pages are well designed.	○	○	○	○	○	○	○
5)	I was able to complete my tasks in a reasonable amount of time.	○	○	○	○	○	○	○
6)	Terminology used in this application is clear.	○	○	○	○	○	○	○
7)	The content of the application met my expectations.	○	○	○	○	○	○	○
8)	Overall the application is easy to use.	○	○	○	○	○	○	○
9)	I would be likely to use this application in the future.	○	○	○	○	○	○	○

Part II: Foundations

		Strongly Disagree	Disagree	Somewhat Disagree	Neither Agree Nor Disagree	Somewhat Agree	Agree	Strongly Agree
10)	I found the lessons easy to understand.	○	○	○	○	○	○	○
11)	I found the lessons interesting.	○	○	○	○	○	○	○
12)	The lessons were about the right length.	○	○	○	○	○	○	○
13)	I found the lessons relevant to me.	○	○	○	○	○	○	○
14)	I learned something new from the lessons.	○	○	○	○	○	○	○
15)	I was motivated to make a change after reading the lessons.	○	○	○	○	○	○	○

Part III: Toolbox

		Strongly Disagree	Disagree	Somewhat Disagree	Neither Agree Nor Disagree	Somewhat Agree	Agree	Strongly Agree
16)	I found the Toolbox easy to understand.	○	○	○	○	○	○	○
17)	I found the Toolbox interesting.	○	○	○	○	○	○	○
18)	I found the Toolbox relevant to me.	○	○	○	○	○	○	○
19)	I learned something new from the Toolbox.	○	○	○	○	○	○	○
20)	I was motivated to make a change by using the Toolbox.	○	○	○	○	○	○	○
21)	I looked over most of the skills in the Toolbox section of the application.	○	○	○	○	○	○	○
22)	I found skills that I practiced routinely.	○	○	○	○	○	○	○
23)	I would practice skills more if there were tools to help me practice them (e.g. audio/visual guided meditation techniques).	○	○	○	○	○	○	○
24)	I would practice skills more if each Foundation lesson had specific instructions on which skills to practice.	○	○	○	○	○	○	○
25)	I would practice skills more if I selected 1-3 skills to practice each week.	○	○	○	○	○	○	○
26)	I would practice skills more if I knew the application would track my practice and provide feedback.	○	○	○	○	○	○	○

Part IV: Wellness Plan

	Strongly Disagree	Disagree	Somewhat Disagree	Neither Agree Nor Disagree	Somewhat Agree	Agree	Strongly Agree
27) I found the Wellness Plan layout easy to understand.	○	○	○	○	○	○	○
28) I found the idea of a Wellness Plan interesting.	○	○	○	○	○	○	○
29) I found the sections of the Wellness Plan were relevant to me.	○	○	○	○	○	○	○
30) I learned something new from developing my Wellness Plan.	○	○	○	○	○	○	○
31) I was motivated to make a change after completing my Wellness Plan.	○	○	○	○	○	○	○
32) I liked being able to personalize my Wellness Plan.	○	○	○	○	○	○	○
33) Having and using my personal Wellness Plan was useful for me.	○	○	○	○	○	○	○
34) I actively used the ideas and plans that I developed for my lifestyle skills (SMARTS).	○	○	○	○	○	○	○
35) I actively used the ideas and plans that I developed for managing symptoms in my Awareness & Action section.	○	○	○	○	○	○	○

Part V: Daily Check In

	Strongly Disagree	Disagree	Somewhat Disagree	Neither Agree Nor Disagree	Somewhat Agree	Agree	Strongly Agree
36) I found the Daily Check In easy to use.	○	○	○	○	○	○	○
37) Using the Daily Check In made me more aware of my medication use.	○	○	○	○	○	○	○
38) Using the Daily Check In made me more aware of how much I was sleeping.	○	○	○	○	○	○	○
39) Using the Daily Check In made me more aware of my routine.	○	○	○	○	○	○	○
40) Using the Daily Check In made me more aware of symptoms and early warning signs.	○	○	○	○	○	○	○
41) I found using the Daily Check In helpful.	○	○	○	○	○	○	○

Part VI: Daily Review

		Strongly Disagree	Disagree	Somewhat Disagree	Neither Agree Nor Disagree	Somewhat Agree	Agree	Strongly Agree
42)	I found the Daily Review easy to understand.	○	○	○	○	○	○	○
43)	I found the Daily Review interesting.	○	○	○	○	○	○	○
44)	I found the Daily Review relevant to me.	○	○	○	○	○	○	○
45)	I learned something new from the Daily Review.	○	○	○	○	○	○	○
46)	I was motivated to make a change after completing the Daily Review.	○	○	○	○	○	○	○
47)	I often followed up and practiced skills suggested in the Daily Review.	○	○	○	○	○	○	○

Part VII: Weekly Survey

		Strongly Disagree	Disagree	Somewhat Disagree	Neither Agree Nor Disagree	Somewhat Agree	Agree	Strongly Agree
48)	Taking the Weekly Survey on Sunday worked well with my schedule.	○	○	○	○	○	○	○
49)	I found the Weekly Survey easy to understand.	○	○	○	○	○	○	○
50)	The Weekly Survey was relevant to me.	○	○	○	○	○	○	○
51)	Using the Weekly Survey made me more aware of how I was doing.	○	○	○	○	○	○	○
52)	I had difficulty remembering or reflecting on if I had symptoms and what symptoms I experienced in the past week.	○	○	○	○	○	○	○
53)	Using the Weekly Survey motivated me to make changes.	○	○	○	○	○	○	○
54)	Taking the Weekly Survey was useful.	○	○	○	○	○	○	○

Part VIII: Reminders

		Strongly Disagree	Disagree	Somewhat Disagree	Neither Agree Nor Disagree	Somewhat Agree	Agree	Strongly Agree
55)	The reminders came on schedule as I programmed them to.	○	○	○	○	○	○	○
56)	Once I completed my daily LiveWell activities the reminders stopped appearing.	○	○	○	○	○	○	○
57)	I found the reminders useful.	○	○	○	○	○	○	○
58)	I relied on the reminders to complete my daily LiveWell activities.	○	○	○	○	○	○	○
59)	I found the reminders irritating.	○	○	○	○	○	○	○

Part IX: LiveWell Clinical Status Summary

		Strongly Disagree	Disagree	Somewhat Disagree	Neither Agree Nor Disagree	Somewhat Agree	Agree	Strongly Agree
60)	I looked at the LiveWell Clinical Status Summary on a regular basis.	○	○	○	○	○	○	○
61)	I looked at the LiveWell Clinical Status Summary when I was having problems.	○	○	○	○	○	○	○
62)	I found the LiveWell Clinical Status Summary useful.	○	○	○	○	○	○	○
63)	I found the LiveWell Clinical Status Summary easy to understand.	○	○	○	○	○	○	○

Part X: Coach

		Strongly Disagree	Disagree	Somewhat Disagree	Neither Agree Nor Disagree	Somewhat Agree	Agree	Strongly Agree
64)	I found the coach supportive.	○	○	○	○	○	○	○
65)	I found the coach calls useful.	○	○	○	○	○	○	○
66)	Having the coach calls motivated me to read the lessons.	○	○	○	○	○	○	○
67)	Having the coach calls motivated me to read and practice skills.	○	○	○	○	○	○	○
68)	I got more out of the application by working with the coach.	○	○	○	○	○	○	○
69)	I was motivated to make a change after phone calls with the coach.	○	○	○	○	○	○	○
70)	I found the coach's role beneficial to my use of the application.	○	○	○	○	○	○	○
71)	The coach calls were an appropriate length.	○	○	○	○	○	○	○
72)	I was able to schedule the coach calls at times that were convenient for me.	○	○	○	○	○	○	○

Part XI: Psychiatrist

	Strongly Disagree	Disagree	Somewhat Disagree	Neither Agree Nor Disagree	Somewhat Agree	Agree	Strongly Agree
73) Using LiveWell helped me communicate with my psychiatrist about how I was doing.	○	○	○	○	○	○	○
74) Using LiveWell led me to make changes in how I worked with my psychiatrist.	○	○	○	○	○	○	○
75) I was comfortable with my psychiatrist having access to the data collected by the application.	○	○	○	○	○	○	○

Part XII: Outcomes

	Strongly Disagree	Disagree	Somewhat Disagree	Neither Agree Nor Disagree	Somewhat Agree	Agree	Strongly Agree
76) My use of the application increased my medication adherence.	○	○	○	○	○	○	○
77) My use of the application helped me maintain a more regular routine.	○	○	○	○	○	○	○
78) My use of the application helped me to get the recommended amount of sleep.	○	○	○	○	○	○	○
79) My use of the application increased my ability to identify, monitor, and manage early warning signs and symptoms.	○	○	○	○	○	○	○
80) I found my moods more balanced after participating in the program.	○	○	○	○	○	○	○
81) I feel that I am better equipped to manage bipolar disorder after participating in the program.	○	○	○	○	○	○	○

Part XIII: Study Equipment/Technical Issues/Privacy

	Strongly Disagree	Disagree	Somewhat Disagree	Neither Agree Nor Disagree	Somewhat Agree	Agree	Strongly Agree
82) I found the study equipment easy to use.	○	○	○	○	○	○	○
83) I enjoyed using the study equipment.	○	○	○	○	○	○	○
84) I used the study phone as I would my primary phone.	○	○	○	○	○	○	○
85) I experienced technical issues that impeded my use of the application or study equipment.	○	○	○	○	○	○	○
86) I was able to problem solve technical issues that came up on my own.	○	○	○	○	○	○	○
87) I was concerned about my privacy while using the study equipment.	○	○	○	○	○	○	○
88) I wore the watch 24/7 as suggested.	○	○	○	○	○	○	○
89) I felt the study team was helpful and responsive to my technical issues.	○	○	○	○	○	○	○
90) The watch was comfortable to wear all day.	○	○	○	○	○	○	○
91) The battery life of the phone was adequate.	○	○	○	○	○	○	○

LW-EI: LiveWell Exit Interview V2
ID:___________ Date:________ Follow-Up Month: ____________ Interviewer:____________

Prepare for interview: $, Check participant completed PDQ and Exit Questionnaire

Introduction

We would like you to share your thoughts about using the *LiveWell* system.

Although we like hearing positive comments, any information about problems is very helpful.

Please don't hesitate to tell us about things that didn't work so well or that you didn't like.

Overview/Outcomes

1. Tell me about your overall experience using the *LiveWell* application?

____

____

____

2. What is your strongest memory from using LiveWell?

____

____

____

3. What do think worked best for you? What, if anything, made it easier to stay well?
What, if anything, did you like or find useful about using the application?

____

____

____

4. What do think worked least well for you? What, if anything, made it harder to stay well?
What did you not like or not find useful about using the application?

____

____

____

5. What impact, if any, did using LiveWell have on your life?

____
____
____

6. Did you have a clear objective when you started using LiveWell?

What was it? Did you achieve it?

____
____
____

7. How would you describe the idea behind how LiveWell is meant to help you?

____
____
____

LW-EI: LiveWell Exit Interview V2
ID:___________ Date:________ Follow-Up Month: ____________ Interviewer:____________

Targets

1. How has your participation in *LiveWell* affected your medication use?

2. How has your participation affected the duration of sleep you get each day? Your sleep habits?

3. How has your participation affected your daily routine?

4. How has your participation affected your ability to identify, monitor, and manage early warning signs? What about lingering symptoms?

Foundations

1. What was your overall experience with the Foundations?

____

____

____

2. What, if anything, did you like or find useful about the lessons?

____

____

____

3. What did you find confusing, unclear, or not useful about the lessons?

____

____

____

4. What did you feel was missing or that you would have liked to learn more about?

   [For each topic area covered in a lesson, should there be more details about which skills to practice?]

   [Should the lessons should be more interactive (e.g. providing tools to practice specific skills)?]

____

____

____

Toolbox

1. What was your overall experience with the Toolbox section?

____

____

____

2. Tell me about your use of the Toolbox? [When, Why, How]

____

____

____

3. What, if anything, did you like or find useful about the Toolbox?

____

____

____

4. What did you find confusing, unclear, or not useful about the Toolbox?

____

____

____

5. What would help you to select and practice skills regularly? [specific skills for each lesson, more interactive skills tools, more instructions, worksheets, selecting and discussing skills practiced with the coach]

____

____

____

6. If you could change something about the Toolbox, what would that be?

____

____

____

LW-EI: LiveWell Exit Interview V2
ID:___________ Date:________ Follow-Up Month: ____________ Interviewer:____________

Wellness Plan

1. What was your overall experience with the Wellness Plan?

____

____

____

2. What, if anything, did you like or find useful about the Wellness Plan?

____

____

____

3. What did you find challenging, unclear, or not useful about the Wellness Plan?

____

____

____

4. If you could change anything about the Wellness Plan, what would you change?

____

____

____

<u>Wellness Plan: My Resources</u>

1. What did you think about the My Resources section?
   [Show on phone: My Medications, My Team, My Skills, My Charts]

____

____

____

2. Can you tell me about your use of this section? [When, Why, How]
   [Use of My Meds; Use of My Team; Use of My Skills; Review of charts]

____

____

____

3. What, if anything, did you like or find useful about My Resources?

____

____

____

4. What did you find challenging, unclear, or not useful about My Resources?

____

____

____

5. If you could change anything about My Resources, what would you change?

____

____

____

Wellness Plan: Reduce Risk

1. What did you think about the Reduce Risk section?
   [Show on phone: personalized SMARTS and links to related skills]

____

____

____

2. Can you tell me about your use of this section? [When, Why, How]
   [Sleep, Meds, Abstain, Routine, Tranquil, Social]

____

____

____

3. What, if anything, did you like or find useful about Reduce Risk?

____

____

____

4. Was there anything you found challenging, unclear, or not useful about Reduce Risk?

____

____

____

5. If you could change anything about Reduce Risk, what would you change?

____

____

____

Wellness Plan: Awareness & Action

1. What did you think about the Awareness & Action section?

   [Show on phone; personalized anchors and plan, definition of wellness ratings]

____

____

____

2. Can you tell me about your use of this section? [When, Why, How]

____

____

____

3. What, if anything, did you like or find useful about Awareness & Action?

____

____

____

4. Was there anything you found challenging, unclear, or not useful about Awareness & Action?

____

____

____

5. If you could change anything about Awareness & Action, what would you change?

____

____

____

Daily Check In

1. What was your overall experience with the Daily Check In?

2. Can you walk me through your typical use of the Daily Check In?

3. Did you experience any difficulties completing the Daily Check In?
   [anything you did to make completing the Daily Check In easier]

4. What, if anything, did you like or find useful about the Daily Check In?

5. Was there anything you found challenging, missing, or not useful about the Check In?

6. If you could change anything about the Daily Check In, what would you change?

LW-EI: LiveWell Exit Interview V2
ID:___________ Date:________ Follow-Up Month: ____________ Interviewer:____________

Daily Review

1. What was your overall experience with the Daily Review?

____
____
____

2. What, if anything, did you like or find useful about the Daily Review?

____
____
____

3. Was there anything you found confusing, unclear, or not useful about the Daily Review?

____
____
____

4. What did you think about the feedback in the Daily Review?

____
____
____

5. How did you use it? Did you follow up on any of the feedback? Did you make any changes?

____
____
____

6. If you could change anything about the Daily Review, what would you change?

____
____
____

Weekly Surveys

1. Overall, how would you describe your experience completing the Weekly Surveys?

____

____

____

2. What, if anything, did you like or find useful about completing the Weekly Surveys?

____

____

____

3. Was there anything you found challenging, unclear, or not useful regarding the Weekly Surveys or its specific questions?

____

____

____

4. What would you change about the Weekly Surveys? [Explore thoughts on ews checklists]

____

____

____

Reminders

1. What was your overall experience with the daily reminders? [Reliability – received, on time]

____
____
____

2. Were the daily reminders useful? How so?

____
____
____
____

3. Was there anything about the daily reminders you didn't like?

____
____
____

4. How do you think the reminders could be improved?

____
____
____

LW-EI: LiveWell Exit Interview V2
ID:___________ Date:________ Follow-Up Month: ____________ Interviewer:____________

Clinical Status Summary

1. Did you look at the summary reports? How often? If not, why not?

____

____

____

2. Any questions, thoughts, or feedback about the report?

____

____

____

3. Are there any additional items that would have been useful to have in the report?

____

____

____

4. Are there any items you think should be dropped from the report?

____

____

____

LW-EI: LiveWell Exit Interview V2
ID:___________ Date:________ Follow-Up Month: ____________ Interviewer:____________

<u>Coach</u>

1. How would you describe your overall experience working with the coach?

____

____

____

2. What, if anything, did you like or find useful about working with the coach?

____

____

____

3. Was there anything you found difficult or not useful about working with the coach?

____

____

____

4. If you could change anything about the coach’s involvement, what would it be?

____

____

____

Psychiatrist

1. In what ways, if any, has your participation in *LiveWell* affected how you work with your psychiatrist?

____

____

____

2. What changes would you suggest to the *LiveWell* program to make it more useful in terms of terms of working with your psychiatrist?

____

____

____

LW-EI: LiveWell Exit Interview V2
ID:___________ Date:________ Follow-Up Month: ____________ Interviewer:____________

Use of Study Equipment

1. Can you walk me through your typical use of the study phone and watch?

   [Where did you keep it, how frequently did you check it, etc.?]

____

____

____

2. What difficulties, if any, did you have using the study phone every day?

____

____

____

3. What difficulties, if any, did you have using the watch all day every day?

____

____

____

4. Anything you wish you'd had during the study to make it easier?

   [extra phone chargers, portable chargers, car chargers, etc]

____

____

____

Technical Issues

1. What technical issues, if any, did you experience with the phone or watch? [trouble with reception, battery life, receiving and responding to prompts, completing daily check-ins and/or weekly surveys]

____

____

____

If issues present, did you feel like staff was responsive in helping you address these issues?

____

____

____

If issues present, did these technical issues affect your intended use of the application? How?

____

____

____

Privacy

1. What concerns, if any, did you have about your privacy while using the system?

____

____

____

Thank you for your valuable feedback, is there anything else you'd like to add before we end?

____

____

____

____

____

Interviewer Comments
