# Supplementary material for: A Smartphone-Based Self-management Intervention for Individuals With Bipolar Disorder (LiveWell): Empirical and Theoretical Framework, Intervention Design, and Study Protocol for a Randomized Controlled Trial
Source: JMIR Res Protoc. 2022 Feb 21;11(2):e30710. doi: 10.2196/30710 (PMC8902672; doi:10.2196/30710)
Supplement: Multimedia Appendix 6 [file resprot_v11i2e30710_app6.pdf]

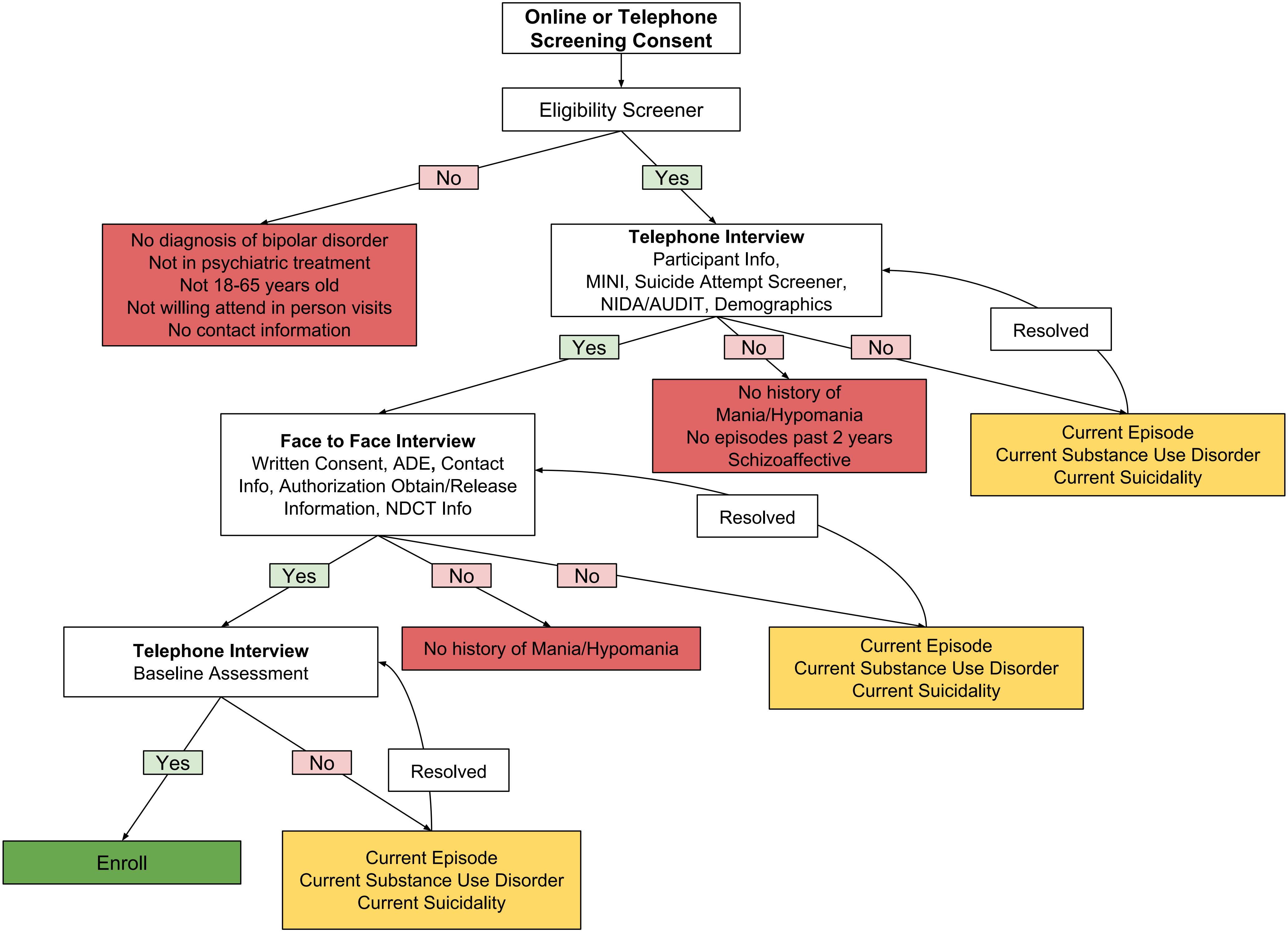
Online or Telephone Screening Consent
Eligibility Screener
No
Yes
No diagnosis of bipolar disorder
Not in psychiatric treatment
Not 18-65 years old
Not willing attend in person visits
No contact information
Telephone Interview
Participant Info,
MINI, Suicide Attempt Screener,
NIDA/AUDIT, Demographics
Resolved
Yes
No
No
No history of
Mania/Hypomania
No episodes past 2 years
Schizoaffective
Current Episode
Current Substance Use Disorder
Current Suicidality
Face to Face Interview
Written Consent, ADE, Contact Info, Authorization Obtain/Release Information, NDCT Info
Resolved
Yes
No
No
Telephone Interview
Baseline Assessment
No history of Mania/Hypomania
Current Episode
Current Substance Use Disorder
Current Suicidality
Yes
No
Resolved
Enroll
Current Episode
Current Substance Use Disorder
Current Suicidality

LiveWell Eligibility Screener (Online or Telephone)

Are you between 18 and 65 years old?	Yes ☐ No ☐
Have you been told by a healthcare professional that you have bipolar disorder?	Yes ☐ No ☐
Are you currently working with a psychiatrist?	Yes ☐ No ☐
Are you willing to travel to two in person visits at our office located at: Chicago IL or Bloomington MN	Yes ☐ No ☐

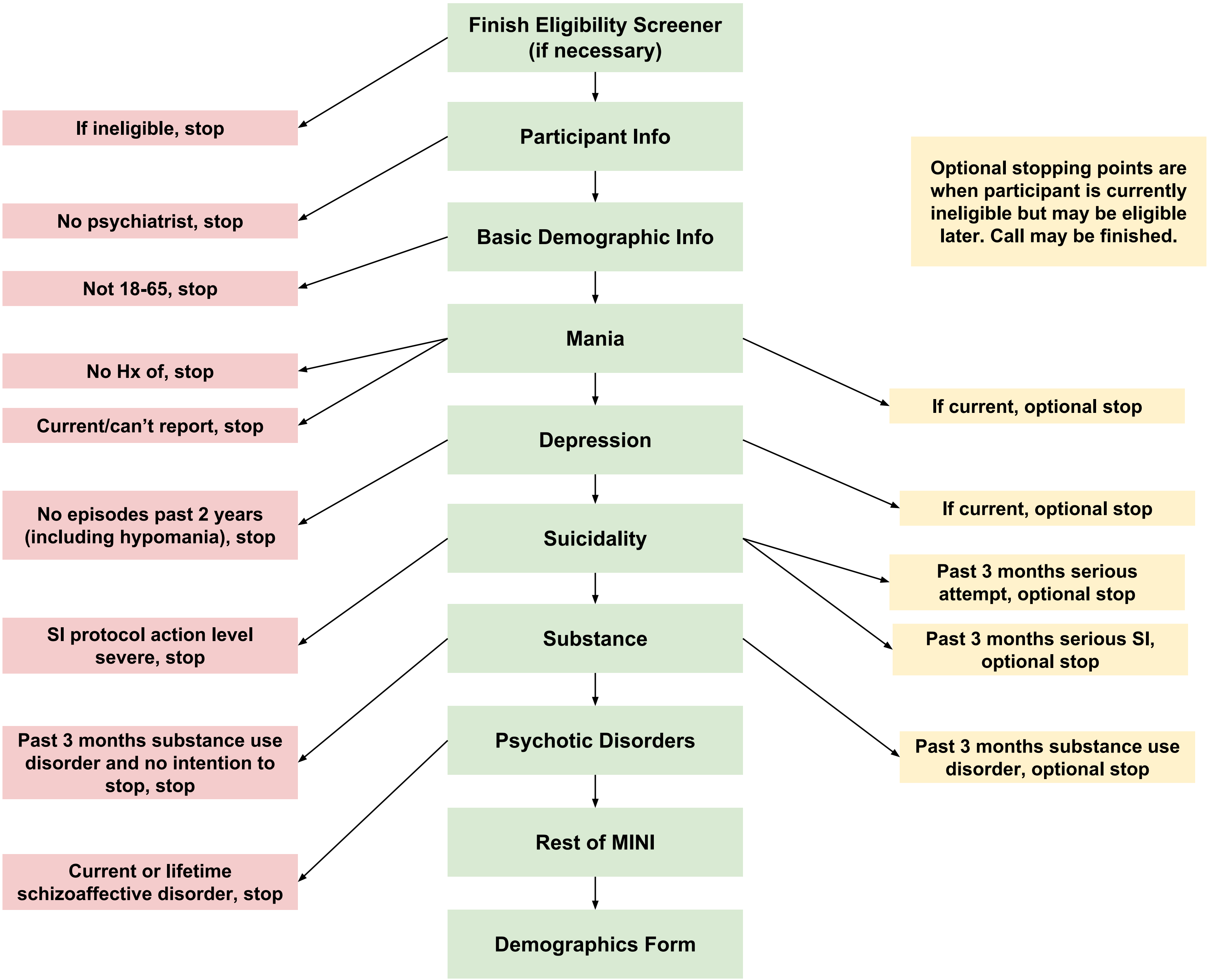
Finish Eligibility Screener (if necessary)
If ineligible, stop
Participant Info
No psychiatrist, stop
Basic Demographic Info
Not 18-65, stop
Optional stopping points are when participant is currently ineligible but may be eligible later. Call may be finished.
Mania
No Hx of, stop
Current/can’t report, stop
If current, optional stop
Depression
No episodes past 2 years (including hypomania), stop
If current, optional stop
Suicidality
Past 3 months serious attempt, optional stop
Past 3 months serious SI, optional stop
SI protocol action level severe, stop
Substance
Past 3 months substance use disorder and no intention to stop, stop
Past 3 months substance use disorder, optional stop
Psychotic Disorders
Current or lifetime schizoaffective disorder, stop
Rest of MINI
Demographics Form

LiveWell Participant Information

Date ______________ Interviewer ________

Participant Name ____

How did you hear about the study? ______________________________

Psychiatrist Information

Name: ______________________________ HealthPartners psychiatrist? Yes No N/A

Phone Number: ______________________________

Office Affiliation: ______________________________

Frequency of Visits/Date of Last Visit: ______________________________

Diagnostic Information

Diagnosis received from psychiatrist/other provider: ______________________________

Current Medications (dose, frequency): ______________________________

____

Contact Information

Confirm email address: ______________________________

Alternate Phone Number (and type): ______________________________

Age

☐☐

Date of birth

☐☐ / ☐☐ / ☐☐☐☐

Month **Day** **Year**

Gender

☐ **Male**

☐ **Female**

☐ **Non Binary/ third gender**

☐ **Prefer to self describe:** ______________

☐ **Prefer not to say**

Ethnicity

☐ **0 = Hispanic or Latino**
(Of Cuban, Mexican, Puerto Rican, Dominican, South or Central American, or other Spanish culture or origin, regardless of race)

☐ **1 = Not Hispanic or Latino**

Race (You may pick more than one)

☐ **0 = Black or African American**
(Having origins in any of the black racial groups of Africa)

☐ **1 = American Indian (North, South or Central) or Alaska Native**
(Having origins in any of the original peoples of North, Central or South America)

☐ **2 = Asian**
(Having origins in any of the original peoples of the Far East, Southeast Asia, or the Indian Subcontinent. For example: Cambodia, China, India, Japan, Korea, Malaysia, Pakistan, the Philippine Islands, Thailand, and Vietnam)

☐ **3 = Native Hawaiian or Other Pacific Islander**
(Having origins in any of the original peoples of Hawaii, Guam, Samoa or other Pacific Islands)

☐ **4 = White**
(Having origins in any of the original peoples of Europe, the Middle East, or North Africa)

M.I.N.I.

MINI INTERNATIONAL NEUROPSYCHIATRIC INTERVIEW

REVISED VERSION B

English Version 5.0.0

DSM-IV

USA: **D. Sheehan, J. Janavs, R. Baker, K. Harnett-Sheehan, E. Knapp, M. Sheehan**
University of South Florida - Tampa

FRANCE: **Y. Lecrubier, E. Weiller, T. Hergueta, P. Amorim, L. I. Bonora, J. P. Lépine**
Hôpital de la Salpétrière - Paris

© Copyright 1992-2006 Sheehan DV & Lecrubier Y

All rights reserved. No part of this document may be reproduced or transmitted in any form, or by any means, electronic or mechanical, including photocopying, or by any information storage or retrieval system, without permission in writing from Dr. Sheehan or Dr. Lecrubier. Researchers and clinicians working in nonprofit or publicly owned settings (including universities, nonprofit hospitals, and government institutions) may make copies of a M.I.N.I. instrument for their own clinical and research use.

DISCLAIMER

Our aim is to assist in the assessment and tracking of patients with greater efficiency and accuracy. Before action is taken on any data collected and processed by this program, it should be reviewed and interpreted by a licensed clinician.

This program is not designed or intended to be used in the place of a full medical and psychiatric evaluation by a qualified licensed physician – psychiatrist. It is intended only as a tool to facilitate accurate data collection and processing of symptoms elicited by trained personnel.

Patient Name:		***Patient Number:***	
Date of Birth:		***Time Interview Began:***	
Interviewer's Name:		***Time Interview Ended:***	
Date of Interview:		***Total Time:***	

	MODULES	TIME FRAME	MEETS CRITERIA	DSM-IV	ICD-10
A	MANIC EPISODE	Current	☐	296.00-296.06	F30.x-F31.9
		Past	☐		
	HYPOMANIC EPISODE	Current	☐	296.80-296.89	F31.8-F31.9/F34.0
		Past	☐		
B	MAJOR DEPRESSIVE EPISODE	Current (2 weeks)	☐	296.20-296.26 Single	F32.x
		Recurrent	☐	296.30-296.36 Recurrent	F33.x
C	SUICIDALITY	Current (Past Month)	☐		
		Risk: ☐ Low ☐ Medium ☐High			
D	ALCOHOL DEPENDENCE	Past 3 Months	☐	303.9	F10.2x
	ALCOHOL ABUSE	Past 3 Months	☐	305.00	F10.1
E	SUBSTANCE DEPENDENCE (Non-alcohol)	Past 3 Months	☐	304.00-.90/305.20-.90	F11.1-F19.1
	SUBSTANCE ABUSE (Non-alcohol)	Past 3 Months	☐	304.00-.90/305.20-.90	F11.1-F19.1
F	PSYCHOTIC DISORDERS	Lifetime	☐	295.10-295.90/297.1/ 297.3/293.81/293.82/ 293.89/298.8/298.9	F20.xx-F29
		Current	☐		
	MOOD DISORDER WITH PSYCHOTIC FEATURES	Lifetime	☐	296.24/296.34/296.44	F32.3/F33.3/
		Current	☐	296.24/296.34/296.44	F30.2/F31.2/F31.5 F31.8/F31.9/F39
G	DYSTHYMIA	Current (Past 2 years)	☐	300.4	F34.1
H	PANIC DISORDER	Current (Past Month)	☐	300.01/300.21	F40.01-F41.0
		Limited Lifetime	☐		
		Lifetime	☐		
I	AGORAPHOBIA	Current (Past month)	☐	300.22	F40.00
J	SOCIAL PHOBIA (Social Anxiety Disorder)	Current (Past month)	☐	300.23	F40.1
K	OBSESSIVE-COMPULSIVE DISORDER	Current (Past month)	☐	300.3	F42.8
L	POSTTRAUMATIC STRESS DISORDER	Current (Past month)	☐	309.81	F43.1
M	GENERALIZED ANXIETY DISORDER	Current (Past 6 months)	☐	300.02	F41.1
N	ANOREXIA NERVOSA	Current (Past 3 months)	☐	307.1	F50.0
O	BULIMIA NERVOSA	Current (Past 3 months)	☐	307.51	F50.2
	ANOREXIA NERVOSA, BINGE EATING/PURGING TYPE	Current	☐	307.1	F50.0

GENERAL INSTRUCTIONS

The M.I.N.I. was designed as a brief structured interview for the major Axis I psychiatric disorders in DSM-IV and ICD-10. Validation and reliability studies have been done comparing the M.I.N.I. to the SCID-P for DSM-III-R and the CIDI (a structured interview developed by the World Health Organization for lay interviewers for ICD-10). The results of these studies show that the M.I.N.I. has acceptably high validation and reliability scores, but can be administered in a much shorter period of time (mean 18.7 ± 11.6 minutes, median 15 minutes) than the above referenced instruments. It can be used by clinicians, after a brief training session. Lay interviewers require more extensive training.

INTERVIEW:

In order to keep the interview as brief as possible, inform the patient that you will conduct a clinical interview that is more structured than usual, with very precise questions about psychological problems which require a yes or no answer.

GENERAL FORMAT:

The M.I.N.I. is divided into **modules** identified by letters, each corresponding to a diagnostic category.
•At the beginning of each diagnostic module (except for psychotic disorders module), screening question(s) corresponding to the main criteria of the disorder are presented in a **gray box**.
•At the end of each module, diagnostic box(es) permit the clinician to indicate whether diagnostic criteria are met.

CONVENTIONS:

Sentences written in « normal font » should be read exactly as written to the patient in order to standardize the assessment of diagnostic criteria.

Sentences written in « CAPITALS » should not be read to the patient. They are instructions for the interviewer to assist in the scoring of the diagnostic algorithms.

Sentences written in « **bold** » indicate the time frame being investigated. The interviewer should read them as often as necessary. Only symptoms occurring during the time frame indicated should be considered in scoring the responses.

Answers with an arrow above them (➨) indicate that one of the criteria necessary for the diagnosis(es) is not met. In this case, the interviewer should go to the end of the module, circle « **NO** » in all the diagnostic boxes and move to the next module.

When terms are separated by a *slash (/)* the interviewer should read only those symptoms known to be present in the patient (for example, question H6).

Phrases in (parentheses) are clinical examples of the symptom. These may be read to the patient to clarify the question.

RATING INSTRUCTIONS:

All questions must be rated. The rating is done at the right of each question by circling either Yes or No. Clinical judgment by the rater should be used in coding the responses. The rater should ask for examples when necessary, to ensure accurate coding. The patient should be encouraged to ask for clarification on any question that is not absolutely clear.

The clinician should be sure that <u>each dimension</u> of the question is taken into account by the patient (for example, time frame, frequency, severity, and/or alternatives).
<u>Symptoms better accounted for by an organic cause or by the use of alcohol or drugs should not be coded positive in the M.I.N.I.</u> The M.I.N.I. Plus has questions that investigate these issues.

For any questions, suggestions, need for a training session, or information about updates of the M.I.N.I., please contact :

David V Sheehan, M.D., M.B.A.
University of South Florida College of Medicine
3515 East Fletcher Avenue
Tampa, FL USA 33613-4788
tel : +1 813 974 4544; fax : +1 813 974 4575
e-mail : dsheehan@hsc.usf.edu

Yves Lecrubier, M.D. / Thierry Hergueta, M.S.
INSERM U302
Hôpital de la Salpétrière
47, boulevard de l'Hôpital
F. 75651 PARIS, FRANCE
tel : +33 (0) 1 42 16 16 59; fax : +33 (0) 1 45 85 28 00
e-mail : hergueta@ext.jussieu.fr

A. MANIC AND HYPOMANIC EPISODES

(➨ MEANS: GO TO DIAGNOSTIC BOXES, CIRCLE NO AND MOVE TO THE NEXT MODULE)

A1	a	Have you **ever** had period of time when you were feeling 'up' or 'high' or 'hyper' or so full of energy or full of yourself that you got into trouble, - or that other people thought you were not your usual self? (Do not consider times when you were intoxicated on drugs or alcohol.)	NO	YES
		IF PATIENT IS PUZZLED OR UNCLEAR ABOUT WHAT YOU MEAN BY 'UP' OR 'HIGH' OR 'HYPER', CLARIFY AS FOLLOWS: By 'up' or 'high' or 'hyper' I mean: having elated mood; increased energy; needing less sleep; having rapid thoughts; being full of ideas; having an increase in productivity, motivation, creativity, or impulsive behavior; phoning or working excessively or spending more money.		
		IF NO, CODE NO TO **A1b**: IF **YES** ASK:		
	b	Are you currently feeling 'up' or 'high' or 'hyper' or full of energy?	NO	YES
A2	a	Have you **ever** been persistently irritable, for several days, so that you had arguments or verbal or physical fights, or shouted at people outside your family? Have you or others noticed that you have been more irritable or over reacted, compared to other people, even in situations that you felt were justified?	NO	YES
		IF NO, CODE NO TO **A2b**: IF **YES** ASK:		
	b	Are you currently feeling persistently irritable?	NO	YES
		IS **A1a** OR **A2a** CODED **YES**?	➨ NO	YES

A3 IF **A1b** OR **A2b** = **YES**: EXPLORE THE **CURRENT** AND THE MOST SYMPTOMATIC **PAST** EPISODE, OTHERWISE

IF **A1b** AND **A2b** = **NO**: EXPLORE ONLY THE MOST SYMPTOMATIC **PAST** EPISODE

During the times when you felt high, full of energy, or irritable did you:

		Current Episode		Past Episode	
a	Feel that you could do things others couldn't do, or that you were an especially important person? IF **YES**, ASK FOR EXAMPLES.<br>THE EXAMPLES ARE CONSISTENT WITH A DELUSIONAL IDEA. Current Episode ❐ No ❐ Yes<br>Past Episode ❐ No ❐ Yes	NO	YES	NO	YES
b	Need less sleep (for example, feel rested after only a few hours sleep)?	NO	YES	NO	YES
c	Talk too much without stopping, or so fast that people had difficulty understanding?	NO	YES	NO	YES
d	Have racing thoughts?	NO	YES	NO	YES

		Current Episode		Past Episode	
e	Become easily distracted so that any little interruption could distract you?	NO	YES	NO	YES
f	Have a significant increase in your activity or drive, at work, at school, socially or sexually or did you become physically or mentally restless?	NO	YES	NO	YES
g	Want so much to engage in pleasurable activities that you ignored the risks or consequences (for example, spending sprees, reckless driving, or sexual indiscretions)?	NO	YES	NO	YES
A4	What is the longest time these symptoms lasted?				
	a) 3 days or less		❒		❒
	b) 4 to 6 days		❒		❒
	c) 7 days or more		❒		❒
A5	Were you hospitalized for these problems?<br>IF YES, STOP HERE AND CIRCLE YES IN MANIC EPISODE FOR THAT TIME FRAME.	NO	YES	NO	YES
A6	Did these symptoms cause significant problems at home, at work, socially in your relationships with others, at school or some other important way?	NO	YES	NO	YES
A3 SUMMARY: WHEN RATING CURRENT OR PAST EPISODE:		NO	YES	NO	YES

IF **A1a or A1b NO** (NO ELATION)**,** ARE 4 OR MORE **A3** (SYMPTOMS) ANSWERS CODED **YES**?

IF **A1a or A1b YES** (YES ELATION), ARE 3 OR MORE **A3** (SYMPTOMS) ANSWERS CODED **YES**?

CODE YES ONLY IF THE ABOVE 3 OR 4 SYMPTOMS OCCURED DURING THE SAME TIME PERIOD

ELATION REQUIRES ONLY THREE A3 SYMPTOMS, WHILE IRRITABLE MOOD ALONE REQUIRES FOUR A3 SYMPTOMS.

ARE **A3** SUMMARY AND **A5** (YES HOSPITAL) AND **A6** (YES SIG PROBLEMS) CODED **YES**?

OR

ARE **A3** SUMMARY AND **A4c** (7 DAYS OR MORE) AND **A6** (YES SIG PROBLEMS) CODED **YES** AND IS **A5** (NO HOSPITAL) CODED **NO**?

**NO**	**YES**
MANIC EPISODE	
CURRENT	❒
PAST	❒

IF **YES** TO CURRENT MANIC EPISODE, THEN CODE CURRENT HYPOMANIC EPISODE AS **NO.**

IF **YES** TO PAST MANIC EPISODE, THEN CODE PAST HYPOMANIC EPISODE AS **NOT EXPLORED.**

Is A3 SUMMARY **CODED YES** AND ARE **A5** (NO HOSPITAL) AND **A6** (NO SIG PROBLEMS) CODED **NO** AND IS EITHER **A4b** (4-6 DAYS) OR **A4c** (7 DAYS OR MORE) CODED **YES**?

OR

ARE **A3** SUMMARY AND **A4b** (4 TO 6 DAYS) AND **A6** (YES SIG PROBLEMS) CODED **YES** AND IS **A5** (NO HOSPITAL) CODED **NO?**

HYPOMANIC EPISODE

CURRENT ❒ **NO**
❒ **YES**

PAST ❒ **NO**
❒ **YES**
❒ **NOT EXPLORED**

A7 a) IF MANIC EPISODE IS POSITIVE FOR EITHER CURRENT OR PAST ASK:
How many times in your life have you been [manic/own words] for 7 days or more and had several of the symptoms that you described? _______________

b) **MANIC EPISODES**

Season Key
Fall: Sep, Oct, Nov
Winter: Dec, Jan, Feb
Spring: Mar, Apr, May
Summer: Jun, Jul, Aug

MOST SEVERE MANIC EPISODE:

Date of onset: ____________ Age: ________ Date of offset: ____________

MOST RECENT MANIC EPISODE:

Date of onset: ____________ Age: ________ Date of offset: ____________

A8 IF NO MANIC EPISODES IN PAST 2 YEARS ASK:

Did you have episodes lasting just 4-6 days that included several of the symptoms you described? NO YES N/A

IF YES: When was the most recent episode? Was it in the past 2 years? NO YES

A9 Do you have any family history of manic depressive illness or bipolar disorder, or any family member who had mood swings treated with a medication like lithium, sodium valproate (Depakote) or lamotrigine (Lamictal)? NO YES MAYBE

THIS QUESTION IS NOT A CRITERION FOR BIPOLAR DISORDER, BUT IS ASKED TO INCREASE THE CLINICIAN'S VIGILANCE ABOUT THE RISK FOR BIPOLAR DISORDER .

IF YES, PLEASE SPECIFY WHO:____

B. MAJOR DEPRESSIVE EPISODE

(➡ MEANS: GO TO DIAGNOSTIC BOX, CIRCLE NO AND MOVE TO THE NEXT MODULE)

B1	a	Were you ever depressed or down, most of the day, nearly every day, for two weeks?	NO	YES
		IF NO, CODE NO TO **B1b**: IF **YES** ASK:		
	b	For the past two weeks, were you depressed or down, most of the day, nearly every day?	NO	YES
B2	a	Were you ever much less interested in most things or much less able to enjoy the things you used to enjoy most of the time, for two weeks?	NO	YES
		IF NO, CODE NO TO **B2b**: IF **YES** ASK:		
	b	In the past two weeks, were you much less interested in most things or much less able to enjoy the things you used to enjoy, most of the time?	NO	YES
		IS **B1a** OR **B2a** CODED **YES**?	➡ NO	YES

B3 IF **B1b** OR **B2b** = **YES**: EXPLORE THE **CURRENT** AND THE MOST SYMPTOMATIC **PAST** EPISODE, OTHERWISE

IF **B1b** AND **B2b** = **NO**: EXPLORE ONLY THE MOST SYMPTOMATIC **PAST** EPISODE

Over that two week period, when you felt depressed or uninterested:

			Past 2 Weeks		Past Episode	
	a	Was your appetite decreased or increased nearly every day? Did your weight decrease or increase without trying intentionally (i.e., by 5% of body weight or ±8 lb or ± 3.5 kg, for a 160 lb/70 kg person in a month)? IF **YES** TO EITHER, CODE **YES**.	NO	YES	NO	YES
	b	Did you have trouble sleeping nearly every night (difficulty falling asleep, waking up in the middle of the night, early morning wakening or sleeping excessively)? *[How many hours a night, on average?]*	NO	YES	NO	YES
	c	Did you talk or move more slowly than normal or were you fidgety, restless or having trouble sitting still almost every day?	NO	YES	NO	YES
	d	Did you feel tired or without energy almost every day?	NO	YES	NO	YES
	e	Did you feel worthless or guilty almost every day?	NO	YES	NO	YES
		IF **YES**, ASK FOR EXAMPLES. THE EXAMPLES ARE CONSISTENT WITH A DELUSIONAL IDEA. Current Episode ❒ No ❒ Yes; Past Episode ❒ No ❒ Yes				
	f	Did you have difficulty concentrating or making decisions almost every day?	NO	YES	NO	YES
	g	Did you repeatedly consider hurting yourself, feel suicidal, or wish that you were dead? Did you attempt suicide or plan a suicide? IF **YES TO EITHER, CODE YES.**	NO	YES	NO	YES
B4		Did these symptoms cause significant problems at home, at work, socially, at school or in some other important way?	NO	YES	NO	YES

B5 a During your lifetime, did you have (other) episodes of two weeks or more when you felt depressed or uninterested in most things, and had most of the problems we just talked about? NO YES

b **IF YES TO OTHER EPISODES:** In between episodes of depression, did you ever have an interval of at least 2 months, without any significant depression or any significant loss of interest? NO YES

ARE **5** OR MORE ANSWERS (**B1-B3**) CODED **YES** AND IS **B4** CODED YES FOR THAT TIME FRAME?

IF **B5b** IS CODED **YES,** CODE **YES** FOR RECURRENT.

**NO**	**YES**
MAJOR DEPRESSIVE EPISODE	
CURRENT	❒
PAST	❒
RECURRENT	❒

B6 How many times in your life have you been [depressed/own words] for 2 weeks or more and had several of the symptoms that you described?
Between each episode there must be at least 2 months without any significant depression. ________________

B7 How many times in your life have you been hospitalized for psychiatric reasons? ________________

B8 MAJOR DEPRESSIVE EPISODES

Season Key
Fall: Sep, Oct, Nov
Winter: Dec, Jan, Feb
Spring: Mar, Apr, May
Summer: Jun, Jul, Aug

MOST SEVERE MAJOR DEPRESSIVE EPISODE:

Date of onset: ____________ Age: ________ Date of offset: ____________

MOST RECENT MAJOR DEPRESSIVE EPISODE:

Date of onset: ____________ Age: ________ Date of offset: ____________

C. SUICIDALITY

In the past month did you:

				Points
C1	Suffer any accident?	NO	YES	0
	IF NO TO C1, SKIP TO C2; IF YES, ASK C1a,:			
C1a	Plan or intend to hurt yourself in that accident either passively or actively?	NO	YES	0
	IF NO TO C1a, SKIP TO C2: IF YES, ASK C1b,:			
C1b	Did you intend to die as a result of this accident?	NO	YES	0
C2	Think that you would be better off dead or wish you were dead?	NO	YES	1
C3	Want to harm yourself or to hurt or to injure yourself?	NO	YES	2
C4	Think about suicide?	NO	YES	6

IF YES, ASK ABOUT THE INTENSITY AND FREQUENCY OF THE SUICIDAL IDEATION:

Frequency		Intensity	
Occasionally	❒	Mild	❒
Often	❒	Moderate	❒
Very often	❒	Severe	❒

→ Can you control these impulses and state that you will not act on them while in this program? Only score 8 points if response is NO. NO YES 8

C5	Have a suicide plan?	NO	YES	8
C6	Take any active steps to prepare to injure yourself or to prepare for a suicide attempt in which you expected or intended to die?	NO	YES	9
C7	Deliberately injure yourself without intending to kill yourself?	NO	YES	4
C8	Attempt suicide?	NO	YES	10

Hoped to be rescued / survive ❒
Expected / intended to die ❒

In your lifetime:

<u>If yes, fill out Suicide Attempt Screener</u>

C9 Did you ever make a suicide attempt? NO YES 4

IS AT LEAST **1** OF THE ABOVE (EXCEPT C1) CODED **YES**?

NO **YES**

SUICIDE RISK CURRENT

1-8 points	Low	❒
9-16 points	Moderate	❒
≥ 17 points	High	❒

If C1a or C1b = YES, GO to SI PROTOCOL.
OR
If SUICIDE RISK CURRENT = Moderate OR High, GO to SI PROTOCOL.

If SUICIDE RISK CURRENT = High, patient has current severe SI

C10. How many suicide attempts have you made?

Number of attempts: ➡ 0 1 2 3 4-5 6-9 10-20 21-50 Too many to count

Prompts

What happened? Did you receive any medical attention?
Were you hospitalized?
- Medical (Emergency department evaluation, Inpatient medicine, Intensive care; How long)
- Psychiatric (Inpatient, Residential, Intensive outpatient; How long)

C11. When did the most serious suicide attempt you have made occur?

Date (season, year): _____________ Age: _____________ Actual or Potential Lethality: _____________

____

____

____

____

C12. When did the most recent suicide attempt you have made occur? ☐ Same as above

Date (season, year): _____________ Age: _____________ Actual or Potential Lethality: _____________

____

____

____

____

Rating Severity

<u>Actual Lethality/Medical Damage or Potential Lethality</u>

1 - No physical damage (e.g. held pills in hand). Behavior not likely to result in injury.

2 - No physical damage. Behavior likely to result in injury but not likely to cause death.

3 - No physical damage. Behavior likely to result in death despite available medical care.

4 - Minor physical damage (e.g. surface scratches).

5 - Moderate physical damage/Medical attention needed (e.g. conscious but sleepy, somewhat responsive; second-degree burns, bleeding of major vessel).

6 - Moderately severe physical damage/Medical hospitalization and likely intensive care required (e.g., comatose with reflexes intact; third-degree burns less than 20% of body; extensive blood loss but can recover; major fractures).

7 - Severe physical damage/Medical hospitalization with intensive care required (e.g., comatose without reflexes, third degree burns over 20% of body, extensive blood loss with unstable vital signs, major damage to a vital area, respiratory arrest or prolonged coma)

If rating severity = 2, 3, 5, 6, or 7, qualifies as serious suicide attempt

D. ALCOHOL ABUSE AND DEPENDENCE

Now I'd like to ask you a few questions that relate to your experience with alcohol and other drugs. First I am going to ask you about your use of alcoholic beverages during the last 3 months. For the purpose of these questions, a standard drink is equal to 12 oz can/bottle of beer, 5 oz glass of wine, 1 shot of liquor or mixed drink.

(➡ MEANS: GO TO DIAGNOSTIC BOXES, CIRCLE NO IN BOTH AND MOVE TO THE NEXT MODULE)

			➡	
D1		**In the past 3 months**, have you had 3 or more alcoholic drinks within a 3 hour period on 3 or more occasions?	NO	YES
D2		**In the past 3 months**:		
	a	Did you need to drink more in order to get the same effect that you got when you first started drinking?	NO	YES
	b	When you cut down on drinking did your hands shake, did you sweat or feel agitated? Did you drink to avoid these symptoms or to avoid being hungover, for example, "the shakes", sweating or agitation? IF **YES** TO EITHER, CODE **YES**.	NO	YES
	c	During the times when you drank alcohol, did you end up drinking more than you planned when you started?	NO	YES
	d	Have you tried to reduce or stop drinking alcohol but failed?	NO	YES
	e	On the days that you drank, did you spend substantial time in obtaining alcohol, drinking, or in recovering from the effects of alcohol?	NO	YES
	f	Did you spend less time working, enjoying hobbies, or being with others because of your drinking?	NO	YES
	g	Have you continued to drink even though you knew that the drinking caused you health or mental problems?	NO	YES

ARE **3** OR MORE **D2** ANSWERS CODED **YES**?

* IF YES, SKIP D3 QUESTIONS, CIRCLE N/A IN THE ABUSE BOX AND MOVE TO THE NEXT DISORDER. DEPENDENCE PREEMPTS ABUSE.

NO **YES***

ALCOHOL DEPENDENCE
CURRENT

D3		**In the past 3 months:**		
	a	Have you been intoxicated, high, or hungover more than once when you had other responsibilities at school, at work, or at home? Did this cause any problems? (CODE **YES** ONLY IF THIS CAUSED PROBLEMS.)	NO	YES
	b	Were you intoxicated more than once in any situation where you were physically at risk, for example, driving a car, riding a motorbike, using machinery, boating, etc.?	NO	YES
	c	Did you have legal problems more than once because of your drinking, for example, an arrest or disorderly conduct?	NO	YES
	d	Did you continue to drink even though your drinking caused problems with your family or other people?	NO	YES

ARE **1** OR MORE **D3** ANSWERS CODED **YES**?

NO **N/A** **YES**

ALCOHOL ABUSE
CURRENT

E. NON-ALCOHOL PSYCHOACTIVE SUBSTANCE USE DISORDERS

Next I am going to read to you a list of street drugs or medicines. Some of the substances we'll talk about are prescribed by a doctor (like pain medications). But I will only record those if you have taken them without a prescription or for reasons or in doses other than prescribed. I'll also ask you about illicit or illegal drug use.

(➡ MEANS : GO TO THE DIAGNOSTIC BOXES, CIRCLE NO IN ALL, AND MOVE TO THE NEXT MODULE)

E1 a **In the past 3 months**, did you take any of these drugs more than once, to get high, to feel better, or to change your mood?	➡ NO	YES

CIRCLE EACH DRUG TAKEN:

Stimulants: amphetamines, "speed", crystal meth, "crank", "rush", Dexedrine, Ritalin, diet pills.

Cocaine: snorting, IV, freebase, crack, "speedball".

Narcotics: heroin, morphine, Dilaudid, opium, Demerol, methadone, codeine, Percodan, Darvon, OxyContin.

Hallucinogens: LSD ("acid"), mescaline, peyote, PCP ("angel dust", "peace pill"), psilocybin, STP, "mushrooms", "ecstasy", MDA, MDMA, or ketamine ("special K").

Inhalants: "glue", ethyl chloride, "rush", nitrous oxide ("laughing gas"), amyl or butyl nitrate ("poppers").

Marijuana: hashish ("hash"), THC, "pot", "grass", "weed", "reefer".

Tranquilizers: Quaalude, Seconal ("reds"), Valium, Xanax, Librium, Ativan, Dalmane, Halcion, barbiturates, Miltown, GHB, Roofinol, "Roofies".

Miscellaneous: steroids, nonprescription sleep or diet pills. Any others?

SPECIFY MOST USED DRUG(S): ____________________

	CHECK ONE BOX
ONLY ONE DRUG / DRUG CLASS HAS BEEN USED	☐
ONLY THE MOST USED DRUG CLASS IS INVESTIGATED.	☐
EACH DRUG CLASS USED IS EXAMINED SEPARATELY (PHOTOCOPY E2 AND E3 AS NEEDED)	☐

b SPECIFY WHICH DRUG/DRUG CLASS WILL BE EXPLORED IN THE INTERVIEW BELOW IF THERE IS CONCURRENT OR SEQUENTIAL POLYSUBSTANCE USE:____________________

Considering your use of (NAME THE DRUG / DRUG CLASS SELECTED), in the past 3 months:

E2 a	Have you found that you needed to use more (NAME OF DRUG / DRUG CLASS SELECTED) to get the same effect that you did when you first started taking it?	NO	YES
b	When you reduced or stopped using (NAME OF DRUG / DRUG CLASS SELECTED), did you have withdrawal symptoms (aches, shaking, fever, weakness, diarrhea, nausea, sweating, heart pounding, difficulty sleeping, or feeling agitated, anxious, irritable, or depressed)? Did you use any drug(s) to keep yourself from getting sick (withdrawal symptoms) or so that you would feel better?<br><br>IF **YES** TO EITHER, CODE **YES**.	NO	YES

c Have you often found that when you used (NAME OF DRUG / DRUG CLASS SELECTED), you ended up taking more than you thought you would? NO YES

d Have you tried to reduce or stop taking (NAME OF DRUG / DRUG CLASS SELECTED) but failed? NO YES

e On the days that you used (NAME OF DRUG / DRUG CLASS SELECTED), did you spend substantial time (>2 HOURS), obtaining, using or in recovering from the drug, or thinking about the drug? NO YES

f Did you spend less time working, enjoying hobbies, or being with family or friends because of your drug use? NO YES

g Have you continued to use (NAME OF DRUG / DRUG CLASS SELECTED), even though it caused you health or mental problems? NO YES

ARE **3** OR MORE **E2** ANSWERS CODED **YES**?

SPECIFY DRUG(S): ____________________

* IF YES, SKIP E3 QUESTIONS, CIRCLE N/A IN THE ABUSE BOX FOR THIS SUBSTANCE AND MOVE TO THE NEXT DISORDER.
DEPENDENCE PREEMPTS ABUSE.

**NO**	**YES** *
SUBSTANCE DEPENDENCE **CURRENT**	

E3 a Have you been intoxicated, high, or hungover from (NAME OF DRUG / DRUG CLASS SELECTED) more than once, when you had other responsibilities at school, at work, or at home? Did this cause any problem? NO YES

(CODE **YES** ONLY IF THIS CAUSED PROBLEMS.)

b Have you been high or intoxicated from (NAME OF DRUG / DRUG CLASS SELECTED) more than once in any situation where you were physically at risk (for example, driving a car, riding a motorbike, using machinery, boating, etc.)? NO YES

c Did you have legal problems more than once because of your drug use, for example, an arrest or disorderly conduct? NO YES

d Did you continue to use (NAME OF DRUG / DRUG CLASS SELECTED), even though it caused problems with your family or other people? NO YES

ARE **1** OR MORE **E3** ANSWERS CODED **YES**?

SPECIFY DRUG(S): ____________________

**NO**	**N/A**	**YES**
SUBSTANCE ABUSE **CURRENT**		

SUBSTANCE USE SCREENER

NIDA Quick Screen

**<u>In the past 3 months</u>, how often have you used the following?**	**Never**	**Once or Twice**	**Monthly**	**Weekly**	**Daily or Almost Daily**
**Alcohol**<br>• **For men, 5 or more drinks a day**<br>• **For women, 4 or more drinks a day**					
**Prescription Drugs not Rx, other than Rx**<br>Prescription stimulants (e.g. Ritalin, Adderall), sedatives or sleeping pills (e.g. Valium, Xanax), prescription opioids (e.g. Vicodin, OxyContin)					
**Illegal Drugs**<br>Cannabis, cocaine (e.g. coke, crack), methamphetamine, hallucinogens (e.g. LSD, mushrooms), street opioids (e.g. heroin, opium)					

- If "NO" for all drugs in the Quick Screen, **screening is complete. Move to next module.**
- If "YES" to *one or more days of heavy drinking*, complete **AUDIT (next page)**
- If "YES" to *use of illegal drugs or prescription drugs for non-medical reasons,* complete **NIDA-Modified ASSIST**

NIDA-Modified ASSIST.

**1. In the last 3 months, which of the following substances have you used?**<br><br>*Note: for prescription medications, please report nonmedical use only	**Yes**	**No**
a. Cannabis (marijuana, pot, grass, hash, etc.)		
b. Cocaine (coke, crack, etc.)		
c. Prescription stimulants (Ritalin, Concerta, Dexedrine, Adderall, diet pills, etc.)		
d. Methamphetamine (speed, crystal meth, ice, etc.)		
e. Inhalants (nitrous oxide, glue, gas, paint thinner, etc.)		
f. Sedatives or sleeping pills (Valium, Serepax, Ativan, Xanax, Librium, Rohypnol, GHB, etc.)		
g. Hallucinogens (LSD, acid, mushrooms, PCP, Special K, ecstasy, etc.)		
h. Street opioids (heroin, opium, etc.)		
i. Prescription opioids (fentanyl, oxycodone [OxyContin, Percocet], hydrocodone [Vicodin], methadone, buprenorphine, etc.)		
j. Other—specify:		

- The patient should not indicate "NO" for all drugs above. If they do, remind them that their answers to the Quick Screen indicated they used an illegal or prescription drug for non-medical reasons within the past 3 months.
- If Yes to any of the drugs, proceed to Questions 2-7 of the NIDA-Modified ASSIST

Alcohol Use Disorders Identification Test (AUDIT)						
**Questions**	**0**	**1**	**2**	**3**	**4**	**Score**
1. How often do you have a drink containing alcohol?	Never	Monthly or less	2-4 times a month	2-3 times a week	4 or more times a week	
2. How many drinks containing alcohol do you have on a typical day when you are drinking?	1or 2	3 or 4	5 or 6	7 to 9	10 or more	
3. How often do you have six or more drinks on one occasion?	Never	Less than monthly	Monthly	Weekly	Daily or almost daily	
4. How often during the three months have you found that you were not able to stop drinking once you had started?	Never	Less than monthly	Monthly	Weekly	Daily or almost daily	
5. How often during the last three months have you failed to do what was normally expected of you because of drinking?	Never	Less than monthly	Monthly	Weekly	Daily or almost daily	
6. How often during the last three months have you needed a first drink in the morning to get yourself going after a heavy drinking session?	Never	Less than monthly	Monthly	Weekly	Daily or almost daily	
7. How often during the last 3 mos have you had a feeling of guilt or remorse after drinking?	Never	Less than monthly	Monthly	Weekly	Daily or almost daily	
8. How often during the last 3 mos have you been unable to remember what happened the night before because of your drinking?	Never	Less than monthly	Monthly	Weekly	Daily or almost daily	
9. Have you or someone else been injured because of your drinking?	No		Yes, but not in the last 3 mos		Yes, during the last 3 mos	
10. Has a relative, friend, doctor, or other health care worker been concerned about your drinking or suggested you cut down?	No		Yes, but not in the last 3 mos		Yes, during the last 3 mos	
					**Total**	
0-7 Alcohol Education |8-15 Advice, BI |16-19 Advice, Brief Counseling, Monitoring |20-40 Referral						

NIDA-Modified ASSIST.

2. <u>In the past three months,</u> how often have you used the substances you mentioned (first drug, second drug, etc.)?	Never	Once or Twice	Monthly	Weekly	Daily or Almost Daily
Cannabis (marijuana, pot, grass, hash, etc.)	0	2	3	4	6
Cocaine (coke, crack, etc.)	0	2	3	4	6
Prescription stimulants (Ritalin, Concerta, Dexedrine, Adderall, diet pills, etc.)	0	2	3	4	6
Methamphetamine (speed, crystal meth, ice, etc.)	0	2	3	4	6
Inhalants (nitrous oxide, glue, gas, paint thinner, etc.)	0	2	3	4	6
Sedatives or sleeping pills (Valium, Serepax, Ativan, Xanax, Librium, Rohypnol, GHB, etc.)	0	2	3	4	6
Hallucinogens (LSD, acid, mushrooms, PCP, Special K, ecstasy, etc.)	0	2	3	4	6
Street opioids (heroin, opium, etc.)	0	2	3	4	6
Prescription opioids (fentanyl, oxycodone [OxyContin, Percocet], hydrocodone [Vicodin], methadone, buprenorphine, etc.)	0	2	3	4	6
Other—specify:	0	2	3	4	6

3. <u>In the past three months,</u> how often have you had a strong desire or urge to use (first drug, second drug, etc.)?	Never	Once or Twice	Monthly	Weekly	Daily or Almost Daily
Cannabis (marijuana, pot, grass, hash, etc.)	0	3	4	5	6
Cocaine (coke, crack, etc.)	0	3	4	5	6
Prescription stimulants (Ritalin, Concerta, Dexedrine, Adderall, diet pills, etc.)	0	3	4	5	6
Methamphetamine (speed, crystal meth, ice, etc.)	0	3	4	5	6
Inhalants (nitrous oxide, glue, gas, paint thinner, etc.)	0	3	4	5	6
Sedatives or sleeping pills (Valium, Serepax, Ativan, Xanax, Librium, Rohypnol, GHB, etc.)	0	3	4	5	6
Hallucinogens (LSD, acid, mushrooms, PCP, Special K, ecstasy, etc.)	0	3	4	5	6
Street opioids (heroin, opium, etc.)	0	3	4	5	6
Prescription opioids (fentanyl, oxycodone [OxyContin, Percocet], hydrocodone [Vicodin], methadone, buprenorphine, etc.)	0	3	4	5	6
Other—specify:	0	3	4	5	6

NIDA-Modified ASSIST.

**4. <u>In the past three months,</u> how often has your use of (first drug, second drug, etc.) led to health, social, legal, or financial problems?**	**Never**	**Once or Twice**	**Monthly**	**Weekly**	**Daily or Almost Daily**
Cannabis (marijuana, pot, grass, hash, etc.)	0	4	5	6	7
Cocaine (coke, crack, etc.)	0	4	5	6	7
Prescription stimulants (Ritalin, Concerta, Dexedrine, Adderall, diet pills, etc.)	0	4	5	6	7
Methamphetamine (speed, crystal meth, ice, etc.)	0	4	5	6	7
Inhalants (nitrous oxide, glue, gas, paint thinner, etc.)	0	4	5	6	7
Sedatives or sleeping pills (Valium, Serepax, Ativan, Xanax, Librium, Rohypnol, GHB, etc.)	0	4	5	6	7
Hallucinogens (LSD, acid, mushrooms, PCP, Special K, ecstasy, etc.)	0	4	5	6	7
Street opioids (heroin, opium, etc.)	0	4	5	6	7
Prescription opioids (fentanyl, oxycodone [OxyContin, Percocet], hydrocodone [Vicodin], methadone, buprenorphine, etc.)	0	4	5	6	7
Other—specify:	0	4	5	6	7

**5. <u>In the past three months,</u> how often have you failed to do what was normally expected of you because of your use of (first drug, second drug, etc.)?**	**Never**	**Once or Twice**	**Monthly**	**Weekly**	**Daily or Almost Daily**
Cannabis (marijuana, pot, grass, hash, etc.)	0	5	6	7	8
Cocaine (coke, crack, etc.)	0	5	6	7	8
Prescription stimulants (Ritalin, Concerta, Dexedrine, Adderall, diet pills, etc.)	0	5	6	7	8
Methamphetamine (speed, crystal meth, ice, etc.)	0	5	6	7	8
Inhalants (nitrous oxide, glue, gas, paint thinner, etc.)	0	5	6	7	8
Sedatives or sleeping pills (Valium, Serepax, Ativan, Xanax, Librium, Rohypnol, GHB, etc.)	0	5	6	7	8
Hallucinogens (LSD, acid, mushrooms, PCP, Special K, ecstasy, etc.)	0	5	6	7	8
Street opioids (heroin, opium, etc.)	0	5	6	7	8
Prescription opioids (fentanyl, oxycodone [OxyContin, Percocet], hydrocodone [Vicodin], methadone, buprenorphine, etc.)	0	5	6	7	8
Other—specify:	0	5	6	7	8

NIDA-Modified ASSIST.

6. Has a friend or relative or anyone else ever expressed concern about your use of (first drug, second drug, etc.)?	No, never	Yes, but not in the past 3 months	Yes, in the past 3 months
a. Cannabis (marijuana, pot, grass, hash, etc.)	0	3	6
b. Cocaine (coke, crack, etc.)	0	3	6
c. Prescription stimulants (Ritalin, Concerta, Dexedrine, Adderall, diet pills, etc.)	0	3	6
d. Methamphetamine (speed, crystal meth, ice, etc.)	0	3	6
e. Inhalants (nitrous oxide, glue, gas, paint thinner, etc.)	0	3	6
f. Sedatives or sleeping pills (Valium, Serepax, Ativan, Xanax, Librium, Rohypnol, GHB, etc.)	0	3	6
g. Hallucinogens (LSD, acid, mushrooms, PCP, Special K, ecstasy, etc.)	0	3	6
h. Street opioids (heroin, opium, etc.)	0	3	6
i. Prescription opioids (fentanyl, oxycodone [OxyContin, Percocet], hydrocodone [Vicodin], methadone, buprenorphine, etc.)	0	3	6
j. Other—specify:	0	3	6

7. Have you ever tried and failed to control, cut down or stop using (first drug, second drug, etc.)?	No, never	Yes, but not in the past 3 months	Yes, in the past 3 months
a. Cannabis (marijuana, pot, grass, hash, etc.)	0	3	6
b. Cocaine (coke, crack, etc.)	0	3	6
c. Prescription stimulants (Ritalin, Concerta, Dexedrine, Adderall, diet pills, etc.)	0	3	6
d. Methamphetamine (speed, crystal meth, ice, etc.)	0	3	6
e. Inhalants (nitrous oxide, glue, gas, paint thinner, etc.)	0	3	6
f. Sedatives or sleeping pills (Valium, Serepax, Ativan, Xanax, Librium, Rohypnol, GHB, etc.)	0	3	6
g. Hallucinogens (LSD, acid, mushrooms, PCP, Special K, ecstasy, etc.)	0	3	6
h. Street opioids (heroin, opium, etc.)	0	3	6
i. Prescription opioids (fentanyl, oxycodone [OxyContin, Percocet], hydrocodone [Vicodin], methadone, buprenorphine, etc.)	0	3	6
j. Other—specify:	0	3	6

Tally Sheet for scoring the NIDA-Modified ASSIST (Q's 2-7):

Instructions: For each substance (labeled a-j) add up the scored received for questions 2-7 above. This is the Substance Involvement (SI) score. Do not include the results from Q1 in your SI scores.

**Substance Involvement Score**	**Total (SI SCORE)**
a. Cannabis (marijuana, pot, grass, hash, etc.)	
b. Cocaine (coke, crack, etc.)	
c. Prescription stimulants (Ritalin, Concerta, Dexedrine, Adderall, diet pills, etc.)	
d. Methamphetamine (speed, crystal meth, ice, etc.)	
e. Inhalants (nitrous oxide, glue, gas, paint thinner, etc.)	
f. Sedatives or sleeping pills (Valium, Serepax, Ativan, Xanax, Librium, Rohypnol, GHB, etc.)	
g. Hallucinogens (LSD, acid, mushrooms, PCP, Special K, ecstasy, etc.)	
h. Street opioids (heroin, opium, etc.)	
i. Prescription opioids (fentanyl, oxycodone [OxyContin, Percocet], hydrocodone [Vicodin], methadone, buprenorphine, etc.)	
j. Other—specify:	

Use the resultant Substance Involvement (SI) Score to identify risk level.

To determine patient's risk level based on his or her SI score, see the table below:

Level of risk associated with different Substance Involvement Score ranges for illicit or nonmedical drug use	
0-3	Lower Risk
4-26	Moderate Risk
27+	High Risk

F. PSYCHOTIC DISORDERS AND MOOD DISORDER WITH PSYCHOTIC FEATURES

Now I am going to ask you about unusual experiences that some people have.

ASK FOR AN EXAMPLE OF EACH QUESTION ANSWERED POSITIVELY. CODE **YES** ONLY IF THE EXAMPLES CLEARLY SHOW A DISTORTION OF THOUGHT OR OF PERCEPTION OR IF THEY ARE NOT CULTURALLY APPROPRIATE. BEFORE CODING, INVESTIGATE WHETHER DELUSIONS QUALIFY AS "BIZARRE".

DELUSIONS ARE "BIZARRE" IF: CLEARLY IMPLAUSIBLE, ABSURD, NOT UNDERSTANDABLE, AND CANNOT DERIVE FROM ORDINARY LIFE EXPERIENCE.

		Question			BIZARRE
F1	a	Have you ever believed that people were spying on you, or that someone was plotting against you, or trying to hurt you?<br>**NOTE**: ASK FOR EXAMPLES TO RULE OUT ACTUAL STALKING.	NO	YES	YES
	b	**IF YES OR YES BIZARRE:** do you currently believe these things?	NO	YES	YES<br>➡**F6**
F2	a	Have you ever believed that someone was reading your mind or could hear your thoughts, or that you could actually read someone's mind or hear what another person was thinking?	NO	YES	YES
	b	**IF YES OR YES BIZARRE:** do you currently believe these things?	NO	YES	YES<br>➡**F6**
F3	a	Have you ever believed that someone or some force outside of yourself put thoughts in your mind that were not your own, or made you act in a way that was not your usual self? Have you ever felt that you were possessed?<br>CLINICIAN: ASK FOR EXAMPLES AND DISCOUNT ANY THAT ARE NOT PSYCHOTIC.	NO	YES	YES
	b	**IF YES OR YES BIZARRE:** do you currently believe these things?	NO	YES	YES<br>➡**F6**
F4	a	Have you ever believed that you were being sent special messages through the TV, radio, or newspaper, or that a person you did not personally know was particularly interested in you?	NO	YES	YES
	b	**IF YES OR YES BIZARRE:** do you currently believe these things?	NO	YES	YES<br>➡**F6**
F5	a	Have your relatives or friends ever considered any of your beliefs strange or unusual?<br>INTERVIEWER**:** ASK FOR EXAMPLES. ONLY CODE **YES** IF THE EXAMPLES ARE **CLEARLY** DELUSIONAL IDEAS NOT EXPLORED IN QUESTIONS K1 TO K4, FOR EXAMPLE, SOMATIC OR RELIGIOUS DELUSIONS OR DELUSIONS OF GRANDIOSITY, JEALOUSY, GUILT, RUIN OR DESTITIUTION, ETC.	NO	YES	YES
	b	**IF YES OR YES BIZARRE:** do they currently consider your beliefs strange?	NO	YES	YES
F6	a	Have you ever heard things other people couldn't hear, such as voices?<br>HALLUCINATIONS ARE SCORED "BIZARRE" ONLY IF PATIENT ANSWERS **YES** TO THE FOLLOWING:	NO	YES	
		**IF YES:** Did you hear a voice commenting on your thoughts or behavior or did you hear two or more voices talking to each other?	NO		YES
	b	**IF YES OR YES BIZARRE TO K6a:** have you heard these things in the past month?<br>HALLUCINATIONS ARE SCORED "BIZARRE" ONLY IF PATIENT ANSWERS **YES** TO THE FOLLOWING:<br>Did you hear a voice commenting on your thoughts or behavior or did you hear two or more voices talking to each other?	NO	YES	YES<br>➡**F8b**

F7 a Have you ever had visions when you were awake or have you ever seen things other people couldn't see? — NO YES

CLINICIAN: CHECK TO SEE IF THESE ARE CULTURALLY INAPPROPRIATE.

b **IF YES:** have you seen these things in the past month? — NO YES

CLINICIAN'S JUDGMENT

F8 b IS THE PATIENT CURRENTLY EXHIBITING INCOHERENCE, DISORGANIZED SPEECH, OR MARKED LOOSENING OF ASSOCIATIONS? — NO YES

F9 b IS THE PATIENT CURRENTLY EXHIBITING DISORGANIZED OR CATATONIC BEHAVIOR? — NO YES

F10 b ARE NEGATIVE SYMPTOMS OF SCHIZOPHRENIA, E.G. SIGNIFICANT AFFECTIVE FLATTENING, POVERTY OF SPEECH (ALOGIA) OR AN INABILITY TO INITIATE OR PERSIST IN GOAL-DIRECTED ACTIVITIES (AVOLITION), PROMINENT DURING THE INTERVIEW? — NO YES

F11 a ARE 1 OR MORE « a » QUESTIONS FROM F1a TO F7a CODED **YES OR YES BIZARRE** AND IS EITHER:

MAJOR DEPRESSIVE EPISODE, (CURRENT OR RECURRENT)
OR
MANIC OR HYPOMANIC EPISODE, (CURRENT OR PAST) CODED **YES**? — NO ➔**F13** YES

IF NO, CIRCLE NO IN 'MOOD DISORDER WITH PSYCHOTIC FEATURES' BOXES

b **IF YES:** You told me earlier that you had period(s) when you felt (depressed/high/persistently irritable).

Were the beliefs and experiences you just described restricted **exclusively** to times when you were feeling depressed/high/irritable?

IF PATIENT EVER HAD A PERIOD OF AT LEAST 2 WEEKS OF HAVING PSYCHOTIC SYMPTOMS WHEN THEY WERE NOT DEPRESSED, MANIC, OR HYPOMANIC, CODE NO TO THIS DISORDER AND TO F12

NO	YES
MOOD DISORDER WITH *PSYCHOTIC FEATURES*	
**LIFETIME**	

F12 a ARE 1 OR MORE « b » QUESTIONS FROM F1b TO F7b CODED **YES OR YES BIZARRE** AND IS EITHER:

MAJOR DEPRESSIVE EPISODE, (CURRENT)
OR
MANIC OR HYPOMANIC EPISODE, (CURRENT) CODED **YES**?

IF THE ANSWER IS YES TO LIFETIME OR CURRENT MOOD DISORDER WITH PSYCHOTIC FEATURES, CIRCLE NO TO F13 AND F14 AND MOVE TO THE NEXT MODULE.

NO	YES
MOOD DISORDER WITH *PSYCHOTIC FEATURES*	
**CURRENT**	

F13 ARE 1 OR MORE « b » QUESTIONS FROM F1b TO F6b, CODED **YES BIZARRE**?

OR

ARE 2 OR MORE « b » QUESTIONS FROM F1b TO F10b, CODED **YES** (RATHER THAN **YES BIZARRE**)?

AND DID AT LEAST TWO OF THE PSYCHOTIC SYMPTOMS OCCUR DURING THE SAME 1 MONTH PERIOD?

NO **YES**

PSYCHOTIC DISORDER
CURRENT

F14 IS **F13** CODED **YES**

OR

ARE 1 OR MORE « a » QUESTIONS FROM F1a TO F6a, CODED **YES BIZARRE**?

OR

ARE 2 OR MORE « a » QUESTIONS FROM F1a TO F7a, CODED **YES** (RATHER THAN **YES BIZARRE**)

AND DID AT LEAST TWO OF THE PSYCHOTIC SYMPTOMS OCCUR DURING THE SAME 1 MONTH PERIOD?

NO **YES**

PSYCHOTIC DISORDER
LIFETIME

G. DYSTHYMIA

(➡ MEANS : GO TO THE DIAGNOSTIC BOX, CIRCLE NO, AND MOVE TO THE NEXT MODULE)

G1	Have you felt sad, low or depressed most of the time for the last two years?	➡ NO	YES
G2	Was this period interrupted by your feeling OK for two months or more?	NO	➡ YES
G3	**During this period of feeling depressed most of the time:**		
a	Did your appetite change significantly?	NO	YES
b	Did you have trouble sleeping or sleep excessively?	NO	YES
c	Did you feel tired or without energy?	NO	YES
d	Did you lose your self-confidence?	NO	YES
e	Did you have trouble concentrating or making decisions?	NO	YES
f	Did you feel hopeless?	NO	YES
	ARE **2** OR MORE **G3** ANSWERS CODED **YES**?	➡ NO	YES

G4 Did the symptoms of depression cause you significant distress or impair your ability to function at work, socially, or in some other important way?

**NO**	**YES**
DYSTHYMIA **CURRENT**	

H. PANIC DISORDER

(➡ MEANS : CIRCLE NO IN H5, H6 AND H7)

H1	a	Have you, on more than one occasion, had spells or attacks when you **suddenly** felt anxious, frightened, uncomfortable or uneasy, even in situations where most people would not feel that way?	➡ NO	YES
	b	Did the spells surge to a peak within 10 minutes of starting?	➡ NO	YES
H2		At any time in the past, did any of those spells or attacks come on unexpectedly or occur in an unpredictable or unprovoked manner?	➡ NO	YES
H3		Have you ever had one such attack followed by a month or more of persistent concern about having another attack, or worries about the consequences of the attack or did you make a significant change in your behavior because of the attacks (e.g., shopping only with a companion, not wanting to leave your house, visiting the emergency room repeatedly, or seeing your doctor more frequently because of the symptoms?)	NO	YES
H4		**During the worst spell that you can remember:**		
	a	Did you have skipping, racing or pounding of your heart?	NO	YES
	b	Did you have sweating or clammy hands?	NO	YES
	c	Were you trembling or shaking?	NO	YES
	d	Did you have shortness of breath or difficulty breathing?	NO	YES
	e	Did you have a choking sensation or a lump in your throat?	NO	YES
	f	Did you have chest pain, pressure or discomfort?	NO	YES
	g	Did you have nausea, stomach problems or sudden diarrhea?	NO	YES
	h	Did you feel dizzy, unsteady, lightheaded or faint?	NO	YES
	i	Did things around you feel strange, unreal, detached or unfamiliar, or did you feel outside of or detached from part or all of your body?	NO	YES
	j	Did you fear that you were losing control or going crazy?	NO	YES
	k	Did you fear that you were dying?	NO	YES
	l	Did you have tingling or numbness in parts of your body?	NO	YES
	m	Did you have hot flushes or chills?	NO	YES
H5		ARE BOTH **H3,** AND **4** OR MORE **H4** ANSWERS, CODED **YES**?<br>IF YES TO H5, SKIP TO H7.	NO	YES<br>***PANIC DISORDER LIFETIME***
H6		IF **H5** = **NO,** ARE ANY H4 ANSWERS CODED **YES**?<br>THEN SKIP TO **I1.**	NO	YES<br>***LIMITED SYMPTOM ATTACKS LIFETIME***
H7		In the past month, did you have such attacks repeatedly (2 or more followed by persistent concern about having another attack?	NO	YES<br>***PANIC DISORDER CURRENT***

I. AGORAPHOBIA

I1	Do you feel anxious or uneasy in places or situations where you might have a panic attack or the panic-like symptoms we just spoke about, or where help might not be available or escape might be difficult: like being in a crowd, standing in a line (queue), when you are alone away from home or alone at home, or when crossing a bridge, traveling in a bus, train or car?	NO YES
I2	IF **I1** = **NO**, CIRCLE **NO** IN **I2**.<br>Do you fear these situations so much that you avoid them, or suffer through them, or need a companion to face them?	NO YES<br>***AGORAPHOBIA CURRENT***

IS **I2** (CURRENT AGORAPHOBIA) CODED **NO**

and

IS **H7** (CURRENT PANIC DISORDER) CODED **YES**?

NO **YES**

PANIC DISORDER without Agoraphobia CURRENT

IS **I2** (CURRENT AGORAPHOBIA) CODED **YES**

and

IS **H7** (CURRENT PANIC DISORDER) CODED **YES**?

NO **YES**

PANIC DISORDER with Agoraphobia CURRENT

IS **I2** (CURRENT AGORAPHOBIA) CODED **YES**

and

IS **H5** (PANIC DISORDER LIFETIME) CODED **NO**?

NO **YES**

AGORAPHOBIA, CURRENT without history of Panic Disorder

J. SOCIAL PHOBIA (Social Anxiety Disorder)

(➨ MEANS : GO TO THE DIAGNOSTIC BOX, CIRCLE NO AND MOVE TO THE NEXT MODULE)

		➨	
J1	In the past month, were you fearful or embarrassed being watched, being the focus of attention, or fearful of being humiliated? This includes things like speaking in public, eating in public or with others, writing while someone watches, or being in social situations.	NO	YES
J2	Is this social fear excessive or unreasonable?	➨ NO	YES
J3	Do you fear these social situations so much that you avoid them or suffer through them?	➨ NO	YES

J4 Do these social fears disrupt your normal work or social functioning or cause you significant distress?

NO **YES**

SOCIAL PHOBIA
(Social Anxiety Disorder)
CURRENT

GENERALIZED ❐

NON-GENERALIZED ❐

SUBTYPES

Do you fear and avoid 4 or more social situations?

If YES Generalized social phobia (social anxiety disorder)

If NO Non-generalized social phobia (social anxiety disorder)

ASSESS WHETHER FEARS ARE RESTRICTED TO NON-GENERALIZED ("ONLY 1 OR SEVERAL") SOCIAL SITUATIONS OR EXTEND TO GENERALIZED ("MOST") SOCIAL SITUATIONS.

"MOST" SOCIAL SITUATIONS IS USUALLY OPERATIONALIZED TO MEAN 4 OR MORE SOCIAL SITUATIONS.

EXAMPLES OF SUCH SOCIAL SITUATIONS: INITIATING OR MAINTAINING A CONVERSATION, PARTICIPATING IN SMALL GROUPS, DATING, SPEAKING TO AUTHORITY FIGURES, ATTENDING PARTIES, PUBLIC SPEAKING, EATING IN FRONT OF OTHERS, URINATING IN A PUBLIC WASHROOM, ETC.

K. OBSESSIVE-COMPULSIVE DISORDER

(➡ MEANS: GO TO THE DIAGNOSTIC BOX, CIRCLE NO AND MOVE TO THE NEXT MODULE)

K1	In the past month, have you been bothered by recurrent thoughts, impulses, or images that were unwanted, distasteful, inappropriate, intrusive, or distressing? (For example, the idea that you were dirty, contaminated or had germs, **or** fear of contaminating others, **or** fear of harming someone even though you didn't want to, **or** fearing you would act on some impulse, **or** fear or superstitions that you would be responsible for things going wrong, **or** obsessions with sexual thoughts, images or impulses, **or** hoarding, collecting, **or** religious obsessions.)<br><br>(DO NOT INCLUDE SIMPLY EXCESSIVE WORRIES ABOUT REAL LIFE PROBLEMS. DO NOT INCLUDE OBSESSIONS DIRECTLY RELATED TO EATING DISORDERS, SEXUAL DEVIATIONS, PATHOLOGICAL GAMBLING, OR ALCOHOL OR DRUG ABUSE BECAUSE THE PATIENT MAY DERIVE PLEASURE FROM THE ACTIVITY AND MAY WANT TO RESIST IT ONLY BECAUSE OF ITS NEGATIVE CONSEQUENCES.)	NO YES<br>↓<br>SKIP TO **K4**
K2	Did they keep coming back into your mind even when you tried to ignore or get rid of them?	NO YES<br>↓<br>SKIP TO **K4**
K3	Do you think that these obsessions are the product of your own mind and that they are not imposed from the outside?	NO YES<br>**obsessions**
K4	In the past month, did you do something repeatedly without being able to resist doing it, like washing or cleaning excessively, counting or checking things over and over, or repeating, collecting, arranging things, or other superstitious rituals?	NO YES<br>**compulsions**
	IS **K3** OR **K4** CODED **YES**?	➡<br>NO YES
K5	Did you recognize that either these obsessive thoughts or these compulsive behaviors were excessive or unreasonable?	➡<br>NO YES
K6	Did these obsessive thoughts and/or compulsive behaviors significantly interfere with your normal routine, your work or school, your usual social activities, or relationships, or did they take more than one hour a day?	**NO** **YES**<br>***O.C.D. CURRENT***

L. POSTTRAUMATIC STRESS DISORDER

(➡ MEANS : GO TO THE DIAGNOSTIC BOX, CIRCLE NO, AND MOVE TO THE NEXT MODULE)

L1	Have you ever experienced or witnessed or had to deal with an extremely traumatic event that included actual or threatened death or serious injury to you or someone else?<br><br>EXAMPLES OF TRAUMATIC EVENTS INCLUDE: SERIOUS ACCIDENTS, SEXUAL OR PHYSICAL ASSAULT, A TERRORIST ATTACK, BEING HELD HOSTAGE, KIDNAPPING, FIRE, DISCOVERING A BODY, SUDDEN DEATH OF SOMEONE CLOSE TO YOU, WAR, OR NATURAL DISASTER.	➡ NO	YES
L2	Did you respond with intense fear, helplessness or horror?	➡ NO	YES
L3	During the past month, have you re-experienced the event in a distressing way (such as, dreams, intense recollections, flashbacks or physical reactions)?	➡ NO	YES

L4	**In the past month:**		
a	Have you avoided thinking about or talking about the event ?	NO	YES
b	Have you avoided activities, places or people that remind you of the event?	NO	YES
c	Have you had trouble recalling some important part of what happened?	NO	YES
d	Have you become much less interested in hobbies or social activities?	NO	YES
e	Have you felt detached or estranged from others?	NO	YES
f	Have you noticed that your feelings are numbed?	NO	YES
g	Have you felt that your life will be shortened or that you will die sooner than other people?	NO	YES
	ARE **3** OR MORE **L4** ANSWERS CODED **YES**?	➡ NO	YES
L5	**In the past month:**		
a	Have you had difficulty sleeping?	NO	YES
b	Were you especially irritable or did you have outbursts of anger?	NO	YES
c	Have you had difficulty concentrating?	NO	YES
d	Were you nervous or constantly on your guard?	NO	YES
e	Were you easily startled?	NO	YES
	ARE **2** OR MORE **L5** ANSWERS CODED **YES**?	➡ NO	YES

L6 During the past month, have these problems significantly interfered with your work or social activities, or caused significant distress?

**NO**	**YES**
POSTTRAUMATIC STRESS DISORDER CURRENT	

M. GENERALIZED ANXIETY DISORDER

(➡ MEANS : GO TO THE DIAGNOSTIC BOX, CIRCLE NO, AND MOVE TO THE NEXT MODULE)

M1	a	Have you worried excessively or been anxious about several things over the past 6 months?	➡ NO	YES
	b	Are these worries present most days?	➡ NO	YES
		IS THE PATIENT'S ANXIETY RESTRICTED EXCLUSIVELY TO, OR BETTER EXPLAINED BY, ANY DISORDER PRIOR TO THIS POINT?	NO	➡ YES
M2		Do you find it difficult to control the worries or do they interfere with your ability to focus on what you are doing?	➡ NO	YES
M3		FOR THE FOLLOWING, CODE **NO** IF THE SYMPTOMS ARE CONFINED TO FEATURES OF ANY DISORDER EXPLORED PRIOR TO THIS POINT**.**		
		**When you were anxious over the past 6 months, did you, most of the time:**		
	a	Feel restless, keyed up or on edge?	NO	YES
	b	Feel tense?	NO	YES
	c	Feel tired, weak or exhausted easily?	NO	YES
	d	Have difficulty concentrating or find your mind going blank?	NO	YES
	e	Feel irritable?	NO	YES
	f	Have difficulty sleeping (difficulty falling asleep, waking up in the middle of the night, early morning wakening or sleeping excessively)?	NO	YES

ARE **3** OR MORE **M3** ANSWERS CODED **YES**?

NO **YES**

GENERALIZED ANXIETY DISORDER
CURRENT

N. ANOREXIA NERVOSA

(➡ MEANS : GO TO THE DIAGNOSTIC BOX, CIRCLE NO, AND MOVE TO THE NEXT MODULE)

N1 a	How tall are you?	❑ ft ❑❑ in.	
		❑ ❑ ❑ cm.	
b.	What was your lowest weight in the past 3 months?	❑ ❑ ❑ lbs.	
		❑ ❑ ❑ kgs.	
c	IS PATIENT'S WEIGHT EQUAL TO OR BELOW THE THRESHOLD CORRESPONDING TO HIS / HER HEIGHT? (SEE TABLE BELOW)	➡ NO	YES

In the past 3 months:

N2	In spite of this low weight, have you tried not to gain weight?	➡ NO	YES
N3	Have you intensely feared gaining weight or becoming fat, even though you were underweight?	➡ NO	YES
N4 a	Have you considered yourself too big / fat or that part of your body was too big / fat?	NO	YES
b	Has your body weight or shape greatly influenced how you felt about yourself?	NO	YES
c	Have you thought that your current low body weight was normal or excessive?	NO	YES
N5	ARE 1 OR MORE ITEMS FROM **N4** CODED **YES**?	➡ NO	YES
N6	FOR WOMEN ONLY: During the last 3 months, did you miss all your menstrual periods when they were expected to occur (when you were not pregnant)?	➡ NO	YES

FOR WOMEN: ARE **N5 AND N6** CODED **YES**?

FOR MEN: IS **N5** CODED **YES**?

NO YES

ANOREXIA NERVOSA
CURRENT

HEIGHT / WEIGHT TABLE CORRESPONDING TO A BMI THRESHOLD OF 17.5 KG/M^2

Height/Weight														
ft/in	4'9	4'10	4'11	5'0	5'1	5'2	5'3	5'4	5'5	5'6	5'7	5'8	5'9	5'10
lbs.	81	84	87	89	92	96	99	102	105	108	112	115	118	122
cm	145	147	150	152	155	158	160	163	165	168	170	173	175	178
kgs	37	38	39	41	42	43	45	46	48	49	51	52	54	55

Height/Weight					
ft/in	5'11	6'0	6'1	6'2	6'3
lbs.	125	129	132	136	140
cm	180	183	185	188	191
kgs	57	59	60	62	64

The weight thresholds above are calculated using a body mass index (BMI) equal to or below 17.5 kg/m^2 for the patient's height. This is the threshold guideline below which a person is deemed underweight by the DSM-IV and the ICD-10 Diagnostic Criteria for Research for Anorexia Nervosa.

O. BULIMIA NERVOSA

(➡ MEANS : GO TO THE DIAGNOSTIC BOXES, CIRCLE NO IN ALL DIAGNOSTIC BOXES, AND MOVE TO THE NEXT MODULE)

O1	In the past three months, did you have eating binges or times when you ate a very large amount of food within a 2-hour period?	➡ NO	YES
O2	In the last 3 months, did you have eating binges as often as twice a week?	➡ NO	YES
O3	During these binges, did you feel that your eating was out of control?	➡ NO	YES
O4	Did you do anything to compensate for, or to prevent a weight gain from these binges, like vomiting, fasting, exercising or taking laxatives, enemas, diuretics (fluid pills), or other medications?	➡ NO	YES
O5	Does your body weight or shape greatly influence how you feel about yourself?	➡ NO	YES
O6	DO THE PATIENT'S SYMPTOMS MEET CRITERIA FOR ANOREXIA NERVOSA?	NO ↓ Skip to O8	YES
O7	Do these binges occur only when you are under (_____lbs./kgs.)? INTERVIEWER: WRITE IN THE ABOVE PARENTHESIS THE THRESHOLD WEIGHT FOR THIS PATIENT'S HEIGHT FROM THE HEIGHT / WEIGHT TABLE IN THE ANOREXIA NERVOSA MODULE.	NO	YES

O8 IS **O5** CODED **YES** AND IS EITHER **O6** OR **O7** CODED **NO**?

NO **YES**

BULIMIA NERVOSA
CURRENT

IS **O7** CODED **YES**?

NO **YES**

ANOREXIA NERVOSA
Binge Eating/Purging Type
CURRENT

LiveWell Demographics

LiveWell ID _________________ Date _____________ Interviewer ________

Marital Status: Please pick most current situation (PICK ONLY ONE)

☐ 0 = Never Married ☐ 2 = Married/Domestic ☐ 4 = Separated

☐ 1 = Divorced ☐ 3 = Partner Widowed ☐ 5 = Living with Significant Other

Number of people in household (including self) ☐☐

Number of children living in the household:
(please write in the **number** of children in each category)

☐ 0 = Under 5 ☐ 2 = Ages 11-19

☐ 1 = Ages 5-10 ☐ 3 = Adult Children

Highest Level of Education (Please select only one)

☐ 0 = 8th Grade or Lower ☐ 3 = Some College ☐ 6 = Master's Degree

☐ 1 = Some High School ☐ 4 = 2-yr College (Associates) ☐ 7 = Doctoral Degree

☐ 2 = Completed High School / GED ☐ 5 = 4-yr College (BA, BS) ☐ 8 = Professional Degree (MD, JD)

Current Employment Status *What is your **main** source of income?*

☐ 0 = Employed ☐ 1 = Unemployed ☐ 2= On Disability Insurance ☐ 3 = Retired

☐ 4 = Temporarily Laid Off (Sick Leave, Maternity Leave) ☐ 5 = Student ☐ 6= Other_________________

Occupation (current) ____

OR

Occupation (at last job)____

If employed: Is the job seasonal?

☐ 0 = Seasonal

☐ 1 = Not Seasonal

☐ N/A

Number of hours per week spent working currently

☐☐

Number of hours per week spent volunteering or doing other non-paid work

☐☐

Number of hours per week spent caring for dependent adults

☐☐

Current total yearly HOUSEHOLD Gross income (pre-tax):
(include the income of all household members, including yourself)

☐☐☐,☐☐☐ ☐ Unknown

Mobile Phone Information

Do you have a computer with internet access? ☐ Yes ☐ No

Do you have wireless at home? ☐ Yes ☐ No

Do you have a Gmail account? ☐ Yes ☐ No

Do you have a mobile phone? ☐ Yes ☐ No

If yes:

How much of the day do you have the phone with you?

☐ Not any part of the day ☐ Less than half the day ☐ More than half the day ☐ All day

Current type of mobile phone

☐ Android ☐ iPhone ☐ Other: ____

Current service provider

☐ T-Mobile ☐ Other: ____

(If Android and/or T-Mobile) Plan details:____

____

____

____

LiveWell Enrollment NDA Demographics

Name: ________________ Date: ________________

1. Please provide the following information as it **appears on your birth certificate**:

First name: ____________________

Middle name: ____________________

Last Name: ____________________

Date of birth: ________ / ________ / ________
mm / *dd* / *yyyy*

Sex: Male / Female

City/municipality of birth: ____________________

2. Please provide the following information regarding your place of employment. If you are not employed but are a student, volunteer, or maintain responsibilities that require you to spend much of your time regularly outside of your household, use that address.

Address Type: Work / School / Volunteer / Other: ____________________

Street Address: ____________________

City: ____________________

State: ____________________

Zip Code: ____________________

3. Please provide your home address.

Street Address: ____________________

City: ____________________

State: ____________________

Zip Code: ____________________

LW-ADE: LiveWell Affective Disorders Evaluation		
ID:____________	Date:_____________	Interviewer:_____________

Mania:

A. Have you ever had a time when most of the day, nearly every day for as long as a week

	Yes	No	Duration
You were feeling so good that other people thought you were not your normal self?	___	___	_____
You were feeling so irritable that you shouted at people, got in fights, or got into trouble. You found yourself yelling at people you didn't really know?	___	___	_____
You were so hyper you got into trouble?	___	___	_____
You felt like you could do much more than ordinary or engaged in more goal directed activities than usual?	___	___	_____
You were physically or mentally restless or had more energy than usual? Too much energy?	___	___	_____

For each question above, how many days like this nearly every day for most of the day?

Did anyone say you were manic? ___ ___

Who? ____________________

B. Other Causes (Optional – Assess mania not due to bipolar disorder)

Are the manic symptoms due to direct physiological effects of a substance or a general medical condition?

Taking an antidepressant ____ Yes ____ No

Using alcohol ____ Yes ____ No

Using substances ____ Yes ____ No

Medical problem ____ Yes ____ No

____

____

____

LW-ADE: LiveWell Affective Disorders Evaluation		
ID:____________	Date:_____________	Interviewer:_____________

C. History of elevated/irritable mood episodes

1. How many times have you been like this **for 1 wk or more (or needed to be hospitalized)**?

Number of episodes: 0 1 2 3 4-5 6-9 10-20 21-50 Too many to count Indeterminate

From phone screen: ______

2. Were these episodes ever accompanied by

a. Paranoia ____ Yes ____ No What ________________________________
b. Delusions: ____ Yes ____ No What ________________________________
c. Hallucinations: ____ Yes ____ No What ________________________________

Season Key

Fall: Sep, Oct, Nov	Spring: Mar, Apr, May
Winter: Dec, Jan, Feb	Summer: Jun, Jul, Aug

3. When was the first time you were like this **for 1 week or more (or needed to be hospitalized)**?

a. Age: ______ b. Year: ______ c. Season: ____________

d. Date Onset*: _________ e. Duration (in weeks): __________

____
____
____

4. When was your most recent episode of mania?

a. Age: ______ b. Year: ______ c. Season: ____________

d. Date Onset*: _________ e. Duration (in weeks): __________

____
____
____

From phone screen:
Date of onset: ___________ Age:____________ Duration: ___________

* If participant can't recall exact date or month of onset, try to obtain season and year

D. For the most severe episode of elevated mood:

Season Key
Fall: Sep, Oct, Nov   Spring: Mar, Apr, May
Winter: Dec, Jan, Feb   Summer: Jun, Jul, Aug

6. When was the worst time you were like this **for 1 week or more (or needed to be hospitalized)**?

a. Age: ______   b. Year: ______   c. Season: ____________

d. Date Onset*: _________   e. Duration (in weeks): __________

* If participant can't recall exact date or month of onset, indicate season and year.

____

____

____

From phone screen:
Date of onset: ___________   Age:_____________   Duration: ___________

Optional

During this most severe episode, were there ***any times*** when your mood was:

❑ Euphoric ☐ Expansive ☐ Irritable ☐ Dysphoric

Was it really markedly different than normal in relation to events in your life or your usual self?

❑ Yes ☐ No

During the worst week of this most severe episode, did you: (Y/N)

___ have work/school/volunteer/housework/child-family care problems

___ have family/social relationship problems

___ engage in any violence   ___ have legal problems

7. Were you admitted to the hospital during this episode due to symptoms of mania? ☐ Yes ☐ No

____

____

____

E. Criteria for scoring the most severe episode of elevated mood:

8a. **CMF Mania 1a. Elevated/Expansive, Severity (DSM A):** Distinct period of elevated or expansive mood during the same 1 week period

During the worst week or two of this episode, did you feel so good that people thought you were not your normal self? Felt very good, too cheerful, high, optimistic attitude out of proportion to circumstances?

			Comments
**0**	**No mood elevation or expansiveness.**		
	**Frequency**	**Intensity**	
**+1/4**	Any	Any elevation/expansiveness not clearly related to events	
**+1/2**			
**+1**	≥4/7 days or hospitalized	Feeling so good high, excited or optimistic people thought not normal self<br>Abnormally persistently elevated, expansive, euphoric, excessively cheerful, high, "on top of the world"	
**+1.5**		Clearly elated, exalted expressions.	
**+2**	≥4/7 days	Psychotic features such as belief in divine powers	

8b. **CMF Mania 1b. Elevated/Expansive, DSM Consecutive Days:**
How many consecutive days with elevated/expansive mood of intensity >= 1? _____ # of days (0-7 days)

9a. **CMF Mania 2a. Irritable, Severity (DSM B):** Distinct period of irritable mood during the same 1 week period

During the worst week or two of this episode, were you so irritable that you shouted at people or started fights or arguments?

			Comments
**0**	**No irritability**		
	**Frequency**	**Intensity**	
**+1/4**	Any	Any irritability not clearly related to events	
**+1/2**			
**+1**	≥4/7 days or hospitalized	Clearly abnormal irritability with behavioral manifestations (e.g. starts fights/arguments)	
**+1.5**			
**+2**	≥4/7 days	Psychotic features such as paranoia	

9b. **CMF Mania 2b. Irritable, DSM Consecutive Days:**
How many consecutive days with irritable mood of intensity >= 1? _____ # of days (0-7 days)

10a. **CMF Mania 8c1. Increased energy (DSM4 A, DSM5 C):** Increased energy during the same 1 week period

During the worst week or two of this episode, did you feel so hyper that people thought you were not your normal self, or were you so hyper you got in trouble? Did you have more energy than usual to do things? Did it seem like too much energy?

Unusually energetic more active than usual self without expected fatigue.			**Comments**
**0**	**No abnormally increased energy**		
	**Frequency**	**Intensity**	
**+1/4**	Any	Slightly more energetic	
**+1/2**		Little change in activity level but less fatigued than usual	
**+1**	≥4/7 days or hospitalized	Hyper and not their normal self.	
		So hyper got into trouble or observed manic behavior.	
		Unusual behavior but didn't get into trouble due to not observed	
		Somewhat more active than usual with little or no fatigue	
**+1.5**			
**+2**	≥4/7 days	Much more active than usual, unusually active all day long with little or no fatigue	

10b. **CMF Mania 8c5. Increased Energy, DSM Consecutive Days:**
How many consecutive days with increased energy of intensity >= 1? _____ # of days (0-7 days)

11a. **CMF Mania 8a1. Goal Directed Activity, Severity (6a, DSM5 C):** Increase in goal directed activity during the same 1 week period

During the worst week or two of this episode, did you have an increase in goal directed activity either socially, sexually, at work or school? Were you so active that people worried about you taking on so much? Did you find you were so active that you really didn't get much done?

Plans, projects, purposeful activities			**Comments**
**0**	**No increase in goal directed behavior**		
	**Frequency**	**Intensity**	
**+1/4**	Any	Initiated one or more new projects	
**+1/2**			
**+1**	≥4/7 days or hospitalized	Multiple creative, self-improvement, home projects w/out external requirements	
		New projects require commitment of > 8hrs/wk or > 5% of income	
**+1.5**			
**+2**	≥4/7 days	Work effort > 10hrs/day or work after 9 pm	

11b. **CMF Mania 8a2. Goal Directed Activity, DSM Consecutive Days:**
How many consecutive days with increased goal directed activity of intensity >= 1? _____ # of days (0-7 days)

12a. **CMF Mania 8b1. Psychomotor agitation (6b, DSM5 C):** Increase in psychomotor agitation during the same 1 week period

During the worst week or two of this episode, were there times you were so fidgety or agitated it was hard for you to stay still? Were you physically or mentally restless?

Fidgetiness, playing with hands, hair, etc. Moving about, can't sit still. Purposeless non-goal-directed activity.			Comments
**0**	**No evidence of motor agitation**		
	**Frequency**	**Intensity**	
**+ 1/4**	Any	Restless, fidgets	
**+ 1/2**			
**+1**	≥4/7 days<br>or hospitalized	Difficulty remaining still or purposeless movement observed by others	
**+1.5**			
**+2**	≥4/7 days	Pacing, unable to sit still when necessary	

12b. **CMF Mania 8b2. Psychomotor Agitation, DSM Consecutive Days:**
How many consecutive days with psychomotor agitation of intensity >= 1? _____ # of days (0-7 days)

13. **CMF Mania 4. Need for Sleep (2):** Decreased need for sleep during the same 1 week period

During the worst week or two of this episode, were there nights when you got less sleep than usual and found you didn't really miss it? (Do not count simple insomnia)

Amount of sleep, subjective need for sleep, ability to function.			Comments
**0**	**Usual need for sleep**		
	**Frequency**	**Intensity**	
**- 1/4**	Any	Mild ↓; no impact on function	
**- 1/2**			
**-1**	≥4/7 days	Sleep reduced ≥ 1.5 hrs without impact on next day function	
**-1.5**			
**-2**	≥4/7 days	Sleep reduced by >5hrs from usual or sleeping <2hrs/day	

14. **CMF Mania 3. Self-Esteem/Grandiosity (1):** Inflated self-esteem or grandiosity during the same 1 week period

During the worst week or two of this episode, were there times when you were feeling more self-confident than usual? Were there times when you were feeling more special, more talented, more attractive or smarter than usual? Were there any times when your thoughts were grandiose?

Inflated self-confidence, feels more attractive, more talented, able to do more than usual.			**Comments**
**0**	**No increase in self-esteem**		
	**Frequency**	**Intensity**	
**+1/4**	Any	Some exaggerated sense of abilities	
**+1/2**			
**+1**	≥ 4/7 days	Clearly inflated estimate of capabilities	
		Actual performance may be increased but assessment excessively positive	
**+1.5**		Grossly excessive ideas of worth or abilities	
**+2**	≥4/7 days	Delusional	

15. **CMF Mania 5. Talking (3):** More talkative than usual or pressure to keep talking during the same 1 week period

During the worst week or two of this episode, were there times when you were more talkative than usual, or you found you said more than you intended? Were there times that you spoke much faster than usual?

Amount and rate of speech.			**Comments**
**0**	**Normal rate and quantity of speech**		
	**Frequency**	**Intensity**	
**+1/4**	Any	Others note talkative, not out of character/bothersome	
**+1/2**			
**+1**	≥4/7 days	Others complain about excessive talking, uncharacteristically cutting others off	
		Pressured speech (described or observed)	
		Conversation seeking	
		Communication reveals more than intended	
**+1.5**			
**+2**	≥4/7 days	Hard for others to get a word in	
		Virtually incessant talking	

16. **CMF Mania 6. FOI/Racing Thoughts (4):** Flight of ideas or subjective experience that thoughts are racing during the same 1 week period

During the worst week or two of this episode, did you find that you had more ideas than usual? Were there times when your thoughts seemed to be racing through your head?

Racing thoughts, ↑ in train of productive, novel, unrelated ideation.			Comments
**0**	**No racing thoughts**		
	**Frequency**	**Intensity**	
**+1/4**	Any	Mild thinking fast	
**+1/2**			
**+1**	≥4/7 days	Ideas race, come tumbling out	
		Rapid train of thoughts	
**+1.5**			
**+2**	≥4/7 days	Speech cannot keep up w/ pressured thoughts.	

17. **CMF Mania 7. Distractibility (5):** Distractibility during the same 1 week period

During the worst week or two of this episode, did you find you were easily distracted? Was it hard to focus in the first place or attention easily draw away but other thoughts or things going on around you?

Unable to maintain focus of attention, easily distracted by external or internal stimuli.			Comments
**0**	**No evidence of distractibility.**		
	**Frequency**	**Intensity**	
**+1/4**	Any	Generally able to maintain focus	
**+1/2**			
**+1**	≥4/7 days	Decreased ability to complete tasks due to distractibility	
		Able to complete tasks but requires great effort	
**+1.5**			
**+2**	≥4/7 days	Obvious in most conversations	
		Can't stay on topic to complete most sentences thoughts	

18. **CMF Mania 9. High Risk Behavior (7):** Excessive involvement in pleasurable activities that have a high potential for painful consequences during the same 1 week period

During the worst week or two of this episode, did you do anything that was unusual for you or that other people might think was excessive, foolish, or risky? Did you do anything which would have caused a problem if you were caught?

Excessive, foolish, risky activities that could have serious consequences for self/others (whether or not caught)			**Comments**
**0**	**No risk taking.**		
	**Frequency**	**Intensity**	
**+1/4**	Any	Mild risk taking/increased pleasure seeking	
**+1/2**			
**+1**	≥4/7 days	Exhibits behavior others would recognize as foolish/risky/excessive	
		Gambling, sex, investments, reckless driving with negative consequences	
		Definitely hazardous physical, financial or social behavior	
**+1.5**			
**+2**	≥4/7 days	Extremely hazardous physical, financial or social behavior	

Mania 10. Hospitalization: Hospitalized due to symptoms of mania?

No (0) _____ Yes (1) _____

Mania 11. Psychosis: Psychotic or delusional symptoms of mania?

No (0) _____ Yes (1) _____

Mania 12. Impairment: Mania - Manic symptoms severe enough to cause moderate impairment in occupational functioning or in usual social activities or relationships with others. **Hypomania -** Manic symptoms not severe enough to cause moderate impairment in social or occupational functioning. However, an unequivocal change in functioning uncharacteristic of person when not symptomatic is present and the disturbance in mood and change in functioning are observable by others.

Have your manic symptoms caused any major changes or problems in your

- *eating, or hygiene and grooming?*
- *interactions with other such as associating with strangers, or conflicts?*
- *sexual activity, spending habits, or substance use?*
- *family responsibilities?*
- *work, school, or volunteer responsibilities?*

Have your manic symptoms resulted in any legal problems?
Have your manic symptoms resulted in any physical altercations or violence?

			Comments
**0**	**No significant distress or impairment**		
	**Frequency**	**Intensity**	
**+1/4**	Any		
**+1/2**			
**+1**	≥4/7 days	Poorly groomed, moderately disheveled	
		Conflicts with others, associating with strangers	
		Decreased function in role at home or work	
		Hazardous, physical, financial or social behavior	
**+1.5**			
**+2**	≥4/7 days	Completely unkempt, disheveled, bizarre garb	
		Inability to function in role at home or work	
		Extremely hazardous physical, financial or social behavior	
		Violence, legal problems	

Symptoms possibly consistent with marked or severe impairment criteria for PSR: Elevated/Expansive, Irritable, Self-Esteem/Grandiosity, Psychomotor agitation, Distractible, High Risk Behavior.

Mania 13. Other Causes: Are the manic symptoms due to direct physiological effects of a substance or a general medical condition.

No (0) _____ Yes (1) _____

Depression:

A. Have you ever had a time when most of the day, nearly every day for as long as two weeks

	Yes	No	Duration
You were feeling down or depressed?	___	___	_____
You lost interest or pleasure in things you usually enjoy?	___	___	_____

For each question, how many days did you experience this nearly every day for most of the day?

____

____

____

B. Other Causes (Optional – Assess depression not due to bipolar disorder)

Are the depressive symptoms due to direct physiological effects of a substance or a general medical condition?

Using alcohol ____ Yes ____ No

Using substances ____ Yes ____ No

Medical problem ____ Yes ____ No

____

____

____

C. History of depressed mood episodes

21. How many times have you been like this **for as long as 2 weeks**?

Number of episodes (circle one): 0 1 2 3 4-5 6-9 10-20 21-50 Too many to count Indeterminate

From phone screen: ______

22. Were these episodes ever accompanied by

a. Paranoia ____ Yes ____ No What ________________________________
b. Delusions: ____ Yes ____ No What ________________________________
c. Hallucinations: ____ Yes ____ No What ________________________________

Season Key

Fall: Sep, Oct, Nov	Spring: Mar, Apr, May
Winter: Dec, Jan, Feb	Summer: Jun, Jul, Aug

23. When was the first time your mood was like that for 2 weeks or more?

a. Age: ______ b. Year: ______ c. Season: ____________

d. Date Onset*: _________ e. Duration (in weeks): __________

____
____
____

24. When was your most recent episode of depression?

a. Age: ______ b. Year: ______ c. Season: ____________

d. Date Onset*: _________ e. Duration (in weeks): __________

____
____
____

From phone screen:
Date of onset: ___________ Age:_____________ Duration: ____________

* If participant can't recall exact date or month of onset, try to obtain season and year

D. For the most severe depressive episode:

Season Key
Fall: Sep, Oct, Nov   Spring: Mar, Apr, May
Winter: Dec, Jan, Feb   Summer: Jun, Jul, Aug

26. When was the worst time you were like this **for 2 weeks or more?**

a. Age: ______   b. Year: ______   c. Season: ____________

d. Date Onset*: _________   e. Duration (in weeks): __________

____

____

____

From phone screen:
Date of onset: ___________   Age:_____________   Duration: ____________

* If participant can't recall exact date or month of onset, indicate season and year.

Optional

During this most severe episode, were there ***any times*** when your mood was:

☐ Euphoric ☐ Expansive ☐ Irritable ☐ Dysphoric

Was it really markedly different than normal in relation to events in your life or your usual self?

☐ Yes ☐ No

During the worst week of this most severe episode, did you: (Y/N)

___ have work/school/volunteer/housework/child-family care problems

___ have family/social relationship problems

___ engage in any violence   ___ have legal problems

27. Were you admitted to the hospital during this episode due to symptoms of depression? ☐ Yes ☐ No

____

____

____

E. Criteria for scoring the most severe episode of depressed mood:

28a. **CMF Depression 1a. Depressed Mood, Severity (DSM A)**: Depressed mood most of the day, nearly every day during the same 2 week period

During the worst two weeks of this episode, did you feel down or depressed?

Persistently feels “depressed”, “sad”, “down”, “blue” or equivalent dysphoria.			**Comments**
**0**	**Not depressed**		
	**Frequency**	**Intensity**	
**+1/4**	Any	Any dysphoria	
**+1/2**			
**+1**	≥10/14 days	Depressed dysphoric mood<br>Most of the day	
**+1.5**			
**+2**	≥10/14 days	Constant unremitting intense dysphoria	

29a. **CMF Depression 2a. Decreased Interest, Severity:** Markedly diminished interest or pleasure in all or almost all activities most of the day, nearly every day during the same 2 week period

During the worst two weeks of this episode, were you able to enjoy pleasant things that happened?

Loss of motivation or connectedness with others, loss of interest in or diminished capacity for enjoyment of pleasurable activities, push self to work or activities.			**Comments**
**0**	**Enjoys activities as usual**		
	**Frequency**	**Intensity**	
**-1/4**	Any	Any decreased interest	
**-1/2**			
**-1**	≥10/14 days	Loss of interest or enjoyment in most things<br>Disinterest decreased motivation but able to enjoy some activities under favorable conditions<br>Most of the day	
**-1.5**			
**-2**	≥10/14 days	Much less interested, Emotionally constricted<br>Can’t cry, no response to favorable stimuli<br>Most of the day	

30. **CMF Depression 4. Insomnia/Hypersomnia:** Insomnia or hypersomnia nearly every day during the same 2 week period

What was your sleep like during the worst two weeks of this episode?

Disturbance in quality or amount of sleep.			**Comments**
**0**	**Sleeping normally every night**		
	**Frequency**	**Intensity**	
**+1/4 or -1/4**	Any	Any sleep disturbance	
**+1/2 or -1/2**			
**+1 or -1**	≥10/14 days	≥ 1 hr/d deviation from normal	
**+1.5 or -1.5**			
**+2 or -2**	≥10/14 days	Sleep increase or decrease 50% above normal	
**If disturbance in both directions, select based on whichever predominates.**			

Range: Min _____ hrs, Max _____ hrs

EBT: *Did you have to go to bed earlier than usual?* _____ (Y/N)

DFA: *Did you have difficulty falling asleep?* _____

MCA: *Were you waking up in the middle of the night?* _____

EMA: *Did you experience early morning awakenings?* _____

DGOOB: *Did you have difficulty getting out of bed in the morning?* _____

Naps: *Were you napping*? _____

31. **CMF Depression 6. Fatigue/Energy:** Fatigue or loss of energy nearly every day during the same 2 week period

How was your energy level during the worst two weeks of this episode? Were there things which you should have done and didn't because you didn't have enough energy or were simply too tired? For example?

Fatigue, decreased energy, feels tired or tires easily.			**Comments**
**0**	**Usual energy level.**		
	**Frequency**	**Intensity**	
**-1/4**	Any	Carries out all activities<br>Occasionally tired, some tasks are harder<br>More drained than usual	
**-1/2**			
**-1**	≥10/14 days	Interferes with some activities at work, home, socially (Not due to a lack of motivation)	
**-1.5**			
**-2**	≥10/14 days	Lethargic, Stays in bed	

32. **CMF Depression 5b. Psychomotor retardation:** Psychomotor retardation nearly every day during the same 2 week period

Were there times you were moving or thinking more slowly than usual? If I had been with you, would I have noticed something was wrong?

Slowness of thought and speech, impaired ability to concentrate, decreased motor activity.			**Comments**
**0**	**No evidence of motor, speech, or cognitive slowing.**		
	**Frequency**	**Intensity**	
**+1/4**	Any	Subjective slowing of thoughts, speech, or movement or rare objective evidence of retardation	
**+1/2**			
**+1**	≥10/14 days	Slowness thought or movement observable by others<br>Increased speech latency	
**+1.5**			
**+2**	≥10/14 days	Apparent on interview	

33. **CMF Depression 5a. Psychomotor agitation:** Psychomotor agitation nearly every day during the same 2 week period

Were there times you were so fidgety or agitated it was hard for you to stay still?

Fidgetiness, playing with hands, hair, etc. Moving about, can't sit still, purposeless activity.			**Comments**
**0**	**No evidence of motor agitation.**		
	**Frequency**	**Intensity**	
**+1/4**	Any	Restlessness, fidgeting, purposeless movement, pacing	
**+1/2**			
**+1**	≥10/14 days	Difficulty remaining still or purposeless movement observed by others	
**+1.5**			
**+2**	≥10/14 days	Pacing, unable to sit still when necessary	

34. **CMF Depression 3. Appetite/Weight:** Significant weight loss when not dieting or weight gain or decrease or increase in appetite nearly every day during the same 2 week period

How was your appetite?

Disturbance of appetite, ↑/↓ from normal.			Comments
**0**	**Normal appetite/weight**		
	**Frequency**	**Intensity**	
**+1/4 or -1/4**	Any	Mild or rare ↑/↓ in appetite	
**+1/2 or -1/2**			
**+1 or -1**	≥10/14 days	Reduced or increased consumption about 25%	
		Needs encouragement to eat	
		Craving food or seeks snacks in addition to usual meals	
**+1.5 or -1.5**			
**+2 or -2**	≥10/14 days	Weight loss or gain ≥ 5% body in 2 weeks	
		Decrease or increase 50% of normal consumption	
**Count all days with appetite disturbance and use +/- to indicate predominant direction**			

35. **CMF Depression 8. Concentration/Indecisiveness:** Diminished ability to think or concentrate, or indecisiveness, nearly every day during the same 2 week period

How was your concentration?

Inability to concentrate, inability to focus on a task, difficulty making decisions.			Comments
**0**	**Normal concentration.**		
	**Frequency**	**Intensity**	
**-1/4**	Any	Rare or limited to unpleasant, very difficult tasks	
**-1/2**			
**-1**	≥10/14 days	Difficult to read or collect thoughts in conversation	
		Difficult to function in role at home/work	
**-1.5**			
**-2**	≥10/14 days	Clear cognitive impairment during casual interaction	
		Unable to function in role at home/work	

36. **CMF Depression 7a. Guilt:** Feelings of excessive or inappropriate guilt nearly every day during the same 2 week period

Were there times you were down on yourself? Did you feel as if you were a bad person or that you deserved to suffer? (Not limited inability function due to illness)

Self-reproach, feels let people down, present illness is a punishment. Delusions of guilt, hears accusatory voices, threatening visual hallucinations.			**Comments**
**0**	**No excessive self-blame or guilty preoccupation.**		
	**Frequency**	**Intensity**	
**+1/4**	Any	Mild, rare self-deprecatory thoughts	
**+1/2**			
**+1**	≥10/14 days	Self-deprecatory thoughts (Not limited inability fx due illness)<br>Guilt or ruminations over past errors/sinful deeds	
**+1.5**			
**+2**	≥10/14 days	Self-deprecatory thoughts (Not limited inability fx due illness)<br>Guilt or ruminations over past errors/sinful deeds<br>Most of the day	

37. **CMF Depression 7b. Worthlessness:** Feelings of worthlessness nearly every day during the same 2 week period

Was your self-esteem or self-confidence down compared to usual?

Feels inferior, defective, incompetent, inadequate			**Comments**
**0**	**Normal self-esteem/self-confidence**		
	**Frequency**	**Intensity**	
**-1/4**	Any	Felt mildly down on self, lacking self-confidence	
**-1/2**			
**-1**	≥10/14 days	Feels inferior to most others<br>Stops work or social activities due to expectation of failure	
**-1.5**		Worthlessness	
**-2**	≥10/14 days	Delusional	

38. **CMF Depression 9. Suicidal Ideation (SI):** Recurrent thoughts of death, recurrent suicidal ideation without a specific plan, or a suicide attempt or a specific plan for committing suicide

During the worst two weeks of this episode, were there times you were feeling so bad that you felt life was not worth living? What about actually thinking about suicide or harming yourself?

Weary of life, would be better off dead, morbid preoccupation, thoughts of harming self, plans for self-destruction, urge to end life			**Comments**
**0**	**No SI.**		
	**Frequency**	**Intensity**	
**+ 1/4**	Rare	Fleeting LNWL, Fleeting passive SI	
**+ 1/2**	Several days	Fleeting LNWL or fleeting passive or active SI	
		Persistent periods of passive SI	
**+1**	≥ 10/14 days	Most of the day with LNWL	
		Persistent periods of passive SI	
	Several days	Brief active SI	
**+1.5**	> 1 day	Persistent periods of active SI	
**+2**	≥ 10/14 days	Most of the day with active SI	
	Any	Active SI with intent, plan, or action	
		Active SI unable to control thoughts impulses	
**IF CMF #9 SI SYMPTOM SCORE ≥ 1, GO TO SI PROTOCOL**			

LNWL: Life Not Worth Living.

Passive SI: Thoughts of death **without** plan for self-destruction, no action or urge to act.

Active SI: Suicidal thoughts with plan for self-destruction, but no action or urge to act.

Fleeting: < 1 minute

Brief: 1-15 minutes

Persistent: ≥ 15 minutes

Depression 10. Impairment: The depressive symptoms cause clinically significant distress or impairment in social, occupational, or other important areas of functioning.

Have your depressive symptoms caused any major changes or problems in your

- sleep, eating, or hygiene and grooming?
- interactions with other such as withdrawal or conflicts?
- family responsibilities?
- work, school, or volunteer responsibilities?

			Comments
**0**	**No significant distress or impairment**		
	**Frequency**	**Intensity**	
**+1/4**	Any		
**+1/2**			
**+1**	≥10/14 days	Poorly groomed, moderately disheveled	
		Conflicts with others	
		Decreased function in role at home or work	
		Social withdrawal	
**+1.5**			
**+2**	≥10/14 days	Completely unkempt, disheveled	
		Inability to function in role at home or work	
		Social isolation	
		Life-threatening behaviors	

Symptoms possibly consistent with marked or severe impairment: Fatigue/Energy, Psychomotor retardation/agitation, Concentration/Indecisiveness, Worthlessness, Suicidal ideation.

Depression 11. Other Causes: Are the depressive symptoms due to direct physiological effects of a substance, a general medical condition, or are better accounted for by bereavement.

No (0) _____ Yes (1) _____

Psychiatric and General Medical History:

A. Psychiatric Co-Moribidity

[Screen for current co-mobid axis 1. Use MINI results as guide and follow up as needed to clarify/verify]

Co-Morbid Axis I Diagnoses				
	Current		MINI	
	No	Yes		Type/Comments
41. **Dysthymia** (past 2 yrs.)				
42. **Panic Disorder** (past mo.)				
43. **Agoraphobia** (past mo.)				
44. **Social Phobia** (past mo.)				
45. **OCD** (past mo.)				
46. **PTSD** (past mo.)				
47. **GAD** (past 6 mos.)				
48a. **Anorexia** (past 3 mos.)				48b. **Lifetime: Y N**
49a. **Bulimia** (past 3 mos.)				49b. **Lifetime: Y N**

Ever weighed much less than people thought you ought to weigh?

1) Refusal maintain body weight at or above minimal normal
2) Intense fear gaining weight becoming fat despite underweight
3) Disturbance way body weight/shape experienced
   Amenorrhea
   Restrict, vomit, laxitives, diuretics, enemas

Often had times when eating out of control?

1) Recurrent episodes binge eating – sense lack control, eating discrete period definitely large amount
2) Recurrent inappropriate compensatory behavior (vomiting, laxatives, diuretics, enemas)
3) Binge and compensatory 2x wk 3 mos
4) Self-eval unduly influenced body weight/shape

B. **Psychosis** (Optional – Rule out schizophrenia and schizoaffective)

If any history of psychotic symptoms (delusions, hallucinations, thought disorder, negative symptoms, bizarre behavior, catatonia, gross disorganization), determine if present in absence of mood symptoms, substance use, or medial problem?

Psychotic symptoms in **absence** of severe mood symptoms? ____ Yes ____ No

Psychotic symptoms in **absence** of intoxication? ____ Yes ____ No

Psychotic symptoms in absence of medical problem? ____ Yes ____ No

Are mood symptoms brief relative duration of psychotic symptoms? ____ Yes ____ No

Do psychotic symptoms persist during one month period? ____ Yes ____ No

Continuous signs psychotic symptoms at least 6 months? ____ Yes ____ No

C. PSYCHOACTIVE SUBSTANCE USE HISTORY

[Review/verify/clarify information from MINI, AUDIT, NIDA Quick Screen]

[Screen for lifetime substance use disorders and treatment]

	Lifetime use	Age Peak Use	HO SUD	Age Onset	HO SUD Tx	Age Treatment
50. EtOH	Y N		Y N		Y N	
51. Cannabis	Y N		Y N		Y N	
52. Cocaine	Y N		Y N		Y N	
53. Amphetamines	Y N		Y N		Y N	
54. Cathinones	Y N		Y N		Y N	
55. Hallucinogens	Y N		Y N		Y N	
56. PCP	Y N		Y N		Y N	
57. Ketamine	Y N		Y N		Y N	
58. Heroin	Y N		Y N		Y N	
59. Rx Stimulant	Y N		Y N		Y N	
60. Rx Opioid	Y N		Y N		Y N	
61. Rx Anxiolytic	Y N		Y N		Y N	

Ever gotten “hooked on”, taken for fun, or used prescription medications for reasons or in ways other than rx or when not prescribed to you?

Felt used too much, caused problems, tried cut down or quit, gone to anyone for help or received tx due to use

1) Recurrent use resulting role failure
2) Recurrent use physically hazardous
3) Continued use despite persistent interpersonal problems related use
4) Craving
5) Taken larger amounts longer periods intended
6) Persistent desire, unsuccessful efforts cut down control use
7) Great deal time spend obtain, use, recover use
8) Important social, occupational, recreational given up due to use
9) Continued use despite knowledge persistent recurrent physical psychological problem related use
10) Tolerance
11) Withdrawal

D. MEDICAL HISTORY (Optional)

HT with LOC ____ Yes ____ No

Other LOC ____ Yes ____ No

Seizure? ____ Yes ____ No

Migraine? ____ Yes ____ No

Multiple Sclerosis? ____ Yes ____ No

CVA (Stroke)? ____ Yes ____ No

Sleep apnea ____ Yes ____ No

Thyroid ____ Yes ____ No

Tx with corticosteroids ____ Yes ____ No

Any medical problems or medications taking impacting mood?

____

____

____

E. Family History

From phone screen: ______________________________________

79a. # Siblings: ___ F 79b. ___ M

80a. # Children:___ F 80b. ___ M

	Nuclear									Maternal							Paternal					
Code:<br>3= Professionally dx/tx<br>2 = Likely description<br>1 = Negative<br>? = No info available	Mother	Father		Sister	Brother		Daughter	Son		Other	GM	GF	Aunt	Uncle	Cousin		Other	GM	GF	Aunt	Uncle	Cousin
62. **Bipolar Disorder**																						
63. Major Depression																						
64. Schizophrenia																						
65. Schizoaffective																						
66. ADD/ADHD																						
67. Panic																						
68. Agoraphobia																						
69. Social Phobia																						
70. PTSD																						
71. GAD																						
72. OCD																						
73. Bulimia																						
74. Anorexia																						
75. Alcohol Use Disorder																						
76. Substance Use Disorder																						
77. Psych Hospitalization																						
78. Suicide																						
79. Suicide Attempt																						

LW-CMF Scoring: LiveWell Clinical Monitoring Form ADE Scoring V1.1

ID:________________ Date:________________ Follow-Up Month:________________ Interviewer:________________

CMF Scoring DSM4<br>Start Date/Week ____________<br>Stop Date/Week ____________<br>Current or Follow-Back	Symptom Severity											Symptom Criteria	Entry Criteria	e c u t
	< < < DECREASED < < <					WELL	> > > INCREASED > > >							
	Severe	Marked	Moderate	Mild		None		Mild	Moderate	Marked	Severe			
**DEPRESSION**														
**1. Depressed mood (A)**	-	-	-	-	-	0	+1/4	+1/2	+1	+1.5	+2			-
**2. Decreased interest (B)**	-2	-1.5	-1	-1/2	-1/4	0	-	-	-	-	-			-
**4. Insomnia/Hypersomnia**	-2	-1.5	-1	-1/2	-1/4	0	+1/4	+1/2	+1	+1.5	+2		-	-
**6. Fatigue/Energy**	-2	-1.5	-1	-1/2	-1/4	0	-	-	-	-	-		-	-
**5b. Psychomotor retardation**	-	-	-	-	-	0	+1/4	+1/2	+1	+1.5	+2		-	-
**5a. Psychomotor agitation**	-	-	-	-	-	0	+1/4	+1/2	+1	+1.5	+2		-	-
**3. Appetite/Weight**	-2	-1.5	-1	-1/2	-1/4	0	+1/4	+1/2	+1	+1.5	+2		-	-
**8. Concentration/Indecisiveness**	-2	-1.5	-1	-1/2	-1/4	0	-	-	-	-	-		-	-
**7a. Guilt**	-	-	-	-	-	0	+1/4	+1/2	+1	+1.5	+2		-	-
**7b. Self-esteem/ Worthless**	-2	-1.5	-1	-1/2	-1/4	0	-	-	-	-	-		-	-
**9. Suicidal ideation**	-	-	-	-	-	0	+1/4	+1/2	+1	+1.5	+2		-	-
**10. Impairment**	-	-	-	-	-	0	+1/4	+1/2	+1	+1.5	+2	-		-
**11. Other causes**	-	-	-	-	-	-	-	-	-	-	-	-		-
**MANIA**														
**1. Elevated/Expansive (A)**	-	-	-	-	-	0	+1/4	+1/2	+1	+1.5	+2	-		
**2. Irritable (B)**	-	-	-	-	-	0	+1/4	+1/2	+1	+1.5	+2	-		
**8c. Increased energy (C)**	-	-	-	-	-	0	+1/4	+1/2	+1	+1.5	+2	-	-	
**8a. Goal directed activity (C, 6a)**	-	-	-	-	-	0	+1/4	+1/2	+1	+1.5	+2		-	
**8b. Psychomotor agitation (6b)**	-	-	-	-	-	0	+1/4	+1/2	+1	+1.5	+2		-	-
**4. Need for sleep (2)**	-2	-1.5	-1	-1/2	-1/4	0	-	-	-	-	-		-	-
**3. Self Esteem (1)**	-	-	-	-	-	0	+1/4	+1/2	+1	+1.5	+2		-	-
**5. More talkative (3)**	-	-	-	-	-	0	+1/4	+1/2	+1	+1.5	+2		-	-
**6. FOI/Racing thoughts (4)**	-	-	-	-	-	0	+1/4	+1/2	+1	+1.5	+2		-	-
**7. Distractible (5)**	-	-	-	-	-	0	+1/4	+1/2	+1	+1.5	+2		-	-
**9. High risk behavior (7)**	-	-	-	-	-	0	+1/4	+1/2	+1	+1.5	+2		-	-
**10. Hospitalized** (due to mania)	-	-	-	-	-	-	-	-	-	-	-	-		-
**11. Psychosis**	-	-	-	-	-	-	-	-	-	-	-	-		-
**12. Impairment**	-	-	-	-	-	0	+1/4	+1/2	+1	+1.5	+2	-		-
**13. Other causes**	-	-	-	-	-	-	-	-	-	-	-	-		-

Symptom criteria: If |severity| ≥ 1 enter 1 otherwise enter 0. For symptoms with parts a and b use highest score.
Symptom and impairment entry criteria: If |severity| ≥ 1 enter 1 otherwise enter 0.
Hospitalized, psychosis, and other causes entry criteria: If present, enter 1 otherwise enter 0.
# of Consecutive days: Enter number of consecutive days with |severity| ≥ 1; if 7 or more enter 7.

LW-CMF Scoring: LiveWell Clinical Monitoring Form ADE Scoring V1.1			
ID:_______________	Date:_______________	Follow-Up Month:_______________	Interviewer:______________

**History of:** <br>**(Check all that apply)**	
Depression	
Mania	
Hypomania	
Mixed	
**RELIABILITY** __________ Use: 1 = very good, 2 = good, 3 = fair Drop: 4 = poor, 5 = very poor	

Manic episodes better explained by direct physiological effects of a substance or a general medical condition? ____ Yes____ No

Diagnosis is more consistent with schizophrenia or schizoaffective disorder than bipolar disorder? ____ Yes____ No

DSM4 Clinical Status Coding (*If new onset depression/mania/hypomania, go to crisis protocol):

Clinical Status	Entry criteria met?	Symptom Criteria	Impairment	Consecutive Days	CSC	PSR
Mixed	Yes, mania and depression#	Both mania and depression#	≥ 1	≥ 7#	8	5-6
Depression	Yes	Five	≥ 1		7	5-6
Mania	Yes	Three if elevated, four if only irritable	≥ 1 or hospitalized or psychosis	≥ 7 or hospitalized	6	5-6
Hypomania	Yes	Three if elevated, four if only irritable	< 1	≥ 4	5	3
	#Mania with concurrent depression for 1 week. Count depressive symptoms for 5/7 days instead of 10/14.					
