# Supplementary material for: A Smartphone-Based Self-management Intervention for Individuals With Bipolar Disorder (LiveWell): Empirical and Theoretical Framework, Intervention Design, and Study Protocol for a Randomized Controlled Trial
Source: JMIR Res Protoc. 2022 Feb 21;11(2):e30710. doi: 10.2196/30710 (PMC8902672; doi:10.2196/30710)
Supplement: Multimedia Appendix 7 [file resprot_v11i2e30710_app7.pdf]

LW-CMA: LiveWell Combined Mood Assessment V1

ID:_____________Date:_____________Follow-Up Month: ____________ Interviewer:_____________

INSTRUCTIONS TO INTERVIEWERS:

This assessment tool covers mood for the last two weeks.

The first question for each item (in bold print) should be asked as written.
Use open-ended follow-up questions until you have enough information to rate the item confidently.
Add your own follow-up questions to obtain necessary information.

If answer to specified question already known, confirm information and make rating.

Final score each item reflects assessment balancing severity and frequency of symptom.

Patients with chronic symptoms may not be able to identify a period of normalcy.
However, depression should not be rated as "normal" (i.e., a rating of "0") in these cases.

Initial Overview

I'd like to ask you some questions about how you have been doing in the **past two weeks**.

Periodically remind participant that you are specifically asking about the last two weeks.

May prompt compare how doing now compared to when feel ok.

This interview covers:	Start Date	_____________	Stop Date	_____________
	Study Start Week	_____________	Stop Week	_____________
	Study Start Month	_____________	Stop Month	_____________

DEPRESSED MOOD

Over the past 2 weeks have you been feeling down or depressed?
Sad? Hopeless? Helpless? Worthless?

IF YES: Can you describe what this feeling has been like for you? How bad is the feeling?

How often have you felt (sad, hopeless, helpless, down, depressed, or own equivalent)? Every day? All day?

Have you been crying at all?

1 - QIDSC 5. Mood (Sad):

0 - Does not feel sad
1 - Feel sad less than half the time
2 - Feels sad more than half the time
3 - Feels intensely sad virtually all of the time

2. – CMF Depression 1a. Depressed Mood, Severity (DSM A): Depressed mood most of the day, nearly every day during the same 2 week period

Over these 2 weeks, did you feel down or depressed?
When something good, even small things have happened, does your mood brighten up? How long?
Were there things that occurred that should have brightened your mood but did not?
How have you been feeling about the future? (optimistic/pessimistic)

Persistently feels "depressed", "sad", "down", "blue" or equivalent dysphoria.			**Comments**
**0**	**Not depressed**		
	**Frequency**	**Intensity**	
**+1/4**	Any	Any dysphoria	
**+1/2**			
**+1**	≥10/14 days	Depressed dysphoric mood<br>Most of the day	
**+1.5**			
**+2**	≥10/14 days	Constant unremitting intense dysphoria	

How have you been spending your time in the past two weeks (when not at work)?

Is that normal for you?

Have you felt interested in doing (usual things, current activities)? Is there anything you look forward to?

Do you feel you have to push yourself to do them?

How would you describe your level of interest and motivation to complete daily activities?

Have you stopped doing anything you used to do? (What about hobbies?) IF YES: Why?

About how many hours a day do you spend doing things that interest you?

3 - QIDSC 13. Involvement:

0 - No change from usual level of interest in other people and activities
1 - Notices a reduction in former interests/activities
2 - Finds only one or two former interests remain
3 - Has virtually no interest in formerly pursued activities

4 - CMF Depression 2a. Decreased Interest, Severity (DSM B): Markedly diminished interest or pleasure in all or almost all activities most of the day, nearly every day during the same 2 week period

Were you unable to enjoy pleasant things that happened?
How would you describe your level of interest and motivation to complete daily activities?

Loss of motivation or connectedness with others, loss of interest in or diminished capacity for enjoyment of pleasurable activities, push self to work or activities.			**Comments**
**0**	**Enjoys activities as usual**		
	**Frequency**	**Intensity**	
**-1/4**	Any	Any decreased interest	
**-1/2**			
**-1**	≥10/14 days	Loss of interest or enjoyment in most things<br>Disinterest decreased motivation but able to enjoy some activities under favorable conditions<br>Most of the day	
**-1.5**			
**-2**	≥10/14 days	Much less interested, Emotionally constricted<br>Can't cry, no response to favorable stimuli<br>Most of the day	

How has your appetite been? What about compared to your usual appetite?

IF LESS: How much less?

Have you had to force yourself to eat?
Have other people had to urge you to eat? (Have you skipped meals?)
Have you found yourself eating more than usual? Every day?
Have you noticed you eat more at meals?
Have you noticed you are snacking or eating more in between meals?
Have you felt driven to eat?
Have you had eating binges?

5 - QIDSC 6. Appetite (Decreased):

0 - No change from usual appetite
1 - Eats somewhat less often and/or lesser amounts than usual
2 - Eats much less than usual and only with personal effort
3 - Eats rarely within a 24-hour period, and only with extreme personal effort or with persuasion by others

6 - QIDSC 7. Appetite (Increased):

0 - No change from usual appetite
1 - More frequently feels a need to eat than usual
2 - Regularly eats more often and/or greater amounts than usual
3 - Feels driven to overeat at and between meals

Have you lost any weight since this (DEPRESSION) began? How much did you lose?

IF YES: Was it because of feeling depressed or down?

IF NOT SURE: Do you think your clothes are any looser on you?

How much has your weight changed in the past 2 weeks?

7 - QIDSC 8. Weight (Decrease) Within The Last Two Weeks:

0 - Has experienced no weight change
1 - Feels as if some slight weight loss occurred
2 - Has lost 2 pounds or more
3 - Has lost 5 pounds or more

8 - QIDSC 9. Weight (Increase) Within The Last Two Weeks:

0 - Has experienced no weight change
1 - Feels as if some slight weight gain has occurred
2 - Has gained 2 pounds or more
3 - Has gained 5 pounds or more

9 - CMF Depression 3. Appetite/Weight: Significant weight loss when not dieting or weight gain or decrease or increase in appetite nearly every day during the same 2 week period

How was your appetite?

Disturbance of appetite, ↑/↓ from normal.			Comments
**0**	**Normal appetite/weight**		
	**Frequency**	**Intensity**	
**+1/4 or -1/4**	Any	Mild or rare ↑/↓ in appetite	
**+1/2 or -1/2**			
**+1 or -1**	≥10/14 days	Reduced or increased consumption about 25%	
		Needs encouragement to eat	
		Craving food or seeks snacks in addition to usual meals	
**+1.5 or -1.5**			
**+2 or -2**	≥10/14 days	Weight loss or gain ≥ 5% body in 2 weeks	
		Decrease or increase 50% of normal consumption	
**Count all days with appetite disturbance and use +/- to indicate predominant direction**			

How has your energy been in the past two weeks?

IF LOW ENERGY: Have you felt tired? (How much of the time? How bad has it been?)

10 - QIDSC 14. Energy/Fatigability:

0 - No change in usual level of energy
1 - Tires more easily than usual
2 - Makes significant personal effort to initiate or maintain usual daily activities
3 - Unable to carry out most of usual daily activities due to lack of energy

11 - CMF Depression 6. Fatigue/Energy: Fatigue or loss of energy nearly every day during the same 2 week period

How was your energy level?
Were there things you should have done and didn't because you didn't have enough energy or were too tired?

Fatigue, decreased energy, feels tired or tires easily.			Comments
**0**	**Usual energy level.**		
	**Frequency**	**Intensity**	
**-1/4**	Any	Carries out all activities	
		Occasionally tired, some tasks are harder	
		More drained than usual	
**-1/2**			
**-1**	≥10/14 days	Interferes with some activities at work, home, socially (Not due to a lack of motivation)	
**-1.5**			
**-2**	≥10/14 days	Lethargic, Stays in bed	

Have you noticed feeling restless or fidgety in the past two weeks?

Have you found yourself unable to stay seated or needing to move around?

RATING BASED ON OBSERVATION DURING INTERVIEW AND PATIENT SELF-REPORT

12 - QIDSC 16. Psychomotor Agitation:

0 - No increased speed or disorganization in thinking or gesturing
1 - Fidgets, wrings hands and shifts positions often
2 - Describes impulse to move about and displays motor restlessness
3 - Unable to stay seated. Paces about with or without permission

13 - CMF Depression 5a. Psychomotor agitation: Psychomotor agitation nearly every day during the same 2 week period

Were there times you were so fidgety or agitated it was hard for you to stay still?

Fidgetiness, playing with hands, hair, etc. Moving about, can't sit still, purposeless activity.			**Comments**
**0**	**No evidence of motor agitation.**		
	**Frequency**	**Intensity**	
**+1/4**	Any	Restlessness, fidgeting, purposeless movement, pacing	
**+1/2**			
**+1**	≥10/14 days	Difficulty remaining still or purposeless movement observed by others	
**+1.5**			
**+2**	≥10/14 days	Pacing, unable to sit still when necessary	

Have you felt slowed down in your thinking, speaking, or movement in the past two weeks?

Have others commented on this?

RATING BASED ON OBSERVATION DURING INTERVIEW AND PATIENT SELF-REPORT

14 - QIDSC 15. Psychomotor Slowing:

0 - Normal speed of thinking, gesturing, and speaking
1 - Patient notes slowed thinking, and voice modulation is reduced
2 - Takes several seconds to respond to most questions; reports slowed thinking
3 - Is largely unresponsive to most questions without strong encouragement

15 - CMF Depression 5b. Psychomotor retardation: Psychomotor retardation nearly every day during the same 2 week period

Were there times you were moving or thinking more slowly than usual?
If I had been with you, would I have noticed something was wrong?

Slowness of thought and speech, impaired ability to concentrate, decreased motor activity.			**Comments**
**0**	**No evidence of motor, speech, or cognitive slowing.**		
	**Frequency**	**Intensity**	
**+1/4**	Any	Subjective slowing of thoughts, speech, or movement or rare objective evidence of retardation	
**+1/2**			
**+1**	≥10/14 days	Slowness thought or movement observable by others<br>Increased speech latency	
**+1.5**			
**+2**	≥10/14 days	Apparent on interview	

16 - CMF Depression 7a. Guilt: Feelings of excessive or inappropriate guilt nearly every day during the same 2 week period

Were there times you were down on yourself? Or felt as if you were a bad person or that you deserved to suffer?

Self-reproach, feels let people down, present illness is a punishment. Delusions of guilt, hears accusatory voices, threatening visual hallucinations.			Comments
**0**	**No excessive self-blame or guilty preoccupation.**		
	**Frequency**	**Intensity**	
**+1/4**	Any	Mild, rare self-deprecatory thoughts	
**+1/2**			
**+1**	≥10/14 days	Self-deprecatory thoughts (Not limited inability fx due illness)<br>Guilt or ruminations over past errors/sinful deeds	
**+1.5**			
**+2**	≥10/14 days	Self-deprecatory thoughts (Not limited inability fx due illness)<br>Guilt or ruminations over past errors/sinful deeds<br>Most of the day	

Have you been putting yourself down this past week, feeling you've done things wrong, or let others down? IF YES: What have your thoughts been? Has this been more than is normal for you?

In the past week, how have you felt about yourself?
Have you noticed your self-esteem has been down in the past two weeks?
How would you rate your worth as a person compared to others?
Have you been feeling guilty about anything that you've done or not done?
What about things that happened a long time ago?

17 - QIDSC 11. Outlook (Self):

0 - Sees self as equally worthwhile and deserving as others
1 - Is more self-blaming than usual
2 - Largely believes that he/she causes problems for others
3 - Ruminates over major and minor defects in self

18 - CMF Depression 7b. Worthlessness: Feelings of worthlessness nearly every day during the same 2 week period

Was your self-esteem or self-confidence down compared to usual?

Feels inferior, defective, incompetent, inadequate			Comments
**0**	**Normal self-esteem/self-confidence**		
	**Frequency**	**Intensity**	
**-1/4**	Any	Felt mildly down on self, lacking self-confidence	
**-1/2**			
**-1**	≥10/14 days	Feels inferior to most others<br>Stops work or social activities due to expectation of failure	
**-1.5**		Worthlessness	
**-2**	≥10/14 days	Delusional	

How has your concentration been in the past two weeks?
Were you able to focus on what you were doing (like reading or watching TV)?
Did you notice that minor decisions were more difficult to make than usual (what to wear, eat, watch on TV)?

19 - QIDSC 10. Concentration/Decision Making:

0 - No change in usual capacity to concentrate and decide
1 - Occasionally feels indecisive or notes that attention often wanders
2 - Most of the time struggles to focus attention or make decisions
3 - Cannot concentrate well enough to read or cannot make even minor decisions

20 - CMF Depression 8. Concentration/Indecisiveness: Diminished ability to think or concentrate, or indecisiveness, nearly every day during the same 2 week period

How was your concentration?
Did you have any difficulty reading or collecting your thoughts in a conversation?

Inability to concentrate, inability to focus on a task, difficulty making decisions.			**Comments**
**0**	**Normal concentration.**		
	**Frequency**	**Intensity**	
**-1/4**	Any	Rare or limited to unpleasant, very difficult tasks	
**-1/2**			
**-1**	≥10/14 days	Difficult to read or collect thoughts in conversation	
		Difficult to function in role at home/work	
**-1.5**			
**-2**	≥10/14 days	Clear cognitive impairment during casual interaction	
		Unable to function in role at home/work	

Have you had thoughts that life is not worth living?
What about thinking you'd be better off dead or wishing you were dead?
Have you had thoughts of hurting or killing yourself?
IF YES: What have you thought about?
How often do these thoughts come? How long do they stay? Have you thought of a plan in the last week?
Have you done anything to try to hurt yourself or taken any steps toward ending your life?

21 - QIDSC 12. Suicidal Ideation:
0 - Does not think of suicide or death
1 - Feels life is empty or is not worth living
2 - Thinks of suicide/death several times a week for several minutes
3 - Thinks of suicide/death several times a day in depth, or has made specific plans, or attempted suicide

IFQIDSC #12 SI ≥ 2, GO TO SI PROTOCOL.

22 - CMF Depression 9. Suicidal Ideation (SI): Recurrent thoughts of death, recurrent suicidal ideation without a specific plan, or a suicide attempt or a specific plan for committing suicide

Were there times you were feeling so bad that you felt life was not worth living?
What about actually thinking about suicide or harming yourself?

Weary of life, would be better off dead, morbid preoccupation, thoughts of harming self, plans for self-destruction, urge to end life			Comments
**0**	**No SI.**		
	**Frequency**	**Intensity**	
**+ 1/4**	Rare	Fleeting LNWL, Fleeting passive SI	
**+ 1/2**	Several days	Fleeting LNWL or fleeting passive or active SI	
		Persistent periods of passive SI	
**+1**	≥ 10/14 days	Most of the day with LNWL	
		Persistent periods of passive SI	
	Several days	Brief active SI	
**+1.5**	> 1 day	Persistent periods of active SI	
**+2**	≥ 10/14 days	Most of the day with active SI	
	Any	Active SI with intent, plan, or action	
		Active SI unable to control thoughts impulses	
**IF CMF #9 SI SYMPTOM SCORE ≥ 1, GO TO SI PROTOCOL**			

LNWL: Life Not Worth Living.
Passive SI: Thoughts of death **without** plan for self-destruction, no action or urge to act.
Active SI: Suicidal thoughts with plan for self-destruction, but no action or urge to act.
Fleeting: < 1 minute **Brief:** 1-15 minutes **Persistent**: ≥ 15 minutes

23 – CMF Depression 10. Impairment: The depressive symptoms cause clinically significant distress or impairment in social, occupational, or other important areas of functioning.

Have your depressive symptoms caused any major changes or problems in your

- *sleep, eating, or hygiene and grooming?*
- *interactions with other such as withdrawal or conflicts?*
- *family responsibilities?*
- *work, school, or volunteer responsibilities?*

			**Comments**
**0**	**No significant distress or impairment**		
	**Frequency**	**Intensity**	
**+1/4**	Any		
**+1/2**			
**+1**	≥ 10/14 days	Poorly groomed, moderately disheveled	
		Conflicts with others	
		Decreased function in role at home or work	
		Social withdrawal	
**+1.5**			
**+2**	≥ 10/14 days	Completely unkempt, disheveled	
		Inability to function in role at home or work	
		Social isolation	
		Life-threatening behaviors	

Symptoms possibly consistent with marked or severe impairment: Fatigue/Energy, Psychomotor retardation/agitation, Concentration/Indecisiveness, Worthlessness, Suicidal ideation.

ELEVATED MOOD

25 - CMF Mania, 1a. Elevated/Expansive, Severity (DSM A): Distinct period of elevated or expansive mood during the same 1 week period

Over these 2 weeks, did you feel so good that people thought you were not your normal self? Felt very good, too cheerful, high, optimistic attitude out of proportion to circumstances?

			**Comments**
**0**	**No mood elevation or expansiveness.**		
	**Frequency**	**Intensity**	
**+1/4**	Any	Any elevation/expansiveness not clearly related to events	
**+1/2**			
**+1**	≥4/7 days<br>or hospitalized	Feeling so good high, excited or optimistic people thought not normal self<br>Abnormally persistently elevated, expansive, euphoric, excessively cheerful, high, “on top of the world”	
**+1.5**		Clearly elated, exalted expressions.	
**+2**	≥4/7 days	Psychotic features such as belief in divine powers	

26. CMF Mania 1b. Elevated/Expansive, DSM Consecutive Days:
How many consecutive days with elevated/expansive mood of intensity >= 1? _____ # of days (0-7 days)

27 - YMRS 1. Elevated Mood

Has your mood been higher (better) than usual? Have there been times when you felt unusually good, cheerful, or happy?

0 Absent
1 Mildly or possibly increased on questioning
2 Definite subjective elevation; optimistic, self-confident; cheerful; appropriate to content
3 Elevated; inappropriate to content; humorous
4 Euphoric; inappropriate laughter; singing

28 - YMRS 5. Irritability

Have you been irritable? IF YES: Is this more than is normal for you?
How have you been getting along with people in general?

Have you been feeling irritable or angry?

How much of the time?

0 Absent
2 Subjectively increased
4 Irritable at times during interview; recent episodes of anger or annoyance on ward
6 Frequently irritable during interview; short, curt throughout
8 Hostile, uncooperative; interview impossible

29 - YMRS 9. Disruptive-Aggressive Behavior

Have you been more disruptive or aggressive?
Have you been involved in any arguments or fights? How often?

0 Absent, cooperative
2 Sarcastic; loud at times, guarded
4 Demanding; threats on ward
6 Threatens interviewer; shouting; interview difficult
8 Assaultive; destructive; interview impossible

30 - CMF Mania 2a. Irritability, Severity (DSM B): Distinct period of irritable mood during the same 1 week period

Were you so irritable that you shouted at people or started fights or arguments?
Have you been unusually argumentative or impatient?
Have you found yourself becoming angry with others for little apparent reason? More so than normal for you?

			Comments
**0**	**No irritability**		
	**Frequency**	**Intensity**	
**+1/4**	Any	Any irritability not clearly related to events	
**+1/2**			
**+1**	≥4/7 days<br>or hospitalized	Clearly abnormal irritability with behavioral manifestations (e.g. starts fights/arguments)	
**+1.5**			
**+2**	≥4/7 days	Psychotic features such as paranoia	

31. CMF Mania Irritable, DSM Consecutive Days:
How many consecutive days with irritable mood of intensity >= 1? _____ # of days (0-7 days)

32 - CMF Mania 8a1. Goal Directed Activity, Severity (6a, DSM5 C): Increase in goal directed activity during the same 1 week period

Did you have an increase in activity either socially, sexually, at work or school? Were you so active that people worried about you taking on so much? Did you find you were so active that you really didn't get much done?

Plans, projects, purposeful activities			**Comments**
**0**	**No increase in goal directed behavior**		
	**Frequency**	**Intensity**	
**+1/4**	Any	Initiated one or more new projects	
**+1/2**			
**+1**	≥4/7 days or hospitalized	Multiple creative, self-improvement, home projects w/out external requirements	
		New projects require commitment of > 8hrs/wk or > 5% of income	
**+1.5**			
**+2**	≥4/7 days	Work effort > 10hrs/day or work after 9 pm	

33. CMF Mania 8a2. Goal Directed Activity, DSM Consecutive Days:
How many consecutive days with increased goal directed activity of intensity >= 1? _____ # of days (0-7 days)

34- YMRS 2. Increased Motor Activity-Energy

Does your energy level or motor activity appear to be greater than usual?

Have there been times when you were unable to sit still or times when you had to be moving or pacing back and forth?

Have you had more energy than usual?

Have you been more active (either socially or sexually) than usual or had the feeling you could go all day without feeling tired?

0 Absent
1 Subjectively increased
2 Animated; gestures increased
3 Excessive energy; hyperactive at times; restless (can be calmed)
4 Motor excitement; continuous hyperactivity (cannot be calmed)

35 - CMF Mania 8b1, Psychomotor agitation, Severity (6b, DSM5 C): Increase in psychomotor agitation during the same 1 week period

Over these 2 weeks, were there times you were so fidgety or agitated it was hard for you to stay still?
Were you physically or mentally restless?

Fidgetiness, playing with hands, hair, etc. Moving about, can't sit still. Purposeless non-goal-directed activity.			**Comments**
**0**	**No evidence of motor agitation**		
	**Frequency**	**Intensity**	
**+ 1/4**	Any	Restless, fidgets	
**+ 1/2**			
**+1**	≥4/7 days<br>or hospitalized	Difficulty remaining still or purposeless movement observed by others	
**+1.5**			
**+2**	≥4/7 days	Pacing, unable to sit still when necessary	

36. CMF Mania 8b2. Psychomotor Agitation, DSM Consecutive Days:
How many consecutive days with psychomotor agitation of intensity >= 1? _____ # of days (0-7 days)

37. - CMF Mania 8c1. Increased energy, Severity (DSM4 A DSM5 C): Increased energy during the same 1 week period

Over these 2 weeks, did you feel so hyper that people thought you were not your normal self, or were you so hyper you got in trouble? Did you have more energy than usual to do things? Did it seem like too much energy?

Unusually energetic more active than usual self without expected fatigue.			**Comments**
**0**	**No abnormally increased energy**		
	**Frequency**	**Intensity**	
**+1/4**	Any	Slightly more energetic	
**+1/2**		Little change in activity level but less fatigued than usual	
**+1**	≥4/7 days<br>or hospitalized	Hyper and not their normal self.<br>So hyper got into trouble or observed manic behavior.<br>Unusual behavior but didn't get into trouble due to not observed<br>Somewhat more active than usual with little or no fatigue	
**+1.5**			
**+2**	≥4/7 days	Much more active than usual, unusually active all day long with little or no fatigue	

38. CMF Mania 8c5. Increased Energy, DSM Consecutive Days:
How many consecutive days with increased energy of intensity >= 1? _____ # of days (0-7 days)

39 - YMRS 4. Sleep

Has your sleep decreased?

How much sleep do you ordinarily need?

Have you needed less sleep than usual to feel rested?

How much sleep do/did you need?

0 Reports no decrease in sleep
1 Sleeping less than normal amount by up to one hour
2 Sleeping less than normal by more than one hour
3 Reports decreased need for sleep
4 Denies need for sleep

40 - CMF Mania 4. Need for Sleep DSM (2): Decreased need for sleep during the same 1 week period

Were there nights when you got less sleep than usual and found you didn't really miss it?
(Do not count simple insomnia)

Amount of sleep, subjective need for sleep, ability to function.			**Comments**
**0**	**Usual need for sleep**		
	**Frequency**	**Intensity**	
**- 1/4**	Any	Mild ↓; no impact on function	
**- 1/2**			
**-1**	≥4/7 days	Sleep reduced ≥ 1.5 hrs without impact on next day function	
**-1.5**			
**-2**	≥4/7 days	Sleep reduced by >5hrs from usual or sleeping <2hrs/day	

41 - YMRS 8. Content

Have you had any new plans, interests, or projects? Have you felt more self-confident than usual?

Have you felt that you were a particularly important person or that you had special powers, knowledge or abilities that were out of the ordinary?

Is there a special mission or purpose to your life? Do you have a special relationship with God?

Have you felt that anyone was trying to harm you or hurt you for no reason? Can you give an example?

Have you felt as if you were being controlled by an external force or power?

Have you felt as if people on the radio or TV were talking to you, about you, or communicating to you in some special way?

Have you had any (other) strange or unusual beliefs or ideas?

Have these beliefs interfered with your functioning in any way?

Have you heard sounds or voices of people talking when there was no one around?

Have you seen any visions or smelled odors that others don't seem to notice?

Have you had any (other) strange or unusual perceptions?

Have these experiences interfered with your functioning in any way?

0 Normal
2 Questionable plans, new interests
4 Special project(s); hyper-religious
6 Grandiose or paranoid ideas; ideas of reference
8 Delusions; hallucinations

42 - CMF Mania 3. Self-Esteem/Grandiosity DSM (1): Inflated self-esteem or grandiosity during the same 1 week period

Were there times when you were feeling more self-confident than usual?
Were there times when you were feeling more special, more talented, more attractive or smarter than usual? Were there any times when your thoughts were grandiose?

Inflated self-confidence, feels more attractive, more talented, able to do more than usual.			**Comments**
**0**	**No increase in self-esteem**		
	**Frequency**	**Intensity**	
**+1/4**	Any	Some exaggerated sense of abilities	
**+1/2**			
**+1**	≥ 4/7 days	Clearly inflated estimate of capabilities<br>Actual performance may be increased but assessment excessively positive	
**+1.5**		Grossly excessive ideas of worth or abilities	
**+2**	≥4/7 days	Delusional	

43 - YMRS 6. Speech (Rate and Amount)

Have you been talking more quickly or more than usual?

0 No increase
2 Feels talkative
4 Increased rate or amount at times, verbose at times
6 Push; consistently increased rate and amount; difficult to interrupt
8 Pressured; uninterruptible, continuous speech

44 - CMF Mania 5. More Talkative DSM (3): More talkative than usual or pressure to keep talking during the same 1 week period

Were there times when you were more talkative than usual, or you found you said more than you intended? Were there times that you spoke much faster than usual?

Amount and rate of speech.			**Comments**
**0**	**Normal rate and quantity of speech**		
	**Frequency**	**Intensity**	
**+1/4**	Any	Others note talkative, not out of character/bothersome	
**+1/2**			
**+1**	≥4/7 days	Others complain about excessive talking, uncharacteristically cutting others off	
		Pressured speech (described or observed)	
		Conversation seeking	
		Communication reveals more than intended	
**+1.5**			
**+2**	≥4/7 days	Hard for others to get a word in	
		Virtually incessant talking	

45 - YMRS 7. Language-Thought Disorder

Have you had changes in your thought patterns?

Have you been bothered by having too many thoughts at one time?

Have you had thoughts racing through your mind? How often? Does it hinder your functioning?

0 Absent
1 Circumstantial; mild distractibility; quick thoughts
2 Distractible, loses goal of thought; changes topics frequently; racing thoughts
3 Flight of ideas; tangentiality; difficult to follow; rhyming, echolalia
4 Incoherent; communication impossible

46 - CMF Mania 6. FOI/Racing Thoughts DSM (4): Flight of ideas or subjective experience that thoughts are racing during the same 1 week period

Did you find that you had more ideas than usual?
Were there times when your thoughts seemed to be racing through your head?

Racing thoughts, ↑ in train of productive, novel, unrelated ideation.			**Comments**
**0**	**No racing thoughts**		
	**Frequency**	**Intensity**	
**+1/4**	Any	Mild thinking fast	
**+1/2**			
**+1**	≥4/7 days	Ideas race, come tumbling out<br>Rapid train of thoughts	
**+1.5**			
**+2**	≥4/7 days	Speech cannot keep up w/ pressured thoughts.	

47 - CMF Mania 7. Distractibility DSM (5): Distractibility during the same 1 week period

Did you find you were easily distracted?

Unable to maintain focus of attention, easily distracted by external or internal stimuli.			**Comments**
**0**	**No evidence of distractibility.**		
	**Frequency**	**Intensity**	
**+1/4**	Any	Generally able to maintain focus	
**+1/2**			
**+1**	≥4/7 days	Decreased ability to complete tasks due to distractibility<br>Able to complete tasks but requires great effort	
**+1.5**			
**+2**	≥4/7 days	Obvious in most conversations<br>Can’t stay on topic to complete most sentences thoughts	

48 - YMRS 3. Sexual Interest

Have you had more than usual interest in sexual matters?

Has your interest in sex increased?

0 Normal; not increased
1 Mildly or possibly increased
2 Definite subjective increase on questioning
3 Spontaneous sexual content; elaborates on sexual matters; hypersexual by self-report
4 Overt sexual acts (towards patients, staff, or interviewer)

49 - CMF Mania 9. High Risk Behavior DSM (7): Excessive involvement in pleasurable activities that have a high potential for painful consequences during the same 1 week period

Did you do anything that was unusual for you or that other people might think was excessive, foolish, or risky? Did you do anything which would have caused a problem if you were caught?

Excessive, foolish, risky activities that could have serious consequences for self/others (whether or not caught)			**Comments**
**0**	**No risk taking.**		
	**Frequency**	**Intensity**	
**+1/4**	Any	Mild risk taking/↑ pleasure seeking	
**+1/2**			
**+1**	≥4/7 days	Exhibits behavior others would recognize as foolish/risky/excessive<br>Gambling, sex, investments, reckless driving →(–) consequences<br>Definitely hazardous behavior → adverse effects social, sexual, financial; outside normal risk tolerance	
**+1.5**			
**+2**	≥4/7 days	Extremely hazardous, life-threatening physical, catastrophic financial or social	

50 - YMRS 10. Appearance

Has your interest in your appearance changed?

0 Appropriate dress and grooming
1 Minimally unkempt
2 Poorly groomed; moderately disheveled; overdressed
3 Disheveled; partly clothed; garish make-up
4 Completely unkempt; decorated; bizarre garb

51 - YMRS 11. Insight

Do you think your treatment is appropriate with how you're doing at this time?

0 Present; admits illness; agrees with need for treatment
1 Possibly ill
2 Admits behavior change, but denies illness
3 Admits possible change in behavior, but denies illness
4 Denies any behavior change

52 - Mania 10. Hospitalization *Over these 2 weeks, were you hospitalized due to symptoms of mania?*

No (0) _____ Yes (1) _____

53- Mania 11. Psychosis: Psychotic or delusional symptoms of mania?

No (0) _____ Yes (1) _____

54 - Mania 12. Impairment: Mania - Manic symptoms severe enough to cause moderate impairment in occupational functioning or in usual social activities or relationships with others. **Hypomania -** Manic symptoms not severe enough to cause moderate impairment in social or occupational functioning. However, an unequivocal change in functioning uncharacteristic of person when not symptomatic is present and the disturbance in mood and change in functioning are observable by others.

Have your manic symptoms caused any major changes or problems in your

- *eating, or hygiene and grooming?*
- *interactions with other such as associating with strangers, or conflicts?*
- *sexual activity, spending habits, or substance use?*
- *family responsibilities?*
- *work, school, or volunteer responsibilities?*

Have your manic symptoms resulted in any legal problems?
Have your manic symptoms resulted in any physical altercations or violence?

			Comments
**0**	**No significant distress or impairment**		
	**Frequency**	**Intensity**	
**+1/4**	Any		
**+1/2**			
**+1**	≥4/7 days	Poorly groomed, moderately disheveled	
		Conflicts with others, associating with strangers	
		Decreased function in role at home or work	
		Hazardous, physical, financial or social behavior	
**+1.5**			
**+2**	≥4/7 days	Completely unkempt, disheveled, bizarre garb	
		Inability to function in role at home or work	
		Extremely hazardous physical, financial or social behavior	
		Violence, legal problems	

Symptoms possibly consistent with marked or severe impairment criteria for PSR: Elevated/Expansive, Irritable, Self-Esteem/Grandiosity, Psychomotor agitation, Distractible, High Risk Behavior.

ANXIETY

56 - CMF Anxiety, Abnormally Anxious Severity 1a: Abnormally anxious nearly every day most of the day

Were you abnormally anxious?
Have you been feeling especially anxious, nervous or on edge in the past 2 weeks? How much of the time?
Have you been worrying a lot about little things, things you don't ordinarily worry about?
IF YES: Like what, for example?

			Comments
**0**	**None.**		
	**Frequency**	**Intensity**	
**+1/4**	Any	Keyed up tense, restless.	
**+1/2**			
**+1**	Majority of days of most recent episode (mania, hypomania, depression)	Keyed up or tense, unusually restless, difficulty concentrating due to worries, feel something awful may happen, feel might lose control (mild-moderate,2-3 symptoms)	
**+1.5**		(moderate-severe,4-5 symptoms)	
**+2**	Majority of days of most recent episode (mania, hypomania, depression)	Severely keyed up or tense, unusually restless, difficulty concentrating due to worries, feel something awful may happen, feel might lose control (severe, 4-5 symptoms + motor agitation)	

Combined Mood Assessment Overview Information:

Combines rating for the Clinical Monitoring Form (CMF)[1,2], Quick Inventory of Depressive Symptomatology – Clinician Rated (QIDS-C)[3,4] and Young Mania Rating Scale (YMRS)[5]

For the CMF, prompts and questions used to guide rating of symptom severity were adapted from Clinical Monitoring Form: Users' Guide, Edition 2.1 Gary Sachs, MD and Jennifer Conley, MA. G.S. Sachs, M.D. 2000

For QIDSC, prompts and additional questions used to guide rating of symptom severity were adapted from: Structured Interview Guide for the Hamilton Depression Scale (SIGH-D) and Inventory of Depressive Symptomatology (IDS-C): (SIGHD-IDSC)[0].

REFERENCES

0. Structured Interview Guide for the Hamilton Depression Scale (SIGH-D) and Inventory of Depressive Symptomatology (IDS-C)[6]: (SIGHD-IDS). Instruments Combined by Kenneth A. Kobak, Janet B.W. Williams, and A. John Rush

Structured Interview Guide for the Hamilton Depression Rating Scale (SIGH-D). Janet, B.W., Williams, D.S.W.

This interview guide is based on the Hamilton Depression Rating Scale (Hamilton, Max: A rating scale for depression. J Neurol Neurosurg Psychiat 23:56-61, 1960). The anchor point descriptions, with very minor modifications, have been taken from the ECDEU Assessment Manual (Guy, William, ECDEU Assessment Manual for Psychopharmacology, Revised 1976, DHEW Publication No. (ADM) 76-338).

A reliability study of the SIGH-D has been reported (Williams JBW: A structured interview guide for the Hamilton Depression Rating Scale. Archives of General Psychiatry 45:742-747, 1988).

Copyright ©1988, 1992, 1996. All rights reserved. Permission is granted for reproduction for use by researchers and clinicians. For correspondence: Dr. Williams, New York State Psychiatric Institute, Unit 60, 1051 Riverside Drive, New York, New York 10032

1. Sachs GS, Guille C, McMurrich SL. A clinical monitoring form for mood disorders. Bipolar disorders. 2002;4(5):323-7. PubMed PMID: 12479665.
2. Sachs GS, Thase ME, Otto MW, Bauer M, Miklowitz D, Wisniewski SR, Lavori P, Lebowitz B, Rudorfer M, Frank E, Nierenberg AA, Fava M, Bowden C, Ketter T, Marangell L, Calabrese J, Kupfer D, Rosenbaum JF. Rationale, design, and methods of the systematic treatment enhancement program for bipolar disorder (STEP-BD). Biological psychiatry. 2003;53(11):1028-42. PubMed PMID: 12788248.
3. Rush AJ, Bernstein IH, Trivedi MH, Carmody TJ, Wisniewski S, Mundt JC, Shores-Wilson K, Biggs MM, Woo A, Nierenberg AA, Fava M. An evaluation of the quick inventory of depressive symptomatology and the hamilton rating scale for depression: a sequenced treatment alternatives to relieve depression trial report. Biological psychiatry. 2006;59(6):493-501. doi: 10.1016/j.biopsych.2005.08.022. PubMed PMID: 16199008; PMCID: PMC2929841.
4. Trivedi MH, Rush AJ, Ibrahim HM, Carmody TJ, Biggs MM, Suppes T, Crismon ML, Shores-Wilson K, Toprac MG, Dennehy EB, Witte B, Kashner TM. The Inventory of Depressive Symptomatology, Clinician Rating (IDS-C) and Self-Report (IDS-SR), and the Quick Inventory of Depressive Symptomatology, Clinician Rating (QIDS-C) and Self-Report (QIDS-SR) in public sector patients with mood disorders: a psychometric evaluation. Psychol Med. 2004;34(1):73-82. PubMed PMID: 14971628.
5. Young RC, Biggs JT, Ziegler VE, Meyer DA. A rating scale for mania: reliability, validity and sensitivity. Br J Psychiatry. 1978;133:429-35. PubMed PMID: 728692.
6. Rush AJ, Gullion CM, Basco MR, Jarrett RB, Trivedi MH. The Inventory of Depressive Symptomatology (IDS): psychometric properties. Psychol Med. 1996;26(3):477-86. doi: 10.1017/s0033291700035558. PubMed PMID: 8733206.

LW-Sleep: LiveWell Sleep Questionnaire V1
ID:_____________Date:_____________Follow-Up Month: ____________ Interviewer:_____________

The following questions relate to your usual sleep habits during the **past two weeks only**.
Your answers should indicate most accurate reply for the majority of days and nights in the past 2 weeks.

1 - PSQI 1: During the past two weeks, when have you usually gone to bed at night?

USUAL BED TIME _____________________

2 - PSQI 3: During the past two weeks, when have you usually gotten up in the morning?

USUAL GETTING UP TIME _____________________

3 - PSQI 4: During the past two weeks, how many hours of actual sleep did you get at night? (This may be different than the number of hours you spend in bed).

HOURS OF SLEEP PER NIGHT _____________________

4 - CMF 1: During the past two weeks, how many hours on average have you been sleeping in any one day, including naps?

HOURS OF SLEEP PER DAY _____________________

5 - CMF 2: During the past two weeks, what is the least you have slept in any one day, including naps?

MINIMUM HOURS OF SLEEP PER DAY _____________________

6 - CMF 3: During the past two weeks, what is the most you have slept in any one day, including naps?

MAXIMUM HOURS OF SLEEP PER DAY _____________________

How many hours on average have you been sleeping in a 24-hour period in the past two weeks, including naps? Is that a normal amount for you? What is the longest you've slept in a 24-hour period last week?

7 - QIDSC, 4: Hypersomnia:

0 - Sleeps no longer than 7-8 hours/night, without naps
1 - Sleeps no longer than 10 hours in a 24 hour period (include naps)
2 - Sleeps no longer than 12 hours in a 24 hour period (include naps)
3 - Sleeps longer than 12 hours in a 24 hour period (include naps)

8 - CMF 4: During the past two weeks, how often have you had to go to bed earlier than usual (EBT)?

Not at all (0) _______ Less than once a week (1)_______ Once or twice a week (2)________ Three or more times a week (3) ________

9 - CMF 5: During the past two weeks, how often have you had difficulty getting out of bed in the morning (DGOOB)?

Not at all (0) _______ Less than once a week (1)_______ Once or twice a week (2)________ Three or more times a week (3) ________

10 - CMF 6: During the past two weeks, how often have you been napping (Naps)?

Not at all (0)	Less than once a week (1)	Once or twice a week (2)	Three or more times a week (3)
_______	_______	________	________

11 - PSQI 8: During the past two weeks, how often have you had trouble staying awake while driving, eating meals, or engaging in social activity?

Not at all (0)	Less than once a week (1)	Once or twice a week (2)	Three or more times a week (3)
_______	_______	________	________

12 - KH HS 1: During the past two weeks, to what extent do you think that you feel sleepy during the daytime?

❑ 0 = Never ❑ 1 = Rarely ❑ 2 = Every once in a while ❑ 3 = Sometimes ❑ 4 = Almost always

13 - PSQI 2: During the past two weeks, how long has it usually taken you to fall asleep each night?

NUMBER OF MINUTES ____________________

14 - PSQI 5a: During the past two weeks, how often have you had trouble sleeping because you cannot get to sleep within 30 minutes

Not at all (0)	Less than once a week (1)	Once or twice a week (2)	Three or more times a week (3)
_______	_______	________	________

Have you had any trouble falling asleep at the beginning of the night?

Right after you go to bed, how long has it been taking you to fall asleep?

How many nights this week have you had trouble falling asleep?

15 - QIDSC, 1. Sleep Onset Insomnia:

0 - Never takes longer than 30 minutes to fall asleep.
1 - Takes at least 30 minutes to fall asleep, less than half the time
2 - Takes at least 30 minutes to fall asleep, more than half the time
3 - Takes more than 60 minutes to fall asleep, more than half the time

16 - PSQI 5b1: During the past two weeks, how often have you had trouble sleeping because you wake up in the middle of the night?

Not at all (0) _______	Less than once a week (1)_______	Once or twice a week (2)________	Three or more times a week (3) ________

During the past two weeks, have you been waking up in the middle of the night?
IF YES: Do you get out of bed? What do you do? (Only go to the bathroom?)

When you get back in bed, are you able to fall right back asleep?

How long do you stay awake?

How many nights this week have you had that kind of trouble?

(IF NO INSOMNIA) Has your sleep been restless or disturbed some nights?

17 - QIDSC, 2. Mid-Nocturnal Insomnia:

0 - Does not wake up at night
1 - Restless, light sleep with few awakenings
2 - Wakes up at least once a night, but goes back to sleep easily
3 - Awakens more than once a night and stays awake for 20 minutes or more, more than half the time

18 - PSQI 5b2: During the past two weeks, how often have you had trouble sleeping because you wake up early in the morning?

Not at all (0) _______	Less than once a week (1)_______	Once or twice a week (2)________	Three or more times a week (3) ________

What time have you been waking up in the morning for the last time, in the past two weeks?
IF EARLY: Is that with an alarm clock, or do you just wake up yourself?
What time do you usually wake up (that is, when you feel well)?
How many mornings this past week have you awakened early? Are you able to go back to sleep?

19 - QIDSC, 3. Early Morning Insomnia:

0 - Less than half the time, awakens no more than 30 minutes before necessary
1 - More than half the time, awakens more than 30 minutes before need be
2 - Awakens at least one hour before need be, more than half the time
3 - Awakens at least two hours before need be, more than half the time

20 - PSQI 7: During the past two weeks, how often have you taken medicine (prescribed or "over the counter") to help you sleep?

Not at all (0) _______ Less than once a week (1)_______ Once or twice a week (2)________ Three or more times a week (3) ________

21- PSQI 6: During the past two weeks, how would you rate your sleep quality overall?

Very good (0) ________ Fairly good (1) ________ Fairly bad (2) _______ Very bad (3) _________

22- CMF4 Depression 4, Insomnia/Hypersomnia: Insomnia or hypersomnia nearly every day during the same 2 week period

What has your sleep been like?

Disturbance in quality of amount of sleep.			**Comments**
**0**	**Sleeping normally every night**		
	**Frequency**	**Intensity**	
**+1/4 or -1/4**	Any	Any sleep disturbance	
**+1/2 or -1/2**			
**+1 or -1**	≥10/14 days	≥ 1 hr/d deviation from normal	
**+1.5 or -1.5**			
**+2 or -2**	≥10/14 days	Sleep increase 50% above normal or decrease 50% below normal.	
**If disturbance is in both directions, assign the sign based on which ever predominates.**			

Combined Sleep Assessment Overview Information:

This assessment tool covers sleep for the **last two weeks**.

Combines ratings for the Clinical Monitoring Form (CMF)[1,2], Quick Inventory of Depressive Symptomatology – Clinician Rated (QIDSC)[3,4], the Pittsburgh Sleep Quality Index (PSQI)[5] and an additional question (12 - KH HS 1) to capture excessive sleepiness[6]

For the CMF, prompts and questions used to guide rating of symptom severity were adapted from:
Clinical Monitoring Form: Users' Guide, Edition 2.1 Gary Sachs, MD and Jennifer Conley, MA. G.S. Sachs, M.D. 2000

For QIDSC, prompts and additional questions used to guide rating of symptom severity were adapted from:
Structured Interview Guide for the Hamilton Depression Scale (SIGH-D) and Inventory of Depressive Symptomatology (IDS-C): (SIGHD-IDSC)[0].

REFERENCES

0. Structured Interview Guide for the Hamilton Depression Scale (SIGH-D) and Inventory of Depressive Symptomatology (IDS-C)[7]: (SIGHD-IDS). Instruments Combined by Kenneth A. Kobak, Janet B.W. Williams, and A. John Rush.

Structured Interview Guide for the Hamilton Depression Rating Scale (SIGH-D). Janet, B.W., Williams, D.S.W.

This interview guide is based on the Hamilton Depression Rating Scale (Hamilton, Max: A rating scale for depression. J Neurol Neurosurg Psychiat 23:56-61, 1960). The anchor point descriptions, with very minor modifications, have been taken from the ECDEU Assessment Manual (Guy, William, ECDEU Assessment Manual for Psychopharmacology, Revised 1976, DHEW Publication No. (ADM) 76-338).

A reliability study of the SIGH-D has been reported (Williams JBW: A structured interview guide for the Hamilton Depression Rating Scale. Archives of General Psychiatry 45:742-747, 1988).

Copyright ©1988, 1992, 1996. All rights reserved. Permission is granted for reproduction for use by researchers and clinicians. For correspondence: Dr. Williams, New York State Psychiatric Institute, Unit 60, 1051 Riverside Drive, New York, New York 10032

1. Sachs GS, Guille C, McMurrich SL. A clinical monitoring form for mood disorders. Bipolar disorders. 2002;4(5):323-7. PubMed PMID: 12479665.
2. Sachs GS, Thase ME, Otto MW, Bauer M, Miklowitz D, Wisniewski SR, Lavori P, Lebowitz B, Rudorfer M, Frank E, Nierenberg AA, Fava M, Bowden C, Ketter T, Marangell L, Calabrese J, Kupfer D, Rosenbaum JF. Rationale, design, and methods of the systematic treatment enhancement program for bipolar disorder (STEP-BD). Biological psychiatry. 2003;53(11):1028-42. PubMed PMID: 12788248.
3. Rush AJ, Bernstein IH, Trivedi MH, Carmody TJ, Wisniewski S, Mundt JC, Shores-Wilson K, Biggs MM, Woo A, Nierenberg AA, Fava M. An evaluation of the quick inventory of depressive symptomatology and the hamilton rating scale for depression: a sequenced treatment alternatives to relieve depression trial report. Biological psychiatry. 2006;59(6):493-501. doi: 10.1016/j.biopsych.2005.08.022. PubMed PMID: 16199008; PMCID: PMC2929841.
4. Trivedi MH, Rush AJ, Ibrahim HM, Carmody TJ, Biggs MM, Suppes T, Crismon ML, Shores-Wilson K, Toprac MG, Dennehy EB, Witte B, Kashner TM. The Inventory of Depressive Symptomatology, Clinician Rating (IDS-C) and Self-Report (IDS-SR), and the Quick Inventory of Depressive Symptomatology, Clinician Rating (QIDS-C) and Self-Report (QIDS-SR) in public sector patients with mood disorders: a psychometric evaluation. Psychol Med. 2004;34(1):73-82. PubMed PMID: 14971628.
5. Buysse DJ, Reynolds CF, 3rd, Monk TH, Berman SR, Kupfer DJ. The Pittsburgh Sleep Quality Index: a new instrument for psychiatric practice and research. Psychiatry research. 1989;28(2):193-213. PubMed PMID: 2748771.
6. Kaplan KA, McGlinchey EL, Soehner A, Gershon A, Talbot LS, Eidelman P, Gruber J, Harvey AG. Hypersomnia subtypes, sleep and relapse in bipolar disorder. Psychol Med. 2015;45(8):1751-63. doi: 10.1017/S0033291714002918. PubMed PMID: 25515854; PMCID: PMC4412779.
7. Rush AJ, Gullion CM, Basco MR, Jarrett RB, Trivedi MH. The Inventory of Depressive Symptomatology (IDS): psychometric properties. Psychol Med. 1996;26(3):477-86. doi: 10.1017/s0033291700035558. PubMed PMID: 8733206.

LW-Sleep: LiveWell Social Rhythm Metric-Trait Version V1
ID:_____________Date:__________Follow-Up Month: _________ Interviewer:_____________

Over the last 2 weeks, which of the following activities occurred at approximately the same time (within ±45 min) at least 3 times per week:

Activity	Activity occurred regularly at the same time (±45 min)		If yes, average number times per week				
1 – SR1<br>Got out of bed	Y	N	3	4	5	6	7
2 – SR2<br>First contact with another person (in person or by phone)	Y	N	3	4	5	6	7
3 – SR3<br>Start work, school, housework, volunteer activities, child or family care	Y	N	3	4	5	6	7
4 – SR4<br>Have dinner	Y	N	3	4	5	6	7
5 – SR5<br>Went to bed	Y	N	3	4	5	6	7

Calculating Regularity Score

The number of activities occurring regularly (i.e., a minimum of three times per week) and at approximately the same time (±45 min) over the past two weeks. Range is 0-5.

Regularity score: ______________

Calculating Frequency Score

If regularity is no, then freq = 0. If regularity is yes, then 3 = 1, 4 = 2, 5 = 3, 6 = 4, 7 = 5.
Range is 0-25.

Frequency score: ______________

REFERENCE

Boland, E. M., et al. (2012). "Life events and social rhythms in bipolar spectrum disorders: an examination of social rhythm sensitivity." J Affect Disord **139**(3): 264-272.

LW-TRQ: LiveWell Tablet Routine Questionnaire V1

ID: ____________ Date: _____________ Follow-Up Month: ____________ Interviewer: ___________

Lead in questions

1 – TRQ1

In the last 2 weeks, did you have any trouble taking all of your psychiatric medications as prescribed?

☐ Yes (1) ☐ No (0)

If yes, what kind of trouble?

2 – TRQ2

In the last 2 weeks, did you stop taking any of your psychiatric medications without your psychiatrist's advice?

☐ Yes (1) ☐ No (0)

If yes, which ones?

3 – TRQ3

In the last 2 weeks, did you change how you are taking any of your psychiatric medications?

☐ Yes (1) ☐ No (0)

If yes, how?

If yes, is this change something you and your psychiatrist discussed?

☐ Yes (1) ☐ No (0)

Quantifying adherence for medication tracker

1) What psychiatric medications have you been prescribed to take in the last 2 weeks?
2) How many times a day have you been prescribed to take (each medication)?

   If more than once a day, at what time of day do you take each dose of (each medication)?

3) What is the dose (each medication) you are prescribed to take?
4) In the last 2 weeks, on how many days did you not take (each dose) of (each medication)? 0-14
5) In the last 2 weeks, on how many days did you take less than prescribed dose of (each medication)?
6) In the last 2 weeks, on how many days did you take additional doses of (each medication)? 0-14
7) In the last 2 weeks, on how many days did you take more than prescribed dose of (each medication)? 0-14

LW-TRQ: LiveWell Tablet Routine Questionnaire V1

ID: _____________ Date: ______________ Follow-Up Month: _____________ Interviewer: ____________

MEDICATION TRACKER

	Start Date	Stop Date	Dose (mg)	When	PRN (T/F)	# Days missed rx dose	# Days less than rx dose	# Days extra rx dose	# Days more than rx dose	% Adher
**Mood stabilizing agents**										
**Antipsychotics**										
**Antidepressants**										

LW-TRQ: LiveWell Tablet Routine Questionnaire V1

ID: ____________ Date: ______________ Follow-Up Month: ____________ Interviewer: ___________

	Start Date	Stop Date	Dose (mg)	When	PRN (T/F)	# Days missed rx dose	# Days less than rx dose	# Days extra rx dose	# Days more than rx dose	% Adher
**Anxiolytics: Benzodiazepines**										
**Anxiolytics: Non-Benzodiazepines**										
**Stimulants**										
**Other**										

When abbreviations: Every day – qd, Every morning – qam, Every evening – qhs, Every afternoon - qnoon

Side Effects

In the last 2 weeks, did you experience any side effects from your psychiatric medications?

☐ Yes (1) ☐ No (0)

If yes, how bothersome or disruptive where they?

___ Mildly = 1, ___ Moderately = 2, ___ Severely = 3

4 – TRQ4 Side Effect Score (0-3): ____________________

Adherence scoring

Calculate the percent adherence for each medication as number of days dose was prescribed (eg 14) minus the number of days in which the medication was not taken or less than the prescribed dose was taken over number of days medication was prescribed (eg 14) then multiple by 100:

Medication Adherence = (Days dose prescribed – Days dose missed)/Days dose prescribed*100

Average Adherence = Sum(Medication Adherence)/Number of Medications*100

This is equivalent to

Calculate the number of doses prescribed for all medications: Number of doses prescribed.

Calculate the number of doses missed for all medications: Number of doses missed.

Average Adherence =

(Number of doses prescribed – Number of doses missed)/Number of doses prescribed*100

Do count taking more than the prescribed frequency or dose in adherence calculation.

Do not include extra doses taken at non-prescribed times in adherence calculation.

Include rx changes agreed upon with psychiatrist (less or more) in adherence calculation.

LW-TRQ: LiveWell Tablet Routine Questionnaire

ID: ____________ Date: _____________ Follow-Up Month: ____________ Interviewer: ___________

Medication List:

**Mood stabilizing agents**	**Antidepressants**	**Anxiolytics: Benzodiazepines**
Lithium *(Lithobid)*	Citalopram *(Celexa)*	Clonazepam *(Klonopin)*
Lamotrigine *(Lamictal)*	Escitalopram *(Lexapro)*	Lorazepam *(Ativan)*
Valproic acid *(Depakote)*	Fluoxetine *(Prozac)*	Diazepam *(Valium)*
Carbamazepine *(Tegretol)*	Sertraline *(Zoloft)*	Alprazolam *(Xanax)*
Oxcarbazepine *(Trileptal)*	Paroxetine *(Paxil)*	**Anxiolytics: Non-Benzodiazepines**
Topiramate *(Topamax)*	Fluvoxamine *(Luvox)*	Eszopiclone *(Lunesta)*
Gabapentin *(Neurontin)*	Vilazadone *(Viibyrd)*	Zaleplon *(Sonata)*
**Antipsychotics**	Vortioxetine *(Brintellix)*	Zolpidem *(Ambien)*
Olanzapine (Zyprexa)	Venlafaxine *(Effexor)*	Buspirone *(Buspar)*
Quetiapine (Seroquel)	Desvenlafaxine *(Pristique)*	Hydroxyzine (*Vistaril)*
Riseperidone *(Risperdal)*	Bupropion *(Wellbutrin)*	**Stimulants**
Aripiprazone (Abilify)	Duloxetine *(Cymbalta)*	Amphetamine *(Adderall, Adderall XR)*
Lurasidone (Latuda)	Levomilnacipran *(Fetzima)*	Dextoamphetamine *(Dexedrine, Vyvanse)*
Ziprasidone (Geodon)	Mirtazapine *(Remeron)*	Methylphenidate *(Ritalin, Focalin, Concerta)*
Paliperidone (Invega)	Trazodone *(Desyrel)*	Atomoxetine *(Stratera)*
Iloperidone (Fanapt)	Nefazodone *(Serzone)*	Modafinil *(Provigil)*
Asenapine (Saphris)	Clomipramine *(Anafranil)*	Armodafinil *(Nuvigil)*
Cariprazine (Vraylar)	Amitriptyline *(Elavil)*	**Antihypertensives**
Brexpiprazole (Rexulti)	Desipramine *(Norpramin)*	Propranolol
Clozapine *(Clozaril)*	Nortriptyline *(Pamelor)*	Clonidine
Haloperidol *(Haldol)*	Doxepin *(Sinequan, Silenor)*	Guanfacine (Intuiv)
Fluphenazine (Prolixin)	Trimipramine *(Surmontil)*	**Hormones**
Chlorpromazine (Thorazine)	Amoxapine *(Asendin)*	Thyroxine
Loxapine (Loxitane)	Imipramine *(Tofranil)*	Levothyroxine
Perphenazine (Trilafon)	Protriptyline *(Vivactil)*	**Substance Use Treatment**
Thioridazine (Mellaril)	Maprotiline *(Ludiomil)*	Disulfiram (Antabuse)
Thiothixene (Navane)	Mianserin *(Norval)*	Acamprosate (Campral)
Trifluoperazine (Stelazine)	Tranylcypromine *(Parnate)*	Naltrexone (ReVia)
**Anticholinergics**	Isocarboxid *(Marplan)*	**Supplements**
Cogentin (*Benztropine*)	Phenelzine *(Nardil)*	Omega-3
	Selegiline *(Emsam)*	**Other**
		Dextromethorphan HBr and Quinidine sulfate (*Nuedexta*)

REFERENCES

1. Depp CA, Lebowitz BD, Patterson TL, Lacro JP, Jeste DV. Medication adherence skills training for middle-aged and elderly adults with bipolar disorder: development and pilot study. Bipolar disorders. 2007;9(6):636-45. doi: 10.1111/j.1399-5618.2007.00397.x. PubMed PMID: 17845279.

2. Sajatovic M, Levin J, Tatsuoka C, Micula-Gondek W, Fuentes-Casiano E, Bialko CS, Cassidy KA. Six-month outcomes of customized adherence enhancement (CAE) therapy in bipolar disorder. Bipolar disorders. 2012;14(3):291-300. doi: 10.1111/j.1399-5618.2012.01010.x. PubMed PMID: 22548902; PMCID: PMC3342843.

3. Scott J, Pope M. Self-reported adherence to treatment with mood stabilizers, plasma levels, and psychiatric hospitalization. The American journal of psychiatry. 2002;159(11):1927-9. doi: 10.1176/appi.ajp.159.11.1927. PubMed PMID: 12411230.

4. Scott J, Pope M. Nonadherence with mood stabilizers: prevalence and predictors. The Journal of clinical psychiatry. 2002;63(5):384-90. doi: 10.4088/jcp.v63n0502. PubMed PMID: 12019661.

LW-QOL: LiveWell WHOQOL-BREF V1

ID: ____________ Date: _____________ Follow-Up Month: ____________ Interviewer:___________

Instructions: Questions are asked on a scale of 1-5 providing answer options at each end of scale. For questions with reversed scoring, ask using regular scale and then reverse score.

The following questions ask how you feel about your quality of life, health, or other areas of your life. I will read out each question to you, along with the response options. **Please choose the answer that appears most appropriate.** If you are unsure about which response to give to a question, the first response you think of is often the best one.

Please keep in mind your standards, hopes, pleasures and concerns. We ask that you think about your life **in the last two weeks.**

		Very dissatisfied	Dissatisfied	Neither satisfied nor dissatisfied	Satisfied	Very satisfied
2.	How satisfied are you with your health?	1	2	3	4	5

		Very poor	Poor	Neither poor nor good	Good	Very good
1.	How would you rate your quality of life?	1	2	3	4	5

The following questions ask about **how much** you have experienced certain things in the last two weeks.

		Not at all	A little	A moderate amount	Very much	An extreme amount
3.	To what extent do you feel that physical pain prevents you from doing what you need to do?	5	4	3	2	1
4.	How much do you need any medical treatment to function in your daily life?	5	4	3	2	1
5.	How much do you enjoy life?	1	2	3	4	5
6.	To what extent do you feel your life to be meaningful?	1	2	3	4	5

		Not at all	A little	A moderate amount	Very much	Extremely
7.	How well are you able to concentrate?	1	2	3	4	5
8.	How safe do you feel in your daily life?	1	2	3	4	5
9.	How healthy is your physical environment?	1	2	3	4	5

The following questions ask about how completely you experience or were able to do certain things in the last two weeks.

		Not at all	A little	Moderately	Mostly	Completely
10.	Do you have enough energy for everyday life?	1	2	3	4	5
11.	Are you able to accept your bodily appearance?	1	2	3	4	5
12.	Have you enough money to meet your needs?	1	2	3	4	5
13.	How available to you is the information that you need in your day-to-day life?	1	2	3	4	5
14.	To what extent do you have the opportunity for leisure activities?	1	2	3	4	5

		Very poor	Poor	Neither poor nor good	Good	Very good
15.	How well are you able to get around?	1	2	3	4	5

		Very dissatisfied	Dissatisfied	Neither satisfied nor dissatisfied	Satisfied	Very satisfied
16.	How satisfied are you with your sleep?	1	2	3	4	5
17.	How satisfied are you with your ability to perform your daily living activities?	1	2	3	4	5
18.	How satisfied are you with your capacity for work?	1	2	3	4	5
19.	How satisfied are you with yourself?	1	2	3	4	5

20.	How satisfied are you with your personal relationships?	1	2	3	4	5
21.	How satisfied are you with your sex life?	1	2	3	4	5
22.	How satisfied are you with the support you get from your friends?	1	2	3	4	5
23.	How satisfied are you with the conditions of your living place?	1	2	3	4	5
24.	How satisfied are you with your access to health services?	1	2	3	4	5
25.	How satisfied are you with your transport?	1	2	3	4	5

The following question refers to how often you have felt or experienced certain things in the last two weeks.

		Never	Seldom	Quite often	Very often	Always
26.	How often do you have negative feelings such as blue mood, despair, anxiety, depression?	5	4	3	2	1

Do you have any comments about the assessment?

____

____

SCORING INSTRUCTIONS

(lowest, highest possible score)	Equations for computing domain scores	Raw score	Transformed scores#	
			4-20	0-100
**Domain 1** (7, 35) **Physical**	Q3* + Q4* + Q10 + Q15 + Q16 + Q17 + Q18<br>D + D + D + D + D + D + D			
**Domain 2** (6,30) **Psychological**	Q5 + Q6 + Q7 + Q11 + Q19 + Q26*<br>D + D + D + D + D + D			
**Domain 3** (3,15) **Relational**	Q20 + Q21 + Q22<br>D + D + D			
**Domain 4** (8,40) **Environmental**	Q8 + Q9 + Q12 + Q13 + Q14 + Q23 + Q24 + Q25<br>D + D + D + D + D + D + D + D			

* Reverse coded in questionnaire above so do not transform (ie 6- question score)

Transformed Scores

(0-20) = Mean(Raw domain score)*4

(0-100) = (Raw domain score – Lowest domain raw score)/(Raw domain score range)*100

REFERENCES

1. Harper A, Power M, Grp W. Development of the World Health Organization WHOQOL-BREF quality of life assessment. Psychological Medicine. 1998;28(3):551-8. PubMed PMID: WOS:000073793600006.

LW-LIFE: LiveWell Longitudinal Interval Follow-Up Evaluation

ID: ______________ Date: ______________ Follow-Up Month: ____________ Interviewer:_______________

Overview:

1) Prepare for the interview by

a. For baseline assessment, review the MINI and ADE in particular identify the last prior episode as you will need to follow back to the most recent prior episode to determine the current clinical status. Also see baseline follow back algorithm at the end of this document.

b. For ongoing assessment, study previous LW-LIFE interviews and CMF clinical status ratings in particular be sure to know the clinical status at that last assessment as you will need to follow back to this status to determine the current clinical status.

2) Start by completing the combined mood assessment for last 2 weeks to assess current status then follow back to last assessment or at baseline to last prior episode. The combined mood assessment contains the CMF questions

2) Obtain an overview of what has happened to the participant since the time of the last interview. This overview serves as a time to reacquaint (or acquaint) the subject and interviewer while providing information on whether the subject has recovered or relapsed.

3) Help participant recall state at last interview. Start by asking whether participant was ever back to or remained as usual self, continue to probe until find best/worst level(s) of recovery and/or decline.

4) Once best/worst levels established, ask about details of what happened between last follow-up and time the participant was feeling best or worst. Try to determine when participant reached each level of and how long remained there. Identify bipolar disorder clinical status and psychiatric status rating.

5) Assess clinical course by inquiring about "change points" in clinical state using probes to determine when participant was in each clinical state. When probing for change points in clinical status, try to relate change points to other events (i.e. holidays, birthdays, other dated events).

6) If the occurrence of a new mood episode is established, return to probes to determine course.

On the following pages are suggested probes to guide the interview. Use the CMF to assess severity of symptoms when changes in clinical status occur or are suspected to have occurred.

Questions for this interview refer only to time period for this interview as defined below.

This interview covers (week of month/year): from _____________ to _____________

A. Interval Course for Bipolar Disorder

THE LAST TIME WE SPOKE TOGETHER YOU WERE (description of subject's condition at that time; e.g. "you were feeling very depressed and had trouble sleeping," "You were feeling well," etc.)

How have things been since then?

____

____

____

____

____

____

____

____

____

____

____

____

Review course of symptom severity for bipolar disorder.

Use specific probes in the next sections as a guide and the Clinical Monitoring Form (CMF) to determine clinical status and establish psychiatric symptom ratings.

Did you begin to feel better or worse or have you remained about the same?

☐ No change ☐ Feels better ☐ Feels worse

Status Prompts

Depression: Since we last spoke, has there been any time that lasted two weeks when nearly every day you felt depressed, sad, blue, moody, or down most of the day?

Anhedonia: Since we last spoke, has there been any time that lasted two weeks when nearly every day you didn't care anymore, didn't really enjoy anything, or lost interest in your activities or things you usually enjoy?

Elevation: Since we last spoke, has there been a time that lasted at least a week (or resulted in your being hospitalized) when you were feeling too good/cheerful/high, excited?

Energy: Since we last spoke, has there been a time that lasted at least a week (or resulted in your being hospitalized) when you were so hyper that you or other people thought you were not your normal self? Or feeling so hyper that you got into trouble?

Irritability: Since we last spoke, has there been a time that lasted at least a week (or resulted in your being hospitalized) when you were so irritable that you found yourself shouting at people or starting fights of arguments?

Anxiety: Since we last spoke, has there been a time were you abnormally anxious? What about keyed up or tense, unusually restless, difficulty concentrating due to worries, feeling something awful may happen, feeling you might lose control, fidgety, agitated, or hard to stay still. Accompanied by mood symptoms?

Timing Prompts

When did that start?

When did it get better?

Where there any other times?

No change

- If prior clinical status recovered, use screening prompts below to review and assure continuing as recovered.
- If screening prompts positive or prior clinical status not recovered, review symptoms of depression/mania and determine clinical status and score severity using the CMF.
- If current clinical status concordant with last clinical status:
  - Review to assure no change in clinical status during time interval for this interview.
- If current clinical status not concordant with last clinical status:
  - Review how current clinical status is different (e.g. "Last time we spoke together, you had the following symptoms [list from prior cmf] now you have [list from current cmf]")
  - Probe for change points (e.g. "When do you think you began to feel better/worse").
  - Complete better or worse as appropriate.

<u>Worse</u>

- When did you begin to feel worse (week of month/year)? _____________
- Do you think this was just a continuation of your former problems? ☐ no ☐ yes
    - What symptoms were you experiencing?

      ____
      ____
      ____
      ____
      ____

    - Did you have any of the following symptoms during this time [review symptoms of depression/mania and score severity using CMF]?
- Did this present a new period of difficulty for you? ☐ no ☐ yes
    - What symptoms were you experiencing? Which were new or worse?

      ____
      ____
      ____
      ____
      ____

    - Did you have any of the following symptoms during this time [review symptoms of depression/mania also anxiety and score severity using CMF]?
- Did you ever have a time when you were a little bit better even though you had number of symptoms? ☐ no ☐ yes
    - When did that happen (week of month/year)? ____________
    - What symptoms got better?

      ____
      ____
      ____
      ____
      ____

    - Did you have any of the following symptoms at this time [review symptoms of depression/mania also anxiety and score severity using CMF]?

Better

- When did you begin to feel better (week of month/year)? ____________
- Did you ever get to a point where you were totally free of all symptoms? ☐ no ☐ yes
  - o When did that happen (week of month/year)? ____________
  - o Did you have any of the following symptoms at this time [review symptoms of depression/mania also anxiety and score severity using CMF]?
- Did you ever get to a point where you had only one or two symptoms that did not bother you very much? ☐ no ☐ yes
  - o When did that happen (week of month/year)? ____________
  - o What were those symptoms?

    ____
    ____
    ____
    ____
    ____
  - o Did you have any of the following symptoms at this time [review symptoms for depression/mania, score severity using CMF]?
- Did you ever get to a point where you felt much better, although you had some symptoms that really bothered you? ☐ no ☐ yes
  - o When did that happen (week of month/year)? ____________
  - o What were those symptoms?

    ____
    ____
    ____
    ____
  - o Did you have any of the following symptoms at this time [review symptoms for depression/mania also anxiety, score severity using CMF]?
- Did you return to feeling ill? ☐ no ☐ yes
  - o When did you return to feeling ill (week of month/year)? ____________
  - o Follow up to determine status during this period and additional change points.

Reliability of Interval Course for Bipolar Disorder CMF Clinical Status ________

Reliability of Interval Course for Bipolar Disorder CMF Anxiety ________

1 = very good , 2 = good, 3 = fair, 4 = poor, 5 = very poor

B. Suicidality

Ideation

- Have you had any thoughts of wanting to die or kill yourself? ☐ 0 no ☐1 yes
  - Number of times: _________

	When	Severity
- Week of month/year	_____________	_____________
- Week of month/year	_____________	_____________
- Week of month/year	_____________	_____________
- Week of month/year	_____________	_____________

1- Wish to be Dead:
Did you wish you were dead or wish you could go to sleep and not wake up?

2- Suicidal Thoughts without Method:
Did you actually have any thoughts of killing yourself?

3- Suicidal Thoughts with Method:
Did you think about how you might kill yourself?

4- Suicidal Intent without Specific Plan:
Did you have these thoughts and have some intention of acting on them?

5- Suicidal Intent with Specific Plan and Intent:
Did you start to work out or worked out the details of how to kill yourself?
Did you intend to carry out this plan?

If suicidal ideation score is not zero, go to the SI Protocol.

Attempts

- Have you made any attempts to harm or kill yourself? ☐ 0 no ☐1 yes
    - Number of attempts: _________

	When	Actual/Potential Lethality
- Week of month/year	____________	____________
- Week of month/year	____________	____________

- o Circumstances of each suicide attempt: Circumstances

- Week of month/year	____________	____________
- Week of month/year	____________	____________

Actual Lethality/Medical Damage or Potential Lethality

1 - No physical damage (e.g. held pills in hand). Behavior not likely to result in injury.

2 - No physical damage. Behavior likely to result in injury but not likely to cause death.

3 - No physical damage. Behavior likely to result in death despite available medical care.

4 - Minor physical damage (e.g. surface scratches).

5 - Moderate physical damage/Medical attention needed (e.g. conscious but sleepy, somewhat responsive; second-degree burns, bleeding of major vessel).

6 - Moderately severe physical damage/Medical hospitalization and likely intensive care required (e.g., comatose with reflexes intact; third-degree burns less than 20% of body; extensive blood loss but can recover; major fractures).

7 - Severe physical damage/Medical hospitalization with intensive care required (e.g., comatose without reflexes, third degree burns over 20% of body, extensive blood loss with unstable vital signs, major damage to a vital area, respiratory arrest or prolonged coma)

Circumstances: Intoxicated, Delusional, Hallucinating, Confused or Disorganized, Significant Life Event.

If suicide attempt score is not zero, go to the SI Protocol.

Reliability of Interval Course for Suicidality ________

1 = very good , 2 = good, 3 = fair, 4 = poor, 5 = very poor

C. Screen for (New and) Psychotic Symptoms

- Have you been bothered by any symptoms that you haven't had before? ☐ no ☐ yes
  - Brief description of the difficulty.

    ____
    ____
    ____
    ____
    ____

- Delusions (Determine if in context of a mood episode or not) ☐ no ☐ yes
  - Did you ever believe people were plotting against you, spying on you, or trying to hurt you?
  - Did you ever believe someone was reading your mind or you could read their mind?
  - Did you ever believe thoughts were being put in your mind or that you were being sent special messages?
  - Brief description of the symptoms.

    ____
    ____
    ____
    ____

- Hallucinations (Determine if in context of a mood episode or not) ☐ no ☐ yes
  - Did you ever hear voices that other people couldn't hear?
  - Did you ever see things that other people couldn't see?
  - Did you ever smell things that other people couldn't smell?
  - Brief description of the symptoms.

    ____
    ____
    ____
    ____

Reliability of Interval Course for Psychotic Symptoms ________

1 = very good, 2 = good, 3 = fair, 4 = poor, 5 = very poor

D. Interval Course for Substance Use

1. Have you been drinking coffee or other caffeinated beverages? ☐ no ☐ yes
   [estimate a cup as 6 ounces of coffee, 4 cups coffee = 10 colas = 2 energy shot drinks]

   - How many cups a day?
   - Was this pretty consistent over the whole time since we last spoke?
   - Caffeine _______ cups/day (record change points as needed)

2. Have you been smoking cigarettes, cigars, a pipe, or chewing tobacco? ☐ no ☐ yes
   [pack is 20-25 cigarettes, 1 pipe/cigar/chew = 5 cigarettes, ecigs?]

   - How many per day?
   - Was this pretty consistent over the whole time since we last spoke?
   - Nicotine _______ packs/day (record change points as needed)

In the next section, I'd like to ask you a few questions that relate to your experience with alcohol and other drugs. Some of the substances we'll talk about are prescribed by a doctor (like pain medications). But I will only record those if you have taken them for reasons or in doses other than prescribed. I'll also ask you about illicit or illegal drug use.

3. Have you been drinking alcohol? ☐ no ☐ yes

[For the purpose of this interview, a standard drink is equal to 12 oz can/bottle of beer, 5 oz glass of wine, 1 shot of liquor or mixed drink]

- How many drinks per day?
- How many days per week?
- How many drinks per week?
- For men, how often have you had 5 or more drinks per day?
- For women, how often have you had 4 or more drinks per day?
- Was this pretty consistent over the whole time since we last spoke?
- Alcohol _______ drinks per day (record change points as needed)

  _______ days per week

  _______ drinks per week (estimate from drinks/day, days/week)

  _______ number of heavy drinking episodes per week (4+/3+ for men/women)

4. Have you been smoking marijuana or eating any products containing cannabis? ☐ no ☐ yes

- o How many times a week?
- o Was this pretty consistent over the whole time since we last spoke?
- o Cannabis _______ times/week (record change points as needed)

5. Have you been using prescription drugs without a prescription or for reasons or in doses other than prescribed? ☐ no ☐ yes

[Prescription Stimulant (e.g. Ritalin, Adderall), Anxiolytic or sleeping pills (e.g. Valium, Xanax), Prescription Opioid (e.g. Hydorcodone, Vicodin, OxyContin, Opana)]

- o How many times a week?
- o Was this pretty consistent over the whole time since we last spoke?
- o Type _______________ _______ times/week (record change points as needed)

6. Have you been using illegal drugs? ☐ no ☐ yes

[Cocaine (coke, crack), Amphetamine (speed, ice, molly, ecstasy), Cathinone (bath salts), Hallucinogen (LSD, mushrooms), Opioid (heroin, opium), PCP (angel dust), Ketamine (special K)]

- o How many times a week?
- o Was this pretty consistent over the whole time since we last spoke?
- o Type _______________ _______ times/week (record change points as needed)

Reliability of Interval Course for Substance Use = ________

1 = very good , 2 = good, 3 = fair, 4 = poor, 5 = very poor

E. Interval Course for Life Events

Have you experienced any major life events, either positive or negative, that might have impacted you? ☐ no ☐ yes

Record if it seems important to the patient. Be sure to assess positive and negative events.

If yes, describe what and when:

To what extent did it affect you? ☐ not at all ☐ a little bit ☐ somewhat ☐ quite a bit ☐ very much so*

(*recommend change scale to: very negatively, negatively, somewhat negatively, neither negatively or positively, somewhat positively, positively, very positively; EHG 02/07/21)

Life Event Prompts

Work: Any change in your work situation?
- job, work conditions or hours, promotion/demotion, fired/retired, troubles at work, new opportunity, new project

Education: Any changes in school or educational efforts?
- start/stop school, change schools, failure/success, exam preparation, new project

Finance: Any changes in finances?
- difficulties, burdens, substantial improvements

Health: Have you experienced any major physical illness, injury, or accident? Other significant health changes?

Bereavement: Have you experienced the death or loss of a close friend or relative?

Housing: Have you had any changes in your housing situation?
- foreclosure, mortgage, loan, move

Legal: Have you had any legal problems?

Family: Have you had any major changes in your family situation?
- children born/leaving/returning/problems, spousal employment, parents/grandparents, serious arguments, engagements

Social: Have you had any major changes in your relationships with your friends or other supports?

Relational: Have you had any major changes in your dating, partner, or marital relationships?
- divorce, separation, marriage, pregnancy, engagement

Reliability of Interval Course of Life Events ________

1 = very good , 2 = good, 3 = fair, 4 = poor, 5 = very poor

F. Interval Course for Medical Information

1. What is your current weight? ________________ lbs

Has this been relatively stable since our last interview? (Compare with last interview)

Calculate BMI (Use height from baseline interview) ___________

2. Onset of Menses: ___________ _______________________

Onset of Menses: ___________ _______________________

Onset of Menses: ___________ _______________________

Date first day of menstruation or 'NA' if not applicable and why (male, post-menopausal, surgical or pharmacological suppression, pregnant, underweight) also code early, late, regular, irregular.

Reliability of Interval Course for Medical Information ________

1 = very good , 2 = good, 3 = fair, 4 = poor, 5 = very poor

G. Interval Course of Mental Health Treatment

1a. Did you have any appointments with your psychiatrist? ☐ no ☐ yes

If yes, frequency (e.g. weekly, monthly): ___________

If yes, number of appointments: _________

If yes, when (only if monthly or less): __________ __________ __________ __________

1b. Did you miss any appointments with your psychiatrist? ☐ no ☐ yes

If yes, # of missed appointments:____________

2a. Did you have any appointments with a therapist? ☐ no ☐ yes

If yes, frequency (e.g. weekly, monthly): ___________

If yes, number of appointments: _________

If yes, when (only if monthly or less): __________ __________ __________ __________

2b. Did you miss any appointments with your therapist? ☐ no ☐ yes

If yes, # of missed appointments:____________

3. Have you had any other psychiatric care/treatment in addition to what we just talked about?

If yes, describe:

☐ Light therapy, ☐ Support group (DBSA, NAMI), ☐ Group therapy, ☐ ECT,

☐ Intensive outpatient, ☐ Residential, ☐ ER visit, ☐ Hospitalization, ☐ Other: _____________

Reliability of Interval Course for Mental Health Treatment ________

1 = very good , 2 = good, 3 = fair, 4 = poor, 5 = very poor

H. Interval Course for Psychiatric Medications

1. Start by reviewing each psychiatric medication being taken by participant now (last 2 weeks from current assessment) and at the last LIFE interview to assess for changes.

    a. If unchanged, verify, no change in prescribed medications nor how taking during the interval.

    b. If changed prescription or adherence, identify change points in prescription and/or adherence.

2. Track changes using the medication tracking form (LW_LIFE_MedicationsTracker) for each period between change points. Record the start and stop dates of medications, dosing (amount and when taken each day) and average weekly adherence measures.

Medication prompts

- What psychiatric medications are you prescribed now?
- What is the dose prescribed for (each medication)?
- How many times a day have you been prescribed to take (each medication)?
- If more than once a day, at what time of day do you take each dose of (each medication)?
-

Timing prompts

- Did any of the psychiatric medications you are prescribed change?
- Did the prescribed dose of any of your medications change?
- Did how often you are prescribed to take any of your medications change?
- Are you prescribed to take any new or additional psychiatric medications?

Adherence prompts

- o Did you have any trouble taking all of your psychiatric medications as prescribed?
  □ Yes □ No If yes, what kind of trouble?

- o Did you take any of your psychiatric medications in ways other than prescribed?
  □ Yes (1) □ No (0) If yes, how?

  If yes, is this change previously agreed on with psychiatrist to do as needed?
  □ Yes (1) □ No (0) [If yes, do not count as more than rx in medication tracker]

- o Did you stop taking any of your psychiatric medications without your psychiatrist's advice?
  □ Yes □ No If yes, which ones?

Quantifying adherence

During an average week

- o On how many days did you not take (each dose) of (each prescribed psychiatric medication)?

- o On how many days did you take less than the prescribed dose of your psychiatric medication?

- o On how many days did you take an extra dose of your prescribed psychiatric medication?

- o On how many days did you take more than the prescribed dose of your psychiatric medication?

Adherence calculation

Calculate the percent adherence for each medication as the number of days per week in which the medication was not taken or less than the prescribed dose was taken.
If large changes in adherence, attempt to identify change point and record change in adherence.
Do count taking more than the prescribed frequency or dose as non-adherence.

Reliability of Interval Course for Psychiatric Medications ________

Reliability of Interval Course for Psychiatric Medication Adherence ________

1 = very good , 2 = good, 3 = fair, 4 = poor, 5 = very poor

MEDICATION TRACKER											
	Start Date	Stop Date	Dose (mg)	When	PRN (T/F)	Avg # Days missed rx per wk	Avg # Days less than rx per wk	Avg # Days extra rx per wk	Avg # Days more than rx per wk	Avg % Adher per wk	Rel Score
**Mood stabilizing agents**											
**Antipsychotics**											
**Antidepressants**											

LW-LIFE: LiveWell Longitudinal Interval Follow-Up Evaluation

ID: _______________ Date: _______________ Follow-Up Month: _____________ Interviewer:________________

	Start Date	Stop Date	Dose (mg)	When	PRN (T/F)	Avg # Days missed rx per wk	Avg # Days less than rx per wk	Avg # Days extra rx per wk	Avg # Days more than rx per wk	Avg % Adher per wk	Rel Score
**Anxiolytics: Benzodiazepines**											
**Anxiolytics: Non-Benzodiazepines**											
**Stimulants**											
**Other**											

When abbreviations: Every day–qd, Every morning–qam, Every evening–qhs, Every afternoon–qnoon

Medication List

Mood stabilizing agents

Lithium *(Lithobid)*
Lamotrigine *(Lamictal)*
Valproic acid *(Depakene, Depakote)*
Carbamazepine *(Tegretol, Equetro)*
Oxcarbazepine *(Trileptal)*
Topiramate *(Topamax)*
Gabapentin *(Neurontin)*

Antipsychotic

Olanzapine (Zyprexa)
Quetiapine (Seroquel)
Riseperidone *(Risperdal)*
Aripiprazone (Abilify)
Lurasidone (Latuda)
Ziprasidone (Geodon)
Paliperidone (Invega)
Iloperidone (Fanapt)
Asenapine (Saphris)
Clozapine *(Clozaril)*
Haloperidol *(Haldol)*
Fluphenazine (Prolixin)
Chlorpromazine (Thorazine)
Loxapine (Loxitane)
Perphenazine (Trilafon)
Thioridazine (Mellaril)
Thiothixene (Navane)
Trifluoperazine (Stelazine)
Cariprazine (Vraylar)
Brexpiprazole (Rexulti)

Antidepressants

Citalopram *(Celexa)*
Escitalopram *(Lexapro)*
Fluoxetine *(Prozac)*
Sertraline *(Zoloft)*
Paroxetine *(Paxil)*
Fluvoxamine *(Luvox)*
Vilazadone *(Viibyrd)*
Vortioxetine *(Brintellix)*
Venlafaxine *(Effexor)*
Desvenlafaxine *(Pristique)*
Bupropion *(Wellbutrin)*
Duloxetine *(Cymbalta)*
Levomilnacipran *(Fetzima)*
Mirtazapine *(Remeron)*
Trazodone *(Desyrel, Oleptro)*
Nefazodone *(Serzone)*
Clomipramine *(Anafranil)*
Amitriptyline *(Elavil)*
Desipramine *(Norpramin)*
Nortriptyline *(Pamelor)*

Antidepressants Continued

Doxepin *(Sinequan, Silenor)*
Trimipramine *(Surmontil)*
Amoxapine *(Asendin)*
Imipramine *(Tofranil)*
Protriptyline *(Vivactil)*
Maprotiline *(Ludiomil)*
Mianserin *(Norval)*
Tranylcypromine *(Parnate)*
Isocarboxid *(Marplan)*
Phenelzine *(Nardil)*
Selegiline *(Emsam)*

Anxiolytics: Benzodiazepines

Clonazepam *(Klonopin)*
Lorazepam *(Ativan)*
Diazepam *(Valium)*
Alprazolam *(Xanax)*

Anxiolytics: Non-Benzodiazepines

Eszopiclone *(Lunesta)*
Zaleplon *(Sonata)*
Zolpidem *(Ambien)*
Buspirone *(Buspar)*
Hydroxyzine (*Vistaril)*

Stimulants

Amphetamine *(Adderall, Adderall XR)*
Dextoamphetamine *(Dexedrine, Dextrostat, Vyvanse)*
Methylphenidate *(Ritalin, Focalin, Concerta)*
Atomoxetine *(Stratera)*
Modafinil *(Provigil)*
Armodafinil *(Nuvigil)*

Antihypertensive

Propranolol
Clonidine
Guanfacine (Intuiv)

Hormone

Thyroxine
Levothyroxine

Addiction

Disulfiram (Antabuse)
Acamprosate (Campral)
Naltrexone (ReVia)

Supplement

Omega-3

Other

Cogentin (Benztropine)
Nuedexta

Baseline Follow Back Algorithm for Assessment of Initial Clinical Status

No additional weeks of data necessary prior to baseline Week minus 7

If Clinical Status at Week minus 7 is:

1) Depression
2) Mania
3) Hypomania
4) Mixed
5) Continued Symptomatic
6) Recovered
7) Prodromal with symptom count ≥ 3 or impairment severity ≥ 1
8) Recovering and continue Recovering to Week 0 (that is in episode at week minus 8)
9) Recovering and convert to Episode prior to Week 0
10) Recovering and convert to Continued Symptomatic prior to Week 0

Additional weeks of data necessary prior to baseline Week minus 7

If Clinical Status at Week minus 7 is:

1) Prodromal with 2 new symptoms and symptom count ≤ 3 and impairment severity ≤ 1
   - Go back as far as necessary to demonstrate onset of 2 new symptoms while Recovered
2) Recovering and convert to Recovered prior to Week 0
   - Go back as far as necessary to demonstrate Recovering for 8 consecutive weeks

Study Week Number Assignment Algorithm

1) If assessment occurs Monday to Wednesday, assign last week of assessment as prior week.

2) If assessment occurs Thursday to Sunday, assign last week of assessment as current week.

3) Week 0 is the final week of the baseline assessment (ie, baseline is the 8 weeks prior to the study start week (Weeks -7 to 0)).

4) Study start week is Week 1.

Note: To align with behavioral data, study assessment weeks are always considered to start on a Monday and end on a Sunday.

REFERENCES

Keller MB, Lavori PW, Friedman B, Nielsen E, Endicott J, McDonald-Scott P, et al. The Longitudinal Interval Follow-up Evaluation. A comprehensive method for assessing outcome in prospective longitudinal studies. Archives of general psychiatry. 1987 Jun;44(6):540-8. PMID: 3579500.

**LW-CMF: LiveWell Clinical Monitoring Form V1**
ID:_____________Date:_____________Follow-Up Month: ____________ Interviewer:_____________

Major Depressive Episode:

Depression 1. Depressed Mood, Severity (DSM A): Depressed mood most of the day, nearly every day during the same 2 week period

Did you feel down or depressed?

Persistently feels "depressed", "sad", "down", "blue" or equivalent dysphoria.			Comments
**0**	**Not depressed**		
	**Frequency**	**Intensity**	
**+1/4**	Any	Any dysphoria	
**+1/2**			
**+1**	≥10/14 days	Depressed dysphoric mood<br>Most of the day	
**+1.5**			
**+2**	≥10/14 days	Constant unremitting intense dysphoria	

Depression 2. Decreased Interest, Severity (DSM B): Markedly diminished interest or pleasure in all or almost all activities most of the day, nearly every day during the same 2 week period

Have you been able to enjoy pleasant things that happened?

Loss of motivation or connectedness with others, loss of interest in or diminished capacity for enjoyment of pleasurable activities, push self to work or activities.			Comments
**0**	**Enjoys activities as usual**		
	**Frequency**	**Intensity**	
**-1/4**	Any	Any decreased interest	
**-1/2**			
**-1**	≥10/14 days	Loss of interest or enjoyment in most things<br>Disinterest decreased motivation but able to enjoy some activities under favorable conditions<br>Most of the day	
**-1.5**			
**-2**	≥10/14 days	Much less interested, Emotionally constricted<br>Can't cry, no response to favorable stimuli<br>Most of the day	

Depression 4. Insomnia/Hypersomnia: Insomnia or hypersomnia nearly every day during the same 2 week period

What has your sleep been like?

Disturbance in quality or amount of sleep.			**Comments**
**0**	**Sleeping normally every night**		
	**Frequency**	**Intensity**	
**+1/4 or -1/4**	Any	Any sleep disturbance	
**+1/2 or -1/2**			
**+1 or -1**	≥10/14 days	≥ 1 hr/d deviation from normal	
**+1.5 or -1.5**			
**+2 or -2**	≥10/14 days	Sleep increase or decrease 50% above normal	
**If disturbance in both directions, select based on whichever predominates.**			

Range: Min _____ Max _____ Avg _____ hrs; Range When Well: Min _____ Max _____ Avg _____ hrs

DFA: *Have you had difficulty falling asleep?* _____ (Y/N)

MCA: *Have you been waking up in the middle of the night?* _____

EMA: *Have you experienced early morning awakenings?* _____

EBT: *Have you had to go to bed earlier than usual?* _____

DGOOB: *Have you had difficulty getting out of bed in the morning?* _____

Naps: *Have you been napping*? _____

Depression 6. Fatigue/Loss of Energy: Fatigue or loss of energy nearly every day during the same 2 week period

How was your energy level? Were there things which you should have done and didn't because you didn't have enough energy or were simply too tired? For example? (Not due lack of motivation)

Fatigue, decreased energy, feels tired or tires easily.			**Comments**
**0**	**Usual energy level.**		
	**Frequency**	**Intensity**	
**-1/4**	Any	Carries out all activities<br>Occasionally tired, some tasks are harder<br>More drained than usual	
**-1/2**			
**-1**	≥10/14 days	Interferes with some activities at work, home, socially (Not due to a lack of motivation)	
**-1.5**			
**-2**	≥10/14 days	Lethargic, Stays in bed	

Depression 5b. Psychomotor retardation: Psychomotor retardation nearly every day during the same 2 week period

Were there times you were moving or thinking more slowly than usual? If I had been with you, would I have noticed something was wrong?

Slowness of thought and speech, impaired ability to concentrate, decreased motor activity.			Comments
**0**	**No evidence of motor, speech, or cognitive slowing.**		
	**Frequency**	**Intensity**	
**+1/4**	Any	Subjective slowing of thoughts, speech, or movement or rare objective evidence of retardation	
**+1/2**			
**+1**	≥10/14 days	Slowness thought or movement observable by others<br>Increased speech latency	
**+1.5**			
**+2**	≥10/14 days	Apparent on interview	

Depression 5a. Psychomotor agitation: Psychomotor agitation nearly every day during the same 2 week period

Were there times you were so fidgety or agitated it was hard for you to stay still?

Fidgetiness, playing with hands, hair, etc. Moving about, can't sit still, purposeless activity.			Comments
**0**	**No evidence of motor agitation.**		
	**Frequency**	**Intensity**	
**+1/4**	Any	Restlessness, fidgeting, purposeless movement, pacing	
**+1/2**			
**+1**	≥10/14 days	Difficulty remaining still or purposeless movement observed by others	
**+1.5**			
**+2**	≥10/14 days	Pacing, unable to sit still when necessary	

Depression 3. Appetite/Weight: Significant weight loss when not dieting or weight gain or decrease or increase in appetite nearly every day during the same 2 week period

How was your appetite?

Disturbance of appetite, ↑/↓ from normal.			Comments
**0**	**Normal appetite/weight**		
	**Frequency**	**Intensity**	
**+1/4 or -1/4**	Any	Mild or rare ↑/↓ in appetite	
**+1/2 or -1/2**			
**+1 or -1**	≥10/14 days	Reduced or increased consumption about 25%	
		Needs encouragement to eat	
		Craving food or seeks snacks in addition to usual meals	
**+1.5 or -1.5**			
**+2 or -2**	≥10/14 days	Weight loss or gain ≥ 5% body in 2 weeks	
		Decrease or increase 50% of normal consumption	
**Count all days with appetite disturbance and use +/- to indicate predominant direction**			

Depression 8. Concentration/Indecisiveness: Diminished ability to think or concentrate, or indecisiveness, nearly every day during the same 2 week period

How was your concentration?

Inability to concentrate, inability to focus on a task, difficulty making decisions.			Comments
**0**	**Normal concentration.**		
	**Frequency**	**Intensity**	
**-1/4**	Any	Rare or limited to unpleasant, very difficult tasks	
**-1/2**			
**-1**	≥10/14 days	Difficult to read or collect thoughts in conversation	
		Difficult to function in role at home/work	
**-1.5**			
**-2**	≥10/14 days	Clear cognitive impairment during casual interaction	
		Unable to function in role at home/work	

Depression 7a. Guilt: Feelings of excessive or inappropriate guilt nearly every day during the same 2 week period

Were there times you were down on yourself? Did you feel as if you were a bad person or that you deserved to suffer? (Not limited inability function due to illness)

Self-reproach, feels let people down, present illness is a punishment. Delusions of guilt, hears accusatory voices, threatening visual hallucinations.			Comments
**0**	**No excessive self-blame or guilty preoccupation.**		
	**Frequency**	**Intensity**	
**+1/4**	Any	Mild, rare self-deprecatory thoughts	
**+1/2**			
**+1**	≥10/14 days	Self-deprecatory thoughts (Not limited inability fx due illness)<br>Guilt or ruminations over past errors/sinful deeds	
**+1.5**			
**+2**	≥10/14 days	Self-deprecatory thoughts (Not limited inability fx due illness)<br>Guilt or ruminations over past errors/sinful deeds<br>Most of the day	

Depression 7b. Worthlessness: Feelings of worthlessness nearly every day during the same 2 week period

Was your self-esteem or self-confidence down compared to usual?

Feels inferior, defective, incompetent, inadequate			Comments
**0**	**Normal self-esteem/self-confidence**		
	**Frequency**	**Intensity**	
**-1/4**	Any	Felt mildly down on self, lacking self-confidence	
**-1/2**			
**-1**	≥10/14 days	Feels inferior to most others<br>Stops work or social activities due to expectation of failure	
**-1.5**		Worthlessness	
**-2**	≥10/14 days	Delusional	

Depression 9. Suicidal Ideation (SI): Recurrent thoughts of death, recurrent suicidal ideation without a specific plan, or a suicide attempt or a specific plan for committing suicide during the same 2 week period

Were there times you were feeling so bad that you felt life was not worth living? What about actually thinking about suicide or harming yourself?

Weary of life, would be better off dead, morbid preoccupation, thoughts of harming self, plans for self-destruction, urge to end life			**Comments**
**0**	**No SI.**		
	**Frequency**	**Intensity**	
**+ 1/4**	Rare	Fleeting LNWL, Fleeting passive SI	
**+ 1/2**	Several days	Fleeting LNWL or fleeting passive or active SI	
		Persistent periods of passive SI	
**+1**	≥ 10/14 days	Most of the day with LNWL	
		Persistent periods of passive SI	
	Several days	Brief active SI	
**+1.5**	> 1 day	Persistent periods of active SI	
**+2**	≥ 10/14 days	Most of the day with active SI	
	Any	Active SI with intent, plan, or action	
		Active SI unable to control thoughts impulses	
**IF CMF #9 SI SYMPTOM SCORE ≥ 1, GO TO SI PROTOCOL**			

LNWL: Life not worth living.

Passive SI: Wish to die or kill self but no method, no intent, no plan.

Active SI: Wish to kill self with method but no intent, no plan.

Fleeting: < 1 minute

Brief: 1-15 minutes

Persistent: ≥ 15 minutes

Depression 10. Impairment: The depressive symptoms cause at least moderate impairment in social, occupational, or other important areas of functioning.

Have your depressive symptoms caused any major changes or problems in your

- sleep, eating, or hygiene and grooming?

- interactions with other such as withdrawal or conflicts?

- family responsibilities?

- work, school, or volunteer responsibilities?

			Comments
**0**	**No significant distress or impairment**		
	**Frequency**	**Intensity**	
**+1/4**	Any		
**+1/2**			
**+1**	≥10/14 days	Poorly groomed, moderately disheveled	
		Conflicts with others	
		Decreased function in role at home or work	
		Social withdrawal	
**+1.5**			
**+2**	≥10/14 days	Completely unkempt, disheveled	
		Inability to function in role at home or work	
		Social isolation	
		Life-threatening behaviors	

Symptoms possibly consistent with marked or severe impairment: Fatigue/Energy, Psychomotor retardation/agitation, Concentration/Indecisiveness, Worthlessness, Suicidal ideation.

Manic/Hypomanic Episode:

Mania 1a. Elevated/Expansive, Severity (DSM A): Distinct period of elevated or expansive mood during the same 1 week period

Did you feel so good that people thought you were not your normal self? Felt very good, too cheerful, high, optimistic attitude out of proportion to circumstances?

			Comments
**0**	**No mood elevation or expansiveness.**		
	**Frequency**	**Intensity**	
**+1/4**	Any	Any elevation/expansiveness not clearly related to events	
**+1/2**			
**+1**	≥4/7 days<br>or hospitalized	Feeling so good high, excited or optimistic people thought not normal self<br>Abnormally persistently elevated, expansive, euphoric, excessively cheerful, high, “on top of the world”	
**+1.5**		Clearly elated, exalted expressions.	
**+2**	≥4/7 days	Psychotic features such as belief in divine powers	

Mania 1b. Elevated/Expansive, DSM Consecutive Days:
How many consecutive days with elevated/expansive mood of intensity >= 1? ______ # of days (0-7 days)

Mania 2a. Irritable, Severity (DSM B): Distinct period of irritable mood during the same 1 week period

Were you so irritable that you shouted at people or started fights or arguments?

			Comments
**0**	**No irritability**		
	**Frequency**	**Intensity**	
**+1/4**	Any	Any irritability not clearly related to events	
**+1/2**			
**+1**	≥4/7 days<br>or hospitalized	Clearly abnormal irritability with behavioral manifestations (e.g. starts fights/arguments)	
**+1.5**			
**+2**	≥4/7 days	Psychotic features such as paranoia	

Mania 2b. Irritable, DSM Consecutive Days:
How many consecutive days with irritable mood of intensity >= 1? ______ # of days (0-7 days)

Mania 8c1. Increased energy, Severity (DSM4 A, DSM5 C): Increased energy during the same 1 week period

Did you feel so hyper that people thought you were not your normal self, or were you so hyper you got in trouble? Did you have more energy than usual to do things? Did it seem like too much energy?

<table>
<tr><th colspan="3">Unusually energetic more active than usual self without expected fatigue.</th><th>Comments</th></tr>
<tr><td>0</td><td colspan="2">No abnormally increased energy</td><td rowspan="11"></td></tr>
<tr><td></td><td>Frequency</td><td>Intensity</td></tr>
<tr><td>+1/4</td><td>Any</td><td>Slightly more energetic</td></tr>
<tr><td>+1/2</td><td></td><td>Little change in activity level but less fatigued than usual</td></tr>
<tr><td rowspan="4">+1</td><td rowspan="4">≥4/7 days
or hospitalized</td><td>Hyper and not their normal self.</td></tr>
<tr><td>So hyper got into trouble or observed manic behavior.</td></tr>
<tr><td>Unusual behavior but didn't get into trouble due to not observed</td></tr>
<tr><td>Somewhat more active than usual with little or no fatigue</td></tr>
<tr><td>+1.5</td><td></td><td></td></tr>
<tr><td>+2</td><td>≥4/7 days</td><td>Much more active than usual, unusually active all day long with little or no fatigue</td></tr>
</table>

Mania 8c2. Increased Energy, DSM Consecutive Days:
How many consecutive days with increased energy of intensity >= 1? _____ # of days (0-7 days)

Mania 8a1. Goal Directed Activity, Severity (6a, DSM5 C): Increase in goal directed activity during the same 1 week period

Did you have an increase in activity either socially, sexually, at work or school? Were you so active that people worried about you taking on so much? Did you find you were so active that you really didn't get much done?

<table>
<tr><th colspan="3">Plans, projects, purposeful activities</th><th>Comments</th></tr>
<tr><td>0</td><td colspan="2">No increase in goal directed behavior</td><td rowspan="8"></td></tr>
<tr><td></td><td>Frequency</td><td>Intensity</td></tr>
<tr><td>+1/4</td><td>Any</td><td>Initiated one or more new projects</td></tr>
<tr><td>+1/2</td><td></td><td></td></tr>
<tr><td rowspan="2">+1</td><td rowspan="2">≥4/7 days
or hospitalized</td><td>Multiple creative, self-improvement, home projects w/out external requirements</td></tr>
<tr><td>New projects require commitment of > 8hrs/wk or > 5% of income</td></tr>
<tr><td>+1.5</td><td></td><td></td></tr>
<tr><td>+2</td><td>≥4/7 days</td><td>Work effort > 10hrs/day or work after 9 pm</td></tr>
</table>

Mania 8a2. Goal Directed Activity, DSM Consecutive Days:
How many consecutive days with increased goal directed activity of intensity >= 1? _____ # of days (0-7 days)

Mania 8b1. Psychomotor Agitation, Severity (6b): Increase in psychomotor agitation during the same 1 week period

Were there times you were so fidgety or agitated it was hard for you to stay still? Were you physically or mentally restless?

Fidgetiness, playing with hands, hair, etc. Moving about, can't sit still. Purposeless non-goal-directed activity.			Comments
**0**	**No evidence of motor agitation**		
	**Frequency**	**Intensity**	
**+ 1/4**	Any	Restless, fidgets	
**+ 1/2**			
**+1**	≥4/7 days<br>or hospitalized	Difficulty remaining still or purposeless movement observed by others	
**+1.5**			
**+2**	≥4/7 days	Pacing, unable to sit still when necessary	

Mania 8b2. Psychomotor Agitation, DSM Consecutive Days:
How many consecutive days with psychomotor agitation of intensity >= 1? _____ # of days (0-7 days)

Mania 4. Need for Sleep (2): Decreased need for sleep during the same 1 week period

Were there nights when you got less sleep than usual and found you didn't really miss it?
(Do not count simple insomnia)

Amount of sleep, subjective need for sleep, ability to function.			Comments
**0**	**Usual need for sleep**		
	**Frequency**	**Intensity**	
**- 1/4**	Any	Mild ↓; no impact on function	
**- 1/2**			
**-1**	≥4/7 days	Sleep reduced ≥ 1.5 hrs without impact on next day function	
**-1.5**			
**-2**	≥4/7 days	Sleep reduced by >5hrs from usual or sleeping <2hrs/day	

Mania 3. Self-Esteem/Grandiosity (1): Inflated self-esteem or grandiosity during the same 1 week period

Were there times when you were feeling more self-confident than usual? Were there times when you were feeling more special, more talented, more attractive or smarter than usual? Were there any times when your thoughts were grandiose?

Inflated self-confidence, feels more attractive, more talented, able to do more than usual.			Comments
**0**	**No increase in self-esteem**		
	**Frequency**	**Intensity**	
**+1/4**	Any	Some exaggerated sense of abilities	
**+1/2**			
**+1**	≥ 4/7 days	Clearly inflated estimate of capabilities	
		Actual performance may be increased but assessment excessively positive	
**+1.5**		Grossly excessive ideas of worth or abilities	
**+2**	≥4/7 days	Delusional	

Mania 5. Talking (3): More talkative than usual or pressure to keep talking during the same 1 week period

Were there times when you were more talkative than usual, or you found you said more than you intended? Were there times that you spoke much faster than usual?

Amount and rate of speech.			Comments
**0**	**Normal rate and quantity of speech**		
	**Frequency**	**Intensity**	
**+1/4**	Any	Others note talkative, not out of character/bothersome	
**+1/2**			
**+1**	≥4/7 days	Others complain about excessive talking, uncharacteristically cutting others off	
		Pressured speech (described or observed)	
		Conversation seeking	
		Communication reveals more than intended	
**+1.5**			
**+2**	≥4/7 days	Hard for others to get a word in	
		Virtually incessant talking	

Mania 6. FOI/Racing Thoughts (4): Flight of ideas or subjective experience that thoughts are racing during the same 1 week period

Did you find that you had more ideas than usual? Were there times when your thoughts seemed to be racing through your head?

Racing thoughts, ↑ in train of productive, novel, unrelated ideation.			Comments
**0**	**No racing thoughts**		
	**Frequency**	**Intensity**	
**+1/4**	Any	Mild thinking fast	
**+1/2**			
**+1**	≥4/7 days	Ideas race, come tumbling out	
		Rapid train of thoughts	
**+1.5**			
**+2**	≥4/7 days	Speech cannot keep up w/ pressured thoughts.	

Mania 7. Distractibility (5): Distractibility during the same 1 week period

Did you find you were easily distracted? Is it hard to focus in the first place or attention easily draw away but other thoughts or things going on around you?

Unable to maintain focus of attention, easily distracted by external or internal stimuli.			Comments
**0**	**No evidence of distractibility.**		
	**Frequency**	**Intensity**	
**+1/4**	Any	Generally able to maintain focus	
**+1/2**			
**+1**	≥4/7 days	Decreased ability to complete tasks due to distractibility	
		Able to complete tasks but requires great effort	
**+1.5**			
**+2**	≥4/7 days	Obvious in most conversations	
		Can’t stay on topic to complete most sentences thoughts	

Mania 9. High Risk Behavior (7): Excessive involvement in pleasurable activities that have a high potential for painful consequences during the same 1 week period

Did you do anything that was unusual for you or that other people might think was excessive, foolish, or risky? Did you do anything which would have caused a problem if you were caught?

Excessive, foolish, risky activities that could have serious consequences for self/others (whether or not caught)			**Comments**
**0**	**No risk taking.**		
	**Frequency**	**Intensity**	
**+1/4**	Any	Mild risk taking/increased pleasure seeking	
**+1/2**			
**+1**	≥4/7 days	Exhibits behavior others would recognize as foolish/risky/excessive	
		Gambling, sex, investments, reckless driving with negative consequences	
		Definitely hazardous physical, financial or social behavior	
**+1.5**			
**+2**	≥4/7 days	Extremely hazardous physical, financial or social behavior	

Mania 10. Hospitalization: Hospitalized due to symptoms of mania?

No (0) _____ Yes (1) _____

Mania 11. Psychosis: Psychotic or delusional symptoms of mania?

No (0) _____ Yes (1) _____

Mania 12. Impairment: Mania - Manic symptoms severe enough to cause at least moderate impairment in occupational functioning or in usual social activities or relationships with others. **Hypomania -** Manic symptoms not severe enough to cause moderate impairment in social or occupational functioning. However, an unequivocal change in functioning uncharacteristic of person when not symptomatic is present and the disturbance in mood and change in functioning are observable by others.

Have your manic symptoms caused any major changes or problems in your

- *eating, or hygiene and grooming?*
- *interactions with other such as associating with strangers, or conflicts?*
- *sexual activity, spending habits, or substance use?*
- *family responsibilities?*
- *work, school, or volunteer responsibilities?*

Have your manic symptoms resulted in any legal problems?
Have your manic symptoms resulted in any physical altercations or violence?

			Comments
**0**	**No significant distress or impairment**		
	**Frequency**	**Intensity**	
**+1/4**	Any		
**+1/2**			
**+1**	≥4/7 days	Poorly groomed, moderately disheveled	
		Conflicts with others, associating with strangers	
		Decreased function in role at home or work	
		Hazardous, physical, financial or social behavior	
**+1.5**			
**+2**	≥4/7 days	Completely unkempt, disheveled, bizarre garb	
		Inability to function in role at home or work	
		Extremely hazardous physical, financial or social behavior	
		Violence, legal problems	

Symptoms possibly consistent with marked or severe impairment criteria for PSR: Elevated/Expansive, Irritable, Self-Esteem/Grandiosity, Psychomotor agitation, Distractible, High Risk Behavior.

Anxiety:

Anxiety 1. Abnormally Anxious: Abnormally anxious nearly every day most of the day

Were you abnormally anxious?

			**Comments**
**0**	**None.**		
	**Frequency**	**Intensity**	
**+1/4**	Any	Keyed up tense, restless.	
**+1/2**			
**+1**	Majority of days of most recent episode (mania, hypomania, depression)	Keyed up or tense, unusually restless, difficulty concentrating due to worries, feel something awful may happen, feel might lose control (mild-moderate,2-3 symptoms)	
**+1.5**		(moderate-severe,4-5 symptoms)	
**+2**	Majority of days of most recent episode (mania, hypomania, depression)	Severely keyed up or tense, unusually restless, difficulty concentrating due to worries, feel something awful may happen, feel might lose control (severe, 4-5 symptoms + motor agitation)	

Symptom Severity Generic Anchors:

	0<br>None or usual	
**Decreased**	From Balanced or Euthymic State	**Increased**
-1/4	Questionable, slight, or rare symptom<br>Occurred once or twice, not clinically significant	+1/4
-1/2	**Mild**<br>Clearly present symptom but subthreshold DSM criteria	+1/2
-1	**Moderate**<br>Clearly present symptom and fulfills DSM criteria	+1
-1.5	**Marked**	+1.5
-2	**Severe**<br>Extreme symptom, need not be worst imaginable	+2
	Nearly every day (≥ 70% of days)<br>for depressive symptoms ≥ 10/14 days for depression or ≥ 5/7 days for mixed episode<br>for manic symptoms ≥ 7 consecutive days for mania or ≥ 4 consecutive days for hypomania	
	Fleeting < 1 minute, Brief 1-15 minutes, Persistent ≥ 15 minutes	

Clinical Status, Symptom Severity and Psychiatric Status Rating Reliability = _______

1 = very good 2 = good 3 = fair 4 = poor 5 = very poor

Symptom severity ratings for the LiveWell Clinical Monitoring Form (CMF)[1,2] are guided by prompts and adapted from: Clinical Monitoring Form: Users' Guide, Edition 2.1 Gary Sachs, MD and Jennifer Conley, MA. G.S. Sachs, M.D. 2000.

REFERENCES

1. Sachs GS, Guille C, McMurrich SL. A clinical monitoring form for mood disorders. Bipolar disorders. 2002;4(5):323-7. PubMed PMID: 12479665.
2. Sachs GS, Thase ME, Otto MW, Bauer M, Miklowitz D, Wisniewski SR, Lavori P, Lebowitz B, Rudorfer M, Frank E, Nierenberg AA, Fava M, Bowden C, Ketter T, Marangell L, Calabrese J, Kupfer D, Rosenbaum JF. Rationale, design, and methods of the systematic treatment enhancement program for bipolar disorder (STEP-BD). Biological psychiatry. 2003;53(11):1028-42. PubMed PMID: 12788248.

LW-CMF Scoring: LiveWell Clinical Monitoring Form DSM4 Scoring V1

ID:________________ Date:________________ Follow-Up Month:________________ Interviewer:_______________

DSM4 Symptom Scoring:

Start Date/Week _____________

Stop Date/Week _____________

Current or Follow-Back

	Symptom Severity														
	< < < DECREASED < < <					WELL	> > > INCREASED > > >								
	Severe	Marked	Moderate	Mild		None		Mild	Moderate	Marked	Severe	Symptom Count	Symptom Criteria	Entry Criteria	# of Consecutive Days
**DEPRESSION**															
**1. Depressed mood (A)**	-	-	-	-	-	0	+1/4	+1/2	+1	+1.5	+2				-
**2. Decreased interest (B)**	-2	-1.5	-1	-1/2	-1/4	0	-	-	-	-	-				-
**4. Insomnia/Hypersomnia**	-2	-1.5	-1	-1/2	-1/4	0	+1/4	+1/2	+1	+1.5	+2			-	-
**6. Fatigue/Energy**	-2	-1.5	-1	-1/2	-1/4	0	-	-	-	-	-			-	-
**5b. Psychomotor retardation**	-	-	-	-	-	0	+1/4	+1/2	+1	+1.5	+2			-	-
**5a. Psychomotor agitation**	-	-	-	-	-	0	+1/4	+1/2	+1	+1.5	+2			-	-
**3. Appetite/Weight**	-2	-1.5	-1	-1/2	-1/4	0	+1/4	+1/2	+1	+1.5	+2			-	-
**8. Concentration/Indecisiveness**	-2	-1.5	-1	-1/2	-1/4	0	-	-	-	-	-			-	-
**7a. Guilt**	-	-	-	-	-	0	+1/4	+1/2	+1	+1.5	+2			-	-
**7b. Self-esteem/Worthless**	-2	-1.5	-1	-1/2	-1/4	0	-	-	-	-	-			-	-
**9. Suicidal ideation**	-	-	-	-	-	0	+1/4	+1/2	+1	+1.5	+2			-	-
**10. Impairment**	-	-	-	-	-	0	+1/4	+1/2	+1	+1.5	+2	-	-		-
**Depression Symptom Criteria Sum**	-	-	-	-	-	-	-	-	-	-	-	-		-	-
**MANIA**															
**1. Elevated/Expansive (A)**	-	-	-	-	-	0	+1/4	+1/2	+1	+1.5	+2		-		
**2. Irritable (B)**	-	-	-	-	-	0	+1/4	+1/2	+1	+1.5	+2		-		
**8c. Increased energy (C)**	-	-	-	-	-	0	+1/4	+1/2	+1	+1.5	+2	-	-	-	
**8a. Goal directed activity (C, 6a)**	-	-	-	-	-	0	+1/4	+1/2	+1	+1.5	+2			-	
**8b. Psychomotor agitation (6b)**	-	-	-	-	-	0	+1/4	+1/2	+1	+1.5	+2			-	-
**4. Need for sleep (2)**	-2	-1.5	-1	-1/2	-1/4	0	-	-	-	-	-			-	-
**3. Self Esteem (1)**	-	-	-	-	-	0	+1/4	+1/2	+1	+1.5	+2			-	-
**5. More talkative (3)**	-	-	-	-	-	0	+1/4	+1/2	+1	+1.5	+2			-	-
**6. FOI/Racing thoughts (4)**	-	-	-	-	-	0	+1/4	+1/2	+1	+1.5	+2			-	-
**7. Distractible (5)**	-	-	-	-	-	0	+1/4	+1/2	+1	+1.5	+2			-	-
**9. High risk behavior (7)**	-	-	-	-	-	0	+1/4	+1/2	+1	+1.5	+2			-	-
**10. Hospitalized due to mania**	-	-	-	-	-	-	-	-	-	-	-	-	-		-
**11. Psychosis**	-	-	-	-	-	-	-	-	-	-	-	-	-		-
**12. Impairment**	-	-	-	-	-	0	+1/4	+1/2	+1	+1.5	+2	-	-		-
**Mania Symptom Criteria Sum**	-	-	-	-	-	-	-	-	-	-	-	-		-	-

**Symptom Count Sum**	-	-	-	-	-	-	-	-	-	-	-		-	-	-

Symptom count sum: If |severity| ≥ 1 enter rounded up severity score otherwise enter 0. For symptoms with a and b use highest score.
Symptom criteria: If |severity| ≥ 1 enter 1 else enter 0. For symptoms with a and b use highest score.
Symptom and impairment entry criteria: If |severity| ≥ 1 enter 1 otherwise enter 0.
Hospitalized and psychosis entry criteria: If present, enter 1 otherwise enter 0.
# of Consecutive days: Enter number of consecutive days with |severity| ≥ 1; if 7 or more enter 7.

LW-CMF Scoring: LiveWell Clinical Monitoring Form DSM4 Scoring V1

ID:_______________ Date:_______________ Follow-Up Month:_______________ Interviewer:______________

CURRENT CLINICAL STATUS	
Depression	
Mania	
Hypomania	
Mixed	
Continued Symptomatic	
Prodromal	
Recovering	
Recovered	

PRIOR CLINICAL STATUS

Type ___________________

Duration ___________________

CURRENT PSR __________

RELIABILITY __________

Use: 1 = very good, 2 = good, 3 = fair

Drop: 4 = poor, 5 = very poor

PSYCHIATRIC STATUS RATING			
**Clinical Status**	**Score**	**Rating**	**Definition**
Depression, Mania, Mixed	6	Severe Episode	psychotic symptoms or severe impairment
	5	Episode	no psychotic symptoms and no severe impairment
Hypomania, Continued Symptomatic, Prodromal	4	Marked Symptoms	symptom count sum > 2 and marked or severe impairment
	3	Moderate Symptoms	symptom count sum > 2 or moderate impairment
Symptomatic Recovery, Recovering, Prodromal	2	Residual or Prodromal Symptoms	symptom count sum > 0 and ≤ 2 and no moderate, marked, or severe impairment
Asymptomatic Recovery, Recovering	1.5	Mild Symptoms	≥ 1 mild symptom and no moderate, marked, or severe symptoms and no moderate, marked, or severe impairment
	1	No Symptoms	no mild, moderate, marked, or severe symptoms and no impairment

DSM4 Clinical Status Coding

Clinical Status	Entry criteria met?	Symptom Criteria	Impairment	Consecutive Days	CSC	PSR
Mania	Yes	Three if elevated, four if only irritable	≥ 1 or hospitalized or psychosis	≥ 7 or hospitalized	6	5-6
Depression	Yes	Five	≥ 1	≥ 10/14	7	5-6
Mixed	Yes, mania and depression#	Both mania and depression#	≥ 1	≥ 7#	8	5-6
Hypomania	Yes	Three if elevated, four if only irritable	< 1 and not hospitalized and no psychosis	≥ 4	5	3

#Mania with concurrent depression for 1 week. Count depressive symptoms for 5/7 days instead of 10/14.

Not In Episode

Clinical Status	Recovered from last acute episode?	Symptom Count Sum/Impairment Severity	CSC	PSR
Continued Sx	No	Symptom Count Sum > 2 or Impairment Severity ≥ 1	4	3-4
Prodromal	Yes	Symptom Count Sum > 2 or new✓ or Impairment Severity ≥ 1	3	2-4
Recovering	No, recovering ≤ 8 consecutive weeks	Symptom Count Sum ≤ 2 and Impairment Severity < 1	2	1-2
Symptomatic Recovery	Yes, after recovering > 8 consecutive weeks	Symptom Count Sum > 0 and ≤ 2 and Impairment Severity < 1	1	2
Asymptomatic Recovery	Yes, after recovering > 8 consecutive weeks	Symptom Count Sum 0 and Impairment Severity < 1	1	< 2

✓Two NEW moderate, marked, or severe symptoms developed while in recovery.

LW-CMF Scoring: LiveWell Clinical Monitoring Form DSM5 Scoring V1

ID:________________ Date:________________ Follow-Up Month:________________ Interviewer:_______________

**DSM5 Symptom Scoring:**<br>Start Date/Week _____________<br>Stop Date/Week _____________<br>Current or Follow-Back	**Symptom Severity**											Symptom Count	Symptom Criteria	Entry Criteria	# of Consecutive Days
	**< < < DECREASED < < <**					**WELL**	**> > > INCREASED > > >**								
	Severe	Marked	Moderate	Mild		None		Mild	Moderate	Marked	Severe				
**DEPRESSION**															
**1. Depressed mood (A)**, DNM	-	-	-	-	-	0	+1/4	+1/2	+1	+1.5	+2				-
**2. Decreased interest (B)**, DNM	-2	-1.5	-1	-1/2	-1/4	0	-	-	-	-	-				-
**4. Insomnia/Hypersomnia**	-2	-1.5	-1	-1/2	-1/4	0	+1/4	+1/2	+1	+1.5	+2			-	-
**6. Fatigue/Energy**, DNM	-2	-1.5	-1	-1/2	-1/4	0	-	-	-	-	-			-	-
**5b. Psychomotor retardation**, DNM	-	-	-	-	-	0	+1/4	+1/2	+1	+1.5	+2			-	-
**5a. Psychomotor agitation**	-	-	-	-	-	0	+1/4	+1/2	+1	+1.5	+2			-	-
**3. Appetite/Weight**	-2	-1.5	-1	-1/2	-1/4	0	+1/4	+1/2	+1	+1.5	+2			-	-
**8. Concentration/Indecisiveness**	-2	-1.5	-1	-1/2	-1/4	0	-	-	-	-	-			-	-
**7a. Guilt**	-	-	-	-	-	0	+1/4	+1/2	+1	+1.5	+2			-	-
**7b. Self-esteem/ Worthless**, DNM	-2	-1.5	-1	-1/2	-1/4	0	-	-	-	-	-			-	-
**9. Suicidal ideation**, DNM	-	-	-	-	-	0	+1/4	+1/2	+1	+1.5	+2			-	-
**10. Impairment**	-	-	-	-	-	0	+1/4	+1/2	+1	+1.5	+2	-	-		-
**Depression Symptom Criteria Sum**	-	-	-	-	-	-	-	-	-	-	-	-		-	-
**MANIA**															
**1. Elevated/Expansive (A)**, MND	-	-	-	-	-	0	+1/4	+1/2	+1	+1.5	+2		-		
**2. Irritable (B)**	-	-	-	-	-	0	+1/4	+1/2	+1	+1.5	+2		-		
**8c. Increased energy (C)**, MND	-	-	-	-	-	0	+1/4	+1/2	+1	+1.5	+2		-		
**8a.Goal directed activity (C, 6a)** MND	-	-	-	-	-	0	+1/4	+1/2	+1	+1.5	+2				
**8b. Psychomotor agitation (6b)**	-	-	-	-	-	0	+1/4	+1/2	+1	+1.5	+2			-	-
**4. Need for sleep (2)**, MND	-2	-1.5	-1	-1/2	-1/4	0	-	-	-	-	-			-	-
**3. Self Esteem (1)**, MND	-	-	-	-	-	0	+1/4	+1/2	+1	+1.5	+2			-	-
**5. More talkative (3)**, MND	-	-	-	-	-	0	+1/4	+1/2	+1	+1.5	+2			-	-
**6. FOI/Racing thoughts (4)**	-	-	-	-	-	0	+1/4	+1/2	+1	+1.5	+2			-	-
**7. Distractible (5)**	-	-	-	-	-	0	+1/4	+1/2	+1	+1.5	+2			-	-
**9. High risk behavior (7)**, MND	-	-	-	-	-	0	+1/4	+1/2	+1	+1.5	+2			-	-
**11. Hospitalized due to mania**	-	-	-	-	-	-	-	-	-	-	-	-	-		-
**12. Psychosis**	-	-	-	-	-	-	-	-	-	-	-	-	-		-
**13. Impairment**	-	-	-	-	-	0	+1/4	+1/2	+1	+1.5	+2	-	-		-
**Mania Symptom Criteria Sum**	-	-	-	-	-	-	-	-	-	-	-	-		-	-
**Symptom Count Sum**	-	-	-	-	-	-	-	-	-	-	-		-	-	-

Symptom count sum: If |severity| ≥ 1 enter rounded up severity score otherwise enter 0. For symptoms with a and b use highest score.
Symptom criteria: If |severity| ≥ 1 enter 1 otherwise enter 0. For symptoms with a and b use highest score.
Symptom and impairment entry criteria: If |severity| ≥ 1 enter 1 otherwise enter 0.
Hospitalized and psychosis entry criteria: If present, enter 1 otherwise enter 0.
# of Consecutive days: Enter number of consecutive days with |severity| ≥ 1; if 7 or more enter 7.

LW-CMF Scoring: LiveWell Clinical Monitoring Form DSM5 Scoring V1

ID:_______________ Date:_______________ Follow-Up Month:_______________ Interviewer:______________

CURRENT CLINICAL STATUS	
Depression	
Mania	
Hypomania	
Mixed	
Continued Symptomatic	
Prodromal	
Recovering	
Recovered	

PRIOR CLINICAL STATUS

Type ___________________

Duration ___________________

CURRENT PSR __________

RELIABILITY __________

Use: 1 = very good, 2 = good, 3 = fair

Drop: 4 = poor, 5 = very poor

PSYCHIATRIC STATUS RATING

Clinical Status	Score	Rating	Definition
Depression, Mania, Mixed	6	Severe Episode	psychotic symptoms or severe impairment
	5	Episode	no psychotic symptoms and no severe impairment
Hypomania, Continued Symptomatic, Prodromal	4	Marked Symptoms	symptom count sum > 2 and marked or severe impairment
	3	Moderate Symptoms	symptom count sum > 2 or moderate impairment
Symptomatic Recovery, Recovering, Prodromal	2	Residual or Prodomal Symptoms	symptom count sum > 0 and ≤ 2 and no moderate, marked, or severe impairment
Asymptomatic Recovery, Recovering	1.5	Mild Symptoms	≥ 1 mild symptom and no moderate, marked, or severe symptoms and no moderate, marked, or severe impairment
	1	No Symptoms	no mild, moderate, marked, or severe symptoms and no impairment

DSM5 Clinical Status Coding

Clinical Status	Entry criteria met?	Symptom Criteria	Impairment	Consecutive Days	CSC	PSR
Mania	Yes	Three if elevated, four if only irritable	≥ 1 or hospitalized or psychosis	≥ 5/7 or hospitalized	6	5-6
Depression	Yes	Five	≥ 1	≥ 10/14	7	5-6
Mixed	Mania with mixed features: Mania and 3 non-overlapping symptoms (DNM)				9	5-6
	Depression with mixed features: Depression and 3 non-overlapping symptoms (MND)				11	
	Hypomania with mixed features: Hypomania and 3 non-overlapping symptoms (DNM)				10	3
Hypomania	Yes	Three if elevated, four if only irritable	< 1 and not hospitalized and no psychosis	≥ 3/4*	5	3

Not In Episode				
**Clinical Status**	**Recovered from last acute episode?**	**Symptom Count**	**CSC**	**PSR**
Continued Sx	No	Symptom Count Sum > 2 or Impairment Severity ≥ 1	4	3-4
Prodromal	Yes	Symptom Count Sum > 2 or new✓ or Impairment Severity ≥ 1	3	2-4
Recovering	No, recovering ≤ 8 consecutive weeks	Symptom Count Sum ≤ 2 and Impairment Severity < 1	2	1-2
Symptomatic Recovery	Yes, after recovering > 8 consecutive weeks	Symptom Count Sum > 0 and ≤ 2 and Impairment Severity < 1	1	1-2
Asymptomatic Recovery	Yes, after recovering > 8 consecutive weeks	Symptom Count Sum 0 and Impairment Severity < 1	1	< 2

✓Two NEW moderate, marked, or severe symptoms developed while in recovery.

* Note LW CMF V1 requires ≥ 5/7 for moderate symptom severity forcing DSMV Hypomania to require 4 consecutive days

LW-SMS: LiveWell Symptom Management Scale V1

ID: ____________ Date: _____________ Follow-Up Month: ____________ Interviewer: ___________

Instructions: Read the prompt and ask the following questions.

Mania

Imagine you are experiencing ongoing or new low level manic symptoms. I am going to read you a list of things you might do. Think about how often you might engage in each behavior.

Please respond with "Never", "Sometimes", "Often", or "Always". Would you…

Mania	Never	Sometimes	Often	Always
1 Engage in calming activities	0	1	2	3
2 Make sure you do not overwork	0	1	2	3
3 Enjoy the feeling of high	0	1	2	3
4 Monitor your mood or action	0	1	2	3
5 Go on as if nothing happened, hoping the symptoms will go away	0	1	2	3
6 Drink to keep going	0	1	2	3
7 Take extra time to rest	0	1	2	3
8 Prioritize and reduce the number of your tasks	0	1	2	3
9 Go out and spend money	0	1	2	3
10 Seek professional help (e.g. call your psychiatrist)	0	1	2	3
11 Tell yourself you are getting into dangerous situations	0	1	2	3
12 Continue to move about and take on more tasks	0	1	2	3
13 Spend time on your own to avoid stimulation	0	1	2	3
14 Modify excessive behavior and restrain yourself	0	1	2	3
15 Find more to do to fill out the extra minutes of the day	0	1	2	3
16 Delay impulsive actions	0	1	2	3
17 Talk to someone to bring some reality into your worries	0	1	2	3
18 Lose your temper easily	0	1	2	3
19 Take extra medication as previously agreed with the doctor	0	1	2	3

Depression

Imagine you are experiencing ongoing or new low level depressive symptoms. I am going to read you a list of things you might do. Think about how often you might engage in each behavior.

Please respond with "Never", "Sometimes", "Often", or "Always". Would you…

Depression	Never	Sometimes	Often	Always
1 Distract yourself from negative thoughts by doing things	0	1	2	3
2 Exercise or keep fit	0	1	2	3
3 Stay in bed and hope it will go away	0	1	2	3
4 Get yourself organized and keep busy	0	1	2	3
5 Just tolerate it and go on as if nothing is happening	0	1	2	3
6 Seek professional help (e.g. call your psychiatrist)	0	1	2	3
7 Maintain a routine	0	1	2	3
8 Take extra medications without prescription	0	1	2	3
9 Get social support and meet people	0	1	2	3
10 Recognize unrealistic thoughts and evaluate if worth worrying about	0	1	2	3

Scoring Instructions

Add together the values for the following questions in each category to calculate the score for that section.

Mania

A. Positive Coping for Mania (12): 1, 2, 4, 7, 8, 10, 11, 13, 14, 16, 17, 19

B. Negative Coping for Mania (7): 3, 5, 6, 9, 12, 15, 18

Depression

C. Positive Coping for Depression (7): 1, 2, 4, 6, 7, 9, 10

D. Negative Coping for Depression (3): 3, 5, 8

Items (Range of Item Summed Values)	Sum of Values	Reverse Code	Score	Range
A. Positive Coping for Mania (0-36)		1		0-36
B. Negative Coping for Mania (0-21)		-1		-21-0
C. Positive Coping for Depression (0-21)		1		0-21
D. Negative Coping for Depression (0-9)		-1		-9-0

Item (Range of Item Summed Values)	Score
E. Positive Coping Score [add A+C] (0-57)	
F. Negative Coping Score [add B+D] (-30-0)	
Total Coping Score [E+F] (-30-57)	

Total Coping Score can range from -30 (all negative coping) to 57 (all positive coping) where a score of 0 represents either equal intensity use of positive and negative coping strategies assessed or no use of coping strategies assessed.

REFERENCES

1. Lam D, Wong G. Prodromes, coping strategies and psychological interventions in bipolar disorders. Clinical psychology review. 2005;25(8):1028-42. doi: 10.1016/j.cpr.2005.06.005. PubMed PMID: 16125292.

2. Lam DH, Bright J, Jones S, Hayward P, Schuck N, Chisholm D, Sham P. Cognitive therapy for bipolar illness - A pilot study of relapse prevention. Cognitive Therapy and Research. 2000;24(5):503-20. doi: 10.1023/a:1005557911051. PubMed PMID: WOS:000088877400002.

3. Wong G, Lam D. The development and validation of the coping inventory for prodromes of mania. J Affect Disord. 1999;53(1):57-65. PubMed PMID: 10363667.

4. Lam DH, Watkins ER, Hayward P, Bright J, Wright K, Kerr N, Parr-Davis G, Sham P. A randomized controlled study of cognitive therapy for relapse prevention for bipolar affective disorder: outcome of the first year. Archives of general psychiatry. 2003;60(2):145-52. PubMed PMID: 12578431.
