# Supplementary material for: A Smartphone-Based Self-management Intervention for Individuals With Bipolar Disorder (LiveWell): Empirical and Theoretical Framework, Intervention Design, and Study Protocol for a Randomized Controlled Trial
Source: JMIR Res Protoc. 2022 Feb 21;11(2):e30710. doi: 10.2196/30710 (PMC8902672; doi:10.2196/30710)
Supplement: Multimedia Appendix 8 [file resprot_v11i2e30710_app8.pdf]

PDOQ

Please complete the survey below.

Thank you!

The questions in this section are about taking medications.

Please consider only the medications prescribed by your psychiatrist for daily use to treat bipolar disorder. This does not include medications taken for other medical reasons (e.g. high blood pressure or diabetes), nor does it include medications that are prescribed on an 'as needed' basis.

1) When I take my medications regularly I feel good about my relationships.

- ◯ Strongly Disagree
- ◯ Disagree
- ◯ Somewhat Disagree
- ◯ Neither Agree Nor Disagree
- ◯ Somewhat Agree
- ◯ Agree
- ◯ Strongly Agree

2) My risk for mood episodes is high enough to offset the trouble of taking medications.

- ◯ Strongly Disagree
- ◯ Disagree
- ◯ Somewhat Disagree
- ◯ Neither Agree Nor Disagree
- ◯ Somewhat Agree
- ◯ Agree
- ◯ Strongly Agree

3) My goal is to learn and do what I need to so I can take my medications as prescribed.

- ◯ Strongly Disagree
- ◯ Disagree
- ◯ Somewhat Disagree
- ◯ Neither Agree Nor Disagree
- ◯ Somewhat Agree
- ◯ Agree
- ◯ Strongly Agree

4) I know the basics about bipolar disorder and medications for bipolar disorder.

- ◯ Strongly Disagree
- ◯ Disagree
- ◯ Somewhat Disagree
- ◯ Neither Agree Nor Disagree
- ◯ Somewhat Agree
- ◯ Agree
- ◯ Strongly Agree

5) I take medications to reduce my risk of being hospitalized.

- ◯ Strongly Disagree
- ◯ Disagree
- ◯ Somewhat Disagree
- ◯ Neither Agree Nor Disagree
- ◯ Somewhat Agree
- ◯ Agree
- ◯ Strongly Agree

6) Problems caused by my mood episodes are bad enough for me to take medications.

- ◯ Strongly Disagree
- ◯ Disagree
- ◯ Somewhat Disagree
- ◯ Neither Agree Nor Disagree
- ◯ Somewhat Agree
- ◯ Agree
- ◯ Strongly Agree

7) The unpleasant effects of medications are always present.

- ◯ Strongly Disagree
- ◯ Disagree
- ◯ Somewhat Disagree
- ◯ Neither Agree Nor Disagree
- ◯ Somewhat Agree
- ◯ Agree
- ◯ Strongly Agree

8) I have people that will help me if I run into problems taking my medications regularly.

- ◯ Strongly Disagree
- ◯ Disagree
- ◯ Somewhat Disagree
- ◯ Neither Agree Nor Disagree
- ◯ Somewhat Agree
- ◯ Agree
- ◯ Strongly Agree

9) I am reluctant to take medications because of the costs.

- ◯ Strongly Disagree
- ◯ Disagree
- ◯ Somewhat Disagree
- ◯ Neither Agree Nor Disagree
- ◯ Somewhat Agree
- ◯ Agree
- ◯ Strongly Agree

10) I am willing to take medications because of my relationship with my psychiatrist.

- ◯ Strongly Disagree
- ◯ Disagree
- ◯ Somewhat Disagree
- ◯ Neither Agree Nor Disagree
- ◯ Somewhat Agree
- ◯ Agree
- ◯ Strongly Agree

11) People with bipolar disorder who stay well take medications.

- ◯ Strongly Disagree
- ◯ Disagree
- ◯ Somewhat Disagree
- ◯ Neither Agree Nor Disagree
- ◯ Somewhat Agree
- ◯ Agree
- ◯ Strongly Agree

12) I have a plan to help me take my medications as prescribed.

- ◯ Strongly Disagree
- ◯ Disagree
- ◯ Somewhat Disagree
- ◯ Neither Agree Nor Disagree
- ◯ Somewhat Agree
- ◯ Agree
- ◯ Strongly Agree

13) Taking medications reduces my risk of having a mood episode.

- ◯ Strongly Disagree
- ◯ Disagree
- ◯ Somewhat Disagree
- ◯ Neither Agree Nor Disagree
- ◯ Somewhat Agree
- ◯ Agree
- ◯ Strongly Agree

14) I have been able to take my medications as prescribed for extended periods of time.

- ◯ Strongly Disagree
- ◯ Disagree
- ◯ Somewhat Disagree
- ◯ Neither Agree Nor Disagree
- ◯ Somewhat Agree
- ◯ Agree
- ◯ Strongly Agree

15) When I notice I am not taking my medications as prescribed, I adjust what I am doing to get back on track.

- ◯ Strongly Disagree
- ◯ Disagree
- ◯ Somewhat Disagree
- ◯ Neither Agree Nor Disagree
- ◯ Somewhat Agree
- ◯ Agree
- ◯ Strongly Agree

16) It is hard to remember to take my medications at the right times.

- ◯ Strongly Disagree
- ◯ Disagree
- ◯ Somewhat Disagree
- ◯ Neither Agree Nor Disagree
- ◯ Somewhat Agree
- ◯ Agree
- ◯ Strongly Agree

17) I am confident that I can take my medications even when difficult situations arise.

- ◯ Strongly Disagree
- ◯ Disagree
- ◯ Somewhat Disagree
- ◯ Neither Agree Nor Disagree
- ◯ Somewhat Agree
- ◯ Agree
- ◯ Strongly Agree

18) I am confident that I could get back to taking my medications even if I stopped taking them for awhile.

- ◯ Strongly Disagree
- ◯ Disagree
- ◯ Somewhat Disagree
- ◯ Neither Agree Nor Disagree
- ◯ Somewhat Agree
- ◯ Agree
- ◯ Strongly Agree

19) When I take my medications regularly I feel good and stable.

- ◯ Strongly Disagree
- ◯ Disagree
- ◯ Somewhat Disagree
- ◯ Neither Agree Nor Disagree
- ◯ Somewhat Agree
- ◯ Agree
- ◯ Strongly Agree

20) I am capable of taking medications daily without missing any doses.

- ◯ Strongly Disagree
- ◯ Disagree
- ◯ Somewhat Disagree
- ◯ Neither Agree Nor Disagree
- ◯ Somewhat Agree
- ◯ Agree
- ◯ Strongly Agree

21) Most people I know would be in favor of my taking medications.

- ◯ Strongly Disagree
- ◯ Disagree
- ◯ Somewhat Disagree
- ◯ Neither Agree Nor Disagree
- ◯ Somewhat Agree
- ◯ Agree
- ◯ Strongly Agree

22) I pay attention to whether or not I take my medications as prescribed.

- ◯ Strongly Disagree
- ◯ Disagree
- ◯ Somewhat Disagree
- ◯ Neither Agree Nor Disagree
- ◯ Somewhat Agree
- ◯ Agree
- ◯ Strongly Agree

23) Being well for several months makes me consider coming off my medications.

- ◯ Strongly Disagree
- ◯ Disagree
- ◯ Somewhat Disagree
- ◯ Neither Agree Nor Disagree
- ◯ Somewhat Agree
- ◯ Agree
- ◯ Strongly Agree

24) I doubt that my bipolar disorder is serious enough for me to take medications.

- ◯ Strongly Disagree
- ◯ Disagree
- ◯ Somewhat Disagree
- ◯ Neither Agree Nor Disagree
- ◯ Somewhat Agree
- ◯ Agree
- ◯ Strongly Agree

25) I have a plan for taking my medications when problems make it difficult.

- ◯ Strongly Disagree
- ◯ Disagree
- ◯ Somewhat Disagree
- ◯ Neither Agree Nor Disagree
- ◯ Somewhat Agree
- ◯ Agree
- ◯ Strongly Agree

26) I don't feel like myself when I take medications.

- ◯ Strongly Disagree
- ◯ Disagree
- ◯ Somewhat Disagree
- ◯ Neither Agree Nor Disagree
- ◯ Somewhat Agree
- ◯ Agree
- ◯ Strongly Agree

27) I intend to take all my medications without missing any doses.

- ◯ Strongly Disagree
- ◯ Disagree
- ◯ Somewhat Disagree
- ◯ Neither Agree Nor Disagree
- ◯ Somewhat Agree
- ◯ Agree
- ◯ Strongly Agree

Determinants	Subtypes	Question	QN	RM
Support		I have people that will help me if I run into problems taking my medications regularly.	8	1
Knowledge		I know the basics about bipolar disorder and medications for bipolar disorder.	4	1
Norms	Injunctive	I am willing to take medications because of my relationship with my psychiatrist.	10	1
		Most people I know would be in favor of my taking medications.	21	1
	Descriptive	People with bipolar disorder who stay well take medications.	11	1
Perceptions	Risk susceptibility	My risk for mood episodes is high enough to offset the trouble of taking medications.	2	1
		Being well for several months makes me consider coming off my medications.	23	-1
	Risk severity	Problems caused by my mood episodes are bad enough for me to take medications.	6	1
		I doubt that my bipolar disorder is serious enough for me to take medications.	24	-1
Attitudes	Positive instrumental	Taking medications reduces my risk of having a mood episode.	13	1
		I take medications to reduce my risk of being hospitalized.	5	1
	Negative instrumental	I am reluctant to take medications because of the costs.	9	-1
		It is hard to remember to take my medications at the right times.	16	-1
	Positive affective	When I take my medications regularly I feel good and stable.	19	1
		When I take my medications regularly I feel good about my relationships.	1	1
	Negative affective	I don't feel like myself when I take medications.	26	-1
		The unpleasant effects of medications are always present.	7	-1
Self-Efficacy	Task	I am capable of taking medications daily without missing any doses.	20	1
	Coping	I am confident that I can take my medications even when difficult situations arise.	17	1
		I am confident that I could get back to taking my medications even if I stopped taking them for awhile.	18	1
Intention		I intend to take all my medications without missing any doses.	27	1
Goal Setting		My goal is to learn and do what I need to so I can take my medications as prescribed.	3	1
Planning	Task	I have a plan to help me take my medications as prescribed.	12	1
	Coping	I have a plan for taking my medications when problems make it difficult.	25	1
Monitoring & Evaluation		I pay attention to whether or not I take my medications as prescribed.	22	1
Evaluation & Adjustment		When I notice I am not taking my medications as prescribed, I adjust what I am doing to get back on track.	15	1
**Past performance**		I have been able to take my medications as prescribed for extended periods of time.	14	1

QN - question number

RM - reverse multiplier

Past performance is not a determinant but is expected to predict future performance

The questions in this section refer to the recommended sleep duration ranges for healthy adults.

When answering, please note that "as recommended" means 7-9 hours of sleep per day (with 6-10 being okay for some people). This is the duration recommended by the American National Sleep Foundation for adults 18 and older.

28) When I sleep as recommended I feel good and stable.

- ◯ Strongly Disagree
- ◯ Disagree
- ◯ Somewhat Disagree
- ◯ Neither Agree Nor Disagree
- ◯ Somewhat Agree
- ◯ Agree
- ◯ Strongly Agree

29) Sleeping as recommended reduces my risk of having a mood episode.

- ◯ Strongly Disagree
- ◯ Disagree
- ◯ Somewhat Disagree
- ◯ Neither Agree Nor Disagree
- ◯ Somewhat Agree
- ◯ Agree
- ◯ Strongly Agree

30) I know the basics about bipolar disorder and the impact of sleep on symptoms.

- ◯ Strongly Disagree
- ◯ Disagree
- ◯ Somewhat Disagree
- ◯ Neither Agree Nor Disagree
- ◯ Somewhat Agree
- ◯ Agree
- ◯ Strongly Agree

31) I have a concrete plan for how to sleep as recommended.

- ◯ Strongly Disagree
- ◯ Disagree
- ◯ Somewhat Disagree
- ◯ Neither Agree Nor Disagree
- ◯ Somewhat Agree
- ◯ Agree
- ◯ Strongly Agree

32) I have been able to sleep as recommended for extended periods of time in the past.

- ◯ Strongly Disagree
- ◯ Disagree
- ◯ Somewhat Disagree
- ◯ Neither Agree Nor Disagree
- ◯ Somewhat Agree
- ◯ Agree
- ◯ Strongly Agree

33) I am confident that I can sleep as recommended.

- ◯ Strongly Disagree
- ◯ Disagree
- ◯ Somewhat Disagree
- ◯ Neither Agree Nor Disagree
- ◯ Somewhat Agree
- ◯ Agree
- ◯ Strongly Agree

34) I am confident that I can return to sleeping as recommended even if I don't for awhile.

- ◯ Strongly Disagree
- ◯ Disagree
- ◯ Somewhat Disagree
- ◯ Neither Agree Nor Disagree
- ◯ Somewhat Agree
- ◯ Agree
- ◯ Strongly Agree

35) My psychiatrist thinks I should sleep as recommended.

- ◯ Strongly Disagree
- ◯ Disagree
- ◯ Somewhat Disagree
- ◯ Neither Agree Nor Disagree
- ◯ Somewhat Agree
- ◯ Agree
- ◯ Strongly Agree

36) I will have less freedom to do what I want if I sleep as recommended.

- ◯ Strongly Disagree
- ◯ Disagree
- ◯ Somewhat Disagree
- ◯ Neither Agree Nor Disagree
- ◯ Somewhat Agree
- ◯ Agree
- ◯ Strongly Agree

37) When I notice I am not sleeping as recommended, I adjust what I am doing to get back on track.

- ◯ Strongly Disagree
- ◯ Disagree
- ◯ Somewhat Disagree
- ◯ Neither Agree Nor Disagree
- ◯ Somewhat Agree
- ◯ Agree
- ◯ Strongly Agree

38) I have a plan for what to do if difficult situations make it hard to sleep as recommended.

- ◯ Strongly Disagree
- ◯ Disagree
- ◯ Somewhat Disagree
- ◯ Neither Agree Nor Disagree
- ◯ Somewhat Agree
- ◯ Agree
- ◯ Strongly Agree

39) My risk for mood episodes is high enough to offset the trouble of sleeping as recommended.

- ◯ Strongly Disagree
- ◯ Disagree
- ◯ Somewhat Disagree
- ◯ Neither Agree Nor Disagree
- ◯ Somewhat Agree
- ◯ Agree
- ◯ Strongly Agree

40) Problems caused by my mood episodes are bad enough for me to sleep as recommended.

- ◯ Strongly Disagree
- ◯ Disagree
- ◯ Somewhat Disagree
- ◯ Neither Agree Nor Disagree
- ◯ Somewhat Agree
- ◯ Agree
- ◯ Strongly Agree

41) I have family or friends who think I should sleep as recommended.

- ◯ Strongly Disagree
- ◯ Disagree
- ◯ Somewhat Disagree
- ◯ Neither Agree Nor Disagree
- ◯ Somewhat Agree
- ◯ Agree
- ◯ Strongly Agree

42) I have people that will help me if I run into problems consistently sleeping as recommended.

- ◯ Strongly Disagree
- ◯ Disagree
- ◯ Somewhat Disagree
- ◯ Neither Agree Nor Disagree
- ◯ Somewhat Agree
- ◯ Agree
- ◯ Strongly Agree

43) Paying attention to sleeping as recommended is annoying.

- ◯ Strongly Disagree
- ◯ Disagree
- ◯ Somewhat Disagree
- ◯ Neither Agree Nor Disagree
- ◯ Somewhat Agree
- ◯ Agree
- ◯ Strongly Agree

44) My goal is to learn and do what I need to so I can sleep as recommended.

- ◯ Strongly Disagree
- ◯ Disagree
- ◯ Somewhat Disagree
- ◯ Neither Agree Nor Disagree
- ◯ Somewhat Agree
- ◯ Agree
- ◯ Strongly Agree

45) I pay attention to whether or not I am sleeping as recommended.

- ◯ Strongly Disagree
- ◯ Disagree
- ◯ Somewhat Disagree
- ◯ Neither Agree Nor Disagree
- ◯ Somewhat Agree
- ◯ Agree
- ◯ Strongly Agree

46) I am confident that I can sleep as recommended even when problems make it difficult.

- ◯ Strongly Disagree
- ◯ Disagree
- ◯ Somewhat Disagree
- ◯ Neither Agree Nor Disagree
- ◯ Somewhat Agree
- ◯ Agree
- ◯ Strongly Agree

47) People with bipolar disorder who stay well take care to get enough sleep each night.

- ◯ Strongly Disagree
- ◯ Disagree
- ◯ Somewhat Disagree
- ◯ Neither Agree Nor Disagree
- ◯ Somewhat Agree
- ◯ Agree
- ◯ Strongly Agree

48) I intend to sleep as recommended.

◯ Strongly Disagree
◯ Disagree
◯ Somewhat Disagree
◯ Neither Agree Nor Disagree
◯ Somewhat Agree
◯ Agree
◯ Strongly Agree

49) I don't worry about sleeping as recommended after I have been well for several months.

◯ Strongly Disagree
◯ Disagree
◯ Somewhat Disagree
◯ Neither Agree Nor Disagree
◯ Somewhat Agree
◯ Agree
◯ Strongly Agree

50) I doubt that my bipolar disorder is serious enough for me to sleep as recommended.

◯ Strongly Disagree
◯ Disagree
◯ Somewhat Disagree
◯ Neither Agree Nor Disagree
◯ Somewhat Agree
◯ Agree
◯ Strongly Agree

Determinants	Subtypes	Question	QN	RM
Support		I have people that will help me if I run into problems consistently sleeping as recommended.	42	1
Knowledge		I know the basics about bipolar disorder and the impact of sleep on symptoms.	30	1
Norms	Injunctive	My psychiatrist thinks I should sleep as recommended.	35	1
		I have family or friends who think I should sleep as recommended.	41	1
	Descriptive	People with bipolar disorder who stay well take care to get enough sleep each night.	47	1
Perceptions	Risk susceptibility	My risk for mood episodes is high enough to offset the trouble of sleeping as recommended.	39	1
		I don't worry about sleeping as recommended after I have been well for several months.	49	-1
	Risk severity	Problems caused by my mood episodes are bad enough for me to sleep as recommended.	40	1
		I doubt that my bipolar disorder is serious enough for me to sleep as recommended.	50	-1
Attitudes	Positive instrumental	Sleeping as recommended reduces my risk of having a mood episode.	29	1
	Negative instrumental	I will have less freedom to do what I want if I sleep as recommended.	36	-1
	Positive affective	When I sleep as recommended I feel good and stable.	28	1
	Negative affective	Paying attention to sleeping as recommended is annoying.	43	-1
Self-Efficacy	Task	I am confident that I can sleep as recommended.	33	1
	Coping	I am confident that I can sleep as recommended even when problems make it difficult.	46	1
		I am confident that I can return to sleeping as recommended even if I don't for awhile.	34	1
Intention		I intend to sleep as recommended.	48	1
Goal Setting		My goal is to learn and do what I need to so I can sleep as recommended.	44	1
Planning	Task	I have a concrete plan for how to sleep as recommended.	31	1
	Coping	I have a plan for what to do if difficult situations make it hard to sleep as recommended.	38	1
Monitoring & Evaluation		I pay attention to whether or not I am sleeping as recommended.	45	1
Evaluation & Adjustment		When I notice I am not sleeping as recommended, I adjust what I am doing to get back on track.	37	1

**Past performance**		I have been able to sleep as recommended for extended periods of time in the past.	32	1

QN - question number

RM - reverse multiplier

Past performance is not a determinant but is expected to predict future performance

The questions in this section refer to keeping a regular routine. A regular routine consists of doing the following about the same time every day: 1) getting up, 2) first contact with another person (in person or by phone), 3) starting work/school/daily responsibilities, 4) eating dinner, and 5) going to bed.

When answering, please note that "routine" refers to those five events, or anchors, outlined above occurring at the same time every day (+/- 45 minutes).

51) My psychiatrist thinks that I should have a regular routine.

- ◯ Strongly Disagree
- ◯ Disagree
- ◯ Somewhat Disagree
- ◯ Neither Agree Nor Disagree
- ◯ Somewhat Agree
- ◯ Agree
- ◯ Strongly Agree

52) Most people I know would be in favor of me keeping a routine.

- ◯ Strongly Disagree
- ◯ Disagree
- ◯ Somewhat Disagree
- ◯ Neither Agree Nor Disagree
- ◯ Somewhat Agree
- ◯ Agree
- ◯ Strongly Agree

53) I know the basics about bipolar disorder and the impact of daily routines on symptoms.

- ◯ Strongly Disagree
- ◯ Disagree
- ◯ Somewhat Disagree
- ◯ Neither Agree Nor Disagree
- ◯ Somewhat Agree
- ◯ Agree
- ◯ Strongly Agree

54) Problems caused by my mood episodes are bad enough for me to keep a routine.

- ◯ Strongly Disagree
- ◯ Disagree
- ◯ Somewhat Disagree
- ◯ Neither Agree Nor Disagree
- ◯ Somewhat Agree
- ◯ Agree
- ◯ Strongly Agree

55) I am confident that I can keep a routine.

- ◯ Strongly Disagree
- ◯ Disagree
- ◯ Somewhat Disagree
- ◯ Neither Agree Nor Disagree
- ◯ Somewhat Agree
- ◯ Agree
- ◯ Strongly Agree

56) I have been able to keep a routine for extended periods of time in the past.

- ◯ Strongly Disagree
- ◯ Disagree
- ◯ Somewhat Disagree
- ◯ Neither Agree Nor Disagree
- ◯ Somewhat Agree
- ◯ Agree
- ◯ Strongly Agree

57) Having a routine lowers my risk for a mood episode.

- ◯ Strongly Disagree
- ◯ Disagree
- ◯ Somewhat Disagree
- ◯ Neither Agree Nor Disagree
- ◯ Somewhat Agree
- ◯ Agree
- ◯ Strongly Agree

58) I will have less freedom to do what I want if I keep a routine.

- ◯ Strongly Disagree
- ◯ Disagree
- ◯ Somewhat Disagree
- ◯ Neither Agree Nor Disagree
- ◯ Somewhat Agree
- ◯ Agree
- ◯ Strongly Agree

59) I have a plan for how to cope with possible setbacks to my routine.

- ◯ Strongly Disagree
- ◯ Disagree
- ◯ Somewhat Disagree
- ◯ Neither Agree Nor Disagree
- ◯ Somewhat Agree
- ◯ Agree
- ◯ Strongly Agree

60) My goal is to learn and do what I need to so I can maintain a routine.

- ◯ Strongly Disagree
- ◯ Disagree
- ◯ Somewhat Disagree
- ◯ Neither Agree Nor Disagree
- ◯ Somewhat Agree
- ◯ Agree
- ◯ Strongly Agree

61) I am certain that I can maintain a routine even when problems make it difficult.

- ◯ Strongly Disagree
- ◯ Disagree
- ◯ Somewhat Disagree
- ◯ Neither Agree Nor Disagree
- ◯ Somewhat Agree
- ◯ Agree
- ◯ Strongly Agree

62) My risk of having a mood episode is high enough to offset the trouble of keeping a routine.

- ◯ Strongly Disagree
- ◯ Disagree
- ◯ Somewhat Disagree
- ◯ Neither Agree Nor Disagree
- ◯ Somewhat Agree
- ◯ Agree
- ◯ Strongly Agree

63) I doubt my bipolar disorder is sufficiently serious to justify keeping a routine.

- ◯ Strongly Disagree
- ◯ Disagree
- ◯ Somewhat Disagree
- ◯ Neither Agree Nor Disagree
- ◯ Somewhat Agree
- ◯ Agree
- ◯ Strongly Agree

64) I have people that will help me if I run into problems keeping a routine.

◯ Strongly Disagree
◯ Disagree
◯ Somewhat Disagree
◯ Neither Agree Nor Disagree
◯ Somewhat Agree
◯ Agree
◯ Strongly Agree

65) I watch carefully that I keep a routine.

◯ Strongly Disagree
◯ Disagree
◯ Somewhat Disagree
◯ Neither Agree Nor Disagree
◯ Somewhat Agree
◯ Agree
◯ Strongly Agree

66) I have a concrete plan for keeping a routine.

◯ Strongly Disagree
◯ Disagree
◯ Somewhat Disagree
◯ Neither Agree Nor Disagree
◯ Somewhat Agree
◯ Agree
◯ Strongly Agree

67) I am confident that I can return to a routine even if my routine is disrupted for awhile.

◯ Strongly Disagree
◯ Disagree
◯ Somewhat Disagree
◯ Neither Agree Nor Disagree
◯ Somewhat Agree
◯ Agree
◯ Strongly Agree

68) Having a routine is boring.

◯ Strongly Disagree
◯ Disagree
◯ Somewhat Disagree
◯ Neither Agree Nor Disagree
◯ Somewhat Agree
◯ Agree
◯ Strongly Agree

69) People with bipolar disorder who stay well take care to keep a routine.

◯ Strongly Disagree
◯ Disagree
◯ Somewhat Disagree
◯ Neither Agree Nor Disagree
◯ Somewhat Agree
◯ Agree
◯ Strongly Agree

70) I intend to keep a routine.

◯ Strongly Disagree
◯ Disagree
◯ Somewhat Disagree
◯ Neither Agree Nor Disagree
◯ Somewhat Agree
◯ Agree
◯ Strongly Agree

71) When I notice I am not keeping a routine, I adjust what I am doing to get back on track.
- ◯ Strongly Disagree
- ◯ Disagree
- ◯ Somewhat Disagree
- ◯ Neither Agree Nor Disagree
- ◯ Somewhat Agree
- ◯ Agree
- ◯ Strongly Agree

72) I don't worry about keeping a routine when I am well for several months.
- ◯ Strongly Disagree
- ◯ Disagree
- ◯ Somewhat Disagree
- ◯ Neither Agree Nor Disagree
- ◯ Somewhat Agree
- ◯ Agree
- ◯ Strongly Agree

73) When I keep a routine I feel good and stable.
- ◯ Strongly Disagree
- ◯ Disagree
- ◯ Somewhat Disagree
- ◯ Neither Agree Nor Disagree
- ◯ Somewhat Agree
- ◯ Agree
- ◯ Strongly Agree

Determinants	Subtypes	Question	QN	RM
Support		I have people that will help me if I run into problems keeping a routine.	64	1
Knowledge		I know the basics about bipolar disorder and the impact of daily routines on symptoms.	53	1
Norms	Injunctive	My psychiatrist thinks that I should have a regular routine.	51	1
		Most people I know would be in favor of me keeping a routine.	52	1
	Descriptive	People with bipolar disorder who stay well take care to keep a routine.	69	1
Perceptions	Risk susceptibility	My risk of having a mood episode is high enough to offset the trouble of keeping a routine.	62	1
		I don't worry about keeping a routine when I am well for several months.	72	-1
	Risk severity	Problems caused by my mood episodes are bad enough for me to keep a routine.	54	1
		I doubt my bipolar disorder is sufficiently serious to justify keeping a routine.	63	-1
Attitudes	Positive instrumental	Having a routine lowers my risk for a mood episode.	57	1
	Negative instrumental	I will have less freedom to do what I want if I keep a routine.	58	-1
	Positive affective	When I keep a routine I feel good and stable.	73	1
	Negative affective	Having a routine is boring.	68	-1
Self-Efficacy	Task	I am confident that I can keep a routine.	55	1
	Coping	I am certain that I can maintain a routine even when problems make it difficult.	61	1
		I am confident that I can return to a routine even if my routine is disrupted for awhile.	67	1
Intention		I intend to keep a routine.	70	1
Goal Setting		My goal is to learn and do what I need to so I can maintain a routine.	60	1
Planning	Task	I have a concrete plan for keeping a routine.	66	1
	Coping	I have a plan for how to cope with possible setbacks to my routine.	59	1
Monitoring & Evaluation		I watch carefully that I keep a routine.	65	1
Evaluation & Adjustment		When I notice I am not keeping a routine, I adjust what I am doing to get back on track.	71	1

**Past performance**		I have been able to keep a routine for extended periods of time in the past.	56	1

QN - question number

RM - reverse multiplier

Past performance is not a determinant but is expected to predict future performance

The questions in this section address managing early warning signs of mania and depression. Early warning signs are the first symptoms or your own unique signs that occur before entering a manic or depressive episode.

Managing early warning signs has three parts: identifying what your early warning signs are, working to spot them, and then taking action on your plan when they occur. Please consider all three aspects carefully when answering.

74) I am confident that I can manage early warning signs.

- ◯ Strongly Disagree
- ◯ Disagree
- ◯ Somewhat Disagree
- ◯ Neither Agree Nor Disagree
- ◯ Somewhat Agree
- ◯ Agree
- ◯ Strongly Agree

75) My goal is to learn and do what I need to so I can effectively manage early warning signs.

- ◯ Strongly Disagree
- ◯ Disagree
- ◯ Somewhat Disagree
- ◯ Neither Agree Nor Disagree
- ◯ Somewhat Agree
- ◯ Agree
- ◯ Strongly Agree

76) Managing early warnings signs helps me feel in control.

- ◯ Strongly Disagree
- ◯ Disagree
- ◯ Somewhat Disagree
- ◯ Neither Agree Nor Disagree
- ◯ Somewhat Agree
- ◯ Agree
- ◯ Strongly Agree

77) I intend to manage early warning signs.

- ◯ Strongly Disagree
- ◯ Disagree
- ◯ Somewhat Disagree
- ◯ Neither Agree Nor Disagree
- ◯ Somewhat Agree
- ◯ Agree
- ◯ Strongly Agree

78) I don't worry about managing early warning signs when I am well for several months.

- ◯ Strongly Disagree
- ◯ Disagree
- ◯ Somewhat Disagree
- ◯ Neither Agree Nor Disagree
- ◯ Somewhat Agree
- ◯ Agree
- ◯ Strongly Agree

79) I am confident that I can return to managing early warning signs even if I stop for awhile.

- ◯ Strongly Disagree
- ◯ Disagree
- ◯ Somewhat Disagree
- ◯ Neither Agree Nor Disagree
- ◯ Somewhat Agree
- ◯ Agree
- ◯ Strongly Agree

80) When I notice I am not actively managing early warning signs, I adjust what I'm doing and get back on track.

- ◯ Strongly Disagree
- ◯ Disagree
- ◯ Somewhat Disagree
- ◯ Neither Agree Nor Disagree
- ◯ Somewhat Agree
- ◯ Agree
- ◯ Strongly Agree

81) I track whether or not I am managing early warning signs.

- ◯ Strongly Disagree
- ◯ Disagree
- ◯ Somewhat Disagree
- ◯ Neither Agree Nor Disagree
- ◯ Somewhat Agree
- ◯ Agree
- ◯ Strongly Agree

82) I have people that will help me if I have problems managing early warning signs.

- ◯ Strongly Disagree
- ◯ Disagree
- ◯ Somewhat Disagree
- ◯ Neither Agree Nor Disagree
- ◯ Somewhat Agree
- ◯ Agree
- ◯ Strongly Agree

83) My risk of having a mood episode is high enough to make managing early warning signs worthwhile.

- ◯ Strongly Disagree
- ◯ Disagree
- ◯ Somewhat Disagree
- ◯ Neither Agree Nor Disagree
- ◯ Somewhat Agree
- ◯ Agree
- ◯ Strongly Agree

84) I doubt my bipolar disorder is sufficiently serious to justify the effort required to manage early warning signs.

- ◯ Strongly Disagree
- ◯ Disagree
- ◯ Somewhat Disagree
- ◯ Neither Agree Nor Disagree
- ◯ Somewhat Agree
- ◯ Agree
- ◯ Strongly Agree

85) I am certain that I can manage early warning signs even when problems make it difficult.

- ◯ Strongly Disagree
- ◯ Disagree
- ◯ Somewhat Disagree
- ◯ Neither Agree Nor Disagree
- ◯ Somewhat Agree
- ◯ Agree
- ◯ Strongly Agree

86) Managing early warning signs is stressful.

- ◯ Strongly Disagree
- ◯ Disagree
- ◯ Somewhat Disagree
- ◯ Neither Agree Nor Disagree
- ◯ Somewhat Agree
- ◯ Agree
- ◯ Strongly Agree

87) I have a plan for coping with setbacks to managing early warning signs.

- ◯ Strongly Disagree
- ◯ Disagree
- ◯ Somewhat Disagree
- ◯ Neither Agree Nor Disagree
- ◯ Somewhat Agree
- ◯ Agree
- ◯ Strongly Agree

88) Problems caused by my mood episodes are bad enough for me to manage early warning signs.

- ◯ Strongly Disagree
- ◯ Disagree
- ◯ Somewhat Disagree
- ◯ Neither Agree Nor Disagree
- ◯ Somewhat Agree
- ◯ Agree
- ◯ Strongly Agree

89) People with bipolar disorder who stay well take care to manage early warning signs.

- ◯ Strongly Disagree
- ◯ Disagree
- ◯ Somewhat Disagree
- ◯ Neither Agree Nor Disagree
- ◯ Somewhat Agree
- ◯ Agree
- ◯ Strongly Agree

90) My psychiatrist thinks that I should manage early warning signs.

- ◯ Strongly Disagree
- ◯ Disagree
- ◯ Somewhat Disagree
- ◯ Neither Agree Nor Disagree
- ◯ Somewhat Agree
- ◯ Agree
- ◯ Strongly Agree

91) I have a concrete plan for how I will manage early warning signs.

- ◯ Strongly Disagree
- ◯ Disagree
- ◯ Somewhat Disagree
- ◯ Neither Agree Nor Disagree
- ◯ Somewhat Agree
- ◯ Agree
- ◯ Strongly Agree

92) My friends and family think I should manage my early warning signs.

- ◯ Strongly Disagree
- ◯ Disagree
- ◯ Somewhat Disagree
- ◯ Neither Agree Nor Disagree
- ◯ Somewhat Agree
- ◯ Agree
- ◯ Strongly Agree

93) Managing early warning signs lowers my risk for a mood episode.

- ◯ Strongly Disagree
- ◯ Disagree
- ◯ Somewhat Disagree
- ◯ Neither Agree Nor Disagree
- ◯ Somewhat Agree
- ◯ Agree
- ◯ Strongly Agree

94) Managing early warning signs requires too much effort.

- ◯ Strongly Disagree
- ◯ Disagree
- ◯ Somewhat Disagree
- ◯ Neither Agree Nor Disagree
- ◯ Somewhat Agree
- ◯ Agree
- ◯ Strongly Agree

95) I know the basics about bipolar disorder and how to manage early warning signs.

- ◯ Strongly Disagree
- ◯ Disagree
- ◯ Somewhat Disagree
- ◯ Neither Agree Nor Disagree
- ◯ Somewhat Agree
- ◯ Agree
- ◯ Strongly Agree

96) I have been able to manage early warning signs for extended periods of time in the past.

- ◯ Strongly Disagree
- ◯ Disagree
- ◯ Somewhat Disagree
- ◯ Neither Agree Nor Disagree
- ◯ Somewhat Agree
- ◯ Agree
- ◯ Strongly Agree

Determinants	Subtypes	Question	QN	RM
Support		I have people that will help me if I have problems managing early warning signs.	82	1
Knowledge		I know the basics about bipolar disorder and how to manage early warning signs.	95	1
Norms	Injunctive	My psychiatrist thinks that I should manage early warning signs.	90	1
		My friends and family think I should manage my early warning signs.	92	1
	Descriptive	People with bipolar disorder who stay well take care to manage early warning signs.	89	1
Perceptions	Risk susceptibility	My risk of having a mood episode is high enough to make managing early warning signs worthwhile.	83	1
		I don't worry about managing early warning signs when I am well for several months.	78	-1
	Risk severity	Problems caused by my mood episodes are bad enough for me to manage early warning signs.	88	1
		I doubt my bipolar disorder is sufficiently serious to justify the effort required to manage early warning signs.	84	-1
Attitudes	Positive instrumental	Managing early warning signs lowers my risk for a mood episode.	93	1
	Negative instrumental	Mangaging early warning signs requires too much effort.	94	-1
	Positive affective	Managing early warnings signs helps me feel in control.	76	1
	Negative affective	Managing early warning signs is stressful.	86	-1
Self-Efficacy	Task	I am confident that I can manage early warning signs.	74	1
	Coping	I am certain that I can manage early warning signs even when problems make it difficult.	85	1
		I am confident that I can return to managing early warning signs even if I stop for awhile.	79	1
Intention		I intend to manage early warning signs.	77	1
Goal Setting		My goal is to learn and do what I need to so I can effectively manage early warning signs.	75	1
Planning	Task	I have a concrete plan for how I will manage early warning signs.	91	1
	Coping	I have a plan for coping with setbacks to managing early warning signs.	87	1
Monitoring & Evaluation		I track whether or not I am managing early warning signs.	81	1
Evaluation & Adjustment		When I notice I am not actively managing early warning signs, I adjust what I'm doing and get back on track.	80	1
Past performance		I have been able to manage early warning signs for extended periods of time in the past.	96	1

QN - question number

RM - reverse multiplier

Past performance is not a determinant but is expected to predict future performance

Scoring

Scale	
Strongly disagree	-3
Disagree	-2
Somewhat disagree	-1
Neither agree nor disagree	0
Somewhat agree	1
Agree	2
Strongly agree	3

I. Convert responses

A. Multiple responses for each question by reverse multiplier

B. Add 3 to responses for each question to convert question response range to 0-6

II. For total scores and sub-scores for each target

A. Sum converted responses for questions

B. Divide by maximum possible score and multiple by 100

C. Range 0-100 for all scales and sub-scales for each target

III. Total scores for each target

A. Sum of all questions except for past performance

B. Medication adherence

1. Number of questions: 26
2. Maximum possible score: 156

C. Sleep duration

1. Number of questions: 22
2. Maximum possible score: 132

D. Routine regularity

1. Number of questions: 22
2. Maximum possible score: 132

E. Manage signs and symptoms

1. Number of questions: 22
2. Maximum possible score: 132

IV. Support and Past performance scores for all targets

A. Number of questions: 1

B. Maximum possible score: 6

V. Motivation scores for each target

A. Sum all questions for Knowledge, Norms, Perceptions, Attitudes, Self-Efficacy, and Intention

B. Medication adherence

1. Number of questions: 20

2. Maximum possible score: 120

C. Sleep duration

1. Number of questions: 16

2. Maximum possible score: 96

D. Routine regularity

1. Number of questions: 16

2. Maximum possible score: 96

E. Manage signs and symptoms

1. Number of questions: 16

2. Maximum possible score: 96

VI. Volition scores for all targets

A. Sum all questions for Goal Setting, Planning, Monitoring & Evaluation, and Evaluation & Adjustment

B. Number of questions: 5

D. Maximum possible score: 30
